# Supplementary material for: Iterative improvement in the automatic modular design of robot swarms
Source: PeerJ Comput Sci. 2020 Dec 7;6:e322. doi: 10.7717/peerj-cs.322 (PMC7924708; doi:10.7717/peerj-cs.322)
Supplement: Supplemental Information 3 [file peerj-cs-06-322-s003.zip › argos3/doc/api/standalone/a00300_source.html]

ARGoS: core/control\_interface/ci\_controller.cpp Source File


- Main Page
- Related Pages
- Namespaces
- Classes
- Files

- File List
- File Members

# core/control\_interface/ci\_controller.cpp

Go to the documentation of this file.

```
00001 
00007 #include "ci_controller.h"
00008 
00009 namespace argos {
00010 
00011    /****************************************/
00012    /****************************************/
00013 
00014    CCI_Controller::~CCI_Controller() {
00015       /* Delete actuators*/
00016       for(CCI_Actuator::TMap::iterator itActuators = m_mapActuators.begin();
00017           itActuators != m_mapActuators.end();
00018           ++itActuators) {
00019          delete itActuators->second;
00020       }
00021       m_mapActuators.clear();
00022 
00023       /* Delete sensors */
00024       for(CCI_Sensor::TMap::iterator itSensors = m_mapSensors.begin();
00025           itSensors != m_mapSensors.end();
00026           ++itSensors) {
00027          delete itSensors->second;
00028       }
00029       m_mapSensors.clear();
00030    }
00031 
00032    /****************************************/
00033    /****************************************/
00034 
00035    bool CCI_Controller::HasActuator(const std::string& str_actuator_type) const {
00036       CCI_Actuator::TMap::const_iterator it = m_mapActuators.find(str_actuator_type);
00037       return (it != m_mapActuators.end());
00038    }
00039 
00040    /****************************************/
00041    /****************************************/
00042 
00043    bool CCI_Controller::HasSensor(const std::string& str_sensor_type) const {
00044       CCI_Sensor::TMap::const_iterator it = m_mapSensors.find(str_sensor_type);
00045       return (it != m_mapSensors.end());
00046    }
00047 
00048    /****************************************/
00049    /****************************************/
00050 
00051 }
```

---

Generated on 10 Jul 2018 for ARGoS by 
 1.6.1 
