# Supplementary material for: Iterative improvement in the automatic modular design of robot swarms
Source: PeerJ Comput Sci. 2020 Dec 7;6:e322. doi: 10.7717/peerj-cs.322 (PMC7924708; doi:10.7717/peerj-cs.322)
Supplement: Supplemental Information 3 [file peerj-cs-06-322-s003.zip › argos3/doc/api/standalone/a00301.html]

ARGoS: core/control\_interface/ci\_controller.h File Reference


- Main Page
- Related Pages
- Namespaces
- Classes
- Files

- File List
- File Members

# core/control\_interface/ci\_controller.h File Reference

`#include <argos3/core/utility/configuration/base_configurable_resource.h>`  
`#include <argos3/core/utility/datatypes/datatypes.h>`  
`#include <argos3/core/control_interface/ci_sensor.h>`  
`#include <argos3/core/control_interface/ci_actuator.h>`  
`#include <argos3/core/utility/plugins/factory.h>`  
`#include <map>`  
`#include <string>`  
`#include <cxxabi.h>`  
`#include <typeinfo>`  

Include dependency graph for ci\_controller.h:

This graph shows which files directly or indirectly include this file:

Go to the source code of this file.

|  |  |
| --- | --- |
| Classes | |
| class | argos::CCI\_Controller |
|  | The basic interface for a robot controller. More... |
| Namespaces | |
| namespace | argos |

|  |  |
| --- | --- |
|  | The namespace containing all the ARGoS related code. |

| Defines | |
| #define | REGISTER\_CONTROLLER(CLASSNAME, LABEL) |
| Functions | |
| argos::CCI\_Controller \* | ControllerMaker (const std::string &str\_label) |
|  | Registers a new controller inside ARGoS. |

---

## Define Documentation

|  |  |  |
| --- | --- | --- |
| #define REGISTER\_CONTROLLER | ( | CLASSNAME, |
|  |  | LABEL |  | ) |  |

**Value:**

```
extern "C" {                                                         \
      argos::CCI_Controller* ControllerMaker(const std::string& str_label) { \
         if(str_label != LABEL) {                                       \
            THROW_ARGOSEXCEPTION("Controller label \"" <<               \
                                 str_label <<                           \
                                 "\" does not match the registered one: \"" << \
                                 LABEL << "\"");                        \
         }                                                              \
         return new CLASSNAME;                                          \
      }                                                                 \
   }
```

Definition at line 248 of file ci\_controller.h.

---

## Function Documentation

|  |  |  |  |  |  |
| --- | --- | --- | --- | --- | --- |
| argos::CCI\_Controller\* ControllerMaker | ( | const std::string & | *str\_label* | ) |  |

Registers a new controller inside ARGoS.

You must register your controller for ARGoS to be able to recognize it. This statement must be included in a .cpp file. It can't be in a header.

---

Generated on 10 Jul 2018 for ARGoS by 
 1.6.1 
