# Supplementary material for: Iterative improvement in the automatic modular design of robot swarms
Source: PeerJ Comput Sci. 2020 Dec 7;6:e322. doi: 10.7717/peerj-cs.322 (PMC7924708; doi:10.7717/peerj-cs.322)
Supplement: Supplemental Information 3 [file peerj-cs-06-322-s003.zip › argos3/doc/api/standalone/a00301_source.html]

ARGoS: core/control\_interface/ci\_controller.h Source File


- Main Page
- Related Pages
- Namespaces
- Classes
- Files

- File List
- File Members

# core/control\_interface/ci\_controller.h

Go to the documentation of this file.

```
00001 
00007 #ifndef CCI_CONTROLLER_H
00008 #define CCI_CONTROLLER_H
00009 
00010 namespace argos {
00011    class CCI_Controller;
00012 }
00013 
00014 #include <argos3/core/utility/configuration/base_configurable_resource.h>
00015 #include <argos3/core/utility/datatypes/datatypes.h>
00016 #include <argos3/core/control_interface/ci_sensor.h>
00017 #include <argos3/core/control_interface/ci_actuator.h>
00018 #include <argos3/core/utility/plugins/factory.h>
00019 
00020 #include <map>
00021 #include <string>
00022 #include <cxxabi.h>
00023 #include <typeinfo>
00024 
00025 namespace argos {
00026 
00030    class CCI_Controller : public CBaseConfigurableResource {
00031 
00032    public:
00033 
00037       virtual ~CCI_Controller();
00038 
00048       virtual void Init(TConfigurationNode& t_node) {}
00049 
00055       virtual void ControlStep() {}
00056 
00065       virtual void Reset() {}
00066 
00072       virtual void Destroy() {}
00073 
00078       inline const std::string& GetId() const {
00079          return m_strId;
00080       }
00081 
00088       inline void SetId(const std::string& str_id) {
00089          m_strId = str_id;
00090       }
00091 
00105       template<typename ACTUATOR_IMPL>
00106       ACTUATOR_IMPL* GetActuator(const std::string& str_actuator_type) {
00107          CCI_Actuator::TMap::const_iterator it = m_mapActuators.find(str_actuator_type);
00108          if (it != m_mapActuators.end()) {
00109             ACTUATOR_IMPL* pcActuator = dynamic_cast<ACTUATOR_IMPL*>(it->second);
00110             if(pcActuator != NULL) {
00111                return pcActuator;
00112             }
00113             else {
00114                char* pchDemangledType = abi::__cxa_demangle(typeid(ACTUATOR_IMPL).name(), NULL, NULL, NULL);
00115                THROW_ARGOSEXCEPTION("Actuator type " << str_actuator_type << " cannot be cast to type " << pchDemangledType);
00116             }
00117          }
00118          else {
00119             THROW_ARGOSEXCEPTION("Unknown actuator type " << str_actuator_type << " requested in controller. Did you add it to the XML file?");
00120          }
00121       }
00122 
00136       template<typename SENSOR_IMPL>
00137       SENSOR_IMPL* GetSensor(const std::string& str_sensor_type) {
00138          CCI_Sensor::TMap::const_iterator it = m_mapSensors.find(str_sensor_type);
00139          if (it != m_mapSensors.end()) {
00140             SENSOR_IMPL* pcSensor = dynamic_cast<SENSOR_IMPL*>(it->second);
00141             if(pcSensor != NULL) {
00142                return pcSensor;
00143             }
00144             else {
00145                char* pchDemangledType = abi::__cxa_demangle(typeid(SENSOR_IMPL).name(), NULL, NULL, NULL);
00146                THROW_ARGOSEXCEPTION("Sensor type " << str_sensor_type << " cannot be cast to type " << pchDemangledType);
00147             }
00148          }
00149          else {
00150             THROW_ARGOSEXCEPTION("Unknown sensor type " << str_sensor_type << " requested in controller. Did you add it to the XML file?");
00151          }
00152       }
00153 
00160       bool HasActuator(const std::string& str_actuator_type) const;
00161 
00168       bool HasSensor(const std::string& str_sensor_type) const;
00169 
00174       inline CCI_Actuator::TMap& GetAllActuators() {
00175           return m_mapActuators;
00176       }
00177 
00182       inline CCI_Sensor::TMap& GetAllSensors() {
00183           return m_mapSensors;
00184       }
00185 
00193       inline void AddActuator(const std::string& str_actuator_type,
00194                               CCI_Actuator* pc_actuator) {
00195          m_mapActuators[str_actuator_type] = pc_actuator;
00196       }
00197 
00205       inline void AddSensor(const std::string& str_sensor_type,
00206                             CCI_Sensor* pc_sensor) {
00207          m_mapSensors[str_sensor_type] = pc_sensor;
00208       }
00209 
00210    protected:
00211 
00213       CCI_Actuator::TMap m_mapActuators;
00214 
00216       CCI_Sensor::TMap m_mapSensors;
00217 
00219       std::string m_strId;
00220 
00221    };
00222 
00223 }
00224 
00230 #ifdef ARGOS_DYNAMIC_LIBRARY_LOADING
00231 
00232 #define REGISTER_CONTROLLER(CLASSNAME, LABEL) \
00233    REGISTER_SYMBOL(CCI_Controller,            \
00234                    CLASSNAME,                 \
00235                    LABEL,                     \
00236                    "undefined",               \
00237                    "undefined",               \
00238                    "undefined",               \
00239                    "undefined",               \
00240                    "undefined")
00241 
00242 #else
00243 
00244 extern "C" {
00245    extern argos::CCI_Controller* ControllerMaker(const std::string& str_label);
00246 }
00247 
00248 #define REGISTER_CONTROLLER(CLASSNAME, LABEL)                           \
00249    extern "C" {                                                         \
00250       argos::CCI_Controller* ControllerMaker(const std::string& str_label) { \
00251          if(str_label != LABEL) {                                       \
00252             THROW_ARGOSEXCEPTION("Controller label \"" <<               \
00253                                  str_label <<                           \
00254                                  "\" does not match the registered one: \"" << \
00255                                  LABEL << "\"");                        \
00256          }                                                              \
00257          return new CLASSNAME;                                          \
00258       }                                                                 \
00259    }
00260 
00261 #endif
00262 
00263 #endif
```

---

Generated on 10 Jul 2018 for ARGoS by 
 1.6.1 
