# Supplementary material for: Iterative improvement in the automatic modular design of robot swarms
Source: PeerJ Comput Sci. 2020 Dec 7;6:e322. doi: 10.7717/peerj-cs.322 (PMC7924708; doi:10.7717/peerj-cs.322)
Supplement: Supplemental Information 3 [file peerj-cs-06-322-s003.zip › argos3/doc/api/standalone/a00302_source.html]

ARGoS: core/control\_interface/ci\_sensor.h Source File


- Main Page
- Related Pages
- Namespaces
- Classes
- Files

- File List
- File Members

# core/control\_interface/ci\_sensor.h

Go to the documentation of this file.

```
00001 
00009 #ifndef CI_SENSOR_H
00010 #define CI_SENSOR_H
00011 
00012 namespace argos {
00013    class CCI_Sensor;
00014 }
00015 
00016 #include <argos3/core/config.h>
00017 #include <argos3/core/utility/datatypes/datatypes.h>
00018 #include <argos3/core/utility/configuration/base_configurable_resource.h>
00019 #include <map>
00020 
00021 #ifdef ARGOS_WITH_LUA
00022 extern "C" {
00023 #include <lua.h>
00024 #include <lualib.h>
00025 #include <lauxlib.h>
00026 }
00027 #endif
00028 
00029 namespace argos {
00030 
00034    class CCI_Sensor : public CBaseConfigurableResource {
00035 
00036    public:
00037 
00038       typedef std::map<std::string, CCI_Sensor*, std::less<std::string> > TMap;
00039 
00040    public:
00041 
00045       virtual ~CCI_Sensor() {}
00046 
00054       virtual void Init(TConfigurationNode& t_node) {}
00055 
00062       virtual void Reset() {}
00063 
00071       virtual void Destroy() {}
00072 
00073 #ifdef ARGOS_WITH_LUA
00074 
00080       virtual void CreateLuaState(lua_State* pt_lua_state) = 0;
00081 
00087       virtual void ReadingsToLuaState(lua_State* pt_lua_state) = 0;
00088 #endif
00089 
00090    };
00091 
00092 }
00093 
00094 #endif
```

---

Generated on 10 Jul 2018 for ARGoS by 
 1.6.1 
