# Supplementary material for: Iterative improvement in the automatic modular design of robot swarms
Source: PeerJ Comput Sci. 2020 Dec 7;6:e322. doi: 10.7717/peerj-cs.322 (PMC7924708; doi:10.7717/peerj-cs.322)
Supplement: Supplemental Information 3 [file peerj-cs-06-322-s003.zip › argos3/doc/api/standalone/a00304_source.html]

ARGoS: core/simulator/argos\_command\_line\_arg\_parser.cpp Source File


- Main Page
- Related Pages
- Namespaces
- Classes
- Files

- File List
- File Members

# core/simulator/argos\_command\_line\_arg\_parser.cpp

Go to the documentation of this file.

```
00001 
00007 #include "argos_command_line_arg_parser.h"
00008 #include <argos3/core/config.h>
00009 
00010 namespace argos {
00011 
00012    /****************************************/
00013    /****************************************/
00014 
00015    CARGoSCommandLineArgParser::CARGoSCommandLineArgParser() :
00016       m_eAction(ACTION_UNKNOWN),
00017       m_pcInitLogStream(NULL),
00018       m_pcInitLogErrStream(NULL) {
00019       AddFlag(
00020          'h',
00021          "help",
00022          "display this usage information",
00023          m_bHelpWanted
00024          );
00025       AddFlag(
00026          'v',
00027          "version",
00028          "display the current version and release",
00029          m_bVersionWanted
00030          );
00031       AddFlag(
00032          'n',
00033          "no-color",
00034          "do not use colored output [OPTIONAL]",
00035          m_bNonColoredLog
00036          );
00037       AddArgument<std::string>(
00038          'c',
00039          "config-file",
00040          "the experiment XML configuration file",
00041          m_strExperimentConfigFile
00042          );
00043       AddArgument<std::string>(
00044          'q',
00045          "query",
00046          "query the available plugins",
00047          m_strQuery
00048          );
00049       AddArgument<std::string>(
00050          'l',
00051          "log-file",
00052          "output log to file [OPTIONAL]",
00053          m_strLogFileName
00054          );
00055       AddArgument<std::string>(
00056          'e',
00057          "logerr-file",
00058          "output logerr to file [OPTIONAL]",
00059          m_strLogErrFileName
00060          );
00061    }
00062 
00063    /****************************************/
00064    /****************************************/
00065 
00066    CARGoSCommandLineArgParser::~CARGoSCommandLineArgParser() {
00067       if(m_cLogFile.is_open()) {
00068          LOG.GetStream().rdbuf(m_pcInitLogStream);
00069          m_cLogFile.close();
00070       }
00071       if(m_cLogErrFile.is_open()) {
00072          LOGERR.GetStream().rdbuf(m_pcInitLogErrStream);
00073          m_cLogErrFile.close();
00074       }
00075    }
00076 
00077    /****************************************/
00078    /****************************************/
00079 
00080    void CARGoSCommandLineArgParser::Parse(SInt32 n_argc,
00081                                           char** ppch_argv) {
00082       CCommandLineArgParser::Parse(n_argc, ppch_argv);
00083       /* Configure LOG/LOGERR coloring */
00084       if(m_bNonColoredLog) {
00085          LOG.DisableColoredOutput();
00086          LOGERR.DisableColoredOutput();
00087       }
00088 
00089       /* Check whether LOG and LOGERR should go to files */
00090       if(m_strLogFileName != "") {
00091          LOG.DisableColoredOutput();
00092          m_cLogFile.open(m_strLogFileName.c_str(), std::ios::trunc | std::ios::out);
00093          if(m_cLogFile.fail()) {
00094             THROW_ARGOSEXCEPTION("Error opening file \"" << m_strLogFileName << "\"");
00095          }
00096          m_pcInitLogStream = LOG.GetStream().rdbuf();
00097          LOG.GetStream().rdbuf(m_cLogFile.rdbuf());
00098       }
00099       if(m_strLogErrFileName != "") {
00100          LOGERR.DisableColoredOutput();
00101          m_cLogErrFile.open(m_strLogErrFileName.c_str(), std::ios::trunc | std::ios::out);
00102          if(m_cLogErrFile.fail()) {
00103             THROW_ARGOSEXCEPTION("Error opening file \"" << m_strLogErrFileName << "\"");
00104          }
00105          m_pcInitLogErrStream = LOGERR.GetStream().rdbuf();
00106          LOGERR.GetStream().rdbuf(m_cLogErrFile.rdbuf());
00107       }
00108 
00109       /* Check that either -h, -v, -c or -q was passed (strictly one of them) */
00110       UInt32 nOptionsOn = 0;
00111       if(m_strExperimentConfigFile != "") ++nOptionsOn;
00112       if(m_strQuery != "") ++nOptionsOn;
00113       if(m_bHelpWanted) ++nOptionsOn;
00114       if(m_bVersionWanted) ++nOptionsOn;
00115       if(nOptionsOn == 0) {
00116          THROW_ARGOSEXCEPTION("No --help, --version, --config-file or --query options specified.");
00117       }
00118       if(nOptionsOn > 1) {
00119          THROW_ARGOSEXCEPTION("Options --help, --version, --config-file and --query are mutually exclusive.");
00120       }
00121 
00122       if(m_strExperimentConfigFile != "") {
00123          m_eAction = ACTION_RUN_EXPERIMENT;
00124       }
00125 
00126       if(m_strQuery != "") {
00127          m_eAction = ACTION_QUERY;
00128       }
00129 
00130       if(m_bHelpWanted) {
00131          m_eAction = ACTION_SHOW_HELP;
00132       }
00133 
00134       if(m_bVersionWanted) {
00135          m_eAction = ACTION_SHOW_VERSION;
00136       }
00137 
00138    }
00139 
00140    /****************************************/
00141    /****************************************/
00142 
00143    void CARGoSCommandLineArgParser::PrintUsage(CARGoSLog& c_log) {
00144       c_log << "Autonomous Robots Go Swarming (ARGoS) v" << ARGOS_VERSION << "-" << ARGOS_RELEASE << std::endl;
00145       c_log << "Released under the terms of the MIT license." << std::endl;
00146       c_log << std::endl;
00147       c_log << "Usage: argos3 [OPTIONS]" << std::endl;
00148       c_log << std::endl;
00149       c_log << "   -h       | --help                  display this usage information" << std::endl;
00150       c_log << "   -v       | --version               display ARGoS version and release" << std::endl;
00151       c_log << "   -c FILE  | --config-file FILE      the experiment XML configuration file" << std::endl;
00152       c_log << "   -q QUERY | --query QUERY           query the available plugins." << std::endl;
00153       c_log << "   -n       | --no-color              do not use colored output [OPTIONAL]" << std::endl;
00154       c_log << "   -l       | --log-file FILE         redirect LOG to FILE [OPTIONAL]" << std::endl;
00155       c_log << "   -e       | --logerr-file FILE      redirect LOGERR to FILE [OPTIONAL]" << std::endl << std::endl;
00156       c_log << "The options --config-file and --query are mutually exclusive. Either you use" << std::endl;
00157       c_log << "the first, and thus you run an experiment, or you use the second to query the" << std::endl;
00158       c_log << "plugins." << std::endl << std::endl;
00159       c_log << "EXAMPLES" << std::endl << std::endl;
00160       c_log << "To run an experiment, type:" << std::endl << std::endl;
00161       c_log << "   argos3 -c /path/to/myconfig.argos" << std::endl << std::endl;
00162       c_log << "To query the plugins, type:" << std::endl << std::endl;
00163       c_log << "   argos3 -q QUERY" << std::endl << std::endl;
00164       c_log << "where QUERY can have the following values:" << std::endl << std::endl;
00165       c_log << "   all                  print a list of all the available plugins" << std::endl;
00166       c_log << "   actuators            print a list of all the available actuators" << std::endl;
00167       c_log << "   sensors              print a list of all the available sensors" << std::endl;
00168       c_log << "   physics_engines      print a list of all the available physics engines" << std::endl;
00169       c_log << "   media                print a list of all the available media" << std::endl;
00170       c_log << "   visualizations       print a list of all the available visualizations" << std::endl;
00171       c_log << "   entities             print a list of all the available entities" << std::endl << std::endl;
00172       c_log << "Alternatively, QUERY can be the name of a specific plugin as returned by the" << std::endl;
00173       c_log << "above commands. In this case, you get a complete description of the matching" << std::endl;
00174       c_log << "plugins." << std::endl << std::endl;
00175    }
00176 
00177    /****************************************/
00178    /****************************************/
00179 
00180    void CARGoSCommandLineArgParser::PrintVersion() {
00181       LOG << ARGOS_VERSION << "-" << ARGOS_RELEASE << std::endl;
00182    }
00183 
00184    /****************************************/
00185    /****************************************/
00186 
00187 }
```

---

Generated on 10 Jul 2018 for ARGoS by 
 1.6.1 
