# Supplementary material for: Iterative improvement in the automatic modular design of robot swarms
Source: PeerJ Comput Sci. 2020 Dec 7;6:e322. doi: 10.7717/peerj-cs.322 (PMC7924708; doi:10.7717/peerj-cs.322)
Supplement: Supplemental Information 3 [file peerj-cs-06-322-s003.zip › argos3/doc/api/standalone/a00305_source.html]

ARGoS: core/simulator/argos\_command\_line\_arg\_parser.h Source File


- Main Page
- Related Pages
- Namespaces
- Classes
- Files

- File List
- File Members

# core/simulator/argos\_command\_line\_arg\_parser.h

Go to the documentation of this file.

```
00001 
00007 #ifndef ARGOS_COMMAND_LINE_ARG_PARSER_H
00008 #define ARGOS_COMMAND_LINE_ARG_PARSER_H
00009 
00010 namespace argos {
00011    class CARGoSCommandLineArgParser;
00012 }
00013 
00014 #include <argos3/core/utility/configuration/command_line_arg_parser.h>
00015 #include <fstream>
00016 
00017 namespace argos {
00018 
00025    class CARGoSCommandLineArgParser : public CCommandLineArgParser {
00026 
00027    public:
00028 
00032       enum EAction {
00033          ACTION_UNKNOWN = 0,
00034          ACTION_SHOW_HELP,
00035          ACTION_SHOW_VERSION,
00036          ACTION_RUN_EXPERIMENT,
00037          ACTION_QUERY
00038       };
00039 
00040    public:
00041 
00045       CARGoSCommandLineArgParser();
00046 
00050       virtual ~CARGoSCommandLineArgParser();
00051 
00059       virtual void PrintUsage(CARGoSLog& c_log);
00060 
00064       virtual void PrintVersion();
00065 
00079       virtual void Parse(SInt32 n_argc,
00080                          char** ppch_argv);
00081 
00087       inline EAction GetAction() {
00088          return m_eAction;
00089       }
00090 
00097       inline const std::string& GetExperimentConfigFile() {
00098          return m_strExperimentConfigFile;
00099       }
00100 
00107       inline const std::string& GetQuery() {
00108          return m_strQuery;
00109       }
00110 
00118       inline bool IsLogColored() {
00119          return ! m_bNonColoredLog;
00120       }
00121 
00128       inline bool IsHelpWanted() {
00129          return m_bHelpWanted;
00130       }
00131 
00132    private:
00133 
00134       EAction m_eAction;
00135       std::string m_strExperimentConfigFile;
00136       std::string m_strQuery;
00137       std::string m_strLogFileName;
00138       std::ofstream m_cLogFile;
00139       std::streambuf* m_pcInitLogStream;
00140       std::string m_strLogErrFileName;
00141       std::ofstream m_cLogErrFile;
00142       std::streambuf* m_pcInitLogErrStream;
00143       bool m_bNonColoredLog;
00144       bool m_bHelpWanted;
00145       bool m_bVersionWanted;
00146 
00147    };
00148 
00149 }
00150 
00151 #endif
```

---

Generated on 10 Jul 2018 for ARGoS by 
 1.6.1 
