# Supplementary material for: Iterative improvement in the automatic modular design of robot swarms
Source: PeerJ Comput Sci. 2020 Dec 7;6:e322. doi: 10.7717/peerj-cs.322 (PMC7924708; doi:10.7717/peerj-cs.322)
Supplement: Supplemental Information 3 [file peerj-cs-06-322-s003.zip › argos3/doc/api/standalone/a00306_source.html]

ARGoS: core/simulator/entity/composable\_entity.cpp Source File


- Main Page
- Related Pages
- Namespaces
- Classes
- Files

- File List
- File Members

# core/simulator/entity/composable\_entity.cpp

Go to the documentation of this file.

```
00001 
00007 #include "composable_entity.h"
00008 
00009 #include <argos3/core/utility/string_utilities.h>
00010 
00011 namespace argos {
00012 
00013    /****************************************/
00014    /****************************************/
00015 
00016    CComposableEntity::CComposableEntity(CComposableEntity* pc_parent) :
00017       CEntity(pc_parent) {}
00018 
00019    /****************************************/
00020    /****************************************/
00021 
00022    CComposableEntity::CComposableEntity(CComposableEntity* pc_parent,
00023                                         const std::string& str_id) :
00024       CEntity(pc_parent, str_id) {}
00025 
00026    /****************************************/
00027    /****************************************/
00028 
00029    void CComposableEntity::Reset() {
00030       for(CEntity::TMultiMap::iterator it = m_mapComponents.begin();
00031           it != m_mapComponents.end();
00032           ++it) {
00033          it->second->Reset();
00034       }
00035    }
00036 
00037    /****************************************/
00038    /****************************************/
00039 
00040    void CComposableEntity::Update() {
00041       UpdateComponents();
00042    }
00043 
00044    /****************************************/
00045    /****************************************/
00046 
00047    void CComposableEntity::SetEnabled(bool b_enabled) {
00048       CEntity::SetEnabled(b_enabled);
00049       for(CEntity::TMultiMap::iterator it = m_mapComponents.begin();
00050           it != m_mapComponents.end();
00051           ++it) {
00052          it->second->SetEnabled(b_enabled);
00053       }
00054    }
00055 
00056    /****************************************/
00057    /****************************************/
00058 
00059    void CComposableEntity::UpdateComponents() {
00060       for(CEntity::TMultiMap::iterator it = m_mapComponents.begin();
00061           it != m_mapComponents.end();
00062           ++it) {
00063          if(it->second->IsEnabled()) {
00064             it->second->Update();
00065          }
00066       }
00067    }
00068 
00069    /****************************************/
00070    /****************************************/
00071 
00072    void CComposableEntity::AddComponent(CEntity& c_component) {
00073       m_mapComponents.insert(
00074          std::pair<std::string, CEntity*>(
00075             c_component.GetTypeDescription(),
00076             &c_component));
00077       m_vecComponents.push_back(&c_component);
00078    }
00079 
00080    /****************************************/
00081    /****************************************/
00082 
00083    CEntity& CComposableEntity::RemoveComponent(const std::string& str_component) {
00084       try {
00085          CEntity::TMultiMap::iterator it = FindComponent(str_component);
00086          if(it == m_mapComponents.end()) {
00087             THROW_ARGOSEXCEPTION("Element \"" << str_component << "\" not found in the component map.");
00088          }
00089          CEntity& cRetVal = *(it->second);
00090          m_mapComponents.erase(it);
00091          size_t i;
00092          for(i = 0; i < m_vecComponents.size() && m_vecComponents[i] != &cRetVal; ++i);
00093          if(i < m_vecComponents.size()) {
00094             m_vecComponents.erase(m_vecComponents.begin() + i);
00095          }
00096          else {
00097             THROW_ARGOSEXCEPTION("Element \"" << str_component << "\" not found in the component vector, but present in the map. BUG!");
00098          }
00099          return cRetVal;
00100       }
00101       catch(CARGoSException& ex) {
00102          THROW_ARGOSEXCEPTION_NESTED("While removing component \"" << str_component << "\" from the composable entity \"" << GetContext() << GetId() << "\"", ex);
00103       }
00104    }
00105 
00106    /****************************************/
00107    /****************************************/
00108 
00109    CEntity& CComposableEntity::GetComponent(const std::string& str_path) {
00110       try {
00111          /* Search for the path separator character and take the first path segment */
00112          size_t unFirstSeparatorIdx = str_path.find(".");
00113          std::string strFrontIdentifier;
00114          if(unFirstSeparatorIdx == std::string::npos) strFrontIdentifier = str_path;
00115          else strFrontIdentifier = str_path.substr(0, unFirstSeparatorIdx);
00116          /* Try to find the relevant component in this context */
00117          CEntity::TMultiMap::iterator itComponent = FindComponent(strFrontIdentifier);
00118          if(itComponent != m_mapComponents.end()) {
00119             if(unFirstSeparatorIdx == std::string::npos) {
00120                /* Path separator not found, found component in the current context is the one we want */
00121                return *(itComponent->second);
00122             }
00123             /* Path separator found, try to cast the found component to a composable entity */
00124             else {
00125                CComposableEntity* pcComposableEntity = dynamic_cast<CComposableEntity*>(itComponent->second);
00126                if(pcComposableEntity != NULL) {
00127                   /* Dynamic cast of component to composable entity was successful, re-execute this function in the new context */
00128                   return pcComposableEntity->GetComponent(str_path.substr(unFirstSeparatorIdx + 1, std::string::npos));
00129                }
00130                else {
00131                   /* Dynamic cast failed, user is requesting an entity from an entity which is not composable -> error */
00132                   THROW_ARGOSEXCEPTION("Component \"" << strFrontIdentifier << "\" of \"" << GetContext() + GetId()
00133                                        << "\" is not a composable entity");
00134                }
00135             }
00136          }
00137          else {
00138             THROW_ARGOSEXCEPTION("Component \"" << strFrontIdentifier << "\" does not exist in \""
00139                                  << GetContext() +  GetId() << "\"");
00140          }
00141       }
00142       catch(CARGoSException& ex) {
00143          THROW_ARGOSEXCEPTION_NESTED("While getting a component from a composable entity", ex);
00144       }
00145    }
00146 
00147    /****************************************/
00148    /****************************************/
00149 
00150    bool CComposableEntity::HasComponent(const std::string& str_path) {
00151       /* Search for the path separator character and take the first path segement */
00152       size_t unFirstSeparatorIdx = str_path.find(".");
00153       std::string strFrontIdentifier;
00154       if(unFirstSeparatorIdx == std::string::npos) strFrontIdentifier = str_path;
00155       else strFrontIdentifier = str_path.substr(0, unFirstSeparatorIdx);
00156       /* Try to find the relevant component in this context */
00157       CEntity::TMultiMap::iterator itComponent = FindComponent(strFrontIdentifier);
00158       if(itComponent != m_mapComponents.end()) {
00159          if(unFirstSeparatorIdx == std::string::npos) {
00160             /* Path separator not found, found component in the current context is the one we want */
00161             return true;
00162          }
00163          else {
00164             /* Path separator found, try to cast the found component to a composable entity */
00165             CComposableEntity* pcComposableEntity = dynamic_cast<CComposableEntity*>(itComponent->second);
00166             if(pcComposableEntity != NULL) {
00167                /* Dynamic cast of component to composable entity was sucessful, re-execute this function in the new context */
00168                return pcComposableEntity->HasComponent(str_path.substr(unFirstSeparatorIdx + 1, std::string::npos));
00169             }
00170             else {
00171                /* Could not cast to a composable entity, the queried component cannot exist in the specified context */
00172                return false;
00173             }
00174          }
00175       }
00176       else {
00177          /* Could not find the queried component in this context */
00178          return false;
00179       }
00180    }
00181 
00182    /****************************************/
00183    /****************************************/
00184 
00185    CEntity::TMultiMap::iterator CComposableEntity::FindComponent(const std::string& str_component) {
00186       /* Check for the presence of [ */
00187       std::string::size_type unIdentifierStart = str_component.find('[');
00188       if(unIdentifierStart != std::string::npos) {
00189          /* Found, now check for the presence of ] after [ */
00190          std::string::size_type unIdentifierEnd = str_component.rfind(']');
00191          if(unIdentifierEnd != std::string::npos &&
00192             unIdentifierEnd > unIdentifierStart) {
00193             /* Use the string between [ and ] as an index and whatever comes before as base id */
00194             /* Count how many components there are for the base type */
00195             std::string strBaseType = str_component.substr(0, unIdentifierStart);
00196             if(m_mapComponents.count(strBaseType) == 0) {
00197                /* No components of this base type, return an iterator to the end of the collection */
00198                return m_mapComponents.end();
00199             }
00200             else {
00201                /* Components of base type found - extract the uid and search for it */
00202                std::string strComponentId = str_component.substr(unIdentifierStart + 1, unIdentifierEnd - unIdentifierStart - 1);
00203                /* Create a pair of iterators which mark the beginning and the end of the components that match the base type */
00204                std::pair<CEntity::TMultiMap::iterator,
00205                          CEntity::TMultiMap::iterator> cRange = m_mapComponents.equal_range(strBaseType);
00206                /* Create an iterator to hold the component we are trying to locate */
00207                CEntity::TMultiMap::iterator itComponent;
00208                /* Search through components of base type and try find a match for the specified Id */
00209                for(itComponent = cRange.first;
00210                    (itComponent != cRange.second) && (itComponent->second->GetId() != strComponentId);
00211                    ++itComponent);
00212                /* If the iterator itComponent is not equal to cRange.second, then we have found our component */
00213                if(itComponent != cRange.second) {
00214                   return itComponent;
00215                }
00216                else {
00217                   /* Identifer not found in the collection of components with the specified base type,
00218                    * return an iterator to the end of the collect to show this */
00219                   return m_mapComponents.end();
00220                }
00221             }
00222          }
00223          else {
00224             THROW_ARGOSEXCEPTION("Syntax error in entity id \"" << str_component << "\"");
00225          }
00226       }
00227       else {
00228          /* Identifier syntax not used, return an iterator to the first element or the end of collection if
00229           * no elements are found */
00230          return m_mapComponents.find(str_component);
00231       }
00232    }
00233 
00234    /****************************************/
00235    /****************************************/
00236 
00237    REGISTER_STANDARD_SPACE_OPERATIONS_ON_COMPOSABLE(CComposableEntity);
00238 
00239    /****************************************/
00240    /****************************************/
00241 
00242 }
```

---

Generated on 10 Jul 2018 for ARGoS by 
 1.6.1 
