# Supplementary material for: Iterative improvement in the automatic modular design of robot swarms
Source: PeerJ Comput Sci. 2020 Dec 7;6:e322. doi: 10.7717/peerj-cs.322 (PMC7924708; doi:10.7717/peerj-cs.322)
Supplement: Supplemental Information 3 [file peerj-cs-06-322-s003.zip › argos3/doc/api/standalone/a00307_source.html]

ARGoS: core/simulator/entity/composable\_entity.h Source File


- Main Page
- Related Pages
- Namespaces
- Classes
- Files

- File List
- File Members

# core/simulator/entity/composable\_entity.h

Go to the documentation of this file.

```
00001 
00013 #ifndef COMPOSABLE_ENTITY_H
00014 #define COMPOSABLE_ENTITY_H
00015 
00016 namespace argos {
00017    class CComposableEntity;
00018 }
00019 
00020 #include <argos3/core/simulator/entity/entity.h>
00021 #include <argos3/core/simulator/space/space.h>
00022 
00023 namespace argos {
00024 
00032    class CComposableEntity : public CEntity {
00033 
00034    public:
00035 
00036       ENABLE_VTABLE();
00037 
00038    public:
00039 
00045       CComposableEntity(CComposableEntity* pc_parent);
00046 
00055       CComposableEntity(CComposableEntity* pc_parent,
00056                         const std::string& str_id);
00057 
00061       virtual ~CComposableEntity() {}
00062 
00067       virtual void Reset();
00068 
00075       virtual void Update();
00076 
00077       virtual std::string GetTypeDescription() const {
00078          return "composite";
00079       }
00080 
00087       virtual void SetEnabled(bool b_enabled);
00088 
00093       virtual void UpdateComponents();
00094 
00099       void AddComponent(CEntity& c_component);
00100 
00109       CEntity& RemoveComponent(const std::string& str_component);
00110 
00121       CEntity& GetComponent(const std::string& str_component);
00122 
00135       template <class E>
00136       E& GetComponent(const std::string& str_component) {
00137          E* pcComponent = dynamic_cast<E*>(&GetComponent(str_component));
00138          if(pcComponent != NULL) {
00139             return *pcComponent;
00140          }
00141          else {
00142             THROW_ARGOSEXCEPTION("Type conversion failed for component type \"" << str_component << "\" of entity \"" << GetId());
00143          }
00144       }
00145 
00156       bool HasComponent(const std::string& str_component);
00157 
00166       CEntity::TMultiMap::iterator FindComponent(const std::string& str_component);
00167 
00172       inline CEntity::TMultiMap& GetComponentMap() {
00173          return m_mapComponents;
00174       }
00175 
00181       inline CEntity::TVector& GetComponentVector() {
00182          return m_vecComponents;
00183       }
00184 
00185    private:
00186 
00187       CEntity::TMultiMap m_mapComponents;
00188       CEntity::TVector m_vecComponents;
00189 
00190    };
00191 
00195 #define SPACE_OPERATION_ADD_COMPOSABLE_ENTITY(ENTITY)                                                               \
00196    class CSpaceOperationAdd ## ENTITY : public CSpaceOperationAddEntity {                                           \
00197    public:                                                                                                          \
00198    void ApplyTo(CSpace& c_space, ENTITY& c_entity) {                                                                \
00199       c_space.AddEntity(c_entity);                                                                                  \
00200       for(size_t i = 0; i < c_entity.GetComponentVector().size(); ++i) {                                         \
00201          CallEntityOperation<CSpaceOperationAddEntity, CSpace, void>(c_space, *(c_entity.GetComponentVector()[i])); \
00202       }                                                                                                             \
00203    }                                                                                                                \
00204    };
00205 
00206 #define SPACE_OPERATION_REMOVE_COMPOSABLE_ENTITY(ENTITY)                \
00207    class CSpaceOperationRemove ## ENTITY : public CSpaceOperationRemoveEntity { \
00208    public:                                                              \
00209    void ApplyTo(CSpace& c_space, ENTITY& c_entity) {                    \
00210       CEntity* pcToRemove;                                              \
00211       while(!c_entity.GetComponentVector().empty()) {                   \
00212          pcToRemove = c_entity.GetComponentVector().back();             \
00213          c_entity.RemoveComponent(pcToRemove->GetTypeDescription() + "[" + pcToRemove->GetId() + "]"); \
00214          CallEntityOperation<CSpaceOperationRemoveEntity, CSpace, void>(c_space, *pcToRemove); \
00215       }                                                                 \
00216       c_space.RemoveEntity(c_entity);                                   \
00217    }                                                                    \
00218    };
00219 
00220 #define REGISTER_STANDARD_SPACE_OPERATION_ADD_COMPOSABLE(ENTITY)        \
00221    SPACE_OPERATION_ADD_COMPOSABLE_ENTITY(ENTITY)                        \
00222    REGISTER_SPACE_OPERATION(CSpaceOperationAddEntity,                   \
00223                             CSpaceOperationAdd ## ENTITY,               \
00224                             ENTITY);
00225 
00226 #define REGISTER_STANDARD_SPACE_OPERATION_REMOVE_COMPOSABLE(ENTITY)        \
00227    SPACE_OPERATION_REMOVE_COMPOSABLE_ENTITY(ENTITY)                        \
00228    REGISTER_SPACE_OPERATION(CSpaceOperationRemoveEntity,                   \
00229                             CSpaceOperationRemove ## ENTITY,               \
00230                             ENTITY);
00231 
00232 #define REGISTER_STANDARD_SPACE_OPERATIONS_ON_COMPOSABLE(ENTITY)  \
00233    REGISTER_STANDARD_SPACE_OPERATION_ADD_COMPOSABLE(ENTITY)       \
00234    REGISTER_STANDARD_SPACE_OPERATION_REMOVE_COMPOSABLE(ENTITY)
00235 
00240 }
00241 
00242 #endif
```

---

Generated on 10 Jul 2018 for ARGoS by 
 1.6.1 
