# Supplementary material for: Iterative improvement in the automatic modular design of robot swarms
Source: PeerJ Comput Sci. 2020 Dec 7;6:e322. doi: 10.7717/peerj-cs.322 (PMC7924708; doi:10.7717/peerj-cs.322)
Supplement: Supplemental Information 3 [file peerj-cs-06-322-s003.zip › argos3/doc/api/standalone/a00308_source.html]

ARGoS: core/simulator/entity/controllable\_entity.cpp Source File


- Main Page
- Related Pages
- Namespaces
- Classes
- Files

- File List
- File Members

# core/simulator/entity/controllable\_entity.cpp

Go to the documentation of this file.

```
00001 
00007 #include "controllable_entity.h"
00008 #include <argos3/core/simulator/actuator.h>
00009 #include <argos3/core/simulator/sensor.h>
00010 #include <argos3/core/simulator/simulator.h>
00011 #include <argos3/core/simulator/entity/composable_entity.h>
00012 #include <argos3/core/simulator/space/space.h>
00013 
00014 namespace argos {
00015 
00016    /****************************************/
00017    /****************************************/
00018 
00019    CControllableEntity::CControllableEntity(CComposableEntity* pc_parent) :
00020       CEntity(pc_parent),
00021       m_pcController(NULL) {}
00022 
00023    /****************************************/
00024    /****************************************/
00025 
00026    CControllableEntity::CControllableEntity(CComposableEntity* pc_parent,
00027                                             const std::string& str_id) :
00028       CEntity(pc_parent, str_id),
00029       m_pcController(NULL) {
00030    }
00031 
00032    /****************************************/
00033    /****************************************/
00034 
00035    CControllableEntity::~CControllableEntity() {
00036       if(m_pcController != NULL) {
00037          delete m_pcController;
00038       }
00039    }
00040 
00041    /****************************************/
00042    /****************************************/
00043 
00044    void CControllableEntity::Init(TConfigurationNode& t_tree) {
00045       try {
00046          /* Init parent */
00047          CEntity::Init(t_tree);
00048          /* Get the controller id */
00049          std::string strControllerId;
00050          GetNodeAttribute(t_tree, "config", strControllerId);
00051          /* Check if the tree has parameters to pass to the controller */
00052          if(NodeExists(t_tree, "params")) {
00053             /* Set the controller */
00054             SetController(strControllerId,
00055                           GetNode(t_tree,
00056                                   "params"));
00057          }
00058          else {
00059             /* Set the controller */
00060             SetController(strControllerId);
00061          }
00062       }
00063       catch(CARGoSException& ex) {
00064          THROW_ARGOSEXCEPTION_NESTED("Failed to initialize controllable entity \"" << GetId() << "\".", ex);
00065       }
00066    }
00067 
00068    /****************************************/
00069    /****************************************/
00070 
00071    void CControllableEntity::Reset() {
00072       /* Clear rays */
00073       m_vecCheckedRays.clear();
00074       m_vecIntersectionPoints.clear();
00075       /* Reset sensors */
00076       for(CCI_Sensor::TMap::iterator it = m_pcController->GetAllSensors().begin();
00077           it != m_pcController->GetAllSensors().end(); ++it) {
00078          it->second->Reset();
00079       }
00080       /* Reset actuators */
00081       for(CCI_Actuator::TMap::iterator it = m_pcController->GetAllActuators().begin();
00082           it != m_pcController->GetAllActuators().end(); ++it) {
00083          it->second->Reset();
00084       }
00085       /* Reset user-defined controller */
00086       m_pcController->Reset();
00087    }
00088 
00089    /****************************************/
00090    /****************************************/
00091 
00092    void CControllableEntity::Destroy() {
00093       /* Clear rays */
00094       m_vecCheckedRays.clear();
00095       m_vecIntersectionPoints.clear();
00096       if(m_pcController) {
00097          /* Destroy sensors */
00098          for(CCI_Sensor::TMap::iterator it = m_pcController->GetAllSensors().begin();
00099              it != m_pcController->GetAllSensors().end(); ++it) {
00100             it->second->Destroy();
00101          }
00102          /* Destroy actuators */
00103          for(CCI_Actuator::TMap::iterator it = m_pcController->GetAllActuators().begin();
00104              it != m_pcController->GetAllActuators().end(); ++it) {
00105             it->second->Destroy();
00106          }
00107          /* Destroy user-defined controller */
00108          m_pcController->Destroy();
00109       }
00110    }
00111 
00112    /****************************************/
00113    /****************************************/
00114    
00115    const CCI_Controller& CControllableEntity::GetController() const {
00116       if(m_pcController != NULL) {
00117          return *m_pcController;
00118       }
00119       else {
00120          THROW_ARGOSEXCEPTION("Entity " << GetId() << " does not have any controller associated.");
00121       }
00122    }
00123    
00124    /****************************************/
00125    /****************************************/
00126    
00127    CCI_Controller& CControllableEntity::GetController() {
00128       if(m_pcController != NULL) {
00129          return *m_pcController;
00130       }
00131       else {
00132          THROW_ARGOSEXCEPTION("Entity " << GetId() << " does not have any controller associated.");
00133       }
00134    }
00135    
00136    /****************************************/
00137    /****************************************/
00138    
00139    void CControllableEntity::SetController(const std::string& str_controller_id) {
00140       TConfigurationNode& tConfig = CSimulator::GetInstance().GetConfigForController(str_controller_id);
00141       TConfigurationNode& tParams = GetNode(tConfig, "params");
00142       SetController(str_controller_id, tParams);
00143    }
00144 
00145    /****************************************/
00146    /****************************************/
00147 
00148    void CControllableEntity::SetController(const std::string& str_controller_id,
00149                                            TConfigurationNode& t_controller_config) {
00150       try {
00151          /* Look in the map for the parsed XML configuration of the wanted controller */
00152          TConfigurationNode& tConfig = CSimulator::GetInstance().GetConfigForController(str_controller_id);
00153          /* tConfig is the base of the XML section of the wanted controller */
00154          std::string strImpl;
00155          /* Create the controller */
00156          m_pcController = CFactory<CCI_Controller>::New(tConfig.Value());
00157          m_pcController->SetId(GetParent().GetId());
00158          /* Go through actuators */
00159          TConfigurationNode& tActuators = GetNode(tConfig, "actuators");
00160          TConfigurationNodeIterator itAct;
00161          for(itAct = itAct.begin(&tActuators);
00162              itAct != itAct.end();
00163              ++itAct) {
00164             /* itAct->Value() is the name of the current actuator */
00165             GetNodeAttribute(*itAct, "implementation", strImpl);
00166             CSimulatedActuator* pcAct = CFactory<CSimulatedActuator>::New(itAct->Value() + " (" + strImpl + ")");
00167             CCI_Actuator* pcCIAct = dynamic_cast<CCI_Actuator*>(pcAct);
00168             if(pcCIAct == NULL) {
00169                THROW_ARGOSEXCEPTION("BUG: actuator \"" << itAct->Value() << "\" does not inherit from CCI_Actuator");
00170             }
00171             pcAct->SetRobot(GetParent());
00172             pcCIAct->Init(*itAct);
00173             m_mapActuators[itAct->Value()] = pcAct;
00174             m_pcController->AddActuator(itAct->Value(), pcCIAct);
00175          }
00176          /* Go through sensors */
00177          TConfigurationNode& tSensors = GetNode(tConfig, "sensors");
00178          TConfigurationNodeIterator itSens;
00179          for(itSens = itSens.begin(&tSensors);
00180              itSens != itSens.end();
00181              ++itSens) {
00182             /* itSens->Value() is the name of the current actuator */
00183             GetNodeAttribute(*itSens, "implementation", strImpl);
00184             CSimulatedSensor* pcSens = CFactory<CSimulatedSensor>::New(itSens->Value() + " (" + strImpl + ")");
00185             CCI_Sensor* pcCISens = dynamic_cast<CCI_Sensor*>(pcSens);
00186             if(pcCISens == NULL) {
00187                THROW_ARGOSEXCEPTION("BUG: sensor \"" << itSens->Value() << "\" does not inherit from CCI_Sensor");
00188             }
00189             pcSens->SetRobot(GetParent());
00190             pcCISens->Init(*itSens);
00191             m_mapSensors[itSens->Value()] = pcSens;
00192             m_pcController->AddSensor(itSens->Value(), pcCISens);
00193          }
00194          /* Configure the controller */
00195          m_pcController->Init(t_controller_config);
00196       }
00197       catch(CARGoSException& ex) {
00198          THROW_ARGOSEXCEPTION_NESTED("Can't set controller for controllable entity \"" << GetId() << "\"", ex);
00199       }
00200    }
00201 
00202    /****************************************/
00203    /****************************************/
00204 
00205    void CControllableEntity::Sense() {
00206       m_vecCheckedRays.clear();
00207       m_vecIntersectionPoints.clear();
00208       for(std::map<std::string, CSimulatedSensor*>::iterator it = m_mapSensors.begin();
00209           it != m_mapSensors.end(); ++it) {
00210          it->second->Update();
00211       }
00212    }
00213 
00214    /****************************************/
00215    /****************************************/
00216 
00217    void CControllableEntity::ControlStep() {
00218       if(m_pcController != NULL) {
00219          m_pcController->ControlStep();
00220       }
00221       else {
00222          THROW_ARGOSEXCEPTION("Entity " << GetId() << " does not have any controller associated.");
00223       }
00224    }
00225 
00226    /****************************************/
00227    /****************************************/
00228 
00229    void CControllableEntity::Act() {
00230       for(std::map<std::string, CSimulatedActuator*>::iterator it = m_mapActuators.begin();
00231           it != m_mapActuators.end(); ++it) {
00232          it->second->Update();
00233       }
00234    }
00235 
00236    /****************************************/
00237    /****************************************/
00238 
00242    class CSpaceOperationAddControllableEntity : public CSpaceOperationAddEntity {
00243    public:
00244       void ApplyTo(CSpace& c_space, CControllableEntity& c_entity) {
00245          c_space.AddEntity(c_entity);
00246          c_space.AddControllableEntity(c_entity);
00247       }
00248    };
00249    REGISTER_SPACE_OPERATION(CSpaceOperationAddEntity,
00250                             CSpaceOperationAddControllableEntity,
00251                             CControllableEntity);
00252 
00253    class CSpaceOperationRemoveControllableEntity : public CSpaceOperationRemoveEntity {
00254    public:
00255       void ApplyTo(CSpace& c_space, CControllableEntity& c_entity) {
00256          c_space.RemoveControllableEntity(c_entity);
00257          c_space.RemoveEntity(c_entity);
00258       }
00259    };
00260    REGISTER_SPACE_OPERATION(CSpaceOperationRemoveEntity,
00261                             CSpaceOperationRemoveControllableEntity,
00262                             CControllableEntity);
00267    /****************************************/
00268    /****************************************/
00269 
00270 }
```

---

Generated on 10 Jul 2018 for ARGoS by 
 1.6.1 
