# Supplementary material for: Iterative improvement in the automatic modular design of robot swarms
Source: PeerJ Comput Sci. 2020 Dec 7;6:e322. doi: 10.7717/peerj-cs.322 (PMC7924708; doi:10.7717/peerj-cs.322)
Supplement: Supplemental Information 3 [file peerj-cs-06-322-s003.zip › argos3/doc/api/standalone/a00309_source.html]

ARGoS: core/simulator/entity/controllable\_entity.h Source File


- Main Page
- Related Pages
- Namespaces
- Classes
- Files

- File List
- File Members

# core/simulator/entity/controllable\_entity.h

Go to the documentation of this file.

```
00001 
00007 #ifndef CONTROLLABLE_ENTITY_H
00008 #define CONTROLLABLE_ENTITY_H
00009 
00010 namespace argos {
00011    class CControllableEntity;
00012    class CSimulatedActuator;
00013    class CSimulatedSensor;
00014 }
00015 
00016 #include <argos3/core/simulator/entity/entity.h>
00017 #include <argos3/core/control_interface/ci_controller.h>
00018 #include <argos3/core/utility/math/ray3.h>
00019 
00020 namespace argos {
00021 
00026    class CControllableEntity : public CEntity {
00027 
00028    public:
00029 
00030       ENABLE_VTABLE();
00031 
00033       typedef std::vector<CControllableEntity*> TVector;
00034 
00036       typedef std::map<std::string, CControllableEntity*> TMap;
00037 
00038    public:
00039 
00047       CControllableEntity(CComposableEntity* pc_parent);
00048 
00061       CControllableEntity(CComposableEntity* pc_parent,
00062                           const std::string& str_id);
00063 
00067       virtual ~CControllableEntity();
00068 
00077       virtual void Init(TConfigurationNode& t_tree);
00078 
00082       virtual void Reset();
00083 
00087       virtual void Destroy();
00088 
00095       const CCI_Controller& GetController() const;
00096 
00103       CCI_Controller& GetController();
00104 
00120       void SetController(const std::string& str_controller_id);
00121 
00138       void SetController(const std::string& str_controller_id,
00139                          TConfigurationNode& t_controller_config);
00140 
00148       virtual void Sense();
00149 
00155       virtual void ControlStep();
00156 
00161       virtual void Act();
00162 
00163       virtual std::string GetTypeDescription() const {
00164          return "controller";
00165       }
00166 
00178       inline void AddCheckedRay(bool b_obstructed,
00179                                 const CRay3& c_ray) {
00180          m_vecCheckedRays.push_back(std::make_pair(b_obstructed, c_ray));
00181       }
00182 
00191       inline void AddIntersectionPoint(const CRay3& c_ray,
00192                                        Real f_t_on_ray) {
00193          CVector3 cPoint;
00194          c_ray.GetPoint(cPoint, f_t_on_ray);
00195          m_vecIntersectionPoints.push_back(cPoint);
00196       }
00197 
00204       inline std::vector<std::pair<bool, CRay3> >& GetCheckedRays() {
00205          return m_vecCheckedRays;
00206       }
00207 
00214       inline std::vector<CVector3>& GetIntersectionPoints() {
00215          return m_vecIntersectionPoints;
00216       }
00217 
00218    protected:
00219 
00221       CCI_Controller* m_pcController;
00222 
00224       std::map<std::string, CSimulatedActuator*> m_mapActuators;
00225 
00227       std::map<std::string, CSimulatedSensor*> m_mapSensors;
00228 
00230       std::vector<std::pair<bool, CRay3> > m_vecCheckedRays;
00231 
00233       std::vector<CVector3> m_vecIntersectionPoints;
00234 
00235    };
00236 
00237 }
00238 
00239 #endif
```

---

Generated on 10 Jul 2018 for ARGoS by 
 1.6.1 
