# Supplementary material for: Iterative improvement in the automatic modular design of robot swarms
Source: PeerJ Comput Sci. 2020 Dec 7;6:e322. doi: 10.7717/peerj-cs.322 (PMC7924708; doi:10.7717/peerj-cs.322)
Supplement: Supplemental Information 3 [file peerj-cs-06-322-s003.zip › argos3/doc/api/standalone/a00310.html]

ARGoS: core/simulator/entity/embodied\_entity.cpp File Reference


- Main Page
- Related Pages
- Namespaces
- Classes
- Files

- File List
- File Members

# core/simulator/entity/embodied\_entity.cpp File Reference

`#include "embodied_entity.h"`  
`#include "composable_entity.h"`  
`#include <argos3/core/simulator/space/space.h>`  
`#include <argos3/core/simulator/simulator.h>`  
`#include <argos3/core/utility/string_utilities.h>`  
`#include <argos3/core/utility/math/matrix/rotationmatrix3.h>`  

Include dependency graph for embodied\_entity.cpp:

Go to the source code of this file.

|  |  |
| --- | --- |
| Namespaces | |
| namespace | argos |

|  |  |
| --- | --- |
|  | The namespace containing all the ARGoS related code. |

| Defines | |
| #define | CHECK\_CORNER(MINMAX, COORD, OP) |
| Functions | |
| bool | argos::operator== (const SAnchor \*ps\_anchor, const std::string &str\_id) |
|  | Returns `true` if the anchor id matches the given id. |

---

## Define Documentation

|  |  |  |
| --- | --- | --- |
| #define CHECK\_CORNER | ( | MINMAX, |
|  |  | COORD, |
|  |  | OP |  | ) |  |

**Value:**

```
if(m_sBoundingBox->MINMAX ## Corner.Get ## COORD() OP sBBox.MINMAX ## Corner.Get ## COORD()) { \
      m_sBoundingBox->MINMAX ## Corner.Set ## COORD(sBBox.MINMAX ## Corner.Get ## COORD()); \
   }
```

Definition at line 375 of file embodied\_entity.cpp.

---

Generated on 10 Jul 2018 for ARGoS by 
 1.6.1 
