# Supplementary material for: Iterative improvement in the automatic modular design of robot swarms
Source: PeerJ Comput Sci. 2020 Dec 7;6:e322. doi: 10.7717/peerj-cs.322 (PMC7924708; doi:10.7717/peerj-cs.322)
Supplement: Supplemental Information 3 [file peerj-cs-06-322-s003.zip › argos3/doc/api/standalone/a00310_source.html]

ARGoS: core/simulator/entity/embodied\_entity.cpp Source File


- Main Page
- Related Pages
- Namespaces
- Classes
- Files

- File List
- File Members

# core/simulator/entity/embodied\_entity.cpp

Go to the documentation of this file.

```
00001 
00007 #include "embodied_entity.h"
00008 #include "composable_entity.h"
00009 #include <argos3/core/simulator/space/space.h>
00010 #include <argos3/core/simulator/simulator.h>
00011 #include <argos3/core/utility/string_utilities.h>
00012 #include <argos3/core/utility/math/matrix/rotationmatrix3.h>
00013 
00014 namespace argos {
00015 
00016    /****************************************/
00017    /****************************************/
00018 
00019    CEmbodiedEntity::CEmbodiedEntity(CComposableEntity* pc_parent) :
00020       CEntity(pc_parent),
00021       m_bMovable(true),
00022       m_sBoundingBox(NULL),
00023       m_psOriginAnchor(NULL) {}
00024 
00025    /****************************************/
00026    /****************************************/
00027 
00028    CEmbodiedEntity::CEmbodiedEntity(CComposableEntity* pc_parent,
00029                                     const std::string& str_id,
00030                                     const CVector3& c_position,
00031                                     const CQuaternion& c_orientation,
00032                                     bool b_movable) :
00033       CEntity(pc_parent, str_id),
00034       m_bMovable(b_movable),
00035       m_sBoundingBox(NULL),
00036       m_psOriginAnchor(new SAnchor(*this,
00037                                    "origin",
00038                                    0,
00039                                    CVector3(),
00040                                    CQuaternion(),
00041                                    c_position,
00042                                    c_orientation)),
00043       m_cInitOriginPosition(c_position),
00044       m_cInitOriginOrientation(c_orientation) {
00045       /* Add anchor to map and enable it */
00046       m_mapAnchors[m_psOriginAnchor->Id] = m_psOriginAnchor;
00047       EnableAnchor("origin");
00048    }
00049 
00050    /****************************************/
00051    /****************************************/
00052 
00053    CEmbodiedEntity::~CEmbodiedEntity() {
00054       if(!m_bMovable && m_sBoundingBox != NULL) {
00055          delete m_sBoundingBox;
00056       }
00057       for(std::map<std::string, SAnchor*>::iterator it = m_mapAnchors.begin();
00058           it != m_mapAnchors.end(); ++it) {
00059          /* it->second points to the current anchor */
00060          delete it->second;
00061       }
00062       m_mapAnchors.clear();
00063    }
00064 
00065    /****************************************/
00066    /****************************************/
00067 
00068    void CEmbodiedEntity::Init(TConfigurationNode& t_tree) {
00069       try {
00070          /* Initialize base entity */
00071          CEntity::Init(t_tree);
00072          /* Get the position of the entity */
00073          GetNodeAttributeOrDefault(t_tree, "position", m_cInitOriginPosition, CVector3());
00074          /* Get the orientation of the entity */
00075          GetNodeAttributeOrDefault(t_tree, "orientation", m_cInitOriginOrientation, CQuaternion());
00076          /* Create origin anchor */
00077          m_psOriginAnchor = new SAnchor(*this,
00078                                         "origin",
00079                                         0,
00080                                         CVector3(),
00081                                         CQuaternion(),
00082                                         m_cInitOriginPosition,
00083                                         m_cInitOriginOrientation);
00084          /* Add anchor to map and enable it */
00085          m_mapAnchors[m_psOriginAnchor->Id] = m_psOriginAnchor;
00086          EnableAnchor("origin");
00087          /* Embodied entities are movable by default */
00088          m_bMovable = true;
00089       }
00090       catch(CARGoSException& ex) {
00091          THROW_ARGOSEXCEPTION_NESTED("Failed to initialize embodied entity \"" << GetContext() << GetId() << "\".", ex);
00092       }
00093    }
00094 
00095    /****************************************/
00096    /****************************************/
00097 
00098    void CEmbodiedEntity::Reset() {
00099       /* Reset origin anchor first */
00100       m_psOriginAnchor->Position = m_cInitOriginPosition;
00101       m_psOriginAnchor->Orientation = m_cInitOriginOrientation;
00102       /* Reset other anchors */
00103       SAnchor* psAnchor;
00104       for(std::map<std::string, SAnchor*>::iterator it = m_mapAnchors.begin();
00105           it != m_mapAnchors.end(); ++it) {
00106          /* it->second points to the current anchor */
00107          psAnchor = it->second;
00108          if(psAnchor->Index > 0) {
00109             /* Calculate global position and orientation */
00110             psAnchor->Position = psAnchor->OffsetPosition;
00111             psAnchor->Position.Rotate(m_cInitOriginOrientation);
00112             psAnchor->Position += m_cInitOriginPosition;
00113             psAnchor->Orientation = m_cInitOriginOrientation * psAnchor->OffsetOrientation;
00114          }
00115       }
00116    }
00117 
00118    /****************************************/
00119    /****************************************/
00120 
00121    SAnchor& CEmbodiedEntity::AddAnchor(const std::string& str_id,
00122                                        const CVector3& c_offset_position,
00123                                        const CQuaternion& c_offset_orientation) {
00124       /* Make sure the anchor id is unique */
00125       if(m_mapAnchors.count(str_id) > 0 ) {
00126          THROW_ARGOSEXCEPTION("Embodied entity \"" << GetContext() + GetId() << "\" already has an anchor with id " << str_id);
00127       }
00128       /* Calculate anchor position */
00129       CVector3 cPos = c_offset_position;
00130       cPos.Rotate(m_psOriginAnchor->Orientation);
00131       cPos += m_psOriginAnchor->Position;
00132       /* Calculate anchor orientation */
00133       CQuaternion cOrient = m_psOriginAnchor->Orientation * c_offset_orientation;
00134       /* Create anchor */
00135       SAnchor* psAnchor = new SAnchor(*this,
00136                                       str_id,
00137                                       m_mapAnchors.size(),
00138                                       c_offset_position,
00139                                       c_offset_orientation,
00140                                       cPos,
00141                                       cOrient);
00142       /* Add anchor to map */
00143       m_mapAnchors[str_id] = psAnchor;
00144       return *psAnchor;
00145    }
00146 
00147    /****************************************/
00148    /****************************************/
00149 
00150    void CEmbodiedEntity::EnableAnchor(const std::string& str_id) {
00151       /* Lookup the anchor id */
00152       std::map<std::string, SAnchor*>::iterator it = m_mapAnchors.find(str_id);
00153       /* Found? */
00154       if(it == m_mapAnchors.end()) {
00155          THROW_ARGOSEXCEPTION("Embodied entity \"" << GetContext() + GetId() << "\" has no anchor with id " << str_id);
00156       }
00157       /* Now it->second points to the requested anchor */
00158       /* Increase the in-use count */
00159       ++(it->second->InUseCount);
00160       /* Add to vector of enabled anchors if necessary */
00161       if(it->second->InUseCount == 1) {
00162          m_vecEnabledAnchors.push_back(it->second);
00163       }
00164    }
00165 
00166    /****************************************/
00167    /****************************************/
00168 
00169    void CEmbodiedEntity::DisableAnchor(const std::string& str_id) {
00170       /* Cannot disable the origin anchor */
00171       if(str_id == "origin") return;
00172       /* Lookup the anchor id */
00173       std::vector<SAnchor*>::iterator it = std::find(m_vecEnabledAnchors.begin(),
00174                                                      m_vecEnabledAnchors.end(),
00175                                                      str_id);
00176       /* Found? */
00177       if(it == m_vecEnabledAnchors.end()) return;
00178       /* Now *it points to the requested anchor */
00179       /* Decrease the in-use count */
00180       --((*it)->InUseCount);
00181       /* Remove from vector of enabled anchors if necessary */
00182       if((*it)->InUseCount == 0) {
00183          m_vecEnabledAnchors.erase(it);
00184       }
00185    }
00186 
00187    /****************************************/
00188    /****************************************/
00189 
00190    const SAnchor& CEmbodiedEntity::GetAnchor(const std::string& str_id) const {
00191       /* Lookup the anchor id */
00192       std::map<std::string, SAnchor*>::const_iterator it = m_mapAnchors.find(str_id);
00193       /* Found? */
00194       if(it == m_mapAnchors.end()) {
00195          THROW_ARGOSEXCEPTION("Embodied entity \"" << GetContext() + GetId() << "\" has no anchor with id " << str_id);
00196       }
00197       /* Now it->second points to the requested anchor */
00198       return *(it->second);
00199    }
00200 
00201    /****************************************/
00202    /****************************************/
00203 
00204    SAnchor& CEmbodiedEntity::GetAnchor(const std::string& str_id) {
00205       /* Lookup the anchor id */
00206       std::map<std::string, SAnchor*>::iterator it = m_mapAnchors.find(str_id);
00207       /* Found? */
00208       if(it == m_mapAnchors.end()) {
00209          THROW_ARGOSEXCEPTION("Embodied entity \"" << GetContext() + GetId() << "\" has no anchor with id " << str_id);
00210       }
00211       /* Now it->second points to the requested anchor */
00212       return *(it->second);
00213    }
00214 
00215    /****************************************/
00216    /****************************************/
00217 
00218    bool CEmbodiedEntity::IsAnchorEnabled(const std::string& str_id) {
00219       /* Lookup the anchor id */
00220       std::map<std::string, SAnchor*>::const_iterator it = m_mapAnchors.find(str_id);
00221       /* Found? */
00222       if(it == m_mapAnchors.end()) {
00223          THROW_ARGOSEXCEPTION("Embodied entity \"" << GetContext() + GetId() << "\" has no anchor with id " << str_id);
00224       }
00225       /* Now it->second points to the requested anchor */
00226       return (it->second->InUseCount > 0);
00227    }
00228 
00229    /****************************************/
00230    /****************************************/
00231 
00232    const SBoundingBox& CEmbodiedEntity::GetBoundingBox() const {
00233       if(GetPhysicsModelsNum() == 0) {
00234          /* No engine associated to this entity */
00235          THROW_ARGOSEXCEPTION("CEmbodiedEntity::GetBoundingBox() : entity \"" << GetContext() << GetId() << "\" is not associated to any engine");
00236       }
00237       return *m_sBoundingBox;
00238    }
00239 
00240    /****************************************/
00241    /****************************************/
00242 
00243    UInt32 CEmbodiedEntity::GetPhysicsModelsNum() const {
00244       return m_tPhysicsModelVector.size();
00245    }
00246 
00247    /****************************************/
00248    /****************************************/
00249 
00250    void CEmbodiedEntity::AddPhysicsModel(const std::string& str_engine_id,
00251                                          CPhysicsModel& c_physics_model) {
00252       if(m_bMovable && GetPhysicsModelsNum() > 0) {
00253          THROW_ARGOSEXCEPTION(GetContext() << GetId() << " is a movable embodied entity and can't have more than 1 physics engine entity associated");
00254       }
00255       m_tPhysicsModelMap[str_engine_id] = &c_physics_model;
00256       m_tPhysicsModelVector.push_back(&c_physics_model);
00257       CalculateBoundingBox();
00258    }
00259 
00260    /****************************************/
00261    /****************************************/
00262 
00263    void CEmbodiedEntity::RemovePhysicsModel(const std::string& str_engine_id) {
00264       CPhysicsModel::TMap::iterator itMap = m_tPhysicsModelMap.find(str_engine_id);
00265       if(itMap == m_tPhysicsModelMap.end()) {
00266          THROW_ARGOSEXCEPTION("Entity \"" << GetContext() << GetId() << "\" has no associated entity in physics engine " << str_engine_id);
00267       }
00268       CPhysicsModel::TVector::iterator itVec = std::find(m_tPhysicsModelVector.begin(),
00269                                                          m_tPhysicsModelVector.end(),
00270                                                          itMap->second);
00271       m_tPhysicsModelMap.erase(itMap);
00272       m_tPhysicsModelVector.erase(itVec);
00273       CalculateBoundingBox();
00274    }
00275 
00276    /****************************************/
00277    /****************************************/
00278 
00279    const CPhysicsModel& CEmbodiedEntity::GetPhysicsModel(size_t un_idx) const {
00280       if(un_idx > m_tPhysicsModelVector.size()) {
00281          THROW_ARGOSEXCEPTION("CEmbodiedEntity::GetPhysicsModel: entity \"" << GetContext() << GetId() << "\": the passed index " << un_idx << " is out of bounds, the max allowed is " << m_tPhysicsModelVector.size());
00282       }
00283       return *m_tPhysicsModelVector[un_idx];
00284    }
00285 
00286    /****************************************/
00287    /****************************************/
00288 
00289    CPhysicsModel& CEmbodiedEntity::GetPhysicsModel(size_t un_idx) {
00290       if(un_idx > m_tPhysicsModelVector.size()) {
00291          THROW_ARGOSEXCEPTION("CEmbodiedEntity::GetPhysicsModel: entity \"" << GetContext() << GetId() << "\": the passed index " << un_idx << " is out of bounds, the max allowed is " << m_tPhysicsModelVector.size());
00292       }
00293       return *m_tPhysicsModelVector[un_idx];
00294    }
00295 
00296    /****************************************/
00297    /****************************************/
00298 
00299    const CPhysicsModel& CEmbodiedEntity::GetPhysicsModel(const std::string& str_engine_id) const {
00300       CPhysicsModel::TMap::const_iterator it = m_tPhysicsModelMap.find(str_engine_id);
00301       if(it == m_tPhysicsModelMap.end()) {
00302          THROW_ARGOSEXCEPTION("Entity \"" << GetContext() << GetId() << "\" has no associated entity in physics engine \"" << str_engine_id << "\"");
00303       }
00304       return *(it->second);
00305    }
00306 
00307    /****************************************/
00308    /****************************************/
00309 
00310    CPhysicsModel& CEmbodiedEntity::GetPhysicsModel(const std::string& str_engine_id) {
00311       CPhysicsModel::TMap::iterator it = m_tPhysicsModelMap.find(str_engine_id);
00312       if(it == m_tPhysicsModelMap.end()) {
00313          THROW_ARGOSEXCEPTION("Entity \"" << GetContext() << GetId() << "\" has no associated entity in physics engine \"" << str_engine_id << "\"");
00314       }
00315       return *(it->second);
00316    }
00317 
00318    /****************************************/
00319    /****************************************/
00320 
00321    bool CEmbodiedEntity::MoveTo(const CVector3& c_position,
00322                                 const CQuaternion& c_orientation,
00323                                 bool b_check_only) {
00324       /* Can't move an entity with no model associated */
00325       if(GetPhysicsModelsNum() == 0) return false;
00326       /* Save current position and orientation */
00327       CVector3    cOriginalPosition    = m_psOriginAnchor->Position;
00328       CQuaternion cOriginalOrientation = m_psOriginAnchor->Orientation;
00329       /* Treat specially the case of movable entity */
00330       if(m_bMovable) {
00331          /* Move entity and check for collisions */
00332          m_tPhysicsModelVector[0]->MoveTo(c_position, c_orientation);
00333          bool bNoCollision = ! m_tPhysicsModelVector[0]->IsCollidingWithSomething();
00334          /* Depending on the presence of collisions... */
00335          if(bNoCollision && !b_check_only) {
00336             /* No collision and not a simple check */
00337             /* Tell the caller that we managed to move the entity */
00338             return true;
00339          }
00340          else {
00341             /* Collision or just a check, undo changes */
00342             m_tPhysicsModelVector[0]->MoveTo(cOriginalPosition, cOriginalOrientation);
00343             /* Tell the caller about collisions */
00344             return bNoCollision;
00345          }
00346       }
00347       else {
00348          /* The entity is not movable, go through all the models */
00349          size_t i;
00350          bool bNoCollision = true;
00351          for(i = 0; i < m_tPhysicsModelVector.size() && bNoCollision; ++i) {
00352             m_tPhysicsModelVector[i]->MoveTo(c_position, c_orientation);
00353             bNoCollision = !m_tPhysicsModelVector[i]->IsCollidingWithSomething();
00354          }
00355          if(bNoCollision && !b_check_only) {
00356             /* No collision and not a simple check */
00357             CalculateBoundingBox();
00358             /* Tell the caller that we managed to move the entity */
00359             return true;
00360          }
00361          else {
00362             /* No collision or just a check, undo changes */
00363             for(size_t j = 0; j < i; ++j) {
00364                m_tPhysicsModelVector[j]->MoveTo(cOriginalPosition, cOriginalOrientation);
00365             }
00366             /* Tell the caller about collisions */
00367             return bNoCollision;
00368          }
00369       }
00370    }
00371 
00372    /****************************************/
00373    /****************************************/
00374 
00375 #define CHECK_CORNER(MINMAX, COORD, OP)                                 \
00376    if(m_sBoundingBox->MINMAX ## Corner.Get ## COORD() OP sBBox.MINMAX ## Corner.Get ## COORD()) { \
00377       m_sBoundingBox->MINMAX ## Corner.Set ## COORD(sBBox.MINMAX ## Corner.Get ## COORD()); \
00378    }
00379 
00380    void CEmbodiedEntity::CalculateBoundingBox() {
00381       if(GetPhysicsModelsNum() > 0) {
00382          /*
00383           * There is at least one physics engine entity associated
00384           */
00385          if(m_bMovable) {
00386             /* The bounding box points directly to the associated model bounding box */
00387             m_sBoundingBox = &m_tPhysicsModelVector[0]->GetBoundingBox();
00388          }
00389          else {
00390             /* The bounding box is obtained taking the extrema of all the bboxes of all the engines */
00391             if(m_sBoundingBox == NULL) {
00392                m_sBoundingBox = new SBoundingBox();
00393             }
00394             *m_sBoundingBox = m_tPhysicsModelVector[0]->GetBoundingBox();
00395             for(size_t i = 1; i < GetPhysicsModelsNum(); ++i) {
00396                const SBoundingBox& sBBox = m_tPhysicsModelVector[0]->GetBoundingBox();
00397                CHECK_CORNER(Min, X, >);
00398                CHECK_CORNER(Min, Y, >);
00399                CHECK_CORNER(Min, Z, >);
00400                CHECK_CORNER(Max, X, <);
00401                CHECK_CORNER(Max, Y, <);
00402                CHECK_CORNER(Max, Z, <);
00403             }
00404          }
00405       }
00406       else {
00407          /*
00408           * No physics engine entity associated
00409           */
00410          if(! m_bMovable && m_sBoundingBox != NULL) {
00411             /* A non-movable entity has its own bounding box, delete it */
00412             delete m_sBoundingBox;
00413          }
00414          m_sBoundingBox = NULL;
00415       }
00416    }
00417 
00418    /****************************************/
00419    /****************************************/
00420 
00421    bool CEmbodiedEntity::IsCollidingWithSomething() const {
00422       /* If no model is associated, you can't call this function */
00423       if(m_tPhysicsModelVector.empty()) {
00424          THROW_ARGOSEXCEPTION("CEmbodiedEntity::IsCollidingWithSomething() called on entity \"" <<
00425                               GetContext() << GetId() <<
00426                               "\", but this entity has not been added to any physics engine.");
00427       }
00428       /* Special case: if there is only one model, check that directly */
00429       if(m_tPhysicsModelVector.size() == 1) {
00430          return m_tPhysicsModelVector[0]->IsCollidingWithSomething();
00431       }
00432       /* Multiple associations, go through them */
00433       else {
00434          /* Return true at the first detected collision */
00435          for(size_t i = 0; i < m_tPhysicsModelVector.size(); ++i) {
00436             if(m_tPhysicsModelVector[i]->IsCollidingWithSomething()) {
00437                return true;
00438             }
00439          }
00440          /* If you get here it's because there are collisions */
00441          return false;
00442       }
00443    }
00444 
00445    /****************************************/
00446    /****************************************/
00447 
00448    bool operator==(const SAnchor* ps_anchor,
00449                    const std::string& str_id) {
00450       return (ps_anchor->Id == str_id);
00451    }
00452 
00453    /****************************************/
00454    /****************************************/
00455 
00456    void CEmbodiedEntitySpaceHashUpdater::operator()(CAbstractSpaceHash<CEmbodiedEntity>& c_space_hash,
00457                                                     CEmbodiedEntity& c_element) {
00458       /* Translate the min corner of the bounding box into the map's coordinate */
00459       c_space_hash.SpaceToHashTable(m_nMinX, m_nMinY, m_nMinZ, c_element.GetBoundingBox().MinCorner);
00460       /* Translate the max corner of the bounding box into the map's coordinate */
00461       c_space_hash.SpaceToHashTable(m_nMaxX, m_nMaxY, m_nMaxZ, c_element.GetBoundingBox().MaxCorner);
00462       /* Finally, go through the affected cells and update them */
00463       for(SInt32 nK = m_nMinZ; nK <= m_nMaxZ; ++nK) {
00464          for(SInt32 nJ = m_nMinY; nJ <= m_nMaxY; ++nJ) {
00465             for(SInt32 nI = m_nMinX; nI <= m_nMaxX; ++nI) {
00466                c_space_hash.UpdateCell(nI, nJ, nK, c_element);
00467             }
00468          }
00469       }
00470    }
00471 
00472    /****************************************/
00473    /****************************************/
00474 
00475    CEmbodiedEntityGridUpdater::CEmbodiedEntityGridUpdater(CGrid<CEmbodiedEntity>& c_grid) :
00476       m_cGrid(c_grid) {}
00477 
00478    bool CEmbodiedEntityGridUpdater::operator()(CEmbodiedEntity& c_entity) {
00479       try {
00480          /* Get cell of bb min corner, clamping it if is out of bounds */
00481          m_cGrid.PositionToCell(m_nMinI, m_nMinJ, m_nMinK, c_entity.GetBoundingBox().MinCorner);
00482          m_cGrid.ClampCoordinates(m_nMinI, m_nMinJ, m_nMinK);
00483          /* Get cell of bb max corner, clamping it if is out of bounds */
00484          m_cGrid.PositionToCell(m_nMaxI, m_nMaxJ, m_nMaxK, c_entity.GetBoundingBox().MaxCorner);
00485          m_cGrid.ClampCoordinates(m_nMaxI, m_nMaxJ, m_nMaxK);
00486          /* Go through cells */
00487          for(SInt32 m_nK = m_nMinK; m_nK <= m_nMaxK; ++m_nK) {
00488             for(SInt32 m_nJ = m_nMinJ; m_nJ <= m_nMaxJ; ++m_nJ) {
00489                for(SInt32 m_nI = m_nMinI; m_nI <= m_nMaxI; ++m_nI) {
00490                   m_cGrid.UpdateCell(m_nI, m_nJ, m_nK, c_entity);
00491                }
00492             }
00493          }
00494          /* Continue with the other entities */
00495          return true;
00496       }
00497       catch(CARGoSException& ex) {
00498          THROW_ARGOSEXCEPTION_NESTED("While updating the embodied entity grid for embodied entity \"" << c_entity.GetContext() << c_entity.GetId() << "\"", ex);
00499       }
00500    }
00501 
00502    /****************************************/
00503    /****************************************/
00504 
00508    class CSpaceOperationAddEmbodiedEntity : public CSpaceOperationAddEntity {
00509    public:
00510       void ApplyTo(CSpace& c_space, CEmbodiedEntity& c_entity) {
00511          /* Add entity to space */
00512          c_space.AddEntity(c_entity);
00513          /* Try to add entity to physics engine(s) */
00514          c_space.AddEntityToPhysicsEngine(c_entity);
00515       }
00516    };
00517    REGISTER_SPACE_OPERATION(CSpaceOperationAddEntity, CSpaceOperationAddEmbodiedEntity, CEmbodiedEntity);
00518 
00519    class CSpaceOperationRemoveEmbodiedEntity : public CSpaceOperationRemoveEntity {
00520    public:
00521       void ApplyTo(CSpace& c_space, CEmbodiedEntity& c_entity) {
00522          /* Get a reference to the root entity */
00523          CEntity* pcRoot = &c_entity;
00524          while(pcRoot->HasParent()) {
00525             pcRoot = &pcRoot->GetParent();
00526          }
00527          /* Remove entity from all physics engines */
00528          try {
00529             while(c_entity.GetPhysicsModelsNum() > 0) {
00530                c_entity.GetPhysicsModel(0).GetEngine().RemoveEntity(*pcRoot);
00531             }
00532          }
00533          catch(CARGoSException& ex) {
00534             /*
00535              * It is safe to ignore errors because they happen only when an entity
00536              * is completely removed from the space. In this case, the body is
00537              * first removed from the composable entity, and then the embodied entity
00538              * is asked to clear up the physics models. In turn, this last operation
00539              * searches for the body component, which is not there anymore.
00540              *
00541              * It is anyway useful to search for the body component because, when robots
00542              * are transferred from an engine to another, only the physics model is to be
00543              * removed.
00544              */
00545          }
00546          /* Remove entity from space */
00547          c_space.RemoveEntity(c_entity);
00548       }
00549    };
00550    REGISTER_SPACE_OPERATION(CSpaceOperationRemoveEntity, CSpaceOperationRemoveEmbodiedEntity, CEmbodiedEntity);
00555    /****************************************/
00556    /****************************************/
00557 
00558 }
```

---

Generated on 10 Jul 2018 for ARGoS by 
 1.6.1 
