# Supplementary material for: Iterative improvement in the automatic modular design of robot swarms
Source: PeerJ Comput Sci. 2020 Dec 7;6:e322. doi: 10.7717/peerj-cs.322 (PMC7924708; doi:10.7717/peerj-cs.322)
Supplement: Supplemental Information 3 [file peerj-cs-06-322-s003.zip › argos3/doc/api/standalone/a00311.html]

ARGoS: core/simulator/entity/embodied\_entity.h File Reference


- Main Page
- Related Pages
- Namespaces
- Classes
- Files

- File List
- File Members

# core/simulator/entity/embodied\_entity.h File Reference

`#include <argos3/core/simulator/entity/entity.h>`  
`#include <argos3/core/simulator/space/positional_indices/grid.h>`  
`#include <argos3/core/simulator/space/positional_indices/space_hash.h>`  
`#include <argos3/core/utility/datatypes/set.h>`  
`#include <argos3/core/utility/math/ray3.h>`  
`#include <argos3/core/utility/math/quaternion.h>`  
`#include <argos3/core/simulator/physics_engine/physics_engine.h>`  
`#include <argos3/core/simulator/physics_engine/physics_model.h>`  
`#include <algorithm>`  

Include dependency graph for embodied\_entity.h:

This graph shows which files directly or indirectly include this file:

Go to the source code of this file.

|  |  |
| --- | --- |
| Classes | |
| class | argos::CEmbodiedEntity |
|  | This entity is a link to a body in the physics engine. More... |
| class | argos::CEmbodiedEntityGridUpdater |
| Namespaces | |
| namespace | argos |

|  |  |
| --- | --- |
|  | The namespace containing all the ARGoS related code. |

| Typedefs | |
| typedef std::vector  < CEmbodiedEntity \* > | argos::TEmbodiedEntityVector |
| typedef std::map< std::string,   CEmbodiedEntity \* > | argos::TEmbodiedEntityMap |
| typedef CSet< CEmbodiedEntity \* > | argos::TEmbodiedEntitySet |
| Functions | |
| bool | argos::operator== (const SAnchor \*ps\_anchor, const std::string &str\_id) |
|  | Returns `true` if the anchor id matches the given id. |

---

Generated on 10 Jul 2018 for ARGoS by 
 1.6.1 
