# Supplementary material for: Iterative improvement in the automatic modular design of robot swarms
Source: PeerJ Comput Sci. 2020 Dec 7;6:e322. doi: 10.7717/peerj-cs.322 (PMC7924708; doi:10.7717/peerj-cs.322)
Supplement: Supplemental Information 3 [file peerj-cs-06-322-s003.zip › argos3/doc/api/standalone/a00311_source.html]

ARGoS: core/simulator/entity/embodied\_entity.h Source File


- Main Page
- Related Pages
- Namespaces
- Classes
- Files

- File List
- File Members

# core/simulator/entity/embodied\_entity.h

Go to the documentation of this file.

```
00001 
00007 #ifndef EMBODIED_ENTITY_H
00008 #define EMBODIED_ENTITY_H
00009 
00010 namespace argos {
00011    class CEmbodiedEntity;
00012 }
00013 
00014 #include <argos3/core/simulator/entity/entity.h>
00015 #include <argos3/core/simulator/space/positional_indices/grid.h>
00016 #include <argos3/core/simulator/space/positional_indices/space_hash.h>
00017 #include <argos3/core/utility/datatypes/set.h>
00018 #include <argos3/core/utility/math/ray3.h>
00019 #include <argos3/core/utility/math/quaternion.h>
00020 #include <argos3/core/simulator/physics_engine/physics_engine.h>
00021 #include <argos3/core/simulator/physics_engine/physics_model.h>
00022 #include <algorithm>
00023 
00024 namespace argos {
00025 
00048    class CEmbodiedEntity : public CEntity {
00049 
00050    public:
00051 
00052       ENABLE_VTABLE();
00053 
00054    public:
00055 
00061       CEmbodiedEntity(CComposableEntity* pc_parent);
00062 
00074       CEmbodiedEntity(CComposableEntity* pc_parent,
00075                       const std::string& str_id,
00076                       const CVector3& c_position = CVector3(),
00077                       const CQuaternion& c_orientation = CQuaternion(),
00078                       bool b_movable = true);
00079 
00083       virtual ~CEmbodiedEntity();
00084 
00093       virtual void Init(TConfigurationNode& t_tree);
00094 
00095       virtual void Reset();
00096 
00101       inline bool IsMovable() const {
00102          return m_bMovable;
00103       }
00104 
00111       inline void SetMovable(bool b_movable) {
00112          m_bMovable = b_movable;
00113       }
00114 
00119       inline const SAnchor& GetOriginAnchor() const {
00120          return *m_psOriginAnchor;
00121       }
00122 
00127       inline SAnchor& GetOriginAnchor() {
00128          return *m_psOriginAnchor;
00129       }
00130 
00142       SAnchor& AddAnchor(const std::string& str_id,
00143                          const CVector3& c_rel_position = CVector3(),
00144                          const CQuaternion& c_rel_orientation = CQuaternion());
00145       
00153       void EnableAnchor(const std::string& str_id);
00154 
00162       void DisableAnchor(const std::string& str_id);
00163 
00173       const SAnchor& GetAnchor(const std::string& str_id) const;
00174 
00184       SAnchor& GetAnchor(const std::string& str_id);
00185 
00193       inline std::map<std::string, SAnchor*>& GetAnchors() {
00194          return m_mapAnchors;
00195       }
00196 
00205       inline std::vector<SAnchor*>& GetEnabledAnchors() {
00206          return m_vecEnabledAnchors;
00207       }
00208 
00215       bool IsAnchorEnabled(const std::string& str_id);
00216 
00226       const SBoundingBox& GetBoundingBox() const;
00227 
00232       UInt32 GetPhysicsModelsNum() const;
00233 
00241       virtual void AddPhysicsModel(const std::string& str_engine_id,
00242                                    CPhysicsModel& c_physics_model);
00243 
00250       void RemovePhysicsModel(const std::string& str_engine_id);
00251 
00258       const CPhysicsModel& GetPhysicsModel(size_t un_idx) const;
00259 
00266       CPhysicsModel& GetPhysicsModel(size_t un_idx);
00267 
00274       const CPhysicsModel& GetPhysicsModel(const std::string& str_engine_id) const;
00275 
00282       CPhysicsModel& GetPhysicsModel(const std::string& str_engine_id);
00283 
00295       virtual bool MoveTo(const CVector3& c_position,
00296                           const CQuaternion& c_orientation,
00297                           bool b_check_only = false);
00298 
00303       virtual bool IsCollidingWithSomething() const;
00304 
00305       virtual std::string GetTypeDescription() const {
00306          return "body";
00307       }
00308 
00309    protected:
00310 
00317       void CalculateBoundingBox();
00318       
00319    protected:
00320       
00321       bool m_bMovable;
00322       CPhysicsModel::TMap m_tPhysicsModelMap;
00323       CPhysicsModel::TVector m_tPhysicsModelVector;
00324       SBoundingBox* m_sBoundingBox;
00325       SAnchor* m_psOriginAnchor;
00326       std::map<std::string, SAnchor*> m_mapAnchors;
00327       std::vector<SAnchor*> m_vecEnabledAnchors;
00328       CVector3 m_cInitOriginPosition;
00329       CQuaternion m_cInitOriginOrientation;
00330 
00331    };
00332 
00340    extern bool operator==(const SAnchor* ps_anchor,
00341                           const std::string& str_id);
00342 
00343    /****************************************/
00344    /****************************************/
00345 
00346    typedef std::vector<CEmbodiedEntity*> TEmbodiedEntityVector;
00347    typedef std::map<std::string, CEmbodiedEntity*> TEmbodiedEntityMap;
00348    typedef CSet<CEmbodiedEntity*> TEmbodiedEntitySet;
00349 
00350    /****************************************/
00351    /****************************************/
00352 
00356    class CEmbodiedEntitySpaceHashUpdater : public CSpaceHashUpdater<CEmbodiedEntity> {
00357       
00358    public:
00359       
00360       virtual void operator()(CAbstractSpaceHash<CEmbodiedEntity>& c_space_hash,
00361                               CEmbodiedEntity& c_element);
00362 
00363    private:
00364 
00365       SInt32 m_nMinX, m_nMinY, m_nMinZ;
00366       SInt32 m_nMaxX, m_nMaxY, m_nMaxZ;
00367 
00368    };
00373    /****************************************/
00374    /****************************************/
00375 
00376    class CEmbodiedEntityGridUpdater : public CGrid<CEmbodiedEntity>::COperation {
00377 
00378    public:
00379 
00380       CEmbodiedEntityGridUpdater(CGrid<CEmbodiedEntity>& c_grid);
00381       virtual bool operator()(CEmbodiedEntity& c_entity);
00382 
00383    private:
00384 
00385       CGrid<CEmbodiedEntity>& m_cGrid;
00386       SInt32 m_nMinI, m_nMinJ, m_nMinK;
00387       SInt32 m_nMaxI, m_nMaxJ, m_nMaxK;
00388    };
00389 
00390    /****************************************/
00391    /****************************************/
00392 
00393 }
00394 
00395 #endif
```

---

Generated on 10 Jul 2018 for ARGoS by 
 1.6.1 
