# Supplementary material for: Iterative improvement in the automatic modular design of robot swarms
Source: PeerJ Comput Sci. 2020 Dec 7;6:e322. doi: 10.7717/peerj-cs.322 (PMC7924708; doi:10.7717/peerj-cs.322)
Supplement: Supplemental Information 3 [file peerj-cs-06-322-s003.zip › argos3/doc/api/standalone/a00312_source.html]

ARGoS: core/simulator/entity/entity.cpp Source File


- Main Page
- Related Pages
- Namespaces
- Classes
- Files

- File List
- File Members

# core/simulator/entity/entity.cpp

Go to the documentation of this file.

```
00001 
00008 #include "entity.h"
00009 #include "composable_entity.h"
00010 #include <argos3/core/utility/logging/argos_log.h>
00011 #include <argos3/core/simulator/space/space.h>
00012 
00013 namespace argos {
00014 
00015    /****************************************/
00016    /****************************************/
00017 
00018    CEntity::CEntity(CComposableEntity* pc_parent) :
00019       m_pcParent(pc_parent),
00020       m_bEnabled(true) {
00021    }
00022 
00023    /****************************************/
00024    /****************************************/
00025 
00026    CEntity::CEntity(CComposableEntity* pc_parent,
00027                     const std::string& str_id) :
00028       m_pcParent(pc_parent),
00029       m_strId(str_id),
00030       m_bEnabled(true) {
00031    }
00032 
00033    /****************************************/
00034    /****************************************/
00035 
00036    void CEntity::Init(TConfigurationNode& t_tree) {
00037       try {
00038          /*
00039           * Set the id of the entity from XML or type description
00040           */
00041          /* Was an id specified explicitly? */
00042          if(NodeAttributeExists(t_tree, "id")) {
00043             /* Yes, use that */
00044             GetNodeAttribute(t_tree, "id", m_strId);
00045          }
00046          else {
00047             /* No, derive it from the parent */
00048             if(m_pcParent != NULL) {
00049                UInt32 unIdCount = 0;
00050                while(GetParent().HasComponent(GetTypeDescription() +
00051                                               "[" + GetTypeDescription() +
00052                                               "_" + ToString(unIdCount) +
00053                                               "]")) {
00054                   ++unIdCount;
00055                }
00056                m_strId = GetTypeDescription() + "_" + ToString(unIdCount);
00057             }
00058             else {
00059                THROW_ARGOSEXCEPTION("Root entities must provide the identifier tag");
00060             }
00061          }
00062       }
00063       catch(CARGoSException& ex) {
00064          THROW_ARGOSEXCEPTION_NESTED("Failed to initialize an entity.", ex);
00065       }
00066    }
00067 
00068    /****************************************/
00069    /****************************************/
00070 
00071    std::string CEntity::GetContext() const {
00072       if(m_pcParent != NULL) {
00073          return GetParent().GetContext() + GetParent().GetId() + ".";
00074       }
00075       else {
00076          return "";
00077       }
00078    }
00079 
00080    /****************************************/
00081    /****************************************/
00082    
00083    CComposableEntity& CEntity::GetParent() {
00084       if(m_pcParent != NULL) {
00085          return *m_pcParent;
00086       }
00087       else {
00088          THROW_ARGOSEXCEPTION("Entity \"" << GetId() << "\" has no parent");
00089       }
00090    }
00091 
00092    /****************************************/
00093    /****************************************/
00094 
00095    const CComposableEntity& CEntity::GetParent() const {
00096       if(m_pcParent != NULL) {
00097          return *m_pcParent;
00098       }
00099       else {
00100          THROW_ARGOSEXCEPTION("Entity \"" << GetId() << "\" has no parent");
00101       }
00102    }
00103 
00104    /****************************************/
00105    /****************************************/
00106 
00107    CEntity& CEntity::GetRootEntity() {
00108       if(m_pcParent != NULL) {
00109          return m_pcParent->GetRootEntity();
00110       }
00111       else {
00112          return *this;
00113       }
00114    }
00115 
00116    /****************************************/
00117    /****************************************/
00118    
00119    const CEntity& CEntity::GetRootEntity() const {
00120       if(m_pcParent != NULL) {
00121          return m_pcParent->GetRootEntity();
00122       }
00123       else {
00124          return *this;
00125       }
00126    }
00127 
00128    /****************************************/
00129    /****************************************/
00130 
00131    void CEntity::SetEnabled(bool b_enabled) {
00132       m_bEnabled = b_enabled;
00133    }
00134 
00135    /****************************************/
00136    /****************************************/
00137 
00138    INIT_VTABLE_FOR(CEntity);
00139 
00140    REGISTER_STANDARD_SPACE_OPERATIONS_ON_ENTITY(CEntity);
00141 
00142    /****************************************/
00143    /****************************************/
00144 
00145 }
```

---

Generated on 10 Jul 2018 for ARGoS by 
 1.6.1 
