# Supplementary material for: Iterative improvement in the automatic modular design of robot swarms
Source: PeerJ Comput Sci. 2020 Dec 7;6:e322. doi: 10.7717/peerj-cs.322 (PMC7924708; doi:10.7717/peerj-cs.322)
Supplement: Supplemental Information 3 [file peerj-cs-06-322-s003.zip › argos3/doc/api/standalone/a00313.html]

ARGoS: core/simulator/entity/entity.h File Reference


- Main Page
- Related Pages
- Namespaces
- Classes
- Files

- File List
- File Members

# core/simulator/entity/entity.h File Reference

`#include <argos3/core/utility/datatypes/datatypes.h>`  
`#include <argos3/core/utility/configuration/argos_configuration.h>`  
`#include <argos3/core/utility/configuration/base_configurable_resource.h>`  
`#include <argos3/core/utility/plugins/factory.h>`  
`#include <argos3/core/utility/plugins/vtable.h>`  
`#include <vector>`  
`#include <map>`  
`#include <string>`  
`#include <tr1/unordered_map>`  

Include dependency graph for entity.h:

This graph shows which files directly or indirectly include this file:

Go to the source code of this file.

|  |  |
| --- | --- |
| Classes | |
| class | argos::CEntity |
|  | The basic entity type. More... |
| class | argos::CEntityOperation< LABEL, PLUGIN, RETURN\_TYPE > |
|  | The basic operation to be stored in the vtable. More... |
| struct | argos::SOperationOutcome |
|  | Type to use as return value for operation outcome. More... |
| Namespaces | |
| namespace | argos |

|  |  |
| --- | --- |
|  | The namespace containing all the ARGoS related code. |

| Defines | |
| #define | REGISTER\_ENTITY(CLASSNAME,LABEL,AUTHOR,VERSION,BRIEF\_DESCRIPTION,LONG\_DESCRIPTION,STATUS) |
| #define | REGISTER\_ENTITY\_OPERATION(LABEL, PLUGIN, OPERATION, RETURN\_VALUE, DERIVED) |
|  | Convenience macro to register vtable entity operations. |
| Functions | |
| template<typename LABEL , typename PLUGIN , typename RETURN\_VALUE > | |
| RETURN\_VALUE | argos::CallEntityOperation (PLUGIN &t\_plugin, CEntity &c\_entity) |
|  | Calls the operation corresponding to the given context and operand Skips the function call if the operation is missing in the vtable. |

---

## Define Documentation

|  |  |  |
| --- | --- | --- |
| #define REGISTER\_ENTITY | ( | CLASSNAME, |
|  |  | LABEL, |
|  |  | AUTHOR, |
|  |  | VERSION, |
|  |  | BRIEF\_DESCRIPTION, |
|  |  | LONG\_DESCRIPTION, |
|  |  | STATUS |  | ) |  |

**Value:**

```
REGISTER_SYMBOL(CEntity,                         \
                   CLASSNAME,                       \
                   LABEL,                           \
                   AUTHOR,                          \
                   VERSION,                         \
                   BRIEF_DESCRIPTION,               \
                   LONG_DESCRIPTION,                \
                   STATUS)
```

Definition at line 385 of file entity.h.

|  |  |  |
| --- | --- | --- |
| #define REGISTER\_ENTITY\_OPERATION | ( | LABEL, |
|  |  | PLUGIN, |
|  |  | OPERATION, |
|  |  | RETURN\_VALUE, |
|  |  | DERIVED |  | ) |  |

**Value:**

```
class C ## LABEL ## PLUGIN ## OPERATION ## RETURN_VALUE ## DERIVED {                                    \
      typedef RETURN_VALUE (CEntityOperation<LABEL, PLUGIN, RETURN_VALUE>::*TFunction)(PLUGIN&, CEntity&); \
   public:                                                                                                 \
      C ## LABEL ## PLUGIN ## OPERATION ## RETURN_VALUE ## DERIVED() {                                     \
         GetVTable<LABEL, CEntity, TFunction>().Add<DERIVED>(&OPERATION::Hook<DERIVED, OPERATION>);        \
         GetEntityOperationInstanceHolder<LABEL, PLUGIN, RETURN_VALUE>().Add<DERIVED>(new OPERATION());    \
      }                                                                                                    \
   } c ## LABEL ## PLUGIN ## OPERATION ## RETURN_VALUE ## DERIVED;
```

Convenience macro to register vtable entity operations.

Definition at line 398 of file entity.h.

---

Generated on 10 Jul 2018 for ARGoS by 
 1.6.1 
