# Supplementary material for: Iterative improvement in the automatic modular design of robot swarms
Source: PeerJ Comput Sci. 2020 Dec 7;6:e322. doi: 10.7717/peerj-cs.322 (PMC7924708; doi:10.7717/peerj-cs.322)
Supplement: Supplemental Information 3 [file peerj-cs-06-322-s003.zip › argos3/doc/api/standalone/a00313_source.html]

ARGoS: core/simulator/entity/entity.h Source File


- Main Page
- Related Pages
- Namespaces
- Classes
- Files

- File List
- File Members

# core/simulator/entity/entity.h

Go to the documentation of this file.

```
00001 
00013 #ifndef ENTITY_H
00014 #define ENTITY_H
00015 
00016 namespace argos {
00017    class CEntity;
00018    class CComposableEntity;
00019    class CSpace;
00020 }
00021 
00022 #include <argos3/core/utility/datatypes/datatypes.h>
00023 #include <argos3/core/utility/configuration/argos_configuration.h>
00024 #include <argos3/core/utility/configuration/base_configurable_resource.h>
00025 #include <argos3/core/utility/plugins/factory.h>
00026 #include <argos3/core/utility/plugins/vtable.h>
00027 
00028 #include <vector>
00029 #include <map>
00030 #include <string>
00031 
00032 #if defined(__apple_build_version__ )
00033 // We are on Apple, check compiler version
00034 # if __clang_major__ >= 5
00035   // XCode 5.0.0 or later
00036 #  include <unordered_map>
00037 using std::unordered_map;
00038 # else
00039   // XCode 4.6.3 or earlier
00040 #  include <tr1/unordered_map>
00041 using std::tr1::unordered_map;
00042 # endif
00043 #else
00044 // We are on Linux, use c++03 standard
00045 # include <tr1/unordered_map>
00046 using std::tr1::unordered_map;
00047 #endif
00048 
00049 namespace argos {
00050 
00089    class CEntity : public CBaseConfigurableResource,
00090                    public EnableVTableFor<CEntity> {
00091 
00092    public:
00093 
00094       ENABLE_VTABLE();
00095 
00097       typedef std::vector<CEntity*> TVector;
00098 
00100       typedef unordered_map<std::string, CEntity*> TMap;
00101 
00103       typedef std::multimap<std::string, CEntity*> TMultiMap;
00104 
00105    public:
00106 
00112       CEntity(CComposableEntity* pc_parent);
00113 
00122       CEntity(CComposableEntity* pc_parent,
00123               const std::string& str_id);
00124 
00128       virtual ~CEntity() {}
00129 
00139       virtual void Init(TConfigurationNode& t_tree);
00140 
00145       virtual void Reset() {}
00146 
00151       virtual void Destroy() {}
00152 
00157       inline const std::string& GetId() const {
00158          return m_strId;
00159       }
00160 
00165       std::string GetContext() const;
00166 
00171       inline bool HasParent() const {
00172          return (m_pcParent != NULL);
00173       }
00174 
00179       CEntity& GetRootEntity();
00180 
00185       const CEntity& GetRootEntity() const;
00186 
00192       CComposableEntity& GetParent();
00193 
00199       const CComposableEntity& GetParent() const;
00200 
00205       inline void SetParent(CComposableEntity& c_parent) {
00206          m_pcParent = &c_parent;
00207       }
00208 
00213       virtual std::string GetTypeDescription() const {
00214          return "entity";
00215       }
00216 
00221       virtual void Update() {}
00222 
00229       bool IsEnabled() const {
00230          return m_bEnabled;
00231       }
00232 
00239       inline void Enable() {
00240          SetEnabled(true);
00241       }
00242 
00249       inline void Disable() {
00250          SetEnabled(false);
00251       }
00252 
00259       virtual void SetEnabled(bool b_enabled);
00260 
00261    private:
00262 
00264       CComposableEntity* m_pcParent;
00265 
00267       std::string m_strId;
00268 
00270       bool m_bEnabled;
00271 
00272    };
00273 
00277    template <typename LABEL, typename PLUGIN, typename RETURN_TYPE>
00278    class CEntityOperation {
00279    public:
00280       template <typename DERIVED, typename OPERATION_IMPL>
00281       RETURN_TYPE Hook(PLUGIN& t_plugin, CEntity& c_entity) {
00282          return Dispatch<DERIVED, OPERATION_IMPL>(t_plugin, c_entity);
00283       }
00284    protected:
00285       template <typename DERIVED, typename OPERATION_IMPL>
00286       RETURN_TYPE Dispatch(PLUGIN& t_plugin, CEntity& c_entity) {
00287          /* First dispatch: cast this operation into the specific operation */
00288          OPERATION_IMPL& tOperation = static_cast<OPERATION_IMPL&>(*this);
00289          /* Second dispatch: cast t_base to DERIVED */
00290          DERIVED& tDerived = static_cast<DERIVED&>(c_entity);
00291          /* Perform visit */
00292          return tOperation.ApplyTo(t_plugin, tDerived);
00293       }
00294    };
00295 
00299    struct SOperationOutcome {
00300       bool Value;
00301       SOperationOutcome() : Value(false) {}
00302       SOperationOutcome(bool b_value) : Value(b_value) {}
00303       bool operator()() const { return Value; }
00304    };
00305 
00309    template <typename LABEL, typename PLUGIN, typename RETURN_TYPE>
00310    class CEntityOperationInstanceHolder {
00311    public:
00312       ~CEntityOperationInstanceHolder() {
00313          while(!m_vecOperationInstances.empty()) {
00314             if(m_vecOperationInstances.back() != NULL) {
00315                delete m_vecOperationInstances.back();
00316             }
00317             m_vecOperationInstances.pop_back();
00318          }
00319       }
00320       template <typename DERIVED>
00321       void Add(CEntityOperation<LABEL, PLUGIN, RETURN_TYPE>* pc_operation) {
00322          /* Find the slot */
00323          size_t unIndex = GetTag<DERIVED, CEntity>();
00324          /* Does the holder have a slot for this index? */
00325          if(unIndex >= m_vecOperationInstances.size()) {
00326             /* No, new slots must be created
00327              * Fill the slots with NULL
00328              */
00329             /* Create new slots up to index+1 and fill them with tDefaultFunction */
00330             m_vecOperationInstances.resize(unIndex+1, NULL);
00331          }
00332          m_vecOperationInstances[unIndex] = pc_operation;
00333       }
00334       CEntityOperation<LABEL, PLUGIN, RETURN_TYPE>* operator[](size_t un_index) const {
00335          if(un_index >= m_vecOperationInstances.size()) {
00336             return NULL;
00337          }
00338          return m_vecOperationInstances[un_index];
00339       }
00340    private:
00341       std::vector<CEntityOperation<LABEL, PLUGIN, RETURN_TYPE>*> m_vecOperationInstances;
00342    };
00351    template <typename LABEL, typename PLUGIN, typename RETURN_VALUE>
00352    CEntityOperationInstanceHolder<LABEL, PLUGIN, RETURN_VALUE>& GetEntityOperationInstanceHolder() {
00353       static CEntityOperationInstanceHolder<LABEL, PLUGIN, RETURN_VALUE> cHolder;
00354       return cHolder;
00355    }
00364    template<typename LABEL, typename PLUGIN, typename RETURN_VALUE>
00365    RETURN_VALUE CallEntityOperation(PLUGIN& t_plugin, CEntity& c_entity) {
00366       typedef RETURN_VALUE (CEntityOperation<LABEL, PLUGIN, RETURN_VALUE>::*TFunction)(PLUGIN& t_plugin, CEntity&);
00367       TFunction tFunction = GetVTable<LABEL, CEntity, TFunction>()[c_entity.GetTag()];
00368       if(tFunction != NULL) {
00369          CEntityOperation<LABEL, PLUGIN, RETURN_VALUE>* pcOperation = GetEntityOperationInstanceHolder<LABEL, PLUGIN, RETURN_VALUE>()[c_entity.GetTag()];
00370          if(pcOperation != NULL) {
00371             return (pcOperation->*tFunction)(t_plugin, c_entity);
00372          }
00373       }
00374       return RETURN_VALUE();
00375    }
00376 
00377 }
00378 
00379 #define REGISTER_ENTITY(CLASSNAME,                  \
00380                         LABEL,                      \
00381                         AUTHOR,                     \
00382                         VERSION,                    \
00383                         BRIEF_DESCRIPTION,          \
00384                         LONG_DESCRIPTION,           \
00385                         STATUS)                     \
00386    REGISTER_SYMBOL(CEntity,                         \
00387                    CLASSNAME,                       \
00388                    LABEL,                           \
00389                    AUTHOR,                          \
00390                    VERSION,                         \
00391                    BRIEF_DESCRIPTION,               \
00392                    LONG_DESCRIPTION,                \
00393                    STATUS)
00394 
00398 #define REGISTER_ENTITY_OPERATION(LABEL, PLUGIN, OPERATION, RETURN_VALUE, DERIVED)                         \
00399    class C ## LABEL ## PLUGIN ## OPERATION ## RETURN_VALUE ## DERIVED {                                    \
00400       typedef RETURN_VALUE (CEntityOperation<LABEL, PLUGIN, RETURN_VALUE>::*TFunction)(PLUGIN&, CEntity&); \
00401    public:                                                                                                 \
00402       C ## LABEL ## PLUGIN ## OPERATION ## RETURN_VALUE ## DERIVED() {                                     \
00403          GetVTable<LABEL, CEntity, TFunction>().Add<DERIVED>(&OPERATION::Hook<DERIVED, OPERATION>);        \
00404          GetEntityOperationInstanceHolder<LABEL, PLUGIN, RETURN_VALUE>().Add<DERIVED>(new OPERATION());    \
00405       }                                                                                                    \
00406    } c ## LABEL ## PLUGIN ## OPERATION ## RETURN_VALUE ## DERIVED;
00407 
00408 #endif
```

---

Generated on 10 Jul 2018 for ARGoS by 
 1.6.1 
