# Supplementary material for: Iterative improvement in the automatic modular design of robot swarms
Source: PeerJ Comput Sci. 2020 Dec 7;6:e322. doi: 10.7717/peerj-cs.322 (PMC7924708; doi:10.7717/peerj-cs.322)
Supplement: Supplemental Information 3 [file peerj-cs-06-322-s003.zip › argos3/doc/api/standalone/a00314.html]

ARGoS: core/simulator/entity/floor\_entity.cpp File Reference


- Main Page
- Related Pages
- Namespaces
- Classes
- Files

- File List
- File Members

# core/simulator/entity/floor\_entity.cpp File Reference

`#include "floor_entity.h"`  
`#include <argos3/core/simulator/simulator.h>`  
`#include <argos3/core/simulator/space/space.h>`  
`#include <argos3/core/simulator/loop_functions.h>`  

Include dependency graph for floor\_entity.cpp:

Go to the source code of this file.

|  |  |
| --- | --- |
| Classes | |
| class | argos::CFloorColorFromLoopFunctions |
| class | argos::CSpaceOperationAddCFloorEntity |
| Namespaces | |
| namespace | argos |

|  |  |
| --- | --- |
|  | The namespace containing all the ARGoS related code. |

| Functions | |
|  | argos::REGISTER\_ENTITY (CFloorEntity,"floor","Carlo Pinciroli [ilpincy@gmail.com]","1.0","It contains the properties of the arena floor.","The floor entity contains the properties of the arena floor. In the current\n""implementation, it contains only the color of the floor. The floor color is\n""detected by the robots' ground sensors.\n\n""REQUIRED XML CONFIGURATION\n\n"" <arena ...>\n"" ...\n"" <floor id=\"floor\"\n"" source=\"SOURCE\" />\n"" ...\n"" </arena>\n\n""The 'id' attribute is necessary and must be unique among the entities. If two\n""entities share the same id, initialization aborts.\n""The 'source' attribute specifies where to get the color of the floor from. Its\n""value, here denoted as SOURCE, can assume the following values:\n\n"" image The color is calculated from the passed image file\n"" loop\_functions The color is calculated calling the loop functions\n\n""When 'source' is set to 'image', as showed in the following example, you have\n""to specify the image path in the additional attribute 'path':\n\n"" <arena ...>\n"" ...\n"" <floor id=\"floor\"\n"" source=\"image\"\n"" path=\"/path/to/imagefile.ext\" />\n"" ...\n"" </arena>\n\n""Many image formats are available, such as PNG, JPG, BMP, GIF and many more.\n""Refer to the FreeImage webpage for a complete list of supported image formats\n""(http://freeimage.sourceforge.net/features.html).\n\n""When 'source' is set to 'loop\_functions', as showed in the following example,\n""an image is implicitly created to be used as texture for graphical\n""visualizations. The algorithm that creates the texture needs to convert from\n""meters (in the arena) to pixels (of the texture). You control how many pixels\n""per meter are used with the attribute 'pixels\_per\_meter'. Clearly, the higher\n""value, the higher the quality, but also the slower the algorithm and the bigger\n""the texture. The algorithm is called only once at init time, so the fact that\n""it is slow is not so important. However, the image size is limited by OpenGL.\n""Every implementation has its own limit, and you should check yours if any\n""texture-related problem arises. Now for the example:\n\n"" <arena ...>\n"" ...\n"" <floor id=\"floor\"\n"" source=\"loop\_functions\"\n"" pixels\_per\_meter=\"100\" />\n"" ...\n"" </arena>\n\n""OPTIONAL XML CONFIGURATION\n\n""None for the time being.\n","Usable") |
|  | argos::REGISTER\_SPACE\_OPERATION (CSpaceOperationAddEntity, CSpaceOperationAddCFloorEntity, CFloorEntity) |
|  | argos::REGISTER\_STANDARD\_SPACE\_OPERATION\_REMOVE\_ENTITY (CFloorEntity) |

---

Generated on 10 Jul 2018 for ARGoS by 
 1.6.1 
