# Supplementary material for: Iterative improvement in the automatic modular design of robot swarms
Source: PeerJ Comput Sci. 2020 Dec 7;6:e322. doi: 10.7717/peerj-cs.322 (PMC7924708; doi:10.7717/peerj-cs.322)
Supplement: Supplemental Information 3 [file peerj-cs-06-322-s003.zip › argos3/doc/api/standalone/a00314_source.html]

ARGoS: core/simulator/entity/floor\_entity.cpp Source File


- Main Page
- Related Pages
- Namespaces
- Classes
- Files

- File List
- File Members

# core/simulator/entity/floor\_entity.cpp

Go to the documentation of this file.

```
00001 
00007 #include "floor_entity.h"
00008 #include <argos3/core/simulator/simulator.h>
00009 #include <argos3/core/simulator/space/space.h>
00010 #include <argos3/core/simulator/loop_functions.h>
00011 
00012 #ifdef ARGOS_WITH_FREEIMAGE
00013 #include <FreeImagePlus.h>
00014 #endif
00015 
00016 namespace argos {
00017 
00018    /****************************************/
00019    /****************************************/
00020 
00021 #ifdef ARGOS_WITH_FREEIMAGE
00022    class CFloorColorFromImageFile : public CFloorEntity::CFloorColorSource {
00023 
00024    public:
00025 
00026       CFloorColorFromImageFile(const std::string& str_path) {
00027          const CVector3& cArenaSize = CSimulator::GetInstance().GetSpace().GetArenaSize();
00028          m_cHalfArenaSize.Set(
00029             cArenaSize.GetX() * 0.5f,
00030             cArenaSize.GetY() * 0.5f);
00031          const CVector3& cArenaCenter = CSimulator::GetInstance().GetSpace().GetArenaCenter();
00032          m_cArenaCenter.Set(cArenaCenter.GetX(),
00033                             cArenaCenter.GetY());
00034          LoadImage(str_path);
00035       }
00036 
00037       virtual void Reset() {
00038          LoadImage(m_strImageFileName);
00039       }
00040 
00041       virtual CColor GetColorAtPoint(Real f_x,
00042                                      Real f_y) {
00043          /* Compute coordinates on the image */
00044          UInt32 x = (f_x + m_cHalfArenaSize.GetX()) * m_fArenaToImageCoordinateXFactor;
00045          UInt32 y = (f_y + m_cHalfArenaSize.GetY()) * m_fArenaToImageCoordinateYFactor;
00046          /* Check the bit depth */
00047          if(m_cImage.getBitsPerPixel() <= 8) {
00048             RGBQUAD* ptColorPalette;
00049             BYTE tPixelIndex;
00050             /* 1, 4 or 8 bits per pixel */
00051             if(! m_cImage.getPixelIndex(x, y, &tPixelIndex)) {
00052                THROW_ARGOSEXCEPTION("Unable to access image pixel at (" << x << "," << y <<
00053                                     "). Image size (" << m_cImage.getWidth() << "," <<
00054                                     m_cImage.getHeight() << ")");
00055             }
00056             ptColorPalette = m_cImage.getPalette();
00057             return CColor(ptColorPalette[tPixelIndex].rgbRed,
00058                           ptColorPalette[tPixelIndex].rgbGreen,
00059                           ptColorPalette[tPixelIndex].rgbBlue);
00060          }
00061          else {
00062             /* 16, 24 or 32 bits per pixel */
00063             RGBQUAD tColorPixel;
00064             if(! m_cImage.getPixelColor(x, y, &tColorPixel)) {
00065                THROW_ARGOSEXCEPTION("Unable to access image pixel at (" << x << "," << y <<
00066                                     "). Image size (" << m_cImage.getWidth() << "," <<
00067                                     m_cImage.getHeight() << ")");
00068             }
00069             return CColor(tColorPixel.rgbRed,
00070                           tColorPixel.rgbGreen,
00071                           tColorPixel.rgbBlue);
00072          }
00073       }
00074 
00075       virtual void SaveAsImage(const std::string& str_path) {
00076          m_strImageFileName = str_path;
00077          m_cImage.save(str_path.c_str());
00078       }
00079 
00080       virtual const std::string& GetImageFileName() const {
00081          return m_strImageFileName;
00082       }
00083 
00084    protected:
00085 
00086       void LoadImage(const std::string& str_path) {
00087          m_strImageFileName = str_path;
00088          if(!m_cImage.load(m_strImageFileName.c_str())) {
00089             THROW_ARGOSEXCEPTION("Could not load image \"" <<
00090                                  m_strImageFileName <<
00091                                  "\"");
00092          }
00093          const CVector3& cArenaSize = CSimulator::GetInstance().GetSpace().GetArenaSize();
00094          m_fArenaToImageCoordinateXFactor = m_cImage.getWidth() / cArenaSize.GetX();
00095          m_fArenaToImageCoordinateYFactor = m_cImage.getHeight() / cArenaSize.GetY();
00096       }
00097 
00098    private:
00099 
00100       fipImage m_cImage;
00101       Real m_fArenaToImageCoordinateXFactor;
00102       Real m_fArenaToImageCoordinateYFactor;
00103       CVector2 m_cHalfArenaSize;
00104       CVector2 m_cArenaCenter;
00105       std::string m_strImageFileName;
00106 
00107    };
00108 #endif
00109 
00110    /****************************************/
00111    /****************************************/
00112 
00113    class CFloorColorFromLoopFunctions : public CFloorEntity::CFloorColorSource {
00114 
00115    public:
00116 
00117       CFloorColorFromLoopFunctions(UInt32 un_pixels_per_meter) :
00118          m_cLoopFunctions(CSimulator::GetInstance().GetLoopFunctions()),
00119          m_unPixelsPerMeter(un_pixels_per_meter) {
00120          const CVector3& cArenaSize = CSimulator::GetInstance().GetSpace().GetArenaSize();
00121          m_cHalfArenaSize.Set(
00122             cArenaSize.GetX() * 0.5f,
00123             cArenaSize.GetY() * 0.5f);
00124          const CVector3& cArenaCenter = CSimulator::GetInstance().GetSpace().GetArenaCenter();
00125          m_cArenaCenter.Set(cArenaCenter.GetX(),
00126                             cArenaCenter.GetY());
00127       }
00128 
00129       virtual CColor GetColorAtPoint(Real f_x,
00130                                      Real f_y) {
00131          return m_cLoopFunctions.GetFloorColor(CVector2(f_x, f_y));
00132       }
00133 
00134 #ifdef ARGOS_WITH_FREEIMAGE
00135       virtual void SaveAsImage(const std::string& str_path) {
00136          fipImage cImage(FIT_BITMAP, m_unPixelsPerMeter * m_cHalfArenaSize.GetX()*2, m_unPixelsPerMeter * m_cHalfArenaSize.GetY()*2, 24);
00137          Real fFactor = 1.0f / static_cast<Real>(m_unPixelsPerMeter);
00138          CVector2 cFloorPos;
00139          CColor cARGoSPixel;
00140          RGBQUAD tFIPPixel;
00141          for(UInt32 y = 0; y < cImage.getHeight(); ++y) {
00142             for(UInt32 x = 0; x < cImage.getWidth(); ++x) {
00143                cFloorPos.Set(x * fFactor, y * fFactor);
00144                cFloorPos -= m_cHalfArenaSize;
00145                cFloorPos += m_cArenaCenter;
00146                cARGoSPixel = m_cLoopFunctions.GetFloorColor(cFloorPos);
00147                tFIPPixel.rgbRed = cARGoSPixel.GetRed();
00148                tFIPPixel.rgbGreen = cARGoSPixel.GetGreen();
00149                tFIPPixel.rgbBlue = cARGoSPixel.GetBlue();
00150                cImage.setPixelColor(x, y, &tFIPPixel);
00151             }
00152          }
00153          if(!cImage.save(str_path.c_str())) {
00154             THROW_ARGOSEXCEPTION("Cannot save image \"" << str_path << "\" for floor entity.");
00155          }
00156       }
00157 #endif
00158 
00159    private:
00160 
00161       CLoopFunctions& m_cLoopFunctions;
00162       UInt32 m_unPixelsPerMeter;
00163       CVector2 m_cHalfArenaSize;
00164       CVector2 m_cArenaCenter;
00165    };
00166 
00167    /****************************************/
00168    /****************************************/
00169 
00170    CFloorEntity::CFloorEntity() :
00171       CEntity(NULL),
00172       m_eColorSource(UNSET),
00173       m_pcColorSource(NULL),
00174       m_bHasChanged(true) {}
00175 
00176    /****************************************/
00177    /****************************************/
00178 
00179 #ifdef ARGOS_WITH_FREEIMAGE
00180    CFloorEntity::CFloorEntity(const std::string& str_id,
00181                               const std::string& str_file_name) :
00182       CEntity(NULL, str_id),
00183       m_eColorSource(FROM_IMAGE),
00184       m_pcColorSource(NULL),
00185       m_bHasChanged(true) {
00186       std::string strFileName = str_file_name;
00187       ExpandEnvVariables(strFileName);
00188       m_pcColorSource = new CFloorColorFromImageFile(strFileName);
00189    }
00190 #endif
00191 
00192    /****************************************/
00193    /****************************************/
00194 
00195    CFloorEntity::CFloorEntity(const std::string& str_id,
00196                               UInt32 un_pixels_per_meter) :
00197       CEntity(NULL, str_id),
00198       m_eColorSource(FROM_LOOP_FUNCTIONS),
00199       m_pcColorSource(new CFloorColorFromLoopFunctions(un_pixels_per_meter)),
00200       m_bHasChanged(true) {}
00201 
00202    /****************************************/
00203    /****************************************/
00204 
00205    CFloorEntity::~CFloorEntity() {
00206       if(m_pcColorSource != NULL) {
00207          delete m_pcColorSource;
00208       }
00209    }
00210 
00211    /****************************************/
00212    /****************************************/
00213 
00214    void CFloorEntity::Init(TConfigurationNode& t_tree) {
00215       /* Init parent */
00216       CEntity::Init(t_tree);
00217       /* Parse XML */
00218       std::string strColorSource;
00219       GetNodeAttribute(t_tree, "source", strColorSource);
00220       if(strColorSource == "loop_functions") {
00221          m_eColorSource = FROM_LOOP_FUNCTIONS;
00222          UInt32 unPixelsPerMeter;
00223          GetNodeAttribute(t_tree, "pixels_per_meter", unPixelsPerMeter);
00224          m_pcColorSource = new CFloorColorFromLoopFunctions(unPixelsPerMeter);
00225       }
00226       else if(strColorSource == "image") {
00227 #ifdef ARGOS_WITH_FREEIMAGE
00228          m_eColorSource = FROM_IMAGE;
00229          std::string strPath;
00230          GetNodeAttribute(t_tree, "path", strPath);
00231          ExpandEnvVariables(strPath);
00232          m_pcColorSource = new CFloorColorFromImageFile(strPath);
00233 #else
00234          THROW_ARGOSEXCEPTION("ARGoS was compiled without FreeImage, this image source is unsupported for the floor entity \"" <<
00235                               GetId() <<
00236                               "\"");
00237 #endif
00238       }
00239       else {
00240          THROW_ARGOSEXCEPTION("Unknown image source \"" <<
00241                               strColorSource <<
00242                               "\" for the floor entity \"" <<
00243                               GetId() <<
00244                               "\"");
00245       }
00246    }
00247 
00248    /****************************************/
00249    /****************************************/
00250 
00251    void CFloorEntity::Reset() {
00252       m_pcColorSource->Reset();
00253    }
00254 
00255    /****************************************/
00256    /****************************************/
00257 
00258 #ifdef ARGOS_WITH_FREEIMAGE
00259       void CFloorEntity::SaveAsImage(const std::string& str_path) {
00260          m_pcColorSource->SaveAsImage(str_path);
00261       }
00262 #endif
00263 
00264    /****************************************/
00265    /****************************************/
00266 
00267    REGISTER_ENTITY(CFloorEntity,
00268                    "floor",
00269                    "Carlo Pinciroli [ilpincy@gmail.com]",
00270                    "1.0",
00271                    "It contains the properties of the arena floor.",
00272                    "The floor entity contains the properties of the arena floor. In the current\n"
00273                    "implementation, it contains only the color of the floor. The floor color is\n"
00274                    "detected by the robots' ground sensors.\n\n"
00275                    "REQUIRED XML CONFIGURATION\n\n"
00276                    "  <arena ...>\n"
00277                    "    ...\n"
00278                    "    <floor id=\"floor\"\n"
00279                    "           source=\"SOURCE\" />\n"
00280                    "    ...\n"
00281                    "  </arena>\n\n"
00282                    "The 'id' attribute is necessary and must be unique among the entities. If two\n"
00283                    "entities share the same id, initialization aborts.\n"
00284                    "The 'source' attribute specifies where to get the color of the floor from. Its\n"
00285                    "value, here denoted as SOURCE, can assume the following values:\n\n"
00286                    "  image            The color is calculated from the passed image file\n"
00287                    "  loop_functions   The color is calculated calling the loop functions\n\n"
00288                    "When 'source' is set to 'image', as showed in the following example, you have\n"
00289                    "to specify the image path in the additional attribute 'path':\n\n"
00290                    "  <arena ...>\n"
00291                    "    ...\n"
00292                    "    <floor id=\"floor\"\n"
00293                    "           source=\"image\"\n"
00294                    "           path=\"/path/to/imagefile.ext\" />\n"
00295                    "    ...\n"
00296                    "  </arena>\n\n"
00297                    "Many image formats are available, such as PNG, JPG, BMP, GIF and many more.\n"
00298                    "Refer to the FreeImage webpage for a complete list of supported image formats\n"
00299                    "(http://freeimage.sourceforge.net/features.html).\n\n"
00300                    "When 'source' is set to 'loop_functions', as showed in the following example,\n"
00301                    "an image is implicitly created to be used as texture for graphical\n"
00302                    "visualizations. The algorithm that creates the texture needs to convert from\n"
00303                    "meters (in the arena) to pixels (of the texture). You control how many pixels\n"
00304                    "per meter are used with the attribute 'pixels_per_meter'. Clearly, the higher\n"
00305                    "value, the higher the quality, but also the slower the algorithm and the bigger\n"
00306                    "the texture. The algorithm is called only once at init time, so the fact that\n"
00307                    "it is slow is not so important. However, the image size is limited by OpenGL.\n"
00308                    "Every implementation has its own limit, and you should check yours if any\n"
00309                    "texture-related problem arises. Now for the example:\n\n"
00310                    "  <arena ...>\n"
00311                    "    ...\n"
00312                    "    <floor id=\"floor\"\n"
00313                    "           source=\"loop_functions\"\n"
00314                    "           pixels_per_meter=\"100\" />\n"
00315                    "    ...\n"
00316                    "  </arena>\n\n"
00317                    "OPTIONAL XML CONFIGURATION\n\n"
00318                    "None for the time being.\n",
00319                    "Usable"
00320       );
00321 
00322    /****************************************/
00323    /****************************************/
00324 
00325    class CSpaceOperationAddCFloorEntity : public CSpaceOperationAddEntity {
00326    public:
00327       void ApplyTo(CSpace& c_space, CFloorEntity& c_entity) {
00328          c_space.AddEntity(c_entity);
00329          c_space.SetFloorEntity(c_entity);
00330       }
00331    };
00332 
00333    REGISTER_SPACE_OPERATION(CSpaceOperationAddEntity,
00334                             CSpaceOperationAddCFloorEntity,
00335                             CFloorEntity);
00336    REGISTER_STANDARD_SPACE_OPERATION_REMOVE_ENTITY(CFloorEntity);
00337 
00338    /****************************************/
00339    /****************************************/
00340 
00341 }
```

---

Generated on 10 Jul 2018 for ARGoS by 
 1.6.1 
