# Supplementary material for: Iterative improvement in the automatic modular design of robot swarms
Source: PeerJ Comput Sci. 2020 Dec 7;6:e322. doi: 10.7717/peerj-cs.322 (PMC7924708; doi:10.7717/peerj-cs.322)
Supplement: Supplemental Information 3 [file peerj-cs-06-322-s003.zip › argos3/doc/api/standalone/a00315_source.html]

ARGoS: core/simulator/entity/floor\_entity.h Source File


- Main Page
- Related Pages
- Namespaces
- Classes
- Files

- File List
- File Members

# core/simulator/entity/floor\_entity.h

Go to the documentation of this file.

```
00001 
00007 #ifndef FLOOR_ENTITY_H
00008 #define FLOOR_ENTITY_H
00009 
00010 namespace argos {
00011    class CFloorEntity;
00012 }
00013 
00014 #include <argos3/core/simulator/entity/entity.h>
00015 #include <argos3/core/utility/math/vector2.h>
00016 #include <argos3/core/utility/math/vector3.h>
00017 #include <argos3/core/utility/datatypes/color.h>
00018 
00019 namespace argos {
00020 
00021    class CFloorEntity : public CEntity {
00022 
00023    public:
00024 
00025       class CFloorColorSource {
00026 
00027       public:
00028 
00029          virtual ~CFloorColorSource() {}
00030 
00031          virtual void Reset() {}
00032 
00033          virtual CColor GetColorAtPoint(Real f_x,
00034                                         Real f_y) = 0;
00035 
00036 #ifdef ARGOS_WITH_FREEIMAGE
00037          virtual void SaveAsImage(const std::string& str_path) = 0;
00038 #endif
00039 
00040       };
00041 
00042    public:
00043 
00044       ENABLE_VTABLE();
00045 
00046       enum EColorSource {
00047          UNSET = 0,
00048          FROM_IMAGE,
00049          FROM_LOOP_FUNCTIONS
00050       };
00051 
00052    public:
00053 
00058       CFloorEntity();
00059 
00066 #ifdef ARGOS_WITH_FREEIMAGE
00067       CFloorEntity(const std::string& str_id,
00068                    const std::string& str_file_name);
00069 #endif
00070       
00075       CFloorEntity(const std::string& str_id,
00076                    UInt32 un_pixels_per_meter);
00077 
00081       virtual ~CFloorEntity();
00082 
00086       virtual void Init(TConfigurationNode& t_tree);
00087 
00091       virtual void Reset();
00092 
00099       inline CColor GetColorAtPoint(Real f_x,
00100                                     Real f_y) {
00101          ARGOS_ASSERT(m_pcColorSource != NULL,
00102                       "The floor entity \"" <<
00103                       GetId() <<
00104                       "\" has no associated color source.");
00105          return m_pcColorSource->GetColorAtPoint(f_x, f_y);
00106       }
00107 
00113       inline bool HasChanged() const {
00114          return m_bHasChanged;
00115       }
00116 
00121       inline void SetChanged() {
00122          m_bHasChanged = true;
00123       }
00124 
00129       inline void ClearChanged() {
00130          m_bHasChanged = false;
00131       }
00132 
00139 #ifdef ARGOS_WITH_FREEIMAGE
00140       void SaveAsImage(const std::string& str_path);
00141 #endif
00142 
00143       virtual std::string GetTypeDescription() const {
00144          return "floor";
00145       }
00146 
00147    private:
00148 
00152       EColorSource       m_eColorSource;
00153 
00157       CFloorColorSource* m_pcColorSource;
00158 
00162       bool               m_bHasChanged;
00163    };
00164 }
00165 
00166 #endif
```

---

Generated on 10 Jul 2018 for ARGoS by 
 1.6.1 
