# Supplementary material for: Iterative improvement in the automatic modular design of robot swarms
Source: PeerJ Comput Sci. 2020 Dec 7;6:e322. doi: 10.7717/peerj-cs.322 (PMC7924708; doi:10.7717/peerj-cs.322)
Supplement: Supplemental Information 3 [file peerj-cs-06-322-s003.zip › argos3/doc/api/standalone/a00316_source.html]

ARGoS: core/simulator/entity/positional\_entity.cpp Source File


- Main Page
- Related Pages
- Namespaces
- Classes
- Files

- File List
- File Members

# core/simulator/entity/positional\_entity.cpp

Go to the documentation of this file.

```
00001 
00007 #include "positional_entity.h"
00008 #include "composable_entity.h"
00009 #include <argos3/core/simulator/space/space.h>
00010 #include <argos3/core/simulator/simulator.h>
00011 #include <argos3/core/utility/string_utilities.h>
00012 
00013 namespace argos {
00014 
00015    /****************************************/
00016    /****************************************/
00017 
00018    CPositionalEntity::CPositionalEntity(CComposableEntity* pc_parent) :
00019       CEntity(pc_parent) {}
00020 
00021    /****************************************/
00022    /****************************************/
00023 
00024    CPositionalEntity::CPositionalEntity(CComposableEntity* pc_parent,
00025                                         const std::string& str_id,
00026                                         const CVector3& c_position,
00027                                         const CQuaternion& c_orientation) :
00028       CEntity(pc_parent, str_id),
00029       m_cPosition(c_position),
00030       m_cInitPosition(c_position),
00031       m_cOrientation(c_orientation),
00032       m_cInitOrientation(c_orientation) {}
00033 
00034    /****************************************/
00035    /****************************************/
00036 
00037    void CPositionalEntity::Init(TConfigurationNode& t_tree) {
00038       try {
00039          /* Initialize base entity */
00040          CEntity::Init(t_tree);
00041          /* Get the position of the entity */
00042          GetNodeAttributeOrDefault(t_tree, "position", m_cPosition, CVector3());
00043          /* Get the orientation of the entity */
00044          GetNodeAttributeOrDefault(t_tree, "orientation", m_cOrientation, CQuaternion());
00045          m_cInitPosition = m_cPosition;
00046          m_cInitOrientation = m_cOrientation;
00047       }
00048       catch(CARGoSException& ex) {
00049          THROW_ARGOSEXCEPTION_NESTED("Failed to initialize positional entity \"" << GetId() << "\".", ex);
00050       }
00051    }
00052 
00053    /****************************************/
00054    /****************************************/
00055 
00056    void CPositionalEntity::Reset() {
00057       /* Reset the entity's initial position and orientation */
00058       SetPosition(m_cInitPosition);
00059       SetOrientation(m_cInitOrientation);
00060    }
00061 
00062    /****************************************/
00063    /****************************************/
00064 
00065    void CPositionalEntity::MoveTo(const CVector3& c_position,
00066                                   const CQuaternion& c_orientation) {
00067       SetPosition(c_position);
00068       SetOrientation(c_orientation);
00069    }
00070 
00071    /****************************************/
00072    /****************************************/
00073 
00074    REGISTER_STANDARD_SPACE_OPERATIONS_ON_ENTITY(CPositionalEntity);
00075 
00076    /****************************************/
00077    /****************************************/
00078 
00079 }
```

---

Generated on 10 Jul 2018 for ARGoS by 
 1.6.1 
