# Supplementary material for: Iterative improvement in the automatic modular design of robot swarms
Source: PeerJ Comput Sci. 2020 Dec 7;6:e322. doi: 10.7717/peerj-cs.322 (PMC7924708; doi:10.7717/peerj-cs.322)
Supplement: Supplemental Information 3 [file peerj-cs-06-322-s003.zip › argos3/doc/api/standalone/a00317_source.html]

ARGoS: core/simulator/entity/positional\_entity.h Source File


- Main Page
- Related Pages
- Namespaces
- Classes
- Files

- File List
- File Members

# core/simulator/entity/positional\_entity.h

Go to the documentation of this file.

```
00001 
00007 #ifndef POSITIONAL_ENTITY_H
00008 #define POSITIONAL_ENTITY_H
00009 
00010 namespace argos {
00011    class CPositionalEntity;
00012 }
00013 
00014 #include <argos3/core/simulator/entity/entity.h>
00015 #include <argos3/core/utility/math/vector3.h>
00016 #include <argos3/core/utility/math/quaternion.h>
00017 
00018 namespace argos {
00019 
00020    class CPositionalEntity : public CEntity {
00021 
00022    public:
00023 
00024       ENABLE_VTABLE();
00025 
00026       CPositionalEntity(CComposableEntity* pc_parent);
00027 
00028       CPositionalEntity(CComposableEntity* pc_parent,
00029                         const std::string& str_id,
00030                         const CVector3& c_position = CVector3(),
00031                         const CQuaternion& c_orientation = CQuaternion());
00032 
00033       virtual void Init(TConfigurationNode& t_tree);
00034       virtual void Reset();
00035 
00036       inline const CVector3& GetPosition() const {
00037          return m_cPosition;
00038       }
00039 
00040       inline const CVector3& GetInitPosition() const {
00041          return m_cInitPosition;
00042       }
00043 
00044       inline void SetPosition(const CVector3& c_position) {
00045          m_cPosition = c_position;
00046       }
00047 
00048       inline void SetInitPosition(const CVector3& c_position) {
00049          m_cInitPosition = c_position;
00050       }
00051 
00052       inline const CQuaternion& GetOrientation() const {
00053          return m_cOrientation;
00054       }
00055 
00056       inline const CQuaternion& GetInitOrientation() const {
00057          return m_cInitOrientation;
00058       }
00059 
00060       inline void SetOrientation(const CQuaternion c_orientation) {
00061          m_cOrientation = c_orientation;
00062       }
00063 
00064       inline void SetInitOrientation(const CQuaternion c_orientation) {
00065          m_cInitOrientation = c_orientation;
00066       }
00067 
00068       virtual void MoveTo(const CVector3& c_position,
00069                           const CQuaternion& c_orientation);
00070 
00071       virtual std::string GetTypeDescription() const {
00072          return "position";
00073       }
00074 
00075    private:
00076       
00077       CVector3 m_cPosition;
00078       CVector3 m_cInitPosition;
00079       CQuaternion m_cOrientation;
00080       CQuaternion m_cInitOrientation;
00081    };
00082 }
00083 
00084 #endif
```

---

Generated on 10 Jul 2018 for ARGoS by 
 1.6.1 
