# Supplementary material for: Iterative improvement in the automatic modular design of robot swarms
Source: PeerJ Comput Sci. 2020 Dec 7;6:e322. doi: 10.7717/peerj-cs.322 (PMC7924708; doi:10.7717/peerj-cs.322)
Supplement: Supplemental Information 3 [file peerj-cs-06-322-s003.zip › argos3/doc/api/standalone/a00318_source.html]

ARGoS: core/simulator/loop\_functions.cpp Source File


- Main Page
- Related Pages
- Namespaces
- Classes
- Files

- File List
- File Members

# core/simulator/loop\_functions.cpp

Go to the documentation of this file.

```
00001 
00007 #include "loop_functions.h"
00008 #include <argos3/core/utility/plugins/factory.h>
00009 #include <argos3/core/simulator/entity/positional_entity.h>
00010 #include <argos3/core/simulator/entity/embodied_entity.h>
00011 
00012 namespace argos {
00013 
00014    /****************************************/
00015    /****************************************/
00016    
00017    void CLoopFunctions::MoveEntity(CPositionalEntity& c_entity,
00018                                    const CVector3& c_position,
00019                                    const CQuaternion& c_orientation) {
00020       c_entity.MoveTo(c_position, c_orientation);
00021    }
00022 
00023    /****************************************/
00024    /****************************************/
00025 
00026    bool CLoopFunctions::MoveEntity(CEmbodiedEntity& c_entity,
00027                                    const CVector3& c_position,
00028                                    const CQuaternion& c_orientation,
00029                                    bool b_check_only) {
00030       return c_entity.MoveTo(c_position, c_orientation, b_check_only);
00031    }
00032 
00033    /****************************************/
00034    /****************************************/
00035 
00036    void CLoopFunctions::AddEntity(CEntity& c_entity) {
00037       CallEntityOperation<CSpaceOperationAddEntity, CSpace, void>(m_cSpace, c_entity);
00038    }
00039    
00040    /****************************************/
00041    /****************************************/
00042 
00043    void CLoopFunctions::RemoveEntity(const std::string& str_entity_id) {
00044       CEntity& entity = m_cSpace.GetEntity(str_entity_id);
00045       RemoveEntity(entity);
00046    }
00047    
00048    /****************************************/
00049    /****************************************/
00050    
00051    void CLoopFunctions::RemoveEntity(CEntity& c_entity) {
00052       CallEntityOperation<CSpaceOperationRemoveEntity, CSpace, void>(m_cSpace, c_entity);
00053    }
00054 
00055    /****************************************/
00056    /****************************************/
00057 
00058 }
```

---

Generated on 10 Jul 2018 for ARGoS by 
 1.6.1 
