# Supplementary material for: Iterative improvement in the automatic modular design of robot swarms
Source: PeerJ Comput Sci. 2020 Dec 7;6:e322. doi: 10.7717/peerj-cs.322 (PMC7924708; doi:10.7717/peerj-cs.322)
Supplement: Supplemental Information 3 [file peerj-cs-06-322-s003.zip › argos3/doc/api/standalone/a00319.html]

ARGoS: core/simulator/loop\_functions.h File Reference


- Main Page
- Related Pages
- Namespaces
- Classes
- Files

- File List
- File Members

# core/simulator/loop\_functions.h File Reference

`#include <argos3/core/utility/configuration/base_configurable_resource.h>`  
`#include <argos3/core/simulator/simulator.h>`  
`#include <argos3/core/simulator/space/space.h>`  
`#include <argos3/core/utility/datatypes/color.h>`  
`#include <argos3/core/utility/math/vector2.h>`  
`#include <argos3/core/utility/math/vector3.h>`  
`#include <argos3/core/utility/math/quaternion.h>`  
`#include <argos3/core/simulator/physics_engine/physics_engine.h>`  

Include dependency graph for loop\_functions.h:

This graph shows which files directly or indirectly include this file:

Go to the source code of this file.

|  |  |
| --- | --- |
| Classes | |
| class | argos::CLoopFunctions |
|  | A set of hook functions to customize an experimental run. More... |
| Namespaces | |
| namespace | argos |

|  |  |
| --- | --- |
|  | The namespace containing all the ARGoS related code. |

| Defines | |
| #define | REGISTER\_LOOP\_FUNCTIONS(CLASSNAME, LABEL) |
|  | Registers a loop function class inside ARGoS. |

---

## Define Documentation

|  |  |  |
| --- | --- | --- |
| #define REGISTER\_LOOP\_FUNCTIONS | ( | CLASSNAME, |
|  |  | LABEL |  | ) |  |

**Value:**

```
REGISTER_SYMBOL(CLoopFunctions,                \
                   CLASSNAME,                     \
                   LABEL,                         \
                   "undefined",                   \
                   "undefined",                   \
                   "undefined",                   \
                   "undefined",                   \
                   "undefined")
```

Registers a loop function class inside ARGoS.

You must register your loop function class for ARGoS to be able to recognize it. This statement must be included in a .cpp file. It can't be in a header.

Definition at line 247 of file loop\_functions.h.

---

Generated on 10 Jul 2018 for ARGoS by 
 1.6.1 
