# Supplementary material for: Iterative improvement in the automatic modular design of robot swarms
Source: PeerJ Comput Sci. 2020 Dec 7;6:e322. doi: 10.7717/peerj-cs.322 (PMC7924708; doi:10.7717/peerj-cs.322)
Supplement: Supplemental Information 3 [file peerj-cs-06-322-s003.zip › argos3/doc/api/standalone/a00319_source.html]

ARGoS: core/simulator/loop\_functions.h Source File


- Main Page
- Related Pages
- Namespaces
- Classes
- Files

- File List
- File Members

# core/simulator/loop\_functions.h

Go to the documentation of this file.

```
00001 
00009 #ifndef LOOP_FUNCTIONS_H
00010 #define LOOP_FUNCTIONS_H
00011 
00012 namespace argos {
00013    class CLoopFunctions;
00014    class CFloorEntity;
00015    class CPositionalEntity;
00016    class CEmbodiedEntity;
00017 }
00018 
00019 #include <argos3/core/utility/configuration/base_configurable_resource.h>
00020 #include <argos3/core/simulator/simulator.h>
00021 #include <argos3/core/simulator/space/space.h>
00022 #include <argos3/core/utility/datatypes/color.h>
00023 #include <argos3/core/utility/math/vector2.h>
00024 #include <argos3/core/utility/math/vector3.h>
00025 #include <argos3/core/utility/math/quaternion.h>
00026 #include <argos3/core/simulator/physics_engine/physics_engine.h>
00027 
00028 namespace argos {
00029 
00068    class CLoopFunctions : public CBaseConfigurableResource {
00069 
00070    public:
00071 
00075       CLoopFunctions() :
00076          m_cSimulator(CSimulator::GetInstance()),
00077          m_cSpace(m_cSimulator.GetSpace()) {
00078       }
00079 
00083       virtual ~CLoopFunctions() {}
00084 
00092       virtual void Init(TConfigurationNode& t_tree) {}
00093 
00101       virtual void Reset() {}
00102 
00109       virtual void Destroy() {}
00110 
00117       virtual void PreStep() {}
00118 
00125       virtual void PostStep() {}
00126 
00136       virtual bool IsExperimentFinished() {
00137          return false;
00138       }
00139 
00149       virtual void PostExperiment() {
00150       }
00151 
00163       virtual CColor GetFloorColor(const CVector2& c_pos_on_floor) {
00164          return CColor::WHITE;
00165       }
00166 
00171       inline CSimulator& GetSimulator() {
00172          return m_cSimulator;
00173       }
00174 
00179       inline CSpace& GetSpace() {
00180          return m_cSpace;
00181       }
00182 
00189       virtual void MoveEntity(CPositionalEntity& c_entity,
00190                               const CVector3& c_position,
00191                               const CQuaternion& c_orientation);
00192 
00203       virtual bool MoveEntity(CEmbodiedEntity& c_entity,
00204                               const CVector3& c_position,
00205                               const CQuaternion& c_orientation,
00206                               bool b_check_only = false);
00207 
00216       virtual void AddEntity(CEntity& c_entity);
00217 
00223       virtual void RemoveEntity(const std::string& str_entity_id);
00224 
00229       virtual void RemoveEntity(CEntity& c_entity);
00230 
00231    private:
00232 
00234       CSimulator& m_cSimulator;
00235 
00237       CSpace& m_cSpace;
00238 
00239    };
00240 }
00241 
00247 #define REGISTER_LOOP_FUNCTIONS(CLASSNAME, LABEL) \
00248    REGISTER_SYMBOL(CLoopFunctions,                \
00249                    CLASSNAME,                     \
00250                    LABEL,                         \
00251                    "undefined",                   \
00252                    "undefined",                   \
00253                    "undefined",                   \
00254                    "undefined",                   \
00255                    "undefined")
00256 
00257 #endif
```

---

Generated on 10 Jul 2018 for ARGoS by 
 1.6.1 
