# Supplementary material for: Iterative improvement in the automatic modular design of robot swarms
Source: PeerJ Comput Sci. 2020 Dec 7;6:e322. doi: 10.7717/peerj-cs.322 (PMC7924708; doi:10.7717/peerj-cs.322)
Supplement: Supplemental Information 3 [file peerj-cs-06-322-s003.zip › argos3/doc/api/standalone/a00320.html]

ARGoS: core/simulator/main.cpp File Reference


- Main Page
- Related Pages
- Namespaces
- Classes
- Files

- File List
- File Members

# core/simulator/main.cpp File Reference

`#include <argos3/core/simulator/simulator.h>`  
`#include <argos3/core/utility/plugins/dynamic_loading.h>`  
`#include <argos3/core/simulator/query_plugins.h>`  
`#include <argos3/core/simulator/argos_command_line_arg_parser.h>`  

Include dependency graph for main.cpp:

Go to the source code of this file.

|  |  |
| --- | --- |
| Functions | |
| int | main (int n\_argc, char \*\*ppch\_argv) |
|  | The standard main() function to run the ARGoS simulator. |

---

## Function Documentation

|  |  |  |  |
| --- | --- | --- | --- |
| int main | ( | int | *n\_argc*, |
|  |  | char \*\* | *ppch\_argv* |  |
|  | ) |  |  |  |

The standard main() function to run the ARGoS simulator.

This main() function provides the basic logic to run the ARGoS simulator: parses the command line, loads the experiment, runs the simulation and disposes all the data.

**Parameters:**
:   |  |  |  |
    | --- | --- | --- |
    |  | *n\_argc* | the number of command line arguments given at the shell. |
    |  | *ppch\_argv* | the actual command line arguments. |

**Returns:**
:   0 if everything OK; 1 in case of errors.

Definition at line 25 of file main.cpp.

---

Generated on 10 Jul 2018 for ARGoS by 
 1.6.1 
