# Supplementary material for: Iterative improvement in the automatic modular design of robot swarms
Source: PeerJ Comput Sci. 2020 Dec 7;6:e322. doi: 10.7717/peerj-cs.322 (PMC7924708; doi:10.7717/peerj-cs.322)
Supplement: Supplemental Information 3 [file peerj-cs-06-322-s003.zip › argos3/doc/api/standalone/a00320_source.html]

ARGoS: core/simulator/main.cpp Source File


- Main Page
- Related Pages
- Namespaces
- Classes
- Files

- File List
- File Members

# core/simulator/main.cpp

Go to the documentation of this file.

```
00001 
00007 #include <argos3/core/simulator/simulator.h>
00008 #include <argos3/core/utility/plugins/dynamic_loading.h>
00009 #include <argos3/core/simulator/query_plugins.h>
00010 #include <argos3/core/simulator/argos_command_line_arg_parser.h>
00011 
00012 using namespace argos;
00013 
00025 int main(int n_argc, char** ppch_argv) {
00026    try {
00027       /* Create a new instance of the simulator */
00028       CSimulator& cSimulator = CSimulator::GetInstance();
00029       /* Configure the command line options */
00030       CARGoSCommandLineArgParser cACLAP;
00031       /* Parse command line */
00032       cACLAP.Parse(n_argc, ppch_argv);
00033       switch(cACLAP.GetAction()) {
00034          case CARGoSCommandLineArgParser::ACTION_RUN_EXPERIMENT:
00035             CDynamicLoading::LoadAllLibraries();
00036             cSimulator.SetExperimentFileName(cACLAP.GetExperimentConfigFile());
00037             cSimulator.LoadExperiment();
00038             cSimulator.Execute();
00039             break;
00040          case CARGoSCommandLineArgParser::ACTION_QUERY:
00041             CDynamicLoading::LoadAllLibraries();
00042             QueryPlugins(cACLAP.GetQuery());
00043             break;
00044          case CARGoSCommandLineArgParser::ACTION_SHOW_HELP:
00045             cACLAP.PrintUsage(LOG);
00046             break;
00047          case CARGoSCommandLineArgParser::ACTION_SHOW_VERSION:
00048             cACLAP.PrintVersion();
00049             break;
00050          case CARGoSCommandLineArgParser::ACTION_UNKNOWN:
00051             /* Should never get here */
00052             break;
00053       }
00054       /* Done, destroy stuff */
00055       cSimulator.Destroy();
00056    }
00057    catch(std::exception& ex) {
00058       /* A fatal error occurred: dispose of data, print error and exit */
00059       LOGERR << ex.what() << std::endl;
00060 #ifdef ARGOS_THREADSAFE_LOG
00061       LOG.Flush();
00062       LOGERR.Flush();
00063 #endif
00064       return 1;
00065    }
00066    /* Everything's ok, exit */
00067    return 0;
00068 }
```

---

Generated on 10 Jul 2018 for ARGoS by 
 1.6.1 
