# Supplementary material for: Iterative improvement in the automatic modular design of robot swarms
Source: PeerJ Comput Sci. 2020 Dec 7;6:e322. doi: 10.7717/peerj-cs.322 (PMC7924708; doi:10.7717/peerj-cs.322)
Supplement: Supplemental Information 3 [file peerj-cs-06-322-s003.zip › argos3/doc/api/standalone/a00321_source.html]

ARGoS: core/simulator/medium/medium.cpp Source File


- Main Page
- Related Pages
- Namespaces
- Classes
- Files

- File List
- File Members

# core/simulator/medium/medium.cpp

Go to the documentation of this file.

```
00001 
00007 #include "medium.h"
00008 #include <argos3/core/simulator/space/space.h>
00009 
00010 namespace argos {
00011 
00012    /****************************************/
00013    /****************************************/
00014 
00015    void CMedium::Init(TConfigurationNode& t_tree) {
00016       try {
00017          /* Get id from the XML */
00018          GetNodeAttribute(t_tree, "id", m_strId);
00019       }
00020       catch(CARGoSException& ex) {
00021          THROW_ARGOSEXCEPTION_NESTED("Error initializing a medium entity", ex);
00022       }
00023    }
00024    
00025    /****************************************/
00026    /****************************************/
00027 
00028 }
```

---

Generated on 10 Jul 2018 for ARGoS by 
 1.6.1 
