# Supplementary material for: Iterative improvement in the automatic modular design of robot swarms
Source: PeerJ Comput Sci. 2020 Dec 7;6:e322. doi: 10.7717/peerj-cs.322 (PMC7924708; doi:10.7717/peerj-cs.322)
Supplement: Supplemental Information 3 [file peerj-cs-06-322-s003.zip › argos3/doc/api/standalone/a00322_source.html]

ARGoS: core/simulator/medium/medium.h Source File


- Main Page
- Related Pages
- Namespaces
- Classes
- Files

- File List
- File Members

# core/simulator/medium/medium.h

Go to the documentation of this file.

```
00001 
00007 #ifndef MEDIUM_H
00008 #define MEDIUM_H
00009 
00010 namespace argos {
00011    class CMedium;
00012 }
00013 
00014 #include <argos3/core/utility/configuration/base_configurable_resource.h>
00015 #include <argos3/core/utility/configuration/argos_configuration.h>
00016 #include <argos3/core/utility/datatypes/datatypes.h>
00017 #include <argos3/core/utility/plugins/factory.h>
00018 
00019 namespace argos {
00020 
00021    class CMedium : public CBaseConfigurableResource {
00022 
00023    public:
00024 
00025       typedef std::vector<CMedium*> TVector;
00026       typedef std::map<std::string, CMedium*> TMap;
00027 
00028    public:
00029 
00030       CMedium() {}
00031       virtual ~CMedium() {}
00032 
00047       virtual void Init(TConfigurationNode& t_tree);
00048       virtual void Reset() {}
00049       virtual void Destroy() {}
00050 
00064       virtual void PostSpaceInit() {}
00065 
00069       virtual void Update() = 0;
00070 
00075       inline const std::string& GetId() const {
00076          return m_strId;
00077       }
00078 
00083       void SetId(const std::string& str_id) {
00084          m_strId = str_id;
00085       }
00086 
00087    private:
00088                
00090       std::string m_strId;
00091 
00092    };
00093 
00094 }
00095 
00096 #define REGISTER_MEDIUM(CLASSNAME,                  \
00097                         LABEL,                      \
00098                         AUTHOR,                     \
00099                         VERSION,                    \
00100                         BRIEF_DESCRIPTION,          \
00101                         LONG_DESCRIPTION,           \
00102                         STATUS)                     \
00103    REGISTER_SYMBOL(CMedium,                         \
00104                    CLASSNAME,                       \
00105                    LABEL,                           \
00106                    AUTHOR,                          \
00107                    VERSION,                         \
00108                    BRIEF_DESCRIPTION,               \
00109                    LONG_DESCRIPTION,                \
00110                    STATUS)
00111 
00112 #endif
```

---

Generated on 10 Jul 2018 for ARGoS by 
 1.6.1 
