# Supplementary material for: Iterative improvement in the automatic modular design of robot swarms
Source: PeerJ Comput Sci. 2020 Dec 7;6:e322. doi: 10.7717/peerj-cs.322 (PMC7924708; doi:10.7717/peerj-cs.322)
Supplement: Supplemental Information 3 [file peerj-cs-06-322-s003.zip › argos3/doc/api/standalone/a00323_source.html]

ARGoS: core/simulator/physics\_engine/physics\_engine.cpp Source File


- Main Page
- Related Pages
- Namespaces
- Classes
- Files

- File List
- File Members

# core/simulator/physics\_engine/physics\_engine.cpp

Go to the documentation of this file.

```
00001 
00007 #include <cstdlib>
00008 #include "physics_engine.h"
00009 #include <argos3/core/utility/logging/argos_log.h>
00010 #include <argos3/core/utility/math/vector3.h>
00011 #include <argos3/core/utility/string_utilities.h>
00012 #include <argos3/core/simulator/simulator.h>
00013 #include <argos3/core/simulator/space/space.h>
00014 #include <argos3/core/simulator/entity/entity.h>
00015 
00016 namespace argos {
00017 
00018    /****************************************/
00019    /****************************************/
00020 
00021    bool GetEmbodiedEntitiesIntersectedByRay(TEmbodiedEntityIntersectionData& t_data,
00022                                             const CRay3& c_ray) {
00023       /* This variable is instantiated at the first call of this function, once and forever */
00024       static CSimulator& cSimulator = CSimulator::GetInstance();
00025       /* Clear data */
00026       t_data.clear();
00027       /* Create a reference to the vector of physics engines */
00028       CPhysicsEngine::TVector& vecEngines = cSimulator.GetPhysicsEngines();
00029       /* Ask each engine to perform the ray query */
00030       for(size_t i = 0; i < vecEngines.size(); ++i)
00031          vecEngines[i]->CheckIntersectionWithRay(t_data, c_ray);
00032       /* Remove duplicates */
00033       // TODO
00034       /* Return true if an intersection was found */
00035       return !t_data.empty();
00036    }
00037 
00038    /****************************************/
00039    /****************************************/
00040 
00041    bool GetClosestEmbodiedEntityIntersectedByRay(SEmbodiedEntityIntersectionItem& s_item,
00042                                                  const CRay3& c_ray) {
00043       /* Initialize s_item */
00044       s_item.IntersectedEntity = NULL;
00045       s_item.TOnRay = 1.0f;
00046       /* Perform full ray query */
00047       TEmbodiedEntityIntersectionData tData;
00048       GetEmbodiedEntitiesIntersectedByRay(tData, c_ray);
00049       /* Go through intersections and find the closest */
00050       for(size_t i = 0; i < tData.size(); ++i) {
00051          if(s_item.TOnRay > tData[i].TOnRay)
00052             s_item = tData[i];
00053       }
00054       /* Return true if an intersection was found */
00055       return (s_item.IntersectedEntity != NULL);
00056    }
00057 
00058    /****************************************/
00059    /****************************************/
00060 
00061    bool GetClosestEmbodiedEntityIntersectedByRay(SEmbodiedEntityIntersectionItem& s_item,
00062                                                  const CRay3& c_ray,
00063                                                  CEmbodiedEntity& c_entity) {
00064       /* Initialize s_item */
00065       s_item.IntersectedEntity = NULL;
00066       s_item.TOnRay = 1.0f;
00067       /* Perform full ray query */
00068       TEmbodiedEntityIntersectionData tData;
00069       GetEmbodiedEntitiesIntersectedByRay(tData, c_ray);
00070       /* Go through intersections and find the closest */
00071       for(size_t i = 0; i < tData.size(); ++i) {
00072          if(s_item.TOnRay > tData[i].TOnRay &&
00073             &c_entity != tData[i].IntersectedEntity) {
00074             s_item = tData[i];
00075          }
00076       }
00077       /* Return true if an intersection was found */
00078       return (s_item.IntersectedEntity != NULL);
00079    }
00080 
00081    /****************************************/
00082    /****************************************/
00083 
00084    /* The default value of the simulation clock tick */
00085    Real CPhysicsEngine::m_fSimulationClockTick = 0.1f;
00086    Real CPhysicsEngine::m_fInverseSimulationClockTick = 1.0f / CPhysicsEngine::m_fSimulationClockTick;
00087 
00088    /****************************************/
00089    /****************************************/
00090 
00091    CPhysicsEngine::SVolume::SVolume() :
00092       TopFace(NULL),
00093       BottomFace(NULL) {
00094    }
00095 
00096    /****************************************/
00097    /****************************************/
00098    
00099    void CPhysicsEngine::SVolume::Init(TConfigurationNode& t_node) {
00100       try {
00101          /* Parse top face, if specified */
00102          if(NodeExists(t_node, "top")) {
00103             TConfigurationNode& tNode = GetNode(t_node, "top");
00104             TopFace = new SHorizontalFace;
00105             GetNodeAttribute(tNode, "height", TopFace->Height);
00106          }
00107          /* Parse bottom face, if specified */
00108          if(NodeExists(t_node, "bottom")) {
00109             TConfigurationNode& tNode = GetNode(t_node, "bottom");
00110             BottomFace = new SHorizontalFace;
00111             GetNodeAttribute(tNode, "height", BottomFace->Height);
00112          }
00113          /* Parse side faces, if specified */
00114          if(NodeExists(t_node, "sides")) {
00115             CVector2 cFirstPoint, cLastPoint, cCurPoint;
00116             std::string strConnectWith;
00117             TConfigurationNode& tNode = GetNode(t_node, "sides");
00118             TConfigurationNodeIterator tVertexIt("vertex");
00119             /* Get the first vertex */
00120             tVertexIt = tVertexIt.begin(&tNode);
00121             if(tVertexIt == tVertexIt.end()) {
00122                THROW_ARGOSEXCEPTION("No <vertex> specified within <sides> section");
00123             }
00124             GetNodeAttribute(*tVertexIt, "point", cFirstPoint);
00125             cLastPoint = cFirstPoint;
00126             /* Go through the other vertices */
00127             ++tVertexIt;
00128             while(tVertexIt != tVertexIt.end()) {
00129                /* Read vertex data and fill in segment struct */
00130                GetNodeAttribute(*tVertexIt, "point", cCurPoint);
00131                SVerticalFace* psFace = new SVerticalFace;
00132                psFace->BaseSegment.SetStart(cLastPoint);
00133                psFace->BaseSegment.SetEnd(cCurPoint);
00134                SideFaces.push_back(psFace);
00135                /* Next vertex */
00136                cLastPoint = cCurPoint;
00137                ++tVertexIt;
00138             }
00139             /* Make sure that the boundary is a closed path */
00140             if(SideFaces.size() < 3) {
00141                THROW_ARGOSEXCEPTION("The <sides> path is not closed; at least 3 segments must be specified");
00142             }
00143             if(cLastPoint != cFirstPoint) {
00144                SVerticalFace* psFace = new SVerticalFace;
00145                psFace->BaseSegment.SetStart(cLastPoint);
00146                psFace->BaseSegment.SetEnd(cFirstPoint);
00147                SideFaces.push_back(psFace);
00148             }
00149          }
00150       }
00151       catch(CARGoSException& ex) {
00152          THROW_ARGOSEXCEPTION_NESTED("Error parsing physics engine <boundaries> information", ex);
00153       }
00154    }
00155 
00156    /****************************************/
00157    /****************************************/
00158 
00159    CPhysicsEngine::SVolume::~SVolume() {
00160       if(TopFace)    delete TopFace;
00161       if(BottomFace) delete BottomFace;
00162       while(!SideFaces.empty()) {
00163          delete SideFaces.back();
00164          SideFaces.pop_back();
00165       }
00166    }
00167 
00168    /****************************************/
00169    /****************************************/
00170 
00171    bool CPhysicsEngine::SVolume::IsActive() const {
00172       return TopFace || BottomFace || (!SideFaces.empty());
00173    }
00174 
00175    /****************************************/
00176    /****************************************/
00177 
00178    CPhysicsEngine::CPhysicsEngine() :
00179       m_unIterations(10),
00180       m_fPhysicsClockTick(m_fSimulationClockTick) {}
00181 
00182    /****************************************/
00183    /****************************************/
00184 
00185    void CPhysicsEngine::Init(TConfigurationNode& t_tree) {
00186       try {
00187          /* Get id from the XML */
00188          GetNodeAttribute(t_tree, "id", m_strId);
00189          /* Get iterations per time step */
00190          GetNodeAttributeOrDefault(t_tree, "iterations", m_unIterations, m_unIterations);
00191          m_fPhysicsClockTick = GetSimulationClockTick() / static_cast<Real>(m_unIterations);
00192          LOG << "[INFO] The physics engine \""
00193              << GetId()
00194              << "\" will perform "
00195              << m_unIterations
00196              << " iterations per tick (dt = "
00197              << GetPhysicsClockTick() << " sec)"
00198              << std::endl;
00199          /* Parse the boundary definition, if necessary */
00200          if(NodeExists(t_tree, "boundaries")) {
00201             m_sVolume.Init(GetNode(t_tree, "boundaries"));
00202          }
00203       }
00204       catch(CARGoSException& ex) {
00205          THROW_ARGOSEXCEPTION("Error initializing a physics engine");
00206       }
00207    }
00208 
00209    /****************************************/
00210    /****************************************/
00211 
00212    bool CPhysicsEngine::IsPointContained(const CVector3& c_point) {
00213       if(! IsEntityTransferActive()) {
00214          /*
00215           * The engine has no boundaries, so the wanted point is in for sure
00216           */
00217          return true;
00218       }
00219       else {
00220          /*
00221           * Check the boundaries
00222           */
00223          /* Check top/bottom boundaries */
00224          if((m_sVolume.TopFace    && c_point.GetZ() > m_sVolume.TopFace->Height) ||
00225             (m_sVolume.BottomFace && c_point.GetZ() < m_sVolume.BottomFace->Height)) {
00226             return false;
00227          }
00228          /* Check side boundaries */
00229          for(size_t i = 0; i < GetVolume().SideFaces.size(); ++i) {
00230             const CVector2& cP0 = GetVolume().SideFaces[i]->BaseSegment.GetStart();
00231             const CVector2& cP1 = GetVolume().SideFaces[i]->BaseSegment.GetEnd();
00232             Real fCriterion =
00233                (c_point.GetY() - cP0.GetY()) * (cP1.GetX() - cP0.GetX()) -
00234                (c_point.GetX() - cP0.GetX()) * (cP1.GetY() - cP0.GetY());
00235             if(fCriterion < 0.0f) {
00236                return false;
00237             }
00238          }
00239          return true;
00240       }
00241    }
00242 
00243    /****************************************/
00244    /****************************************/
00245 
00246    void CPhysicsEngine::ScheduleEntityForTransfer(CEmbodiedEntity& c_entity) {
00247       m_vecTransferData.push_back(&c_entity);
00248    }
00249 
00250    /****************************************/
00251    /****************************************/
00252 
00253    void CPhysicsEngine::TransferEntities() {
00254       for(size_t i = 0; i < m_vecTransferData.size(); ++i) {
00255          RemoveEntity(m_vecTransferData[i]->GetRootEntity());
00256          CSimulator::GetInstance().GetSpace().AddEntityToPhysicsEngine(*m_vecTransferData[i]);
00257       }
00258       m_vecTransferData.clear();
00259    }
00260 
00261    /****************************************/
00262    /****************************************/
00263 
00264    Real CPhysicsEngine::GetSimulationClockTick() {
00265       return m_fSimulationClockTick;
00266    }
00267 
00268    /****************************************/
00269    /****************************************/
00270 
00271    Real CPhysicsEngine::GetInverseSimulationClockTick() {
00272       return m_fInverseSimulationClockTick;
00273    }
00274 
00275    /****************************************/
00276    /****************************************/
00277 
00278    void CPhysicsEngine::SetSimulationClockTick(Real f_simulation_clock_tick) {
00279       LOG << "[INFO] Using simulation clock tick = " << f_simulation_clock_tick << std::endl;
00280       m_fSimulationClockTick = f_simulation_clock_tick;
00281       m_fInverseSimulationClockTick = 1.0f / f_simulation_clock_tick;
00282    }
00283 
00284    /****************************************/
00285    /****************************************/
00286 
00287 }
```

---

Generated on 10 Jul 2018 for ARGoS by 
 1.6.1 
