# Supplementary material for: Iterative improvement in the automatic modular design of robot swarms
Source: PeerJ Comput Sci. 2020 Dec 7;6:e322. doi: 10.7717/peerj-cs.322 (PMC7924708; doi:10.7717/peerj-cs.322)
Supplement: Supplemental Information 3 [file peerj-cs-06-322-s003.zip › argos3/doc/api/standalone/a00324.html]

ARGoS: core/simulator/physics\_engine/physics\_engine.h File Reference


- Main Page
- Related Pages
- Namespaces
- Classes
- Files

- File List
- File Members

# core/simulator/physics\_engine/physics\_engine.h File Reference

`#include <map>`  
`#include <argos3/core/utility/logging/argos_log.h>`  
`#include <argos3/core/utility/math/ray2.h>`  
`#include <argos3/core/utility/configuration/base_configurable_resource.h>`  
`#include <argos3/core/utility/configuration/argos_configuration.h>`  
`#include <argos3/core/utility/datatypes/datatypes.h>`  
`#include <argos3/core/utility/plugins/factory.h>`  

Include dependency graph for physics\_engine.h:

This graph shows which files directly or indirectly include this file:

Go to the source code of this file.

|  |  |
| --- | --- |
| Classes | |
| struct | argos::SEmbodiedEntityIntersectionItem |
| class | argos::CPhysicsEngine |
| struct | argos::CPhysicsEngine::SBoundaryFace |
|  | A boundary face for entity transfer among physics engines. More... |
| struct | argos::CPhysicsEngine::SHorizontalFace |
|  | A boundary face for top/bottom parts of the volume. More... |
| struct | argos::CPhysicsEngine::SVerticalFace |
|  | A boundary face for side parts of the volume. More... |
| struct | argos::CPhysicsEngine::SVolume |
|  | The volume boundaries. More... |
| Namespaces | |
| namespace | argos |

|  |  |
| --- | --- |
|  | The namespace containing all the ARGoS related code. |

| Defines | |
| #define | REGISTER\_PHYSICS\_ENGINE(CLASSNAME,LABEL,AUTHOR,VERSION,BRIEF\_DESCRIPTION,LONG\_DESCRIPTION,STATUS) |
| Typedefs | |
| typedef std::vector  < SEmbodiedEntityIntersectionItem > | argos::TEmbodiedEntityIntersectionData |
| Functions | |
| bool | argos::GetEmbodiedEntitiesIntersectedByRay (TEmbodiedEntityIntersectionData &t\_data, const CRay3 &c\_ray) |
|  | Checks whether the given ray intersects any entity. |
| bool | argos::GetClosestEmbodiedEntityIntersectedByRay (SEmbodiedEntityIntersectionItem &s\_item, const CRay3 &c\_ray) |
|  | Returns the closest intersection with an embodied entity to the ray start. |
| bool | argos::GetClosestEmbodiedEntityIntersectedByRay (SEmbodiedEntityIntersectionItem &s\_item, const CRay3 &c\_ray, CEmbodiedEntity &c\_entity) |
|  | Returns the closest intersection with an embodied entity to the ray start. |

---

## Define Documentation

|  |  |  |
| --- | --- | --- |
| #define REGISTER\_PHYSICS\_ENGINE | ( | CLASSNAME, |
|  |  | LABEL, |
|  |  | AUTHOR, |
|  |  | VERSION, |
|  |  | BRIEF\_DESCRIPTION, |
|  |  | LONG\_DESCRIPTION, |
|  |  | STATUS |  | ) |  |

**Value:**

```
REGISTER_SYMBOL(CPhysicsEngine,                  \
                   CLASSNAME,                       \
                   LABEL,                           \
                   AUTHOR,                          \
                   VERSION,                         \
                   BRIEF_DESCRIPTION,               \
                   LONG_DESCRIPTION,                \
                   STATUS)
```

Definition at line 328 of file physics\_engine.h.

---

Generated on 10 Jul 2018 for ARGoS by 
 1.6.1 
