# Supplementary material for: Iterative improvement in the automatic modular design of robot swarms
Source: PeerJ Comput Sci. 2020 Dec 7;6:e322. doi: 10.7717/peerj-cs.322 (PMC7924708; doi:10.7717/peerj-cs.322)
Supplement: Supplemental Information 3 [file peerj-cs-06-322-s003.zip › argos3/doc/api/standalone/a00324_source.html]

ARGoS: core/simulator/physics\_engine/physics\_engine.h Source File


- Main Page
- Related Pages
- Namespaces
- Classes
- Files

- File List
- File Members

# core/simulator/physics\_engine/physics\_engine.h

Go to the documentation of this file.

```
00001 
00007 #ifndef PHYSICS_ENGINE_H
00008 #define PHYSICS_ENGINE_H
00009 
00010 namespace argos {
00011    class CPhysicsEngine;
00012    class CPhysicsModel;
00013    class CEntity;
00014    class CEmbodiedEntity;
00015    class CVector3;
00016    class CRay3;
00017 }
00018 
00019 #include <map>
00020 #include <argos3/core/utility/logging/argos_log.h>
00021 #include <argos3/core/utility/math/ray2.h>
00022 #include <argos3/core/utility/configuration/base_configurable_resource.h>
00023 #include <argos3/core/utility/configuration/argos_configuration.h>
00024 #include <argos3/core/utility/datatypes/datatypes.h>
00025 #include <argos3/core/utility/plugins/factory.h>
00026 
00027 namespace argos {
00028 
00029    /****************************************/
00030    /****************************************/
00031 
00032    struct SEmbodiedEntityIntersectionItem {
00033       CEmbodiedEntity* IntersectedEntity;
00034       Real TOnRay;
00035 
00036       SEmbodiedEntityIntersectionItem() :
00037          IntersectedEntity(NULL),
00038          TOnRay(1.0f) {}
00039 
00040       SEmbodiedEntityIntersectionItem(CEmbodiedEntity* pc_entity,
00041                                       Real f_t_on_ray) :
00042          IntersectedEntity(pc_entity),
00043          TOnRay(f_t_on_ray) {}
00044 
00045       SEmbodiedEntityIntersectionItem(const SEmbodiedEntityIntersectionItem& s_item) :
00046          IntersectedEntity(s_item.IntersectedEntity),
00047          TOnRay(s_item.TOnRay) {}
00048 
00049       inline bool operator<(const SEmbodiedEntityIntersectionItem& s_item) {
00050          return TOnRay < s_item.TOnRay;
00051       }
00052    };
00053 
00054    typedef std::vector<SEmbodiedEntityIntersectionItem> TEmbodiedEntityIntersectionData;
00055 
00063    extern bool GetEmbodiedEntitiesIntersectedByRay(TEmbodiedEntityIntersectionData& t_data,
00064                                                    const CRay3& c_ray);
00065 
00072    extern bool GetClosestEmbodiedEntityIntersectedByRay(SEmbodiedEntityIntersectionItem& s_item,
00073                                                         const CRay3& c_ray);
00074 
00083    extern bool GetClosestEmbodiedEntityIntersectedByRay(SEmbodiedEntityIntersectionItem& s_item,
00084                                                         const CRay3& c_ray,
00085                                                         CEmbodiedEntity& c_entity);
00086 
00087    /****************************************/
00088    /****************************************/
00089 
00090    class CPhysicsEngine : public CBaseConfigurableResource {
00091 
00092    public:
00093 
00097       struct SBoundaryFace {
00098          std::string EngineId;
00099       };
00100 
00104       struct SHorizontalFace : public SBoundaryFace {
00105          Real Height;
00106       };
00107 
00111       struct SVerticalFace : public SBoundaryFace {
00112          CRay2 BaseSegment;
00113       };
00114 
00118       struct SVolume {
00119          SHorizontalFace*            TopFace;
00120          SHorizontalFace*            BottomFace;
00121          std::vector<SVerticalFace*> SideFaces;
00122          SVolume();
00123          ~SVolume();
00124          void Init(TConfigurationNode& t_node);
00125          bool IsActive() const;
00126       };
00127       
00128    public:
00129 
00130       typedef std::vector<CPhysicsEngine*> TVector;
00131       typedef std::map<std::string, CPhysicsEngine*, std::less<std::string> > TMap;
00132 
00133    public:
00134 
00135       CPhysicsEngine();
00136       virtual ~CPhysicsEngine() {}
00137 
00138       virtual void Init(TConfigurationNode& t_tree);
00139       virtual void Reset() {}
00140       virtual void Destroy() {}
00141 
00142       virtual void Update() = 0;
00143 
00157       virtual void PostSpaceInit() {}
00158 
00162       virtual bool IsPointContained(const CVector3& c_point);
00163 
00164       virtual size_t GetNumPhysicsModels() = 0;
00165 
00172       virtual bool AddEntity(CEntity& c_entity) = 0;
00173 
00180       virtual bool RemoveEntity(CEntity& c_entity) = 0;
00181 
00185       inline bool IsEntityTransferNeeded() const {
00186          return !m_vecTransferData.empty();
00187       }
00188 
00192       inline bool IsEntityTransferActive() const {
00193          return m_sVolume.IsActive();
00194       }
00195 
00201       virtual void ScheduleEntityForTransfer(CEmbodiedEntity& c_entity);
00202 
00206       virtual void TransferEntities();
00207 
00211       inline SVolume& GetVolume() {
00212          return m_sVolume;
00213       }
00214 
00218       inline const SVolume& GetVolume() const {
00219          return m_sVolume;
00220       }
00221 
00227       virtual void CheckIntersectionWithRay(TEmbodiedEntityIntersectionData& t_data,
00228                                             const CRay3& c_ray) const = 0;
00229 
00238       static Real GetSimulationClockTick();
00239 
00244       static Real GetInverseSimulationClockTick();
00245 
00255       static void SetSimulationClockTick(Real f_simulation_clock_tick);
00256 
00266       inline UInt32 GetIterations() const {
00267          return m_unIterations;
00268       }
00269 
00276       inline Real GetPhysicsClockTick() const {
00277          return m_fPhysicsClockTick;
00278       }
00279 
00284       inline const std::string& GetId() const {
00285          return m_strId;
00286       }
00287 
00292       void SetId(const std::string& str_id) {
00293          m_strId = str_id;
00294       }
00295                
00296    private:
00297 
00299       UInt32 m_unIterations;
00300 
00302       Real m_fPhysicsClockTick;
00303 
00305       std::string m_strId;
00306 
00308       static Real m_fSimulationClockTick;
00309 
00311       static Real m_fInverseSimulationClockTick;
00312 
00314       SVolume m_sVolume;
00315 
00317       std::vector<CEmbodiedEntity*> m_vecTransferData;
00318    };
00319 
00320 }
00321 
00322 #define REGISTER_PHYSICS_ENGINE(CLASSNAME,          \
00323                                 LABEL,              \
00324                                 AUTHOR,             \
00325                                 VERSION,            \
00326                                 BRIEF_DESCRIPTION,  \
00327                                 LONG_DESCRIPTION,   \
00328                                 STATUS)             \
00329    REGISTER_SYMBOL(CPhysicsEngine,                  \
00330                    CLASSNAME,                       \
00331                    LABEL,                           \
00332                    AUTHOR,                          \
00333                    VERSION,                         \
00334                    BRIEF_DESCRIPTION,               \
00335                    LONG_DESCRIPTION,                \
00336                    STATUS)
00337 
00338 #endif
```

---

Generated on 10 Jul 2018 for ARGoS by 
 1.6.1 
