# Supplementary material for: Iterative improvement in the automatic modular design of robot swarms
Source: PeerJ Comput Sci. 2020 Dec 7;6:e322. doi: 10.7717/peerj-cs.322 (PMC7924708; doi:10.7717/peerj-cs.322)
Supplement: Supplemental Information 3 [file peerj-cs-06-322-s003.zip › argos3/doc/api/standalone/a00325_source.html]

ARGoS: core/simulator/physics\_engine/physics\_model.cpp Source File


- Main Page
- Related Pages
- Namespaces
- Classes
- Files

- File List
- File Members

# core/simulator/physics\_engine/physics\_model.cpp

Go to the documentation of this file.

```
00001 #include "physics_model.h"
00002 #include <argos3/core/simulator/entity/embodied_entity.h>
00003 #include <argos3/core/simulator/entity/composable_entity.h>
00004 
00005 namespace argos {
00006 
00007    /****************************************/
00008    /****************************************/
00009 
00010    SAnchor::SAnchor(CEmbodiedEntity& c_body,
00011                     const std::string& str_id,
00012                     UInt32 un_index,
00013                     const CVector3& c_offset_position,
00014                     const CQuaternion& c_offset_orientation,
00015                     const CVector3& c_position,
00016                     const CQuaternion& c_orientation) :
00017       Body(c_body),
00018       Id(str_id),
00019       Index(un_index),
00020       OffsetPosition(c_offset_position),
00021       OffsetOrientation(c_offset_orientation),
00022       Position(c_position),
00023       Orientation(c_orientation),
00024       InUseCount(0) {
00025    }
00026 
00027    /****************************************/
00028    /****************************************/
00029 
00030    void SAnchor::Enable() {
00031       Body.EnableAnchor(Id);
00032    }
00033 
00034    /****************************************/
00035    /****************************************/
00036 
00037    void SAnchor::Disable() {
00038       Body.DisableAnchor(Id);
00039    }
00040 
00041    /****************************************/
00042    /****************************************/
00043 
00044    CPhysicsModel::CPhysicsModel(CPhysicsEngine& c_engine,
00045                                 CEmbodiedEntity& c_entity) :
00046       m_cEngine(c_engine),
00047       m_cEmbodiedEntity(c_entity),
00048       m_sBoundingBox(),
00049       m_vecAnchorMethodHolders(c_entity.GetAnchors().size(), NULL),
00050       m_vecThunks(c_entity.GetAnchors().size(), NULL) {}
00051 
00052    /****************************************/
00053    /****************************************/
00054 
00055    void CPhysicsModel::UpdateEntityStatus() {
00056       CalculateAnchors();
00057       CalculateBoundingBox();
00058       /*
00059        * Update entity components
00060        */
00061       /* Get a reference to the root entity */
00062       /* NOTE: here the cast is static because we know that an embodied entity MUST have a parent
00063        * which, by definition, is a composable entity */
00064       CComposableEntity& cRoot = static_cast<CComposableEntity&>(m_cEmbodiedEntity.GetRootEntity());
00065       /* Update its components */
00066       cRoot.UpdateComponents();
00067       /*
00068        * Check whether a transfer is necessary
00069        */
00070       if(!m_cEngine.IsPointContained(GetEmbodiedEntity().GetOriginAnchor().Position))
00071          m_cEngine.ScheduleEntityForTransfer(m_cEmbodiedEntity);
00072    }
00073 
00074    /****************************************/
00075    /****************************************/
00076 
00077    void CPhysicsModel::CalculateAnchors() {
00078       std::vector<SAnchor*>& vecAnchors = m_cEmbodiedEntity.GetEnabledAnchors();
00079       for(size_t i = 0; i < vecAnchors.size(); ++i) {
00080          if(m_vecThunks[vecAnchors[i]->Index] != NULL) {
00081             TThunk tThunk = m_vecThunks[vecAnchors[i]->Index];
00082             (this->*tThunk)(*vecAnchors[i]);
00083          }
00084       }
00085    }
00086 
00087    /****************************************/
00088    /****************************************/
00089 
00090 }
```

---

Generated on 10 Jul 2018 for ARGoS by 
 1.6.1 
