# Supplementary material for: Iterative improvement in the automatic modular design of robot swarms
Source: PeerJ Comput Sci. 2020 Dec 7;6:e322. doi: 10.7717/peerj-cs.322 (PMC7924708; doi:10.7717/peerj-cs.322)
Supplement: Supplemental Information 3 [file peerj-cs-06-322-s003.zip › argos3/doc/api/standalone/a00326_source.html]

ARGoS: core/simulator/physics\_engine/physics\_model.h Source File


- Main Page
- Related Pages
- Namespaces
- Classes
- Files

- File List
- File Members

# core/simulator/physics\_engine/physics\_model.h

Go to the documentation of this file.

```
00001 
00007 #ifndef PHYSICS_MODEL_H
00008 #define PHYSICS_MODEL_H
00009 
00010 namespace argos {
00011    class CPhysicsModel;
00012    class CPhysicsEngine;
00013    class CRay3;
00014    class CQuaternion;
00015    class CEmbodiedEntity;
00016    struct SAnchor;
00017 }
00018 
00019 #include <argos3/core/utility/datatypes/datatypes.h>
00020 #include <argos3/core/utility/math/vector3.h>
00021 #include <argos3/core/utility/math/quaternion.h>
00022 #include <map>
00023 #include <vector>
00024 #include <string>
00025 
00026 namespace argos {
00027 
00028    /****************************************/
00029    /****************************************/
00030 
00038    struct SAnchor {
00040       CEmbodiedEntity& Body;
00042       std::string Id;
00045       UInt32 Index;
00047       CVector3 OffsetPosition;
00049       CQuaternion OffsetOrientation;
00051       CVector3 Position;
00053       CQuaternion Orientation;
00055       UInt32 InUseCount;
00067       SAnchor(CEmbodiedEntity& c_body,
00068               const std::string& str_id,
00069               UInt32 un_index,
00070               const CVector3& c_offset_position,
00071               const CQuaternion& c_offset_orientation,
00072               const CVector3& c_position,
00073               const CQuaternion& c_orientation);
00077       void Enable();
00081       void Disable();
00082    };
00083 
00084    /****************************************/
00085    /****************************************/
00086 
00087    struct SBoundingBox {
00088       CVector3 MinCorner;
00089       CVector3 MaxCorner;
00090 
00091       inline bool Intersects(const SBoundingBox& s_bb) const {
00092          return
00093             (MinCorner.GetX() < s_bb.MaxCorner.GetX()) && (MaxCorner.GetX() > s_bb.MinCorner.GetX()) &&
00094             (MinCorner.GetY() < s_bb.MaxCorner.GetY()) && (MaxCorner.GetY() > s_bb.MinCorner.GetY()) &&
00095             (MinCorner.GetZ() < s_bb.MaxCorner.GetZ()) && (MaxCorner.GetZ() > s_bb.MinCorner.GetZ());
00096       }
00097    };
00098 
00099    /****************************************/
00100    /****************************************/
00101 
00102    class CPhysicsModel {
00103 
00104    public:
00105 
00106       typedef std::map<std::string, CPhysicsModel*> TMap;
00107       typedef std::vector<CPhysicsModel*> TVector;
00108 
00109    public:
00110 
00111       CPhysicsModel(CPhysicsEngine& c_engine,
00112                     CEmbodiedEntity& c_entity);
00113 
00114       virtual ~CPhysicsModel() {
00115          while(!m_vecAnchorMethodHolders.empty()) {
00116             delete m_vecAnchorMethodHolders.back();
00117             m_vecAnchorMethodHolders.pop_back();
00118          }
00119       }
00120 
00125       inline CPhysicsEngine& GetEngine() {
00126          return m_cEngine;
00127       }
00128 
00133       inline CEmbodiedEntity& GetEmbodiedEntity() {
00134          return m_cEmbodiedEntity;
00135       }
00136 
00141       inline const CEmbodiedEntity& GetEmbodiedEntity() const {
00142          return m_cEmbodiedEntity;
00143       }
00144 
00160       virtual void UpdateEntityStatus();
00161 
00168       virtual void UpdateFromEntityStatus() = 0;
00169 
00190       virtual void MoveTo(const CVector3& c_position,
00191                           const CQuaternion& c_orientation) = 0;
00192 
00198       inline const SBoundingBox& GetBoundingBox() const {
00199          return m_sBoundingBox;
00200       }
00201 
00206       virtual void CalculateBoundingBox() = 0;
00207 
00213       virtual void CalculateAnchors();
00214 
00219       virtual bool IsCollidingWithSomething() const = 0;
00220 
00226       inline SBoundingBox& GetBoundingBox() {
00227          return m_sBoundingBox;
00228       }
00229 
00230    private:
00231 
00232       CPhysicsEngine& m_cEngine;
00233       CEmbodiedEntity& m_cEmbodiedEntity;
00234       SBoundingBox m_sBoundingBox;
00235 
00236    private:
00237 
00242       typedef void (CPhysicsModel::*TThunk)(SAnchor&);
00243 
00248       class CAnchorMethodHolder {};
00249 
00256       template <typename MODEL> class CAnchorMethodHolderImpl : public CAnchorMethodHolder {
00257       public:
00258          typedef void (MODEL::*TMethod)(SAnchor&);
00259          TMethod Method;
00260          CAnchorMethodHolderImpl(TMethod t_method) : Method(t_method) {}
00261       };
00262 
00263    private:
00264 
00273       template <typename USER_IMPL>
00274       void Thunk(SAnchor& s_anchor);
00275 
00276    private:
00277 
00282       std::vector<CAnchorMethodHolder*> m_vecAnchorMethodHolders;
00283 
00289       std::vector<TThunk> m_vecThunks;
00290 
00291    public:
00292 
00299       template <typename MODEL>
00300       void RegisterAnchorMethod(const SAnchor& s_anchor,
00301                                 void(MODEL::*pt_method)(SAnchor&));
00302 
00303    };
00304 
00305    /****************************************/
00306    /****************************************/
00307 
00308    template <typename MODEL>
00309    void CPhysicsModel::Thunk(SAnchor& s_anchor) {
00310       /*
00311        * When this method is called, the static type of 'this'
00312        * is CPhysicsModel. Since we want to call
00313        * method in MODEL (subclass of CPhysicsModel),
00314        * we need a cast. The cast is static because we trust
00315        * the user on not doing anything stupid.
00316        */
00317       MODEL& cImpl = static_cast<MODEL&>(*this);
00318       /* Cast the method holder to its effective type */
00319       CAnchorMethodHolderImpl<MODEL>& cMethodHolder = static_cast<CAnchorMethodHolderImpl<MODEL>&>(*m_vecAnchorMethodHolders[s_anchor.Index]);
00320       /* Call the user-defined method */
00321       (cImpl.*(cMethodHolder.Method))(s_anchor);
00322    }
00323 
00324    template <typename MODEL>
00325    void CPhysicsModel::RegisterAnchorMethod(const SAnchor& s_anchor,
00326                                             void(MODEL::*pt_method)(SAnchor&)) {
00327       /* Add the thunk to the VTable */
00328       m_vecThunks[s_anchor.Index] = &CPhysicsModel::Thunk<MODEL>;
00329       /* Add the method holder to the map */
00330       m_vecAnchorMethodHolders[s_anchor.Index] = new CAnchorMethodHolderImpl<MODEL>(pt_method);
00331    }
00332 
00333    /****************************************/
00334    /****************************************/
00335 
00336 }
00337 
00338 #endif
```

---

Generated on 10 Jul 2018 for ARGoS by 
 1.6.1 
