# Supplementary material for: Iterative improvement in the automatic modular design of robot swarms
Source: PeerJ Comput Sci. 2020 Dec 7;6:e322. doi: 10.7717/peerj-cs.322 (PMC7924708; doi:10.7717/peerj-cs.322)
Supplement: Supplemental Information 3 [file peerj-cs-06-322-s003.zip › argos3/doc/api/standalone/a00327_source.html]

ARGoS: core/simulator/query\_plugins.cpp Source File


- Main Page
- Related Pages
- Namespaces
- Classes
- Files

- File List
- File Members

# core/simulator/query\_plugins.cpp

Go to the documentation of this file.

```
00001 
00007 #include "query_plugins.h"
00008 #include <argos3/core/simulator/visualization/visualization.h>
00009 #include <argos3/core/simulator/physics_engine/physics_engine.h>
00010 #include <argos3/core/simulator/entity/entity.h>
00011 #include <argos3/core/simulator/actuator.h>
00012 #include <argos3/core/simulator/sensor.h>
00013 
00014 namespace argos {
00015 
00016    /****************************************/
00017    /****************************************/
00018 
00019    void QueryShowPluginDescription(const std::string& str_query) {
00020       LOG << "Plugins matching \"" << str_query << "\":" << std::endl << std::endl;
00021       /* Buffer to contain the search results */
00022       TQueryResult tResult;
00023       /* Search among the plugins */
00024       QuerySearchPlugins<CSimulatedActuator>(str_query, tResult);
00025       QuerySearchPlugins<CSimulatedSensor>  (str_query, tResult);
00026       QuerySearchPlugins<CPhysicsEngine>    (str_query, tResult);
00027       QuerySearchPlugins<CMedium>           (str_query, tResult);
00028       QuerySearchPlugins<CVisualization>    (str_query, tResult);
00029       QuerySearchPlugins<CEntity>           (str_query, tResult);
00030       /* Print the result */
00031       if(tResult.empty()) {
00032          LOG << "   None found." << std::endl << std::endl;
00033       }
00034       else {
00035          LOG << "==============================================================================" << std::endl << std::endl;
00036          for(UInt32 i = 0; i < tResult.size(); ++i) {
00037             LOG << "[ " << tResult[i].Label << " ] " << std::endl;
00038             LOG << tResult[i].BriefDescription << std::endl;
00039             LOG << "by " << tResult[i].Author << std::endl;
00040             LOG << "Version: " << tResult[i].Version << std::endl;
00041             LOG << "Status: " << tResult[i].Status << std::endl << std::endl;
00042             LOG << tResult[i].LongDescription << std::endl;
00043             LOG << std::endl;
00044             LOG << "==============================================================================" << std::endl << std::endl;
00045          }
00046       }
00047    }
00048 
00049    /****************************************/
00050    /****************************************/
00051 
00052    void QueryPlugins(const std::string& str_query) {
00053       bool bIsLogColored = LOG.IsColoredOutput();
00054       LOG.DisableColoredOutput();
00055       LOGERR.DisableColoredOutput();
00056       if(str_query == "actuators") {
00057          QueryShowList<CSimulatedActuator>("AVAILABLE ACTUATORS");
00058       } else if(str_query == "sensors") {
00059          QueryShowList<CSimulatedSensor>("AVAILABLE SENSORS");
00060       } else if(str_query == "physics_engines") {
00061          QueryShowList<CPhysicsEngine>("AVAILABLE PHYSICS ENGINES");
00062       } else if(str_query == "media") {
00063          QueryShowList<CMedium>("AVAILABLE MEDIA");
00064       } else if(str_query == "visualizations") {
00065          QueryShowList<CVisualization>("AVAILABLE VISUALIZATIONS");
00066       } else if(str_query == "entities") {
00067          QueryShowList<CEntity>("AVAILABLE ENTITIES");
00068       } else if(str_query == "all") {
00069          QueryShowList<CSimulatedActuator>("AVAILABLE ACTUATORS");
00070          QueryShowList<CSimulatedSensor>  ("AVAILABLE SENSORS");
00071          QueryShowList<CPhysicsEngine>    ("AVAILABLE PHYSICS ENGINES");
00072          QueryShowList<CMedium>           ("AVAILABLE MEDIA");
00073          QueryShowList<CVisualization>    ("AVAILABLE VISUALIZATIONS");
00074          QueryShowList<CEntity>           ("AVAILABLE ENTITIES");
00075       } else {
00076          QueryShowPluginDescription(str_query);
00077       }
00078       if(bIsLogColored) {
00079          LOG.EnableColoredOutput();
00080          LOGERR.EnableColoredOutput();
00081       }
00082    }
00083 
00084    /****************************************/
00085    /****************************************/
00086 
00087 }
```

---

Generated on 10 Jul 2018 for ARGoS by 
 1.6.1 
