# Supplementary material for: Iterative improvement in the automatic modular design of robot swarms
Source: PeerJ Comput Sci. 2020 Dec 7;6:e322. doi: 10.7717/peerj-cs.322 (PMC7924708; doi:10.7717/peerj-cs.322)
Supplement: Supplemental Information 3 [file peerj-cs-06-322-s003.zip › argos3/doc/api/standalone/a00328_source.html]

ARGoS: core/simulator/query\_plugins.h Source File


- Main Page
- Related Pages
- Namespaces
- Classes
- Files

- File List
- File Members

# core/simulator/query\_plugins.h

Go to the documentation of this file.

```
00001 
00007 #ifndef QUERY_PLUGINS_H
00008 #define QUERY_PLUGINS_H
00009 
00010 #include <argos3/core/utility/plugins/factory.h>
00011 #include <argos3/core/utility/logging/argos_log.h>
00012 
00013 #include <map>
00014 #include <vector>
00015 #include <string>
00016 
00017 namespace argos {
00018 
00019    /****************************************/
00020    /****************************************/
00021 
00022    struct SQueryResultItem {
00023       std::string Label;
00024       std::string Author;
00025       std::string Version;
00026       std::string BriefDescription;
00027       std::string LongDescription;
00028       std::string Status;
00029 
00030       SQueryResultItem(std::string str_label,
00031                        std::string str_author,
00032                        std::string str_version,
00033                        std::string str_brief_description,
00034                        std::string str_long_description,
00035                        std::string str_status) :
00036          Label(str_label),
00037          Author(str_author),
00038          Version(str_version),
00039          BriefDescription(str_brief_description),
00040          LongDescription(str_long_description),
00041          Status(str_status) {}
00042    };
00043 
00044    typedef std::vector<SQueryResultItem> TQueryResult;
00045 
00046    /****************************************/
00047    /****************************************/
00048 
00049    void QueryPlugins(const std::string& str_query);
00050 
00051    /****************************************/
00052    /****************************************/
00053 
00054    void QueryShowPluginDescription(const std::string& str_query);
00055 
00056    /****************************************/
00057    /****************************************/
00058 
00059    template <class TYPE>
00060    void QuerySearchPlugins(const std::string& str_query,
00061                            TQueryResult& t_result) {
00062       typename CFactory<TYPE>::TTypeMap& tTypeMap = CFactory<TYPE>::GetTypeMap();
00063       for(typename CFactory<TYPE>::TTypeMap::const_iterator it = tTypeMap.begin();
00064           it != tTypeMap.end();
00065           ++it) {
00066          /* If the current plugin name contains the passed query */
00067          if(it->first.find(str_query) != std::string::npos) {
00068             t_result.push_back(
00069                SQueryResultItem(
00070                   it->first,
00071                   it->second->Author,
00072                   it->second->Version,
00073                   it->second->BriefDescription,
00074                   it->second->LongDescription,
00075                   it->second->Status));
00076          }
00077       }
00078    }
00079 
00080    /****************************************/
00081    /****************************************/
00082 
00083    template <class TYPE>
00084    void QueryShowList(const std::string& str_header) {      
00085       typename CFactory<TYPE>::TTypeMap& tTypeMap = CFactory<TYPE>::GetTypeMap();
00086       LOG << str_header << std::endl << std::endl;
00087       for(typename CFactory<TYPE>::TTypeMap::iterator it = tTypeMap.begin();
00088           it != tTypeMap.end();
00089           ++it) {
00090          LOG << "   [ " << it->first << " ]" << std::endl;
00091          LOG << "      " << it->second->BriefDescription << std::endl << std::endl;
00092       }
00093    }
00094 
00095    /****************************************/
00096    /****************************************/
00097 
00098 }
00099 
00100 #endif
00101
```

---

Generated on 10 Jul 2018 for ARGoS by 
 1.6.1 
