# Supplementary material for: Iterative improvement in the automatic modular design of robot swarms
Source: PeerJ Comput Sci. 2020 Dec 7;6:e322. doi: 10.7717/peerj-cs.322 (PMC7924708; doi:10.7717/peerj-cs.322)
Supplement: Supplemental Information 3 [file peerj-cs-06-322-s003.zip › argos3/doc/api/standalone/a00329_source.html]

ARGoS: core/simulator/sensor.h Source File


- Main Page
- Related Pages
- Namespaces
- Classes
- Files

- File List
- File Members

# core/simulator/sensor.h

Go to the documentation of this file.

```
00001 
00007 #ifndef SENSOR_H
00008 #define SENSOR_H
00009 
00010 namespace argos {
00011    class CComposableEntity;
00012 }
00013 
00014 namespace argos {
00015 
00022    class CSimulatedSensor {
00023 
00024    public:
00025 
00029       virtual ~CSimulatedSensor() {}
00030 
00041       virtual void SetRobot(CComposableEntity& c_entity) = 0;
00042 
00046       virtual void Update() = 0;
00047 
00048    };
00049 
00050    /****************************************/
00051    /****************************************/
00052 
00053 }
00054 
00060 #define REGISTER_SENSOR(CLASSNAME,                          \
00061                         LABEL,                              \
00062                         IMPLEMENTATION,                     \
00063                         AUTHOR,                             \
00064                         VERSION,                            \
00065                         BRIEF_DESCRIPTION,                  \
00066                         LONG_DESCRIPTION,                   \
00067                         STATUS)                             \
00068    REGISTER_SYMBOL(CSimulatedSensor,                        \
00069                    CLASSNAME,                               \
00070                    LABEL " (" IMPLEMENTATION ")",           \
00071                    AUTHOR,                                  \
00072                    VERSION,                                 \
00073                    BRIEF_DESCRIPTION,                       \
00074                    LONG_DESCRIPTION,                        \
00075                    STATUS)
00076 
00077 #endif
```

---

Generated on 10 Jul 2018 for ARGoS by 
 1.6.1 
