# Supplementary material for: Iterative improvement in the automatic modular design of robot swarms
Source: PeerJ Comput Sci. 2020 Dec 7;6:e322. doi: 10.7717/peerj-cs.322 (PMC7924708; doi:10.7717/peerj-cs.322)
Supplement: Supplemental Information 3 [file peerj-cs-06-322-s003.zip › argos3/doc/api/standalone/a00330_source.html]

ARGoS: core/simulator/simulator.cpp Source File


- Main Page
- Related Pages
- Namespaces
- Classes
- Files

- File List
- File Members

# core/simulator/simulator.cpp

Go to the documentation of this file.

```
00001 
00007 #include "simulator.h"
00008 
00009 #include <iostream>
00010 #include <string>
00011 #include <sys/time.h>
00012 #include <argos3/core/utility/logging/argos_log.h>
00013 #include <argos3/core/utility/profiler/profiler.h>
00014 #include <argos3/core/utility/string_utilities.h>
00015 #include <argos3/core/utility/plugins/dynamic_loading.h>
00016 #include <argos3/core/utility/math/rng.h>
00017 #include <argos3/core/simulator/space/space_no_threads.h>
00018 #include <argos3/core/simulator/space/space_multi_thread_balance_quantity.h>
00019 #include <argos3/core/simulator/space/space_multi_thread_balance_length.h>
00020 #include <argos3/core/simulator/visualization/default_visualization.h>
00021 #include <argos3/core/simulator/physics_engine/physics_engine.h>
00022 #include <argos3/core/simulator/loop_functions.h>
00023 #include <argos3/core/simulator/entity/composable_entity.h>
00024 #include <argos3/core/simulator/entity/embodied_entity.h>
00025 
00026 namespace argos {
00027 
00031 #if __cplusplus >= 201103L
00032    template <typename T>
00033    using auto_ptr = std::unique_ptr<T>;
00034 #else
00035    using std::auto_ptr;
00036 #endif
00037 
00038    /****************************************/
00039    /****************************************/
00040 
00041    CSimulator::CSimulator() :
00042       m_pcVisualization(NULL),
00043       m_pcSpace(NULL),
00044       m_pcLoopFunctions(NULL),
00045       m_unMaxSimulationClock(0),
00046       m_bWasRandomSeedSet(false),
00047       m_unThreads(0),
00048       m_pcProfiler(NULL),
00049       m_bHumanReadableProfile(true),
00050       m_bRealTimeClock(false),
00051       m_bTerminated(false) {}
00052 
00053    /****************************************/
00054    /****************************************/
00055 
00056    CSimulator::~CSimulator() {
00057       if(IsProfiling()) {
00058          delete m_pcProfiler;
00059       }
00060       /* Delete the visualization */
00061       if(m_pcVisualization != NULL) delete m_pcVisualization;
00062       /* Delete all the media */
00063       for(CMedium::TMap::iterator it = m_mapMedia.begin();
00064           it != m_mapMedia.end(); ++it) {
00065          delete it->second;
00066       }
00067       m_mapMedia.clear();
00068       m_vecMedia.clear();
00069       /* Delete all the physics engines */
00070       for(CPhysicsEngine::TMap::iterator it = m_mapPhysicsEngines.begin();
00071           it != m_mapPhysicsEngines.end(); ++it) {
00072          delete it->second;
00073       }
00074       m_mapPhysicsEngines.clear();
00075       m_vecPhysicsEngines.clear();
00076       /* Delete the space and the dynamic linking manager */
00077       if(m_pcSpace != NULL) {
00078          delete m_pcSpace;
00079       }
00080       /* Get rid of all libraries */
00081       CDynamicLoading::UnloadAllLibraries();
00082    }
00083 
00084    /****************************************/
00085    /****************************************/
00086 
00087    CSimulator& CSimulator::GetInstance() {
00088       static auto_ptr<CSimulator> pcSimulatorInstance(new CSimulator());
00089       return *(pcSimulatorInstance.get());
00090    }
00091 
00092    /****************************************/
00093    /****************************************/
00094 
00095    CPhysicsEngine& CSimulator::GetPhysicsEngine(const std::string& str_id) const {
00096       CPhysicsEngine::TMap::const_iterator it = m_mapPhysicsEngines.find(str_id);
00097       ARGOS_ASSERT(it != m_mapPhysicsEngines.end(), "Physics engine \"" << str_id << "\" not found.")
00098          return *(it->second);
00099    }
00100 
00101    /****************************************/
00102    /****************************************/
00103 
00104    TConfigurationNode& CSimulator::GetConfigForController(const std::string& str_id) {
00105       TControllerConfigurationMap::iterator it = m_mapControllerConfig.find(str_id);
00106       if(it == m_mapControllerConfig.end()) {
00107          THROW_ARGOSEXCEPTION("Can't find XML configuration for controller id \"" << str_id << "\"");
00108       }
00109       return *(it->second);
00110    }
00111 
00112    /****************************************/
00113    /****************************************/
00114 
00115    void CSimulator::LoadExperiment() {
00116       /* Build configuration tree */
00117       m_tConfiguration.LoadFile(m_strExperimentConfigFileName);
00118       m_tConfigurationRoot = *m_tConfiguration.FirstChildElement();
00119       /* Init the experiment */
00120       Init();
00121       LOG.Flush();
00122       LOGERR.Flush();
00123    }
00124 
00125    /****************************************/
00126    /****************************************/
00127 
00128    void CSimulator::Init() {
00129       /* General configuration */
00130       InitFramework(GetNode(m_tConfigurationRoot, "framework"));
00131       /* Initialize controllers */
00132       InitControllers(GetNode(m_tConfigurationRoot, "controllers"));
00133       /* Create loop functions */
00134       if(NodeExists(m_tConfigurationRoot, "loop_functions")) {
00135          /* User specified a loop_functions section in the XML */
00136          InitLoopFunctions(GetNode(m_tConfigurationRoot, "loop_functions"));
00137       }
00138       else {
00139          /* No loop_functions in the XML */
00140          m_pcLoopFunctions = new CLoopFunctions;
00141       }
00142       /* Physics engines */
00143       InitPhysics(GetNode(m_tConfigurationRoot, "physics_engines"));
00144       /* Media */
00145       InitMedia(GetNode(m_tConfigurationRoot, "media"));
00146       /* Space */
00147       InitSpace(GetNode(m_tConfigurationRoot, "arena"));
00148       /* Call user init function */
00149       if(NodeExists(m_tConfigurationRoot, "loop_functions")) {
00150          m_pcLoopFunctions->Init(GetNode(m_tConfigurationRoot, "loop_functions"));
00151       }
00152       /* Physics engines */
00153       InitPhysics2();
00154       /* Media */
00155       InitMedia2();
00156       /* Initialise visualization */
00157       TConfigurationNodeIterator itVisualization;
00158       if(NodeExists(m_tConfigurationRoot, "visualization") &&
00159          ((itVisualization = itVisualization.begin(&GetNode(m_tConfigurationRoot, "visualization"))) != itVisualization.end())) {
00160          InitVisualization(GetNode(m_tConfigurationRoot, "visualization"));
00161       }
00162       else {
00163          LOG << "[INFO] No visualization selected." << std::endl;
00164          m_pcVisualization = new CDefaultVisualization();
00165       }
00166       /* Start profiling, if needed */
00167       if(IsProfiling()) {
00168          m_pcProfiler->Start();
00169       }
00170    }
00171 
00172    /****************************************/
00173    /****************************************/
00174 
00175    void CSimulator::Reset() {
00176       /* Reset terminated flag */
00177       m_bTerminated = false;
00178       /* if random seed is 0 or is not specified, init with the current timeval */
00179       if(m_bWasRandomSeedSet) {
00180          CRandom::SetSeedOf("argos", m_unRandomSeed);
00181       }
00182       else {
00183          /* Prepare the default value based on the current clock time */
00184          struct timeval sTimeValue;
00185          ::gettimeofday(&sTimeValue, NULL);
00186          UInt32 unSeed = static_cast<UInt32>(sTimeValue.tv_usec);
00187          CRandom::SetSeedOf("argos", unSeed);
00188          m_unRandomSeed = unSeed;
00189          LOG << "[INFO] Using random seed = " << m_unRandomSeed << std::endl;
00190       }
00191       CRandom::GetCategory("argos").ResetRNGs();
00192       /* Reset the space */
00193       m_pcSpace->Reset();
00194       /* Reset the media */
00195       for(CMedium::TMap::iterator it = m_mapMedia.begin();
00196           it != m_mapMedia.end(); ++it) {
00197          it->second->Reset();
00198       }
00199       /* Reset the physics engines */
00200       for(CPhysicsEngine::TMap::iterator it = m_mapPhysicsEngines.begin();
00201           it != m_mapPhysicsEngines.end(); ++it) {
00202          it->second->Reset();
00203       }
00204       /* Reset the loop functions */
00205       m_pcLoopFunctions->Reset();
00206       LOG.Flush();
00207       LOGERR.Flush();
00208    }
00209 
00210    /****************************************/
00211    /****************************************/
00212 
00213    void CSimulator::Destroy() {
00214       /* Call user destroy function */
00215       if (m_pcLoopFunctions != NULL) {
00216          m_pcLoopFunctions->Destroy();
00217          delete m_pcLoopFunctions;
00218          m_pcLoopFunctions = NULL;
00219       }
00220       /* Destroy the visualization */
00221       if(m_pcVisualization != NULL) {
00222          m_pcVisualization->Destroy();
00223       }
00224       /* Destroy simulated space */
00225       if(m_pcSpace != NULL) {
00226          m_pcSpace->Destroy();
00227       }
00228       /* Destroy media */
00229       for(CMedium::TMap::iterator it = m_mapMedia.begin();
00230           it != m_mapMedia.end(); ++it) {
00231          it->second->Destroy();
00232          delete it->second;
00233       }
00234       m_mapMedia.clear();
00235       m_vecMedia.clear();
00236       /* Destroy physics engines */
00237       for(CPhysicsEngine::TMap::iterator it = m_mapPhysicsEngines.begin();
00238           it != m_mapPhysicsEngines.end(); ++it) {
00239          it->second->Destroy();
00240          delete it->second;
00241       }
00242       m_mapPhysicsEngines.clear();
00243       m_vecPhysicsEngines.clear();
00244       /* Get rid of ARGoS category */
00245       if(CRandom::ExistsCategory("argos")) {
00246          CRandom::RemoveCategory("argos");
00247       }
00248       /* Free up factory data */
00249       CFactory<CMedium>::Destroy();
00250       CFactory<CPhysicsEngine>::Destroy();
00251       CFactory<CVisualization>::Destroy();
00252       CFactory<CSimulatedActuator>::Destroy();
00253       CFactory<CSimulatedSensor>::Destroy();
00254       CFactory<CCI_Controller>::Destroy();
00255       CFactory<CEntity>::Destroy();
00256       CFactory<CLoopFunctions>::Destroy();
00257       /* Stop profiling and flush the data */
00258       if(IsProfiling()) {
00259          m_pcProfiler->Stop();
00260          m_pcProfiler->Flush(m_bHumanReadableProfile);
00261       }
00262       LOG.Flush();
00263       LOGERR.Flush();
00264    }
00265 
00266    /****************************************/
00267    /****************************************/
00268 
00269    void CSimulator::Execute() {
00270       m_pcVisualization->Execute();
00271    }
00272 
00273    /****************************************/
00274    /****************************************/
00275 
00276    void CSimulator::UpdateSpace() {
00277       /* Update the space */
00278       m_pcSpace->Update();
00279    }
00280 
00281    /****************************************/
00282    /****************************************/
00283 
00284    bool CSimulator::IsExperimentFinished() const {
00285       /* Check if the simulation must be terminated */
00286       if(m_bTerminated) {
00287          return true;
00288       }
00289       /* Check simulation clock */
00290       if (m_unMaxSimulationClock > 0 &&
00291           m_pcSpace->GetSimulationClock() >= m_unMaxSimulationClock) {
00292          return true;
00293       }
00294       /* Call loop function */
00295       return m_pcLoopFunctions->IsExperimentFinished();
00296    }
00297 
00298    /****************************************/
00299    /****************************************/
00300 
00301    void CSimulator::InitFramework(TConfigurationNode& t_tree) {
00302       try {
00303          /* Parse the 'system' node */
00304          if(NodeExists(t_tree, "system")) {
00305             TConfigurationNode tSystem;
00306             tSystem = GetNode(t_tree, "system");
00307             GetNodeAttributeOrDefault(tSystem, "threads", m_unThreads, m_unThreads);
00308             if(m_unThreads == 0) {
00309                LOG << "[INFO] Not using threads" << std::endl;
00310                m_pcSpace = new CSpaceNoThreads();
00311             }
00312             else {
00313                LOG << "[INFO] Using " << m_unThreads << " parallel threads" << std::endl;
00314                std::string strThreadingMethod = "balance_quantity";
00315                GetNodeAttributeOrDefault(tSystem, "method", strThreadingMethod, strThreadingMethod);
00316                if(strThreadingMethod == "balance_quantity") {
00317                   LOG << "[INFO]   Chosen method \"balance_quantity\": threads will be assigned the same"
00318                       << std::endl
00319                       << "[INFO]   number of tasks, independently of the task length."
00320                       << std::endl;
00321                   m_pcSpace = new CSpaceMultiThreadBalanceQuantity();
00322                }
00323                else if(strThreadingMethod == "balance_length") {
00324                   LOG << "[INFO]   Chosen method \"balance_length\": threads will be assigned different"
00325                       << std::endl
00326                       << "[INFO]   numbers of tasks, depending on the task length."
00327                       << std::endl;
00328                   m_pcSpace = new CSpaceMultiThreadBalanceLength();
00329                }
00330                else {
00331                   THROW_ARGOSEXCEPTION("Error parsing the <system> tag. Unknown threading method \"" << strThreadingMethod << "\". Available methods: \"balance_quantity\" and \"balance_length\".");
00332                }
00333             }
00334          }
00335          else {
00336             LOG << "[INFO] Not using threads" << std::endl;
00337             m_pcSpace = new CSpaceNoThreads();
00338          }
00339          /* Get 'experiment' node */
00340          TConfigurationNode tExperiment;
00341          tExperiment = GetNode(t_tree, "experiment");
00342          /* Parse random seed */
00343          /* Buffer to hold the random seed */
00344          if(!m_bWasRandomSeedSet)
00345             GetNodeAttributeOrDefault(tExperiment,
00346                                       "random_seed",
00347                                       m_unRandomSeed,
00348                                       static_cast<UInt32>(0));
00349          /* if random seed is 0 or is not specified, init with the current timeval */
00350          if(m_unRandomSeed != 0) {
00351             CRandom::CreateCategory("argos", m_unRandomSeed);
00352             LOG << "[INFO] Using random seed = " << m_unRandomSeed << std::endl;
00353             m_bWasRandomSeedSet = true;
00354          }
00355          else {
00356             /* Prepare the default value based on the current clock time */
00357             m_bWasRandomSeedSet = false;
00358             struct timeval sTimeValue;
00359             ::gettimeofday(&sTimeValue, NULL);
00360             UInt32 unSeed = static_cast<UInt32>(sTimeValue.tv_usec);
00361             m_unRandomSeed = unSeed;
00362             CRandom::CreateCategory("argos", unSeed);
00363             LOG << "[INFO] Using random seed = " << unSeed << std::endl;
00364          }
00365          m_pcRNG = CRandom::CreateRNG("argos");
00366          /* Set the simulation clock tick length */
00367          UInt32 unTicksPerSec;
00368          GetNodeAttribute(tExperiment,
00369                           "ticks_per_second",
00370                           unTicksPerSec);
00371          CPhysicsEngine::SetSimulationClockTick(1.0 / static_cast<Real>(unTicksPerSec));
00372          /* Set the maximum simulation duration (in seconds) */
00373          Real fExpLength;
00374          GetNodeAttributeOrDefault<Real>(tExperiment,
00375                                          "length",
00376                                          fExpLength,
00377                                          0.0f);
00378          m_unMaxSimulationClock = fExpLength * unTicksPerSec;
00379          LOG << "[INFO] Total experiment length in clock ticks = "
00380              << (m_unMaxSimulationClock ? ToString(m_unMaxSimulationClock) : "unlimited")
00381              << std::endl;
00382          /* Check for the 'real_time' attribute */
00383          GetNodeAttributeOrDefault(tExperiment, "real_time", m_bRealTimeClock, m_bRealTimeClock);
00384          if(m_bRealTimeClock) {
00385             LOG << "[INFO] Using the real-time clock." << std::endl;
00386          }
00387          /* Get the profiling tag, if present */
00388          if(NodeExists(t_tree, "profiling")) {
00389             TConfigurationNode& tProfiling = GetNode(t_tree, "profiling");
00390             std::string strFile;
00391             GetNodeAttribute(tProfiling, "file", strFile);
00392             std::string strFormat;
00393             GetNodeAttribute(tProfiling, "format", strFormat);
00394             if(strFormat == "human_readable") {
00395                m_bHumanReadableProfile = true;
00396             }
00397             else if(strFormat == "table") {
00398                m_bHumanReadableProfile = false;
00399             }
00400             else {
00401                THROW_ARGOSEXCEPTION("Unrecognized profile format \"" << strFormat << "\". Accepted values are \"human_readable\" and \"table\".");
00402             }
00403             bool bTrunc = true;
00404             GetNodeAttributeOrDefault(tProfiling, "truncate_file", bTrunc, bTrunc);
00405             m_pcProfiler = new CProfiler(strFile, bTrunc);
00406          }
00407       }
00408       catch(CARGoSException& ex) {
00409          THROW_ARGOSEXCEPTION_NESTED("Failed to initialize the simulator. Parse error inside the <framework> tag.", ex);
00410       }
00411    }
00412 
00413    /****************************************/
00414    /****************************************/
00415 
00416    void CSimulator::InitLoopFunctions(TConfigurationNode& t_tree) {
00417       try {
00418          std::string strLibrary, strLabel;
00419          GetNodeAttributeOrDefault(t_tree, "library", strLibrary, strLibrary);
00420          GetNodeAttribute(t_tree, "label", strLabel);
00421          if(! strLibrary.empty()) {
00422             CDynamicLoading::LoadLibrary(strLibrary);
00423          }
00424          m_pcLoopFunctions = CFactory<CLoopFunctions>::New(strLabel);
00425       }
00426       catch(CARGoSException& ex) {
00427          THROW_ARGOSEXCEPTION_NESTED("Error initializing loop functions", ex);
00428       }
00429    }
00430 
00431    /****************************************/
00432    /****************************************/
00433 
00434    void CSimulator::InitControllers(TConfigurationNode& t_tree) {
00435       /*
00436        * Go through controllers, loading the library of each of them
00437        * and storing type, id and XML tree of each of them for later use
00438        */
00439       if(! t_tree.NoChildren()) {
00440          try {
00441             std::string strLibrary;
00442             std::string strId;
00443             TConfigurationNodeIterator it;
00444             for(it = it.begin(&t_tree);
00445                 it != it.end(); ++it) {
00446                /* Get controller id */
00447                try {
00448                   GetNodeAttribute(*it, "id", strId);
00449                }
00450                catch(CARGoSException& ex) {
00451                   std::string strValue;
00452                   it->GetValue(&strValue);
00453                   THROW_ARGOSEXCEPTION_NESTED("Controller type \"" << strValue << "\" has no assigned id.", ex);
00454                }
00455                /* Bomb out if id is already in map */
00456                if(m_mapControllerConfig.find(strId) != m_mapControllerConfig.end()) {
00457                   THROW_ARGOSEXCEPTION("Controller id \"" << strId << "\" duplicated");
00458                }
00459                /* Optionally, process "library" attribute if present */
00460                if(NodeAttributeExists(*it, "library")) {
00461                   /* Get library name */
00462                   GetNodeAttribute(*it, "library", strLibrary);
00463                   /* Load library */
00464                   CDynamicLoading::LoadLibrary(strLibrary);
00465                }
00466                /* Store XML info in map by id */
00467                m_mapControllerConfig.insert(std::pair<std::string, TConfigurationNode*>(strId, &(*it)));
00468             }
00469          }
00470          catch(CARGoSException& ex) {
00471             THROW_ARGOSEXCEPTION_NESTED("Error initializing controllers", ex);
00472          }
00473       }
00474    }
00475 
00476    /****************************************/
00477    /****************************************/
00478 
00479    void CSimulator::InitSpace(TConfigurationNode& t_tree) {
00480       try {
00481          m_pcSpace->Init(t_tree);
00482       }
00483       catch(CARGoSException& ex) {
00484          THROW_ARGOSEXCEPTION_NESTED("Failed to initialize the space.", ex);
00485       }
00486    }
00487 
00488    /****************************************/
00489    /****************************************/
00490 
00491    void CSimulator::InitPhysics(TConfigurationNode& t_tree) {
00492       try {
00493          /* Cycle through the physics engines */
00494          TConfigurationNodeIterator itEngines;
00495          for(itEngines = itEngines.begin(&t_tree);
00496              itEngines != itEngines.end();
00497              ++itEngines) {
00498             /* Create the physics engine */
00499             CPhysicsEngine* pcEngine = CFactory<CPhysicsEngine>::New(itEngines->Value());
00500             try {
00501                /* Initialize the engine */
00502                pcEngine->Init(*itEngines);
00503                /* Check that an engine with that ID does not exist yet */
00504                if(m_mapPhysicsEngines.find(pcEngine->GetId()) == m_mapPhysicsEngines.end()) {
00505                   /* Add it to the lists */
00506                   m_mapPhysicsEngines[pcEngine->GetId()] = pcEngine;
00507                   m_vecPhysicsEngines.push_back(pcEngine);
00508                }
00509                else {
00510                   /* Duplicate id -> error */
00511                   THROW_ARGOSEXCEPTION("A physics engine with id \"" << pcEngine->GetId() << "\" exists already. The ids must be unique!");
00512                }
00513             }
00514             catch(CARGoSException& ex) {
00515                /* Error while executing engine init, destroy what done to prevent memory leaks */
00516                pcEngine->Destroy();
00517                delete pcEngine;
00518                THROW_ARGOSEXCEPTION_NESTED("Error initializing physics engine type \"" << itEngines->Value() << "\"", ex);
00519             }
00520          }
00521       }
00522       catch(CARGoSException& ex) {
00523          THROW_ARGOSEXCEPTION_NESTED("Failed to initialize the physics engines. Parse error in the <physics_engines> subtree.", ex);
00524       }
00525    }
00526 
00527    /****************************************/
00528    /****************************************/
00529 
00530    void CSimulator::InitPhysics2() {
00531       try {
00532          /* Cycle through the physics engines */
00533          CPhysicsEngine::TMap::iterator it;
00534          for(it = m_mapPhysicsEngines.begin(); it != m_mapPhysicsEngines.end(); ++it) {
00535             CPhysicsEngine& cPhysicsEngine = *(it->second);
00536             try {
00537                /* Initialize the physicsengine */
00538                cPhysicsEngine.PostSpaceInit();
00539             }
00540             catch(CARGoSException& ex) {
00541                /* Error while executing physicsengine post space init, destroy what done to prevent memory leaks */
00542                std::ostringstream ossMsg;
00543                ossMsg << "Error executing post-space initialization of physics engine \"" << cPhysicsEngine.GetId() << "\"";
00544                cPhysicsEngine.Destroy();
00545                THROW_ARGOSEXCEPTION_NESTED(ossMsg.str(), ex);
00546             }
00547          }
00548       }
00549       catch(CARGoSException& ex) {
00550          THROW_ARGOSEXCEPTION_NESTED("Failed to initialize the physics engines. Parse error in the <physics_engines> subtree.", ex);
00551       }
00552    }
00553 
00554    /****************************************/
00555    /****************************************/
00556 
00557    void CSimulator::InitMedia(TConfigurationNode& t_tree) {
00558       try {
00559          /* Cycle through the media */
00560          TConfigurationNodeIterator itMedia;
00561          for(itMedia = itMedia.begin(&t_tree);
00562              itMedia != itMedia.end();
00563              ++itMedia) {
00564             /* Create the  medium */
00565             CMedium* pcMedium = CFactory<CMedium>::New(itMedia->Value());
00566             try {
00567                /* Initialize the medium */
00568                pcMedium->Init(*itMedia);
00569                /* Check that an medium with that ID does not exist yet */
00570                if(m_mapMedia.find(pcMedium->GetId()) == m_mapMedia.end()) {
00571                   /* Add it to the lists */
00572                   m_mapMedia[pcMedium->GetId()] = pcMedium;
00573                   m_vecMedia.push_back(pcMedium);
00574                }
00575                else {
00576                   /* Duplicate id -> error */
00577                   THROW_ARGOSEXCEPTION("A medium with id \"" << pcMedium->GetId() << "\" exists already. The ids must be unique!");
00578                }
00579             }
00580             catch(CARGoSException& ex) {
00581                /* Error while executing medium init, destroy what done to prevent memory leaks */
00582                pcMedium->Destroy();
00583                delete pcMedium;
00584                THROW_ARGOSEXCEPTION_NESTED("Error initializing medium type \"" << itMedia->Value() << "\"", ex);
00585             }
00586          }
00587       }
00588       catch(CARGoSException& ex) {
00589          THROW_ARGOSEXCEPTION_NESTED("Failed to initialize the media. Parse error in the <media> subtree.", ex);
00590       }
00591    }
00592 
00593    /****************************************/
00594    /****************************************/
00595 
00596    void CSimulator::InitMedia2() {
00597       try {
00598          /* Cycle through the media */
00599          CMedium::TMap::iterator it;
00600          for(it = m_mapMedia.begin(); it != m_mapMedia.end(); ++it) {
00601             CMedium& cMedium = *(it->second);
00602             try {
00603                /* Initialize the medium */
00604                cMedium.PostSpaceInit();
00605             }
00606             catch(CARGoSException& ex) {
00607                /* Error while executing medium post space init, destroy what done to prevent memory leaks */
00608                std::ostringstream ossMsg;
00609                ossMsg << "Error executing post-space initialization of medium \"" << cMedium.GetId() << "\"";
00610                cMedium.Destroy();
00611                THROW_ARGOSEXCEPTION_NESTED(ossMsg.str(), ex);
00612             }
00613          }
00614       }
00615       catch(CARGoSException& ex) {
00616          THROW_ARGOSEXCEPTION_NESTED("Failed to initialize the media. Parse error in the <media> subtree.", ex);
00617       }
00618    }
00619 
00620    /****************************************/
00621    /****************************************/
00622 
00623    void CSimulator::InitVisualization(TConfigurationNode& t_tree) {
00624       try {
00625          /* Consider only the first visualization */
00626          TConfigurationNodeIterator itVisualization;
00627          itVisualization = itVisualization.begin(&t_tree);
00628          /* Create the visualization */
00629          m_pcVisualization = CFactory<CVisualization>::New(itVisualization->Value());
00630          /* Initialize the visualization */
00631          m_pcVisualization->Init(*itVisualization);
00632       }
00633       catch(CARGoSException& ex) {
00634          THROW_ARGOSEXCEPTION_NESTED("Failed to initialize the visualization. Parse error in the <visualization> subtree.", ex);
00635       }
00636    }
00637 
00638    /****************************************/
00639    /****************************************/
00640 
00641 }
```

---

Generated on 10 Jul 2018 for ARGoS by 
 1.6.1 
