# Supplementary material for: Iterative improvement in the automatic modular design of robot swarms
Source: PeerJ Comput Sci. 2020 Dec 7;6:e322. doi: 10.7717/peerj-cs.322 (PMC7924708; doi:10.7717/peerj-cs.322)
Supplement: Supplemental Information 3 [file peerj-cs-06-322-s003.zip › argos3/doc/api/standalone/a00331_source.html]

ARGoS: core/simulator/simulator.h Source File


- Main Page
- Related Pages
- Namespaces
- Classes
- Files

- File List
- File Members

# core/simulator/simulator.h

Go to the documentation of this file.

```
00001 
00023 #ifndef SIMULATOR_H
00024 #define SIMULATOR_H
00025 
00026 namespace argos {
00027    class CSimulator;
00028    class CLoopFunctions;
00029    class CVisualization;
00030    class CPhysicsEngine;
00031    class CMedium;
00032    class CSpace;
00033    class CProfiler;
00034 }
00035 
00036 #include <argos3/core/config.h>
00037 #include <argos3/core/utility/math/rng.h>
00038 #include <argos3/core/utility/configuration/argos_configuration.h>
00039 #include <argos3/core/utility/datatypes/datatypes.h>
00040 #include <argos3/core/simulator/physics_engine/physics_engine.h>
00041 #include <argos3/core/simulator/medium/medium.h>
00042 #include <string>
00043 #include <map>
00044 
00050 namespace argos {
00051 
00062    class CSimulator {
00063 
00064    private:
00065 
00069       CSimulator();
00070 
00075       CSimulator(const CSimulator&) {}
00076 
00081       CSimulator& operator=(const CSimulator&) {
00082          return *this;
00083       }
00084 
00085    public:
00086 
00090       ~CSimulator();
00091 
00098       static CSimulator& GetInstance();
00099 
00104       inline CSpace& GetSpace() const {
00105          return *m_pcSpace;
00106       }
00107 
00113       CPhysicsEngine& GetPhysicsEngine(const std::string& str_id) const;
00114 
00119       inline CPhysicsEngine::TVector& GetPhysicsEngines() {
00120          return m_vecPhysicsEngines;
00121       }
00122 
00128       template <typename T>
00129       T& GetMedium(const std::string& str_id) {
00130          CMedium::TMap::const_iterator it = m_mapMedia.find(str_id);
00131          if(it != m_mapMedia.end()) {
00132             T* pcMedium = dynamic_cast<T*>(it->second);
00133             if(pcMedium != NULL) {
00134                return *pcMedium;
00135             }
00136             else {
00137                THROW_ARGOSEXCEPTION("Medium \"" << str_id << "\" can't be converted to the wanted type");
00138             }
00139          }
00140          else {
00141             THROW_ARGOSEXCEPTION("Medium \"" << str_id << "\" not found.");
00142          }
00143       }
00144 
00149       inline CMedium::TVector& GetMedia() {
00150          return m_vecMedia;
00151       }
00152 
00157       inline CVisualization& GetVisualization() {
00158          ARGOS_ASSERT(m_pcVisualization != NULL, "No visualization specified in the XML file.");
00159          return *m_pcVisualization;
00160       }
00161 
00166       inline TConfigurationNode& GetConfigurationRoot() {
00167          return m_tConfigurationRoot;
00168       }
00169 
00174       inline CProfiler& GetProfiler() {
00175          return *m_pcProfiler;
00176       }
00177 
00182       inline bool IsProfiling() const {
00183          return m_pcProfiler != NULL;
00184       }
00185 
00191       inline UInt32 GetRandomSeed() const {
00192          return m_unRandomSeed;
00193       }
00194 
00200       inline void SetRandomSeed(UInt32 un_random_seed) {
00201          m_unRandomSeed = un_random_seed;
00202          m_bWasRandomSeedSet = true;
00203       }
00204 
00210       inline CRandom::CRNG* GetRNG() {
00211          return m_pcRNG;
00212       }
00213 
00219       inline const std::string& GetExperimentFileName() const {
00220          return m_strExperimentConfigFileName;
00221       }
00222 
00228       inline void SetExperimentFileName(const std::string& str_file_name) {
00229          m_strExperimentConfigFileName = str_file_name;
00230       }
00231 
00236       inline CLoopFunctions& GetLoopFunctions() {
00237          return *m_pcLoopFunctions;
00238       }
00239 
00244       inline void SetLoopFunctions(CLoopFunctions& c_loop_functions) {
00245          m_pcLoopFunctions = &c_loop_functions;
00246       }
00247 
00252       inline UInt32 GetMaxSimulationClock() const {
00253          return m_unMaxSimulationClock;
00254       }
00255 
00260       inline UInt32 GetNumThreads() const {
00261          return m_unThreads;
00262       }
00263 
00268       inline bool IsRealTimeClock() const {
00269          return m_bRealTimeClock;
00270       }
00271 
00276       inline void SetRealTimeClock(bool b_real_time) {
00277          m_bRealTimeClock = b_real_time;
00278       }
00279 
00283       inline void Terminate() {
00284          m_bTerminated = true;
00285       }
00286 
00292       inline std::string GetInstallationDirectory() const {
00293          return ARGOS_INSTALL_PREFIX;
00294       }
00295 
00299       TConfigurationNode& GetConfigForController(const std::string& str_id);
00300 
00306       void LoadExperiment();
00307 
00312       void Init();
00313 
00318       void Reset();
00319 
00329       inline void Reset(UInt32 un_new_random_seed) {
00330          SetRandomSeed(un_new_random_seed);
00331          Reset();
00332       }
00333 
00337       void Destroy();
00338 
00342       void Execute();
00343 
00347       void UpdateSpace();
00348 
00363       bool IsExperimentFinished() const;
00364       
00365    private:
00366 
00367       void InitFramework(TConfigurationNode& t_tree);
00368       void InitLoopFunctions(TConfigurationNode& t_tree);
00369       void InitControllers(TConfigurationNode& t_tree);
00370       void InitSpace(TConfigurationNode& t_tree);
00371       void InitPhysics(TConfigurationNode& t_tree);
00372       void InitPhysics2();
00373       void InitMedia(TConfigurationNode& t_tree);
00374       void InitMedia2();
00375       void InitVisualization(TConfigurationNode& t_tree);
00376 
00377    private:
00378 
00379       typedef std::map<std::string, TConfigurationNode*> TControllerConfigurationMap;
00380 
00381    private:
00382 
00386       TControllerConfigurationMap m_mapControllerConfig;
00387 
00391       CVisualization* m_pcVisualization;
00392 
00396       CPhysicsEngine::TMap m_mapPhysicsEngines;
00397 
00401       CPhysicsEngine::TVector m_vecPhysicsEngines;
00402 
00406       CMedium::TMap m_mapMedia;
00407 
00411       CMedium::TVector m_vecMedia;
00412 
00416       CSpace* m_pcSpace;
00417 
00421       CLoopFunctions* m_pcLoopFunctions;
00422 
00426       std::string m_strExperimentConfigFileName;
00427 
00431       UInt32 m_unMaxSimulationClock;
00432 
00436       UInt32 m_unRandomSeed;
00437 
00441       CRandom::CRNG* m_pcRNG;
00442 
00448       bool m_bWasRandomSeedSet;
00449 
00453       ticpp::Document m_tConfiguration;
00454 
00458       TConfigurationNode m_tConfigurationRoot;
00459 
00463       UInt32 m_unThreads;
00464 
00468       CProfiler* m_pcProfiler;
00469 
00473       bool m_bHumanReadableProfile;
00474 
00478       bool m_bRealTimeClock;
00479 
00483       bool m_bTerminated;
00484 
00485    };
00486 
00487 }
00488 
00489 #endif
```

---

Generated on 10 Jul 2018 for ARGoS by 
 1.6.1 
