# Supplementary material for: Iterative improvement in the automatic modular design of robot swarms
Source: PeerJ Comput Sci. 2020 Dec 7;6:e322. doi: 10.7717/peerj-cs.322 (PMC7924708; doi:10.7717/peerj-cs.322)
Supplement: Supplemental Information 3 [file peerj-cs-06-322-s003.zip › argos3/doc/api/standalone/a00332_source.html]

ARGoS: core/simulator/space/positional\_indices/grid.h Source File


- Main Page
- Related Pages
- Namespaces
- Classes
- Files

- File List
- File Members

# core/simulator/space/positional\_indices/grid.h

Go to the documentation of this file.

```
00001 #ifndef GRID_H
00002 #define GRID_H
00003 
00004 #include <argos3/core/utility/datatypes/set.h>
00005 #include <argos3/core/utility/math/range.h>
00006 #include <argos3/core/utility/math/ray3.h>
00007 #include <argos3/core/simulator/space/positional_indices/positional_index.h>
00008 
00009 namespace argos {
00010 
00011    template<class ENTITY>
00012    class CGrid : public CPositionalIndex<ENTITY> {
00013 
00014    public:
00015 
00016       typedef typename CPositionalIndex<ENTITY>::COperation CEntityOperation;
00017 
00018       struct SCell {
00019          CSet<ENTITY*> Entities;
00020          size_t Timestamp;
00021 
00022          SCell() : Timestamp(0) {}
00023          inline void Reset() {
00024             Entities.clear();
00025             Timestamp = 0;
00026          }
00027       };
00028 
00029       class CCellOperation {
00030       public:
00031          virtual ~CCellOperation() {}
00032          virtual bool operator()(SInt32 n_i,
00033                                  SInt32 n_j,
00034                                  SInt32 n_k,
00035                                  SCell& s_cell) = 0;
00036       };
00037 
00038    public:
00039 
00040       CGrid(const CVector3& c_area_min_corner,
00041             const CVector3& c_area_max_corner,
00042             SInt32 n_size_i,
00043             SInt32 n_size_j,
00044             SInt32 n_size_k);
00045 
00046       virtual ~CGrid();
00047 
00048       virtual void Init(TConfigurationNode& t_tree);
00049       virtual void Reset();
00050       virtual void Destroy();
00051 
00052       virtual void AddEntity(ENTITY& c_entity);
00053 
00054       virtual void RemoveEntity(ENTITY& c_entity);
00055 
00056       virtual void Update();
00057 
00058       virtual void GetEntitiesAt(CSet<ENTITY*>& c_entities,
00059                                  const CVector3& c_position) const;
00060 
00061       virtual void ForAllEntities(CEntityOperation& c_operation);
00062 
00063       virtual void ForEntitiesInSphereRange(const CVector3& c_center,
00064                                             Real f_radius,
00065                                             CEntityOperation& c_operation);
00066 
00067       virtual void ForEntitiesInBoxRange(const CVector3& c_center,
00068                                          const CVector3& c_half_size,
00069                                          CEntityOperation& c_operation);
00070 
00071       virtual void ForEntitiesInCircleRange(const CVector3& c_center,
00072                                             Real f_radius,
00073                                             CEntityOperation& c_operation);
00074 
00075       virtual void ForEntitiesInRectangleRange(const CVector3& c_center,
00076                                                const CVector2& c_half_size,
00077                                                CEntityOperation& c_operation);
00078 
00079       virtual void ForEntitiesAlongRay(const CRay3& c_ray,
00080                                        CEntityOperation& c_operation,
00081                                        bool b_stop_at_closest_match = false);
00082 
00083       virtual void ForAllCells(CCellOperation& c_operation);
00084 
00085       virtual void ForCellsInSphereRange(const CVector3& c_center,
00086                                          Real f_radius,
00087                                          CCellOperation& c_operation);
00088 
00089       virtual void ForCellsInBoxRange(const CVector3& c_center,
00090                                       const CVector3& c_half_size,
00091                                       CCellOperation& c_operation);
00092 
00093       virtual void ForCellsInCircleRange(const CVector3& c_center,
00094                                          Real f_radius,
00095                                          CCellOperation& c_operation);
00096 
00097       virtual void ForCellsInRectangleRange(const CVector3& c_center,
00098                                             const CVector2& c_half_size,
00099                                             CCellOperation& c_operation);
00100 
00101       virtual void ForCellsAlongRay(const CRay3& c_ray,
00102                                     CCellOperation& c_operation);
00103 
00104       inline SInt32 GetSizeI() const {
00105          return m_nSizeI;
00106       }
00107 
00108       inline SInt32 GetSizeJ() const {
00109          return m_nSizeJ;
00110       }
00111 
00112       inline SInt32 GetSizeK() const {
00113          return m_nSizeK;
00114       }
00115 
00116       inline void SetUpdateEntityOperation(CEntityOperation* pc_operation);
00117 
00118       void UpdateCell(SInt32 n_i,
00119                       SInt32 n_j,
00120                       SInt32 n_k,
00121                       ENTITY& c_entity);
00122 
00123       inline void PositionToCell(SInt32& n_i,
00124                                  SInt32& n_j,
00125                                  SInt32& n_k,
00126                                  const CVector3& c_position) const;
00127 
00128       inline void PositionToCellUnsafe(SInt32& n_i,
00129                                        SInt32& n_j,
00130                                        SInt32& n_k,
00131                                        const CVector3& c_position) const;
00132 
00133       inline void ClampCoordinates(SInt32& n_i,
00134                                    SInt32& n_j,
00135                                    SInt32& n_k) const;
00136 
00137       inline void ClampCoordinates(CVector3& c_pos) const;
00138 
00139       inline SCell& GetCellAt(SInt32 n_i,
00140                               SInt32 n_j,
00141                               SInt32 n_k);
00142 
00143       inline const SCell& GetCellAt(SInt32 n_i,
00144                                     SInt32 n_j,
00145                                     SInt32 n_k) const;
00146 
00147    protected:
00148 
00149       CVector3 m_cAreaMinCorner;
00150       CVector3 m_cAreaMaxCorner;
00151       SInt32 m_nSizeI;
00152       SInt32 m_nSizeJ;
00153       SInt32 m_nSizeK;
00154       CRange<Real> m_cRangeX;
00155       CRange<Real> m_cRangeY;
00156       CRange<Real> m_cRangeZ;
00157       CVector3 m_cCellSize;
00158       CVector3 m_cInvCellSize;
00159       SCell* m_psCells;
00160       size_t m_unCurTimestamp;
00161       CSet<ENTITY*> m_cEntities;
00162       CEntityOperation* m_pcUpdateEntityOperation;
00163 
00164    };
00165 
00166 }
00167 
00168 #include <argos3/core/simulator/space/positional_indices/grid_impl.h>
00169 
00170 #endif
```

---

Generated on 10 Jul 2018 for ARGoS by 
 1.6.1 
