# Supplementary material for: Iterative improvement in the automatic modular design of robot swarms
Source: PeerJ Comput Sci. 2020 Dec 7;6:e322. doi: 10.7717/peerj-cs.322 (PMC7924708; doi:10.7717/peerj-cs.322)
Supplement: Supplemental Information 3 [file peerj-cs-06-322-s003.zip › argos3/doc/api/standalone/a00333.html]

ARGoS: core/simulator/space/positional\_indices/grid\_impl.h File Reference


- Main Page
- Related Pages
- Namespaces
- Classes
- Files

- File List
- File Members

# core/simulator/space/positional\_indices/grid\_impl.h File Reference

Go to the source code of this file.

|  |  |
| --- | --- |
| Namespaces | |
| namespace | argos |

|  |  |
| --- | --- |
|  | The namespace containing all the ARGoS related code. |

| Defines | |
| #define | APPLY\_ENTITY\_OPERATION\_TO\_CELL(nI, nJ, nK) |
| #define | APPLY\_ENTITY\_OPERATION\_TO\_CELL\_ALONG\_RAY(nI, nJ, nK) |
| #define | APPLY\_CELL\_OPERATION\_TO\_CELL(nI, nJ, nK) |

---

## Define Documentation

|  |  |  |
| --- | --- | --- |
| #define APPLY\_CELL\_OPERATION\_TO\_CELL | ( | nI, |
|  |  | nJ, |
|  |  | nK |  | ) |  |

**Value:**

```
{                                                    \
      SCell& sCell = GetCellAt((nI), (nJ), (nK));       \
      if(!c_operation((nI), (nJ), (nK), sCell)) return; \
   }
```

Definition at line 38 of file grid\_impl.h.

|  |  |  |
| --- | --- | --- |
| #define APPLY\_ENTITY\_OPERATION\_TO\_CELL | ( | nI, |
|  |  | nJ, |
|  |  | nK |  | ) |  |

**Value:**

```
{                                                                    \
      SCell& sCell = GetCellAt((nI), (nJ), (nK));                       \
      if((sCell.Timestamp == m_unCurTimestamp) &&                       \
         (! sCell.Entities.empty())) {                                  \
         for(typename CSet<ENTITY*>::iterator it = sCell.Entities.begin(); \
             it != sCell.Entities.end();                                \
             ++it) {                                                    \
            if(!c_operation(**it)) return;                              \
         }                                                              \
      }                                                                 \
   }
```

Definition at line 11 of file grid\_impl.h.

|  |  |  |
| --- | --- | --- |
| #define APPLY\_ENTITY\_OPERATION\_TO\_CELL\_ALONG\_RAY | ( | nI, |
|  |  | nJ, |
|  |  | nK |  | ) |  |

**Value:**

```
{                                                                    \
      SCell& sCell = GetCellAt(nI, nJ, nK);                             \
      if((sCell.Timestamp == m_unCurTimestamp) &&                       \
         (! sCell.Entities.empty())) {                                  \
         for(typename CSet<ENTITY*>::iterator it = sCell.Entities.begin(); \
             it != sCell.Entities.end();                                \
             ++it) {                                                    \
            if(!c_operation(**it)) return;                              \
         }                                                              \
         if(b_stop_at_closest_match) return;                            \
      }                                                                 \
   }
```

Definition at line 24 of file grid\_impl.h.

---

Generated on 10 Jul 2018 for ARGoS by 
 1.6.1 
