# Supplementary material for: Iterative improvement in the automatic modular design of robot swarms
Source: PeerJ Comput Sci. 2020 Dec 7;6:e322. doi: 10.7717/peerj-cs.322 (PMC7924708; doi:10.7717/peerj-cs.322)
Supplement: Supplemental Information 3 [file peerj-cs-06-322-s003.zip › argos3/doc/api/standalone/a00333_source.html]

ARGoS: core/simulator/space/positional\_indices/grid\_impl.h Source File


- Main Page
- Related Pages
- Namespaces
- Classes
- Files

- File List
- File Members

# core/simulator/space/positional\_indices/grid\_impl.h

Go to the documentation of this file.

```
00001 namespace argos {
00002 
00003    /****************************************/
00004    /****************************************/
00005 
00006    static const Real EPSILON = 1e-6;
00007 
00008    /****************************************/
00009    /****************************************/
00010 
00011 #define APPLY_ENTITY_OPERATION_TO_CELL(nI,nJ,nK)                        \
00012    {                                                                    \
00013       SCell& sCell = GetCellAt((nI), (nJ), (nK));                       \
00014       if((sCell.Timestamp == m_unCurTimestamp) &&                       \
00015          (! sCell.Entities.empty())) {                                  \
00016          for(typename CSet<ENTITY*>::iterator it = sCell.Entities.begin(); \
00017              it != sCell.Entities.end();                                \
00018              ++it) {                                                    \
00019             if(!c_operation(**it)) return;                              \
00020          }                                                              \
00021       }                                                                 \
00022    }
00023 
00024 #define APPLY_ENTITY_OPERATION_TO_CELL_ALONG_RAY(nI,nJ,nK)              \
00025    {                                                                    \
00026       SCell& sCell = GetCellAt(nI, nJ, nK);                             \
00027       if((sCell.Timestamp == m_unCurTimestamp) &&                       \
00028          (! sCell.Entities.empty())) {                                  \
00029          for(typename CSet<ENTITY*>::iterator it = sCell.Entities.begin(); \
00030              it != sCell.Entities.end();                                \
00031              ++it) {                                                    \
00032             if(!c_operation(**it)) return;                              \
00033          }                                                              \
00034          if(b_stop_at_closest_match) return;                            \
00035       }                                                                 \
00036    }
00037 
00038 #define APPLY_CELL_OPERATION_TO_CELL(nI,nJ,nK)          \
00039    {                                                    \
00040       SCell& sCell = GetCellAt((nI), (nJ), (nK));       \
00041       if(!c_operation((nI), (nJ), (nK), sCell)) return; \
00042    }
00043 
00044 /****************************************/
00045 /****************************************/
00046 
00047 template<class ENTITY>
00048 CGrid<ENTITY>::CGrid(const CVector3& c_area_min_corner,
00049                      const CVector3& c_area_max_corner,
00050                      SInt32 n_size_i,
00051                      SInt32 n_size_j,
00052                      SInt32 n_size_k) :
00053    m_cAreaMinCorner(c_area_min_corner),
00054    m_cAreaMaxCorner(c_area_max_corner),
00055    m_nSizeI(n_size_i),
00056    m_nSizeJ(n_size_j),
00057    m_nSizeK(n_size_k),
00058    m_cRangeX(m_cAreaMinCorner.GetX(), m_cAreaMaxCorner.GetX()),
00059    m_cRangeY(m_cAreaMinCorner.GetY(), m_cAreaMaxCorner.GetY()),
00060    m_cRangeZ(m_cAreaMinCorner.GetZ(), m_cAreaMaxCorner.GetZ()),
00061    m_unCurTimestamp(0),
00062    m_pcUpdateEntityOperation(NULL) {
00063    m_cCellSize.Set(m_cRangeX.GetSpan() / m_nSizeI,
00064                    m_cRangeY.GetSpan() / m_nSizeJ,
00065                    m_cRangeZ.GetSpan() / m_nSizeK);
00066    m_cInvCellSize.Set(1.0f / m_cCellSize.GetX(),
00067                       1.0f / m_cCellSize.GetY(),
00068                       1.0f / m_cCellSize.GetZ());
00069    m_psCells = new SCell[m_nSizeI * m_nSizeJ * m_nSizeK];
00070 }
00071 
00072    /****************************************/
00073    /****************************************/
00074 
00075    template<class ENTITY>
00076    CGrid<ENTITY>::~CGrid() {
00077       delete[] m_psCells;
00078    }
00079 
00080    /****************************************/
00081    /****************************************/
00082 
00083    template<class ENTITY>
00084    void CGrid<ENTITY>::Init(TConfigurationNode& t_tree) {
00085    }
00086 
00087    /****************************************/
00088    /****************************************/
00089 
00090    template<class ENTITY>
00091    void CGrid<ENTITY>::Reset() {
00092       m_unCurTimestamp = 0;
00093       for(SInt32 i = 0; i < m_nSizeI; ++i) {
00094          for(SInt32 j = 0; j < m_nSizeJ; ++j) {
00095             for(SInt32 k = 0; k < m_nSizeK; ++k) {
00096                GetCellAt(i,j,k).Reset();
00097             }
00098          }
00099       }
00100       Update();
00101    }
00102 
00103    /****************************************/
00104    /****************************************/
00105 
00106    template<class ENTITY>
00107    void CGrid<ENTITY>::Destroy() {
00108    }
00109 
00110    /****************************************/
00111    /****************************************/
00112 
00113    template<class ENTITY>
00114    void CGrid<ENTITY>::AddEntity(ENTITY& c_entity) {
00115       m_cEntities.insert(&c_entity);
00116    }
00117 
00118    /****************************************/
00119    /****************************************/
00120 
00121    template<class ENTITY>
00122    void CGrid<ENTITY>::RemoveEntity(ENTITY& c_entity) {
00123       m_cEntities.erase(&c_entity);
00124    }
00125 
00126    /****************************************/
00127    /****************************************/
00128 
00129    template<class ENTITY>
00130    void CGrid<ENTITY>::Update() {
00131       ++m_unCurTimestamp;
00132       ForAllEntities(*m_pcUpdateEntityOperation);
00133    }
00134 
00135    /****************************************/
00136    /****************************************/
00137 
00138    template<class ENTITY>
00139    void CGrid<ENTITY>::GetEntitiesAt(CSet<ENTITY*>& c_entities,
00140                                      const CVector3& c_position) const {
00141       try {
00142          SInt32 i, j, k;
00143          PositionToCell(i, j, k, c_position);
00144          const SCell& sCell = GetCellAt(i, j, k);
00145          if(sCell.Timestamp < m_unCurTimestamp) {
00146             c_entities.clear();
00147          }
00148          else {
00149             c_entities = sCell.Entities;
00150          }
00151       }
00152       catch(CARGoSException& ex) {
00153          THROW_ARGOSEXCEPTION_NESTED("CGrid<ENTITY>::GetEntitiesAt() : Position <" << c_position << "> out of bounds X -> " << m_cRangeX << " Y -> " << m_cRangeY << " Z -> " << m_cRangeZ, ex);
00154       }
00155    }
00156 
00157    /****************************************/
00158    /****************************************/
00159 
00160    template<class ENTITY>
00161    void CGrid<ENTITY>::ForAllEntities(CEntityOperation& c_operation) {
00162       for(typename CSet<ENTITY*>::iterator it = m_cEntities.begin();
00163           it != m_cEntities.end() && c_operation(**it);
00164           ++it);
00165    }
00166 
00167    /****************************************/
00168    /****************************************/
00169 
00170    template<class ENTITY>
00171    void CGrid<ENTITY>::ForEntitiesInSphereRange(const CVector3& c_center,
00172                                                 Real f_radius,
00173                                                 CEntityOperation& c_operation) {
00174       /* Calculate cells for center */
00175       SInt32 nIC, nJC, nKC, nIR, nJR, nKR;
00176       PositionToCellUnsafe(nIC, nJC, nKC, c_center);
00177       if(nKC >= 0 && nKC < m_nSizeK) {
00178          /* Check circle center */
00179          if((nIC >= 0 && nIC < m_nSizeI) && (nJC >= 0 && nJC < m_nSizeJ)) APPLY_ENTITY_OPERATION_TO_CELL(nIC, nJC, nKC);
00180          /* Calculate radia of circle */
00181          nIR = Floor(f_radius * m_cInvCellSize.GetX() + 0.5f);
00182          nJR = Floor(f_radius * m_cInvCellSize.GetY() + 0.5f);
00183          /* Go through diameter on j at i = 0 */
00184          if(nIC >= 0 && nIC < m_nSizeI) {
00185             for(SInt32 j = nJR; j > 0; --j) {
00186                if(nJC + j >= 0 && nJC + j < m_nSizeJ) APPLY_ENTITY_OPERATION_TO_CELL(nIC, nJC + j, nKC);
00187                if(nJC - j >= 0 && nJC - j < m_nSizeJ) APPLY_ENTITY_OPERATION_TO_CELL(nIC, nJC - j, nKC);
00188             }
00189          }
00190          /* Go through diameter on i at j = 0 */
00191          if(nJC >= 0 && nJC < m_nSizeJ) {
00192             for(SInt32 i = nIR; i > 0; --i) {
00193                if(nIC + i >= 0 && nIC + i < m_nSizeI) APPLY_ENTITY_OPERATION_TO_CELL(nIC + i, nJC, nKC);
00194                if(nIC - i >= 0 && nIC - i < m_nSizeI) APPLY_ENTITY_OPERATION_TO_CELL(nIC - i, nJC, nKC);
00195             }
00196          }
00197          /* Go through cells with k = nKC */
00198          for(SInt32 j = nJR; j > 0; --j) {
00199             nIR = Floor(Sqrt(Max<Real>(0.0f, f_radius * f_radius - j * m_cCellSize.GetY() * j * m_cCellSize.GetY())) * m_cInvCellSize.GetX() + 0.5f);
00200             for(SInt32 i = nIR; i > 0; --i) {
00201                if(nIC + i >= 0 && nIC + i < m_nSizeI && nJC + j >= 0 && nJC + j < m_nSizeJ) APPLY_ENTITY_OPERATION_TO_CELL(nIC + i, nJC + j, nKC);
00202                if(nIC + i >= 0 && nIC + i < m_nSizeI && nJC - j >= 0 && nJC - j < m_nSizeJ) APPLY_ENTITY_OPERATION_TO_CELL(nIC + i, nJC - j, nKC);
00203                if(nIC - i >= 0 && nIC - i < m_nSizeI && nJC + j >= 0 && nJC + j < m_nSizeJ) APPLY_ENTITY_OPERATION_TO_CELL(nIC - i, nJC + j, nKC);
00204                if(nIC - i >= 0 && nIC - i < m_nSizeI && nJC - j >= 0 && nJC - j < m_nSizeJ) APPLY_ENTITY_OPERATION_TO_CELL(nIC - i, nJC - j, nKC);
00205             }
00206          }
00207       }
00208       /* Go through other cells */
00209       nKR = Floor(f_radius * m_cInvCellSize.GetZ() + 0.5f);
00210       Real fCircleRadius2;
00211       for(SInt32 k = nKR; k > 0; --k) {
00212          /* Check center of circle at k and -k */
00213          if((nIC >= 0 && nIC < m_nSizeI) && (nJC >= 0 && nJC < m_nSizeJ)) {
00214             if(nKC + k >= 0 && nKC + k < m_nSizeK) APPLY_ENTITY_OPERATION_TO_CELL(nIC, nJC, nKC + k);
00215             if(nKC - k >= 0 && nKC - k < m_nSizeK) APPLY_ENTITY_OPERATION_TO_CELL(nIC, nJC, nKC - k);
00216          }
00217          /* Calculate radius of circle at k and -k */
00218          fCircleRadius2 = Max<Real>(0.0f, f_radius * f_radius - k * m_cCellSize.GetZ() * k * m_cCellSize.GetZ());
00219          /* Calculate radius of circle at j,k */
00220          nIR = Floor(Sqrt(fCircleRadius2) * m_cInvCellSize.GetX() + 0.5f);
00221          /* Go through diameter on i at j = 0 */
00222          if(nJC >= 0 && nJC < m_nSizeJ) {
00223             for(SInt32 i = nIR; i > 0; --i) {
00224                if(nIC + i >= 0 && nIC + i < m_nSizeI && nKC + k >= 0 && nKC + k < m_nSizeK) APPLY_ENTITY_OPERATION_TO_CELL(nIC + i, nJC, nKC + k);
00225                if(nIC + i >= 0 && nIC + i < m_nSizeI && nKC - k >= 0 && nKC - k < m_nSizeK) APPLY_ENTITY_OPERATION_TO_CELL(nIC + i, nJC, nKC - k);
00226                if(nIC - i >= 0 && nIC - i < m_nSizeI && nKC + k >= 0 && nKC + k < m_nSizeK) APPLY_ENTITY_OPERATION_TO_CELL(nIC - i, nJC, nKC + k);
00227                if(nIC - i >= 0 && nIC - i < m_nSizeI && nKC - k >= 0 && nKC - k < m_nSizeK) APPLY_ENTITY_OPERATION_TO_CELL(nIC - i, nJC, nKC - k);
00228             }
00229          }
00230          /* Calculate circle radius in cells on j */
00231          nJR = Floor(Sqrt(fCircleRadius2) * m_cInvCellSize.GetY() + 0.5f);
00232          for(SInt32 j = nJR; j > 0; --j) {
00233             /* Go through diameter on j at i = 0 */
00234             if(nIC >= 0 && nIC < m_nSizeI) {
00235                if(nJC + j >= 0 && nJC + j < m_nSizeJ && nKC + k >= 0 && nKC + k < m_nSizeK) APPLY_ENTITY_OPERATION_TO_CELL(nIC, nJC + j, nKC + k);
00236                if(nJC + j >= 0 && nJC + j < m_nSizeJ && nKC - k >= 0 && nKC - k < m_nSizeK) APPLY_ENTITY_OPERATION_TO_CELL(nIC, nJC + j, nKC - k);
00237                if(nJC - j >= 0 && nJC - j < m_nSizeJ && nKC + k >= 0 && nKC + k < m_nSizeK) APPLY_ENTITY_OPERATION_TO_CELL(nIC, nJC - j, nKC + k);
00238                if(nJC - j >= 0 && nJC - j < m_nSizeJ && nKC - k >= 0 && nKC - k < m_nSizeK) APPLY_ENTITY_OPERATION_TO_CELL(nIC, nJC - j, nKC - k);
00239             }
00240             /* Calculate radius of circle at j,k */
00241             nIR = Floor(Sqrt(Max<Real>(0.0f, fCircleRadius2 - j * m_cCellSize.GetY() * j * m_cCellSize.GetY())) * m_cInvCellSize.GetX() + 0.5f);
00242             for(SInt32 i = nIR; i > 0; --i) {
00243                if(nIC + i >= 0 && nIC + i < m_nSizeI && nJC + j >= 0 && nJC + j < m_nSizeJ && nKC + k >= 0 && nKC + k < m_nSizeK) APPLY_ENTITY_OPERATION_TO_CELL(nIC + i, nJC + j, nKC + k);
00244                if(nIC + i >= 0 && nIC + i < m_nSizeI && nJC + j >= 0 && nJC + j < m_nSizeJ && nKC - k >= 0 && nKC - k < m_nSizeK) APPLY_ENTITY_OPERATION_TO_CELL(nIC + i, nJC + j, nKC - k);
00245                if(nIC + i >= 0 && nIC + i < m_nSizeI && nJC - j >= 0 && nJC - j < m_nSizeJ && nKC + k >= 0 && nKC + k < m_nSizeK) APPLY_ENTITY_OPERATION_TO_CELL(nIC + i, nJC - j, nKC + k);
00246                if(nIC + i >= 0 && nIC + i < m_nSizeI && nJC - j >= 0 && nJC - j < m_nSizeJ && nKC - k >= 0 && nKC - k < m_nSizeK) APPLY_ENTITY_OPERATION_TO_CELL(nIC + i, nJC - j, nKC - k);
00247                if(nIC - i >= 0 && nIC - i < m_nSizeI && nJC + j >= 0 && nJC + j < m_nSizeJ && nKC + k >= 0 && nKC + k < m_nSizeK) APPLY_ENTITY_OPERATION_TO_CELL(nIC - i, nJC + j, nKC + k);
00248                if(nIC - i >= 0 && nIC - i < m_nSizeI && nJC + j >= 0 && nJC + j < m_nSizeJ && nKC - k >= 0 && nKC - k < m_nSizeK) APPLY_ENTITY_OPERATION_TO_CELL(nIC - i, nJC + j, nKC - k);
00249                if(nIC - i >= 0 && nIC - i < m_nSizeI && nJC - j >= 0 && nJC - j < m_nSizeJ && nKC + k >= 0 && nKC + k < m_nSizeK) APPLY_ENTITY_OPERATION_TO_CELL(nIC - i, nJC - j, nKC + k);
00250                if(nIC - i >= 0 && nIC - i < m_nSizeI && nJC - j >= 0 && nJC - j < m_nSizeJ && nKC - k >= 0 && nKC - k < m_nSizeK) APPLY_ENTITY_OPERATION_TO_CELL(nIC - i, nJC - j, nKC - k);
00251             }
00252          }
00253       }
00254    }
00255 
00256    /****************************************/
00257    /****************************************/
00258 
00259    template<class ENTITY>
00260    void CGrid<ENTITY>::ForEntitiesInBoxRange(const CVector3& c_center,
00261                                              const CVector3& c_half_size,
00262                                              CEntityOperation& c_operation) {
00263       /* Calculate cell range */
00264       SInt32 nI1, nJ1, nK1, nI2, nJ2, nK2;
00265       PositionToCellUnsafe(nI1, nJ1, nK1, c_center - c_half_size);
00266       ClampCoordinates(nI1, nJ1, nK1);
00267       PositionToCellUnsafe(nI2, nJ2, nK2, c_center + c_half_size);
00268       ClampCoordinates(nI2, nJ2, nK2);
00269       /* Go through cells */
00270       for(SInt32 k = nK1; k <= nK2; ++k) {
00271          for(SInt32 j = nJ1; j <= nJ2; ++j) {
00272             for(SInt32 i = nI1; i <= nI2; ++i) {
00273                APPLY_ENTITY_OPERATION_TO_CELL(i, j, k);
00274             }
00275          }
00276       }
00277    }
00278 
00279    /****************************************/
00280    /****************************************/
00281 
00282    template<class ENTITY>
00283    void CGrid<ENTITY>::ForEntitiesInCircleRange(const CVector3& c_center,
00284                                                 Real f_radius,
00285                                                 CEntityOperation& c_operation) {
00286       /* Make sure the Z coordinate is inside the range */
00287       if(! m_cRangeZ.WithinMinBoundIncludedMaxBoundIncluded(c_center.GetZ())) return;
00288       /* Calculate cells for center */
00289       SInt32 nI, nJ, nK;
00290       PositionToCellUnsafe(nI, nJ, nK, c_center);
00291       /* Check circle center */
00292       if((nI >= 0 && nI < m_nSizeI) && (nJ >= 0 && nJ < m_nSizeJ)) APPLY_ENTITY_OPERATION_TO_CELL(nI, nJ, nK);
00293       /* Check circle diameter on I */
00294       SInt32 nID = Floor(f_radius * m_cInvCellSize.GetX() + 0.5f);
00295       for(SInt32 h = nID; h > 0; --h) {
00296          if((nI + h >= 0 && nI + h < m_nSizeI) && (nJ >= 0 && nJ < m_nSizeJ)) APPLY_ENTITY_OPERATION_TO_CELL(nI + h, nJ, nK);
00297          if((nI - h >= 0 && nI - h < m_nSizeI) && (nJ >= 0 && nJ < m_nSizeJ)) APPLY_ENTITY_OPERATION_TO_CELL(nI - h, nJ, nK);
00298       }
00299       /* Check circle diameter on J */
00300       SInt32 nJD = Floor(f_radius * m_cInvCellSize.GetY() + 0.5f);
00301       for(SInt32 h = nJD; h > 0; --h) {
00302          if((nI >= 0 && nI < m_nSizeI) && (nJ + h >= 0 && nJ + h < m_nSizeJ)) APPLY_ENTITY_OPERATION_TO_CELL(nI, nJ + h, nK);
00303          if((nI >= 0 && nI < m_nSizeI) && (nJ - h >= 0 && nJ - h < m_nSizeJ)) APPLY_ENTITY_OPERATION_TO_CELL(nI, nJ - h, nK);
00304       }
00305       /* Check rest of the circle */
00306       for(SInt32 i = nID; i > 0; --i) {
00307          nJD = Floor(Sqrt(f_radius * f_radius - i * m_cCellSize.GetX() * i * m_cCellSize.GetX()) * m_cInvCellSize.GetY() + 0.5f);
00308          for(SInt32 j = nJD; j > 0; --j) {
00309             if((nI + i >= 0 && nI + i < m_nSizeI) && (nJ + j >= 0 && nJ + j < m_nSizeJ)) APPLY_ENTITY_OPERATION_TO_CELL(nI + i, nJ + j, nK);
00310             if((nI + i >= 0 && nI + i < m_nSizeI) && (nJ - j >= 0 && nJ - j < m_nSizeJ)) APPLY_ENTITY_OPERATION_TO_CELL(nI + i, nJ - j, nK);
00311             if((nI - i >= 0 && nI - i < m_nSizeI) && (nJ + j >= 0 && nJ + j < m_nSizeJ)) APPLY_ENTITY_OPERATION_TO_CELL(nI - i, nJ + j, nK);
00312             if((nI - i >= 0 && nI - i < m_nSizeI) && (nJ - j >= 0 && nJ - j < m_nSizeJ)) APPLY_ENTITY_OPERATION_TO_CELL(nI - i, nJ - j, nK);
00313          }
00314       }
00315    }
00316 
00317    /****************************************/
00318    /****************************************/
00319 
00320    template<class ENTITY>
00321    void CGrid<ENTITY>::ForEntitiesInRectangleRange(const CVector3& c_center,
00322                                                    const CVector2& c_half_size,
00323                                                    CEntityOperation& c_operation) {
00324       /* Calculate cell range */
00325       SInt32 nI1 = Min<SInt32>(m_nSizeI-1, Max<SInt32>(0, Floor((c_center.GetX() - c_half_size.GetX() - m_cAreaMinCorner.GetX()) * m_cInvCellSize.GetX())));
00326       SInt32 nJ1 = Min<SInt32>(m_nSizeJ-1, Max<SInt32>(0, Floor((c_center.GetY() - c_half_size.GetY() - m_cAreaMinCorner.GetY()) * m_cInvCellSize.GetY())));
00327       SInt32 nI2 = Min<SInt32>(m_nSizeI-1, Max<SInt32>(0, Floor((c_center.GetX() + c_half_size.GetX() - m_cAreaMinCorner.GetX()) * m_cInvCellSize.GetX())));
00328       SInt32 nJ2 = Min<SInt32>(m_nSizeJ-1, Max<SInt32>(0, Floor((c_center.GetY() + c_half_size.GetY() - m_cAreaMinCorner.GetY()) * m_cInvCellSize.GetY())));
00329       SInt32 nK  = Min<SInt32>(m_nSizeK-1, Max<SInt32>(0, Floor((c_center.GetZ()                      - m_cAreaMinCorner.GetZ()) * m_cInvCellSize.GetZ())));
00330       /* Go through cells */
00331       for(SInt32 j = nJ1; j <= nJ2; ++j) {
00332          for(SInt32 i = nI1; i <= nI2; ++i) {
00333             APPLY_ENTITY_OPERATION_TO_CELL(i, j, nK);
00334          }
00335       }
00336    }
00337 
00338    /****************************************/
00339    /****************************************/
00340 
00341    template<class ENTITY>
00342    void CGrid<ENTITY>::ForEntitiesAlongRay(const CRay3& c_ray,
00343                                            CEntityOperation& c_operation,
00344                                            bool b_stop_at_closest_match) {
00345       /* Transform ray start and end position into cell coordinates */
00346       SInt32 nI1, nJ1, nK1, nI2, nJ2, nK2;
00347       PositionToCellUnsafe(nI1, nJ1, nK1, c_ray.GetStart());
00348       ClampCoordinates(nI1, nJ1, nK1);
00349       PositionToCellUnsafe(nI2, nJ2, nK2, c_ray.GetEnd());
00350       ClampCoordinates(nI2, nJ2, nK2);
00351       /* Go through cells one by one, from start to end.
00352          Stop as soon as an entity is found.
00353          If the loop is completed, it means no entities were found -> no intersection.
00354       */
00355       /* Calculate deltas for later use */
00356       SInt32 nDI(Abs(nI2 - nI1));
00357       SInt32 nDJ(Abs(nJ2 - nJ1));
00358       SInt32 nDK(Abs(nK2 - nK1));
00359       /* Calculate the increment for each direction */
00360       SInt32 nSI(nI2 >= nI1 ? 1 : -1);
00361       SInt32 nSJ(nJ2 >= nJ1 ? 1 : -1);
00362       SInt32 nSK(nK2 >= nK1 ? 1 : -1);
00363       /* Set the starting cell */
00364       SInt32 nI(nI1), nJ(nJ1), nK(nK1);
00365       if(nDI >= nDJ && nDI >= nDK) {
00366          /* I is the driving axis */
00367          /* Calculate error used to know when to move on other axes */
00368          SInt32 nEJ(3 * nDJ - nDI);
00369          SInt32 nEK(3 * nDK - nDI);
00370          APPLY_ENTITY_OPERATION_TO_CELL_ALONG_RAY(nI, nJ, nK);
00371          /* Cycle through cells */
00372          for(SInt32 nCell = nDI; nCell > 0; --nCell) {
00373             /* Advance on driving axis */
00374             nI += nSI;
00375             APPLY_ENTITY_OPERATION_TO_CELL_ALONG_RAY(nI, nJ, nK);
00376             /* Advance on other axes, if necessary */
00377             if(nEJ > 0 && nEK > 0) {
00378                /* Advance on both the other axes */
00379                if(nEJ * nDK > nEK * nDJ) {
00380                   nJ += nSJ;
00381                   APPLY_ENTITY_OPERATION_TO_CELL_ALONG_RAY(nI, nJ, nK);
00382                   nK += nSK;
00383                   APPLY_ENTITY_OPERATION_TO_CELL_ALONG_RAY(nI, nJ, nK);
00384                }
00385                else {
00386                   nK += nSK;
00387                   APPLY_ENTITY_OPERATION_TO_CELL_ALONG_RAY(nI, nJ, nK);
00388                   nJ += nSJ;
00389                   APPLY_ENTITY_OPERATION_TO_CELL_ALONG_RAY(nI, nJ, nK);
00390                }
00391                nEJ += 2 * (nDJ - nDI);
00392                nEK += 2 * (nDK - nDI);
00393             }
00394             else if(nEJ > 0) {
00395                /* Advance only on J */
00396                nJ += nSJ;
00397                APPLY_ENTITY_OPERATION_TO_CELL_ALONG_RAY(nI, nJ, nK);
00398                nEJ += 2 * (nDJ - nDI);
00399                nEK += 2 * nDK;
00400             }
00401             else {
00402                nEJ += 2 * nDJ;
00403                if(nEK > 0) {
00404                   /* Advance only on K */
00405                   nK += nSK;
00406                   APPLY_ENTITY_OPERATION_TO_CELL_ALONG_RAY(nI, nJ, nK);
00407                   nEK += 2 * (nDK - nDI);
00408                }
00409                else {
00410                   nEK += 2 * nDK;
00411                }
00412             }
00413          }
00414       }
00415       else if(nDJ >= nDI && nDJ >= nDK) {
00416          /* J is the driving axis */
00417          /* Calculate error used to know when to move on other axes */
00418          SInt32 nEI(3 * nDI - nDJ);
00419          SInt32 nEK(3 * nDK - nDJ);
00420          APPLY_ENTITY_OPERATION_TO_CELL_ALONG_RAY(nI, nJ, nK);
00421          /* Cycle through cells */
00422          for(SInt32 nCell = nDJ; nCell > 0; --nCell) {
00423             /* Advance on driving axis */
00424             nJ += nSJ;
00425             APPLY_ENTITY_OPERATION_TO_CELL_ALONG_RAY(nI, nJ, nK);
00426             /* Advance on other axes, if necessary */
00427             if(nEI > 0 && nEK > 0) {
00428                /* Advance on both the other axes */
00429                if(nEI * nDK > nEK * nDI) {
00430                   nI += nSI;
00431                   APPLY_ENTITY_OPERATION_TO_CELL_ALONG_RAY(nI, nJ, nK);
00432                   nK += nSK;
00433                   APPLY_ENTITY_OPERATION_TO_CELL_ALONG_RAY(nI, nJ, nK);
00434                }
00435                else {
00436                   nK += nSK;
00437                   APPLY_ENTITY_OPERATION_TO_CELL_ALONG_RAY(nI, nJ, nK);
00438                   nI += nSI;
00439                   APPLY_ENTITY_OPERATION_TO_CELL_ALONG_RAY(nI, nJ, nK);
00440                }
00441                nEI += 2 * (nDI - nDJ);
00442                nEK += 2 * (nDK - nDJ);
00443             }
00444             else if(nEI > 0) {
00445                /* Advance only on I */
00446                nI += nSI;
00447                APPLY_ENTITY_OPERATION_TO_CELL_ALONG_RAY(nI, nJ, nK);
00448                nEI += 2 * (nDI - nDJ);
00449                nEK += 2 * nDK;
00450             }
00451             else {
00452                nEI += 2 * nDI;
00453                if(nEK > 0) {
00454                   /* Advance only on K */
00455                   nK += nSK;
00456                   APPLY_ENTITY_OPERATION_TO_CELL_ALONG_RAY(nI, nJ, nK);
00457                   nEK += 2 * (nDK - nDJ);
00458                }
00459                else {
00460                   nEK += 2 * nDK;
00461                }
00462             }
00463          }
00464       }
00465       else {
00466          /* K is the driving axis */
00467          /* Calculate error used to know when to move on other axes */
00468          SInt32 nEI(3 * nDI - nDK);
00469          SInt32 nEJ(3 * nDJ - nDK);
00470          APPLY_ENTITY_OPERATION_TO_CELL_ALONG_RAY(nI, nJ, nK);
00471          /* Cycle through cells */
00472          for(SInt32 nCell = nDK; nCell > 0; --nCell) {
00473             /* Advance on driving axis */
00474             nK += nSK;
00475             APPLY_ENTITY_OPERATION_TO_CELL_ALONG_RAY(nI, nJ, nK);
00476             /* Advance on other axes, if necessary */
00477             if(nEI > 0 && nEJ > 0) {
00478                /* Advance on both the other axes */
00479                if(nEI * nDJ > nEJ * nDI) {
00480                   nI += nSI;
00481                   APPLY_ENTITY_OPERATION_TO_CELL_ALONG_RAY(nI, nJ, nK);
00482                   nJ += nSJ;
00483                   APPLY_ENTITY_OPERATION_TO_CELL_ALONG_RAY(nI, nJ, nK);
00484                }
00485                else {
00486                   nJ += nSJ;
00487                   APPLY_ENTITY_OPERATION_TO_CELL_ALONG_RAY(nI, nJ, nK);
00488                   nI += nSI;
00489                   APPLY_ENTITY_OPERATION_TO_CELL_ALONG_RAY(nI, nJ, nK);
00490                }
00491                nEI += 2 * (nDI - nDK);
00492                nEJ += 2 * (nDJ - nDK);
00493             }
00494             else if(nEI > 0) {
00495                /* Advance only on I */
00496                nI += nSI;
00497                APPLY_ENTITY_OPERATION_TO_CELL_ALONG_RAY(nI, nJ, nK);
00498                nEI += 2 * (nDI - nDK);
00499                nEJ += 2 * nDJ;
00500             }
00501             else {
00502                nEI += 2 * nDI;
00503                if(nEJ > 0) {
00504                   /* Advance only on J */
00505                   nJ += nSJ;
00506                   APPLY_ENTITY_OPERATION_TO_CELL_ALONG_RAY(nI, nJ, nK);
00507                   nEJ += 2 * (nDJ - nDK);
00508                }
00509                else {
00510                   nEJ += 2 * nDJ;
00511                }
00512             }
00513          }
00514       }
00515    }
00516 
00517    /****************************************/
00518    /****************************************/
00519 
00520    template<class ENTITY>
00521    void CGrid<ENTITY>::ForAllCells(CCellOperation& c_operation) {
00522       for(SInt32 k = 0; k < m_nSizeK; ++k) {
00523          for(SInt32 j = 0; j < m_nSizeJ; ++j) {
00524             for(SInt32 i = 0; i < m_nSizeI; ++i) {
00525                APPLY_CELL_OPERATION_TO_CELL(i, j, k);
00526             }
00527          }
00528       }
00529    }
00530 
00531    /****************************************/
00532    /****************************************/
00533 
00534    template<class ENTITY>
00535    void CGrid<ENTITY>::ForCellsInSphereRange(const CVector3& c_center,
00536                                              Real f_radius,
00537                                              CCellOperation& c_operation) {
00538       /* Calculate cells for center */
00539       SInt32 nIC, nJC, nKC, nIR, nJR, nKR;
00540       PositionToCellUnsafe(nIC, nJC, nKC, c_center);
00541       if(nKC >= 0 && nKC < m_nSizeK) {
00542          /* Check circle center */
00543          if((nIC >= 0 && nIC < m_nSizeI) && (nJC >= 0 && nJC < m_nSizeJ)) APPLY_CELL_OPERATION_TO_CELL(nIC, nJC, nKC);
00544          /* Calculate radia of circle */
00545          nIR = Floor(f_radius * m_cInvCellSize.GetX() + 0.5f);
00546          nJR = Floor(f_radius * m_cInvCellSize.GetY() + 0.5f);
00547          /* Go through diameter on j at i = 0 */
00548          if(nIC >= 0 && nIC < m_nSizeI) {
00549             for(SInt32 j = nJR; j > 0; --j) {
00550                if(nJC + j >= 0 && nJC + j < m_nSizeJ) APPLY_CELL_OPERATION_TO_CELL(nIC, nJC + j, nKC);
00551                if(nJC - j >= 0 && nJC - j < m_nSizeJ) APPLY_CELL_OPERATION_TO_CELL(nIC, nJC - j, nKC);
00552             }
00553          }
00554          /* Go through diameter on i at j = 0 */
00555          if(nJC >= 0 && nJC < m_nSizeJ) {
00556             for(SInt32 i = nIR; i > 0; --i) {
00557                if(nIC + i >= 0 && nIC + i < m_nSizeI) APPLY_CELL_OPERATION_TO_CELL(nIC + i, nJC, nKC);
00558                if(nIC - i >= 0 && nIC - i < m_nSizeI) APPLY_CELL_OPERATION_TO_CELL(nIC - i, nJC, nKC);
00559             }
00560          }
00561          /* Go through cells with k = nKC */
00562          for(SInt32 j = nJR; j > 0; --j) {
00563             nIR = Floor(Sqrt(Max<Real>(0.0f, f_radius * f_radius - j * m_cCellSize.GetY() * j * m_cCellSize.GetY())) * m_cInvCellSize.GetX() + 0.5f);
00564             for(SInt32 i = nIR; i > 0; --i) {
00565                if(nIC + i >= 0 && nIC + i < m_nSizeI && nJC + j >= 0 && nJC + j < m_nSizeJ) APPLY_CELL_OPERATION_TO_CELL(nIC + i, nJC + j, nKC);
00566                if(nIC + i >= 0 && nIC + i < m_nSizeI && nJC - j >= 0 && nJC - j < m_nSizeJ) APPLY_CELL_OPERATION_TO_CELL(nIC + i, nJC - j, nKC);
00567                if(nIC - i >= 0 && nIC - i < m_nSizeI && nJC + j >= 0 && nJC + j < m_nSizeJ) APPLY_CELL_OPERATION_TO_CELL(nIC - i, nJC + j, nKC);
00568                if(nIC - i >= 0 && nIC - i < m_nSizeI && nJC - j >= 0 && nJC - j < m_nSizeJ) APPLY_CELL_OPERATION_TO_CELL(nIC - i, nJC - j, nKC);
00569             }
00570          }
00571       }
00572       /* Go through other cells */
00573       nKR = Floor(f_radius * m_cInvCellSize.GetZ() + 0.5f);
00574       Real fCircleRadius2;
00575       for(SInt32 k = nKR; k > 0; --k) {
00576          /* Check center of circle at k and -k */
00577          if((nIC >= 0 && nIC < m_nSizeI) && (nJC >= 0 && nJC < m_nSizeJ)) {
00578             if(nKC + k >= 0 && nKC + k < m_nSizeK) APPLY_CELL_OPERATION_TO_CELL(nIC, nJC, nKC + k);
00579             if(nKC - k >= 0 && nKC - k < m_nSizeK) APPLY_CELL_OPERATION_TO_CELL(nIC, nJC, nKC - k);
00580          }
00581          /* Calculate radius of circle at k and -k */
00582          fCircleRadius2 = Max<Real>(0.0f, f_radius * f_radius - k * m_cCellSize.GetZ() * k * m_cCellSize.GetZ());
00583          /* Calculate radius of circle at j,k */
00584          nIR = Floor(Sqrt(fCircleRadius2) * m_cInvCellSize.GetX() + 0.5f);
00585          /* Go through diameter on i at j = 0 */
00586          if(nJC >= 0 && nJC < m_nSizeJ) {
00587             for(SInt32 i = nIR; i > 0; --i) {
00588                if(nIC + i >= 0 && nIC + i < m_nSizeI && nKC + k >= 0 && nKC + k < m_nSizeK) APPLY_CELL_OPERATION_TO_CELL(nIC + i, nJC, nKC + k);
00589                if(nIC + i >= 0 && nIC + i < m_nSizeI && nKC - k >= 0 && nKC - k < m_nSizeK) APPLY_CELL_OPERATION_TO_CELL(nIC + i, nJC, nKC - k);
00590                if(nIC - i >= 0 && nIC - i < m_nSizeI && nKC + k >= 0 && nKC + k < m_nSizeK) APPLY_CELL_OPERATION_TO_CELL(nIC - i, nJC, nKC + k);
00591                if(nIC - i >= 0 && nIC - i < m_nSizeI && nKC - k >= 0 && nKC - k < m_nSizeK) APPLY_CELL_OPERATION_TO_CELL(nIC - i, nJC, nKC - k);
00592             }
00593          }
00594          /* Calculate circle radius in cells on j */
00595          nJR = Floor(Sqrt(fCircleRadius2) * m_cInvCellSize.GetY() + 0.5f);
00596          for(SInt32 j = nJR; j > 0; --j) {
00597             /* Go through diameter on j at i = 0 */
00598             if(nIC >= 0 && nIC < m_nSizeI) {
00599                if(nJC + j >= 0 && nJC + j < m_nSizeJ && nKC + k >= 0 && nKC + k < m_nSizeK) APPLY_CELL_OPERATION_TO_CELL(nIC, nJC + j, nKC + k);
00600                if(nJC + j >= 0 && nJC + j < m_nSizeJ && nKC - k >= 0 && nKC - k < m_nSizeK) APPLY_CELL_OPERATION_TO_CELL(nIC, nJC + j, nKC - k);
00601                if(nJC - j >= 0 && nJC - j < m_nSizeJ && nKC + k >= 0 && nKC + k < m_nSizeK) APPLY_CELL_OPERATION_TO_CELL(nIC, nJC - j, nKC + k);
00602                if(nJC - j >= 0 && nJC - j < m_nSizeJ && nKC - k >= 0 && nKC - k < m_nSizeK) APPLY_CELL_OPERATION_TO_CELL(nIC, nJC - j, nKC - k);
00603             }
00604             /* Calculate radius of circle at j,k */
00605             nIR = Floor(Sqrt(Max<Real>(0.0f, fCircleRadius2 - j * m_cCellSize.GetY() * j * m_cCellSize.GetY())) * m_cInvCellSize.GetX() + 0.5f);
00606             for(SInt32 i = nIR; i > 0; --i) {
00607                if(nIC + i >= 0 && nIC + i < m_nSizeI && nJC + j >= 0 && nJC + j < m_nSizeJ && nKC + k >= 0 && nKC + k < m_nSizeK) APPLY_CELL_OPERATION_TO_CELL(nIC + i, nJC + j, nKC + k);
00608                if(nIC + i >= 0 && nIC + i < m_nSizeI && nJC + j >= 0 && nJC + j < m_nSizeJ && nKC - k >= 0 && nKC - k < m_nSizeK) APPLY_CELL_OPERATION_TO_CELL(nIC + i, nJC + j, nKC - k);
00609                if(nIC + i >= 0 && nIC + i < m_nSizeI && nJC - j >= 0 && nJC - j < m_nSizeJ && nKC + k >= 0 && nKC + k < m_nSizeK) APPLY_CELL_OPERATION_TO_CELL(nIC + i, nJC - j, nKC + k);
00610                if(nIC + i >= 0 && nIC + i < m_nSizeI && nJC - j >= 0 && nJC - j < m_nSizeJ && nKC - k >= 0 && nKC - k < m_nSizeK) APPLY_CELL_OPERATION_TO_CELL(nIC + i, nJC - j, nKC - k);
00611                if(nIC - i >= 0 && nIC - i < m_nSizeI && nJC + j >= 0 && nJC + j < m_nSizeJ && nKC + k >= 0 && nKC + k < m_nSizeK) APPLY_CELL_OPERATION_TO_CELL(nIC - i, nJC + j, nKC + k);
00612                if(nIC - i >= 0 && nIC - i < m_nSizeI && nJC + j >= 0 && nJC + j < m_nSizeJ && nKC - k >= 0 && nKC - k < m_nSizeK) APPLY_CELL_OPERATION_TO_CELL(nIC - i, nJC + j, nKC - k);
00613                if(nIC - i >= 0 && nIC - i < m_nSizeI && nJC - j >= 0 && nJC - j < m_nSizeJ && nKC + k >= 0 && nKC + k < m_nSizeK) APPLY_CELL_OPERATION_TO_CELL(nIC - i, nJC - j, nKC + k);
00614                if(nIC - i >= 0 && nIC - i < m_nSizeI && nJC - j >= 0 && nJC - j < m_nSizeJ && nKC - k >= 0 && nKC - k < m_nSizeK) APPLY_CELL_OPERATION_TO_CELL(nIC - i, nJC - j, nKC - k);
00615             }
00616          }
00617       }
00618    }
00619 
00620    /****************************************/
00621    /****************************************/
00622 
00623    template<class ENTITY>
00624    void CGrid<ENTITY>::ForCellsInBoxRange(const CVector3& c_center,
00625                                           const CVector3& c_half_size,
00626                                           CCellOperation& c_operation) {
00627       /* Calculate cell range */
00628       SInt32 nI1, nJ1, nK1, nI2, nJ2, nK2;
00629       PositionToCellUnsafe(nI1, nJ1, nK1, c_center - c_half_size);
00630       ClampCoordinates(nI1, nJ1, nK1);
00631       PositionToCellUnsafe(nI2, nJ2, nK2, c_center + c_half_size);
00632       ClampCoordinates(nI2, nJ2, nK2);
00633       /* Go through cells */
00634       for(SInt32 k = nK1; k <= nK2; ++k) {
00635          for(SInt32 j = nJ1; j <= nJ2; ++j) {
00636             for(SInt32 i = nI1; i <= nI2; ++i) {
00637                APPLY_CELL_OPERATION_TO_CELL(i, j, k);
00638             }
00639          }
00640       }
00641    }
00642 
00643    /****************************************/
00644    /****************************************/
00645 
00646    template<class ENTITY>
00647    void CGrid<ENTITY>::ForCellsInCircleRange(const CVector3& c_center,
00648                                              Real f_radius,
00649                                              CCellOperation& c_operation) {
00650       /* Make sure the Z coordinate is inside the range */
00651       if(! m_cRangeZ.WithinMinBoundIncludedMaxBoundIncluded(c_center.GetZ())) return;
00652       /* Calculate cells for center */
00653       SInt32 nI, nJ, nK;
00654       PositionToCellUnsafe(nI, nJ, nK, c_center);
00655       /* Check circle center */
00656       if((nI >= 0 && nI < m_nSizeI) && (nJ >= 0 && nJ < m_nSizeJ)) APPLY_CELL_OPERATION_TO_CELL(nI, nJ, nK);
00657       /* Check circle diameter on I */
00658       SInt32 nID = Floor(f_radius * m_cInvCellSize.GetX() + 0.5f);
00659       for(SInt32 h = nID; h > 0; --h) {
00660          if((nI + h >= 0 && nI + h < m_nSizeI) && (nJ >= 0 && nJ < m_nSizeJ)) APPLY_CELL_OPERATION_TO_CELL(nI + h, nJ, nK);
00661          if((nI - h >= 0 && nI - h < m_nSizeI) && (nJ >= 0 && nJ < m_nSizeJ)) APPLY_CELL_OPERATION_TO_CELL(nI - h, nJ, nK);
00662       }
00663       /* Check circle diameter on J */
00664       SInt32 nJD = Floor(f_radius * m_cInvCellSize.GetY() + 0.5f);
00665       for(SInt32 h = nJD; h > 0; --h) {
00666          if((nI >= 0 && nI < m_nSizeI) && (nJ + h >= 0 && nJ + h < m_nSizeJ)) APPLY_CELL_OPERATION_TO_CELL(nI, nJ + h, nK);
00667          if((nI >= 0 && nI < m_nSizeI) && (nJ - h >= 0 && nJ - h < m_nSizeJ)) APPLY_CELL_OPERATION_TO_CELL(nI, nJ - h, nK);
00668       }
00669       /* Check rest of the circle */
00670       for(SInt32 i = nID; i > 0; --i) {
00671          nJD = Floor(Sqrt(f_radius * f_radius - i * m_cCellSize.GetX() * i * m_cCellSize.GetX()) * m_cInvCellSize.GetY() + 0.5f);
00672          for(SInt32 j = nJD; j > 0; --j) {
00673             if((nI + i >= 0 && nI + i < m_nSizeI) && (nJ + j >= 0 && nJ + j < m_nSizeJ)) APPLY_CELL_OPERATION_TO_CELL(nI + i, nJ + j, nK);
00674             if((nI + i >= 0 && nI + i < m_nSizeI) && (nJ - j >= 0 && nJ - j < m_nSizeJ)) APPLY_CELL_OPERATION_TO_CELL(nI + i, nJ - j, nK);
00675             if((nI - i >= 0 && nI - i < m_nSizeI) && (nJ + j >= 0 && nJ + j < m_nSizeJ)) APPLY_CELL_OPERATION_TO_CELL(nI - i, nJ + j, nK);
00676             if((nI - i >= 0 && nI - i < m_nSizeI) && (nJ - j >= 0 && nJ - j < m_nSizeJ)) APPLY_CELL_OPERATION_TO_CELL(nI - i, nJ - j, nK);
00677          }
00678       }
00679    }
00680 
00681    /****************************************/
00682    /****************************************/
00683 
00684    template<class ENTITY>
00685    void CGrid<ENTITY>::ForCellsInRectangleRange(const CVector3& c_center,
00686                                                 const CVector2& c_half_size,
00687                                                 CCellOperation& c_operation) {
00688       /* Calculate cell range */
00689       SInt32 nI1 = Min<SInt32>(m_nSizeI-1, Max<SInt32>(0, Floor((c_center.GetX() - c_half_size.GetX() - m_cAreaMinCorner.GetX()) * m_cInvCellSize.GetX())));
00690       SInt32 nJ1 = Min<SInt32>(m_nSizeJ-1, Max<SInt32>(0, Floor((c_center.GetY() - c_half_size.GetY() - m_cAreaMinCorner.GetY()) * m_cInvCellSize.GetY())));
00691       SInt32 nI2 = Min<SInt32>(m_nSizeI-1, Max<SInt32>(0, Floor((c_center.GetX() + c_half_size.GetX() - m_cAreaMinCorner.GetX()) * m_cInvCellSize.GetX())));
00692       SInt32 nJ2 = Min<SInt32>(m_nSizeJ-1, Max<SInt32>(0, Floor((c_center.GetY() + c_half_size.GetY() - m_cAreaMinCorner.GetY()) * m_cInvCellSize.GetY())));
00693       SInt32 nK  = Min<SInt32>(m_nSizeK-1, Max<SInt32>(0, Floor((c_center.GetZ()                      - m_cAreaMinCorner.GetZ()) * m_cInvCellSize.GetZ())));
00694       /* Go through cells */
00695       for(SInt32 j = nJ1; j <= nJ2; ++j) {
00696          for(SInt32 i = nI1; i <= nI2; ++i) {
00697             APPLY_CELL_OPERATION_TO_CELL(i, j, nK);
00698          }
00699       }
00700    }
00701 
00702    /****************************************/
00703    /****************************************/
00704 
00705    template<class ENTITY>
00706    void CGrid<ENTITY>::ForCellsAlongRay(const CRay3& c_ray,
00707                                         CCellOperation& c_operation) {
00708       /* Transform ray start and end position into cell coordinates */
00709       SInt32 nI1, nJ1, nK1, nI2, nJ2, nK2;
00710       PositionToCellUnsafe(nI1, nJ1, nK1, c_ray.GetStart());
00711       ClampCoordinates(nI1, nJ1, nK1);
00712       PositionToCellUnsafe(nI2, nJ2, nK2, c_ray.GetEnd());
00713       ClampCoordinates(nI2, nJ2, nK2);
00714       /* Go through cells one by one, from start to end.
00715          Stop as soon as an entity is found.
00716          If the loop is completed, it means no entities were found -> no intersection.
00717       */
00718       /* Calculate deltas for later use */
00719       SInt32 nDI(Abs(nI2 - nI1));
00720       SInt32 nDJ(Abs(nJ2 - nJ1));
00721       SInt32 nDK(Abs(nK2 - nK1));
00722       /* Calculate the increment for each direction */
00723       SInt32 nSI(nI2 >= nI1 ? 1 : -1);
00724       SInt32 nSJ(nJ2 >= nJ1 ? 1 : -1);
00725       SInt32 nSK(nK2 >= nK1 ? 1 : -1);
00726       /* Set the starting cell */
00727       SInt32 nI(nI1), nJ(nJ1), nK(nK1);
00728       if(nDI >= nDJ && nDI >= nDK) {
00729          /* I is the driving axis */
00730          /* Calculate error used to know when to move on other axes */
00731          SInt32 nEJ(3 * nDJ - nDI);
00732          SInt32 nEK(3 * nDK - nDI);
00733          APPLY_CELL_OPERATION_TO_CELL(nI, nJ, nK);
00734          /* Cycle through cells */
00735          for(SInt32 nCell = nDI; nCell > 0; --nCell) {
00736             /* Advance on driving axis */
00737             nI += nSI;
00738             APPLY_CELL_OPERATION_TO_CELL(nI, nJ, nK);
00739             /* Advance on other axes, if necessary */
00740             if(nEJ > 0 && nEK > 0) {
00741                /* Advance on both the other axes */
00742                if(nEJ * nDK > nEK * nDJ) {
00743                   nJ += nSJ;
00744                   APPLY_CELL_OPERATION_TO_CELL(nI, nJ, nK);
00745                   nK += nSK;
00746                   APPLY_CELL_OPERATION_TO_CELL(nI, nJ, nK);
00747                }
00748                else {
00749                   nK += nSK;
00750                   APPLY_CELL_OPERATION_TO_CELL(nI, nJ, nK);
00751                   nJ += nSJ;
00752                   APPLY_CELL_OPERATION_TO_CELL(nI, nJ, nK);
00753                }
00754                nEJ += 2 * (nDJ - nDI);
00755                nEK += 2 * (nDK - nDI);
00756             }
00757             else if(nEJ > 0) {
00758                /* Advance only on J */
00759                nJ += nSJ;
00760                APPLY_CELL_OPERATION_TO_CELL(nI, nJ, nK);
00761                nEJ += 2 * (nDJ - nDI);
00762                nEK += 2 * nDK;
00763             }
00764             else {
00765                nEJ += 2 * nDJ;
00766                if(nEK > 0) {
00767                   /* Advance only on K */
00768                   nK += nSK;
00769                   APPLY_CELL_OPERATION_TO_CELL(nI, nJ, nK);
00770                   nEK += 2 * (nDK - nDI);
00771                }
00772                else {
00773                   nEK += 2 * nDK;
00774                }
00775             }
00776          }
00777       }
00778       else if(nDJ >= nDI && nDJ >= nDK) {
00779          /* J is the driving axis */
00780          /* Calculate error used to know when to move on other axes */
00781          SInt32 nEI(3 * nDI - nDJ);
00782          SInt32 nEK(3 * nDK - nDJ);
00783          APPLY_CELL_OPERATION_TO_CELL(nI, nJ, nK);
00784          /* Cycle through cells */
00785          for(SInt32 nCell = nDJ; nCell > 0; --nCell) {
00786             /* Advance on driving axis */
00787             nJ += nSJ;
00788             APPLY_CELL_OPERATION_TO_CELL(nI, nJ, nK);
00789             /* Advance on other axes, if necessary */
00790             if(nEI > 0 && nEK > 0) {
00791                /* Advance on both the other axes */
00792                if(nEI * nDK > nEK * nDI) {
00793                   nI += nSI;
00794                   APPLY_CELL_OPERATION_TO_CELL(nI, nJ, nK);
00795                   nK += nSK;
00796                   APPLY_CELL_OPERATION_TO_CELL(nI, nJ, nK);
00797                }
00798                else {
00799                   nK += nSK;
00800                   APPLY_CELL_OPERATION_TO_CELL(nI, nJ, nK);
00801                   nI += nSI;
00802                   APPLY_CELL_OPERATION_TO_CELL(nI, nJ, nK);
00803                }
00804                nEI += 2 * (nDI - nDJ);
00805                nEK += 2 * (nDK - nDJ);
00806             }
00807             else if(nEI > 0) {
00808                /* Advance only on I */
00809                nI += nSI;
00810                APPLY_CELL_OPERATION_TO_CELL(nI, nJ, nK);
00811                nEI += 2 * (nDI - nDJ);
00812                nEK += 2 * nDK;
00813             }
00814             else {
00815                nEI += 2 * nDI;
00816                if(nEK > 0) {
00817                   /* Advance only on K */
00818                   nK += nSK;
00819                   APPLY_CELL_OPERATION_TO_CELL(nI, nJ, nK);
00820                   nEK += 2 * (nDK - nDJ);
00821                }
00822                else {
00823                   nEK += 2 * nDK;
00824                }
00825             }
00826          }
00827       }
00828       else {
00829          /* K is the driving axis */
00830          /* Calculate error used to know when to move on other axes */
00831          SInt32 nEI(3 * nDI - nDK);
00832          SInt32 nEJ(3 * nDJ - nDK);
00833          APPLY_CELL_OPERATION_TO_CELL(nI, nJ, nK);
00834          /* Cycle through cells */
00835          for(SInt32 nCell = nDK; nCell > 0; --nCell) {
00836             /* Advance on driving axis */
00837             nK += nSK;
00838             APPLY_CELL_OPERATION_TO_CELL(nI, nJ, nK);
00839             /* Advance on other axes, if necessary */
00840             if(nEI > 0 && nEJ > 0) {
00841                /* Advance on both the other axes */
00842                if(nEI * nDJ > nEJ * nDI) {
00843                   nI += nSI;
00844                   APPLY_CELL_OPERATION_TO_CELL(nI, nJ, nK);
00845                   nJ += nSJ;
00846                   APPLY_CELL_OPERATION_TO_CELL(nI, nJ, nK);
00847                }
00848                else {
00849                   nJ += nSJ;
00850                   APPLY_CELL_OPERATION_TO_CELL(nI, nJ, nK);
00851                   nI += nSI;
00852                   APPLY_CELL_OPERATION_TO_CELL(nI, nJ, nK);
00853                }
00854                nEI += 2 * (nDI - nDK);
00855                nEJ += 2 * (nDJ - nDK);
00856             }
00857             else if(nEI > 0) {
00858                /* Advance only on I */
00859                nI += nSI;
00860                APPLY_CELL_OPERATION_TO_CELL(nI, nJ, nK);
00861                nEI += 2 * (nDI - nDK);
00862                nEJ += 2 * nDJ;
00863             }
00864             else {
00865                nEI += 2 * nDI;
00866                if(nEJ > 0) {
00867                   /* Advance only on J */
00868                   nJ += nSJ;
00869                   APPLY_CELL_OPERATION_TO_CELL(nI, nJ, nK);
00870                   nEJ += 2 * (nDJ - nDK);
00871                }
00872                else {
00873                   nEJ += 2 * nDJ;
00874                }
00875             }
00876          }
00877       }
00878    }
00879 
00880    /****************************************/
00881    /****************************************/
00882 
00883    template<class ENTITY>
00884    void CGrid<ENTITY>::UpdateCell(SInt32 n_i,
00885                                   SInt32 n_j,
00886                                   SInt32 n_k,
00887                                   ENTITY& c_entity) {
00888       if((n_i >= 0) && (n_i < m_nSizeI) &&
00889          (n_j >= 0) && (n_j < m_nSizeJ) &&
00890          (n_k >= 0) && (n_k < m_nSizeK)) {
00891          SCell& sCell = GetCellAt(n_i, n_j, n_k);
00892          if(sCell.Timestamp < m_unCurTimestamp) {
00893             sCell.Entities.clear();
00894             sCell.Timestamp = m_unCurTimestamp;
00895          }
00896          sCell.Entities.insert(&c_entity);
00897       }
00898       else {
00899          THROW_ARGOSEXCEPTION("CGrid<ENTITY>::UpdateCell() : index (" << n_i << "," << n_j << "," << n_k << ") out of bounds (" << m_nSizeI-1 << "," << m_nSizeJ-1 << "," << m_nSizeK-1 << ")");
00900       }
00901    }
00902 
00903    /****************************************/
00904    /****************************************/
00905 
00906    template<class ENTITY>
00907    void CGrid<ENTITY>::SetUpdateEntityOperation(CEntityOperation* pc_operation) {
00908       m_pcUpdateEntityOperation = pc_operation;
00909    }
00910 
00911    /****************************************/
00912    /****************************************/
00913 
00914    template<class ENTITY>
00915    void CGrid<ENTITY>::PositionToCell(SInt32& n_i,
00916                                       SInt32& n_j,
00917                                       SInt32& n_k,
00918                                       const CVector3& c_position) const {
00919       if(m_cRangeX.WithinMinBoundIncludedMaxBoundIncluded(c_position.GetX()) &&
00920          m_cRangeY.WithinMinBoundIncludedMaxBoundIncluded(c_position.GetY()) &&
00921          m_cRangeZ.WithinMinBoundIncludedMaxBoundIncluded(c_position.GetZ())) {
00922          n_i = Floor((c_position.GetX() - m_cAreaMinCorner.GetX()) * m_cInvCellSize.GetX());
00923          n_j = Floor((c_position.GetY() - m_cAreaMinCorner.GetY()) * m_cInvCellSize.GetY());
00924          n_k = Floor((c_position.GetZ() - m_cAreaMinCorner.GetZ()) * m_cInvCellSize.GetZ());
00925       }
00926       else {
00927          THROW_ARGOSEXCEPTION("CGrid<ENTITY>::PositionToCell() : Position <" << c_position << "> out of bounds X -> " << m_cRangeX << " Y -> " << m_cRangeY << " Z -> " << m_cRangeZ);
00928       }
00929    }
00930 
00931    /****************************************/
00932    /****************************************/
00933 
00934    template<class ENTITY>
00935    void CGrid<ENTITY>::PositionToCellUnsafe(SInt32& n_i,
00936                                             SInt32& n_j,
00937                                             SInt32& n_k,
00938                                             const CVector3& c_position) const {
00939       n_i = Floor((c_position.GetX() - m_cAreaMinCorner.GetX()) * m_cInvCellSize.GetX());
00940       n_j = Floor((c_position.GetY() - m_cAreaMinCorner.GetY()) * m_cInvCellSize.GetY());
00941       n_k = Floor((c_position.GetZ() - m_cAreaMinCorner.GetZ()) * m_cInvCellSize.GetZ());
00942    }
00943 
00944    /****************************************/
00945    /****************************************/
00946 
00947    template<class ENTITY>
00948    void CGrid<ENTITY>::ClampCoordinates(SInt32& n_i,
00949                                         SInt32& n_j,
00950                                         SInt32& n_k) const {
00951       if(n_i < 0) n_i = 0;
00952       else if(n_i >= m_nSizeI) n_i = m_nSizeI - 1;
00953       if(n_j < 0) n_j = 0;
00954       else if(n_j >= m_nSizeJ) n_j = m_nSizeJ - 1;
00955       if(n_k < 0) n_k = 0;
00956       else if(n_k >= m_nSizeK) n_k = m_nSizeK - 1;
00957    }
00958 
00959    /****************************************/
00960    /****************************************/
00961    
00962    template<class ENTITY>
00963    void CGrid<ENTITY>::ClampCoordinates(CVector3& c_pos) const {
00964       if(c_pos.GetX() < m_cRangeX.GetMin()) c_pos.SetX(m_cRangeX.GetMin() + EPSILON);
00965       else if(c_pos.GetX() > m_cRangeX.GetMax()) c_pos.SetX(m_cRangeX.GetMax() - EPSILON);
00966       if(c_pos.GetY() < m_cRangeY.GetMin()) c_pos.SetY(m_cRangeY.GetMin() + EPSILON);
00967       else if(c_pos.GetY() > m_cRangeY.GetMax()) c_pos.SetY(m_cRangeY.GetMax() - EPSILON);
00968       if(c_pos.GetZ() < m_cRangeZ.GetMin()) c_pos.SetZ(m_cRangeZ.GetMin() + EPSILON);
00969       else if(c_pos.GetZ() > m_cRangeZ.GetMax()) c_pos.SetZ(m_cRangeZ.GetMax() - EPSILON);
00970    }
00971 
00972    /****************************************/
00973    /****************************************/
00974    
00975    template<class ENTITY>
00976    typename CGrid<ENTITY>::SCell& CGrid<ENTITY>::GetCellAt(SInt32 n_i,
00977                                                            SInt32 n_j,
00978                                                            SInt32 n_k) {
00979       return m_psCells[m_nSizeI * m_nSizeJ * n_k +
00980                        m_nSizeI * n_j +
00981                        n_i];
00982    }
00983 
00984    /****************************************/
00985    /****************************************/
00986 
00987    template<class ENTITY>
00988    const typename CGrid<ENTITY>::SCell& CGrid<ENTITY>::GetCellAt(SInt32 n_i,
00989                                                                  SInt32 n_j,
00990                                                                  SInt32 n_k) const {
00991       return m_psCells[m_nSizeI * m_nSizeJ * n_k +
00992                        m_nSizeI * n_j +
00993                        n_i];
00994    }
00995 
00996    /****************************************/
00997    /****************************************/
00998    
00999 }
```

---

Generated on 10 Jul 2018 for ARGoS by 
 1.6.1 
