# Supplementary material for: Iterative improvement in the automatic modular design of robot swarms
Source: PeerJ Comput Sci. 2020 Dec 7;6:e322. doi: 10.7717/peerj-cs.322 (PMC7924708; doi:10.7717/peerj-cs.322)
Supplement: Supplemental Information 3 [file peerj-cs-06-322-s003.zip › argos3/doc/api/standalone/a00334_source.html]

ARGoS: core/simulator/space/positional\_indices/positional\_index.h Source File


- Main Page
- Related Pages
- Namespaces
- Classes
- Files

- File List
- File Members

# core/simulator/space/positional\_indices/positional\_index.h

Go to the documentation of this file.

```
00001 #ifndef POSITIONAL_INDEX_H
00002 #define POSITIONAL_INDEX_H
00003 
00004 namespace argos {
00005    class CVector2;
00006    class CVector3;
00007    class CRay3;
00008 }
00009 
00010 #include <argos3/core/utility/configuration/base_configurable_resource.h>
00011 #include <argos3/core/utility/datatypes/datatypes.h>
00012 #include <argos3/core/utility/datatypes/set.h>
00013 #include <argos3/core/utility/math/vector2.h>
00014 #include <argos3/core/utility/math/vector3.h>
00015 
00016 #include <string>
00017 
00018 namespace argos {
00019 
00028    template<class ENTITY>
00029    class CPositionalIndex : CBaseConfigurableResource {
00030 
00031    public:
00032 
00059       class COperation {
00060       public:
00061          virtual ~COperation() {}
00062          virtual bool operator()(ENTITY&) = 0;
00063       };
00064 
00065    public:
00066 
00067       CPositionalIndex() {}
00068       virtual ~CPositionalIndex() {}
00069 
00070       virtual void Init(TConfigurationNode& t_tree) = 0;
00071       virtual void Reset() = 0;
00072       virtual void Destroy() = 0;
00073 
00078       virtual void AddEntity(ENTITY& c_entity) = 0;
00079 
00084       virtual void RemoveEntity(ENTITY& c_entity) = 0;
00085 
00089       virtual void Update() = 0;
00090 
00096       virtual void GetEntitiesAt(CSet<ENTITY*>& c_entities,
00097                                  const CVector3& c_position) const = 0;
00098 
00104       virtual void ForAllEntities(COperation& c_operation) = 0;
00105       
00113       virtual void ForEntitiesInSphereRange(const CVector3& c_center,
00114                                             Real f_radius,
00115                                             COperation& c_operation) = 0;
00116       
00125       virtual void ForEntitiesInBoxRange(const CVector3& c_center,
00126                                          const CVector3& c_half_size,
00127                                          COperation& c_operation) = 0;
00128       
00137       virtual void ForEntitiesInCircleRange(const CVector3& c_center,
00138                                             Real f_radius,
00139                                             COperation& c_operation) = 0;
00140       
00149       virtual void ForEntitiesInRectangleRange(const CVector3& c_center,
00150                                                const CVector2& c_half_size,
00151                                                COperation& c_operation) = 0;
00152 
00164       virtual void ForEntitiesAlongRay(const CRay3& c_ray,
00165                                        COperation& c_operation,
00166                                        bool b_stop_at_closest_match = false) = 0;
00167 
00168    };
00169 
00170 }
00171 
00172 #endif
```

---

Generated on 10 Jul 2018 for ARGoS by 
 1.6.1 
