# Supplementary material for: Iterative improvement in the automatic modular design of robot swarms
Source: PeerJ Comput Sci. 2020 Dec 7;6:e322. doi: 10.7717/peerj-cs.322 (PMC7924708; doi:10.7717/peerj-cs.322)
Supplement: Supplemental Information 3 [file peerj-cs-06-322-s003.zip › argos3/doc/api/standalone/a00335_source.html]

ARGoS: core/simulator/space/positional\_indices/space\_hash.h Source File


- Main Page
- Related Pages
- Namespaces
- Classes
- Files

- File List
- File Members

# core/simulator/space/positional\_indices/space\_hash.h

Go to the documentation of this file.

```
00001 
00007 #ifndef SPACE_HASH_H
00008 #define SPACE_HASH_H
00009 
00010 namespace argos {
00011    class CSpace;
00012    class CVector3;
00013    class CRay3;
00014 }
00015 
00016 #include <argos3/core/utility/math/ray3.h>
00017 #include <argos3/core/utility/datatypes/set.h>
00018 #include <argos3/core/simulator/space/positional_indices/positional_index.h>
00019 
00020 namespace argos {
00021 
00022    /****************************************/
00023    /****************************************/
00024 
00033    template <class ENTITY>
00034    class CAbstractSpaceHash : public CPositionalIndex<ENTITY> {
00035 
00036    public:
00037 
00041       typedef CSet<ENTITY*> TEntityList;
00042 
00043    public:
00044 
00052       CAbstractSpaceHash() : m_unSize(0) {}
00053 
00057       virtual ~CAbstractSpaceHash() {}
00058 
00063       virtual void AddEntity(ENTITY& c_entity) {
00064          m_tEntities.insert(&c_entity);
00065       }
00066 
00071       inline TEntityList& GetEntities() {
00072          return m_tEntities;
00073       }
00074 
00079       virtual void RemoveEntity(ENTITY& c_entity) {
00080          typename TEntityList::iterator it = m_tEntities.find(&c_entity);
00081          if(it != m_tEntities.end()) {
00082             m_tEntities.erase(it);
00083          }
00084          else {
00085             THROW_ARGOSEXCEPTION("Entity not found when removing it from space hash.");
00086          }
00087       }
00088 
00094       inline size_t GetSize() {
00095          return m_unSize;
00096       }
00097 
00103       virtual void SetSize(size_t un_size) {
00104          m_unSize = un_size;
00105       }
00106 
00112       inline CVector3& GetCellSize() {
00113          return m_cCellSize;
00114       }
00115 
00122       inline CVector3& GetInvCellSize() {
00123          return m_cInvCellSize;
00124       }
00125 
00131       virtual void SetCellSize(const CVector3& c_cell_size) {
00132          m_cCellSize = c_cell_size;
00133          m_cInvCellSize.Set(1.0f / m_cCellSize.GetX(),
00134                             1.0f / m_cCellSize.GetY(),
00135                             1.0f / m_cCellSize.GetZ());
00136       }
00137 
00143       virtual void Update() = 0;
00144 
00152       virtual void UpdateCell(SInt32 n_x,
00153                               SInt32 n_y,
00154                               SInt32 n_z,
00155                               ENTITY& c_entity) = 0;
00156 
00163       virtual SInt32 SpaceToHashTable(Real f_coord,
00164                                              UInt32 un_axis) {
00165          return RoundClosestToZero(f_coord * GetInvCellSize()[un_axis]);
00166       }
00167 
00174       virtual Real HashTableToSpace(SInt32 n_coord,
00175                                            UInt32 un_axis) {
00176          return n_coord * m_cCellSize[un_axis];
00177       }
00178 
00187       virtual void SpaceToHashTable(SInt32& n_i,
00188                                            SInt32& n_j,
00189                                            SInt32& n_k,
00190                                            const CVector3& c_pos) {
00191          n_i = RoundClosestToZero(c_pos.GetX() * CAbstractSpaceHash::GetInvCellSize().GetX());
00192          n_j = RoundClosestToZero(c_pos.GetY() * CAbstractSpaceHash::GetInvCellSize().GetY());
00193          n_k = RoundClosestToZero(c_pos.GetZ() * CAbstractSpaceHash::GetInvCellSize().GetZ());
00194       }
00195 
00204       virtual bool CheckCell(SInt32 n_i,
00205                              SInt32 n_j,
00206                              SInt32 n_k,
00207                              TEntityList& t_entities) = 0;
00208 
00209       virtual void Dump(CARGoSLog& c_os) = 0;
00210 
00211    protected:
00212 
00220       inline UInt32 CoordinateHash(SInt32 n_i,
00221                                    SInt32 n_j,
00222                                    SInt32 n_k) {
00223          return
00224             ((73856093u * n_i) ^
00225              (19349663u * n_j) ^
00226              (83492791u * n_k)) %
00227             m_unSize;
00228       }
00229       
00230    private:
00231 
00235       TEntityList m_tEntities;
00236 
00240       size_t m_unSize;
00241 
00246       CVector3 m_cCellSize;
00247 
00252       CVector3 m_cInvCellSize;
00253 
00254    };
00255 
00256    /****************************************/
00257    /****************************************/
00258 
00268    template <class ENTITY>
00269    class CSpaceHashUpdater {
00270 
00271      public:
00272 
00276       virtual ~CSpaceHashUpdater() {}
00277 
00283       virtual void operator()(CAbstractSpaceHash<ENTITY>& c_space_hash,
00284                               ENTITY& c_entity) = 0;
00285 
00286    };
00287 
00288    /****************************************/
00289    /****************************************/
00290 
00299    template <class ENTITY, class UPDATER>
00300    class CSpaceHash : public CAbstractSpaceHash<ENTITY> {
00301 
00302    public:
00303 
00309       virtual void Update() {
00310          /* Go through all the entities */
00311          for(typename CAbstractSpaceHash<ENTITY>::TEntityList::const_iterator el = CAbstractSpaceHash<ENTITY>::GetEntities().begin();
00312              el != CAbstractSpaceHash<ENTITY>::GetEntities().end(); ++el) {
00313             m_cUpdater(*this, **el);
00314          }
00315       }
00316 
00317    private:
00318 
00319       UPDATER m_cUpdater;
00320 
00321    };
00322 
00323    /****************************************/
00324    /****************************************/
00325 
00326 }
00327 
00328 #endif
```

---

Generated on 10 Jul 2018 for ARGoS by 
 1.6.1 
