# Supplementary material for: Iterative improvement in the automatic modular design of robot swarms
Source: PeerJ Comput Sci. 2020 Dec 7;6:e322. doi: 10.7717/peerj-cs.322 (PMC7924708; doi:10.7717/peerj-cs.322)
Supplement: Supplemental Information 3 [file peerj-cs-06-322-s003.zip › argos3/doc/api/standalone/a00336_source.html]

ARGoS: core/simulator/space/positional\_indices/space\_hash\_native.h Source File


- Main Page
- Related Pages
- Namespaces
- Classes
- Files

- File List
- File Members

# core/simulator/space/positional\_indices/space\_hash\_native.h

Go to the documentation of this file.

```
00001 
00007 #ifndef SPACE_HASH_NATIVE_H
00008 #define SPACE_HASH_NATIVE_H
00009 
00010 #include <argos3/core/simulator/space/space_hash.h>
00011 
00012 namespace argos {
00013 
00018    template <class Element, class Updater>
00019    class CSpaceHashNative : public CSpaceHash<Element,Updater> {
00020 
00021    private:
00022 
00026       struct SBucket {
00027 
00032          struct SBucketData {
00036             Element* Elem;
00040             SInt32 I,J,K;
00044             SBucketData* Next;
00045 
00053             SBucketData(Element& c_element,
00054                         SInt32 n_i,
00055                         SInt32 n_j,
00056                         SInt32 n_k,
00057                         SBucketData* ps_next = NULL) :
00058                Elem(&c_element),
00059                I(n_i),
00060                J(n_j),
00061                K(n_k),
00062                Next(ps_next) {}
00063 
00064          };
00065 
00069          UInt64 StoreTimestamp;
00070 
00074          SBucketData* ElementList;
00075 
00080          SBucket() :
00081             StoreTimestamp(0),
00082             ElementList(NULL) {}
00083 
00089          ~SBucket() {
00090             Clear();
00091          }
00092 
00097          inline bool Empty() const {
00098             return (ElementList == NULL);
00099          }
00100 
00105          inline void Clear() {
00106             if(!Empty()) {
00107                SBucketData* psCur = ElementList;
00108                SBucketData* psNext = psCur->Next;
00109                do {
00110                   delete psCur;
00111                   psCur = psNext;
00112                   if(psCur) psNext = psCur->Next;
00113                } while(psCur);
00114                ElementList = NULL;
00115             }
00116          }
00117 
00125          inline void Add(Element& c_element,
00126                          SInt32 n_i,
00127                          SInt32 n_j,
00128                          SInt32 n_k) {
00129             if(Empty()) ElementList = new SBucketData(c_element, n_i, n_j, n_k);
00130             else ElementList = new SBucketData(c_element, n_i, n_j, n_k, ElementList);
00131          }
00132 
00140          bool Exists(const Element& c_element,
00141                      SInt32 n_i,
00142                      SInt32 n_j,
00143                      SInt32 n_k) {
00144             SBucketData* psCur = ElementList;
00145             while(psCur) {
00146                if(psCur->Elem == &c_element &&
00147                   psCur->I == n_i &&
00148                   psCur->J == n_j &&
00149                   psCur->K == n_k) return true;
00150                psCur = psCur->Next;
00151             }
00152             return false;
00153          }
00154 
00155       };
00156 
00157    public:
00158 
00166       CSpaceHashNative() :
00167          m_psBuckets(NULL),
00168          m_unCurrentStoreTimestamp(0) {}
00169 
00173       ~CSpaceHashNative() {
00174          Clear();
00175          delete[] m_psBuckets;
00176       }
00177 
00181       inline void Clear() {
00182          for(size_t i = 0; i < CSpaceHash<Element,Updater>::GetSize(); ++i) {
00183             m_psBuckets[i].Clear();
00184          }
00185       }
00186 
00192       inline virtual void SetSize(size_t un_size) {
00193          CSpaceHash<Element,Updater>::SetSize(un_size);
00194          m_psBuckets = new SBucket[CSpaceHash<Element,Updater>::GetSize()];
00196       }
00197 
00203       inline virtual void Update() {
00204          /* Set the current store time stamp */
00205          m_unCurrentStoreTimestamp++;
00206          /* Call base class method */
00207          CSpaceHash<Element,Updater>::Update();
00208       }
00209 
00217       inline virtual void UpdateCell(SInt32 n_i,
00218                                      SInt32 n_j,
00219                                      SInt32 n_k,
00220                                      Element& c_element) {
00221          /* Calculate the hash of the current position */
00222          SInt32 nHash = CSpaceHash<Element,Updater>::CoordinateHash(n_i, n_j, n_k);
00223          /* Get a reference to the bucket */
00224          SBucket& sBucket = m_psBuckets[nHash];
00225          /* Check if the bucket's content is obsolete */
00226          if(sBucket.StoreTimestamp == m_unCurrentStoreTimestamp) {
00227             /* Add the current element to the bucket */
00228             if(! sBucket.Exists(c_element, n_i, n_j, n_k)) {
00229                sBucket.Add(c_element, n_i, n_j, n_k);
00230             }
00231          }
00232          else {
00233             /* The bucket's content is obsolete, erase it */
00234             sBucket.Clear();
00235             /* Set the store timestamp to the current time */
00236             sBucket.StoreTimestamp = m_unCurrentStoreTimestamp;
00237             /* Add the current element to the bucket */
00238             sBucket.Add(c_element, n_i, n_j, n_k);
00239          }
00240       }
00241 
00249       virtual bool CheckCell(SInt32 n_i,
00250                              SInt32 n_j,
00251                              SInt32 n_k,
00252                              typename CSpaceHash<Element,Updater>::TElementList& t_elements) {
00253          /* In the beginning, no new elements have been found */
00254          bool bNewElements = false;
00255          /* Calculate the hash of the current position */
00256          SInt32 nHash = CSpaceHash<Element,Updater>::CoordinateHash(n_i, n_j, n_k);
00257          /* Get a reference to the bucket */
00258          SBucket& sBucket = m_psBuckets[nHash];
00259          /* Visit the bucket IF:
00260             1. its data is up-to-date AND
00261             2. it is not empty
00262           */
00263          if((sBucket.StoreTimestamp == m_unCurrentStoreTimestamp) && /* 1. */
00264             !sBucket.Empty()) /* 2. */ {
00265             /* Check the bucket's elements */
00266             for(typename SBucket::SBucketData* psCur = sBucket.ElementList;
00267                 psCur;
00268                 psCur = psCur->Next) {
00269                /* Check that the element is in the wanted cell */
00270                if(n_i == psCur->I &&
00271                   n_j == psCur->J &&
00272                   n_k == psCur->K) {
00273                   /* We have a new element to add to the list */
00274                   bNewElements = true;
00275                   t_elements.insert(psCur->Elem);
00276                }
00277             }
00278          }
00279          return bNewElements;
00280       }
00281 
00282       virtual void Dump(CARGoSLog& c_os) {
00283          for(size_t i = 0; i < CSpaceHash<Element,Updater>::GetSize(); ++i) {
00284             if((m_psBuckets[i].StoreTimestamp == m_unCurrentStoreTimestamp) &&
00285                !m_psBuckets[i].Empty()) {
00286                c_os << "BUCKET " << i << std::endl;
00287                for(typename SBucket::SBucketData* psCur = m_psBuckets[i].ElementList;
00288                    psCur;
00289                    psCur = psCur->Next) {
00290                   c_os << "   "
00291                        << psCur->Elem->GetId()
00292                        << " ("
00293                        << psCur->I
00294                        << ","
00295                        << psCur->J
00296                        << ","
00297                        << psCur->K
00298                        << ")"
00299                        << std::endl;
00300                }
00301             }
00302          }
00303       }
00304       
00305    private:
00306 
00310       SBucket* m_psBuckets;
00311 
00319       UInt64 m_unCurrentStoreTimestamp;
00320 
00321    };
00322 
00323 }
00324 
00325 #endif
```

---

Generated on 10 Jul 2018 for ARGoS by 
 1.6.1 
