# Supplementary material for: Iterative improvement in the automatic modular design of robot swarms
Source: PeerJ Comput Sci. 2020 Dec 7;6:e322. doi: 10.7717/peerj-cs.322 (PMC7924708; doi:10.7717/peerj-cs.322)
Supplement: Supplemental Information 3 [file peerj-cs-06-322-s003.zip › argos3/doc/api/standalone/a00337_source.html]

ARGoS: core/simulator/space/space.cpp Source File


- Main Page
- Related Pages
- Namespaces
- Classes
- Files

- File List
- File Members

# core/simulator/space/space.cpp

Go to the documentation of this file.

```
00001 
00011 #include <argos3/core/utility/string_utilities.h>
00012 #include <argos3/core/utility/math/range.h>
00013 #include <argos3/core/utility/logging/argos_log.h>
00014 #include <argos3/core/utility/math/rng.h>
00015 #include <argos3/core/simulator/simulator.h>
00016 #include <argos3/core/simulator/entity/composable_entity.h>
00017 #include <argos3/core/simulator/entity/positional_entity.h>
00018 #include <argos3/core/simulator/loop_functions.h>
00019 #include <cstring>
00020 #include "space.h"
00021 
00022 namespace argos {
00023 
00024    /****************************************/
00025    /****************************************/
00026 
00027    CSpace::CSpace() :
00028       m_cSimulator(CSimulator::GetInstance()),
00029       m_unSimulationClock(0),
00030       m_pcFloorEntity(NULL),
00031       m_ptPhysicsEngines(NULL),
00032       m_ptMedia(NULL) {}
00033    
00034    /****************************************/
00035    /****************************************/
00036 
00037    void CSpace::Init(TConfigurationNode& t_tree) {
00038       /* Get reference to physics engine and media vectors */
00039       m_ptPhysicsEngines = &(m_cSimulator.GetPhysicsEngines());
00040       m_ptMedia = &(m_cSimulator.GetMedia());
00041       /* Get the arena center and size */
00042       GetNodeAttributeOrDefault(t_tree, "center", m_cArenaCenter, m_cArenaCenter);
00043       GetNodeAttribute(t_tree, "size", m_cArenaSize);
00044       m_cArenaLimits.Set(m_cArenaCenter - m_cArenaSize / 2.0f,
00045                          m_cArenaCenter + m_cArenaSize / 2.0f);
00046       /*
00047        * Add and initialize all entities in XML
00048        */
00049       /* Start from the entities placed manually */
00050       TConfigurationNodeIterator itArenaItem;
00051       for(itArenaItem = itArenaItem.begin(&t_tree);
00052           itArenaItem != itArenaItem.end();
00053           ++itArenaItem) {
00054          if(itArenaItem->Value() != "distribute") {
00055             CEntity* pcEntity = CFactory<CEntity>::New(itArenaItem->Value());
00056             pcEntity->Init(*itArenaItem);
00057             CallEntityOperation<CSpaceOperationAddEntity, CSpace, void>(*this, *pcEntity);
00058          }
00059       }
00060       /* Place the entities to distribute automatically */
00061       for(itArenaItem = itArenaItem.begin(&t_tree);
00062           itArenaItem != itArenaItem.end();
00063           ++itArenaItem) {
00064          if(itArenaItem->Value() == "distribute") {
00065             Distribute(*itArenaItem);
00066          }
00067       }
00068    }
00069 
00070    /****************************************/
00071    /****************************************/
00072 
00073    void CSpace::Reset() {
00074       /* Reset the simulation clock */
00075       m_unSimulationClock = 0;
00076       /* Reset the entities */
00077       for(UInt32 i = 0; i < m_vecEntities.size(); ++i) {
00078          m_vecEntities[i]->Reset();
00079       }
00080    }
00081 
00082    /****************************************/
00083    /****************************************/
00084 
00085    void CSpace::Destroy() {
00086       /* Remove all entities */
00087       while(!m_vecRootEntities.empty()) {
00088          CallEntityOperation<CSpaceOperationRemoveEntity, CSpace, void>(*this, *m_vecRootEntities.back());
00089       }
00090    }
00091 
00092    /****************************************/
00093    /****************************************/
00094 
00095    void CSpace::GetEntitiesMatching(CEntity::TVector& t_buffer,
00096                                     const std::string& str_pattern) {
00097       for(CEntity::TVector::iterator it = m_vecEntities.begin();
00098           it != m_vecEntities.end(); ++it) {
00099          if(MatchPattern((*it)->GetId(), str_pattern)) {
00100             t_buffer.push_back(*it);
00101          }
00102       }
00103    }
00104 
00105    /****************************************/
00106    /****************************************/
00107 
00108    CSpace::TMapPerType& CSpace::GetEntitiesByType(const std::string& str_type) {
00109       TMapPerTypePerId::iterator itEntities = m_mapEntitiesPerTypePerId.find(str_type);
00110       if (itEntities != m_mapEntitiesPerTypePerId.end()){
00111          return itEntities->second;
00112       }
00113       else {
00114          THROW_ARGOSEXCEPTION("Entity map for type \"" << str_type << "\" not found.");
00115       }
00116    }
00117 
00118    /****************************************/
00119    /****************************************/
00120 
00121    void CSpace::Update() {
00122       /* Increase the simulation clock */
00123       IncreaseSimulationClock();
00124       /* Perform the 'act' phase for controllable entities */
00125       UpdateControllableEntitiesAct();
00126       /* Update the physics engines */
00127       UpdatePhysics();
00128       /* Update media */
00129       UpdateMedia();
00130       /* Call loop functions */
00131       m_cSimulator.GetLoopFunctions().PreStep();
00132       /* Perform the 'sense+step' phase for controllable entities */
00133       UpdateControllableEntitiesSenseStep();
00134       /* Call loop functions */
00135       m_cSimulator.GetLoopFunctions().PostStep();
00136       /* Flush logs */
00137       LOG.Flush();
00138       LOGERR.Flush();
00139    }
00140 
00141    /****************************************/
00142    /****************************************/
00143 
00144    void CSpace::AddControllableEntity(CControllableEntity& c_entity) {
00145       m_vecControllableEntities.push_back(&c_entity);
00146    }
00147 
00148    /****************************************/
00149    /****************************************/
00150 
00151    void CSpace::RemoveControllableEntity(CControllableEntity& c_entity) {
00152       CControllableEntity::TVector::iterator it = find(m_vecControllableEntities.begin(),
00153                                                        m_vecControllableEntities.end(),
00154                                                        &c_entity);
00155       if(it != m_vecControllableEntities.end()) {
00156          m_vecControllableEntities.erase(it);
00157       }
00158    }
00159       
00160    /****************************************/
00161    /****************************************/
00162 
00163    void CSpace::AddEntityToPhysicsEngine(CEmbodiedEntity& c_entity) {
00164       /* Get a reference to the root entity */
00165       CEntity* pcToAdd = &c_entity.GetRootEntity();
00166       /* Get a reference to the position of the entity */
00167       const CVector3& cPos = c_entity.GetOriginAnchor().Position;
00168       /* Go through engines and check which ones could house the entity */
00169       CPhysicsEngine::TVector vecPotentialEngines;
00170       for(size_t i = 0; i < m_ptPhysicsEngines->size(); ++i) {
00171          if((*m_ptPhysicsEngines)[i]->IsPointContained(cPos)) {
00172             vecPotentialEngines.push_back((*m_ptPhysicsEngines)[i]);
00173          }
00174       }
00175       /* If no engine can house the entity, bomb out */
00176       if(vecPotentialEngines.empty()) {
00177          THROW_ARGOSEXCEPTION("No physics engine can house entity \"" << pcToAdd->GetId() << "\".");
00178       }
00179       /* If the entity is not movable, add the entity to all the matching engines */
00180       if(! c_entity.IsMovable()) {
00181          bool bAdded = false;
00182          for(size_t i = 0; i < vecPotentialEngines.size(); ++i) {
00183             bAdded |= vecPotentialEngines[i]->AddEntity(*pcToAdd);
00184          }
00185          if(!bAdded) {
00186             THROW_ARGOSEXCEPTION("No physics engine can house entity \"" << pcToAdd->GetId() << "\".");
00187          }
00188       }
00189       /* If the entity is movable, only one engine can be associated to the embodied entity */
00190       else if(vecPotentialEngines.size() == 1) {
00191          /* Only one engine matches, bingo! */
00192          if(!vecPotentialEngines[0]->AddEntity(*pcToAdd)) {
00193             THROW_ARGOSEXCEPTION("No physics engine can house entity \"" << pcToAdd->GetId() << "\".");
00194          }
00195       }
00196       else {
00197          /* More than one engine matches, pick the first that can manage the entity */
00198          for(size_t i = 0; i < vecPotentialEngines.size(); ++i) {
00199             if(vecPotentialEngines[i]->AddEntity(*pcToAdd)) return;
00200          }
00201          /* No engine can house the entity */
00202          THROW_ARGOSEXCEPTION("No physics engine can house entity \"" << pcToAdd->GetId() << "\".");
00203       }
00204    }
00205       
00206    /****************************************/
00207    /****************************************/
00208 
00209    class RealNumberGenerator {
00210    public:
00211       virtual ~RealNumberGenerator() {}
00212       virtual CVector3 operator()(bool b_is_retry) = 0;
00213    };
00214 
00215    class ConstantGenerator : public RealNumberGenerator {
00216    public:
00217       ConstantGenerator(const CVector3& c_value) :
00218          m_cValue(c_value) {}
00219 
00220       inline virtual CVector3 operator()(bool b_is_retry) {
00221          return m_cValue;
00222       }
00223    private:
00224       CVector3 m_cValue;
00225 
00226    };
00227 
00228    class UniformGenerator : public RealNumberGenerator {
00229    public:
00230       UniformGenerator(const CVector3& c_min,
00231                        const CVector3& c_max) :
00232          m_cMin(c_min),
00233          m_cMax(c_max) {}
00234       inline virtual CVector3 operator()(bool b_is_retry) {
00235          Real fRandX =
00236             m_cMax.GetX() > m_cMin.GetX() ?
00237             CSimulator::GetInstance().GetRNG()->Uniform(CRange<Real>(m_cMin.GetX(), m_cMax.GetX())) :
00238             m_cMax.GetX();
00239          Real fRandY =
00240             m_cMax.GetY() > m_cMin.GetY() ?
00241             CSimulator::GetInstance().GetRNG()->Uniform(CRange<Real>(m_cMin.GetY(), m_cMax.GetY())) :
00242             m_cMax.GetY();
00243          Real fRandZ =
00244             m_cMax.GetZ() > m_cMin.GetZ() ?
00245             CSimulator::GetInstance().GetRNG()->Uniform(CRange<Real>(m_cMin.GetZ(), m_cMax.GetZ())) :
00246             m_cMax.GetZ();
00247          return CVector3(fRandX, fRandY, fRandZ);
00248       }
00249    private:
00250       CVector3 m_cMin;
00251       CVector3 m_cMax;
00252    };
00253 
00254    class GaussianGenerator : public RealNumberGenerator {
00255    public:
00256       GaussianGenerator(const CVector3& c_mean,
00257                         const CVector3& c_std_dev) :
00258          m_cMean(c_mean),
00259          m_cStdDev(c_std_dev) {}
00260       inline virtual CVector3 operator()(bool b_is_retry) {
00261          return CVector3(CSimulator::GetInstance().GetRNG()->Gaussian(m_cStdDev.GetX(), m_cMean.GetX()),
00262                          CSimulator::GetInstance().GetRNG()->Gaussian(m_cStdDev.GetY(), m_cMean.GetY()),
00263                          CSimulator::GetInstance().GetRNG()->Gaussian(m_cStdDev.GetZ(), m_cMean.GetZ()));
00264       }
00265    private:
00266       CVector3 m_cMean;
00267       CVector3 m_cStdDev;
00268    };
00269 
00270    class GridGenerator : public RealNumberGenerator {
00271    public:
00272       GridGenerator(const CVector3 c_center,
00273                     const UInt32 un_layout[],
00274                     const CVector3 c_distances):
00275          m_cCenter(c_center),
00276          m_cDistances(c_distances),
00277          m_unNumEntityPlaced(0) {
00278          m_unLayout[0] = un_layout[0];
00279          m_unLayout[1] = un_layout[1];
00280          m_unLayout[2] = un_layout[2];
00281          /* Check if layout is sane */
00282          if( m_unLayout[0] == 0 || m_unLayout[1] == 0 || m_unLayout[2] == 0 ) {
00283             THROW_ARGOSEXCEPTION("'layout' values (distribute position, method 'grid') must all be different than 0");
00284          }
00285       }
00286 
00287       virtual CVector3 operator()(bool b_is_retry) {
00288          if(b_is_retry) {
00289             THROW_ARGOSEXCEPTION("Impossible to place entity #" << m_unNumEntityPlaced << " in grid");
00290          }
00291          CVector3 cReturn;
00292          if(m_unNumEntityPlaced < m_unLayout[0] * m_unLayout[1] * m_unLayout[2]) {
00293             cReturn.SetX(m_cCenter.GetX() + ( m_unLayout[0] - 1 ) * m_cDistances.GetX() * 0.5 - ( m_unNumEntityPlaced  % m_unLayout[0] ) * m_cDistances.GetX());
00294             cReturn.SetY(m_cCenter.GetY() + ( m_unLayout[1] - 1 ) * m_cDistances.GetY() * 0.5 - ( m_unNumEntityPlaced  / m_unLayout[0] ) % m_unLayout[1] * m_cDistances.GetY());
00295             cReturn.SetZ(m_cCenter.GetZ() + ( m_unLayout[2] - 1 ) * m_cDistances.GetZ() * 0.5 - ( m_unNumEntityPlaced / ( m_unLayout[0] * m_unLayout[1] ) ) * m_cDistances.GetZ());
00296             ++m_unNumEntityPlaced;
00297          }
00298          else {
00299             THROW_ARGOSEXCEPTION("Distribute position, method 'grid': trying to place more entities than allowed "
00300                                  "by the 'layout', check your 'quantity' tag");
00301          }
00302          return cReturn;
00303       }
00304 
00305    private:
00306       CVector3 m_cCenter;
00307       UInt32 m_unLayout[3];
00308       CVector3 m_cDistances;
00309       UInt32 m_unNumEntityPlaced;
00310    };
00311 
00312    /****************************************/
00313    /****************************************/
00314 
00315    RealNumberGenerator* CreateGenerator(TConfigurationNode& t_tree) {
00316       std::string strMethod;
00317       GetNodeAttribute(t_tree, "method", strMethod);
00318       if(strMethod == "uniform") {
00319          CVector3 cMin, cMax;
00320          GetNodeAttribute(t_tree, "min", cMin);
00321          GetNodeAttribute(t_tree, "max", cMax);
00322          if(! (cMin <= cMax)) {
00323             THROW_ARGOSEXCEPTION("Uniform generator: the min is not less than or equal to max: " << cMin << " / " << cMax);
00324          }
00325          return new UniformGenerator(cMin, cMax);
00326       }
00327       else if(strMethod == "gaussian") {
00328          CVector3 cMean, cStdDev;
00329          GetNodeAttribute(t_tree, "mean", cMean);
00330          GetNodeAttribute(t_tree, "std_dev", cStdDev);
00331          return new GaussianGenerator(cMean, cStdDev);
00332       }
00333       else if(strMethod == "constant") {
00334          CVector3 cValues;
00335          GetNodeAttribute(t_tree, "values", cValues);
00336          return new ConstantGenerator(cValues);
00337       }
00338       else if(strMethod == "grid") {
00339          CVector3 cCenter,cDistances;
00340          GetNodeAttribute(t_tree, "center", cCenter);
00341          GetNodeAttribute(t_tree, "distances", cDistances);
00342          UInt32 unLayout[3];
00343          std::string strLayout;
00344          GetNodeAttribute(t_tree, "layout", strLayout);
00345          ParseValues<UInt32> (strLayout, 3, unLayout, ',');
00346          return new GridGenerator(cCenter, unLayout, cDistances);
00347       }
00348       else {
00349          THROW_ARGOSEXCEPTION("Unknown distribution method \"" << strMethod << "\"");
00350       }
00351    }
00352 
00353    /****************************************/
00354    /****************************************/
00355 
00356    static CEmbodiedEntity* GetEmbodiedEntity(CEntity* pc_entity) {
00357       /* Is the entity embodied itself? */
00358       CEmbodiedEntity* pcEmbodiedTest = dynamic_cast<CEmbodiedEntity*>(pc_entity);
00359       if(pcEmbodiedTest != NULL) {
00360          return pcEmbodiedTest;
00361       }
00362       /* Is the entity composable with an embodied component? */
00363       CComposableEntity* pcComposableTest = dynamic_cast<CComposableEntity*>(pc_entity);
00364       if(pcComposableTest != NULL) {
00365          if(pcComposableTest->HasComponent("body")) {
00366             return &(pcComposableTest->GetComponent<CEmbodiedEntity>("body"));
00367          }
00368       }
00369       /* No embodied entity found */
00370       return NULL;
00371    }
00372 
00373    /****************************************/
00374    /****************************************/
00375 
00376    static CPositionalEntity* GetPositionalEntity(CEntity* pc_entity) {
00377       /* Is the entity positional itself? */
00378       CPositionalEntity* pcPositionalTest = dynamic_cast<CPositionalEntity*>(pc_entity);
00379       if(pcPositionalTest != NULL) {
00380          return pcPositionalTest;
00381       }
00382       /* Is the entity composable with a positional component? */
00383       CComposableEntity* pcComposableTest = dynamic_cast<CComposableEntity*>(pc_entity);
00384       if(pcComposableTest != NULL) {
00385          if(pcComposableTest->HasComponent("position")) {
00386             return &(pcComposableTest->GetComponent<CPositionalEntity>("position"));
00387          }
00388       }
00389       /* No positional entity found */
00390       return NULL;
00391    }
00392 
00393    /****************************************/
00394    /****************************************/
00395 
00396    void CSpace::Distribute(TConfigurationNode& t_tree) {
00397       try {
00398          /* Get the needed nodes */
00399          TConfigurationNode cPositionNode;
00400          cPositionNode = GetNode(t_tree, "position");
00401          TConfigurationNode cOrientationNode;
00402          cOrientationNode = GetNode(t_tree, "orientation");
00403          TConfigurationNode cEntityNode;
00404          cEntityNode = GetNode(t_tree, "entity");
00405          /* Create the real number generators */
00406          RealNumberGenerator* pcPositionGenerator = CreateGenerator(cPositionNode);
00407          RealNumberGenerator* pcOrientationGenerator = CreateGenerator(cOrientationNode);
00408          /* How many entities? */
00409          UInt32 unQuantity;
00410          GetNodeAttribute(cEntityNode, "quantity", unQuantity);
00411          /* How many trials before failing? */
00412          UInt32 unMaxTrials;
00413          GetNodeAttribute(cEntityNode, "max_trials", unMaxTrials);
00414          /* Get the (optional) entity base numbering */
00415          UInt64 unBaseNum = 0;
00416          GetNodeAttributeOrDefault(cEntityNode, "base_num", unBaseNum, unBaseNum);
00417          /* Get the entity type to add (take only the first, ignore additional if any) */
00418          TConfigurationNodeIterator itEntity;
00419          itEntity = itEntity.begin(&cEntityNode);
00420          if(itEntity == itEntity.end()) {
00421             THROW_ARGOSEXCEPTION("No entity to distribute specified.");
00422          }
00423          /* Get the entity base ID */
00424          std::string strBaseId;
00425          GetNodeAttribute(*itEntity, "id", strBaseId);
00426          /* Add the requested entities */
00427          for(UInt32 i = 0; i < unQuantity; ++i) {
00428             /* Copy the entity XML tree */
00429             TConfigurationNode tEntityTree = *itEntity;
00430             /* Set progressive ID */
00431             SetNodeAttribute(tEntityTree, "id", strBaseId + ToString(i+unBaseNum));
00432             /* Go on until the entity is placed with no collisions or
00433                the max number of trials has been exceeded */
00434             UInt32 unTrials = 0;
00435             bool bDone = false;
00436             bool bRetry = false;
00437             CEntity* pcEntity;
00438             do {
00439                /* Create entity */
00440                pcEntity = CFactory<CEntity>::New(tEntityTree.Value());
00441                /* If the tree does not have a 'body' node, create a new one */
00442                if(!NodeExists(tEntityTree, "body")) {
00443                   TConfigurationNode tBodyNode("body");
00444                   AddChildNode(tEntityTree, tBodyNode);
00445                }
00446                /* Get 'body' node */
00447                TConfigurationNode& tBodyNode = GetNode(tEntityTree, "body");
00448                /* Set the position */
00449                SetNodeAttribute(tBodyNode, "position", (*pcPositionGenerator)(bRetry));
00450                /* Set the orientation */
00451                SetNodeAttribute(tBodyNode, "orientation", (*pcOrientationGenerator)(bRetry));
00452                /* Init the entity (this also creates the components, if pcEntity is a composable) */
00453                pcEntity->Init(tEntityTree);
00454                /*
00455                 * Now that you have the entity and its components, check whether the entity is positional or embodied
00456                 * or has one such component.
00457                 * In case the entity is positional but not embodied, there's no need to check for collisions
00458                 * In case the entity is embodied, we must check for collisions
00459                 * To check for collisions, we add the entity in the place where it's supposed to be,
00460                 * then we ask the engine if that entity is colliding with something
00461                 * In case of collision, we remove the entity and try a different position/orientation
00462                 */
00463                /* Check for embodied */
00464                CEmbodiedEntity* pcEmbodiedEntity = GetEmbodiedEntity(pcEntity);
00465                if(pcEmbodiedEntity == NULL) {
00466                   /* Check failed, then check for positional */
00467                   CPositionalEntity* pcPositionalEntity = GetPositionalEntity(pcEntity);
00468                   if(pcPositionalEntity == NULL) {
00469                      THROW_ARGOSEXCEPTION("Cannot distribute entities that are not positional nor embodied, and \"" << tEntityTree.Value() << "\" is neither.");
00470                   }
00471                   else {
00472                      /* Wherever we want to put the entity, it's OK, add it */
00473                      CallEntityOperation<CSpaceOperationAddEntity, CSpace, void>(*this, *pcEntity);
00474                   }
00475                }
00476                else {
00477                   /* The entity is embodied */
00478                   /* Add it to the space and to the designated physics engine */
00479                   CallEntityOperation<CSpaceOperationAddEntity, CSpace, void>(*this, *pcEntity);
00480                   /* Check if it's colliding with anything else */
00481                   if(pcEmbodiedEntity->IsCollidingWithSomething()) {
00482                      /* Set retry to true */
00483                      bRetry = true;
00484                      /* Get rid of the entity */
00485                      CallEntityOperation<CSpaceOperationRemoveEntity, CSpace, void>(*this, *pcEntity);
00486                      /* Increase the trial count */
00487                      ++unTrials;
00488                      /* Too many trials? */
00489                      if(unTrials > unMaxTrials) {
00490                         /* Yes, bomb out */
00491                         THROW_ARGOSEXCEPTION("Exceeded max trials when trying to distribute objects of type " <<
00492                                              tEntityTree.Value() << " with base id \"" <<
00493                                              strBaseId << "\". I managed to place only " << i << " objects.");
00494                      }
00495                      /* Retry with a new position */
00496                   }
00497                   else {
00498                      /* No collision, we're done with this entity */
00499                      bDone = true;
00500                   }
00501                }
00502             }
00503             while(!bDone);
00504          }
00505          /* Delete the generators, now unneeded */
00506          delete pcPositionGenerator;
00507          delete pcOrientationGenerator;
00508       }
00509       catch(CARGoSException& ex) {
00510          THROW_ARGOSEXCEPTION_NESTED("Error while trying to distribute entities", ex);
00511       }
00512    }
00513 
00514    /****************************************/
00515    /****************************************/
00516 
00517 }
```

---

Generated on 10 Jul 2018 for ARGoS by 
 1.6.1 
