# Supplementary material for: Iterative improvement in the automatic modular design of robot swarms
Source: PeerJ Comput Sci. 2020 Dec 7;6:e322. doi: 10.7717/peerj-cs.322 (PMC7924708; doi:10.7717/peerj-cs.322)
Supplement: Supplemental Information 3 [file peerj-cs-06-322-s003.zip › argos3/doc/api/standalone/a00338.html]

ARGoS: core/simulator/space/space.h File Reference


- Main Page
- Related Pages
- Namespaces
- Classes
- Files

- File List
- File Members

# core/simulator/space/space.h File Reference

`#include <argos3/core/utility/datatypes/any.h>`  
`#include <argos3/core/simulator/medium/medium.h>`  
`#include <argos3/core/simulator/space/positional_indices/positional_index.h>`  
`#include <argos3/core/simulator/entity/embodied_entity.h>`  
`#include <argos3/core/simulator/entity/controllable_entity.h>`  

Include dependency graph for space.h:

This graph shows which files directly or indirectly include this file:

Go to the source code of this file.

|  |  |
| --- | --- |
| Classes | |
| class | argos::CSpace |
| class | argos::CSpaceOperation< ACTION > |
| class | argos::CSpaceOperationAddEntity |
| class | argos::CSpaceOperationRemoveEntity |
| Namespaces | |
| namespace | argos |

|  |  |
| --- | --- |
|  | The namespace containing all the ARGoS related code. |

| Defines | |
| #define | SPACE\_OPERATION\_ADD\_ENTITY(ENTITY) |
| #define | SPACE\_OPERATION\_REMOVE\_ENTITY(ENTITY) |
| #define | REGISTER\_SPACE\_OPERATION(ACTION, OPERATION, ENTITY)   REGISTER\_ENTITY\_OPERATION(ACTION, CSpace, OPERATION, void, ENTITY); |
| #define | REGISTER\_STANDARD\_SPACE\_OPERATION\_ADD\_ENTITY(ENTITY) |
| #define | REGISTER\_STANDARD\_SPACE\_OPERATION\_REMOVE\_ENTITY(ENTITY) |
| #define | REGISTER\_STANDARD\_SPACE\_OPERATIONS\_ON\_ENTITY(ENTITY) |

---

## Define Documentation

|  |  |  |
| --- | --- | --- |
| #define REGISTER\_SPACE\_OPERATION | ( | ACTION, |
|  |  | OPERATION, |
|  |  | ENTITY |  | ) | REGISTER\_ENTITY\_OPERATION(ACTION, CSpace, OPERATION, void, ENTITY); |

Definition at line 489 of file space.h.

|  |  |  |  |  |  |
| --- | --- | --- | --- | --- | --- |
| #define REGISTER\_STANDARD\_SPACE\_OPERATION\_ADD\_ENTITY | ( | ENTITY |  | ) |  |

**Value:**

```
SPACE_OPERATION_ADD_ENTITY(ENTITY)                                   \
   REGISTER_SPACE_OPERATION(CSpaceOperationAddEntity,                   \
                            CSpaceOperationAdd ## ENTITY,               \
                            ENTITY);
```

Definition at line 492 of file space.h.

|  |  |  |  |  |  |
| --- | --- | --- | --- | --- | --- |
| #define REGISTER\_STANDARD\_SPACE\_OPERATION\_REMOVE\_ENTITY | ( | ENTITY |  | ) |  |

**Value:**

```
SPACE_OPERATION_REMOVE_ENTITY(ENTITY)                                \
   REGISTER_SPACE_OPERATION(CSpaceOperationRemoveEntity,                \
                            CSpaceOperationRemove ## ENTITY,            \
                            ENTITY);
```

Definition at line 498 of file space.h.

|  |  |  |  |  |  |
| --- | --- | --- | --- | --- | --- |
| #define REGISTER\_STANDARD\_SPACE\_OPERATIONS\_ON\_ENTITY | ( | ENTITY |  | ) |  |

**Value:**

```
REGISTER_STANDARD_SPACE_OPERATION_ADD_ENTITY(ENTITY)      \
   REGISTER_STANDARD_SPACE_OPERATION_REMOVE_ENTITY(ENTITY)
```

Definition at line 504 of file space.h.

|  |  |  |  |  |  |
| --- | --- | --- | --- | --- | --- |
| #define SPACE\_OPERATION\_ADD\_ENTITY | ( | ENTITY |  | ) |  |

**Value:**

```
class CSpaceOperationAdd ## ENTITY : public CSpaceOperationAddEntity {  \
   public:                                                                 \
      void ApplyTo(CSpace& c_space, ENTITY& c_entity) {                    \
         c_space.AddEntity(c_entity);                                      \
      }                                                                    \
   };
```

Definition at line 473 of file space.h.

|  |  |  |  |  |  |
| --- | --- | --- | --- | --- | --- |
| #define SPACE\_OPERATION\_REMOVE\_ENTITY | ( | ENTITY |  | ) |  |

**Value:**

```
class CSpaceOperationRemove ## ENTITY : public CSpaceOperationRemoveEntity { \
   public:                                                                      \
      void ApplyTo(CSpace& c_space, ENTITY& c_entity) {                         \
         c_space.RemoveEntity(c_entity);                                        \
      }                                                                         \
   };
```

Definition at line 481 of file space.h.

---

Generated on 10 Jul 2018 for ARGoS by 
 1.6.1 
