# Supplementary material for: Iterative improvement in the automatic modular design of robot swarms
Source: PeerJ Comput Sci. 2020 Dec 7;6:e322. doi: 10.7717/peerj-cs.322 (PMC7924708; doi:10.7717/peerj-cs.322)
Supplement: Supplemental Information 3 [file peerj-cs-06-322-s003.zip › argos3/doc/api/standalone/a00338_source.html]

ARGoS: core/simulator/space/space.h Source File


- Main Page
- Related Pages
- Namespaces
- Classes
- Files

- File List
- File Members

# core/simulator/space/space.h

Go to the documentation of this file.

```
00001 
00009 #ifndef SPACE_H
00010 #define SPACE_H
00011 
00012 namespace argos {
00013    class CSpace;
00014    class CRay3;
00015    class CFloorEntity;
00016    class CSimulator;
00017 }
00018 
00019 #include <argos3/core/utility/datatypes/any.h>
00020 #include <argos3/core/simulator/medium/medium.h>
00021 #include <argos3/core/simulator/space/positional_indices/positional_index.h>
00022 #include <argos3/core/simulator/entity/embodied_entity.h>
00023 #include <argos3/core/simulator/entity/controllable_entity.h>
00024 
00025 namespace argos {
00026 
00027    /****************************************/
00028    /****************************************/
00029 
00030    class CSpace : public CBaseConfigurableResource {
00031 
00032    public:
00033 
00054       typedef std::map <std::string, CAny, std::less <std::string> > TMapPerType;
00055 
00077       typedef std::map <std::string, TMapPerType, std::less <std::string> > TMapPerTypePerId;
00078 
00079       /****************************************/
00080       /****************************************/
00081 
00082    public:
00083 
00087       CSpace();
00088 
00092       virtual ~CSpace() {}
00093 
00098       virtual void Init(TConfigurationNode& t_tree);
00099 
00103       virtual void Reset();
00104 
00108       virtual void Destroy();
00109 
00113       inline UInt32 GetNumberEntities() const {
00114          return m_vecEntities.size();
00115       }
00116 
00123       inline CEntity::TVector& GetEntityVector() {
00124          return m_vecEntities;
00125       }
00126 
00137       inline CEntity::TVector& GetRootEntityVector() {
00138          return m_vecRootEntities;
00139       }
00140 
00147       inline CEntity& GetEntity(const std::string& str_id) {
00148          CEntity::TMap::const_iterator it = m_mapEntitiesPerId.find(str_id);
00149          if ( it != m_mapEntitiesPerId.end()) {
00150             return *(it->second);
00151          }
00152          THROW_ARGOSEXCEPTION("Unknown entity id \"" << str_id <<
00153                               "\" when requesting entity from space.");
00154       }
00155 
00164       void GetEntitiesMatching(CEntity::TVector& t_buffer,
00165                                const std::string& str_pattern);
00166 
00171       inline CEntity::TMap& GetEntityMapPerId() {
00172          return m_mapEntitiesPerId;
00173       }
00174 
00192       inline TMapPerTypePerId& GetEntityMapPerTypePerId() {
00193          return m_mapEntitiesPerTypePerId;
00194       }
00195 
00212       TMapPerType& GetEntitiesByType(const std::string& str_type);
00213 
00219       inline CFloorEntity& GetFloorEntity() {
00220          if(m_pcFloorEntity != NULL) return *m_pcFloorEntity;
00221          else THROW_ARGOSEXCEPTION("No floor entity has been added to the arena.");
00222       }
00223 
00228       inline void SetFloorEntity(CFloorEntity& c_floor_entity) {
00229          m_pcFloorEntity = &c_floor_entity;
00230       }
00231 
00246       virtual void Update();
00247 
00253       template <typename ENTITY>
00254       void AddEntity(ENTITY& c_entity) {
00255          std::string strEntityQualifiedName = c_entity.GetContext() + c_entity.GetId();
00256          /* Check that the id of the entity is not already present */
00257          if(m_mapEntitiesPerId.find(strEntityQualifiedName) != m_mapEntitiesPerId.end()) {
00258             THROW_ARGOSEXCEPTION("Error inserting a " <<
00259                                  c_entity.GetTypeDescription() <<
00260                                  " entity with id \"" <<
00261                                  strEntityQualifiedName <<
00262                                  "\". An entity with that id already exists.");
00263          }
00264          /* Add the entity to the indexes */
00265          if(!c_entity.HasParent()) {
00266             m_vecRootEntities.push_back(&c_entity);
00267          }
00268          m_vecEntities.push_back(&c_entity);
00269          m_mapEntitiesPerId[strEntityQualifiedName] = &c_entity;
00270          m_mapEntitiesPerTypePerId[c_entity.GetTypeDescription()][strEntityQualifiedName] = &c_entity;
00271       }
00272 
00278       template <typename ENTITY>
00279       void RemoveEntity(ENTITY& c_entity) {
00280          std::string strEntityQualifiedName = c_entity.GetContext() + c_entity.GetId();
00281          /* Search for entity in the index per type */
00282          TMapPerTypePerId::iterator itMapPerType = m_mapEntitiesPerTypePerId.find(c_entity.GetTypeDescription());
00283          if(itMapPerType != m_mapEntitiesPerTypePerId.end()) {
00284             /* Search for entity in the index per type per id */
00285             TMapPerType::iterator itMapPerTypePerId = itMapPerType->second.find(strEntityQualifiedName);
00286             if(itMapPerTypePerId != itMapPerType->second.end()) {
00287                /* Remove the entity from the indexes */
00288                CEntity::TVector::iterator itVec = find(m_vecEntities.begin(),
00289                                                        m_vecEntities.end(),
00290                                                        &c_entity);
00291                m_vecEntities.erase(itVec);
00292                CEntity::TMap::iterator itMap = m_mapEntitiesPerId.find(strEntityQualifiedName);
00293                itMapPerType->second.erase(itMapPerTypePerId);
00294                m_mapEntitiesPerId.erase(itMap);
00295                if(!c_entity.HasParent()) {
00296                   CEntity::TVector::iterator itRootVec = find(m_vecRootEntities.begin(),
00297                                                               m_vecRootEntities.end(),
00298                                                               &c_entity);
00299                   m_vecRootEntities.erase(itRootVec);
00300                }
00301                /* Remove entity object */
00302                c_entity.Destroy();
00303                delete &c_entity;
00304                return;
00305             }
00306          }
00307          THROW_ARGOSEXCEPTION("CSpace::RemoveEntity() : Entity \"" <<
00308                               strEntityQualifiedName <<
00309                               "\" has not been found in the indexes.");
00310       }
00311 
00317       inline UInt32 GetSimulationClock() const {
00318          return m_unSimulationClock;
00319       }
00320 
00326       inline void SetSimulationClock(UInt32 un_simulation_clock) {
00327          m_unSimulationClock = un_simulation_clock;
00328       }
00329 
00335       inline void IncreaseSimulationClock(UInt32 un_increase = 1) {
00336          m_unSimulationClock += un_increase;
00337       }
00338 
00343       inline const CVector3& GetArenaSize() const {
00344          return m_cArenaSize;
00345       }
00346 
00351       inline void SetArenaSize(const CVector3& c_size) {
00352          m_cArenaSize = c_size;
00353          m_cArenaLimits.Set(m_cArenaCenter - m_cArenaSize,
00354                             m_cArenaCenter + m_cArenaSize);
00355       }
00356 
00361       inline const CVector3& GetArenaCenter() const {
00362          return m_cArenaCenter;
00363       }
00364 
00369       inline void SetArenaCenter(const CVector3& c_center) {
00370          m_cArenaCenter = c_center;
00371          m_cArenaLimits.Set(m_cArenaCenter - m_cArenaSize,
00372                             m_cArenaCenter + m_cArenaSize);
00373       }
00374 
00375       /*
00376        * Returns the arena limits.
00377        * The arena limits are defined by <tt>arena center - arena size</tt> and
00378        * <tt>arena center - arena size</tt>.
00379        * @return the arena limits.
00380        */
00381       inline const CRange<CVector3>& GetArenaLimits() const {
00382          return m_cArenaLimits;
00383       }
00384 
00385       virtual void AddControllableEntity(CControllableEntity& c_entity);
00386       virtual void RemoveControllableEntity(CControllableEntity& c_entity);
00387       virtual void AddEntityToPhysicsEngine(CEmbodiedEntity& c_entity);
00388       
00389    protected:
00390 
00391       virtual void UpdateControllableEntitiesAct() = 0;
00392       virtual void UpdatePhysics() = 0;
00393       virtual void UpdateMedia() = 0;
00394       virtual void UpdateControllableEntitiesSenseStep() = 0;
00395 
00396       void Distribute(TConfigurationNode& t_tree);
00397 
00398       void AddBoxStrip(TConfigurationNode& t_tree);
00399 
00400    protected:
00401 
00402       friend class CSpaceOperationAddControllableEntity;
00403       friend class CSpaceOperationRemoveControllableEntity;
00404       friend class CSpaceOperationAddEmbodiedEntity;
00405 
00406    protected:
00407 
00408       /* The active simulator instance */
00409       CSimulator& m_cSimulator;
00410 
00412       UInt32 m_unSimulationClock;
00413 
00415       CVector3 m_cArenaCenter;
00416 
00418       CVector3 m_cArenaSize;
00419 
00421       CRange<CVector3> m_cArenaLimits;
00422 
00424       CEntity::TVector m_vecEntities;
00425 
00427       CEntity::TVector m_vecRootEntities;
00428 
00430       CEntity::TMap m_mapEntitiesPerId;
00431 
00435       TMapPerTypePerId m_mapEntitiesPerTypePerId;
00436 
00438       CControllableEntity::TVector m_vecControllableEntities;
00439 
00441       CFloorEntity* m_pcFloorEntity;
00442 
00444       CPhysicsEngine::TVector* m_ptPhysicsEngines;
00445 
00447       CMedium::TVector* m_ptMedia;
00448    };
00449 
00450    /****************************************/
00451    /****************************************/
00452 
00453    template <typename ACTION>
00454    class CSpaceOperation : public CEntityOperation<ACTION, CSpace, void> {
00455    public:
00456       virtual ~CSpaceOperation() {}
00457    };
00458 
00459    class CSpaceOperationAddEntity : public CSpaceOperation<CSpaceOperationAddEntity> {
00460    public:
00461       virtual ~CSpaceOperationAddEntity() {}
00462    };
00463    class CSpaceOperationRemoveEntity : public CSpaceOperation<CSpaceOperationRemoveEntity> {
00464    public:
00465       virtual ~CSpaceOperationRemoveEntity() {}
00466    };
00467 
00468 }
00469 
00470    /****************************************/
00471    /****************************************/
00472 
00473 #define SPACE_OPERATION_ADD_ENTITY(ENTITY)                                 \
00474    class CSpaceOperationAdd ## ENTITY : public CSpaceOperationAddEntity {  \
00475    public:                                                                 \
00476       void ApplyTo(CSpace& c_space, ENTITY& c_entity) {                    \
00477          c_space.AddEntity(c_entity);                                      \
00478       }                                                                    \
00479    };
00480 
00481 #define SPACE_OPERATION_REMOVE_ENTITY(ENTITY)                                   \
00482    class CSpaceOperationRemove ## ENTITY : public CSpaceOperationRemoveEntity { \
00483    public:                                                                      \
00484       void ApplyTo(CSpace& c_space, ENTITY& c_entity) {                         \
00485          c_space.RemoveEntity(c_entity);                                        \
00486       }                                                                         \
00487    };
00488 
00489 #define REGISTER_SPACE_OPERATION(ACTION, OPERATION, ENTITY)             \
00490    REGISTER_ENTITY_OPERATION(ACTION, CSpace, OPERATION, void, ENTITY);
00491 
00492 #define REGISTER_STANDARD_SPACE_OPERATION_ADD_ENTITY(ENTITY)            \
00493    SPACE_OPERATION_ADD_ENTITY(ENTITY)                                   \
00494    REGISTER_SPACE_OPERATION(CSpaceOperationAddEntity,                   \
00495                             CSpaceOperationAdd ## ENTITY,               \
00496                             ENTITY);
00497 
00498 #define REGISTER_STANDARD_SPACE_OPERATION_REMOVE_ENTITY(ENTITY)         \
00499    SPACE_OPERATION_REMOVE_ENTITY(ENTITY)                                \
00500    REGISTER_SPACE_OPERATION(CSpaceOperationRemoveEntity,                \
00501                             CSpaceOperationRemove ## ENTITY,            \
00502                             ENTITY);
00503 
00504 #define REGISTER_STANDARD_SPACE_OPERATIONS_ON_ENTITY(ENTITY) \
00505    REGISTER_STANDARD_SPACE_OPERATION_ADD_ENTITY(ENTITY)      \
00506    REGISTER_STANDARD_SPACE_OPERATION_REMOVE_ENTITY(ENTITY)
00507 
00508 #endif
```

---

Generated on 10 Jul 2018 for ARGoS by 
 1.6.1 
