# Supplementary material for: Iterative improvement in the automatic modular design of robot swarms
Source: PeerJ Comput Sci. 2020 Dec 7;6:e322. doi: 10.7717/peerj-cs.322 (PMC7924708; doi:10.7717/peerj-cs.322)
Supplement: Supplemental Information 3 [file peerj-cs-06-322-s003.zip › argos3/doc/api/standalone/a00339.html]

ARGoS: core/simulator/space/space\_multi\_thread\_balance\_length.cpp File Reference


- Main Page
- Related Pages
- Namespaces
- Classes
- Files

- File List
- File Members

# core/simulator/space/space\_multi\_thread\_balance\_length.cpp File Reference

`#include "space_multi_thread_balance_length.h"`  
`#include <argos3/core/simulator/simulator.h>`  
`#include <argos3/core/utility/profiler/profiler.h>`  

Include dependency graph for space\_multi\_thread\_balance\_length.cpp:

Go to the source code of this file.

|  |  |
| --- | --- |
| Classes | |
| struct | argos::SCleanupThreadData |
| Namespaces | |
| namespace | argos |

|  |  |
| --- | --- |
|  | The namespace containing all the ARGoS related code. |

| Defines | |
| #define | MAIN\_START\_PHASE(PHASE) |
| #define | MAIN\_WAIT\_FOR\_END\_OF(PHASE) |
| #define | THREAD\_WAIT\_FOR\_START\_OF(PHASE) |
| #define | THREAD\_PERFORM\_TASK(PHASE, TASKVEC, SNIPPET) |
| Functions | |
| void \* | argos::LaunchThreadBalanceLength (void \*p\_data) |

---

## Define Documentation

|  |  |  |  |  |  |
| --- | --- | --- | --- | --- | --- |
| #define MAIN\_START\_PHASE | ( | PHASE |  | ) |  |

**Value:**

```
pthread_mutex_lock(&m_tStart ## PHASE ## PhaseMutex);    \
   m_un ## PHASE ## PhaseIdleCounter = 0;                   \
   m_unTaskIndex = 0;                                       \
   pthread_cond_broadcast(&m_tStart ## PHASE ## PhaseCond); \
   pthread_mutex_unlock(&m_tStart ## PHASE ## PhaseMutex);
```

Definition at line 156 of file space\_multi\_thread\_balance\_length.cpp.

|  |  |  |  |  |  |
| --- | --- | --- | --- | --- | --- |
| #define MAIN\_WAIT\_FOR\_END\_OF | ( | PHASE |  | ) |  |

**Value:**

```
pthread_mutex_lock(&m_tStart ## PHASE ## PhaseMutex);                                    \
   while(m_un ## PHASE ## PhaseIdleCounter < CSimulator::GetInstance().GetNumThreads()) {   \
      pthread_cond_wait(&m_tStart ## PHASE ## PhaseCond, &m_tStart ## PHASE ## PhaseMutex); \
   }                                                                                        \
   pthread_mutex_unlock(&m_tStart ## PHASE ## PhaseMutex);
```

Definition at line 163 of file space\_multi\_thread\_balance\_length.cpp.

|  |  |  |
| --- | --- | --- |
| #define THREAD\_PERFORM\_TASK | ( | PHASE, |
|  |  | TASKVEC, |
|  |  | SNIPPET |  | ) |  |

**Value:**

```
while(1) {                                                       \
      pthread_mutex_lock(&m_tFetchTaskMutex);                       \
      if(m_unTaskIndex < (TASKVEC).size()) {                        \
         unTaskIndex = m_unTaskIndex;                               \
         ++m_unTaskIndex;                                           \
         pthread_mutex_unlock(&m_tFetchTaskMutex);                  \
         pthread_testcancel();                                      \
         {                                                          \
            SNIPPET;                                                \
         }                                                          \
         pthread_testcancel();                                      \
      }                                                             \
      else {                                                        \
         pthread_mutex_unlock(&m_tFetchTaskMutex);                  \
         pthread_testcancel();                                      \
         pthread_mutex_lock(&m_tStart ## PHASE ## PhaseMutex);      \
         ++m_un ## PHASE ## PhaseIdleCounter;                       \
         pthread_cond_broadcast(&m_tStart ## PHASE ## PhaseCond);   \
         pthread_mutex_unlock(&m_tStart ## PHASE ## PhaseMutex);    \
         pthread_testcancel();                                      \
         break;                                                     \
      }                                                             \
   }                                                                \
   pthread_testcancel();
```

Definition at line 241 of file space\_multi\_thread\_balance\_length.cpp.

|  |  |  |  |  |  |
| --- | --- | --- | --- | --- | --- |
| #define THREAD\_WAIT\_FOR\_START\_OF | ( | PHASE |  | ) |  |

**Value:**

```
pthread_mutex_lock(&m_tStart ## PHASE ## PhaseMutex);                                    \
   while(m_un ## PHASE ## PhaseIdleCounter == CSimulator::GetInstance().GetNumThreads()) {  \
      pthread_cond_wait(&m_tStart ## PHASE ## PhaseCond, &m_tStart ## PHASE ## PhaseMutex); \
   }                                                                                        \
   pthread_mutex_unlock(&m_tStart ## PHASE ## PhaseMutex);                                  \
   pthread_testcancel();
```

Definition at line 233 of file space\_multi\_thread\_balance\_length.cpp.

---

Generated on 10 Jul 2018 for ARGoS by 
 1.6.1 
