# Supplementary material for: Iterative improvement in the automatic modular design of robot swarms
Source: PeerJ Comput Sci. 2020 Dec 7;6:e322. doi: 10.7717/peerj-cs.322 (PMC7924708; doi:10.7717/peerj-cs.322)
Supplement: Supplemental Information 3 [file peerj-cs-06-322-s003.zip › argos3/doc/api/standalone/a00339_source.html]

ARGoS: core/simulator/space/space\_multi\_thread\_balance\_length.cpp Source File


- Main Page
- Related Pages
- Namespaces
- Classes
- Files

- File List
- File Members

# core/simulator/space/space\_multi\_thread\_balance\_length.cpp

Go to the documentation of this file.

```
00001 
00007 #include "space_multi_thread_balance_length.h"
00008 #include <argos3/core/simulator/simulator.h>
00009 #include <argos3/core/utility/profiler/profiler.h>
00010 
00011 namespace argos {
00012 
00013    /****************************************/
00014    /****************************************/
00015 
00016    struct SCleanupThreadData {
00017       pthread_mutex_t* StartSenseControlPhaseMutex;
00018       pthread_mutex_t* StartActPhaseMutex;
00019       pthread_mutex_t* StartPhysicsPhaseMutex;
00020       pthread_mutex_t* StartMediaPhaseMutex;
00021       pthread_mutex_t* FetchTaskMutex;
00022    };
00023 
00024    static void CleanupThread(void* p_data) {
00025       CSimulator& cSimulator = CSimulator::GetInstance();
00026       if(cSimulator.IsProfiling()) {
00027          cSimulator.GetProfiler().CollectThreadResourceUsage();
00028       }
00029       SCleanupThreadData& sData =
00030          *reinterpret_cast<SCleanupThreadData*>(p_data);
00031       pthread_mutex_unlock(sData.FetchTaskMutex);
00032       pthread_mutex_unlock(sData.StartSenseControlPhaseMutex);
00033       pthread_mutex_unlock(sData.StartActPhaseMutex);
00034       pthread_mutex_unlock(sData.StartPhysicsPhaseMutex);
00035       pthread_mutex_unlock(sData.StartMediaPhaseMutex);
00036    }
00037 
00038    void* LaunchThreadBalanceLength(void* p_data) {
00039       /* Set up thread-safe buffers for this new thread */
00040       LOG.AddThreadSafeBuffer();
00041       LOGERR.AddThreadSafeBuffer();
00042       /* Make this thread cancellable */
00043       pthread_setcancelstate(PTHREAD_CANCEL_ENABLE, NULL);
00044       pthread_setcanceltype(PTHREAD_CANCEL_DEFERRED, NULL);
00045       /* Get a handle to the thread launch data */
00046       CSpaceMultiThreadBalanceLength::SThreadLaunchData* psData = reinterpret_cast<CSpaceMultiThreadBalanceLength::SThreadLaunchData*>(p_data);
00047       /* Create cancellation data */
00048       SCleanupThreadData sCancelData;
00049       sCancelData.StartSenseControlPhaseMutex = &(psData->Space->m_tStartSenseControlPhaseMutex);
00050       sCancelData.StartActPhaseMutex = &(psData->Space->m_tStartActPhaseMutex);
00051       sCancelData.StartPhysicsPhaseMutex = &(psData->Space->m_tStartPhysicsPhaseMutex);
00052       sCancelData.StartMediaPhaseMutex = &(psData->Space->m_tStartMediaPhaseMutex);
00053       sCancelData.FetchTaskMutex = &(psData->Space->m_tFetchTaskMutex);
00054       pthread_cleanup_push(CleanupThread, &sCancelData);
00055       psData->Space->SlaveThread();
00056       /* Dispose of cancellation data */
00057       pthread_cleanup_pop(1);
00058       return NULL;
00059    }
00060 
00061    /****************************************/
00062    /****************************************/
00063 
00064    void CSpaceMultiThreadBalanceLength::Init(TConfigurationNode& t_tree) {
00065       /* Initialize the space */
00066       CSpace::Init(t_tree);
00067       /* Initialize thread related structures */
00068       int nErrors;
00069       /* Init mutexes */
00070       if((nErrors = pthread_mutex_init(&m_tStartSenseControlPhaseMutex, NULL)) ||
00071          (nErrors = pthread_mutex_init(&m_tStartActPhaseMutex, NULL)) ||
00072          (nErrors = pthread_mutex_init(&m_tStartPhysicsPhaseMutex, NULL)) ||
00073          (nErrors = pthread_mutex_init(&m_tStartMediaPhaseMutex, NULL)) ||
00074          (nErrors = pthread_mutex_init(&m_tFetchTaskMutex, NULL))) {
00075          THROW_ARGOSEXCEPTION("Error creating thread mutexes " << ::strerror(nErrors));
00076       }
00077       /* Init conditionals */
00078       if((nErrors = pthread_cond_init(&m_tStartSenseControlPhaseCond, NULL)) ||
00079          (nErrors = pthread_cond_init(&m_tStartActPhaseCond, NULL)) ||
00080          (nErrors = pthread_cond_init(&m_tStartPhysicsPhaseCond, NULL)) ||
00081          (nErrors = pthread_cond_init(&m_tStartMediaPhaseCond, NULL)) ||
00082          (nErrors = pthread_cond_init(&m_tFetchTaskCond, NULL))) {
00083          THROW_ARGOSEXCEPTION("Error creating thread conditionals " << ::strerror(nErrors));
00084       }
00085       /* Reset the idle thread count */
00086       m_unSenseControlPhaseIdleCounter = CSimulator::GetInstance().GetNumThreads();
00087       m_unActPhaseIdleCounter = CSimulator::GetInstance().GetNumThreads();
00088       m_unPhysicsPhaseIdleCounter = CSimulator::GetInstance().GetNumThreads();
00089       m_unMediaPhaseIdleCounter = CSimulator::GetInstance().GetNumThreads();
00090       /* Start threads */
00091       StartThreads();
00092    }
00093 
00094    /****************************************/
00095    /****************************************/
00096 
00097    void CSpaceMultiThreadBalanceLength::Destroy() {
00098       /* Destroy the threads to update the controllable entities */
00099       int nErrors;
00100       if(m_ptThreads != NULL) {
00101          for(UInt32 i = 0; i < CSimulator::GetInstance().GetNumThreads(); ++i) {
00102             if((nErrors = pthread_cancel(m_ptThreads[i]))) {
00103                THROW_ARGOSEXCEPTION("Error canceling threads " << ::strerror(nErrors));
00104             }
00105          }
00106          void** ppJoinResult = new void*[CSimulator::GetInstance().GetNumThreads()];
00107          for(UInt32 i = 0; i < CSimulator::GetInstance().GetNumThreads(); ++i) {
00108             if((nErrors = pthread_join(m_ptThreads[i], ppJoinResult + i))) {
00109                THROW_ARGOSEXCEPTION("Error joining threads " << ::strerror(nErrors));
00110             }
00111             if(ppJoinResult[i] != PTHREAD_CANCELED) {
00112                LOGERR << "[WARNING] Thread #" << i<< " not canceled" << std::endl;
00113             }
00114          }
00115          delete[] ppJoinResult;
00116       }
00117       delete[] m_ptThreads;
00118       /* Destroy the thread launch info */
00119       if(m_psThreadData != NULL) {
00120          for(UInt32 i = 0; i < CSimulator::GetInstance().GetNumThreads(); ++i) {
00121             delete m_psThreadData[i];
00122          }
00123       }
00124       delete[] m_psThreadData;
00125       pthread_mutex_destroy(&m_tStartSenseControlPhaseMutex);
00126       pthread_mutex_destroy(&m_tStartActPhaseMutex);
00127       pthread_mutex_destroy(&m_tStartPhysicsPhaseMutex);
00128       pthread_mutex_destroy(&m_tStartMediaPhaseMutex);
00129       pthread_mutex_destroy(&m_tFetchTaskMutex);
00130       pthread_cond_destroy(&m_tStartSenseControlPhaseCond);
00131       pthread_cond_destroy(&m_tStartActPhaseCond);
00132       pthread_cond_destroy(&m_tStartPhysicsPhaseCond);
00133       pthread_cond_destroy(&m_tStartMediaPhaseCond);
00134       pthread_cond_destroy(&m_tFetchTaskCond);
00135 
00136       /* Destroy the base space */
00137       CSpace::Destroy();
00138    }
00139 
00140    /****************************************/
00141    /****************************************/
00142 
00143    void CSpaceMultiThreadBalanceLength::Update() {
00144       /* Reset the idle thread count */
00145       m_unSenseControlPhaseIdleCounter = CSimulator::GetInstance().GetNumThreads();
00146       m_unActPhaseIdleCounter = CSimulator::GetInstance().GetNumThreads();
00147       m_unPhysicsPhaseIdleCounter = CSimulator::GetInstance().GetNumThreads();
00148       m_unMediaPhaseIdleCounter = CSimulator::GetInstance().GetNumThreads();
00149       /* Update the space */
00150       CSpace::Update();
00151    }
00152 
00153    /****************************************/
00154    /****************************************/
00155 
00156 #define MAIN_START_PHASE(PHASE)                             \
00157    pthread_mutex_lock(&m_tStart ## PHASE ## PhaseMutex);    \
00158    m_un ## PHASE ## PhaseIdleCounter = 0;                   \
00159    m_unTaskIndex = 0;                                       \
00160    pthread_cond_broadcast(&m_tStart ## PHASE ## PhaseCond); \
00161    pthread_mutex_unlock(&m_tStart ## PHASE ## PhaseMutex);
00162 
00163 #define MAIN_WAIT_FOR_END_OF(PHASE)                                                         \
00164    pthread_mutex_lock(&m_tStart ## PHASE ## PhaseMutex);                                    \
00165    while(m_un ## PHASE ## PhaseIdleCounter < CSimulator::GetInstance().GetNumThreads()) {   \
00166       pthread_cond_wait(&m_tStart ## PHASE ## PhaseCond, &m_tStart ## PHASE ## PhaseMutex); \
00167    }                                                                                        \
00168    pthread_mutex_unlock(&m_tStart ## PHASE ## PhaseMutex);
00169 
00170    void CSpaceMultiThreadBalanceLength::UpdateControllableEntitiesAct() {
00171       /* Act phase */
00172       MAIN_START_PHASE(Act);
00173       MAIN_WAIT_FOR_END_OF(Act);
00174    }
00175 
00176    /****************************************/
00177    /****************************************/
00178 
00179    void CSpaceMultiThreadBalanceLength::UpdatePhysics() {
00180       /* Physics phase */
00181       MAIN_START_PHASE(Physics);
00182       MAIN_WAIT_FOR_END_OF(Physics);
00183       /* Perform entity transfer from engine to engine, if needed */
00184       for(size_t i = 0; i < m_ptPhysicsEngines->size(); ++i) {
00185          if((*m_ptPhysicsEngines)[i]->IsEntityTransferNeeded()) {
00186             (*m_ptPhysicsEngines)[i]->TransferEntities();
00187          }
00188       }
00189    }
00190 
00191    /****************************************/
00192    /****************************************/
00193 
00194    void CSpaceMultiThreadBalanceLength::UpdateMedia() {
00195       /* Media phase */
00196       MAIN_START_PHASE(Media);
00197       MAIN_WAIT_FOR_END_OF(Media);
00198    }
00199 
00200    /****************************************/
00201    /****************************************/
00202 
00203    void CSpaceMultiThreadBalanceLength::UpdateControllableEntitiesSenseStep() {
00204       /* Sense/control phase */
00205       MAIN_START_PHASE(SenseControl);
00206       MAIN_WAIT_FOR_END_OF(SenseControl);
00207    }
00208 
00209    /****************************************/
00210    /****************************************/
00211 
00212    void CSpaceMultiThreadBalanceLength::StartThreads() {
00213       int nErrors;
00214       /* Create the threads to update the controllable entities */
00215       m_ptThreads = new pthread_t[CSimulator::GetInstance().GetNumThreads()];
00216       m_psThreadData = new SThreadLaunchData*[CSimulator::GetInstance().GetNumThreads()];
00217       for(UInt32 i = 0; i < CSimulator::GetInstance().GetNumThreads(); ++i) {
00218          /* Create the struct with the info to launch the thread */
00219          m_psThreadData[i] = new SThreadLaunchData(i, this);
00220          /* Create the thread */
00221          if((nErrors = pthread_create(m_ptThreads + i,
00222                                       NULL,
00223                                       LaunchThreadBalanceLength,
00224                                       reinterpret_cast<void*>(m_psThreadData[i])))) {
00225             THROW_ARGOSEXCEPTION("Error creating thread: " << ::strerror(nErrors));
00226          }
00227       }
00228    }
00229 
00230    /****************************************/
00231    /****************************************/
00232 
00233 #define THREAD_WAIT_FOR_START_OF(PHASE)                                                     \
00234    pthread_mutex_lock(&m_tStart ## PHASE ## PhaseMutex);                                    \
00235    while(m_un ## PHASE ## PhaseIdleCounter == CSimulator::GetInstance().GetNumThreads()) {  \
00236       pthread_cond_wait(&m_tStart ## PHASE ## PhaseCond, &m_tStart ## PHASE ## PhaseMutex); \
00237    }                                                                                        \
00238    pthread_mutex_unlock(&m_tStart ## PHASE ## PhaseMutex);                                  \
00239    pthread_testcancel();
00240 
00241 #define THREAD_PERFORM_TASK(PHASE, TASKVEC, SNIPPET)                \
00242    while(1) {                                                       \
00243       pthread_mutex_lock(&m_tFetchTaskMutex);                       \
00244       if(m_unTaskIndex < (TASKVEC).size()) {                        \
00245          unTaskIndex = m_unTaskIndex;                               \
00246          ++m_unTaskIndex;                                           \
00247          pthread_mutex_unlock(&m_tFetchTaskMutex);                  \
00248          pthread_testcancel();                                      \
00249          {                                                          \
00250             SNIPPET;                                                \
00251          }                                                          \
00252          pthread_testcancel();                                      \
00253       }                                                             \
00254       else {                                                        \
00255          pthread_mutex_unlock(&m_tFetchTaskMutex);                  \
00256          pthread_testcancel();                                      \
00257          pthread_mutex_lock(&m_tStart ## PHASE ## PhaseMutex);      \
00258          ++m_un ## PHASE ## PhaseIdleCounter;                       \
00259          pthread_cond_broadcast(&m_tStart ## PHASE ## PhaseCond);   \
00260          pthread_mutex_unlock(&m_tStart ## PHASE ## PhaseMutex);    \
00261          pthread_testcancel();                                      \
00262          break;                                                     \
00263       }                                                             \
00264    }                                                                \
00265    pthread_testcancel();
00266 
00267    void CSpaceMultiThreadBalanceLength::SlaveThread() {
00268       /* Task index */
00269       size_t unTaskIndex;
00270       while(1) {
00271          THREAD_WAIT_FOR_START_OF(Act);
00272          THREAD_PERFORM_TASK(
00273             Act,
00274             m_vecControllableEntities,
00275             m_vecControllableEntities[unTaskIndex]->Act();
00276             );
00277          THREAD_WAIT_FOR_START_OF(Physics);
00278          THREAD_PERFORM_TASK(
00279             Physics,
00280             *m_ptPhysicsEngines,
00281             (*m_ptPhysicsEngines)[unTaskIndex]->Update();
00282             );
00283          THREAD_WAIT_FOR_START_OF(Media);
00284          THREAD_PERFORM_TASK(
00285             Media,
00286             *m_ptMedia,
00287             (*m_ptMedia)[unTaskIndex]->Update();
00288             );
00289          THREAD_WAIT_FOR_START_OF(SenseControl);
00290          THREAD_PERFORM_TASK(
00291             SenseControl,
00292             m_vecControllableEntities,
00293             m_vecControllableEntities[unTaskIndex]->Sense();
00294             m_vecControllableEntities[unTaskIndex]->ControlStep();
00295             );
00296       }
00297    }
00298 
00299    /****************************************/
00300    /****************************************/
00301 
00302 }
```

---

Generated on 10 Jul 2018 for ARGoS by 
 1.6.1 
