# Supplementary material for: Iterative improvement in the automatic modular design of robot swarms
Source: PeerJ Comput Sci. 2020 Dec 7;6:e322. doi: 10.7717/peerj-cs.322 (PMC7924708; doi:10.7717/peerj-cs.322)
Supplement: Supplemental Information 3 [file peerj-cs-06-322-s003.zip › argos3/doc/api/standalone/a00340_source.html]

ARGoS: core/simulator/space/space\_multi\_thread\_balance\_length.h Source File


- Main Page
- Related Pages
- Namespaces
- Classes
- Files

- File List
- File Members

# core/simulator/space/space\_multi\_thread\_balance\_length.h

Go to the documentation of this file.

```
00001 
00011 #ifndef SPACE_MULTI_THREAD_BALANCE_LENGTH_H
00012 #define SPACE_MULTI_THREAD_BALANCE_LENGTH_H
00013 
00014 namespace argos {
00015    class CSpace;
00016 }
00017 
00018 #include <argos3/core/simulator/space/space.h>
00019 
00020 namespace argos {
00021 
00022    class CSpaceMultiThreadBalanceLength : public CSpace {
00023 
00024    public:
00025 
00026       CSpaceMultiThreadBalanceLength() {}
00027       virtual ~CSpaceMultiThreadBalanceLength() {}
00028 
00029       virtual void Init(TConfigurationNode& t_tree);
00030       virtual void Destroy();
00031 
00032       virtual void Update();
00033       virtual void UpdateControllableEntitiesAct();
00034       virtual void UpdatePhysics();
00035       virtual void UpdateMedia();
00036       virtual void UpdateControllableEntitiesSenseStep();
00037 
00038    private:
00039 
00040       void StartThreads();
00041       void SlaveThread();
00042       friend void* LaunchThreadBalanceLength(void* p_data);
00043 
00044    private:
00045 
00047       struct SThreadLaunchData {
00048          UInt32 ThreadId;
00049          CSpaceMultiThreadBalanceLength* Space;
00050          
00051          SThreadLaunchData(UInt32 un_thread_id,
00052                            CSpaceMultiThreadBalanceLength* pc_space) :
00053             ThreadId(un_thread_id),
00054             Space(pc_space) {}
00055       };
00056 
00058       pthread_t* m_ptThreads;
00059 
00061       SThreadLaunchData** m_psThreadData;
00062 
00064       size_t m_unTaskIndex;
00065 
00067       pthread_mutex_t m_tStartSenseControlPhaseMutex;
00069       pthread_mutex_t m_tStartActPhaseMutex;
00071       pthread_mutex_t m_tStartPhysicsPhaseMutex;
00073       pthread_mutex_t m_tStartMediaPhaseMutex;
00075       pthread_mutex_t m_tFetchTaskMutex;
00076 
00078       pthread_cond_t m_tStartSenseControlPhaseCond;
00080       pthread_cond_t m_tStartActPhaseCond;
00082       pthread_cond_t m_tStartPhysicsPhaseCond;
00084       pthread_cond_t m_tStartMediaPhaseCond;
00086       pthread_cond_t m_tFetchTaskCond;
00087 
00089       UInt32 m_unSenseControlPhaseIdleCounter;
00091       UInt32 m_unActPhaseIdleCounter;
00093       UInt32 m_unPhysicsPhaseIdleCounter;
00095       UInt32 m_unMediaPhaseIdleCounter;
00096 
00097    };
00098 
00099 }
00100 
00101 #endif
```

---

Generated on 10 Jul 2018 for ARGoS by 
 1.6.1 
