# Supplementary material for: Iterative improvement in the automatic modular design of robot swarms
Source: PeerJ Comput Sci. 2020 Dec 7;6:e322. doi: 10.7717/peerj-cs.322 (PMC7924708; doi:10.7717/peerj-cs.322)
Supplement: Supplemental Information 3 [file peerj-cs-06-322-s003.zip › argos3/doc/api/standalone/a00341.html]

ARGoS: core/simulator/space/space\_multi\_thread\_balance\_quantity.cpp File Reference


- Main Page
- Related Pages
- Namespaces
- Classes
- Files

- File List
- File Members

# core/simulator/space/space\_multi\_thread\_balance\_quantity.cpp File Reference

`#include <unistd.h>`  
`#include <cstring>`  
`#include <argos3/core/simulator/simulator.h>`  
`#include <argos3/core/utility/profiler/profiler.h>`  
`#include "space_multi_thread_balance_quantity.h"`  

Include dependency graph for space\_multi\_thread\_balance\_quantity.cpp:

Go to the source code of this file.

|  |  |
| --- | --- |
| Classes | |
| struct | argos::SCleanupUpdateThreadData |
| Namespaces | |
| namespace | argos |

|  |  |
| --- | --- |
|  | The namespace containing all the ARGoS related code. |

| Defines | |
| #define | MAIN\_SEND\_GO\_FOR\_PHASE(PHASE) |
| #define | MAIN\_WAIT\_FOR\_PHASE\_END(PHASE) |
| #define | THREAD\_WAIT\_FOR\_GO\_SIGNAL(PHASE) |
| #define | THREAD\_SIGNAL\_PHASE\_DONE(PHASE) |
| Functions | |
| void \* | argos::LaunchUpdateThreadBalanceQuantity (void \*p\_data) |
| CRange< size\_t > | argos::CalculatePluginRangeForThread (size\_t un\_id, size\_t un\_tot\_plugins) |

---

## Define Documentation

|  |  |  |  |  |  |
| --- | --- | --- | --- | --- | --- |
| #define MAIN\_SEND\_GO\_FOR\_PHASE | ( | PHASE |  | ) |  |

**Value:**

```
LOG.Flush();                                             \
   LOGERR.Flush();                                          \
   pthread_mutex_lock(&m_t ## PHASE ## ConditionalMutex);   \
   m_un ## PHASE ## PhaseDoneCounter = 0;                   \
   pthread_cond_broadcast(&m_t ## PHASE ## Conditional);    \
   pthread_mutex_unlock(&m_t ## PHASE ## ConditionalMutex);
```

Definition at line 168 of file space\_multi\_thread\_balance\_quantity.cpp.

|  |  |  |  |  |  |
| --- | --- | --- | --- | --- | --- |
| #define MAIN\_WAIT\_FOR\_PHASE\_END | ( | PHASE |  | ) |  |

**Value:**

```
pthread_mutex_lock(&m_t ## PHASE ## ConditionalMutex);               \
   while(m_un ## PHASE ## PhaseDoneCounter < CSimulator::GetInstance().GetNumThreads()) { \
      pthread_cond_wait(&m_t ## PHASE ## Conditional, &m_t ## PHASE ## ConditionalMutex); \
   }                                                                    \
   pthread_mutex_unlock(&m_t ## PHASE ## ConditionalMutex);
```

Definition at line 176 of file space\_multi\_thread\_balance\_quantity.cpp.

|  |  |  |  |  |  |
| --- | --- | --- | --- | --- | --- |
| #define THREAD\_SIGNAL\_PHASE\_DONE | ( | PHASE |  | ) |  |

**Value:**

```
pthread_mutex_lock(&m_t ## PHASE ## ConditionalMutex);   \
   ++m_un ## PHASE ## PhaseDoneCounter;                     \
   pthread_cond_broadcast(&m_t ## PHASE ## Conditional);    \
   pthread_mutex_unlock(&m_t ## PHASE ## ConditionalMutex); \
   pthread_testcancel();
```

Definition at line 235 of file space\_multi\_thread\_balance\_quantity.cpp.

|  |  |  |  |  |  |
| --- | --- | --- | --- | --- | --- |
| #define THREAD\_WAIT\_FOR\_GO\_SIGNAL | ( | PHASE |  | ) |  |

**Value:**

```
pthread_mutex_lock(&m_t ## PHASE ## ConditionalMutex);                                  \
   while(m_un ## PHASE ## PhaseDoneCounter == CSimulator::GetInstance().GetNumThreads()) { \
      pthread_cond_wait(&m_t ## PHASE ## Conditional, &m_t ## PHASE ## ConditionalMutex);  \
   }                                                                                       \
   pthread_mutex_unlock(&m_t ## PHASE ## ConditionalMutex);                                \
   pthread_testcancel();
```

Definition at line 227 of file space\_multi\_thread\_balance\_quantity.cpp.

---

Generated on 10 Jul 2018 for ARGoS by 
 1.6.1 
