# Supplementary material for: Iterative improvement in the automatic modular design of robot swarms
Source: PeerJ Comput Sci. 2020 Dec 7;6:e322. doi: 10.7717/peerj-cs.322 (PMC7924708; doi:10.7717/peerj-cs.322)
Supplement: Supplemental Information 3 [file peerj-cs-06-322-s003.zip › argos3/doc/api/standalone/a00341_source.html]

ARGoS: core/simulator/space/space\_multi\_thread\_balance\_quantity.cpp Source File


- Main Page
- Related Pages
- Namespaces
- Classes
- Files

- File List
- File Members

# core/simulator/space/space\_multi\_thread\_balance\_quantity.cpp

Go to the documentation of this file.

```
00001 
00007 #include <unistd.h>
00008 #include <cstring>
00009 #include <argos3/core/simulator/simulator.h>
00010 #include <argos3/core/utility/profiler/profiler.h>
00011 #include "space_multi_thread_balance_quantity.h"
00012 
00013 namespace argos {
00014 
00015    /****************************************/
00016    /****************************************/
00017 
00018    struct SCleanupUpdateThreadData {
00019       pthread_mutex_t* SenseControlStepConditionalMutex;
00020       pthread_mutex_t* ActConditionalMutex;
00021       pthread_mutex_t* PhysicsConditionalMutex;
00022       pthread_mutex_t* MediaConditionalMutex;
00023    };
00024 
00025    static void CleanupUpdateThread(void* p_data) {
00026       CSimulator& cSimulator = CSimulator::GetInstance();
00027       if(cSimulator.IsProfiling()) {
00028          cSimulator.GetProfiler().CollectThreadResourceUsage();
00029       }
00030       SCleanupUpdateThreadData& sData =
00031          *reinterpret_cast<SCleanupUpdateThreadData*>(p_data);
00032       pthread_mutex_unlock(sData.SenseControlStepConditionalMutex);
00033       pthread_mutex_unlock(sData.ActConditionalMutex);
00034       pthread_mutex_unlock(sData.PhysicsConditionalMutex);
00035       pthread_mutex_unlock(sData.MediaConditionalMutex);
00036    }
00037 
00038    void* LaunchUpdateThreadBalanceQuantity(void* p_data) {
00039       LOG.AddThreadSafeBuffer();
00040       LOGERR.AddThreadSafeBuffer();
00041       CSpaceMultiThreadBalanceQuantity::SUpdateThreadData* psData = reinterpret_cast<CSpaceMultiThreadBalanceQuantity::SUpdateThreadData*>(p_data);
00042       psData->Space->UpdateThread(psData->ThreadId);
00043       return NULL;
00044    }
00045 
00046    /****************************************/
00047    /****************************************/
00048 
00049    CSpaceMultiThreadBalanceQuantity::CSpaceMultiThreadBalanceQuantity() :
00050       m_psUpdateThreadData(NULL),
00051       m_ptUpdateThreads(NULL),
00052       m_bIsControllableEntityAssignmentRecalculationNeeded(true) {}
00053 
00054    /****************************************/
00055    /****************************************/
00056 
00057    void CSpaceMultiThreadBalanceQuantity::Init(TConfigurationNode& t_tree) {
00058       /* Initialize the space */
00059       CSpace::Init(t_tree);
00060       /* Initialize thread related structures */
00061       int nErrors;
00062       /* First the counters */
00063       m_unSenseControlStepPhaseDoneCounter = CSimulator::GetInstance().GetNumThreads();
00064       m_unActPhaseDoneCounter = CSimulator::GetInstance().GetNumThreads();
00065       m_unPhysicsPhaseDoneCounter = CSimulator::GetInstance().GetNumThreads();
00066       m_unMediaPhaseDoneCounter = CSimulator::GetInstance().GetNumThreads();
00067       /* Then the mutexes */
00068       if((nErrors = pthread_mutex_init(&m_tSenseControlStepConditionalMutex, NULL)) ||
00069          (nErrors = pthread_mutex_init(&m_tActConditionalMutex, NULL)) ||
00070          (nErrors = pthread_mutex_init(&m_tPhysicsConditionalMutex, NULL)) ||
00071          (nErrors = pthread_mutex_init(&m_tMediaConditionalMutex, NULL))) {
00072          THROW_ARGOSEXCEPTION("Error creating thread mutexes " << ::strerror(nErrors));
00073       }
00074       /* Finally the conditionals */
00075       if((nErrors = pthread_cond_init(&m_tSenseControlStepConditional, NULL)) ||
00076          (nErrors = pthread_cond_init(&m_tActConditional, NULL)) ||
00077          (nErrors = pthread_cond_init(&m_tPhysicsConditional, NULL)) ||
00078          (nErrors = pthread_cond_init(&m_tMediaConditional, NULL))) {
00079          THROW_ARGOSEXCEPTION("Error creating thread conditionals " << ::strerror(nErrors));
00080       }
00081       /* Start threads */
00082       StartThreads();
00083    }
00084 
00085    /****************************************/
00086    /****************************************/
00087 
00088    void CSpaceMultiThreadBalanceQuantity::StartThreads() {
00089       int nErrors;
00090       /* Create the threads to update the controllable entities */
00091       m_ptUpdateThreads = new pthread_t[CSimulator::GetInstance().GetNumThreads()];
00092       m_psUpdateThreadData = new SUpdateThreadData*[CSimulator::GetInstance().GetNumThreads()];
00093       for(UInt32 i = 0; i < CSimulator::GetInstance().GetNumThreads(); ++i) {
00094          /* Create the struct with the info to launch the thread */
00095          m_psUpdateThreadData[i] = new SUpdateThreadData(i, this);
00096          /* Create the thread */
00097          if((nErrors = pthread_create(m_ptUpdateThreads + i,
00098                                       NULL,
00099                                       LaunchUpdateThreadBalanceQuantity,
00100                                       reinterpret_cast<void*>(m_psUpdateThreadData[i])))) {
00101             THROW_ARGOSEXCEPTION("Error creating thread: " << ::strerror(nErrors));
00102          }
00103       }
00104    }
00105 
00106    /****************************************/
00107    /****************************************/
00108 
00109    void CSpaceMultiThreadBalanceQuantity::Destroy() {
00110       /* Destroy the threads to update the controllable entities */
00111       int nErrors;
00112       if(m_ptUpdateThreads != NULL) {
00113          for(UInt32 i = 0; i < CSimulator::GetInstance().GetNumThreads(); ++i) {
00114             if((nErrors = pthread_cancel(m_ptUpdateThreads[i]))) {
00115                THROW_ARGOSEXCEPTION("Error canceling controllable entities update threads " << ::strerror(nErrors));
00116             }
00117          }
00118          void** ppJoinResult = new void*[CSimulator::GetInstance().GetNumThreads()];
00119          for(UInt32 i = 0; i < CSimulator::GetInstance().GetNumThreads(); ++i) {
00120             if((nErrors = pthread_join(m_ptUpdateThreads[i], ppJoinResult + i))) {
00121                THROW_ARGOSEXCEPTION("Error joining controllable entities update threads " << ::strerror(nErrors));
00122             }
00123             if(ppJoinResult[i] != PTHREAD_CANCELED) {
00124                LOGERR << "[WARNING] Controllable entities update thread #" << i<< " not canceled" << std::endl;
00125             }
00126          }
00127          delete[] ppJoinResult;
00128       }
00129       delete[] m_ptUpdateThreads;
00130       /* Destroy the thread launch info */
00131       if(m_psUpdateThreadData != NULL) {
00132          for(UInt32 i = 0; i < CSimulator::GetInstance().GetNumThreads(); ++i) {
00133             delete m_psUpdateThreadData[i];
00134          }
00135       }
00136       delete[] m_psUpdateThreadData;
00137       pthread_mutex_destroy(&m_tSenseControlStepConditionalMutex);
00138       pthread_mutex_destroy(&m_tActConditionalMutex);
00139       pthread_mutex_destroy(&m_tPhysicsConditionalMutex);
00140       pthread_mutex_destroy(&m_tMediaConditionalMutex);
00141       pthread_cond_destroy(&m_tSenseControlStepConditional);
00142       pthread_cond_destroy(&m_tActConditional);
00143       pthread_cond_destroy(&m_tPhysicsConditional);
00144       pthread_cond_destroy(&m_tMediaConditional);
00145       /* Destroy the base space */
00146       CSpace::Destroy();
00147    }
00148 
00149    /****************************************/
00150    /****************************************/
00151    
00152    void CSpaceMultiThreadBalanceQuantity::AddControllableEntity(CControllableEntity& c_entity) {
00153       m_bIsControllableEntityAssignmentRecalculationNeeded = true;
00154       CSpace::AddControllableEntity(c_entity);
00155    }
00156 
00157    /****************************************/
00158    /****************************************/
00159    
00160    void CSpaceMultiThreadBalanceQuantity::RemoveControllableEntity(CControllableEntity& c_entity) {
00161       m_bIsControllableEntityAssignmentRecalculationNeeded = true;
00162       CSpace::RemoveControllableEntity(c_entity);
00163    }
00164 
00165    /****************************************/
00166    /****************************************/
00167    
00168 #define MAIN_SEND_GO_FOR_PHASE(PHASE)                       \
00169    LOG.Flush();                                             \
00170    LOGERR.Flush();                                          \
00171    pthread_mutex_lock(&m_t ## PHASE ## ConditionalMutex);   \
00172    m_un ## PHASE ## PhaseDoneCounter = 0;                   \
00173    pthread_cond_broadcast(&m_t ## PHASE ## Conditional);    \
00174    pthread_mutex_unlock(&m_t ## PHASE ## ConditionalMutex);
00175 
00176 #define MAIN_WAIT_FOR_PHASE_END(PHASE)                                  \
00177    pthread_mutex_lock(&m_t ## PHASE ## ConditionalMutex);               \
00178    while(m_un ## PHASE ## PhaseDoneCounter < CSimulator::GetInstance().GetNumThreads()) { \
00179       pthread_cond_wait(&m_t ## PHASE ## Conditional, &m_t ## PHASE ## ConditionalMutex); \
00180    }                                                                    \
00181    pthread_mutex_unlock(&m_t ## PHASE ## ConditionalMutex);
00182    
00183    void CSpaceMultiThreadBalanceQuantity::UpdateControllableEntitiesAct() {
00184       MAIN_SEND_GO_FOR_PHASE(Act);
00185       MAIN_WAIT_FOR_PHASE_END(Act);
00186       /* Avoid recalculation at the next time step */
00187       m_bIsControllableEntityAssignmentRecalculationNeeded = false;
00188    }
00189 
00190    /****************************************/
00191    /****************************************/
00192 
00193    void CSpaceMultiThreadBalanceQuantity::UpdatePhysics() {
00194       /* Update the physics engines */
00195       MAIN_SEND_GO_FOR_PHASE(Physics);
00196       MAIN_WAIT_FOR_PHASE_END(Physics);
00197       /* Perform entity transfer from engine to engine, if needed */
00198       for(size_t i = 0; i < m_ptPhysicsEngines->size(); ++i) {
00199          if((*m_ptPhysicsEngines)[i]->IsEntityTransferNeeded()) {
00200             (*m_ptPhysicsEngines)[i]->TransferEntities();
00201          }
00202       }
00203    }
00204 
00205    /****************************************/
00206    /****************************************/
00207 
00208    void CSpaceMultiThreadBalanceQuantity::UpdateMedia() {
00209       /* Update the media */
00210       MAIN_SEND_GO_FOR_PHASE(Media);
00211       MAIN_WAIT_FOR_PHASE_END(Media);
00212    }
00213 
00214    /****************************************/
00215    /****************************************/
00216 
00217    void CSpaceMultiThreadBalanceQuantity::UpdateControllableEntitiesSenseStep() {
00218       MAIN_SEND_GO_FOR_PHASE(SenseControlStep);
00219       MAIN_WAIT_FOR_PHASE_END(SenseControlStep);
00220       /* Avoid recalculation at the next time step */
00221       m_bIsControllableEntityAssignmentRecalculationNeeded = false;
00222    }
00223 
00224    /****************************************/
00225    /****************************************/
00226 
00227 #define THREAD_WAIT_FOR_GO_SIGNAL(PHASE)                                                   \
00228    pthread_mutex_lock(&m_t ## PHASE ## ConditionalMutex);                                  \
00229    while(m_un ## PHASE ## PhaseDoneCounter == CSimulator::GetInstance().GetNumThreads()) { \
00230       pthread_cond_wait(&m_t ## PHASE ## Conditional, &m_t ## PHASE ## ConditionalMutex);  \
00231    }                                                                                       \
00232    pthread_mutex_unlock(&m_t ## PHASE ## ConditionalMutex);                                \
00233    pthread_testcancel();
00234    
00235 #define THREAD_SIGNAL_PHASE_DONE(PHASE)                     \
00236    pthread_mutex_lock(&m_t ## PHASE ## ConditionalMutex);   \
00237    ++m_un ## PHASE ## PhaseDoneCounter;                     \
00238    pthread_cond_broadcast(&m_t ## PHASE ## Conditional);    \
00239    pthread_mutex_unlock(&m_t ## PHASE ## ConditionalMutex); \
00240    pthread_testcancel();
00241 
00242    CRange<size_t> CalculatePluginRangeForThread(size_t un_id,
00243                                                 size_t un_tot_plugins) {
00244       /* This is the minimum number of plugins assigned to a thread */
00245       size_t unMinPortion = un_tot_plugins / CSimulator::GetInstance().GetNumThreads();
00246       /* If the division has a remainder, the extra plugins must be assigned too */
00247       size_t unExtraPortion = un_tot_plugins % CSimulator::GetInstance().GetNumThreads();
00248       /* Calculate the range */
00249       if(unMinPortion == 0) {
00250          /* Not all threads get a plugin */
00251          if(un_id < unExtraPortion) {
00252             /* This thread does */
00253             return CRange<size_t>(un_id, un_id+1);
00254          }
00255          else {
00256             /* This thread does not */
00257             return CRange<size_t>();
00258          }
00259       }
00260       else {
00261          /* For sure this thread will get unMinPortion plugins, does it get an extra too? */
00262          if(un_id < unExtraPortion) {
00263             /* Yes, it gets an extra */
00264             return CRange<size_t>( un_id    * (unMinPortion+1),
00265                                   (un_id+1) * (unMinPortion+1));
00266          }
00267          else {
00268             /* No, it doesn't get an extra */
00269             return CRange<size_t>(unExtraPortion * (unMinPortion+1) + (un_id-unExtraPortion)   * unMinPortion,
00270                                   unExtraPortion * (unMinPortion+1) + (un_id-unExtraPortion+1) * unMinPortion);
00271          }
00272       }
00273    }
00274 
00275    void CSpaceMultiThreadBalanceQuantity::UpdateThread(UInt32 un_id) {
00276       /* Copy the id */
00277       UInt32 unId = un_id;
00278       /* Create cancellation data */
00279       SCleanupUpdateThreadData sCancelData;
00280       sCancelData.SenseControlStepConditionalMutex = &m_tSenseControlStepConditionalMutex;
00281       sCancelData.ActConditionalMutex = &m_tActConditionalMutex;
00282       sCancelData.PhysicsConditionalMutex = &m_tPhysicsConditionalMutex;
00283       sCancelData.MediaConditionalMutex = &m_tMediaConditionalMutex;
00284       pthread_cleanup_push(CleanupUpdateThread, &sCancelData);
00285       /* Id range for the physics engines assigned to this thread */
00286       CRange<size_t> cPhysicsRange = CalculatePluginRangeForThread(unId, m_ptPhysicsEngines->size());
00287       /* Id range for the physics engines assigned to this thread */
00288       CRange<size_t> cMediaRange = CalculatePluginRangeForThread(unId, m_ptMedia->size());
00289       /* Variables storing the portion of entities to update */
00290       CRange<size_t> cEntityRange;
00291       while(1) {
00292          THREAD_WAIT_FOR_GO_SIGNAL(Act);
00293          /* Calculate the portion of entities to update, if needed */
00294          if(m_bIsControllableEntityAssignmentRecalculationNeeded) {
00295             cEntityRange = CalculatePluginRangeForThread(unId, m_vecControllableEntities.size());
00296          }
00297          /* Cope with the fact that there may be less entities than threads */
00298          if(cEntityRange.GetSpan() > 0) {
00299             /* This thread has entities */
00300             /* Actuate control choices */
00301             for(size_t i = cEntityRange.GetMin(); i < cEntityRange.GetMax(); ++i) {
00302                m_vecControllableEntities[i]->Act();
00303             }
00304             pthread_testcancel();
00305             THREAD_SIGNAL_PHASE_DONE(Act);
00306          }
00307          else {
00308             /* This thread has no entities -> dummy computation */
00309             THREAD_SIGNAL_PHASE_DONE(Act);
00310          }
00311          /* Update physics engines, if this thread has been assigned to them */
00312          THREAD_WAIT_FOR_GO_SIGNAL(Physics);
00313          if(cPhysicsRange.GetSpan() > 0) {
00314             /* This thread has engines, update them */
00315             for(size_t i = cPhysicsRange.GetMin(); i < cPhysicsRange.GetMax(); ++i) {
00316                (*m_ptPhysicsEngines)[i]->Update();
00317             }
00318             pthread_testcancel();
00319             THREAD_SIGNAL_PHASE_DONE(Physics);
00320          }
00321          else {
00322             /* This thread has no engines -> dummy computation */
00323             THREAD_SIGNAL_PHASE_DONE(Physics);
00324          }
00325          /* Update media, if this thread has been assigned to them */
00326          THREAD_WAIT_FOR_GO_SIGNAL(Media);
00327          if(cMediaRange.GetSpan() > 0) {
00328             /* This thread has media, update them */
00329             for(size_t i = cMediaRange.GetMin(); i < cMediaRange.GetMax(); ++i) {
00330                (*m_ptMedia)[i]->Update();
00331             }
00332             pthread_testcancel();
00333             THREAD_SIGNAL_PHASE_DONE(Media);
00334          }
00335          else {
00336             /* This thread has no media -> dummy computation */
00337             THREAD_SIGNAL_PHASE_DONE(Media);
00338          }
00339          /* Update sensor readings and call controllers */
00340          THREAD_WAIT_FOR_GO_SIGNAL(SenseControlStep);
00341          /* Cope with the fact that there may be less entities than threads */
00342          if(cEntityRange.GetSpan() > 0) {
00343             /* This thread has entities */
00344             for(size_t i = cEntityRange.GetMin(); i < cEntityRange.GetMax(); ++i) {
00345                m_vecControllableEntities[i]->Sense();
00346                m_vecControllableEntities[i]->ControlStep();
00347             }
00348             pthread_testcancel();
00349             THREAD_SIGNAL_PHASE_DONE(SenseControlStep);
00350          }
00351          else {
00352             /* This thread has no entities -> dummy computation */
00353             THREAD_SIGNAL_PHASE_DONE(SenseControlStep);
00354          }
00355       }
00356       pthread_cleanup_pop(1);
00357    }
00358 
00359    /****************************************/
00360    /****************************************/
00361 
00362 }
```

---

Generated on 10 Jul 2018 for ARGoS by 
 1.6.1 
