# Supplementary material for: Iterative improvement in the automatic modular design of robot swarms
Source: PeerJ Comput Sci. 2020 Dec 7;6:e322. doi: 10.7717/peerj-cs.322 (PMC7924708; doi:10.7717/peerj-cs.322)
Supplement: Supplemental Information 3 [file peerj-cs-06-322-s003.zip › argos3/doc/api/standalone/a00342_source.html]

ARGoS: core/simulator/space/space\_multi\_thread\_balance\_quantity.h Source File


- Main Page
- Related Pages
- Namespaces
- Classes
- Files

- File List
- File Members

# core/simulator/space/space\_multi\_thread\_balance\_quantity.h

Go to the documentation of this file.

```
00001 
00007 #ifndef SPACE_MULTI_THREAD_BALANCE_QUANTITY_H
00008 #define SPACE_MULTI_THREAD_BALANCE_QUANTITY_H
00009 
00010 #include <argos3/core/simulator/space/space.h>
00011 #include <pthread.h>
00012 
00013 namespace argos {
00014 
00015    class CSpaceMultiThreadBalanceQuantity : public CSpace {
00016 
00017       /****************************************/
00018       /****************************************/
00019       
00020    private:
00021       
00022       struct SUpdateThreadData {
00023          UInt32 ThreadId;
00024          CSpaceMultiThreadBalanceQuantity* Space;
00025          
00026          SUpdateThreadData(UInt32 un_thread_id,
00027                            CSpaceMultiThreadBalanceQuantity* pc_space) :
00028             ThreadId(un_thread_id),
00029             Space(pc_space) {}
00030       };
00031 
00032       /****************************************/
00033       /****************************************/
00034 
00035    private:
00036 
00038       SUpdateThreadData** m_psUpdateThreadData;
00039 
00041       pthread_t* m_ptUpdateThreads;
00042 
00044       UInt32 m_unSenseControlStepPhaseDoneCounter;
00045       UInt32 m_unActPhaseDoneCounter;
00046       UInt32 m_unPhysicsPhaseDoneCounter;
00047       UInt32 m_unMediaPhaseDoneCounter;
00048 
00050       pthread_mutex_t m_tSenseControlStepConditionalMutex;
00051       pthread_mutex_t m_tActConditionalMutex;
00052       pthread_mutex_t m_tPhysicsConditionalMutex;
00053       pthread_mutex_t m_tMediaConditionalMutex;
00054 
00056       pthread_cond_t m_tSenseControlStepConditional;
00057       pthread_cond_t m_tActConditional;
00058       pthread_cond_t m_tPhysicsConditional;
00059       pthread_cond_t m_tMediaConditional;
00060 
00063       bool m_bIsControllableEntityAssignmentRecalculationNeeded;
00064 
00065    public:
00066 
00067       CSpaceMultiThreadBalanceQuantity();
00068       virtual ~CSpaceMultiThreadBalanceQuantity() {}
00069 
00070       virtual void Init(TConfigurationNode& t_tree);
00071       virtual void Destroy();
00072 
00073       virtual void UpdateControllableEntitiesAct();
00074       virtual void UpdatePhysics();
00075       virtual void UpdateMedia();
00076       virtual void UpdateControllableEntitiesSenseStep();
00077 
00078    protected:
00079 
00080       virtual void AddControllableEntity(CControllableEntity& c_entity);
00081       virtual void RemoveControllableEntity(CControllableEntity& c_entity);
00082 
00083    private:
00084 
00085       void StartThreads();
00086       void UpdateThread(UInt32 un_id);
00087       friend void* LaunchUpdateThreadBalanceQuantity(void* p_data);
00088 
00089    };
00090 
00091 }
00092 
00093 #endif
```

---

Generated on 10 Jul 2018 for ARGoS by 
 1.6.1 
