# Supplementary material for: Iterative improvement in the automatic modular design of robot swarms
Source: PeerJ Comput Sci. 2020 Dec 7;6:e322. doi: 10.7717/peerj-cs.322 (PMC7924708; doi:10.7717/peerj-cs.322)
Supplement: Supplemental Information 3 [file peerj-cs-06-322-s003.zip › argos3/doc/api/standalone/a00343_source.html]

ARGoS: core/simulator/space/space\_no\_threads.cpp Source File


- Main Page
- Related Pages
- Namespaces
- Classes
- Files

- File List
- File Members

# core/simulator/space/space\_no\_threads.cpp

Go to the documentation of this file.

```
00001 
00011 #include "space_no_threads.h"
00012 #include <argos3/core/simulator/simulator.h>
00013 
00014 namespace argos {
00015 
00016    /****************************************/
00017    /****************************************/
00018 
00019    void CSpaceNoThreads::UpdateControllableEntitiesAct() {
00020       for(size_t i = 0; i < m_vecControllableEntities.size(); ++i) {
00021          m_vecControllableEntities[i]->Act();
00022       }
00023    }
00024 
00025    /****************************************/
00026    /****************************************/
00027 
00028    void CSpaceNoThreads::UpdatePhysics() {
00029       /* Update the physics engines */
00030       for(size_t i = 0; i < m_ptPhysicsEngines->size(); ++i) {
00031          (*m_ptPhysicsEngines)[i]->Update();
00032       }
00033       /* Perform entity transfer from engine to engine, if needed */
00034       for(size_t i = 0; i < m_ptPhysicsEngines->size(); ++i) {
00035          if((*m_ptPhysicsEngines)[i]->IsEntityTransferNeeded()) {
00036             (*m_ptPhysicsEngines)[i]->TransferEntities();
00037          }
00038       }
00039    }
00040 
00041    /****************************************/
00042    /****************************************/
00043 
00044    void CSpaceNoThreads::UpdateMedia() {
00045       for(size_t i = 0; i < m_ptMedia->size(); ++i) {
00046          (*m_ptMedia)[i]->Update();
00047       }
00048    }
00049 
00050    /****************************************/
00051    /****************************************/
00052 
00053    void CSpaceNoThreads::UpdateControllableEntitiesSenseStep() {
00054       for(size_t i = 0; i < m_vecControllableEntities.size(); ++i) {
00055          m_vecControllableEntities[i]->Sense();
00056          m_vecControllableEntities[i]->ControlStep();
00057       }
00058    }
00059 
00060    /****************************************/
00061    /****************************************/
00062 
00063 }
```

---

Generated on 10 Jul 2018 for ARGoS by 
 1.6.1 
