# Supplementary material for: Iterative improvement in the automatic modular design of robot swarms
Source: PeerJ Comput Sci. 2020 Dec 7;6:e322. doi: 10.7717/peerj-cs.322 (PMC7924708; doi:10.7717/peerj-cs.322)
Supplement: Supplemental Information 3 [file peerj-cs-06-322-s003.zip › argos3/doc/api/standalone/a00344_source.html]

ARGoS: core/simulator/space/space\_no\_threads.h Source File


- Main Page
- Related Pages
- Namespaces
- Classes
- Files

- File List
- File Members

# core/simulator/space/space\_no\_threads.h

Go to the documentation of this file.

```
00001 
00011 #ifndef SPACE_NO_THREADS_H
00012 #define SPACE_NO_THREADS_H
00013 
00014 namespace argos {
00015    class CSpace;
00016 }
00017 
00018 #include <argos3/core/simulator/space/space.h>
00019 
00020 namespace argos {
00021 
00022    class CSpaceNoThreads : public CSpace {
00023 
00024    public:
00025 
00026       CSpaceNoThreads() {}
00027       virtual ~CSpaceNoThreads() {}
00028 
00029       virtual void UpdateControllableEntitiesAct();
00030       virtual void UpdatePhysics();
00031       virtual void UpdateMedia();
00032       virtual void UpdateControllableEntitiesSenseStep();
00033 
00034    };
00035 
00036 }
00037 
00038 #endif
```

---

Generated on 10 Jul 2018 for ARGoS by 
 1.6.1 
