# Supplementary material for: Iterative improvement in the automatic modular design of robot swarms
Source: PeerJ Comput Sci. 2020 Dec 7;6:e322. doi: 10.7717/peerj-cs.322 (PMC7924708; doi:10.7717/peerj-cs.322)
Supplement: Supplemental Information 3 [file peerj-cs-06-322-s003.zip › argos3/doc/api/standalone/a00345_source.html]

ARGoS: core/simulator/visualization/default\_visualization.cpp Source File


- Main Page
- Related Pages
- Namespaces
- Classes
- Files

- File List
- File Members

# core/simulator/visualization/default\_visualization.cpp

Go to the documentation of this file.

```
00001 
00007 #include <argos3/core/simulator/visualization/default_visualization.h>
00008 #include <argos3/core/simulator/space/space.h>
00009 #include <argos3/core/simulator/loop_functions.h>
00010 
00011 #include <unistd.h>
00012 
00013 namespace argos {
00014 
00015    /****************************************/
00016    /****************************************/
00017 
00018    static Real TVTimeToHumanReadable(::timeval& t_time) {
00019       return
00020          static_cast<Real>(t_time.tv_sec) +
00021          static_cast<Real>(t_time.tv_usec * 10e-6);
00022    }
00023 
00024    /****************************************/
00025    /****************************************/
00026 
00027    CDefaultVisualization::CDefaultVisualization() {
00028       /* Set the pointer to the step function */
00029       if(m_cSimulator.IsRealTimeClock()) {
00030          /* Use real-time clock and set time structures */
00031          m_tStepFunction = &CDefaultVisualization::RealTimeStep;
00032          timerclear(&m_tStepClockTime);
00033          m_tStepClockTime.tv_usec = 1e6 * CPhysicsEngine::GetSimulationClockTick();
00034          ::gettimeofday(&m_tStepStartTime, NULL);
00035       }
00036       else {
00037          /* Use normal clock */
00038          m_tStepFunction = &CDefaultVisualization::NormalStep;
00039       }
00040    }
00041 
00042    /****************************************/
00043    /****************************************/
00044 
00045    void CDefaultVisualization::Execute() {
00046       /* Main cycle */
00047       while(!m_cSimulator.IsExperimentFinished()) {
00048          (this->*m_tStepFunction)();
00049       }
00050       /* The experiment is finished */
00051       m_cSimulator.GetLoopFunctions().PostExperiment();
00052       LOG.Flush();
00053       LOGERR.Flush();
00054    }
00055 
00056    /****************************************/
00057    /****************************************/
00058 
00059    void CDefaultVisualization::NormalStep() {
00060       m_cSimulator.UpdateSpace();
00061    }
00062 
00063    /****************************************/
00064    /****************************************/
00065 
00066    void CDefaultVisualization::RealTimeStep() {
00067       /* m_tStepStartTime has already been set */
00068       m_cSimulator.UpdateSpace();
00069       /* Take the time now */
00070       ::gettimeofday(&m_tStepEndTime, NULL);
00071       /* Calculate the elapsed time */
00072       timersub(&m_tStepEndTime, &m_tStepStartTime, &m_tStepElapsedTime);
00073       /* If the elapsed time is lower than the tick length, wait */
00074       if(!timercmp(&m_tStepElapsedTime, &m_tStepClockTime, >)) {
00075          /* Calculate the waiting time */
00076          timersub(&m_tStepClockTime, &m_tStepElapsedTime, &m_tStepWaitTime);
00077          /* Wait */
00078          ::usleep(m_tStepWaitTime.tv_sec * 1e6 + m_tStepWaitTime.tv_usec);
00079          /* Get the new step end */
00080          ::gettimeofday(&m_tStepEndTime, NULL);
00081       }
00082       else {
00083          LOGERR << "[WARNING] Clock tick took "
00084                 << TVTimeToHumanReadable(m_tStepElapsedTime)
00085                 << " sec, more than the expected "
00086                 << TVTimeToHumanReadable(m_tStepClockTime)
00087                 << " sec."
00088                 << std::endl;
00089       }
00090       /* Set the step start time to whatever the step end time is */
00091       m_tStepStartTime.tv_sec = m_tStepEndTime.tv_sec;
00092       m_tStepStartTime.tv_usec = m_tStepEndTime.tv_usec;
00093    }
00094 
00095    /****************************************/
00096    /****************************************/
00097 
00098 }
```

---

Generated on 10 Jul 2018 for ARGoS by 
 1.6.1 
