# Supplementary material for: Iterative improvement in the automatic modular design of robot swarms
Source: PeerJ Comput Sci. 2020 Dec 7;6:e322. doi: 10.7717/peerj-cs.322 (PMC7924708; doi:10.7717/peerj-cs.322)
Supplement: Supplemental Information 3 [file peerj-cs-06-322-s003.zip › argos3/doc/api/standalone/a00346_source.html]

ARGoS: core/simulator/visualization/default\_visualization.h Source File


- Main Page
- Related Pages
- Namespaces
- Classes
- Files

- File List
- File Members

# core/simulator/visualization/default\_visualization.h

Go to the documentation of this file.

```
00001 
00009 #ifndef DEFAULT_VISUALIZATION_H
00010 #define DEFAULT_VISUALIZATION_H
00011 
00012 namespace argos {
00013    class CDefaultVisualization;
00014 }
00015 
00016 #include <argos3/core/simulator/visualization/visualization.h>
00017 #include <sys/time.h>
00018 
00019 namespace argos {
00020 
00021    class CDefaultVisualization : public CVisualization {
00022 
00023    public:
00024 
00025       CDefaultVisualization();
00026 
00027       virtual ~CDefaultVisualization() {}
00028 
00029       virtual void Init(TConfigurationNode& t_tree) {}
00030 
00031       virtual void Reset() {}
00032 
00033       virtual void Destroy() {}
00034 
00035       virtual void Execute();
00036       
00037    private:
00038 
00040       void NormalStep();
00041 
00043       void RealTimeStep();
00044 
00045    private:
00046 
00047       typedef void (CDefaultVisualization::*TStepFunction)();
00048 
00049    private:
00050 
00052       TStepFunction m_tStepFunction;
00053 
00055       ::timeval m_tStepClockTime;
00056 
00058       ::timeval m_tStepStartTime;
00059 
00061       ::timeval m_tStepEndTime;
00062 
00064       ::timeval m_tStepElapsedTime;
00065 
00067       ::timeval m_tStepWaitTime;
00068 
00069    };
00070 
00071 }
00072 
00073 #endif
```

---

Generated on 10 Jul 2018 for ARGoS by 
 1.6.1 
