# Supplementary material for: Iterative improvement in the automatic modular design of robot swarms
Source: PeerJ Comput Sci. 2020 Dec 7;6:e322. doi: 10.7717/peerj-cs.322 (PMC7924708; doi:10.7717/peerj-cs.322)
Supplement: Supplemental Information 3 [file peerj-cs-06-322-s003.zip › argos3/doc/api/standalone/a00347_source.html]

ARGoS: core/simulator/visualization/visualization.cpp Source File


- Main Page
- Related Pages
- Namespaces
- Classes
- Files

- File List
- File Members

# core/simulator/visualization/visualization.cpp

Go to the documentation of this file.

```
00001 
00007 #include <argos3/core/simulator/visualization/visualization.h>
00008 #include <argos3/core/simulator/space/space.h>
00009 
00010 #include <unistd.h>
00011 
00012 namespace argos {
00013 
00014    /****************************************/
00015    /****************************************/
00016 
00017    static Real TVTimeToHumanReadable(::timeval& t_time) {
00018       return
00019          static_cast<Real>(t_time.tv_sec) +
00020          static_cast<Real>(t_time.tv_usec * 10e-6);
00021    }
00022 
00023    /****************************************/
00024    /****************************************/
00025 
00026    void CDefaultVisualization::Init(TConfigurationNode& t_tree) {
00027       /* Get visualization id from the XML */
00028       //GetNodeAttribute(t_tree, "id", m_strId);
00029       /* Set the pointer to the step function */
00030       if(m_cSimulator.IsRealTimeClock()) {
00031          /* Use real-time clock and set time structures */
00032          m_tStepFunction = &CDefaultVisualization::RealTimeStep;
00033          timerclear(&m_tStepClockTime);
00034          m_tStepClockTime.tv_usec = 1e6 * CPhysicsEngine::GetSimulationClockTick();
00035          ::gettimeofday(&m_tStepStartTime, NULL);
00036       }
00037       else {
00038          /* Use normal clock */
00039          m_tStepFunction = &CDefaultVisualization::NormalStep;
00040       }
00041    }
00042 
00043    /****************************************/
00044    /****************************************/
00045 
00046    void CDefaultVisualization::Execute() {
00047       /* Main cycle */
00048       while(!m_cSimulator.IsExperimentFinished()) {
00049          (this->*m_tStepFunction)();
00050       }
00051    }
00052 
00053    /****************************************/
00054    /****************************************/
00055 
00056    void CDefaultVisualization::NormalStep() {
00057       m_cSimulator.UpdateSpace();
00058    }
00059 
00060    /****************************************/
00061    /****************************************/
00062 
00063    void CDefaultVisualization::RealTimeStep() {
00064       /* m_tStepStartTime has already been set */
00065       m_cSimulator.UpdateSpace();
00066       /* Take the time now */
00067       ::gettimeofday(&m_tStepEndTime, NULL);
00068       /* Calculate the elapsed time */
00069       timersub(&m_tStepEndTime, &m_tStepStartTime, &m_tStepElapsedTime);
00070       /* If the elapsed time is lower than the tick length, wait */
00071       if(!timercmp(&m_tStepElapsedTime, &m_tStepClockTime, >)) {
00072          /* Calculate the waiting time */
00073          timersub(&m_tStepClockTime, &m_tStepElapsedTime, &m_tStepWaitTime);
00074          /* Wait */
00075          ::usleep(m_tStepWaitTime.tv_sec * 1e6 + m_tStepWaitTime.tv_usec);
00076          /* Get the new step end */
00077          ::gettimeofday(&m_tStepEndTime, NULL);
00078       }
00079       else {
00080          LOGERR << "[WARNING] Clock tick took "
00081                 << TVTimeToHumanReadable(m_tStepElapsedTime)
00082                 << " sec, more than the expected "
00083                 << TVTimeToHumanReadable(m_tStepClockTime)
00084                 << " sec."
00085                 << std::endl;
00086       }
00087       /* Set the step start time to whatever the step end time is */
00088       m_tStepStartTime.tv_sec = m_tStepEndTime.tv_sec;
00089       m_tStepStartTime.tv_usec = m_tStepEndTime.tv_usec;
00090    }
00091 
00092    /****************************************/
00093    /****************************************/
00094 
00095 }
```

---

Generated on 10 Jul 2018 for ARGoS by 
 1.6.1 
