# Supplementary material for: Iterative improvement in the automatic modular design of robot swarms
Source: PeerJ Comput Sci. 2020 Dec 7;6:e322. doi: 10.7717/peerj-cs.322 (PMC7924708; doi:10.7717/peerj-cs.322)
Supplement: Supplemental Information 3 [file peerj-cs-06-322-s003.zip › argos3/doc/api/standalone/a00348_source.html]

ARGoS: core/simulator/visualization/visualization.h Source File


- Main Page
- Related Pages
- Namespaces
- Classes
- Files

- File List
- File Members

# core/simulator/visualization/visualization.h

Go to the documentation of this file.

```
00001 
00012 #ifndef VISUALIZATION_H
00013 #define VISUALIZATION_H
00014 
00015 namespace argos {
00016    class CVisualization;
00017 }
00018 
00019 #include <argos3/core/utility/configuration/base_configurable_resource.h>
00020 #include <argos3/core/simulator/simulator.h>
00021 
00022 namespace argos {
00023 
00024    class CVisualization : public CBaseConfigurableResource {
00025 
00026    public:
00027 
00028       CVisualization() :
00029          m_cSimulator(CSimulator::GetInstance()),
00030          m_cSpace(m_cSimulator.GetSpace()) {}
00031 
00032       virtual ~CVisualization() {}
00033 
00034       virtual void Init(TConfigurationNode& t_tree) = 0;
00035 
00036       virtual void Reset() = 0;
00037 
00038       virtual void Destroy() = 0;
00039 
00040       virtual void Execute() = 0;
00041       
00042    protected:
00043 
00045       CSimulator& m_cSimulator;
00046 
00048       CSpace& m_cSpace;
00049 
00050    };
00051 
00052 #define REGISTER_VISUALIZATION(CLASSNAME,           \
00053                                LABEL,               \
00054                                AUTHOR,              \
00055                                VERSION,             \
00056                                BRIEF_DESCRIPTION,   \
00057                                LONG_DESCRIPTION,    \
00058                                STATUS)              \
00059    REGISTER_SYMBOL(CVisualization,                  \
00060                    CLASSNAME,                       \
00061                    LABEL,                           \
00062                    AUTHOR,                          \
00063                    VERSION,                         \
00064                    BRIEF_DESCRIPTION,               \
00065                    LONG_DESCRIPTION,                \
00066                    STATUS)
00067 
00068 }
00069 
00070 #endif
```

---

Generated on 10 Jul 2018 for ARGoS by 
 1.6.1 
