# Supplementary material for: Iterative improvement in the automatic modular design of robot swarms
Source: PeerJ Comput Sci. 2020 Dec 7;6:e322. doi: 10.7717/peerj-cs.322 (PMC7924708; doi:10.7717/peerj-cs.322)
Supplement: Supplemental Information 3 [file peerj-cs-06-322-s003.zip › argos3/doc/api/standalone/a00349.html]

ARGoS: core/utility/configuration/argos\_configuration.h File Reference


- Main Page
- Related Pages
- Namespaces
- Classes
- Files

- File List
- File Members

# core/utility/configuration/argos\_configuration.h File Reference

`#include <argos3/core/utility/datatypes/datatypes.h>`  
`#include <argos3/core/utility/configuration/argos_exception.h>`  
`#include <argos3/core/utility/configuration/tinyxml/ticpp.h>`  
`#include <string>`  

Include dependency graph for argos\_configuration.h:

Go to the source code of this file.

|  |  |
| --- | --- |
| Namespaces | |
| namespace | argos |

|  |  |
| --- | --- |
|  | The namespace containing all the ARGoS related code. |

| Typedefs | |
| typedef ticpp::Element | argos::TConfigurationNode |
|  | The ARGoS configuration XML node. |
| typedef ticpp::Iterator  < ticpp::Element > | argos::TConfigurationNodeIterator |
|  | The iterator for the ARGoS configuration XML node. |
| typedef ticpp::Iterator  < ticpp::Attribute > | argos::TConfigurationAttributeIterator |
|  | The iterator for the attributes of an XML node. |
| Functions | |
| bool | argos::NodeExists (TConfigurationNode &t\_node, const std::string &str\_tag) throw () |
|  | Given a tree root node, returns `true` if one of its child nodes has the wanted name. |
| TConfigurationNode & | argos::GetNode (TConfigurationNode &t\_node, const std::string &str\_tag) |
|  | Given a tree root node, returns the first of its child nodes with the wanted name. |
| void | argos::AddChildNode (TConfigurationNode &t\_parent\_node, TConfigurationNode &t\_child\_node) |
|  | Adds an XML node as child of another XML node. |
| template<typename T > | |
| void | argos::GetNodeText (TConfigurationNode &t\_node, T &t\_buffer) |
|  | Returns the text of the passed XML node A node text is as follows:. |
| template<typename T > | |
| void | argos::GetNodeTextOrDefault (TConfigurationNode &t\_node, T &t\_buffer, const T &t\_default) |
|  | Returns the text of the passed XML node, or the passed default value. |
| bool | argos::NodeAttributeExists (TConfigurationNode &t\_node, const std::string &str\_attribute) |
|  | Returns `true` if the specified attribute of a node exists. |
| template<typename T > | |
| void | argos::GetNodeAttribute (TConfigurationNode &t\_node, const std::string &str\_attribute, T &t\_buffer) |
|  | Returns the value of a node's attribute. |
| void | argos::GetNodeAttribute (TConfigurationNode &t\_node, const std::string &str\_attribute, bool &b\_buffer) |
|  | Returns the value of a node's attribute. |
| void | argos::GetNodeAttribute (TConfigurationNode &t\_node, const std::string &str\_attribute, UInt8 &un\_buffer) |
|  | Returns the value of a node's attribute. |
| void | argos::GetNodeAttribute (TConfigurationNode &t\_node, const std::string &str\_attribute, SInt8 &n\_buffer) |
|  | Returns the value of a node's attribute. |
| template<typename T > | |
| void | argos::GetNodeAttributeOrDefault (TConfigurationNode &t\_node, const std::string &str\_attribute, T &t\_buffer, const T &t\_default) |
|  | Returns the value of a node's attribute, or the passed default value. |
| void | argos::GetNodeAttributeOrDefault (TConfigurationNode &t\_node, const std::string &str\_attribute, bool &b\_buffer, const bool b\_default) |
|  | Returns the value of a node's attribute, or the passed default value. |
| void | argos::GetNodeAttributeOrDefault (TConfigurationNode &t\_node, const std::string &str\_attribute, UInt8 &un\_buffer, const UInt8 un\_default) |
|  | Returns the value of a node's attribute, or the passed default value. |
| void | argos::GetNodeAttributeOrDefault (TConfigurationNode &t\_node, const std::string &str\_attribute, SInt8 &n\_buffer, const SInt8 n\_default) |
|  | Returns the value of a node's attribute, or the passed default value. |
| template<typename T > | |
| void | argos::SetNodeAttribute (TConfigurationNode &t\_node, const std::string &str\_attribute, const T &t\_value) |
|  | Sets the value of the wanted node's attribute. |
| void | argos::SetNodeAttribute (TConfigurationNode &t\_node, const std::string &str\_attribute, const bool b\_value) |
|  | Sets the value of the wanted node's attribute. |
| void | argos::SetNodeAttribute (TConfigurationNode &t\_node, const std::string &str\_attribute, const SInt8 n\_value) |
|  | Sets the value of the wanted node's attribute. |
| void | argos::SetNodeAttribute (TConfigurationNode &t\_node, const std::string &str\_attribute, const UInt8 un\_value) |
|  | Sets the value of the wanted node's attribute. |

---

Generated on 10 Jul 2018 for ARGoS by 
 1.6.1 
