# Supplementary material for: Iterative improvement in the automatic modular design of robot swarms
Source: PeerJ Comput Sci. 2020 Dec 7;6:e322. doi: 10.7717/peerj-cs.322 (PMC7924708; doi:10.7717/peerj-cs.322)
Supplement: Supplemental Information 3 [file peerj-cs-06-322-s003.zip › argos3/doc/api/standalone/a00349_source.html]

ARGoS: core/utility/configuration/argos\_configuration.h Source File


- Main Page
- Related Pages
- Namespaces
- Classes
- Files

- File List
- File Members

# core/utility/configuration/argos\_configuration.h

Go to the documentation of this file.

```
00001 
00013 #ifndef ARGOS_CONFIGURATION_H
00014 #define ARGOS_CONFIGURATION_H
00015 
00016 #include <argos3/core/utility/datatypes/datatypes.h>
00017 #include <argos3/core/utility/configuration/argos_exception.h>
00018 #include <argos3/core/utility/configuration/tinyxml/ticpp.h>
00019 #include <string>
00020 
00021 namespace argos {
00022 
00023    /****************************************/
00024    /****************************************/
00025 
00027    typedef ticpp::Element TConfigurationNode;
00029    typedef ticpp::Iterator <ticpp::Element> TConfigurationNodeIterator;
00031    typedef ticpp::Iterator <ticpp::Attribute> TConfigurationAttributeIterator;
00032 
00033    /****************************************/
00034    /****************************************/
00035 
00044    inline bool NodeExists(TConfigurationNode& t_node,
00045                           const std::string& str_tag) throw() {
00046       TConfigurationNodeIterator it(str_tag);
00047       it = it.begin(&t_node);
00048       return it != NULL;
00049    }
00050 
00051    /****************************************/
00052    /****************************************/
00053 
00063    inline TConfigurationNode& GetNode(TConfigurationNode& t_node,
00064                                       const std::string& str_tag) {
00065       try {
00066          TConfigurationNodeIterator it(str_tag);
00067          it = it.begin(&t_node);
00068          if(it == NULL) {
00069             THROW_ARGOSEXCEPTION("Node '" << str_tag << "' not found");
00070          }
00071          return *it;
00072       }
00073       catch(ticpp::Exception& ex) {
00074          THROW_ARGOSEXCEPTION_NESTED("Error searching for '" << str_tag << "' ", ex);
00075       }
00076    }
00077 
00078    /****************************************/
00079    /****************************************/
00080 
00088    inline void AddChildNode(TConfigurationNode& t_parent_node,
00089                             TConfigurationNode& t_child_node) {
00090       try {
00091          t_parent_node.InsertEndChild(t_child_node);
00092       }
00093       catch(ticpp::Exception& ex) {
00094          THROW_ARGOSEXCEPTION_NESTED("Error inserting node '" << t_child_node << "' into node '" << t_parent_node << "'", ex);
00095       }      
00096    }
00097 
00098    /****************************************/
00099    /****************************************/
00100 
00127    template <typename T>
00128    void GetNodeText(TConfigurationNode& t_node,
00129                     T& t_buffer) {
00130       try {
00131          t_node.GetText(&t_buffer);
00132       }
00133       catch(std::exception& ex) {
00134          THROW_ARGOSEXCEPTION_NESTED("Parse error", ex);
00135       }
00136    }
00137 
00138    /****************************************/
00139    /****************************************/
00140 
00151    template <typename T>
00152    void GetNodeTextOrDefault(TConfigurationNode& t_node,
00153                              T& t_buffer,
00154                              const T& t_default) {
00155       try {
00156          t_node.GetTextOrDefault(&t_buffer, t_default);
00157       }
00158       catch(std::exception& ex) {
00159          THROW_ARGOSEXCEPTION_NESTED("Parse error", ex);
00160       }
00161    }
00162 
00163    /****************************************/
00164    /****************************************/
00165 
00172    inline bool NodeAttributeExists(TConfigurationNode& t_node,
00173                                    const std::string& str_attribute) {
00174       return t_node.HasAttribute(str_attribute);
00175    }
00176 
00177    /****************************************/
00178    /****************************************/
00179 
00207    template <typename T>
00208    void GetNodeAttribute(TConfigurationNode& t_node,
00209                          const std::string& str_attribute,
00210                          T& t_buffer) {
00211       try {
00212          t_node.GetAttribute(str_attribute, &t_buffer, true);
00213       }
00214       catch(ticpp::Exception& ex) {
00215          THROW_ARGOSEXCEPTION_NESTED("Error parsing attribute \"" << str_attribute << "\"", ex);
00216       }
00217    }
00218 
00219    /****************************************/
00220    /****************************************/
00221 
00231    inline void GetNodeAttribute(TConfigurationNode& t_node,
00232                                 const std::string& str_attribute,
00233                                 bool& b_buffer) {
00234       std::string strBuffer;
00235       try {
00236          t_node.GetAttribute(str_attribute, &strBuffer, true);
00237          if(strBuffer == "true") {
00238             b_buffer = true;
00239          }
00240          else if(strBuffer == "false") {
00241             b_buffer = false;
00242          }
00243          else {
00244             THROW_ARGOSEXCEPTION("Cannot convert '" << strBuffer << "' into a bool. Accepted values: 'true', 'false'.");
00245          }
00246       }
00247       catch(ticpp::Exception& ex) {
00248          THROW_ARGOSEXCEPTION_NESTED("Error parsing attribute \"" << str_attribute << "\"", ex);
00249       }
00250    }
00251 
00252    /****************************************/
00253    /****************************************/
00254 
00264    inline void GetNodeAttribute(TConfigurationNode& t_node,
00265                                 const std::string& str_attribute,
00266                                 UInt8& un_buffer) {
00267       try {
00268          UInt32 unTmpBuffer;
00269          t_node.GetAttribute(str_attribute, &unTmpBuffer, true);
00270          un_buffer = unTmpBuffer;
00271       }
00272       catch(ticpp::Exception& ex) {
00273          THROW_ARGOSEXCEPTION_NESTED("Error parsing attribute \"" << str_attribute << "\"", ex);
00274       }
00275    }
00276 
00277    /****************************************/
00278    /****************************************/
00279 
00289    inline void GetNodeAttribute(TConfigurationNode& t_node,
00290                                 const std::string& str_attribute,
00291                                 SInt8& n_buffer) {
00292       try {
00293          SInt32 nTmpBuffer;
00294          t_node.GetAttribute(str_attribute, &nTmpBuffer, true);
00295          n_buffer = nTmpBuffer;
00296       }
00297       catch(ticpp::Exception& ex) {
00298          THROW_ARGOSEXCEPTION_NESTED("Error parsing attribute \"" << str_attribute << "\"", ex);
00299       }
00300    }
00301 
00302    /****************************************/
00303    /****************************************/
00304 
00317    template <typename T>
00318    void GetNodeAttributeOrDefault(TConfigurationNode& t_node,
00319                                   const std::string& str_attribute,
00320                                   T& t_buffer,
00321                                   const T& t_default) {
00322       try {
00323          t_node.GetAttributeOrDefault(str_attribute, &t_buffer, t_default);
00324       }
00325       catch(ticpp::Exception& ex) {
00326          THROW_ARGOSEXCEPTION_NESTED("Error parsing attribute \"" << str_attribute << "\"", ex);
00327       }
00328    }
00329 
00330    /****************************************/
00331    /****************************************/
00332 
00344    inline void GetNodeAttributeOrDefault(TConfigurationNode& t_node,
00345                                          const std::string& str_attribute,
00346                                          bool& b_buffer,
00347                                          const bool b_default) {
00348       std::string strBuffer;
00349       const std::string strDefault = (b_default ? "true" : "false");
00350       try {
00351          t_node.GetAttributeOrDefault(str_attribute, &strBuffer, strDefault);
00352          if(strBuffer == "true") {
00353             b_buffer = true;
00354          }
00355          else if(strBuffer == "false") {
00356             b_buffer = false;
00357          }
00358          else {
00359             THROW_ARGOSEXCEPTION("Cannot convert '" << strBuffer << "' into a bool. Accepted values: 'true', 'false'.");
00360          }
00361       }
00362       catch(ticpp::Exception& ex) {
00363          THROW_ARGOSEXCEPTION_NESTED("Error parsing attribute \"" << str_attribute << "\"", ex);
00364       }
00365    }
00366 
00367    /****************************************/
00368    /****************************************/
00369 
00381    inline void GetNodeAttributeOrDefault(TConfigurationNode& t_node,
00382                                          const std::string& str_attribute,
00383                                          UInt8& un_buffer,
00384                                          const UInt8 un_default) {
00385       try {
00386          UInt32 unTmpBuffer;
00387          t_node.GetAttributeOrDefault(str_attribute, &unTmpBuffer, static_cast<UInt32>(un_default));
00388          un_buffer = unTmpBuffer;
00389       }
00390       catch(ticpp::Exception& ex) {
00391          THROW_ARGOSEXCEPTION_NESTED("Error parsing attribute \"" << str_attribute << "\"", ex);
00392       }
00393    }
00394 
00395    /****************************************/
00396    /****************************************/
00397 
00409    inline void GetNodeAttributeOrDefault(TConfigurationNode& t_node,
00410                                          const std::string& str_attribute,
00411                                          SInt8& n_buffer,
00412                                          const SInt8 n_default) {
00413       try {
00414          SInt32 nTmpBuffer;
00415          t_node.GetAttributeOrDefault(str_attribute, &nTmpBuffer, static_cast<SInt32>(n_default));
00416          n_buffer = nTmpBuffer;
00417       }
00418       catch(ticpp::Exception& ex) {
00419          THROW_ARGOSEXCEPTION_NESTED("Error parsing attribute \"" << str_attribute << "\"", ex);
00420       }
00421    }
00422 
00423    /****************************************/
00424    /****************************************/
00425 
00433    template <typename T>
00434    void SetNodeAttribute(TConfigurationNode& t_node,
00435                          const std::string& str_attribute,
00436                          const T& t_value) {
00437       t_node.SetAttribute(str_attribute, t_value);
00438    }
00439 
00440    /****************************************/
00441    /****************************************/
00442 
00451    inline void SetNodeAttribute(TConfigurationNode& t_node,
00452                                 const std::string& str_attribute,
00453                                 const bool b_value) {
00454       if(b_value) {
00455          t_node.SetAttribute(str_attribute, "true");
00456       }
00457       else {
00458          t_node.SetAttribute(str_attribute, "false");
00459       }
00460    }
00461 
00462    /****************************************/
00463    /****************************************/
00464 
00473    inline void SetNodeAttribute(TConfigurationNode& t_node,
00474                                 const std::string& str_attribute,
00475                                 const SInt8 n_value) {
00476       t_node.SetAttribute(str_attribute, static_cast<SInt32>(n_value));
00477    }
00478 
00479    /****************************************/
00480    /****************************************/
00481 
00490    inline void SetNodeAttribute(TConfigurationNode& t_node,
00491                                 const std::string& str_attribute,
00492                                 const UInt8 un_value) {
00493       t_node.SetAttribute(str_attribute, static_cast<UInt32>(un_value));
00494    }
00495 
00496    /****************************************/
00497    /****************************************/
00498 
00499 }
00500 
00501 #endif
```

---

Generated on 10 Jul 2018 for ARGoS by 
 1.6.1 
