# Supplementary material for: Iterative improvement in the automatic modular design of robot swarms
Source: PeerJ Comput Sci. 2020 Dec 7;6:e322. doi: 10.7717/peerj-cs.322 (PMC7924708; doi:10.7717/peerj-cs.322)
Supplement: Supplemental Information 3 [file peerj-cs-06-322-s003.zip › argos3/doc/api/standalone/a00350.html]

ARGoS: core/utility/configuration/argos\_exception.h File Reference


- Main Page
- Related Pages
- Namespaces
- Classes
- Files

- File List
- File Members

# core/utility/configuration/argos\_exception.h File Reference

`#include <stdexcept>`  
`#include <sstream>`  

Include dependency graph for argos\_exception.h:

Go to the source code of this file.

|  |  |
| --- | --- |
| Classes | |
| class | argos::CARGoSException |
|  | The exception that wraps all errors in ARGoS. More... |
| Namespaces | |
| namespace | argos |

|  |  |
| --- | --- |
|  | The namespace containing all the ARGoS related code. |

| Defines | |
| #define | THROW\_ARGOSEXCEPTION(message)   { std::ostringstream what; what << message; throw CARGoSException(what.str()); } |
|  | This macro throws an ARGoS exception with the passed message. |
| #define | THROW\_ARGOSEXCEPTION\_NESTED(message, nested)   { std::ostringstream what; what << message; throw CARGoSException(what.str(), &nested); } |
|  | This macro throws an ARGoS exception with the passed message and nesting the passed exception. |
| #define | ARGOS\_ASSERT(condition, message)   { if ( !(condition) ) THROW\_ARGOSEXCEPTION(message); } |
|  | When code is compiled in debug, this macro throws an ARGoS exception with the passed message if the specified condition is not met. |

---

## Define Documentation

|  |  |  |
| --- | --- | --- |
| #define ARGOS\_ASSERT | ( | condition, |
|  |  | message |  | ) | { if ( !(condition) ) THROW\_ARGOSEXCEPTION(message); } |

When code is compiled in debug, this macro throws an ARGoS exception with the passed message if the specified condition is not met.

When code is compiled in release, this macro does nothing.

Definition at line 122 of file argos\_exception.h.

|  |  |  |  |  |  |
| --- | --- | --- | --- | --- | --- |
| #define THROW\_ARGOSEXCEPTION | ( | message |  | ) | { std::ostringstream what; what << message; throw CARGoSException(what.str()); } |

This macro throws an ARGoS exception with the passed message.

Definition at line 111 of file argos\_exception.h.

|  |  |  |
| --- | --- | --- |
| #define THROW\_ARGOSEXCEPTION\_NESTED | ( | message, |
|  |  | nested |  | ) | { std::ostringstream what; what << message; throw CARGoSException(what.str(), &nested); } |

This macro throws an ARGoS exception with the passed message and nesting the passed exception.

Definition at line 115 of file argos\_exception.h.

---

Generated on 10 Jul 2018 for ARGoS by 
 1.6.1 
