# Supplementary material for: Iterative improvement in the automatic modular design of robot swarms
Source: PeerJ Comput Sci. 2020 Dec 7;6:e322. doi: 10.7717/peerj-cs.322 (PMC7924708; doi:10.7717/peerj-cs.322)
Supplement: Supplemental Information 3 [file peerj-cs-06-322-s003.zip › argos3/doc/api/standalone/a00350_source.html]

ARGoS: core/utility/configuration/argos\_exception.h Source File


- Main Page
- Related Pages
- Namespaces
- Classes
- Files

- File List
- File Members

# core/utility/configuration/argos\_exception.h

Go to the documentation of this file.

```
00001 
00043 #ifndef ARGOS_EXCEPTION_H
00044 #define ARGOS_EXCEPTION_H
00045 
00046 namespace argos {
00047    class CARGoSException;
00048 }
00049 
00050 #include <stdexcept>
00051 #include <sstream>
00052 
00053 namespace argos {
00054 
00061    class CARGoSException : public std::exception {
00062 
00063    public:
00064 
00070       CARGoSException(const std::string& str_what, std::exception* pc_nested = NULL) throw() :
00071          m_strWhat("[FATAL] " + str_what), m_pcNested(pc_nested) {
00072          if (m_pcNested != NULL) {
00073             std::ostringstream w;
00074             w << m_strWhat
00075               << std::endl
00076               << m_pcNested->what();
00077             m_strWhat = w.str();
00078          }
00079       }
00083       virtual ~CARGoSException() throw() {
00084       }
00085 
00090       virtual const char* what() const throw() {
00091          return m_strWhat.c_str();
00092       }
00093 
00094    private:
00095 
00100       std::string m_strWhat;
00102       std::exception* m_pcNested;
00103 
00104    };
00105 
00106 }
00107 
00111 #define THROW_ARGOSEXCEPTION(message) { std::ostringstream what; what << message; throw CARGoSException(what.str()); }
00112 
00115 #define THROW_ARGOSEXCEPTION_NESTED(message, nested) { std::ostringstream what; what << message; throw CARGoSException(what.str(), &nested); }
00116 
00117 #ifndef NDEBUG
00118 
00122 #define ARGOS_ASSERT(condition, message) { if ( !(condition) ) THROW_ARGOSEXCEPTION(message); }
00123 #else
00124 #define ARGOS_ASSERT(condition, message)
00125 #endif
00126 
00127 #endif
```

---

Generated on 10 Jul 2018 for ARGoS by 
 1.6.1 
