# Supplementary material for: Iterative improvement in the automatic modular design of robot swarms
Source: PeerJ Comput Sci. 2020 Dec 7;6:e322. doi: 10.7717/peerj-cs.322 (PMC7924708; doi:10.7717/peerj-cs.322)
Supplement: Supplemental Information 3 [file peerj-cs-06-322-s003.zip › argos3/doc/api/standalone/a00351_source.html]

ARGoS: core/utility/configuration/base\_configurable\_resource.h Source File


- Main Page
- Related Pages
- Namespaces
- Classes
- Files

- File List
- File Members

# core/utility/configuration/base\_configurable\_resource.h

Go to the documentation of this file.

```
00001 
00009 #ifndef BASE_CONFIGURABLE_RESOURCE_H
00010 #define BASE_CONFIGURABLE_RESOURCE_H
00011 
00012 namespace argos {
00013    class CBaseConfigurableResource;
00014 }
00015 
00016 #include <argos3/core/utility/configuration/argos_configuration.h>
00017 
00018 namespace argos {
00019 
00023    class CBaseConfigurableResource {
00024 
00025    public:
00026 
00030       virtual ~CBaseConfigurableResource() {}
00031 
00037       virtual void Init(TConfigurationNode& t_tree) = 0;
00038 
00042       virtual void Reset() = 0;
00043 
00047       virtual void Destroy() = 0;
00048 
00049    };
00050 
00051 }
00052 
00053 #endif
```

---

Generated on 10 Jul 2018 for ARGoS by 
 1.6.1 
