# Supplementary material for: Iterative improvement in the automatic modular design of robot swarms
Source: PeerJ Comput Sci. 2020 Dec 7;6:e322. doi: 10.7717/peerj-cs.322 (PMC7924708; doi:10.7717/peerj-cs.322)
Supplement: Supplemental Information 3 [file peerj-cs-06-322-s003.zip › argos3/doc/api/standalone/a00352_source.html]

ARGoS: core/utility/configuration/command\_line\_arg\_parser.cpp Source File


- Main Page
- Related Pages
- Namespaces
- Classes
- Files

- File List
- File Members

# core/utility/configuration/command\_line\_arg\_parser.cpp

Go to the documentation of this file.

```
00001 
00007 #include "command_line_arg_parser.h"
00008 #include <cstring>
00009 
00010 namespace argos {
00011 
00012    /****************************************/
00013    /****************************************/
00014 
00015    CCommandLineArgParser::CCommandLineArgParser() :
00016       m_nCurrentArgument(0) {
00017    }
00018 
00019    /****************************************/
00020    /****************************************/
00021 
00022    CCommandLineArgParser::~CCommandLineArgParser() {
00023       while(! m_vecArguments.empty()) {
00024          delete m_vecArguments.back();
00025          m_vecArguments.pop_back();
00026       }
00027    }
00028 
00029    /****************************************/
00030    /****************************************/
00031 
00032    void CCommandLineArgParser::PrintUsage(CARGoSLog& c_log) {
00033       for(size_t i = 0; i < m_vecArguments.size(); ++i) {
00034          c_log << "-" << m_vecArguments[i]->ShortOption << "|"
00035                << "--" << m_vecArguments[i]->LongOption;
00036          if(!m_vecArguments[i]->IsFlag) {
00037             c_log << " <value>";
00038          }
00039          c_log << "\t"
00040                << m_vecArguments[i]->Description
00041                << std::endl;
00042       }
00043    }
00044 
00045    /****************************************/
00046    /****************************************/
00047 
00048    void CCommandLineArgParser::Parse(SInt32 n_argc,
00049                                      char** ppch_argv) {
00050       /* Used to store information about each argument to parse */
00051       size_t unArgLength;
00052       char* pchCurrentArg;
00053       /* Start parsing from the beginning */
00054       m_nCurrentArgument = 1;
00055       /* Go through all the passed arguments */
00056       while(m_nCurrentArgument < n_argc) {
00057          /*
00058           * Parsing does as follows:
00059           * If the first characters is a - followed by other charactares, then it's an option
00060           *   If the second character is a - too, it's a long one
00061           *   Otherwise it's a (set of) short option(s)
00062           * If the first character is not a -, or the string is just "-", error
00063           */
00064          pchCurrentArg = ppch_argv[m_nCurrentArgument];
00065          unArgLength = ::strlen(pchCurrentArg);
00066          if(ppch_argv[m_nCurrentArgument][0] != '-' ||
00067             unArgLength == 1) {
00068             THROW_ARGOSEXCEPTION("Unrecognized option \"" << pchCurrentArg << "\".");
00069          }
00070          else {
00071             /* The first character is a - and more characters are to come */
00072             if(pchCurrentArg[1] == '-') {
00073                /* Also the second char is a - */
00074                if(unArgLength > 2) {
00075                   /* We have a long option */
00076                   ParseLongOption(n_argc, ppch_argv);
00077                }
00078                else {
00079                   /* It's just a --, which we don't support */
00080                   THROW_ARGOSEXCEPTION("Unrecognized option \"" << pchCurrentArg << "\".");
00081                }
00082             }
00083             else {
00084                /* We have a short option like -x, or a set of them like -xyz */
00085                if(unArgLength == 2) {
00086                   /* Single short option: -x */
00087                   ParseShortOption(n_argc, ppch_argv);
00088                }
00089                else {
00090                   /* Set of short options: -xyz */
00091                   ParseShortOptions(n_argc, ppch_argv);
00092                }
00093             }
00094          }
00095          /* Parse next option */
00096          ++m_nCurrentArgument;
00097       }
00098    }
00099 
00100    /****************************************/
00101    /****************************************/
00102 
00103    void CCommandLineArgParser::ParseLongOption(SInt32 n_argc,
00104                                                char** ppch_argv) {
00105       /* Strip the initial -- from the option */
00106       std::string strArg(ppch_argv[m_nCurrentArgument]+2);
00107       try {
00108          /* Search for the option in the vector */
00109          for(size_t i = 0; i < m_vecArguments.size(); ++i) {
00110             if(m_vecArguments[i]->LongOption == strArg) {
00111                /* The option has been found */
00112                if(m_vecArguments[i]->IsFlag) {
00113                   /* It's a flag, pass "true" to set the boolean to true */
00114                   m_vecArguments[i]->Parse("true");
00115                }
00116                else {
00117                   /* It's an argument, use what follows as parameter */
00118                   ++m_nCurrentArgument;
00119                   if(m_nCurrentArgument == n_argc) {
00120                      THROW_ARGOSEXCEPTION("Missing argument for option \"--" << strArg << "\".");
00121                   }
00122                   m_vecArguments[i]->Parse(ppch_argv[m_nCurrentArgument]);
00123                }
00124                /* No need to continue searching */
00125                return;
00126             }
00127          }
00128       }
00129       catch(CARGoSException& ex) {
00130          /* Something went wrong with the parsing */
00131          THROW_ARGOSEXCEPTION_NESTED("Error parsing option \"--" << strArg << "\".", ex);
00132       }
00133       /* If you get here, it's because the option hasn't been found */
00134       THROW_ARGOSEXCEPTION("Unrecognized option \"--" << strArg << "\".");
00135    }
00136 
00137    /****************************************/
00138    /****************************************/
00139 
00140    void CCommandLineArgParser::ParseShortOption(SInt32 n_argc,
00141                                                 char** ppch_argv) {
00142       /* Strip the initial - from the option */
00143       char strArg(ppch_argv[m_nCurrentArgument][1]);
00144       try {
00145          /* Search for the option in the vector */
00146          for(size_t i = 0; i < m_vecArguments.size(); ++i) {
00147             if(m_vecArguments[i]->ShortOption == strArg) {
00148                /* The option has been found */
00149                if(m_vecArguments[i]->IsFlag) {
00150                   /* It's a flag, pass "true" to set the boolean to true */
00151                   m_vecArguments[i]->Parse("true");
00152                }
00153                else {
00154                   /* It's an argument, use what follows as parameter */
00155                   ++m_nCurrentArgument;
00156                   if(m_nCurrentArgument == n_argc) {
00157                      THROW_ARGOSEXCEPTION("Missing argument for option \"-" << strArg << "\".");
00158                   }
00159                   m_vecArguments[i]->Parse(ppch_argv[m_nCurrentArgument]);
00160                }
00161                /* No need to continue searching */
00162                return;
00163             }
00164          }
00165       }
00166       catch(CARGoSException& ex) {
00167          /* Something went wrong with the parsing */
00168          THROW_ARGOSEXCEPTION_NESTED("Error parsing option \"-" << strArg << "\".", ex);
00169       }
00170       /* If you get here, it's because the option hasn't been found */
00171       THROW_ARGOSEXCEPTION("Unrecognized option \"-" << strArg << "\".");
00172    }
00173 
00174    /****************************************/
00175    /****************************************/
00176 
00177    void CCommandLineArgParser::ParseShortOptions(SInt32 n_argc,
00178                                                  char** ppch_argv) {
00179       /* Strip the initial - from the option */
00180       std::string strArg(ppch_argv[m_nCurrentArgument]+1);
00181       /* Go through all options */
00182       for(size_t o = 0; o < strArg.length(); ++o) {
00183          /* Search for the option in the vector */
00184          size_t i = 0;
00185          bool bFound = false;
00186          try {
00187             while(i < m_vecArguments.size() && !bFound) {
00188                if(m_vecArguments[i]->ShortOption == strArg[o]) {
00189                   /* The option has been found */
00190                   if(m_vecArguments[i]->IsFlag) {
00191                      /* It's a flag, pass "true" to set the boolean to true */
00192                      m_vecArguments[i]->Parse("true");
00193                   }
00194                   else {
00195                      /* It's an argument, only the last short option is allowed to have one */
00196                      if(o < strArg.length()-1) {
00197                         THROW_ARGOSEXCEPTION("Option \"-" << strArg[o] << "\" requires an argument.");
00198                      }
00199                      else {
00200                         ++m_nCurrentArgument;
00201                         m_vecArguments[i]->Parse(ppch_argv[m_nCurrentArgument]);
00202                      }
00203                   }
00204                   /* No need to continue searching */
00205                   bFound = true;
00206                }
00207                else {
00208                   /* Try next option */
00209                   ++i;
00210                }
00211             }
00212          }
00213          catch(CARGoSException& ex) {
00214             /* Something went wrong with the parsing */
00215             THROW_ARGOSEXCEPTION_NESTED("Error parsing option \"-" << strArg << "\".", ex);
00216          }
00217          if(!bFound) {
00218             /* The option hasn't been found */
00219             THROW_ARGOSEXCEPTION("Unrecognized option \"-" << strArg[o] << "\".");
00220          }
00221       }
00222    }
00223 
00224    /****************************************/
00225    /****************************************/
00226    
00227 }
```

---

Generated on 10 Jul 2018 for ARGoS by 
 1.6.1 
