# Supplementary material for: Iterative improvement in the automatic modular design of robot swarms
Source: PeerJ Comput Sci. 2020 Dec 7;6:e322. doi: 10.7717/peerj-cs.322 (PMC7924708; doi:10.7717/peerj-cs.322)
Supplement: Supplemental Information 3 [file peerj-cs-06-322-s003.zip › argos3/doc/api/standalone/a00353_source.html]

ARGoS: core/utility/configuration/command\_line\_arg\_parser.h Source File


- Main Page
- Related Pages
- Namespaces
- Classes
- Files

- File List
- File Members

# core/utility/configuration/command\_line\_arg\_parser.h

Go to the documentation of this file.

```
00001 
00026 #ifndef COMMAND_LINE_ARG_PARSER_H
00027 #define COMMAND_LINE_ARG_PARSER_H
00028 
00029 namespace argos {
00030    class CCommandLineArgParser;
00031 }
00032 
00033 #include <argos3/core/utility/datatypes/datatypes.h>
00034 #include <argos3/core/utility/string_utilities.h>
00035 #include <argos3/core/utility/logging/argos_log.h>
00036 #include <vector>
00037 #include <string>
00038 #include <iostream>
00039 
00040 namespace argos {
00041 
00090    class CCommandLineArgParser {
00091 
00092    public:
00093 
00097       CCommandLineArgParser();
00098 
00102       virtual ~CCommandLineArgParser();
00103 
00111       void AddFlag(char ch_short_option,
00112                    const std::string& str_long_option,
00113                    const std::string& str_description,
00114                    bool& b_flag) {
00115          m_vecArguments.push_back(
00116             new CArgument<bool>(
00117                ch_short_option,
00118                str_long_option,
00119                str_description,
00120                true,
00121                b_flag));
00122          b_flag = false;
00123       }
00124 
00132       template <typename T> void AddArgument(char ch_short_option,
00133                                              const std::string& str_long_option,
00134                                              const std::string& str_description,
00135                                              T& t_buffer) {
00136          m_vecArguments.push_back(
00137             new CArgument<T>(
00138                ch_short_option,
00139                str_long_option,
00140                str_description,
00141                false,
00142                t_buffer));
00143       }
00144 
00151       virtual void PrintUsage(CARGoSLog& c_log);
00152 
00158       virtual void Parse(SInt32 n_argc,
00159                          char** ppch_argv);
00160 
00161    private:
00162 
00163       void ParseLongOption (SInt32 n_argc,  char** ppch_argv);
00164       void ParseShortOption (SInt32 n_argc, char** ppch_argv);
00165       void ParseShortOptions(SInt32 n_argc, char** ppch_argv);
00166 
00167    private:
00168 
00169       class CAbstractArgument {
00170 
00171       public:
00172 
00173          virtual ~CAbstractArgument() {}
00174          virtual void Parse(const std::string& str_value) = 0;
00175 
00176       public:
00177 
00178          char        ShortOption;
00179          std::string LongOption;
00180          std::string Description;
00181          bool IsFlag;
00182 
00183       };
00184 
00185       template <typename T> class CArgument : public CAbstractArgument {
00186 
00187       public:
00188       
00189          CArgument(char ch_short_option,
00190                    const std::string& str_long_option,
00191                    const std::string& str_description,
00192                    bool b_is_flag,
00193                    T& t_buffer) :
00194             m_tBuffer(t_buffer) {
00195             ShortOption = ch_short_option;
00196             LongOption  = str_long_option;
00197             Description = str_description;
00198             IsFlag      = b_is_flag;
00199          }
00200       
00201          virtual void Parse(const std::string& str_value) {
00202             m_tBuffer = FromString<T>(str_value);
00203          }
00204 
00205          T& m_tBuffer;
00206       };
00207 
00208    private:
00209 
00210       std::vector<CAbstractArgument*> m_vecArguments;
00211       SInt32 m_nCurrentArgument;
00212 
00213    };
00214 
00215 }
00216 
00217 #endif
```

---

Generated on 10 Jul 2018 for ARGoS by 
 1.6.1 
