# Supplementary material for: Iterative improvement in the automatic modular design of robot swarms
Source: PeerJ Comput Sci. 2020 Dec 7;6:e322. doi: 10.7717/peerj-cs.322 (PMC7924708; doi:10.7717/peerj-cs.322)
Supplement: Supplemental Information 3 [file peerj-cs-06-322-s003.zip › argos3/doc/api/standalone/a00354_source.html]

ARGoS: core/utility/configuration/memento.h Source File


- Main Page
- Related Pages
- Namespaces
- Classes
- Files

- File List
- File Members

# core/utility/configuration/memento.h

Go to the documentation of this file.

```
00001 
00013 #ifndef MEMENTO_H
00014 #define MEMENTO_H
00015 
00016 namespace argos {
00017    class CMemento;
00018 }
00019 
00020 #include <argos3/core/utility/datatypes/byte_array.h>
00021 
00022 namespace argos {
00023 
00027    class CMemento {
00028 
00029    public:
00030 
00034       virtual ~CMemento() {}
00035 
00041       virtual void SaveState(CByteArray& c_buffer) = 0;
00042 
00048       virtual void LoadState(CByteArray& c_buffer) = 0;
00049 
00050    };
00051 
00052 }
00053 
00054 #endif
```

---

Generated on 10 Jul 2018 for ARGoS by 
 1.6.1 
