# Supplementary material for: Iterative improvement in the automatic modular design of robot swarms
Source: PeerJ Comput Sci. 2020 Dec 7;6:e322. doi: 10.7717/peerj-cs.322 (PMC7924708; doi:10.7717/peerj-cs.322)
Supplement: Supplemental Information 3 [file peerj-cs-06-322-s003.zip › argos3/doc/api/standalone/a00355.html]

ARGoS: core/utility/datatypes/any.h File Reference


- Main Page
- Related Pages
- Namespaces
- Classes
- Files

- File List
- File Members

# core/utility/datatypes/any.h File Reference

`#include <argos3/core/utility/configuration/argos_exception.h>`  
`#include <cxxabi.h>`  
`#include <typeinfo>`  
`#include <algorithm>`  

Include dependency graph for any.h:

Go to the source code of this file.

|  |  |
| --- | --- |
| Classes | |
| class | argos::CAny |
| class | argos::CAny::CAbstractReference |
|  | Abstract reference to object. More... |
| class | argos::CAny::CReference< T > |
|  | Actual templetized reference to object. More... |
| Namespaces | |
| namespace | argos |

|  |  |
| --- | --- |
|  | The namespace containing all the ARGoS related code. |

| Functions | |
| template<typename T > | |
| T \* | argos::any\_cast (CAny \*pc\_any) |
|  | Performs a cast on the any type to the desired type, when the any type is passed by non-const pointer. |
| template<typename T > | |
| const T \* | argos::any\_cast (const CAny \*pc\_any) |
|  | Performs a cast on the any type to the desired type, when the any type is passed by const pointer. |
| template<typename T > | |
| const T & | argos::any\_cast (const CAny &c\_any) |
|  | Performs a cast on the any type to the desired type, when the any type is passed by const reference. |
| template<typename T > | |
| T & | argos::any\_cast (CAny &c\_any) |
|  | Performs a cast on the any type to the desired type, when the any type is passed by non-const reference. |

---

Generated on 10 Jul 2018 for ARGoS by 
 1.6.1 
