# Supplementary material for: Iterative improvement in the automatic modular design of robot swarms
Source: PeerJ Comput Sci. 2020 Dec 7;6:e322. doi: 10.7717/peerj-cs.322 (PMC7924708; doi:10.7717/peerj-cs.322)
Supplement: Supplemental Information 3 [file peerj-cs-06-322-s003.zip › argos3/doc/api/standalone/a00355_source.html]

ARGoS: core/utility/datatypes/any.h Source File


- Main Page
- Related Pages
- Namespaces
- Classes
- Files

- File List
- File Members

# core/utility/datatypes/any.h

Go to the documentation of this file.

```
00001 
00014 #ifndef ANY_H
00015 #define ANY_H
00016 
00017 #include <argos3/core/utility/configuration/argos_exception.h>
00018 
00019 #include <cxxabi.h>
00020 #include <typeinfo>
00021 #include <algorithm>
00022 
00023 namespace argos {
00024 
00025    class CAny {
00026 
00027    public:
00028 
00032       CAny() :
00033          m_pcRef(NULL) {}
00034 
00038       template<typename T>
00039       CAny(const T& t_object) :
00040          m_pcRef(new CReference<T>(t_object)) {}
00041 
00046       CAny(const CAny& c_any) :
00047          m_pcRef(c_any.m_pcRef ? c_any.m_pcRef->Clone() : NULL) {}
00048 
00052       ~CAny() {
00053          if(m_pcRef != NULL)
00054             delete m_pcRef;
00055       }
00056 
00060       CAny& swap(CAny& c_any) {
00061          std::swap(m_pcRef, c_any.m_pcRef);
00062          return *this;
00063       }
00064 
00068       template <typename T>
00069       CAny& operator=(const T& t_object) {
00070          CAny(t_object).swap(*this);
00071          return *this;
00072       }
00073 
00077       CAny& operator=(const CAny& c_any) {
00078          CAny(c_any).swap(*this);
00079          return *this;
00080       }
00081 
00082    public:
00083 
00087       class CAbstractReference {
00088       public:
00092          virtual ~CAbstractReference() {}
00096          virtual const std::type_info& GetType() const = 0;
00100          virtual CAbstractReference* Clone() const = 0;
00101       };
00102 
00106       template<typename T>
00107       class CReference : public CAbstractReference {
00108       public:
00109          CReference(const T& t_object) : m_tObject(t_object) {}
00113          virtual ~CReference() {}
00117          virtual const std::type_info& GetType() const {
00118             return typeid(T);
00119          }
00123          virtual CAbstractReference* Clone() const {
00124             return new CReference<T>(m_tObject);
00125          }
00126       public:
00127 
00131          T m_tObject;
00132       };
00133       
00134    public:
00135 
00139       CAbstractReference* m_pcRef;
00140 
00141    };
00142 
00147    template<typename T>
00148    T* any_cast(CAny* pc_any) {
00149       /* NULL pointer passed? */
00150       if(pc_any == NULL)
00151          return NULL;
00152       /* OK, Non-NULL pointer; do types match? */
00153       if(pc_any->m_pcRef->GetType() == typeid(T))
00154          /* Types match -> return cast object */
00155          return &(static_cast<CAny::CReference<T>*>(pc_any->m_pcRef))->m_tObject;
00156       else
00157          /* Types don't match -> return NULL */
00158          return NULL;
00159    }
00160 
00165    template<typename T>
00166    const T* any_cast(const CAny* pc_any) {
00167       /* NULL pointer passed? */
00168       if(pc_any == NULL)
00169          return NULL;
00170       /* OK, Non-NULL pointer; do types match? */
00171       if(pc_any->m_pcRef->GetType() == typeid(T))
00172          /* Types match -> return cast object */
00173          return &(static_cast<CAny::CReference<T>*>(pc_any->m_pcRef))->m_tObject;
00174       else
00175          /* Types don't match -> return NULL */
00176          return NULL;
00177    }
00178 
00183    template<typename T>
00184    const T& any_cast(const CAny& c_any) {
00185       /* Fall back to using pass-by-pointer version */
00186       const T* ptResult = any_cast<T>(&c_any);
00187       /* Did the cast succeed? */
00188       if(ptResult != NULL)
00189          return *ptResult;
00190       else {
00191          char* pchDemangledOperandType = abi::__cxa_demangle(c_any.m_pcRef->GetType().name(), NULL, NULL, NULL);
00192          char* pchDemangledTargetType = abi::__cxa_demangle(typeid(T).name(), NULL, NULL, NULL);
00193          THROW_ARGOSEXCEPTION("Failed any_cast conversion from " <<
00194                               pchDemangledOperandType <<
00195                               " to " <<
00196                               pchDemangledTargetType);
00197       }
00198    }
00199 
00204    template<typename T>
00205    T& any_cast(CAny& c_any) {
00206       /* Fall back to using pass-by-pointer version */
00207       T* ptResult = any_cast<T>(&c_any);
00208       /* Did the cast succeed? */
00209       if(ptResult != NULL)
00210          return *ptResult;
00211       else {
00212          char* pchDemangledOperandType = abi::__cxa_demangle(c_any.m_pcRef->GetType().name(), NULL, NULL, NULL);
00213          char* pchDemangledTargetType = abi::__cxa_demangle(typeid(T).name(), NULL, NULL, NULL);
00214          THROW_ARGOSEXCEPTION("Failed any_cast conversion from " <<
00215                               pchDemangledOperandType <<
00216                               " to " <<
00217                               pchDemangledTargetType);
00218       }
00219    }
00220 
00221 }
00222 
00223 #endif
```

---

Generated on 10 Jul 2018 for ARGoS by 
 1.6.1 
