# Supplementary material for: Iterative improvement in the automatic modular design of robot swarms
Source: PeerJ Comput Sci. 2020 Dec 7;6:e322. doi: 10.7717/peerj-cs.322 (PMC7924708; doi:10.7717/peerj-cs.322)
Supplement: Supplemental Information 3 [file peerj-cs-06-322-s003.zip › argos3/doc/api/standalone/a00356_source.html]

ARGoS: core/utility/datatypes/byte\_array.cpp Source File


- Main Page
- Related Pages
- Namespaces
- Classes
- Files

- File List
- File Members

# core/utility/datatypes/byte\_array.cpp

Go to the documentation of this file.

```
00001 
00007 #include "byte_array.h"
00008 
00009 #include <argos3/core/utility/math/general.h>
00010 
00011 #include <arpa/inet.h>
00012 #include <cstdlib>
00013 #include <cstring>
00014 #include <cmath>
00015 
00016 namespace argos {
00017 
00018    /****************************************/
00019    /****************************************/
00020 
00021    static SInt64 MAX_MANTISSA = 9223372036854775806LL; // 2 << 63 - 2;
00022 
00023    /****************************************/
00024    /****************************************/
00025 
00026 #ifndef htonll
00027    static UInt64 htonll(UInt64 un_value) {
00028       /* Define a test variable for endianness - 42 is 'the' answer */
00029       static const SInt32 nTest = 42;
00030       /* Test for host endianness */
00031       if(*reinterpret_cast<const UInt8*>(&nTest) == nTest) {
00032          /* Host is little endian, network is big -> convert to big */
00033          const UInt32 unHighWord = htonl(static_cast<UInt32>(un_value >> 32));
00034          const UInt32 unLowWord  = htonl(static_cast<UInt32>(un_value & 0xFFFFFFFFLL));
00035          return static_cast<UInt64>(unLowWord) << 32 | unHighWord;
00036       }
00037       else {
00038          /* Host is big endian - leave as is */
00039          return un_value;
00040       }
00041    }
00042 #endif
00043 
00044 #ifndef ntohll
00045    static UInt64 ntohll(UInt64 un_value) {
00046       /* Define a test variable for endianness - 42 is 'the' answer */
00047       static const SInt32 nTest = 42;
00048       /* Test for host endianness */
00049       if(*reinterpret_cast<const UInt8*>(&nTest) == nTest) {
00050          /* Host is little endian, network is big -> convert to big */
00051          const UInt32 unHighWord = ntohl(static_cast<UInt32>(un_value >> 32));
00052          const UInt32 unLowWord  = ntohl(static_cast<UInt32>(un_value & 0xFFFFFFFFLL));
00053          return static_cast<UInt64>(unLowWord) << 32 | unHighWord;
00054       }
00055       else {
00056          /* Host is big endian - leave as is */
00057          return un_value;
00058       }
00059    }
00060 #endif
00061    
00062    /****************************************/
00063    /****************************************/
00064 
00065    CByteArray::CByteArray(const UInt8* pun_buffer,
00066                           size_t un_size) {
00067       AddBuffer(pun_buffer, un_size);
00068    }
00069 
00070    /****************************************/
00071    /****************************************/
00072 
00073    CByteArray::CByteArray(size_t un_size,
00074                           UInt8 un_value) {
00075       m_vecBuffer.assign(un_size, un_value);
00076    }
00077 
00078    /****************************************/
00079    /****************************************/
00080 
00081    void CByteArray::Zero() {
00082       ::memset(&m_vecBuffer[0], 0, sizeof(UInt8) * Size());
00083    }
00084 
00085    /****************************************/
00086    /****************************************/
00087 
00088    CByteArray& CByteArray::operator=(const CByteArray& c_byte_array) {
00089       if(this != &c_byte_array) {
00090          m_vecBuffer = c_byte_array.m_vecBuffer;
00091       }
00092       return *this;
00093    }
00094 
00095    /****************************************/
00096    /****************************************/
00097 
00098    bool CByteArray::operator==(const CByteArray& c_byte_array) const {
00099       return m_vecBuffer == c_byte_array.m_vecBuffer;
00100    }
00101 
00102    /****************************************/
00103    /****************************************/
00104 
00105    CByteArray& CByteArray::AddBuffer(const UInt8* pun_buffer,
00106                                      size_t un_size) {      
00107       for(size_t i = 0; i < un_size; ++i) {
00108          m_vecBuffer.push_back(pun_buffer[i]);
00109       }
00110       return *this;
00111    }
00112 
00113    /****************************************/
00114    /****************************************/
00115 
00116    CByteArray& CByteArray::FetchBuffer(UInt8* pun_buffer,
00117                                        size_t un_size) {
00118       if(Size() < un_size) THROW_ARGOSEXCEPTION("Attempting to extract too many bytes from byte array (" << un_size << " requested, " << Size() << " available)");
00119       for(size_t i = 0; i < un_size; ++i) {
00120          *(pun_buffer+i) = m_vecBuffer[i];
00121       }
00122       m_vecBuffer.erase(m_vecBuffer.begin(), m_vecBuffer.begin() + un_size);
00123       return *this;
00124    }
00125 
00126    /****************************************/
00127    /****************************************/
00128 
00129    CByteArray* CByteArray::operator()(size_t un_start,
00130                                       ssize_t un_end) {
00131       if(un_start >= Size()) THROW_ARGOSEXCEPTION("Attempting to extract from byte array beyond the limits (" << un_start << " requested, " << Size() << " size)");
00132       un_end = un_end < 0 ? Size() : Min<ssize_t>(un_end, Size());
00133       return new CByteArray(ToCArray() + un_start, un_end - un_start);
00134    }
00135    
00136    /****************************************/
00137    /****************************************/
00138 
00139    CByteArray& CByteArray::operator<<(UInt8 un_value) {
00140       m_vecBuffer.push_back(un_value);
00141       return *this;
00142    }
00143 
00144    /****************************************/
00145    /****************************************/
00146 
00147    CByteArray& CByteArray::operator>>(UInt8& un_value) {
00148       if(Size() < 1) THROW_ARGOSEXCEPTION("Attempting to extract too many bytes from byte array (1 requested, " << Size() << " available)");
00149       un_value = m_vecBuffer.front();
00150       m_vecBuffer.erase(m_vecBuffer.begin());
00151       return *this;
00152    }
00153 
00154    /****************************************/
00155    /****************************************/
00156 
00157    CByteArray& CByteArray::operator<<(SInt8 n_value) {
00158       m_vecBuffer.push_back(n_value);
00159       return *this;
00160    }
00161 
00162    /****************************************/
00163    /****************************************/
00164 
00165    CByteArray& CByteArray::operator>>(SInt8& n_value) {
00166       if(Size() < 1) THROW_ARGOSEXCEPTION("Attempting to extract too many bytes from byte array (1 requested, " << Size() << " available)");
00167       n_value = m_vecBuffer.front();
00168       m_vecBuffer.erase(m_vecBuffer.begin());
00169       return *this;
00170    }
00171 
00172    /****************************************/
00173    /****************************************/
00174 
00175    CByteArray& CByteArray::operator<<(UInt16 un_value) {
00176       un_value = htons(un_value);
00177       UInt8* punByte = reinterpret_cast<UInt8*>(&un_value);
00178       m_vecBuffer.push_back(punByte[0]);
00179       m_vecBuffer.push_back(punByte[1]);
00180       return *this;
00181    }
00182 
00183    /****************************************/
00184    /****************************************/
00185 
00186    CByteArray& CByteArray::operator>>(UInt16& un_value) {
00187       if(Size() < 2) THROW_ARGOSEXCEPTION("Attempting to extract too many bytes from byte array (2 requested, " << Size() << " available)");
00188       UInt8* punByte = reinterpret_cast<UInt8*>(&un_value);
00189       punByte[0] = m_vecBuffer[0];
00190       punByte[1] = m_vecBuffer[1];
00191       m_vecBuffer.erase(m_vecBuffer.begin(), m_vecBuffer.begin() + 2);
00192       un_value = ntohs(un_value);
00193       return *this;
00194    }
00195 
00196    /****************************************/
00197    /****************************************/
00198 
00199    CByteArray& CByteArray::operator<<(SInt16 n_value) {
00200       n_value = htons(n_value);
00201       UInt8* punByte = reinterpret_cast<UInt8*>(&n_value);
00202       m_vecBuffer.push_back(punByte[0]);
00203       m_vecBuffer.push_back(punByte[1]);
00204       return *this;
00205    }
00206 
00207    /****************************************/
00208    /****************************************/
00209 
00210    CByteArray& CByteArray::operator>>(SInt16& n_value) {
00211       if(Size() < 2) THROW_ARGOSEXCEPTION("Attempting to extract too many bytes from byte array (2 requested, " << Size() << " available)");
00212       UInt8* punByte = reinterpret_cast<UInt8*>(&n_value);
00213       punByte[0] = m_vecBuffer[0];
00214       punByte[1] = m_vecBuffer[1];
00215       m_vecBuffer.erase(m_vecBuffer.begin(), m_vecBuffer.begin() + 2);
00216       n_value = ntohs(n_value);
00217       return *this;
00218    }
00219 
00220    /****************************************/
00221    /****************************************/
00222 
00223    CByteArray& CByteArray::operator<<(UInt32 un_value) {
00224       un_value = htonl(un_value);
00225       UInt8* punByte = reinterpret_cast<UInt8*>(&un_value);
00226       m_vecBuffer.push_back(punByte[0]);
00227       m_vecBuffer.push_back(punByte[1]);
00228       m_vecBuffer.push_back(punByte[2]);
00229       m_vecBuffer.push_back(punByte[3]);
00230       return *this;
00231    }
00232 
00233    /****************************************/
00234    /****************************************/
00235 
00236    CByteArray& CByteArray::operator>>(UInt32& un_value) {
00237       if(Size() < 4) THROW_ARGOSEXCEPTION("Attempting to extract too many bytes from byte array (4 requested, " << Size() << " available)");
00238       UInt8* punByte = reinterpret_cast<UInt8*>(&un_value);
00239       punByte[0] = m_vecBuffer[0];
00240       punByte[1] = m_vecBuffer[1];
00241       punByte[2] = m_vecBuffer[2];
00242       punByte[3] = m_vecBuffer[3];
00243       m_vecBuffer.erase(m_vecBuffer.begin(), m_vecBuffer.begin() + 4);
00244       un_value = ntohl(un_value);
00245       return *this;
00246    }
00247 
00248    /****************************************/
00249    /****************************************/
00250 
00251    CByteArray& CByteArray::operator<<(SInt32 n_value) {
00252       n_value = htonl(n_value);
00253       UInt8* punByte = reinterpret_cast<UInt8*>(&n_value);
00254       m_vecBuffer.push_back(punByte[0]);
00255       m_vecBuffer.push_back(punByte[1]);
00256       m_vecBuffer.push_back(punByte[2]);
00257       m_vecBuffer.push_back(punByte[3]);
00258       return *this;
00259    }
00260 
00261    /****************************************/
00262    /****************************************/
00263 
00264    CByteArray& CByteArray::operator>>(SInt32& n_value) {
00265       if(Size() < 4) THROW_ARGOSEXCEPTION("Attempting to extract too many bytes from byte array (4 requested, " << Size() << " available)");
00266       UInt8* punByte = reinterpret_cast<UInt8*>(&n_value);
00267       punByte[0] = m_vecBuffer[0];
00268       punByte[1] = m_vecBuffer[1];
00269       punByte[2] = m_vecBuffer[2];
00270       punByte[3] = m_vecBuffer[3];
00271       m_vecBuffer.erase(m_vecBuffer.begin(), m_vecBuffer.begin() + 4);
00272       n_value = ntohl(n_value);
00273       return *this;
00274    }
00275 
00276    /****************************************/
00277    /****************************************/
00278 
00279    CByteArray& CByteArray::operator<<(UInt64 un_value) {
00280       un_value = htonll(un_value);
00281       UInt8* punByte = reinterpret_cast<UInt8*>(&un_value);
00282       m_vecBuffer.push_back(punByte[0]);
00283       m_vecBuffer.push_back(punByte[1]);
00284       m_vecBuffer.push_back(punByte[2]);
00285       m_vecBuffer.push_back(punByte[3]);
00286       m_vecBuffer.push_back(punByte[4]);
00287       m_vecBuffer.push_back(punByte[5]);
00288       m_vecBuffer.push_back(punByte[6]);
00289       m_vecBuffer.push_back(punByte[7]);
00290       return *this;
00291    }
00292 
00293    /****************************************/
00294    /****************************************/
00295 
00296    CByteArray& CByteArray::operator>>(UInt64& un_value) {
00297       if(Size() < 8) THROW_ARGOSEXCEPTION("Attempting to extract too many bytes from byte array (8 requested, " << Size() << " available)");         
00298       UInt8* punByte = reinterpret_cast<UInt8*>(&un_value);
00299       punByte[0] = m_vecBuffer[0];
00300       punByte[1] = m_vecBuffer[1];
00301       punByte[2] = m_vecBuffer[2];
00302       punByte[3] = m_vecBuffer[3];
00303       punByte[4] = m_vecBuffer[4];
00304       punByte[5] = m_vecBuffer[5];
00305       punByte[6] = m_vecBuffer[6];
00306       punByte[7] = m_vecBuffer[7];
00307       m_vecBuffer.erase(m_vecBuffer.begin(), m_vecBuffer.begin() + 8);
00308       un_value = ntohll(un_value);
00309       return *this;
00310    }
00311 
00312    /****************************************/
00313    /****************************************/
00314 
00315    CByteArray& CByteArray::operator<<(SInt64 n_value) {
00316       n_value = htonll(n_value);
00317       UInt8* punByte = reinterpret_cast<UInt8*>(&n_value);
00318       m_vecBuffer.push_back(punByte[0]);
00319       m_vecBuffer.push_back(punByte[1]);
00320       m_vecBuffer.push_back(punByte[2]);
00321       m_vecBuffer.push_back(punByte[3]);
00322       m_vecBuffer.push_back(punByte[4]);
00323       m_vecBuffer.push_back(punByte[5]);
00324       m_vecBuffer.push_back(punByte[6]);
00325       m_vecBuffer.push_back(punByte[7]);
00326       return *this;
00327    }
00328 
00329    /****************************************/
00330    /****************************************/
00331 
00332    CByteArray& CByteArray::operator>>(SInt64& n_value) {
00333       if(Size() < 8) THROW_ARGOSEXCEPTION("Attempting to extract too many bytes from byte array (8 requested, " << Size() << " available)");         
00334       UInt8* punByte = reinterpret_cast<UInt8*>(&n_value);
00335       punByte[0] = m_vecBuffer[0];
00336       punByte[1] = m_vecBuffer[1];
00337       punByte[2] = m_vecBuffer[2];
00338       punByte[3] = m_vecBuffer[3];
00339       punByte[4] = m_vecBuffer[4];
00340       punByte[5] = m_vecBuffer[5];
00341       punByte[6] = m_vecBuffer[6];
00342       punByte[7] = m_vecBuffer[7];
00343       m_vecBuffer.erase(m_vecBuffer.begin(), m_vecBuffer.begin() + 8);
00344       n_value = ntohll(n_value);
00345       return *this;
00346    }
00347 
00348    /****************************************/
00349    /****************************************/
00350 
00351    CByteArray& CByteArray::operator<<(unsigned long int un_value) {
00352       if(sizeof(un_value) == sizeof(UInt32)) {
00353          *this << static_cast<UInt32>(un_value);
00354       }
00355       else if(sizeof(un_value) == sizeof(UInt64)) {
00356          *this << static_cast<UInt64>(un_value);
00357       }
00358       return *this;
00359    }
00360 
00361    /****************************************/
00362    /****************************************/
00363 
00364    CByteArray& CByteArray::operator>>(unsigned long int& un_value) {
00365       if(sizeof(un_value) == sizeof(UInt32)) {
00366          *this >> *reinterpret_cast<UInt32*>(&un_value);
00367       }      
00368       else if(sizeof(un_value) == sizeof(UInt64)) {
00369          *this >> *reinterpret_cast<UInt64*>(&un_value);
00370       }      
00371       return *this;
00372    }
00373 
00374    /****************************************/
00375    /****************************************/
00376 
00377    CByteArray& CByteArray::operator<<(signed long int n_value) {
00378       if(sizeof(n_value) == sizeof(SInt32)) {
00379          *this << static_cast<SInt32>(n_value);
00380       }
00381       else if(sizeof(n_value) == sizeof(SInt64)) {
00382          *this << static_cast<SInt64>(n_value);
00383       }
00384       return *this;
00385    }
00386 
00387    /****************************************/
00388    /****************************************/
00389 
00390    CByteArray& CByteArray::operator>>(signed long int& n_value) {
00391       if(sizeof(n_value) == sizeof(SInt32)) {
00392          *this >> *reinterpret_cast<SInt32*>(&n_value);
00393       }      
00394       else if(sizeof(n_value) == sizeof(SInt64)) {
00395          *this >> *reinterpret_cast<SInt64*>(&n_value);
00396       }      
00397       return *this;
00398    }
00399 
00400    /****************************************/
00401    /****************************************/
00402 
00403    CByteArray& CByteArray::operator<<(double f_value) {
00404       /* Buffer for the mantissa */
00405       SInt64 nMantissa;
00406       /* Buffer for the exponent */
00407       SInt32 nExponent;
00408       /* Calculate exponent and shifted significand */
00409       /* The absolute value of the significand is either 0 (when f_value is 0) or 
00410        * in the range [0.5,1). The sign of the significand is the same as f_value.
00411        * The shifted significand is then -0.5 when the significand is 0, and in the
00412        * range [0,0.5) otherwise.
00413        */
00414       double fShiftedSignificand = Abs(frexp(f_value, &nExponent)) - 0.5;
00415       /* Calculate mantissa */
00416       if(fShiftedSignificand < 0.0) {
00417          /* This means f_value was either +0 or -0 */
00418          nMantissa = 0;
00419       }
00420       else {
00421          /* Calculate the mantissa
00422           * Idea:
00423           * - take the shifted significand, which is in [0,0.5)
00424           * - multiply it by 2, so it's in [0,1)
00425           * - multiply this by the maximum value the mantissa can have
00426           * - add 1 to the result to avoid having a zero mantissa
00427           *   (because it maps to a zero double when trasforming back)
00428           */
00429          nMantissa = static_cast<SInt64>(fShiftedSignificand * 2.0 * (MAX_MANTISSA)) + 1;
00430          /* Take care of the sign */
00431          if(f_value < 0.0) {
00432             nMantissa = -nMantissa;
00433          }
00434       }
00435       /* Store exponent and mantissa in the buffer */
00436       *this << nMantissa;
00437       *this << nExponent;
00438       return *this;
00439    }
00440 
00441    /****************************************/
00442    /****************************************/
00443 
00444    CByteArray& CByteArray::operator>>(double& f_value) {
00445       if(Size() < sizeof(SInt64) + sizeof(SInt32)) THROW_ARGOSEXCEPTION("Attempting to extract too many bytes from byte array (" << sizeof(SInt64) + sizeof(SInt32) << " requested, " << Size() << " available)");
00446       /* Buffer for the mantissa */
00447       SInt64 nMantissa;
00448       /* Buffer for the exponent */
00449       SInt32 nExponent;
00450       /* Extract the values from the buffer */
00451       *this >> nMantissa;
00452       *this >> nExponent;
00453       /* Calculate double value */
00454       if(nMantissa == 0) {
00455          /* Special case: zero mantissa */
00456          f_value = 0.0;
00457       }
00458       else {
00459          /* Calculate the significand, whose absolute value must be in the range [0.5,1).
00460           * Idea:
00461           * - take the absolute value of the mantissa, which is in the range [1,MANTISSA_MAX]
00462           * - subtract 1, so it's in [0,MANTISSA_MAX-1]
00463           * - divide by MANTISSA_MAX, so you get a value in [0,1)
00464           * - divide by 2, so it's in [0,0.5)
00465           * - add 0.5, so you end up in [0.5,1)
00466           */
00467          double fSignificand = (static_cast<double>(llabs(nMantissa) - 1) / MAX_MANTISSA) / 2.0 + 0.5;
00468          /* Calculate f_value */
00469          f_value = ldexp(fSignificand, nExponent);
00470          /* Take care of the sign */
00471          if(nMantissa < 0.0) {
00472             f_value = -f_value;
00473          }
00474       }
00475       return *this;
00476    }
00477 
00478    /****************************************/
00479    /****************************************/
00480 
00481    CByteArray& CByteArray::operator<<(float f_value) {
00482       *this << static_cast<double>(f_value);
00483       return *this;
00484    }
00485 
00486    /****************************************/
00487    /****************************************/
00488 
00489    CByteArray& CByteArray::operator>>(float& f_value) {
00490       double fDoubleValue;
00491       *this >> fDoubleValue;
00492       f_value = fDoubleValue;
00493       return *this;
00494    }
00495 
00496    /****************************************/
00497    /****************************************/
00498 
00499    CByteArray& CByteArray::operator<<(const std::string& str_value) {
00500       /* Insert string contents */
00501       for(size_t i = 0; i < str_value.size(); ++i) {
00502          *this << static_cast<UInt8>(str_value[i]);
00503       }
00504       /* Terminate string with a \0 */
00505       *this << static_cast<UInt8>(0);
00506       return *this;
00507    }
00508 
00509    /****************************************/
00510    /****************************************/
00511 
00512    CByteArray& CByteArray::operator>>(std::string& str_value) {
00513       if(Empty()) THROW_ARGOSEXCEPTION("Attempting to extract values from empty byte array");
00514       str_value.clear();
00515       size_t i = 0;
00516       while(i < Size() && m_vecBuffer[i] != '\0') {
00517          str_value += m_vecBuffer[i];
00518          ++i;
00519       }
00520       if(m_vecBuffer[i] == '\0') {
00521          ++i;
00522       }
00523       m_vecBuffer.erase(m_vecBuffer.begin(), m_vecBuffer.begin() + i);
00524       return *this;
00525    }
00526 
00527    /****************************************/
00528    /****************************************/
00529 
00530    std::ostream& operator<<(std::ostream& c_os, const CByteArray& c_byte_array) {
00531       c_os << "CByteArray [";
00532       for(size_t i = 0; i < c_byte_array.Size(); ++i) {
00533          c_os << " " << c_byte_array.m_vecBuffer[i];
00534       }
00535       c_os << " ]" << std::endl;
00536       return c_os;
00537    }
00538 
00539    /****************************************/
00540    /****************************************/
00541 
00542 }
```

---

Generated on 10 Jul 2018 for ARGoS by 
 1.6.1 
