# Supplementary material for: Iterative improvement in the automatic modular design of robot swarms
Source: PeerJ Comput Sci. 2020 Dec 7;6:e322. doi: 10.7717/peerj-cs.322 (PMC7924708; doi:10.7717/peerj-cs.322)
Supplement: Supplemental Information 3 [file peerj-cs-06-322-s003.zip › argos3/doc/api/standalone/a00357_source.html]

ARGoS: core/utility/datatypes/byte\_array.h Source File


- Main Page
- Related Pages
- Namespaces
- Classes
- Files

- File List
- File Members

# core/utility/datatypes/byte\_array.h

Go to the documentation of this file.

```
00001 
00011 #ifndef BYTE_ARRAY_H
00012 #define BYTE_ARRAY_H
00013 
00014 #include <argos3/core/utility/datatypes/datatypes.h>
00015 #include <argos3/core/utility/configuration/argos_exception.h>
00016 #include <vector>
00017 #include <iterator>
00018 #include <unistd.h>
00019 
00020 namespace argos {
00021 
00028    class CByteArray {
00029 
00030    public:
00031 
00035       CByteArray() {}
00036 
00040       CByteArray(const CByteArray& c_byte_array) :
00041          m_vecBuffer(c_byte_array.m_vecBuffer) {}
00042 
00050       CByteArray(const UInt8* pun_buffer,
00051                  size_t un_size);
00052 
00059       CByteArray(size_t un_size,
00060                  UInt8 un_value = 0);
00061 
00066       inline size_t Size() const {
00067          return m_vecBuffer.size();
00068       }
00069 
00083       inline void Resize(size_t un_size,
00084                          UInt8 un_value = 0) {
00085          m_vecBuffer.resize(un_size, un_value);
00086       }
00087 
00092       inline void Swap(CByteArray& c_other) {
00093          m_vecBuffer.swap(c_other.m_vecBuffer);
00094       }
00095 
00100       inline bool Empty() const {
00101          return m_vecBuffer.empty();
00102       }
00103 
00112       inline const UInt8* ToCArray() const {
00113          return !Empty() ? &m_vecBuffer[0] : NULL;
00114       }
00115 
00124       inline UInt8* ToCArray() {
00125          return !Empty() ? &m_vecBuffer[0] : NULL;
00126       }
00127 
00134       inline void Clear() {
00135          m_vecBuffer.clear();
00136       }
00137 
00143       void Zero();
00144 
00149       CByteArray& operator=(const CByteArray& c_byte_array);
00150 
00157       inline UInt8& operator[](size_t un_index) {
00158          if(un_index >= Size()) THROW_ARGOSEXCEPTION("CByteArray: index out of bounds [index = " << un_index << ", size=" << Size() << "]");
00159          return m_vecBuffer.at(un_index);
00160       }
00161 
00168       inline UInt8 operator[](size_t un_index) const {
00169          if(un_index >= Size()) THROW_ARGOSEXCEPTION("CByteArray: index out of bounds [index = " << un_index << ", size=" << Size() << "]");
00170          return m_vecBuffer.at(un_index);
00171       }
00172 
00177       bool operator==(const CByteArray& c_byte_array) const;
00178 
00186       CByteArray& AddBuffer(const UInt8* pun_buffer,
00187                             size_t un_size);
00188 
00196       CByteArray& FetchBuffer(UInt8* pun_buffer,
00197                               size_t un_size);
00198 
00209       template<typename T> T PopFront() {
00210          T tRetVal;
00211          *this >> tRetVal;
00212          return tRetVal;
00213       }
00214 
00223       CByteArray* operator()(size_t un_start,
00224                              ssize_t un_end = -1);
00225 
00231       CByteArray& operator<<(UInt8 un_value);
00232 
00239       CByteArray& operator>>(UInt8& un_value);
00240 
00246       CByteArray& operator<<(SInt8 n_value);
00247 
00254       CByteArray& operator>>(SInt8& n_value);
00255 
00261       CByteArray& operator<<(UInt16 un_value);
00262 
00269       CByteArray& operator>>(UInt16& un_value);
00270 
00276       CByteArray& operator<<(SInt16 n_value);
00277 
00284       CByteArray& operator>>(SInt16& n_value);
00285 
00291       CByteArray& operator<<(UInt32 un_value);
00292 
00299       CByteArray& operator>>(UInt32& un_value);
00300 
00306       CByteArray& operator<<(SInt32 n_value);
00307 
00314       CByteArray& operator>>(SInt32& n_value);
00315 
00321       CByteArray& operator<<(UInt64 un_value);
00322 
00329       CByteArray& operator>>(UInt64& un_value);
00330 
00336       CByteArray& operator<<(SInt64 n_value);
00337 
00344       CByteArray& operator>>(SInt64& n_value);
00345 
00354       CByteArray& operator<<(unsigned long int un_value);
00355 
00365       CByteArray& operator>>(unsigned long int& un_value);
00366 
00375       CByteArray& operator<<(signed long int n_value);
00376 
00386       CByteArray& operator>>(signed long int& n_value);
00387 
00395       CByteArray& operator<<(double f_value);
00396 
00406       CByteArray& operator>>(double& f_value);
00407 
00415       CByteArray& operator<<(float f_value);
00416 
00426       CByteArray& operator>>(float& f_value);
00427 
00433       CByteArray& operator<<(const std::string& str_value);
00434 
00441       CByteArray& operator>>(std::string& str_value);
00442 
00450       friend std::ostream& operator<<(std::ostream& c_os, const CByteArray& c_byte_array);
00451 
00452    private:
00453 
00454       std::vector<UInt8> m_vecBuffer;
00455 
00456    };
00457 
00458 }
00459 
00460 #endif
```

---

Generated on 10 Jul 2018 for ARGoS by 
 1.6.1 
