# Supplementary material for: Iterative improvement in the automatic modular design of robot swarms
Source: PeerJ Comput Sci. 2020 Dec 7;6:e322. doi: 10.7717/peerj-cs.322 (PMC7924708; doi:10.7717/peerj-cs.322)
Supplement: Supplemental Information 3 [file peerj-cs-06-322-s003.zip › argos3/doc/api/standalone/a00358_source.html]

ARGoS: core/utility/datatypes/color.cpp Source File


- Main Page
- Related Pages
- Namespaces
- Classes
- Files

- File List
- File Members

# core/utility/datatypes/color.cpp

Go to the documentation of this file.

```
00001 
00009 #include "color.h"
00010 
00011 namespace argos {
00012 
00013    CColor CColor::BLACK(0,0,0);
00014    CColor CColor::WHITE(255, 255, 255);
00015    CColor CColor::RED(255,0,0);
00016    CColor CColor::GREEN(0,255,0);
00017    CColor CColor::BLUE(0,0,255);
00018    CColor CColor::MAGENTA(255,0,255);
00019    CColor CColor::CYAN(0,255,255);
00020    CColor CColor::YELLOW(255,255,0);
00021    CColor CColor::ORANGE(255,140,0);
00022    CColor CColor::BROWN(165,42,42);
00023    CColor CColor::PURPLE(160,32,240);
00024    CColor CColor::GRAY10(26,26,26);
00025    CColor CColor::GRAY20(51,51,51);
00026    CColor CColor::GRAY30(77,77,77);
00027    CColor CColor::GRAY40(102,102,102);
00028    CColor CColor::GRAY50(127,127,127);
00029    CColor CColor::GRAY60(153,153,153);
00030    CColor CColor::GRAY70(179,179,179);
00031    CColor CColor::GRAY80(204,204,204);
00032    CColor CColor::GRAY90(229,229,229);
00033 
00034 }
```

---

Generated on 10 Jul 2018 for ARGoS by 
 1.6.1 
