# Supplementary material for: Iterative improvement in the automatic modular design of robot swarms
Source: PeerJ Comput Sci. 2020 Dec 7;6:e322. doi: 10.7717/peerj-cs.322 (PMC7924708; doi:10.7717/peerj-cs.322)
Supplement: Supplemental Information 3 [file peerj-cs-06-322-s003.zip › argos3/doc/api/standalone/a00359_source.html]

ARGoS: core/utility/datatypes/color.h Source File


- Main Page
- Related Pages
- Namespaces
- Classes
- Files

- File List
- File Members

# core/utility/datatypes/color.h

Go to the documentation of this file.

```
00001 
00011 #ifndef COLOR_H
00012 #define COLOR_H
00013 
00014 #include <argos3/core/utility/datatypes/datatypes.h>
00015 #include <argos3/core/utility/string_utilities.h>
00016 #include <string.h>
00017 #include <iostream>
00018 
00019 namespace argos {
00020 
00025    class CColor {
00026 
00027    public:
00028 
00029       static CColor BLACK;
00030       static CColor WHITE;
00031       static CColor RED;
00032       static CColor GREEN;
00033       static CColor BLUE;
00034       static CColor MAGENTA;
00035       static CColor CYAN;
00036       static CColor YELLOW;
00037       static CColor ORANGE;
00038       static CColor BROWN;
00039       static CColor PURPLE;
00040       static CColor GRAY10;
00041       static CColor GRAY20;
00042       static CColor GRAY30;
00043       static CColor GRAY40;
00044       static CColor GRAY50;
00045       static CColor GRAY60;
00046       static CColor GRAY70;
00047       static CColor GRAY80;
00048       static CColor GRAY90;
00049 
00053       CColor() {}
00054 
00058       explicit CColor(UInt8 un_red,
00059                       UInt8 un_green,
00060                       UInt8 un_blue,
00061                       UInt8 un_alpha = 255) throw() :
00062          m_tChannels(un_red, un_green, un_blue, un_alpha) {}
00063 
00068       inline Real ToGrayScale() const throw() {
00069          return
00070             0.299f * m_tChannels.m_unRed +
00071             0.587f * m_tChannels.m_unGreen +
00072             0.114f * m_tChannels.m_unBlue;
00073       }
00074 
00079       inline UInt8 GetRed() const throw() { return m_tChannels.m_unRed; }
00084       inline void SetRed(UInt8 un_red) throw() { m_tChannels.m_unRed = un_red; }
00085 
00090       inline UInt8 GetGreen() const throw() { return m_tChannels.m_unGreen; }
00095       inline void SetGreen(UInt8 un_green) throw() { m_tChannels.m_unGreen = un_green; }
00096 
00101       inline UInt8 GetBlue() const throw() { return m_tChannels.m_unBlue; }
00106       inline void SetBlue(UInt8 un_blue) throw() { m_tChannels.m_unBlue = un_blue; }
00107 
00112       inline UInt8 GetAlpha() const throw() { return m_tChannels.m_unAlpha; }
00117       inline void SetAlpha(UInt8 un_alpha) throw() { m_tChannels.m_unAlpha = un_alpha; }
00118 
00126       inline void Set(UInt8 un_red,
00127                       UInt8 un_green,
00128                       UInt8 un_blue,
00129                       UInt8 un_alpha = 255) throw() {
00130          SetRed(un_red);
00131          SetGreen(un_green);
00132          SetBlue(un_blue);
00133          SetAlpha(un_alpha);
00134       }
00135 
00143       inline void Set(const std::string& str_color) {
00144          try {
00145             if      (str_color == "black")   *this = CColor::BLACK;
00146             else if (str_color == "white")   *this = CColor::WHITE;
00147             else if (str_color == "red")     *this = CColor::RED;
00148             else if (str_color == "green")   *this = CColor::GREEN;
00149             else if (str_color == "blue")    *this = CColor::BLUE;
00150             else if (str_color == "magenta") *this = CColor::MAGENTA;
00151             else if (str_color == "cyan")    *this = CColor::CYAN;
00152             else if (str_color == "yellow")  *this = CColor::YELLOW;
00153             else if (str_color == "orange")  *this = CColor::ORANGE;
00154             else if (str_color == "brown")   *this = CColor::BROWN;
00155             else if (str_color == "purple")  *this = CColor::PURPLE;
00156             else if (str_color == "gray10")  *this = CColor::GRAY10;
00157             else if (str_color == "gray20")  *this = CColor::GRAY20;
00158             else if (str_color == "gray30")  *this = CColor::GRAY30;
00159             else if (str_color == "gray40")  *this = CColor::GRAY40;
00160             else if (str_color == "gray50")  *this = CColor::GRAY50;
00161             else if (str_color == "gray60")  *this = CColor::GRAY60;
00162             else if (str_color == "gray70")  *this = CColor::GRAY70;
00163             else if (str_color == "gray80")  *this = CColor::GRAY80;
00164             else if (str_color == "gray90")  *this = CColor::GRAY90;
00165             else {
00166                UInt16 unValues[4];
00167                ParseValues<UInt16>(str_color, 4, unValues, ',');
00168                for(UInt16 i = 0; i < 4; ++i) {
00169                   if(unValues[i] > 255) {
00170                      THROW_ARGOSEXCEPTION("Color value " << unValues[i] << " is larger than 255.");
00171                   }
00172                }
00173                Set(unValues[0], unValues[1], unValues[2], unValues[3]);
00174             }
00175          }
00176          catch(CARGoSException& ex) {
00177             THROW_ARGOSEXCEPTION_NESTED("Error while parsing color input string", ex);
00178          }
00179       }
00180 
00185       inline operator UInt32() {
00186          return *reinterpret_cast<UInt32*>(&m_tChannels);
00187       }
00188 
00194       inline bool operator==(const CColor& c_color2) const throw() {
00195          return m_tChannels == c_color2.m_tChannels;
00196       }
00197 
00203       inline bool operator!=(const CColor& c_color2) const throw() {
00204          return m_tChannels != c_color2.m_tChannels;
00205       }
00206 
00218       friend std::ostream& operator<<(std::ostream& os,
00219                                       const CColor& c_color) {
00220          if      (c_color == CColor::BLACK)   os << "black";
00221          else if (c_color == CColor::WHITE)   os << "white";
00222          else if (c_color == CColor::RED)     os << "red";
00223          else if (c_color == CColor::GREEN)   os << "green";
00224          else if (c_color == CColor::BLUE)    os << "blue";
00225          else if (c_color == CColor::MAGENTA) os << "magenta";
00226          else if (c_color == CColor::CYAN)    os << "cyan";
00227          else if (c_color == CColor::YELLOW)  os << "yellow";
00228          else if (c_color == CColor::ORANGE)  os << "orange";
00229          else if (c_color == CColor::BROWN)   os << "brown";
00230          else if (c_color == CColor::PURPLE)  os << "purple";
00231          else if (c_color == CColor::GRAY10)  os << "gray10";
00232          else if (c_color == CColor::GRAY20)  os << "gray20";
00233          else if (c_color == CColor::GRAY30)  os << "gray30";
00234          else if (c_color == CColor::GRAY40)  os << "gray40";
00235          else if (c_color == CColor::GRAY50)  os << "gray50";
00236          else if (c_color == CColor::GRAY60)  os << "gray60";
00237          else if (c_color == CColor::GRAY70)  os << "gray70";
00238          else if (c_color == CColor::GRAY80)  os << "gray80";
00239          else if (c_color == CColor::GRAY90)  os << "gray90";
00240          else {
00241             os << c_color.m_tChannels.m_unRed
00242                << "," << c_color.m_tChannels.m_unGreen
00243                << "," << c_color.m_tChannels.m_unBlue
00244                << "," << c_color.m_tChannels.m_unAlpha;
00245          }
00246          return os;
00247       }
00248 
00258       friend std::istream& operator>>(std::istream& is,
00259                                       CColor& c_color) {
00260          std::string strColor;
00261          is >> strColor;
00262          c_color.Set(strColor);
00263          return is;
00264       }
00265 
00266 
00267    private:
00268 
00269       struct TChannels {
00270          UInt8 m_unRed;
00271          UInt8 m_unGreen;
00272          UInt8 m_unBlue;
00273          UInt8 m_unAlpha;
00274 
00275          TChannels() :
00276             m_unRed(0),
00277             m_unGreen(0),
00278             m_unBlue(0),
00279             m_unAlpha(255) {}
00280 
00281          TChannels(const UInt8 un_red,
00282                    const UInt8 un_green,
00283                    const UInt8 un_blue,
00284                    const UInt8 un_alpha = 255) :
00285             m_unRed(un_red),
00286             m_unGreen(un_green),
00287             m_unBlue(un_blue),
00288             m_unAlpha(un_alpha) {}
00289 
00290          inline bool operator==(const TChannels& t_channels) const {
00291             return
00292                (m_unRed == t_channels.m_unRed) &&
00293                (m_unGreen == t_channels.m_unGreen) &&
00294                (m_unBlue == t_channels.m_unBlue) &&
00295                (m_unAlpha == t_channels.m_unAlpha);
00296          }
00297 
00298          inline bool operator!=(const TChannels& t_channels) const {
00299             return
00300                (m_unRed != t_channels.m_unRed) ||
00301                (m_unGreen != t_channels.m_unGreen) ||
00302                (m_unBlue != t_channels.m_unBlue) ||
00303                (m_unAlpha != t_channels.m_unAlpha);
00304          }
00305 
00306       } m_tChannels;
00307 
00308    };
00309 
00310 }
00311 
00312 #endif
```

---

Generated on 10 Jul 2018 for ARGoS by 
 1.6.1 
