# Supplementary material for: Iterative improvement in the automatic modular design of robot swarms
Source: PeerJ Comput Sci. 2020 Dec 7;6:e322. doi: 10.7717/peerj-cs.322 (PMC7924708; doi:10.7717/peerj-cs.322)
Supplement: Supplemental Information 3 [file peerj-cs-06-322-s003.zip › argos3/doc/api/standalone/a00360.html]

ARGoS: core/utility/datatypes/datatypes.h File Reference


- Main Page
- Related Pages
- Namespaces
- Classes
- Files

- File List
- File Members

# core/utility/datatypes/datatypes.h File Reference

`#include <argos3/core/config.h>`  

Include dependency graph for datatypes.h:

Go to the source code of this file.

|  |  |
| --- | --- |
| Typedefs | |
| typedef float | Real |
|  | Collects all ARGoS code. |
| typedef signed char | SInt8 |
|  | 8-bit signed integer. |
| typedef unsigned char | UInt8 |
|  | 8-bit unsigned integer. |
| typedef signed short | SInt16 |
|  | 16-bit signed integer. |
| typedef unsigned short | UInt16 |
|  | 16-bit unsigned integer. |
| typedef signed int | SInt32 |
|  | 32-bit signed integer. |
| typedef unsigned int | UInt32 |
|  | 32-bit unsigned integer. |
| typedef signed long long | SInt64 |
|  | 64-bit signed integer. |
| typedef unsigned long long | UInt64 |
|  | 64-bit unsigned integer. |

---

## Typedef Documentation

|  |
| --- |
| typedef float Real |

Collects all ARGoS code.

The basic floating point type in ARGoS. Real is defined either as `float` or `double`, depending on the flags set for compilation.

Definition at line 39 of file datatypes.h.

|  |
| --- |
| typedef signed short SInt16 |

16-bit signed integer.

Definition at line 74 of file datatypes.h.

|  |
| --- |
| typedef signed int SInt32 |

32-bit signed integer.

Definition at line 93 of file datatypes.h.

|  |
| --- |
| typedef signed long long SInt64 |

64-bit signed integer.

Definition at line 103 of file datatypes.h.

|  |
| --- |
| typedef signed char SInt8 |

8-bit signed integer.

Definition at line 45 of file datatypes.h.

|  |
| --- |
| typedef unsigned short UInt16 |

16-bit unsigned integer.

Definition at line 78 of file datatypes.h.

|  |
| --- |
| typedef unsigned int UInt32 |

32-bit unsigned integer.

Definition at line 97 of file datatypes.h.

|  |
| --- |
| typedef unsigned long long UInt64 |

64-bit unsigned integer.

Definition at line 107 of file datatypes.h.

|  |
| --- |
| typedef unsigned char UInt8 |

8-bit unsigned integer.

Definition at line 60 of file datatypes.h.

---

Generated on 10 Jul 2018 for ARGoS by 
 1.6.1 
