# Supplementary material for: Iterative improvement in the automatic modular design of robot swarms
Source: PeerJ Comput Sci. 2020 Dec 7;6:e322. doi: 10.7717/peerj-cs.322 (PMC7924708; doi:10.7717/peerj-cs.322)
Supplement: Supplemental Information 3 [file peerj-cs-06-322-s003.zip › argos3/doc/api/standalone/a00360_source.html]

ARGoS: core/utility/datatypes/datatypes.h Source File


- Main Page
- Related Pages
- Namespaces
- Classes
- Files

- File List
- File Members

# core/utility/datatypes/datatypes.h

Go to the documentation of this file.

```
00001 
00015 #ifndef DATATYPES_H
00016 #define DATATYPES_H
00017 
00018 #include <argos3/core/config.h>
00019 
00020 #ifdef __cplusplus
00021 #include <iostream>
00022 #endif
00023 
00027 #ifdef __cplusplus
00028 namespace argos {
00029 #endif
00030     
00036 #ifdef ARGOS_USE_DOUBLE
00037    typedef double Real;
00038 #else
00039    typedef float Real;
00040 #endif
00041 
00045    typedef signed char SInt8;
00046 
00047 #ifdef __cplusplus
00048 
00052    inline std::ostream& operator<<(std::ostream& c_os, const SInt8 n_value) {
00053       c_os << static_cast<signed int>(n_value);
00054       return c_os;
00055    }
00056 #endif
00057 
00060    typedef unsigned char UInt8;
00061 #ifdef __cplusplus
00062 
00066    inline std::ostream& operator<<(std::ostream& c_os, const UInt8 un_value) {
00067       c_os << static_cast<unsigned int>(un_value);
00068       return c_os;
00069    }
00070 #endif
00071 
00074    typedef signed short SInt16;
00078    typedef unsigned short UInt16;
00079 
00080 #ifdef __dsPIC30
00081 
00084    typedef signed long int SInt32;
00088    typedef unsigned long int UInt32;
00089 #else
00090 
00093    typedef signed int SInt32;
00097    typedef unsigned int UInt32;
00098 #endif
00099    
00103    typedef signed long long SInt64;
00107    typedef unsigned long long UInt64;
00108    
00109    
00110 #ifdef __cplusplus
00111 }
00112 #endif
00113 
00114 #endif
```

---

Generated on 10 Jul 2018 for ARGoS by 
 1.6.1 
