# Supplementary material for: Iterative improvement in the automatic modular design of robot swarms
Source: PeerJ Comput Sci. 2020 Dec 7;6:e322. doi: 10.7717/peerj-cs.322 (PMC7924708; doi:10.7717/peerj-cs.322)
Supplement: Supplemental Information 3 [file peerj-cs-06-322-s003.zip › argos3/doc/api/standalone/a00361_source.html]

ARGoS: core/utility/datatypes/set.h Source File


- Main Page
- Related Pages
- Namespaces
- Classes
- Files

- File List
- File Members

# core/utility/datatypes/set.h

Go to the documentation of this file.

```
00001 
00007 #ifndef SET_H
00008 #define SET_H
00009 
00010 #include <cstddef>
00011 #include <iterator>
00012 
00013 namespace argos {
00014 
00019    template <class T>
00020    struct SSetElement {
00021       T Data;
00022       SSetElement* Previous;
00023       SSetElement* Next;
00024       
00025       SSetElement(const T& t_data,
00026                   SSetElement* ps_prev = NULL,
00027                   SSetElement* ps_next = NULL) :
00028          Data(t_data),
00029          Previous(ps_prev),
00030          Next(ps_next) {}
00031    };
00032 
00038    template<class CONTAINED_TYPE, class REFERENCED_TYPE>
00039    class CSetIterator {
00040 
00041    public:
00042 
00043       typedef std::forward_iterator_tag iterator_category;
00044       typedef REFERENCED_TYPE value_type;
00045       typedef std::ptrdiff_t difference_type;
00046       typedef REFERENCED_TYPE& reference;
00047       typedef REFERENCED_TYPE* pointer;
00048 
00049    public:
00050 
00051       CSetIterator(SSetElement<CONTAINED_TYPE>* ps_elem = NULL) :
00052          m_psElem(ps_elem) {}
00053 
00054       CSetIterator(const CSetIterator& c_it) :
00055          m_psElem(c_it.m_psElem) {}
00056 
00057       CSetIterator& operator=(const CSetIterator& c_it) {
00058          if(this != &c_it) {
00059             m_psElem = c_it.m_psElem;
00060          }
00061          return *this;
00062       }
00063 
00064       reference operator*() {
00065          return m_psElem->Data;
00066       }
00067 
00068       pointer operator->() {
00069          return &(m_psElem->Data);
00070       }
00071 
00072       CSetIterator& operator++() {
00073          m_psElem = m_psElem->Next;
00074          return *this;
00075       }
00076 
00077       bool operator==(const CSetIterator& c_it) {
00078          return (m_psElem == c_it.m_psElem);
00079       }
00080 
00081       bool operator!=(const CSetIterator& c_it) {
00082          return (m_psElem != c_it.m_psElem);
00083       }
00084 
00085       SSetElement<CONTAINED_TYPE>* m_psElem;
00086 
00087    };
00088 
00099    template <class T>
00100    class CSet {
00101 
00102    public:
00103 
00104       typedef CSetIterator<T, T> iterator;
00105 
00106       class const_iterator : public CSetIterator<T, const T> {
00107       public:
00108          const_iterator(const iterator& c_it) : CSetIterator<T, const T>(c_it.m_psElem) {}
00109          const_iterator& operator=(const iterator& c_it) {
00110             CSetIterator<T, const T>::m_psElem = c_it.m_psElem;
00111             return *this;
00112          }
00113          bool operator==(const iterator& c_it) { return (CSetIterator<T, const T>::m_psElem == c_it.m_psElem); }
00114          bool operator!=(const iterator& c_it) { return (CSetIterator<T, const T>::m_psElem != c_it.m_psElem); }
00115       };
00116 
00117    public:
00118 
00123       CSet() :
00124          m_psFirst(NULL),
00125          m_psLast(NULL),
00126          m_unSize(0) {}
00127 
00133       CSet(const CSet& c_set) :
00134          m_psFirst(NULL),
00135          m_psLast(NULL),
00136          m_unSize(0) {
00137          *this = c_set;
00138       }
00139 
00143       ~CSet() {
00144          clear();
00145       }
00146 
00152       CSet& operator=(const CSet& c_set) {
00153          /* Is the passed set a different set? */
00154          if(this != &c_set) {
00155             /* Yes, copy from it */
00156             /* First, erase this set's contents */
00157             clear();
00158             /* Is the other set empty? */
00159             if(! c_set.empty()) {
00160                /* Not empty, there's at least one element to copy */
00161                /* Start by copying the size of the other set into this one */
00162                m_unSize = c_set.m_unSize;
00163                /* Create the first element */
00164                m_psFirst = new SSetElement<T>(c_set.m_psFirst->Data);
00165                /* Is the size of the other set 1? */
00166                if(m_unSize == 1) {
00167                   /* Yes, the first element is also the last one */
00168                   m_psLast = m_psFirst;
00169                }
00170                else {
00171                   /* There's more than just one element */
00172                   /* Copy all the elements starting from the second */
00173                   /* Current element on other set to be copied */
00174                   SSetElement<T>* psCurElemOnOther = c_set.m_psFirst->Next;
00175                   /* Last copied element on this set */
00176                   SSetElement<T>* psLastElemOnThis = m_psFirst;
00177                   /* Current element on this set being created */
00178                   SSetElement<T>* psCurElemOnThis = NULL;
00179                   /* Go on until we hit the end of the list on the other set */
00180                   while(psCurElemOnOther != NULL) {
00181                      /* Create a new element for this set, setting as previous psLastElemOnThis */
00182                      psCurElemOnThis = new SSetElement<T>(psCurElemOnOther->Data, psLastElemOnThis);
00183                      /* Set the next of psLastElemOnThis to the element just created */
00184                      psLastElemOnThis->Next = psCurElemOnThis;
00185                      /* Advance with the last element on this */
00186                      psLastElemOnThis = psCurElemOnThis;
00187                      /* Advance with the element to copy on the other set */
00188                      psCurElemOnOther = psCurElemOnOther->Next;
00189                   }
00190                   /* At this point, psCurElemOnThis corresponds to the last element of the list */
00191                   m_psLast = psCurElemOnThis;
00192                }
00193             }
00194          }
00195          return *this;
00196       }
00197 
00202       inline bool empty() const {
00203          return m_unSize == 0;
00204       }
00205 
00210       inline size_t size() const {
00211          return m_unSize;
00212       }
00213 
00214       inline T& first() {
00215          return m_psFirst->Data;
00216       }
00217 
00218       inline const T& first() const {
00219          return m_psFirst->Data;
00220       }
00221 
00222       inline T& last() {
00223          return m_psLast->Data;
00224       }
00225 
00226       inline const T& last() const {
00227          return m_psLast->Data;
00228       }
00229 
00235       void insert(const T& t_element) {
00236          /* Is the list empty? */
00237          if(m_unSize == 0) {
00238             /* Yes, the first and last element coincide */
00239             m_psFirst = new SSetElement<T>(t_element);
00240             m_psLast = m_psFirst;
00241             m_unSize = 1;
00242          }
00243          else {
00244             /* No, we have at least 1 element */
00245             /* Search for the element in the list that will be the next
00246                of the element to add */
00247             SSetElement<T>* psNextElem = m_psFirst;
00248             while(psNextElem != NULL &&
00249                   psNextElem->Data < t_element) {
00250                psNextElem = psNextElem->Next;
00251             }
00252             /* Did we get to the end of the list? */
00253             if(psNextElem == NULL) {
00254                /* Yes, add the new element after the last one */
00255                SSetElement<T>* psNewElem = new SSetElement<T>(t_element, m_psLast);
00256                m_psLast->Next = psNewElem;
00257                m_psLast = psNewElem;
00258                ++m_unSize;
00259                return;
00260             }
00261             /* Is the element already present? */
00262             if(psNextElem->Data == t_element) {
00263                /* Yes, nothing to add */
00264                return;
00265             }
00266             /* Is the next element the first in the list? */
00267             if(psNextElem == m_psFirst) {
00268                /* Yes, we must add the new element as the new first */
00269                SSetElement<T>* psNewElem = new SSetElement<T>(t_element, NULL, m_psFirst);
00270                m_psFirst->Previous = psNewElem;
00271                m_psFirst = psNewElem;
00272                ++m_unSize;
00273                return;
00274             }
00275             /* If we get here, it's because we have to add the new element in the middle */
00276             SSetElement<T>* psNewElem = new SSetElement<T>(t_element, psNextElem->Previous, psNextElem);
00277             psNextElem->Previous->Next = psNewElem;
00278             psNextElem->Previous = psNewElem;
00279             ++m_unSize;
00280          }
00281       }
00282 
00287       void erase(const T& t_element) {
00288          /* Is the list empty? */
00289          if(m_unSize == 0) {
00290             /* Yes, nothing to do */
00291             return;
00292          }
00293          /* Is the list composed of a single element? */
00294          if(m_unSize == 1) {
00295             /* Is that the element we want to eliminate? */
00296             if(m_psFirst->Data == t_element) {
00297                /* Yes, erase it! */
00298                delete m_psFirst;
00299                m_psFirst = NULL;
00300                m_psLast = NULL;
00301                m_unSize = 0;
00302             }
00303             return;
00304          }
00305          /* If we get here, it's because the trivial cases
00306             don't apply */
00307          /* Look for the passed element */
00308          SSetElement<T>* psElem = find_impl(t_element);
00309          /* Did we find it? */
00310          if(psElem != NULL) {
00311             /* Yes, let's erase it */
00312             /* Are we removing the first element? */
00313             if(psElem == m_psFirst) {
00314                /* Yes, we need to update m_psFirst */
00315                m_psFirst = m_psFirst->Next;
00316                m_psFirst->Previous = NULL;
00317                delete psElem;
00318                --m_unSize;
00319                return;
00320             }
00321             /* Are we removing the last element? */
00322             if(psElem == m_psLast) {
00323                /* Yes, we need to update m_psLast */
00324                m_psLast = m_psLast->Previous;
00325                m_psLast->Next = NULL;
00326                delete psElem;
00327                --m_unSize;
00328                return;
00329             }
00330             /* If we get here, it's because we need to remove
00331                an element in the middle */
00332             psElem->Previous->Next = psElem->Next;
00333             psElem->Next->Previous = psElem->Previous;
00334             delete psElem;
00335             --m_unSize;
00336          }
00337       }
00338 
00343       inline void erase(iterator& c_it) {
00344          erase(*c_it);
00345       }
00346 
00350       void clear() {
00351          if(m_unSize == 0) {
00352             return;
00353          }
00354          if(m_unSize == 1) {
00355             delete m_psFirst;
00356             m_psFirst = NULL;
00357             m_psLast = NULL;
00358             m_unSize = 0;
00359             return;
00360          }
00361          SSetElement<T>* psCurElem = m_psFirst;
00362          SSetElement<T>* psNextElem = psCurElem->Next;
00363          while(psCurElem != NULL) {
00364             delete psCurElem;
00365             psCurElem = psNextElem;
00366             if(psCurElem != NULL) {
00367                psNextElem = psNextElem->Next;
00368             }
00369          }
00370          m_psFirst = NULL;
00371          m_psLast = NULL;
00372          m_unSize = 0;
00373       }
00374 
00380       inline bool exists(const T& t_element) {
00381          return find_impl(t_element) != NULL;
00382       }
00383 
00388       inline iterator begin() const {
00389          return iterator(m_psFirst);
00390       }
00391 
00396       inline iterator end() const {
00397          return iterator();
00398       }
00399 
00404       inline iterator find(const T& t_element) {
00405          return iterator(find_impl(t_element));
00406       }
00407 
00408    private:
00409 
00410       SSetElement<T>* find_impl(const T& t_element) const {
00411          if(m_psFirst == NULL) {
00412             return NULL;
00413          }
00414          SSetElement<T>* psElem = m_psFirst;
00415          while(psElem != NULL &&
00416                psElem->Data < t_element) {
00417             psElem = psElem->Next;
00418          }
00419          if(psElem == NULL) {
00420             return NULL;
00421          }
00422          else {
00423             return (psElem->Data == t_element) ? psElem : NULL;
00424          }
00425       }
00426 
00427    private:
00428 
00429       SSetElement<T>* m_psFirst;
00430       SSetElement<T>* m_psLast;
00431       size_t m_unSize;
00432 
00433    };
00434 
00435 }
00436 
00437 #endif
```

---

Generated on 10 Jul 2018 for ARGoS by 
 1.6.1 
