# Supplementary material for: Iterative improvement in the automatic modular design of robot swarms
Source: PeerJ Comput Sci. 2020 Dec 7;6:e322. doi: 10.7717/peerj-cs.322 (PMC7924708; doi:10.7717/peerj-cs.322)
Supplement: Supplemental Information 3 [file peerj-cs-06-322-s003.zip › argos3/doc/api/standalone/a00362.html]

ARGoS: core/utility/logging/argos\_colored\_text.h File Reference


- Main Page
- Related Pages
- Namespaces
- Classes
- Files

- File List
- File Members

# core/utility/logging/argos\_colored\_text.h File Reference

`#include <argos3/core/utility/datatypes/datatypes.h>`  

Include dependency graph for argos\_colored\_text.h:

Go to the source code of this file.

|  |  |
| --- | --- |
| Classes | |
| struct | argos::SLogColor |
|  | Stream modifier to set attribute and color of the subsequent text. More... |
| Namespaces | |
| namespace | argos |

|  |  |
| --- | --- |
|  | The namespace containing all the ARGoS related code. |

| Defines | |
| #define | DEFINE\_ARGOS\_STREAM\_COLOR\_HELPER(lc\_color, uc\_color) |
|  | Utility macro to ease the definition of the presets. |
| Enumerations | |
| enum | argos::EARGoSLogAttributes {     argos::ARGOS\_LOG\_ATTRIBUTE\_RESET = 0, argos::ARGOS\_LOG\_ATTRIBUTE\_BRIGHT = 1, argos::ARGOS\_LOG\_ATTRIBUTE\_DIM = 2, argos::ARGOS\_LOG\_ATTRIBUTE\_UNDERSCORE = 3,     argos::ARGOS\_LOG\_ATTRIBUTE\_BLINK = 5, argos::ARGOS\_LOG\_ATTRIBUTE\_REVERSE = 7, argos::ARGOS\_LOG\_ATTRIBUTE\_HIDDEN = 8   } |
|  | The possible attributes of the logged text. More... |
| enum | argos::EARGoSLogColors {     argos::ARGOS\_LOG\_COLOR\_BLACK = 0, argos::ARGOS\_LOG\_COLOR\_RED = 1, argos::ARGOS\_LOG\_COLOR\_GREEN = 2, argos::ARGOS\_LOG\_COLOR\_YELLOW = 3,     argos::ARGOS\_LOG\_COLOR\_BLUE = 4, argos::ARGOS\_LOG\_COLOR\_MAGENTA = 5, argos::ARGOS\_LOG\_COLOR\_CYAN = 6, argos::ARGOS\_LOG\_COLOR\_WHITE = 7   } |
|  | The possible colors of the logged text. More... |
| Functions | |
| std::ostream & | argos::operator<< (std::ostream &c\_os, const SLogColor &s\_log\_color) |
|  | Stream operator that accepts the stream modifier. |
|  | argos::DEFINE\_ARGOS\_STREAM\_COLOR\_HELPER (red, RED) |
|  | Bright red text modifier. |
|  | argos::DEFINE\_ARGOS\_STREAM\_COLOR\_HELPER (green, GREEN) |
|  | Bright green text modifier. |
|  | argos::DEFINE\_ARGOS\_STREAM\_COLOR\_HELPER (yellow, YELLOW) |
|  | Bright yellow text modifier. |
|  | argos::DEFINE\_ARGOS\_STREAM\_COLOR\_HELPER (blue, BLUE) |
|  | Bright blue text modifier. |
|  | argos::DEFINE\_ARGOS\_STREAM\_COLOR\_HELPER (magenta, MAGENTA) |
|  | Bright magenta text modifier. |
|  | argos::DEFINE\_ARGOS\_STREAM\_COLOR\_HELPER (cyan, CYAN) |
|  | Bright cyan text modifier. |
|  | argos::DEFINE\_ARGOS\_STREAM\_COLOR\_HELPER (white, WHITE) |
|  | Bright white text modifier. |
| std::ostream & | argos::reset (std::ostream &c\_os) |
|  | Resets the text to the default settings. |

---

## Define Documentation

|  |  |  |
| --- | --- | --- |
| #define DEFINE\_ARGOS\_STREAM\_COLOR\_HELPER | ( | lc\_color, |
|  |  | uc\_color |  | ) |  |

**Value:**

```
inline std::ostream& lc_color(std::ostream& c_os) {                  \
      c_os << SLogColor(ARGOS_LOG_ATTRIBUTE_BRIGHT, ARGOS_LOG_COLOR_ ## uc_color); \
      return c_os;                                                      \
   }
```

Utility macro to ease the definition of the presets.

Definition at line 89 of file argos\_colored\_text.h.

---

Generated on 10 Jul 2018 for ARGoS by 
 1.6.1 
