# Supplementary material for: Iterative improvement in the automatic modular design of robot swarms
Source: PeerJ Comput Sci. 2020 Dec 7;6:e322. doi: 10.7717/peerj-cs.322 (PMC7924708; doi:10.7717/peerj-cs.322)
Supplement: Supplemental Information 3 [file peerj-cs-06-322-s003.zip › argos3/doc/api/standalone/a00362_source.html]

ARGoS: core/utility/logging/argos\_colored\_text.h Source File


- Main Page
- Related Pages
- Namespaces
- Classes
- Files

- File List
- File Members

# core/utility/logging/argos\_colored\_text.h

Go to the documentation of this file.

```
00001 
00012 #ifndef ARGOS_COLORED_TEXT_H
00013 #define ARGOS_COLORED_TEXT_H
00014 
00015 #include <argos3/core/utility/datatypes/datatypes.h>
00016 
00017 namespace argos {
00018 
00022    enum EARGoSLogAttributes {
00023       ARGOS_LOG_ATTRIBUTE_RESET      = 0,
00024       ARGOS_LOG_ATTRIBUTE_BRIGHT     = 1,
00025       ARGOS_LOG_ATTRIBUTE_DIM        = 2,
00026       ARGOS_LOG_ATTRIBUTE_UNDERSCORE = 3,
00027       ARGOS_LOG_ATTRIBUTE_BLINK      = 5,
00028       ARGOS_LOG_ATTRIBUTE_REVERSE    = 7,
00029       ARGOS_LOG_ATTRIBUTE_HIDDEN     = 8
00030    };
00031 
00035    enum EARGoSLogColors {
00036       ARGOS_LOG_COLOR_BLACK   = 0,
00037       ARGOS_LOG_COLOR_RED     = 1,
00038       ARGOS_LOG_COLOR_GREEN   = 2,
00039       ARGOS_LOG_COLOR_YELLOW  = 3,
00040       ARGOS_LOG_COLOR_BLUE    = 4,
00041       ARGOS_LOG_COLOR_MAGENTA = 5,
00042       ARGOS_LOG_COLOR_CYAN    = 6,
00043       ARGOS_LOG_COLOR_WHITE   = 7
00044    };
00045 
00049    struct SLogColor {
00053       UInt8 Attribute;
00057       UInt8 Foreground;
00058 
00062       SLogColor() :
00063          Attribute(ARGOS_LOG_ATTRIBUTE_RESET),
00064          Foreground(ARGOS_LOG_COLOR_WHITE) {}
00068       SLogColor(EARGoSLogAttributes e_attribute,
00069                 EARGoSLogColors e_foreground_color) :
00070          Attribute(e_attribute),
00071          Foreground(e_foreground_color) {}
00072    };
00073 
00078    inline std::ostream& operator<<(std::ostream& c_os, const SLogColor& s_log_color)
00079    {
00080       c_os << "\033["
00081            << s_log_color.Attribute << ";"
00082            << (s_log_color.Foreground + 30) << "m";
00083       return c_os;
00084    }
00085 
00089 #define DEFINE_ARGOS_STREAM_COLOR_HELPER(lc_color, uc_color)            \
00090    inline std::ostream& lc_color(std::ostream& c_os) {                  \
00091       c_os << SLogColor(ARGOS_LOG_ATTRIBUTE_BRIGHT, ARGOS_LOG_COLOR_ ## uc_color); \
00092       return c_os;                                                      \
00093    }
00094 
00098    DEFINE_ARGOS_STREAM_COLOR_HELPER(red, RED);
00102    DEFINE_ARGOS_STREAM_COLOR_HELPER(green, GREEN);
00106    DEFINE_ARGOS_STREAM_COLOR_HELPER(yellow, YELLOW);
00110    DEFINE_ARGOS_STREAM_COLOR_HELPER(blue, BLUE);
00114    DEFINE_ARGOS_STREAM_COLOR_HELPER(magenta, MAGENTA);
00118    DEFINE_ARGOS_STREAM_COLOR_HELPER(cyan, CYAN);
00122    DEFINE_ARGOS_STREAM_COLOR_HELPER(white, WHITE);
00123 
00128    inline std::ostream& reset(std::ostream& c_os) {
00129       c_os << "\033["
00130            << ARGOS_LOG_ATTRIBUTE_RESET
00131            << "m";
00132       return c_os;
00133    }
00134 
00135 }
00136 
00137 #endif
```

---

Generated on 10 Jul 2018 for ARGoS by 
 1.6.1 
