# Supplementary material for: Iterative improvement in the automatic modular design of robot swarms
Source: PeerJ Comput Sci. 2020 Dec 7;6:e322. doi: 10.7717/peerj-cs.322 (PMC7924708; doi:10.7717/peerj-cs.322)
Supplement: Supplemental Information 3 [file peerj-cs-06-322-s003.zip › argos3/doc/api/standalone/a00363.html]

ARGoS: core/utility/logging/argos\_log.cpp File Reference


- Main Page
- Related Pages
- Namespaces
- Classes
- Files

- File List
- File Members

# core/utility/logging/argos\_log.cpp File Reference

`#include "argos_log.h"`  

Include dependency graph for argos\_log.cpp:

Go to the source code of this file.

|  |  |
| --- | --- |
| Namespaces | |
| namespace | argos |

|  |  |
| --- | --- |
|  | The namespace containing all the ARGoS related code. |

| Variables | |
| size\_t | argos::DEBUG\_INDENTATION = 0 |
| CARGoSLog | argos::LOG (std::cout, SLogColor(ARGOS\_LOG\_ATTRIBUTE\_BRIGHT, ARGOS\_LOG\_COLOR\_GREEN)) |
| CARGoSLog | argos::LOGERR (std::cerr, SLogColor(ARGOS\_LOG\_ATTRIBUTE\_BRIGHT, ARGOS\_LOG\_COLOR\_RED)) |

---

Generated on 10 Jul 2018 for ARGoS by 
 1.6.1 
