# Supplementary material for: Iterative improvement in the automatic modular design of robot swarms
Source: PeerJ Comput Sci. 2020 Dec 7;6:e322. doi: 10.7717/peerj-cs.322 (PMC7924708; doi:10.7717/peerj-cs.322)
Supplement: Supplemental Information 3 [file peerj-cs-06-322-s003.zip › argos3/doc/api/standalone/a00363_source.html]

ARGoS: core/utility/logging/argos\_log.cpp Source File


- Main Page
- Related Pages
- Namespaces
- Classes
- Files

- File List
- File Members

# core/utility/logging/argos\_log.cpp

Go to the documentation of this file.

```
00001 
00007 #include "argos_log.h"
00008 
00009 namespace argos {
00010 
00011    size_t DEBUG_INDENTATION = 0;
00012    CARGoSLog LOG(std::cout, SLogColor(ARGOS_LOG_ATTRIBUTE_BRIGHT, ARGOS_LOG_COLOR_GREEN));
00013    CARGoSLog LOGERR(std::cerr, SLogColor(ARGOS_LOG_ATTRIBUTE_BRIGHT, ARGOS_LOG_COLOR_RED));
00014 
00015 }
```

---

Generated on 10 Jul 2018 for ARGoS by 
 1.6.1 
