# Supplementary material for: Iterative improvement in the automatic modular design of robot swarms
Source: PeerJ Comput Sci. 2020 Dec 7;6:e322. doi: 10.7717/peerj-cs.322 (PMC7924708; doi:10.7717/peerj-cs.322)
Supplement: Supplemental Information 3 [file peerj-cs-06-322-s003.zip › argos3/doc/api/standalone/a00364.html]

ARGoS: core/utility/logging/argos\_log.h File Reference


- Main Page
- Related Pages
- Namespaces
- Classes
- Files

- File List
- File Members

# core/utility/logging/argos\_log.h File Reference

`#include <argos3/core/config.h>`  
`#include <iomanip>`  
`#include <string>`  
`#include <iostream>`  
`#include <fstream>`  
`#include <cstdio>`  
`#include <cstring>`  
`#include <cstdlib>`  
`#include <argos3/core/utility/logging/argos_colored_text.h>`  

Include dependency graph for argos\_log.h:

This graph shows which files directly or indirectly include this file:

Go to the source code of this file.

|  |  |
| --- | --- |
| Classes | |
| class | argos::CARGoSLog |
| Namespaces | |
| namespace | argos |

|  |  |
| --- | --- |
|  | The namespace containing all the ARGoS related code. |

| Defines | |
| #define | DEBUG(MSG,...)   { fprintf(stderr, "[DEBUG] "); for(size\_t ARGOS\_I = 0; ARGOS\_I < DEBUG\_INDENTATION; ++ARGOS\_I) fprintf(stderr, " "); fprintf(stderr, MSG, ##\_\_VA\_ARGS\_\_); } |
| #define | DEBUG\_FUNCTION\_ENTER   { ++DEBUG\_INDENTATION; DEBUG("%s - START\n", \_\_PRETTY\_FUNCTION\_\_ ); } |
| #define | DEBUG\_FUNCTION\_EXIT   { DEBUG("%s - END\n", \_\_PRETTY\_FUNCTION\_\_ ); --DEBUG\_INDENTATION; } |
| #define | TRACE(LINE)   LINE; DEBUG(#LINE "\n"); |
| #define | RLOG   LOG << "[" << GetId() << "] " |
| #define | RLOGERR   LOGERR << "[" << GetId() << "] " |

---

## Define Documentation

|  |  |  |
| --- | --- | --- |
| #define DEBUG | ( | MSG, |
|  |  | *...* |  | ) | { fprintf(stderr, "[DEBUG] "); for(size\_t ARGOS\_I = 0; ARGOS\_I < DEBUG\_INDENTATION; ++ARGOS\_I) fprintf(stderr, " "); fprintf(stderr, MSG, ##\_\_VA\_ARGS\_\_); } |

Definition at line 48 of file argos\_log.h.

|  |
| --- |
| #define DEBUG\_FUNCTION\_ENTER   { ++DEBUG\_INDENTATION; DEBUG("%s - START\n", \_\_PRETTY\_FUNCTION\_\_ ); } |

Definition at line 50 of file argos\_log.h.

|  |
| --- |
| #define DEBUG\_FUNCTION\_EXIT   { DEBUG("%s - END\n", \_\_PRETTY\_FUNCTION\_\_ ); --DEBUG\_INDENTATION; } |

Definition at line 52 of file argos\_log.h.

|  |
| --- |
| #define RLOG   LOG << "[" << GetId() << "] " |

Definition at line 187 of file argos\_log.h.

|  |
| --- |
| #define RLOGERR   LOGERR << "[" << GetId() << "] " |

Definition at line 188 of file argos\_log.h.

|  |  |  |  |  |  |
| --- | --- | --- | --- | --- | --- |
| #define TRACE | ( | LINE |  | ) | LINE; DEBUG(#LINE "\n"); |

Definition at line 54 of file argos\_log.h.

---

Generated on 10 Jul 2018 for ARGoS by 
 1.6.1 
