# Supplementary material for: Iterative improvement in the automatic modular design of robot swarms
Source: PeerJ Comput Sci. 2020 Dec 7;6:e322. doi: 10.7717/peerj-cs.322 (PMC7924708; doi:10.7717/peerj-cs.322)
Supplement: Supplemental Information 3 [file peerj-cs-06-322-s003.zip › argos3/doc/api/standalone/a00364_source.html]

ARGoS: core/utility/logging/argos\_log.h Source File


- Main Page
- Related Pages
- Namespaces
- Classes
- Files

- File List
- File Members

# core/utility/logging/argos\_log.h

Go to the documentation of this file.

```
00001 
00016 #ifndef ARGOSLOG_H
00017 #define ARGOSLOG_H
00018 
00019 #include <argos3/core/config.h>
00020 #include <iomanip>
00021 #include <string>
00022 #include <iostream>
00023 #include <fstream>
00024 #include <cstdio>
00025 #include <cstring>
00026 #include <cstdlib>
00027 
00028 #ifdef ARGOS_THREADSAFE_LOG
00029 #include <pthread.h>
00030 #include <sstream>
00031 #include <map>
00032 #include <vector>
00033 #endif
00034 
00035 namespace argos {
00036    class CARGOSLogger;
00037 }
00038 
00039 #include <argos3/core/utility/logging/argos_colored_text.h>
00040 
00041 namespace argos {
00042 
00043    /****************************************/
00044    /****************************************/
00045 
00046    extern size_t DEBUG_INDENTATION;
00047 
00048 #define DEBUG(MSG, ...) { fprintf(stderr, "[DEBUG] "); for(size_t ARGOS_I = 0; ARGOS_I < DEBUG_INDENTATION; ++ARGOS_I) fprintf(stderr, "  "); fprintf(stderr, MSG, ##__VA_ARGS__); }
00049 
00050 #define DEBUG_FUNCTION_ENTER { ++DEBUG_INDENTATION; DEBUG("%s - START\n", __PRETTY_FUNCTION__ ); }
00051 
00052 #define DEBUG_FUNCTION_EXIT { DEBUG("%s - END\n", __PRETTY_FUNCTION__ ); --DEBUG_INDENTATION; }
00053 
00054 #define TRACE(LINE) LINE; DEBUG(#LINE "\n");
00055 
00056    /****************************************/
00057    /****************************************/
00058 
00059    class CARGoSLog {
00060 
00061    private:
00062 
00064       std::ostream& m_cStream;
00065 
00067       SLogColor m_sLogColor;
00068 
00070       bool m_bColoredOutput;
00071 
00072 #ifdef ARGOS_THREADSAFE_LOG
00073 
00074       std::map<pthread_t, size_t> m_mapStreamOrder;
00075 
00077       std::vector<std::stringstream*> m_vecStreams;
00078 
00080       pthread_mutex_t m_tMutex;
00081 #endif
00082 
00083    public:
00084 
00085       CARGoSLog(std::ostream& c_stream,
00086                 const SLogColor& s_log_color,
00087                 bool b_colored_output_enabled = true) :
00088          m_cStream(c_stream),
00089          m_sLogColor(s_log_color),
00090          m_bColoredOutput(b_colored_output_enabled) {
00091 #ifdef ARGOS_THREADSAFE_LOG
00092          pthread_mutex_init(&m_tMutex, NULL);
00093          AddThreadSafeBuffer();
00094 #endif
00095       }
00096 
00097       ~CARGoSLog() {
00098 #ifdef ARGOS_THREADSAFE_LOG
00099          pthread_mutex_destroy(&m_tMutex);
00100          while(!m_vecStreams.empty()) {
00101             delete m_vecStreams.back();
00102             m_vecStreams.pop_back();
00103          }
00104 #endif
00105          if(m_bColoredOutput) {
00106             reset(m_cStream);
00107          }
00108       }
00109 
00110       inline void EnableColoredOutput() {
00111          m_bColoredOutput = true;
00112       }
00113 
00114       inline void DisableColoredOutput() {
00115          m_bColoredOutput = false;
00116       }
00117 
00118       inline bool IsColoredOutput() const {
00119          return m_bColoredOutput;
00120       }
00121 
00122       inline std::ostream& GetStream() {
00123          return m_cStream;
00124       }
00125 
00126       inline void RedirectToFile(const std::string& str_fname) {
00127          m_cStream.rdbuf(std::ofstream(str_fname.c_str(), std::ios::out | std::ios::trunc).rdbuf());
00128       }
00129 
00130 #ifdef ARGOS_THREADSAFE_LOG
00131       inline void Flush() {
00132          pthread_mutex_lock(&m_tMutex);
00133          for(size_t i = 0; i < m_vecStreams.size(); ++i) {
00134             m_cStream << m_vecStreams[i]->str();
00135             m_vecStreams[i]->str("");
00136          }
00137          pthread_mutex_unlock(&m_tMutex);
00138       }
00139 
00140       inline void AddThreadSafeBuffer() {
00141          pthread_mutex_lock(&m_tMutex);
00142          m_mapStreamOrder.insert(std::make_pair<pthread_t, size_t>(pthread_self(), m_vecStreams.size()));
00143          m_vecStreams.push_back(new std::stringstream);
00144          pthread_mutex_unlock(&m_tMutex);
00145       }
00146 #else
00147       void Flush() {}
00148 #endif
00149       
00150       inline CARGoSLog& operator<<(std::ostream& (*c_stream)(std::ostream&)) {
00151 #ifdef ARGOS_THREADSAFE_LOG
00152          *(m_vecStreams[m_mapStreamOrder.find(pthread_self())->second]) << c_stream;
00153 #else
00154          m_cStream << c_stream;
00155 #endif
00156          return *this;
00157       }
00158 
00159       template <typename T> CARGoSLog& operator<<(const T t_msg) {
00160          if(m_bColoredOutput) {
00161 #ifdef ARGOS_THREADSAFE_LOG
00162             *(m_vecStreams[m_mapStreamOrder.find(pthread_self())->second]) << m_sLogColor << t_msg << reset;
00163 #else
00164             m_cStream << m_sLogColor << t_msg << reset;
00165 #endif
00166          }
00167          else {
00168 #ifdef ARGOS_THREADSAFE_LOG
00169             *(m_vecStreams[m_mapStreamOrder.find(pthread_self())->second]) << t_msg;
00170 #else
00171             m_cStream << m_sLogColor << t_msg << reset;
00172 #endif
00173          }
00174          return *this;
00175       }
00176 
00177    };
00178 
00179    extern CARGoSLog LOG;
00180    extern CARGoSLog LOGERR;
00181 
00182 }
00183 
00184    /****************************************/
00185    /****************************************/
00186 
00187 #define RLOG    LOG    << "[" << GetId() << "] "
00188 #define RLOGERR LOGERR << "[" << GetId() << "] "
00189 
00190    /****************************************/
00191    /****************************************/
00192 
00193 #endif
```

---

Generated on 10 Jul 2018 for ARGoS by 
 1.6.1 
