# Supplementary material for: Iterative improvement in the automatic modular design of robot swarms
Source: PeerJ Comput Sci. 2020 Dec 7;6:e322. doi: 10.7717/peerj-cs.322 (PMC7924708; doi:10.7717/peerj-cs.322)
Supplement: Supplemental Information 3 [file peerj-cs-06-322-s003.zip › argos3/doc/api/standalone/a00365_source.html]

ARGoS: core/utility/math/angles.cpp Source File


- Main Page
- Related Pages
- Namespaces
- Classes
- Files

- File List
- File Members

# core/utility/math/angles.cpp

Go to the documentation of this file.

```
00001 
00007 #include "angles.h"
00008 
00009 namespace argos {
00010 
00011    const CRadians CRadians::PI(ARGOS_PI);
00012    const CRadians CRadians::TWO_PI(2.0f * CRadians::PI);
00013    const CRadians CRadians::PI_OVER_TWO(CRadians::PI / 2.0f);
00014    const CRadians CRadians::PI_OVER_THREE(CRadians::PI / 3.0f);
00015    const CRadians CRadians::PI_OVER_FOUR(CRadians::PI / 4.0f);
00016    const CRadians CRadians::PI_OVER_SIX(CRadians::PI / 6.0f);
00017    const CRadians CRadians::ZERO;
00018 
00019    const CRange<CRadians> CRadians::SIGNED_RANGE(-CRadians::PI, CRadians::PI);
00020    const CRange<CRadians> CRadians::UNSIGNED_RANGE(CRadians(), CRadians::TWO_PI);
00021    const Real CRadians::RADIANS_TO_DEGREES(180.0/CRadians::PI.GetValue());
00022 
00023    const CRange<CDegrees> CDegrees::SIGNED_RANGE(CDegrees(-180.0), CDegrees(180.0));
00024    const CRange<CDegrees> CDegrees::UNSIGNED_RANGE(CDegrees(0.0), CDegrees(360.0));
00025    const Real CDegrees::DEGREES_TO_RADIANS(CRadians::PI.GetValue()/180.0);
00026 
00027 }
```

---

Generated on 10 Jul 2018 for ARGoS by 
 1.6.1 
