# Supplementary material for: Iterative improvement in the automatic modular design of robot swarms
Source: PeerJ Comput Sci. 2020 Dec 7;6:e322. doi: 10.7717/peerj-cs.322 (PMC7924708; doi:10.7717/peerj-cs.322)
Supplement: Supplemental Information 3 [file peerj-cs-06-322-s003.zip › argos3/doc/api/standalone/a00366.html]

ARGoS: core/utility/math/angles.h File Reference


- Main Page
- Related Pages
- Namespaces
- Classes
- Files

- File List
- File Members

# core/utility/math/angles.h File Reference

`#include <argos3/core/utility/datatypes/datatypes.h>`  
`#include <argos3/core/utility/math/general.h>`  
`#include <argos3/core/utility/math/range.h>`  
`#include <cmath>`  

Include dependency graph for angles.h:

This graph shows which files directly or indirectly include this file:

Go to the source code of this file.

|  |  |
| --- | --- |
| Classes | |
| class | argos::CRadians |
|  | It defines the basic type CRadians, used to store an angle value in radians. More... |
| class | argos::CDegrees |
|  | It defines the basic type CDegrees, used to store an angle value in degrees. More... |
| Namespaces | |
| namespace | argos |

|  |  |
| --- | --- |
|  | The namespace containing all the ARGoS related code. |

| Defines | |
| #define | ARGOS\_PI   3.14159265358979323846264338327950288 |
|  | To be used when initializing static variables. |
| #define | ARGOS\_SINCOS   ::sincosf |
| #define | ARGOS\_SIN   ::sinf |
| #define | ARGOS\_ASIN   ::asinf |
| #define | ARGOS\_COS   ::cosf |
| #define | ARGOS\_ACOS   ::acosf |
| #define | ARGOS\_TAN   ::tanf |
| #define | ARGOS\_ATAN2   ::atan2f |
| Functions | |
| CDegrees | argos::ToDegrees (const CRadians &c\_radians) |
|  | Converts CRadians to CDegrees. |
| CRadians | argos::ToRadians (const CDegrees &c\_degrees) |
|  | Converts CDegrees to CRadians. |
| CRadians | argos::NormalizedDifference (const CRadians &c\_angle1, const CRadians &c\_angle2) |
|  | Calculates the normalized difference between the given angles. |
| CDegrees | argos::NormalizedDifference (const CDegrees &c\_angle1, const CDegrees &c\_angle2) |
|  | Calculates the normalized difference between the given angles. |
| void | argos::SinCos (const CRadians &c\_radians, Real &f\_sin, Real &f\_cos) |
|  | Computes the sine and cosine of the passed value in radians. |
| Real | argos::Sin (const CRadians &c\_radians) |
|  | Computes the sine of the passed value in radians. |
| Real | argos::Cos (const CRadians &c\_radians) |
|  | Computes the cosine of the passed value in radians. |
| Real | argos::Tan (const CRadians &c\_radians) |
|  | Computes the tangent of the passed value in radians. |
| CRadians | argos::ASin (Real f\_value) |
|  | Computes the arcsine of the passed value. |
| CRadians | argos::ACos (Real f\_value) |
|  | Computes the arccosine of the passed value. |
| CRadians | argos::ATan2 (const Real f\_y, const Real f\_x) |
|  | Computes the arctangent of the passed values. |

---

## Define Documentation

|  |
| --- |
| #define ARGOS\_ACOS   ::acosf |

Definition at line 562 of file angles.h.

|  |
| --- |
| #define ARGOS\_ASIN   ::asinf |

Definition at line 560 of file angles.h.

|  |
| --- |
| #define ARGOS\_ATAN2   ::atan2f |

Definition at line 564 of file angles.h.

|  |
| --- |
| #define ARGOS\_COS   ::cosf |

Definition at line 561 of file angles.h.

|  |
| --- |
| #define ARGOS\_PI   3.14159265358979323846264338327950288 |

To be used when initializing static variables.

Definition at line 32 of file angles.h.

|  |
| --- |
| #define ARGOS\_SIN   ::sinf |

Definition at line 559 of file angles.h.

|  |
| --- |
| #define ARGOS\_SINCOS   ::sincosf |

Definition at line 555 of file angles.h.

|  |
| --- |
| #define ARGOS\_TAN   ::tanf |

Definition at line 563 of file angles.h.

---

Generated on 10 Jul 2018 for ARGoS by 
 1.6.1 
