# Supplementary material for: Iterative improvement in the automatic modular design of robot swarms
Source: PeerJ Comput Sci. 2020 Dec 7;6:e322. doi: 10.7717/peerj-cs.322 (PMC7924708; doi:10.7717/peerj-cs.322)
Supplement: Supplemental Information 3 [file peerj-cs-06-322-s003.zip › argos3/doc/api/standalone/a00366_source.html]

ARGoS: core/utility/math/angles.h Source File


- Main Page
- Related Pages
- Namespaces
- Classes
- Files

- File List
- File Members

# core/utility/math/angles.h

Go to the documentation of this file.

```
00001 
00016 #ifndef ANGLES_H
00017 #define ANGLES_H
00018 
00019 namespace argos {
00020    class CRadians;
00021    class CDegrees;
00022 }
00023 
00024 #include <argos3/core/utility/datatypes/datatypes.h>
00025 #include <argos3/core/utility/math/general.h>
00026 #include <argos3/core/utility/math/range.h>
00027 #include <cmath>
00028 
00032 #define ARGOS_PI 3.14159265358979323846264338327950288
00033 
00034 namespace argos {
00035 
00036    /****************************************/
00037    /****************************************/
00038 
00042    class CRadians {
00043 
00044    public:
00045 
00049       static const CRadians PI;
00050 
00054       static const CRadians TWO_PI;
00055 
00059       static const CRadians PI_OVER_TWO;
00060 
00064       static const CRadians PI_OVER_THREE;
00065 
00069       static const CRadians PI_OVER_FOUR;
00070 
00074       static const CRadians PI_OVER_SIX;
00075 
00079       static const CRadians ZERO;
00080 
00085       CRadians() :
00086          m_fValue(0.0) {
00087       }
00088 
00094       explicit CRadians(Real f_value) :
00095          m_fValue(f_value) {
00096       }
00097 
00103       inline void FromValueInDegrees(Real f_value) {
00104          m_fValue = f_value / RADIANS_TO_DEGREES;
00105       }
00106 
00111       inline Real GetValue() const {
00112          return m_fValue;
00113       }
00114 
00119       inline Real GetAbsoluteValue() const {
00120          return Abs(m_fValue);
00121       }
00122 
00127       inline void SetValue(Real f_value) {
00128          m_fValue = f_value;
00129       }
00130 
00137       inline CRadians& SignedNormalize() {
00138          SIGNED_RANGE.WrapValue(*this);
00139          return *this;
00140       }
00141 
00148       inline CRadians& UnsignedNormalize() {
00149          UNSIGNED_RANGE.WrapValue(*this);
00150          return *this;
00151       }
00152 
00153       inline CRadians& Negate() {
00154          m_fValue = -m_fValue;
00155          return *this;
00156       }
00157 
00158       inline CRadians& operator+() {
00159          return *this;
00160       }
00161 
00162       inline CRadians operator-() const {
00163          return CRadians(-m_fValue);
00164       }
00165 
00166       inline CRadians& operator+=(const CRadians& c_radians) {
00167          m_fValue += c_radians.m_fValue;
00168          return *this;
00169       }
00170 
00171       inline CRadians& operator-=(const CRadians& c_radians) {
00172          m_fValue -= c_radians.m_fValue;
00173          return *this;
00174       }
00175 
00176       inline CRadians& operator*=(Real f_value) {
00177          m_fValue *= f_value;
00178          return *this;
00179       }
00180 
00181       inline CRadians& operator/=(Real f_value) {
00182          m_fValue /= f_value;
00183          return *this;
00184       }
00185 
00186       inline CRadians operator+(const CRadians& c_radians) const {
00187          CRadians cResult(*this);
00188          cResult += c_radians;
00189          return cResult;
00190       }
00191 
00192       inline CRadians operator-(const CRadians& c_radians) const {
00193          CRadians cResult(*this);
00194          cResult -= c_radians;
00195          return cResult;
00196       }
00197 
00198       inline CRadians operator*(Real f_value) const {
00199          CRadians cResult(*this);
00200          cResult *= f_value;
00201          return cResult;
00202       }
00203 
00204       inline friend CRadians operator*(Real f_value,
00205                                        const CRadians& c_radians) {
00206          CRadians cResult(c_radians);
00207          cResult *= f_value;
00208          return cResult;
00209       }
00210 
00211       inline Real operator/(const CRadians& c_radians) const {
00212          return m_fValue / c_radians.m_fValue;
00213       }
00214 
00215       inline CRadians operator/(Real f_value) const {
00216          CRadians cResult(*this);
00217          cResult /= f_value;
00218          return cResult;
00219       }
00220 
00221       inline bool operator<(const CRadians& c_radians) const {
00222          return m_fValue < c_radians.m_fValue;
00223       }
00224 
00225       inline bool operator<=(const CRadians& c_radians) const {
00226          return m_fValue <= c_radians.m_fValue;
00227       }
00228 
00229       inline bool operator>(const CRadians& c_radians) const {
00230          return m_fValue > c_radians.m_fValue;
00231       }
00232 
00233       inline bool operator>=(const CRadians& c_radians) const {
00234          return m_fValue >= c_radians.m_fValue;
00235       }
00236 
00237       inline bool operator==(const CRadians& c_radians) const {
00238          return m_fValue == c_radians.m_fValue;
00239       }
00240 
00241       inline bool operator!=(const CRadians& c_radians) const {
00242          return m_fValue != c_radians.m_fValue;
00243       }
00244 
00249       friend CDegrees ToDegrees(const CRadians& c_radians);
00250 
00251       friend CRadians NormalizedDifference(const CRadians& c_rad1,
00252                                            const CRadians& c_rad2);
00253 
00254       inline friend std::ostream& operator<<(std::ostream& c_os,
00255                                              const CRadians& c_radians) {
00256          c_os << "CRadians("
00257               << c_radians.m_fValue
00258               << " -> "
00259               << c_radians.m_fValue * RADIANS_TO_DEGREES
00260               << " degrees"
00261               << ")";
00262          return c_os;
00263       }
00264 
00265       inline friend std::istream& operator>>(std::istream& is,
00266                                              CRadians& c_radians) {
00267          is >> c_radians.m_fValue;
00268          return is;
00269       }
00270 
00271    public:
00272 
00273       static const CRange<CRadians> SIGNED_RANGE; 
00274       static const CRange<CRadians> UNSIGNED_RANGE; 
00275       static const Real RADIANS_TO_DEGREES; 
00277    private:
00278 
00279       Real m_fValue;            
00280    };
00281 
00282    /****************************************/
00283    /****************************************/
00284 
00288    class CDegrees {
00289 
00290    public:
00291 
00296       CDegrees() :
00297          m_fValue(0.0) {
00298       }
00299 
00305       explicit CDegrees(Real f_value) :
00306          m_fValue(f_value) {
00307       }
00308 
00314       inline void FromValueInRadians(Real f_value) {
00315          m_fValue = f_value / DEGREES_TO_RADIANS;
00316       }
00317 
00322       inline Real GetValue() const {
00323          return m_fValue;
00324       }
00325 
00330       inline Real GetAbsoluteValue() const {
00331        return Abs(m_fValue);
00332       }
00333 
00338       inline void SetValue(Real f_value) {
00339          m_fValue = f_value;
00340       }
00341 
00347       CDegrees& SignedNormalize() {
00348          SIGNED_RANGE.WrapValue(*this);
00349          return (*this);
00350       }
00351 
00357       CDegrees& UnsignedNormalize() {
00358          UNSIGNED_RANGE.WrapValue(*this);
00359          return (*this);
00360       }
00361 
00362       inline CDegrees& operator+() {
00363          return *this;
00364       }
00365 
00366       inline CDegrees operator-() const {
00367          return CDegrees(-m_fValue);
00368       }
00369 
00370       inline CDegrees& operator+=(const CDegrees& c_degrees) {
00371          m_fValue += c_degrees.m_fValue;
00372          return *this;
00373       }
00374 
00375       inline CDegrees& operator-=(const CDegrees& c_degrees) {
00376          m_fValue -= c_degrees.m_fValue;
00377          return *this;
00378       }
00379 
00380       inline CDegrees& operator*=(Real f_value) {
00381          m_fValue *= f_value;
00382          return *this;
00383       }
00384 
00385       inline CDegrees& operator/=(Real f_value) {
00386          m_fValue /= f_value;
00387          return *this;
00388       }
00389 
00390       inline CDegrees operator+(const CDegrees& c_degrees) const {
00391          CDegrees cResult(*this);
00392          cResult += c_degrees;
00393          return cResult;
00394       }
00395 
00396       inline CDegrees operator-(const CDegrees& c_degrees) const {
00397          CDegrees cResult(*this);
00398          cResult -= c_degrees;
00399          return cResult;
00400       }
00401 
00402       inline CDegrees operator*(Real f_value) const {
00403          CDegrees cResult(*this);
00404          cResult *= f_value;
00405          return cResult;
00406       }
00407 
00408       inline friend CDegrees operator*(Real f_value,
00409                                        const CDegrees& c_degrees) {
00410          CDegrees cResult(c_degrees);
00411          cResult *= f_value;
00412          return cResult;
00413       }
00414 
00415       inline Real operator/(const CDegrees& c_degrees) const {
00416          return m_fValue / c_degrees.m_fValue;
00417       }
00418 
00419       inline CDegrees operator/(Real f_value) const {
00420          CDegrees cResult(*this);
00421          cResult /= f_value;
00422          return cResult;
00423       }
00424 
00425       inline bool operator<(const CDegrees& c_degrees) const {
00426          return m_fValue < c_degrees.m_fValue;
00427       }
00428 
00429       inline bool operator<=(const CDegrees& c_degrees) const {
00430          return m_fValue <= c_degrees.m_fValue;
00431       }
00432 
00433       inline bool operator>(const CDegrees& c_degrees) const {
00434          return m_fValue > c_degrees.m_fValue;
00435       }
00436 
00437       inline bool operator>=(const CDegrees& c_degrees) const {
00438          return m_fValue >= c_degrees.m_fValue;
00439       }
00440 
00441       inline bool operator==(const CDegrees& c_degrees) const {
00442          return m_fValue == c_degrees.m_fValue;
00443       }
00444 
00445       inline bool operator!=(const CDegrees& c_degrees) const {
00446          return m_fValue != c_degrees.m_fValue;
00447       }
00448 
00453       friend CRadians ToRadians(const CDegrees& c_degrees);
00454 
00455       friend CDegrees NormalizedDifference(const CDegrees& c_angle1,
00456                                            const CDegrees& c_angle2);
00457 
00458       inline friend std::ostream& operator<<(std::ostream& c_os,
00459                                              const CDegrees& c_degrees) {
00460          c_os << "CDegrees("
00461               << c_degrees.m_fValue
00462               << ")";
00463          return c_os;
00464       }
00465 
00466       inline friend std::istream& operator>>(std::istream& is,
00467                                              CDegrees& c_degrees) {
00468          is >> c_degrees.m_fValue;
00469          return is;
00470       }
00471 
00472    private:
00473 
00474       Real m_fValue; 
00475       static const CRange<CDegrees> SIGNED_RANGE; 
00476       static const CRange<CDegrees> UNSIGNED_RANGE; 
00477       static const Real DEGREES_TO_RADIANS; 
00479    };
00480 
00481    /****************************************/
00482    /****************************************/
00483 
00489    inline CDegrees ToDegrees(const CRadians& c_radians) {
00490       return CDegrees(c_radians.m_fValue * CRadians::RADIANS_TO_DEGREES);
00491    }
00492 
00498    inline CRadians ToRadians(const CDegrees& c_degrees) {
00499       return CRadians(c_degrees.m_fValue * CDegrees::DEGREES_TO_RADIANS);
00500    }
00501 
00510    inline CRadians NormalizedDifference(const CRadians& c_angle1,
00511                                         const CRadians& c_angle2) {
00512       CRadians cResult;
00513       cResult.m_fValue = Mod(c_angle1.m_fValue - c_angle2.m_fValue + CRadians::PI.m_fValue,
00514                              CRadians::TWO_PI.m_fValue);
00515       if(cResult.m_fValue < 0.0f) cResult.m_fValue += CRadians::TWO_PI.m_fValue;
00516       cResult.m_fValue -= CRadians::PI.m_fValue;
00517       return cResult;
00518    }
00519    
00528    inline CDegrees NormalizedDifference(const CDegrees& c_angle1,
00529                                         const CDegrees& c_angle2) {
00530       CDegrees cResult;
00531       cResult.m_fValue = Mod(c_angle1.m_fValue - c_angle2.m_fValue + 180.0f, 360.0f);
00532       if(cResult.m_fValue < 0.0f) cResult.m_fValue += 360.0f;
00533       cResult.m_fValue -= 180.0f;
00534       return cResult;
00535    }
00536    
00537    /****************************************/
00538    /****************************************/
00539 
00540 #undef ARGOS_SINCOS
00541 #ifdef ARGOS_USE_DOUBLE
00542 #  ifndef __APPLE__
00543 #    define ARGOS_SINCOS ::sincos
00544 #  else
00545 #    define ARGOS_SINCOS ::__sincos
00546 #  endif
00547 #  define ARGOS_SIN    ::sin
00548 #  define ARGOS_ASIN   ::asin
00549 #  define ARGOS_COS    ::cos
00550 #  define ARGOS_ACOS   ::acos
00551 #  define ARGOS_TAN    ::tan
00552 #  define ARGOS_ATAN2  ::atan2
00553 #else
00554 #  ifndef __APPLE__
00555 #    define ARGOS_SINCOS ::sincosf
00556 #  else
00557 #    define ARGOS_SINCOS ::__sincosf
00558 #  endif
00559 #  define ARGOS_SIN    ::sinf
00560 #  define ARGOS_ASIN   ::asinf
00561 #  define ARGOS_COS    ::cosf
00562 #  define ARGOS_ACOS   ::acosf
00563 #  define ARGOS_TAN    ::tanf
00564 #  define ARGOS_ATAN2  ::atan2f
00565 #endif
00566 
00567 #ifdef ARGOS_SINCOS
00568 
00574    inline void SinCos(const CRadians& c_radians,
00575                       Real& f_sin,
00576                       Real& f_cos) {
00577       ARGOS_SINCOS(c_radians.GetValue(), &f_sin, &f_cos);
00578    }
00579 #endif
00580 
00586    inline Real Sin(const CRadians& c_radians) {
00587       return ARGOS_SIN(c_radians.GetValue());
00588    }
00589 
00595    inline Real Cos(const CRadians& c_radians) {
00596       return ARGOS_COS(c_radians.GetValue());
00597    }
00598 
00604    inline Real Tan(const CRadians& c_radians) {
00605       return ARGOS_TAN(c_radians.GetValue());
00606    }
00607 
00613    inline CRadians ASin(Real f_value) {
00614       return CRadians(ARGOS_ASIN(f_value));
00615    }
00616 
00622    inline CRadians ACos(Real f_value) {
00623       return CRadians(ARGOS_ACOS(f_value));
00624    }
00625 
00633    inline CRadians ATan2(const Real f_y, const Real f_x) {
00634       return CRadians(ARGOS_ATAN2(f_y, f_x));
00635    }
00636 
00637    /****************************************/
00638    /****************************************/
00639 
00640 }
00641 
00642 #endif
```

---

Generated on 10 Jul 2018 for ARGoS by 
 1.6.1 
