# Supplementary material for: Iterative improvement in the automatic modular design of robot swarms
Source: PeerJ Comput Sci. 2020 Dec 7;6:e322. doi: 10.7717/peerj-cs.322 (PMC7924708; doi:10.7717/peerj-cs.322)
Supplement: Supplemental Information 3 [file peerj-cs-06-322-s003.zip › argos3/doc/api/standalone/a00367_source.html]

ARGoS: core/utility/math/box.cpp Source File


- Main Page
- Related Pages
- Namespaces
- Classes
- Files

- File List
- File Members

# core/utility/math/box.cpp

Go to the documentation of this file.

```
00001 #include "box.h"
00002 #include "ray3.h"
00003 
00004 namespace argos {
00005 
00006    /****************************************/
00007    /****************************************/
00008 
00009    bool CBox::Intersects(Real& f_t_on_ray,
00010                          const CRay3& c_ray) {
00011       /* Transform the ray so the origin is the axis-aligned box base */
00012       CVector3 cRayStart = c_ray.GetStart();
00013       CVector3 cInvRayDir;
00014       c_ray.GetDirection(cInvRayDir);
00015       cRayStart -= m_cBasePos;
00016       cRayStart.Rotate(m_cOrientation.Inverse());
00017       cInvRayDir.Rotate(m_cOrientation.Inverse());
00018       /* Calculate the inverse direction */
00019       cInvRayDir.Set(1.0 / cInvRayDir.GetX(),
00020                      1.0 / cInvRayDir.GetY(),
00021                      1.0 / cInvRayDir.GetZ());
00022       /* X plane */
00023       Real fT1 = (m_cXBounds.GetMin() - cRayStart.GetX()) * cInvRayDir.GetX();
00024       Real fT2 = (m_cXBounds.GetMax() - cRayStart.GetX()) * cInvRayDir.GetX();
00025       Real fTmin = Min(fT1, fT2);
00026       Real fTmax = Max(fT1, fT2);
00027       /* Y plane */
00028       fT1 = (m_cYBounds.GetMin() - cRayStart.GetY()) * cInvRayDir.GetY();
00029       fT2 = (m_cYBounds.GetMax() - cRayStart.GetY()) * cInvRayDir.GetY();
00030       fTmin = Max(fTmin, Min(fT1, fT2));
00031       fTmax = Min(fTmax, Max(fT1, fT2));
00032       if(fTmin > fTmax) return false;
00033       /* Z plane */
00034       fT1 = (m_cZBounds.GetMin() - cRayStart.GetZ()) * cInvRayDir.GetZ();
00035       fT2 = (m_cZBounds.GetMax() - cRayStart.GetZ()) * cInvRayDir.GetZ();
00036       fTmin = Max(fTmin, Min(fT1, fT2));
00037       fTmax = Min(fTmax, Max(fT1, fT2));
00038       if(fTmin > fTmax) return false;
00039       /* The t we search for is the smallest non-negative */
00040       if(fTmin >= 0) f_t_on_ray = fTmin / c_ray.GetLength();
00041       else if(fTmax >= 0) f_t_on_ray = fTmax / c_ray.GetLength();
00042       else return false;
00043       return true;
00044    }
00045 
00046    /****************************************/
00047    /****************************************/
00048 
00049 }
```

---

Generated on 10 Jul 2018 for ARGoS by 
 1.6.1 
