# Supplementary material for: Iterative improvement in the automatic modular design of robot swarms
Source: PeerJ Comput Sci. 2020 Dec 7;6:e322. doi: 10.7717/peerj-cs.322 (PMC7924708; doi:10.7717/peerj-cs.322)
Supplement: Supplemental Information 3 [file peerj-cs-06-322-s003.zip › argos3/doc/api/standalone/a00368_source.html]

ARGoS: core/utility/math/box.h Source File


- Main Page
- Related Pages
- Namespaces
- Classes
- Files

- File List
- File Members

# core/utility/math/box.h

Go to the documentation of this file.

```
00001 
00007 #ifndef BOX_H
00008 #define BOX_H
00009 
00010 namespace argos {
00011    class CBox;
00012    class CRay3;
00013 }
00014 
00015 #include <argos3/core/utility/math/vector3.h>
00016 #include <argos3/core/utility/math/quaternion.h>
00017 
00018 namespace argos {
00019 
00020    class CBox {
00021 
00022    public:
00023 
00024       CBox(const CVector3& c_size,
00025            const CVector3& c_base_pos = CVector3(),
00026            const CQuaternion& c_orient = CQuaternion()) :
00027          m_cBasePos(c_base_pos),
00028          m_cOrientation(c_orient) {
00029          SetSize(c_size);
00030       }
00031 
00032       inline const CVector3& GetSize() const {
00033          return m_cSize;
00034       }
00035 
00036       inline void SetSize(const CVector3& c_size) {
00037          m_cSize = c_size;
00038          m_cXBounds.Set(-m_cSize.GetX() * 0.5, m_cSize.GetX() * 0.5);
00039          m_cYBounds.Set(-m_cSize.GetY() * 0.5, m_cSize.GetY() * 0.5);
00040          m_cZBounds.Set(0.0, m_cSize.GetZ());
00041       }
00042 
00043       inline const CVector3& GetBasePosition() const {
00044          return m_cBasePos;
00045       }
00046 
00047       inline void SetBasePosition(const CVector3& c_base_pos) {
00048          m_cBasePos = c_base_pos;
00049       }
00050 
00051       inline const CQuaternion& GetOrientation() const {
00052          return m_cOrientation;
00053       }
00054 
00055       inline void SetOrientation(const CQuaternion& c_orient) {
00056          m_cOrientation = c_orient;
00057       }
00058 
00059       bool Intersects(Real& f_t_on_ray,
00060                       const CRay3& c_ray);
00061 
00062    private:
00063 
00064       CVector3 m_cSize;
00065       CVector3 m_cBasePos;
00066       CQuaternion m_cOrientation;
00067       CRange<Real> m_cXBounds;
00068       CRange<Real> m_cYBounds;
00069       CRange<Real> m_cZBounds;
00070 
00071    };
00072 }
00073 
00074 #endif
```

---

Generated on 10 Jul 2018 for ARGoS by 
 1.6.1 
