# Supplementary material for: Iterative improvement in the automatic modular design of robot swarms
Source: PeerJ Comput Sci. 2020 Dec 7;6:e322. doi: 10.7717/peerj-cs.322 (PMC7924708; doi:10.7717/peerj-cs.322)
Supplement: Supplemental Information 3 [file peerj-cs-06-322-s003.zip › argos3/doc/api/standalone/a00369_source.html]

ARGoS: core/utility/math/cylinder.cpp Source File


- Main Page
- Related Pages
- Namespaces
- Classes
- Files

- File List
- File Members

# core/utility/math/cylinder.cpp

Go to the documentation of this file.

```
00001 #include "cylinder.h"
00002 #include "ray3.h"
00003 
00004 namespace argos {
00005 
00006    /****************************************/
00007    /****************************************/
00008 
00009    bool CCylinder::Intersects(Real& f_t_on_ray,
00010                               const CRay3& c_ray) {
00011       /* Solution candidates */
00012       UInt32 unSolutionCount = 0;
00013       Real pfSolutions[4];
00014       /* Position of top cap relative to bottom one */
00015       CVector3 cRelTopCap = m_fHeight * m_cAxis;
00016       /*
00017        * Check intersection with cylinder
00018        */
00019       CVector3 cPRA = c_ray.GetStart() - m_cBasePos;
00020       CVector3 cBeta = cPRA - m_cAxis.DotProduct(cPRA) * m_cAxis;
00021       CVector3 cRayDir;
00022       c_ray.GetDirection(cRayDir);
00023       Real fDRA = m_cAxis.DotProduct(cRayDir);
00024       CVector3 cAlpha = cRayDir - fDRA * m_cAxis;
00025       Real fA = cAlpha.SquareLength();
00026       Real fB = 2.0 * cAlpha.DotProduct(cBeta);
00027       Real fC = cBeta.SquareLength() - Square(m_fRadius);
00028       Real fDelta = Square(fB) - 4.0 * fA * fC;
00029       if(fDelta == 0) {
00030          /* One candidate solution, is it within the cylinder caps? */
00031          pfSolutions[0] = -fB / (2.0 * fA);
00032          if(pfSolutions[0] > 0.0) {
00033             CVector3 cTest = cPRA + pfSolutions[0] * cRayDir;
00034             if(m_cAxis.DotProduct(cTest) > 0) {
00035                cTest -= cRelTopCap;
00036                if(m_cAxis.DotProduct(cTest) < 0) {
00037                   ++unSolutionCount;
00038                }
00039             }
00040          }
00041       }
00042       else if(fDelta > 0) {
00043          /* Two candidate solutions, are they within the cylinder caps? */
00044          CVector3 cTest;
00045          /* Test first solution */
00046          pfSolutions[0] = (-fB + Sqrt(fDelta)) / (2.0 * fA);
00047          if(pfSolutions[0] > 0.0) {
00048             cTest = cPRA + pfSolutions[0] * cRayDir;
00049             if(m_cAxis.DotProduct(cTest) > 0) {
00050                cTest -= cRelTopCap;
00051                if(m_cAxis.DotProduct(cTest) < 0) {
00052                   ++unSolutionCount;
00053                }
00054             }
00055          }
00056          /* Test second solution */
00057          pfSolutions[unSolutionCount] = (-fB - Sqrt(fDelta)) / (2.0 * fA);
00058          if(pfSolutions[unSolutionCount] > 0.0) {
00059             cTest = cPRA + pfSolutions[unSolutionCount] * cRayDir;
00060             if(m_cAxis.DotProduct(cTest) > 0) {
00061                cTest -= cRelTopCap;
00062                if(m_cAxis.DotProduct(cTest) < 0) {
00063                   ++unSolutionCount;
00064                }
00065             }
00066          }
00067       }
00068       /*
00069        * Check intersection with bottom and top caps
00070        */
00071       /* If the directions of the cylinder axis and the ray are parallel,
00072        * nothing to do */
00073       if(fDRA > 10e-6) {
00074          Real fPPRA = m_cAxis.DotProduct(cPRA);
00075          /* Bottom cap */
00076          pfSolutions[unSolutionCount] = -fPPRA / fDRA;
00077          if(pfSolutions[unSolutionCount] > 0.0 &&
00078             (cPRA + pfSolutions[unSolutionCount] * cRayDir).SquareLength() < Square(m_fRadius)) {
00079             ++unSolutionCount;
00080          }
00081          /* Top cap */
00082          pfSolutions[unSolutionCount] = -(fPPRA - m_fHeight) / fDRA;
00083          if(pfSolutions[unSolutionCount] > 0.0 &&
00084             (cPRA - cRelTopCap + pfSolutions[unSolutionCount] * cRayDir).SquareLength() < Square(m_fRadius)) {
00085             ++unSolutionCount;
00086          }
00087       }
00088       /*
00089        * All possible solutions have been found, take the closest
00090        */
00091       if(unSolutionCount == 0) {
00092          return false;
00093       }
00094       f_t_on_ray = pfSolutions[0];
00095       for(UInt32 i = 1; i < unSolutionCount; ++i) {
00096          if(pfSolutions[i] < f_t_on_ray)
00097             f_t_on_ray = pfSolutions[i];
00098       }
00099       f_t_on_ray /= c_ray.GetLength();
00100       return true;
00101    }
00102 
00103    /****************************************/
00104    /****************************************/
00105 
00106 }
```

---

Generated on 10 Jul 2018 for ARGoS by 
 1.6.1 
