# Supplementary material for: Iterative improvement in the automatic modular design of robot swarms
Source: PeerJ Comput Sci. 2020 Dec 7;6:e322. doi: 10.7717/peerj-cs.322 (PMC7924708; doi:10.7717/peerj-cs.322)
Supplement: Supplemental Information 3 [file peerj-cs-06-322-s003.zip › argos3/doc/api/standalone/a00370_source.html]

ARGoS: core/utility/math/cylinder.h Source File


- Main Page
- Related Pages
- Namespaces
- Classes
- Files

- File List
- File Members

# core/utility/math/cylinder.h

Go to the documentation of this file.

```
00001 
00007 #ifndef CYLINDER_H
00008 #define CYLINDER_H
00009 
00010 namespace argos {
00011    class CCylinder;
00012    class CRay3;
00013 }
00014 
00015 #include <argos3/core/utility/math/vector3.h>
00016 
00017 namespace argos {
00018 
00019    class CCylinder {
00020 
00021    public:
00022 
00023       CCylinder(Real f_radius,
00024                 Real f_height,
00025                 const CVector3& c_base_pos = CVector3(),
00026                 const CVector3& c_axis = CVector3::Z) :
00027          m_fRadius(f_radius),
00028          m_fHeight(f_height),
00029          m_cBasePos(c_base_pos),
00030          m_cAxis(c_axis) {
00031       }
00032 
00033       inline Real GetRadius() const {
00034          return m_fRadius;
00035       }
00036 
00037       inline void SetRadius(Real f_radius) {
00038          m_fRadius = f_radius;
00039       }
00040 
00041       inline Real GetHeight() const {
00042          return m_fHeight;
00043       }
00044 
00045       inline void SetHeight(Real f_height) {
00046          m_fHeight = f_height;
00047       }
00048 
00049       inline const CVector3& GetBasePosition() const {
00050          return m_cBasePos;
00051       }
00052 
00053       inline void SetBasePosition(const CVector3& c_base_pos) {
00054          m_cBasePos = c_base_pos;
00055       }
00056 
00057       inline const CVector3& GetAxis() const {
00058          return m_cAxis;
00059       }
00060 
00061       inline void SetAxis(const CVector3& c_axis) {
00062          m_cAxis = c_axis;
00063       }
00064 
00065       bool Intersects(Real& f_t_on_ray,
00066                       const CRay3& c_ray);
00067 
00068    private:
00069 
00070       Real m_fRadius;
00071       Real m_fHeight;
00072       CVector3 m_cBasePos;
00073       CVector3 m_cAxis;
00074 
00075    };
00076 }
00077 
00078 #endif
```

---

Generated on 10 Jul 2018 for ARGoS by 
 1.6.1 
