# Supplementary material for: Iterative improvement in the automatic modular design of robot swarms
Source: PeerJ Comput Sci. 2020 Dec 7;6:e322. doi: 10.7717/peerj-cs.322 (PMC7924708; doi:10.7717/peerj-cs.322)
Supplement: Supplemental Information 3 [file peerj-cs-06-322-s003.zip › argos3/doc/api/standalone/a00371.html]

ARGoS: core/utility/math/general.h File Reference


- Main Page
- Related Pages
- Namespaces
- Classes
- Files

- File List
- File Members

# core/utility/math/general.h File Reference

`#include <argos3/core/utility/datatypes/datatypes.h>`  
`#include <vector>`  
`#include <utility>`  
`#include <cmath>`  

Include dependency graph for general.h:

Go to the source code of this file.

|  |  |
| --- | --- |
| Namespaces | |
| namespace | argos |

|  |  |
| --- | --- |
|  | The namespace containing all the ARGoS related code. |

| Defines | |
| #define | Log   ::logf |
| #define | Sqrt   ::sqrtf |
| #define | Exp   ::expf |
| #define | Mod   ::fmodf |
| Functions | |
| template<typename T > | |
| T | argos::Abs (const T &t\_v) |
|  | Returns the absolute value of the passed argument. |
| SInt32 | argos::Abs (SInt32 t\_v) |
|  | Returns the absolute value of the passed argument. |
| Real | argos::Abs (Real t\_v) |
|  | Returns the absolute value of the passed argument. |
| template<typename T > | |
| T | argos::Min (const T &t\_v1, const T &t\_v2) |
|  | Returns the smaller of the two passed arguments. |
| template<typename T > | |
| T & | argos::Min (T &t\_v1, T &t\_v2) |
|  | Returns the smaller of the two passed arguments. |
| template<typename T > | |
| T | argos::Max (const T &t\_v1, const T &t\_v2) |
|  | Returns the bigger of the two passed arguments. |
| template<typename T > | |
| T & | argos::Max (T &t\_v1, T &t\_v2) |
|  | Returns the bigger of the two passed arguments. |
| template<typename T > | |
| SInt32 | argos::Sign (const T &t\_v) |
|  | Returns the sign of the value of the passed argument. |
| template<typename T > | |
| T | argos::Square (const T &t\_v) |
|  | Returns the square of the value of the passed argument. |
| SInt32 | argos::Floor (Real f\_value) |
|  | Rounds the passed floating-point value to the closest lower integer. |
| SInt32 | argos::Ceil (Real f\_value) |
|  | Rounds the passed floating-point value to the closest higher integer. |
| SInt32 | argos::Round (Real f\_value) |
|  | Rounds the passed floating-point value to the closest integer. |
| SInt32 | argos::RoundClosestToZero (Real f\_value) |
|  | Rounds the passed floating-point value to the integer closest to zero. |
| bool | argos::DoubleEqAbsolute (Real f\_value1, Real f\_value2, Real f\_epsilon) |
|  | Tests whether a floating-point value is lower than another. |
| bool | argos::DoubleEq (Real f\_value1, Real f\_value2) |
|  | Tests whether a floating-point value is lower than another. |
| Real | argos::Interpolate (Real f\_x, const std::vector< std::pair< Real, Real > > &c\_points) |
|  | Return the value of the linear interpolation. |

---

## Define Documentation

|  |
| --- |
| #define Exp   ::expf |

Definition at line 65 of file general.h.

|  |
| --- |
| #define Log   ::logf |

Definition at line 63 of file general.h.

|  |
| --- |
| #define Mod   ::fmodf |

Definition at line 66 of file general.h.

|  |
| --- |
| #define Sqrt   ::sqrtf |

Definition at line 64 of file general.h.

---

Generated on 10 Jul 2018 for ARGoS by 
 1.6.1 
