# Supplementary material for: Iterative improvement in the automatic modular design of robot swarms
Source: PeerJ Comput Sci. 2020 Dec 7;6:e322. doi: 10.7717/peerj-cs.322 (PMC7924708; doi:10.7717/peerj-cs.322)
Supplement: Supplemental Information 3 [file peerj-cs-06-322-s003.zip › argos3/doc/api/standalone/a00371_source.html]

ARGoS: core/utility/math/general.h Source File


- Main Page
- Related Pages
- Namespaces
- Classes
- Files

- File List
- File Members

# core/utility/math/general.h

Go to the documentation of this file.

```
00001 
00009 #ifndef GENERAL_H
00010 #define GENERAL_H
00011 
00012 #include <argos3/core/utility/datatypes/datatypes.h>
00013 #include <vector>
00014 #include <utility>
00015 #include <cmath>
00016 
00017 namespace argos {
00018     /****************************************/
00019     /****************************************/
00020 
00025     template<typename T> T Abs(const T& t_v) {
00026         if (t_v > T(0)) return t_v;
00027         if (t_v < T(0)) return -t_v;
00028         return T(0);
00029     }
00030 
00037     inline SInt32 Abs(SInt32 t_v) {
00038         if (t_v > 0) return t_v;
00039         if (t_v < 0) return -t_v;
00040         return 0;
00041     }
00042 
00048     inline Real Abs(Real t_v) {
00049         if (t_v > 0.0f) return t_v;
00050         if (t_v < 0.0f) return -t_v;
00051         return 0.0f;
00052     }
00053 
00054     /****************************************/
00055     /****************************************/
00056 
00057 #ifdef ARGOS_USE_DOUBLE
00058 #define Log  ::log
00059 #define Sqrt ::sqrt
00060 #define Exp ::exp
00061 #define Mod ::fmod
00062 #else
00063 #define Log ::logf
00064 #define Sqrt ::sqrtf
00065 #define Exp ::expf
00066 #define Mod ::fmodf
00067 #endif
00068 
00069     /****************************************/
00070     /****************************************/
00071 
00077     template<typename T> T Min(const T& t_v1, const T& t_v2) {
00078         return t_v1 < t_v2 ? t_v1 : t_v2;
00079     }
00080 
00086     template<typename T> T& Min(T& t_v1, T& t_v2) {
00087         return t_v1 < t_v2 ? t_v1 : t_v2;
00088     }
00089 
00095     template<typename T> T Max(const T& t_v1, const T& t_v2) {
00096         return t_v1 > t_v2 ? t_v1 : t_v2;
00097     }
00098 
00104     template<typename T> T& Max(T& t_v1, T& t_v2) {
00105         return t_v1 > t_v2 ? t_v1 : t_v2;
00106     }
00107 
00108     /****************************************/
00109     /****************************************/
00110 
00115     template<typename T> SInt32 Sign(const T& t_v) {
00116         if (t_v > T(0)) return 1;
00117         if (t_v < T(0)) return -1;
00118         return 0;
00119     }
00120 
00121     /****************************************/
00122     /****************************************/
00123 
00128     template<typename T> T Square(const T& t_v) {
00129         return t_v * t_v;
00130     }
00131 
00132     /****************************************/
00133     /****************************************/
00134 
00140     inline SInt32 Floor(Real f_value) {
00141         SInt32 nI = static_cast<SInt32> (f_value);
00142         if (f_value >= 0.0f) return nI;
00143         return nI - 1;
00144     }
00145 
00151     inline SInt32 Ceil(Real f_value) {
00152         SInt32 nI = static_cast<SInt32> (f_value);
00153         if (nI < f_value) return nI + 1;
00154         return nI;
00155     }
00156 
00162     inline SInt32 Round(Real f_value) {
00163         if (f_value > 0.0f) return Floor(f_value + 0.5f);
00164         return Ceil(f_value - 0.5f);
00165     }
00166 
00172     inline SInt32 RoundClosestToZero(Real f_value) {
00173         if (f_value > 0.0f) return Floor(f_value);
00174         return Ceil(f_value);
00175     }
00176 
00177     /****************************************/
00178     /****************************************/
00179 
00194     inline bool DoubleEqAbsolute(Real f_value1, Real f_value2, Real f_epsilon) {
00195         return Abs<Real > (f_value1 - f_value2) <= f_epsilon * Max<Real > (1.0f, Max<Real > (Abs<Real > (f_value1), Abs<Real > (f_value2)));
00196     }
00197 
00206     inline bool DoubleEq(Real f_value1, Real f_value2) {
00207         return Abs<Real > (f_value1 - f_value2) <= 0.0001f * Max<Real > (1.0f, Max<Real > (Abs<Real > (f_value1), Abs<Real > (f_value2)));
00208     }
00209 
00210     /****************************************/
00211     /****************************************/
00212 
00219     inline Real Interpolate(Real f_x, const std::vector<std::pair<Real, Real> >& c_points) {
00220         std::pair<Real, Real> cP0 = c_points.at(0);
00221         std::pair<Real, Real> cP1;
00222         for (UInt32 i = 1; i < c_points.size(); ++i) {
00223             cP1 = c_points.at(i);
00224             if (cP1.first >= f_x) {
00225                 break;
00226             } else if (i < c_points.size() - 1) {
00227                 cP0 = cP1;
00228             }
00229         }
00230         return (f_x * (cP0.second - cP1.second)) / (cP0.first - cP1.first)+(-cP1.first * cP0.second + cP0.first * cP1.second) / (cP0.first - cP1.first);;
00231     }
00232 }
00233 
00234 #endif
```

---

Generated on 10 Jul 2018 for ARGoS by 
 1.6.1 
