# Supplementary material for: Iterative improvement in the automatic modular design of robot swarms
Source: PeerJ Comput Sci. 2020 Dec 7;6:e322. doi: 10.7717/peerj-cs.322 (PMC7924708; doi:10.7717/peerj-cs.322)
Supplement: Supplemental Information 3 [file peerj-cs-06-322-s003.zip › argos3/doc/api/standalone/a00372_source.html]

ARGoS: core/utility/math/matrix/matrix.h Source File


- Main Page
- Related Pages
- Namespaces
- Classes
- Files

- File List
- File Members

# core/utility/math/matrix/matrix.h

Go to the documentation of this file.

```
00001 
00009 #ifndef MATRIX_H
00010 #define MATRIX_H
00011 
00012 #include <argos3/core/utility/math/general.h>
00013 #include <argos3/core/utility/math/angles.h>
00014 #include <cmath>
00015 #include <iomanip>
00016 
00017 namespace argos {
00018 
00019    template <UInt32 ROWS, UInt32 COLS>
00020    class CMatrix {
00021 
00022    /* Matrices of different dimensions can access each others data */
00023    template <UInt32 SMROWS, UInt32 SMCOLS> friend class CMatrix;
00024    
00025    /* The specified derived classes are allowed to access members of the base class */
00026    friend class CRotationMatrix2;
00027    friend class CTransformationMatrix2;
00028    friend class CRotationMatrix3;
00029    friend class CTransformationMatrix3;
00030       
00031    public:
00032       CMatrix() {
00033          for(UInt32 i = 0; i < ROWS * COLS; i++)
00034             m_pfValues[i] = 0;
00035       }
00036       
00037       CMatrix(const Real* pf_values) {
00038          Set(pf_values);
00039       }
00040       
00041       CMatrix(const CMatrix<ROWS,COLS>& c_matrix) {
00042          Set(c_matrix.m_pfValues);
00043       }
00044       
00045       CMatrix<ROWS,COLS>& operator=(const CMatrix<ROWS,COLS>& c_matrix) {
00046          if(this != &c_matrix) {
00047             Set(c_matrix.m_pfValues);
00048          }
00049          return *this;
00050       }
00051           
00052       inline Real& operator()(UInt32 un_row,
00053                               UInt32 un_col) {
00054          ARGOS_ASSERT(un_row < ROWS && un_col < COLS,
00055                       "Matrix index out of bounds: un_row = " <<
00056                       un_row <<
00057                       ", un_col = " <<
00058                       un_col);
00059          return m_pfValues[un_row * COLS + un_col];
00060       }
00061       
00062       inline Real operator()(UInt32 un_row,
00063                              UInt32 un_col) const {
00064          ARGOS_ASSERT(un_row < ROWS && un_col < COLS,
00065                       "Matrix index out of bounds: un_row = " <<
00066                       un_row <<
00067                       ", un_col = " <<
00068                       un_col);
00069          return m_pfValues[un_row * COLS + un_col];
00070       }
00071       
00072       inline Real operator()(UInt32 un_idx) const {
00073          ARGOS_ASSERT(un_idx < ROWS * COLS,
00074                       "Matrix index out of bounds: un_idx = " <<
00075                       un_idx);
00076          return m_pfValues[un_idx];
00077       }
00078 
00079       inline Real& operator()(UInt32 un_idx) {
00080          ARGOS_ASSERT(un_idx < ROWS * COLS,
00081                       "Matrix index out of bounds: un_idx = " <<
00082                       un_idx);
00083          return m_pfValues[un_idx];
00084       }
00085       
00086       void Set(const Real* f_values) {
00087          for(UInt32 i = 0; i < ROWS * COLS; i++)
00088             m_pfValues[i] = f_values[i];
00089       }
00090       
00091       CMatrix<COLS, ROWS> GetTransposed() {
00092          Real fNewValues[COLS * ROWS];
00093          for(UInt32 i = 0; i < ROWS; i++)
00094             for(UInt32 j = 0; j < COLS; j++)
00095                fNewValues[j * ROWS + i] = m_pfValues[i * COLS + j];
00096 
00097          return CMatrix<COLS, ROWS>(fNewValues);
00098       }
00099       
00100       template <UInt32 SMROWS, UInt32 SMCOLS>
00101       CMatrix<SMROWS, SMCOLS>& GetSubmatrix(CMatrix<SMROWS, SMCOLS>& c_matrix,
00102                                             UInt32 un_offset_row, 
00103                                             UInt32 un_offset_col) {
00104          ARGOS_ASSERT((SMROWS - 1 + un_offset_row) < ROWS &&
00105                       (SMCOLS - 1 + un_offset_col) < COLS,
00106                       "Submatrix range is out of bounds: cannot extract a " <<
00107                       SMROWS << "x" << SMCOLS << " submatrix from a " <<
00108                       ROWS << "x" << COLS << " matrix with offsets " <<
00109                       " un_offset_row = " <<
00110                       un_offset_row <<
00111                       ", un_offset_col = " <<
00112                       un_offset_col);
00113                       
00114          for(UInt32 i = 0; i < SMROWS; i++)
00115             for(UInt32 j = 0; j < SMCOLS; j++)
00116                c_matrix.m_pfValues[i * SMCOLS + j] = m_pfValues[(i + un_offset_row) * COLS + (j + un_offset_col)];
00117          
00118          return c_matrix;
00119       }
00120       
00121       bool operator==(const CMatrix<ROWS, COLS>& c_matrix) const {
00122          for(UInt32 i = 0; i < ROWS * COLS; i++) {
00123             if(m_pfValues[i] != c_matrix.m_pfValues[i])
00124                return false;
00125          }
00126          return true;
00127       }
00128 
00129       CMatrix<ROWS, COLS>& operator+=(const CMatrix<ROWS, COLS>& c_matrix) {
00130          for(UInt32 i = 0; i < ROWS * COLS; i++) {
00131             m_pfValues[i] += c_matrix.m_pfValues[i];
00132          }
00133          return *this;
00134       }
00135       
00136       CMatrix<ROWS, COLS>& operator-=(const CMatrix<ROWS, COLS>& c_matrix) {
00137          for(UInt32 i = 0; i < ROWS * COLS; i++) {
00138             m_pfValues[i] -= c_matrix.m_pfValues[i];
00139          }
00140          return *this;
00141       }
00142       
00143       CMatrix<ROWS, COLS>& operator*=(Real f_scale) {
00144          for(UInt32 i = 0; i < ROWS * COLS; i++) {
00145             m_pfValues[i] *= f_scale;
00146          }
00147          return *this;
00148       }
00149       
00150       CMatrix<ROWS, COLS> operator+(const CMatrix<ROWS, COLS>& c_matrix) const {
00151          CMatrix<ROWS, COLS> cResult = (*this);
00152          cResult += c_matrix;
00153          return cResult;
00154       }
00155 
00156       CMatrix<ROWS, COLS> operator-(const CMatrix<ROWS, COLS>& c_matrix) const {
00157          CMatrix<ROWS, COLS> cResult = (*this);
00158          cResult -= c_matrix;
00159          return cResult;
00160       }
00161       
00162       CMatrix<ROWS, COLS>& operator*=(const CMatrix<COLS, COLS>& c_matrix) {
00163          Real fNewValues[ROWS * COLS];
00164          for(UInt32 i = 0; i < ROWS; i++) {
00165             for(UInt32 j = 0; j < COLS; j++) {
00166                fNewValues[i * COLS + j] = 0.0f;
00167                for(UInt32 k = 0; k < COLS; k++) {
00168                   fNewValues[i * COLS + j] += m_pfValues[i * COLS + k] * c_matrix.m_pfValues[k * COLS + j];
00169                }
00170             }
00171          }
00172          Set(fNewValues);
00173          return *this;
00174       }
00175       
00176       template <UInt32 OTRCOLS>
00177       CMatrix<ROWS, OTRCOLS> operator*(const CMatrix<COLS, OTRCOLS>& c_matrix) const {
00178          Real fNewValues[ROWS * OTRCOLS];
00179          for(UInt32 i = 0; i < ROWS; i++) {
00180             for(UInt32 j = 0; j < OTRCOLS; j++) {
00181                fNewValues[i * OTRCOLS + j] = 0.0f;
00182                for(UInt32 k = 0; k < COLS; k++) {
00183                   fNewValues[i * OTRCOLS + j] += m_pfValues[i * COLS + k] * c_matrix.m_pfValues[k * OTRCOLS + j];
00184                }
00185             }
00186          }
00187          return CMatrix<ROWS, OTRCOLS>(fNewValues);
00188       }
00189 
00190       friend std::ostream& operator<<(std::ostream& c_os,
00191                                       const CMatrix& c_matrix) {
00192          
00193          std::ios_base::fmtflags unInitalFlags = c_os.flags();
00194          std::streamsize nInitalPrec = c_os.precision();
00195          
00196          c_os.setf(std::ios::fixed);
00197          c_os.precision(1);
00198          
00199          for(UInt32 i = 0; i < ROWS; i++) {
00200             c_os << "| ";
00201             for(UInt32 j = 0; j < COLS; j++) {
00202                c_os << std::setw(6) << c_matrix(i, j) << " ";
00203             }
00204             c_os << "|" << std::endl;
00205          }
00206          
00207          c_os.flags(unInitalFlags);
00208          c_os.precision(nInitalPrec);
00209          return c_os;
00210       }
00211       
00212    protected:
00213 
00214       Real m_pfValues[ROWS * COLS];
00215 
00216    };
00217 }
00218 
00219 #endif
```

---

Generated on 10 Jul 2018 for ARGoS by 
 1.6.1 
