# Supplementary material for: Iterative improvement in the automatic modular design of robot swarms
Source: PeerJ Comput Sci. 2020 Dec 7;6:e322. doi: 10.7717/peerj-cs.322 (PMC7924708; doi:10.7717/peerj-cs.322)
Supplement: Supplemental Information 3 [file peerj-cs-06-322-s003.zip › argos3/doc/api/standalone/a00373_source.html]

ARGoS: core/utility/math/matrix/rotationmatrix2.cpp Source File


- Main Page
- Related Pages
- Namespaces
- Classes
- Files

- File List
- File Members

# core/utility/math/matrix/rotationmatrix2.cpp

Go to the documentation of this file.

```
00001 
00009 #include "rotationmatrix2.h"
00010 #include <argos3/core/utility/math/angles.h>
00011 
00012 namespace argos {
00013 
00014    /****************************************/
00015    /****************************************/
00016 
00017    void CRotationMatrix2::SetFromAngle(const CRadians& c_angle) {
00018       Real cos_angle = Cos(c_angle);
00019       Real sin_angle = Sin(c_angle);
00020 
00021       /* Set values */
00022       m_pfValues[0] =  cos_angle;
00023       m_pfValues[1] = -sin_angle;
00024       m_pfValues[2] =  sin_angle;
00025       m_pfValues[3] =  cos_angle;
00026    }
00027    
00028    /****************************************/
00029    /****************************************/
00030    
00031    void CRotationMatrix2::SetFromMatrix(const CMatrix<2,2>& c_matrix) {
00032       m_pfValues[0] =  c_matrix.m_pfValues[0];
00033       m_pfValues[1] =  c_matrix.m_pfValues[1];
00034       m_pfValues[2] =  c_matrix.m_pfValues[2];
00035       m_pfValues[3] =  c_matrix.m_pfValues[3];
00036    }
00037    
00038    /****************************************/
00039    /****************************************/
00040    
00041    void CRotationMatrix2::SetFromValues(Real f_value0, Real f_value1,
00042                                         Real f_value2, Real f_value3) {
00043       m_pfValues[0] = f_value0;
00044       m_pfValues[1] = f_value1;
00045       m_pfValues[2] = f_value2;
00046       m_pfValues[3] = f_value3;
00047    }
00048    
00049    /****************************************/
00050    /****************************************/
00051 
00052 }
```

---

Generated on 10 Jul 2018 for ARGoS by 
 1.6.1 
