# Supplementary material for: Iterative improvement in the automatic modular design of robot swarms
Source: PeerJ Comput Sci. 2020 Dec 7;6:e322. doi: 10.7717/peerj-cs.322 (PMC7924708; doi:10.7717/peerj-cs.322)
Supplement: Supplemental Information 3 [file peerj-cs-06-322-s003.zip › argos3/doc/api/standalone/a00374_source.html]

ARGoS: core/utility/math/matrix/rotationmatrix2.h Source File


- Main Page
- Related Pages
- Namespaces
- Classes
- Files

- File List
- File Members

# core/utility/math/matrix/rotationmatrix2.h

Go to the documentation of this file.

```
00001 
00009 #ifndef ROTATION_MATRIX2_H
00010 #define ROTATION_MATRIX2_H
00011 
00012 namespace argos {
00013    class CRadians;
00014 }
00015 
00016 #include "squarematrix.h"
00017 #include <argos3/core/utility/math/vector2.h>
00018 
00019 namespace argos {
00020 
00021    class CRotationMatrix2 : public CSquareMatrix<2> {
00022    
00023    friend class CTransformationMatrix2;
00024       
00025    public:
00026       CRotationMatrix2() : CSquareMatrix<2>() {
00027          SetIdentityMatrix();
00028       }
00029       
00030       CRotationMatrix2(const CMatrix<2,2>& c_matrix) {
00031          SetFromMatrix(c_matrix);
00032       }
00033       
00034       CRotationMatrix2(const CRadians& c_angle) : CSquareMatrix<2>() {
00035          SetFromAngle(c_angle);
00036       }
00037       
00038       CRotationMatrix2(Real f_value0, Real f_value1,
00039                        Real f_value2, Real f_value3) : CSquareMatrix<2>() {
00040          SetFromValues(f_value0, f_value1,
00041                        f_value2, f_value3);
00042       }
00043       
00044       void SetFromMatrix(const CMatrix<2,2>& c_matrix);
00045       
00046       void SetFromAngle(const CRadians& c_angle);
00047       
00048       void SetFromValues(Real f_value0, Real f_value1,
00049                          Real f_value2, Real f_value3);
00050       
00051       CVector2 operator*(const CVector2& c_vector) const {
00052          return CVector2(m_pfValues[0]*c_vector.m_fX + m_pfValues[1]*c_vector.m_fY,
00053                                    m_pfValues[2]*c_vector.m_fX + m_pfValues[3]*c_vector.m_fY);
00054       }
00055    };
00056 }
00057 
00058 #endif
```

---

Generated on 10 Jul 2018 for ARGoS by 
 1.6.1 
