# Supplementary material for: Iterative improvement in the automatic modular design of robot swarms
Source: PeerJ Comput Sci. 2020 Dec 7;6:e322. doi: 10.7717/peerj-cs.322 (PMC7924708; doi:10.7717/peerj-cs.322)
Supplement: Supplemental Information 3 [file peerj-cs-06-322-s003.zip › argos3/doc/api/standalone/a00375_source.html]

ARGoS: core/utility/math/matrix/rotationmatrix3.cpp Source File


- Main Page
- Related Pages
- Namespaces
- Classes
- Files

- File List
- File Members

# core/utility/math/matrix/rotationmatrix3.cpp

Go to the documentation of this file.

```
00001 
00009 #include "rotationmatrix3.h"
00010 #include <argos3/core/utility/math/quaternion.h>
00011 #include <argos3/core/utility/math/vector3.h>
00012 
00013 namespace argos {
00014 
00015    CQuaternion CRotationMatrix3::ToQuaternion() const
00016    {
00017            const Real trace = 1.0f + m_pfValues[0] + m_pfValues[4] + m_pfValues[8];
00018 
00019            if (trace > 0.00001f) {
00020                    const Real s = Sqrt(trace) * 2.0f;
00021                    return CQuaternion(
00022                            s / 4.0f,
00023                            (m_pfValues[7] - m_pfValues[5]) / s,
00024                            (m_pfValues[2] - m_pfValues[6]) / s,
00025                            (m_pfValues[3] - m_pfValues[1]) / s);
00026            }
00027            else if (m_pfValues[0] > m_pfValues[4] && m_pfValues[0] > m_pfValues[8]) {
00028                    const Real s = Sqrt(1.0f + m_pfValues[0] - m_pfValues[4] - m_pfValues[8]) * 2.0f;
00029                    return CQuaternion(
00030                            (m_pfValues[7] - m_pfValues[5]) / s,
00031                            s / 4.0f,
00032                            (m_pfValues[3] + m_pfValues[1]) / s,
00033                            (m_pfValues[2] + m_pfValues[6]) / s);
00034            }
00035            else if (m_pfValues[4] > m_pfValues[8]) {
00036                    const Real s = Sqrt(1.0f + m_pfValues[4] - m_pfValues[0] - m_pfValues[8]) * 2.0f;
00037                    return CQuaternion(
00038                            (m_pfValues[2] - m_pfValues[6]) / s,
00039                            (m_pfValues[3] + m_pfValues[1]) / s,
00040                            s / 4.0f,
00041                            (m_pfValues[7] + m_pfValues[5]) / s);
00042            }
00043            else {
00044                    const Real s = Sqrt(1.0f + m_pfValues[8] - m_pfValues[0] - m_pfValues[4]) * 2.0f;
00045                    return CQuaternion(
00046                            (m_pfValues[3] - m_pfValues[1]) / s,
00047                            (m_pfValues[2] + m_pfValues[6]) / s,
00048                            (m_pfValues[7] + m_pfValues[5]) / s,
00049                            s / 4.0f);
00050            }
00051    }
00052 
00053    /****************************************/
00054    /****************************************/
00055 
00056    void CRotationMatrix3::SetFromMatrix(const CMatrix<3,3>& c_matrix) {
00057       m_pfValues[0] = c_matrix.m_pfValues[0];
00058       m_pfValues[1] = c_matrix.m_pfValues[1];
00059       m_pfValues[2] = c_matrix.m_pfValues[2];
00060       m_pfValues[3] = c_matrix.m_pfValues[3];
00061       m_pfValues[4] = c_matrix.m_pfValues[4];
00062       m_pfValues[5] = c_matrix.m_pfValues[5];
00063       m_pfValues[6] = c_matrix.m_pfValues[6];
00064       m_pfValues[7] = c_matrix.m_pfValues[7];
00065       m_pfValues[8] = c_matrix.m_pfValues[8];
00066    }
00067    
00068    /****************************************/
00069    /****************************************/
00070 
00071    void CRotationMatrix3::SetFromQuaternion(const CQuaternion& c_quaternion) {
00072       /* This code is adapted from the Bullet source code */
00073       Real d = c_quaternion.SquareLength();
00074       Real s = 2.0f / d;
00075       Real xs = c_quaternion.GetX() * s,   ys = c_quaternion.GetY() * s,   zs = c_quaternion.GetZ() * s;
00076       Real wx = c_quaternion.GetW() * xs,  wy = c_quaternion.GetW() * ys,  wz = c_quaternion.GetW() * zs;
00077       Real xx = c_quaternion.GetX() * xs,  xy = c_quaternion.GetX() * ys,  xz = c_quaternion.GetX() * zs;
00078       Real yy = c_quaternion.GetY() * ys,  yz = c_quaternion.GetY() * zs,  zz = c_quaternion.GetZ() * zs;
00079       
00080       /* Set values */
00081       m_pfValues[0] = 1.0f - (yy + zz);
00082       m_pfValues[1] = xy - wz;
00083       m_pfValues[2] = xz + wy;
00084       m_pfValues[3] = xy + wz;
00085       m_pfValues[4] = 1.0f - (xx + zz);
00086       m_pfValues[5] = yz - wx;
00087       m_pfValues[6] = xz - wy;
00088       m_pfValues[7] = yz + wx;
00089       m_pfValues[8] = 1.0f - (xx + yy);
00090    }
00091 
00092    /****************************************/
00093    /****************************************/
00094    
00095   void CRotationMatrix3::SetFromAngles(const CRadians& c_z_angle, const CRadians& c_y_angle, const CRadians& c_x_angle) {
00096       Real fSinX = Sin(c_x_angle);
00097       Real fCosX = Cos(c_x_angle);
00098       Real fSinY = Sin(c_y_angle);
00099       Real fCosY = Cos(c_y_angle);
00100       Real fSinZ = Sin(c_z_angle);
00101       Real fCosZ = Cos(c_z_angle);
00102       
00103       m_pfValues[0] = (fCosZ * fCosY);
00104       m_pfValues[1] = (fCosZ * fSinY * fSinX) - (fCosX * fSinZ);
00105       m_pfValues[2] = (fSinZ * fSinX) + (fCosZ * fCosX * fSinY);
00106       m_pfValues[3] = (fCosY * fSinZ);
00107       m_pfValues[4] = (fCosZ * fCosX) + (fSinZ * fSinY * fSinX);
00108       m_pfValues[5] = (fCosX * fSinZ * fSinY) - (fCosZ * fSinX);
00109       m_pfValues[6] = -fSinY;
00110       m_pfValues[7] = (fCosY * fSinX);
00111       m_pfValues[8] = (fCosY * fCosX);
00112    }
00113    
00114    /****************************************/
00115    /****************************************/
00116    
00117    void CRotationMatrix3::SetFromValues(Real f_value0, Real f_value1, Real f_value2,
00118                                         Real f_value3, Real f_value4, Real f_value5,
00119                                         Real f_value6, Real f_value7, Real f_value8) {
00120       m_pfValues[0] = f_value0;
00121       m_pfValues[1] = f_value1;
00122       m_pfValues[2] = f_value2;
00123       m_pfValues[3] = f_value3;
00124       m_pfValues[4] = f_value4;
00125       m_pfValues[5] = f_value5;
00126       m_pfValues[6] = f_value6;
00127       m_pfValues[7] = f_value7;
00128       m_pfValues[8] = f_value8;
00129    }
00130 
00131    /****************************************/
00132    /****************************************/
00133    
00134    CVector3 CRotationMatrix3::operator*(const CVector3& c_vector) const {
00135       return CVector3(m_pfValues[0]*c_vector.m_fX + m_pfValues[1]*c_vector.m_fY + m_pfValues[2]*c_vector.m_fZ,
00136                            m_pfValues[3]*c_vector.m_fX + m_pfValues[4]*c_vector.m_fY + m_pfValues[5]*c_vector.m_fZ,
00137                            m_pfValues[6]*c_vector.m_fX + m_pfValues[7]*c_vector.m_fY + m_pfValues[8]*c_vector.m_fZ);
00138    }
00139       
00140    /****************************************/
00141    /****************************************/
00142 }
```

---

Generated on 10 Jul 2018 for ARGoS by 
 1.6.1 
