# Supplementary material for: Iterative improvement in the automatic modular design of robot swarms
Source: PeerJ Comput Sci. 2020 Dec 7;6:e322. doi: 10.7717/peerj-cs.322 (PMC7924708; doi:10.7717/peerj-cs.322)
Supplement: Supplemental Information 3 [file peerj-cs-06-322-s003.zip › argos3/doc/api/standalone/a00376_source.html]

ARGoS: core/utility/math/matrix/rotationmatrix3.h Source File


- Main Page
- Related Pages
- Namespaces
- Classes
- Files

- File List
- File Members

# core/utility/math/matrix/rotationmatrix3.h

Go to the documentation of this file.

```
00001 
00009 #ifndef ROTATION_MATRIX3_H
00010 #define ROTATION_MATRIX3_H
00011 
00012 namespace argos {
00013    class CQuaternion;
00014    class CVector3;
00015 }
00016 
00017 #include "squarematrix.h"
00018 
00019 namespace argos {
00020 
00021    class CRotationMatrix3 : public CSquareMatrix<3> {
00022    
00023    friend class CTransformationMatrix3;
00024    
00025    public:
00026       CRotationMatrix3() : CSquareMatrix<3>() {
00027          SetIdentityMatrix();
00028       }
00029       
00030       CRotationMatrix3(const CMatrix<3,3>& c_matrix) : CSquareMatrix<3>() {
00031          SetFromMatrix(c_matrix);
00032       }
00033       
00034       CRotationMatrix3(const CQuaternion& c_quaternion) : CSquareMatrix<3>() {
00035          SetFromQuaternion(c_quaternion);
00036       }
00037       
00038       CRotationMatrix3(const CRadians& c_z_angle, const CRadians& c_y_angle, const CRadians& c_x_angle) : CSquareMatrix<3>() {
00039          SetFromAngles(c_z_angle, c_y_angle, c_x_angle);
00040       }
00041       
00042       CRotationMatrix3(Real f_value0, Real f_value1, Real f_value2,
00043                        Real f_value3, Real f_value4, Real f_value5,
00044                        Real f_value6, Real f_value7, Real f_value8) : CSquareMatrix<3>() {
00045          SetFromValues(f_value0, f_value1, f_value2,
00046                        f_value3, f_value4, f_value5,
00047                        f_value6, f_value7, f_value8);               
00048       }
00049       
00050       void SetFromMatrix(const CMatrix<3,3>& c_matrix);
00051       
00052       void SetFromQuaternion(const CQuaternion& c_quaternion);
00053       
00054       void SetFromAngles(const CRadians& c_x_angle, const CRadians& c_y_angle, const CRadians& c_z_angle);
00055       
00056       void SetFromValues(Real f_value0, Real f_value1, Real f_value2,
00057                          Real f_value3, Real f_value4, Real f_value5,
00058                          Real f_value6, Real f_value7, Real f_value8);
00059       
00060       CQuaternion ToQuaternion() const;
00061       
00062       CVector3 operator*(const CVector3& c_vector) const;
00063    };
00064 }
00065 
00066 #endif
```

---

Generated on 10 Jul 2018 for ARGoS by 
 1.6.1 
