# Supplementary material for: Iterative improvement in the automatic modular design of robot swarms
Source: PeerJ Comput Sci. 2020 Dec 7;6:e322. doi: 10.7717/peerj-cs.322 (PMC7924708; doi:10.7717/peerj-cs.322)
Supplement: Supplemental Information 3 [file peerj-cs-06-322-s003.zip › argos3/doc/api/standalone/a00377_source.html]

ARGoS: core/utility/math/matrix/squarematrix.h Source File


- Main Page
- Related Pages
- Namespaces
- Classes
- Files

- File List
- File Members

# core/utility/math/matrix/squarematrix.h

Go to the documentation of this file.

```
00001 
00009 #ifndef SQUARE_MATRIX_H
00010 #define SQUARE_MATRIX_H
00011 
00012 #include "matrix.h"
00013 
00014 namespace argos {
00015 
00016    template <UInt32 DIM>
00017    class CSquareMatrix : public CMatrix<DIM, DIM> {
00018       
00019    public:
00020       CSquareMatrix() : CMatrix<DIM, DIM>() {}
00021       
00022       void SetIdentityMatrix() {
00023          for(UInt32 i = 0; i < DIM; i++) {
00024             for(UInt32 j = 0; j < DIM; j++) {
00025                if(i == j) {
00026                   CMatrix<DIM, DIM>::m_pfValues[i * DIM + j] = 1;
00027                }
00028                else {
00029                   CMatrix<DIM, DIM>::m_pfValues[i * DIM + j] = 0;
00030                }
00031             }
00032          }
00033       }
00034       
00035       bool IsIdentityMatrix() {
00036          bool bIsIdentMat = true;
00037          for(UInt32 i = 0; i < DIM; i++) {
00038             for(UInt32 j = 0; j < DIM; j++) {
00039                if(i == j) {
00040                   if(CMatrix<DIM, DIM>::m_pfValues[i * DIM + j] != 1) {
00041                      bIsIdentMat = false;
00042                      break;
00043                   }
00044                }
00045                else {
00046                   if(CMatrix<DIM, DIM>::m_pfValues[i * DIM + j] != 0) {
00047                      bIsIdentMat = false;
00048                      break;
00049                   }
00050                }
00051             }
00052          }
00053          return bIsIdentMat;
00054       }
00055    };
00056 }
00057 
00058 #endif
```

---

Generated on 10 Jul 2018 for ARGoS by 
 1.6.1 
