# Supplementary material for: Iterative improvement in the automatic modular design of robot swarms
Source: PeerJ Comput Sci. 2020 Dec 7;6:e322. doi: 10.7717/peerj-cs.322 (PMC7924708; doi:10.7717/peerj-cs.322)
Supplement: Supplemental Information 3 [file peerj-cs-06-322-s003.zip › argos3/doc/api/standalone/a00378_source.html]

ARGoS: core/utility/math/matrix/transformationmatrix2.cpp Source File


- Main Page
- Related Pages
- Namespaces
- Classes
- Files

- File List
- File Members

# core/utility/math/matrix/transformationmatrix2.cpp

Go to the documentation of this file.

```
00001 
00009 #include "transformationmatrix2.h"
00010 #include "rotationmatrix2.h"
00011 #include <argos3/core/utility/math/vector2.h>
00012 
00013 namespace argos {
00014 
00015    void CTransformationMatrix2::SetFromComponents(const CRotationMatrix2& c_rotation, const CVector2& c_translation) {
00016       /* Set the rotation elements */
00017       SetRotationMatrix(c_rotation);
00018       
00019       /* Set the translation elements */
00020       SetTranslationVector(c_translation);
00021       
00022       /* Set the bottom row elements */
00023       m_pfValues[6] = 0.0f; m_pfValues[7] = 0.0f; m_pfValues[8] = 1.0f;
00024    }
00025    
00026    void CTransformationMatrix2::SetFromMatrix(const CMatrix<3,3>& c_matrix) {
00027       m_pfValues[0] = c_matrix.m_pfValues[0];
00028       m_pfValues[1] = c_matrix.m_pfValues[1];
00029       m_pfValues[2] = c_matrix.m_pfValues[2];
00030       m_pfValues[3] = c_matrix.m_pfValues[3];
00031       m_pfValues[4] = c_matrix.m_pfValues[4];
00032       m_pfValues[5] = c_matrix.m_pfValues[5];
00033       m_pfValues[6] = c_matrix.m_pfValues[6];
00034       m_pfValues[7] = c_matrix.m_pfValues[7];
00035       m_pfValues[8] = c_matrix.m_pfValues[8];
00036    }
00037    
00038    void CTransformationMatrix2::SetFromValues(Real f_value0, Real f_value1, Real f_value2,
00039                                               Real f_value3, Real f_value4, Real f_value5,
00040                                               Real f_value6, Real f_value7, Real f_value8) {
00041       m_pfValues[0] = f_value0;
00042       m_pfValues[1] = f_value1;
00043       m_pfValues[2] = f_value2;
00044       m_pfValues[3] = f_value3;
00045       m_pfValues[4] = f_value4;
00046       m_pfValues[5] = f_value5;
00047       m_pfValues[6] = f_value6;
00048       m_pfValues[7] = f_value7;
00049       m_pfValues[8] = f_value8;
00050    }
00051    
00052    void CTransformationMatrix2::SetRotationMatrix(const CRotationMatrix2& c_rotation) {
00053       m_pfValues[0] = c_rotation.m_pfValues[0]; m_pfValues[1] = c_rotation.m_pfValues[1];
00054       m_pfValues[3] = c_rotation.m_pfValues[2]; m_pfValues[4] = c_rotation.m_pfValues[3];
00055    }
00056    
00057    const CRotationMatrix2 CTransformationMatrix2::GetRotationMatrix() const {
00058       return CRotationMatrix2(m_pfValues[0], m_pfValues[1],
00059                               m_pfValues[3], m_pfValues[4]);
00060    }
00061    
00062    void CTransformationMatrix2::SetTranslationVector(const CVector2& c_translation) {
00063       m_pfValues[2] = c_translation.m_fX;
00064       m_pfValues[5] = c_translation.m_fY;
00065    }
00066    
00067    const CVector2 CTransformationMatrix2::GetTranslationVector() const {
00068       return CVector2(m_pfValues[2], m_pfValues[5]);
00069    }
00070 
00071    /****************************************/
00072    /****************************************/
00073 }
```

---

Generated on 10 Jul 2018 for ARGoS by 
 1.6.1 
