# Supplementary material for: Iterative improvement in the automatic modular design of robot swarms
Source: PeerJ Comput Sci. 2020 Dec 7;6:e322. doi: 10.7717/peerj-cs.322 (PMC7924708; doi:10.7717/peerj-cs.322)
Supplement: Supplemental Information 3 [file peerj-cs-06-322-s003.zip › argos3/doc/api/standalone/a00379_source.html]

ARGoS: core/utility/math/matrix/transformationmatrix2.h Source File


- Main Page
- Related Pages
- Namespaces
- Classes
- Files

- File List
- File Members

# core/utility/math/matrix/transformationmatrix2.h

Go to the documentation of this file.

```
00001 
00009 #ifndef TRANSFORMATION_MATRIX2_H
00010 #define TRANSFORMATION_MATRIX2_H
00011 
00012 namespace argos {
00013    class CVector2;
00014    class CRotationMatrix2;
00015 }
00016 
00017 #include "squarematrix.h"
00018 
00019 namespace argos {
00020 
00021    class CTransformationMatrix2 : public CSquareMatrix<3> {
00022       
00023    public:
00024       CTransformationMatrix2() : CSquareMatrix<3>() {
00025          SetIdentityMatrix();
00026       }
00027       
00028       CTransformationMatrix2(const CMatrix<3,3>& c_matrix) : CSquareMatrix<3>() {
00029          SetFromMatrix(c_matrix);
00030       }
00031       
00032       CTransformationMatrix2(const CRotationMatrix2& c_rotation, const CVector2& c_translation) : CSquareMatrix<3>() {
00033          SetFromComponents(c_rotation, c_translation);
00034       }
00035       
00036       CTransformationMatrix2(Real f_value0, Real f_value1, Real f_value2,
00037                              Real f_value3, Real f_value4, Real f_value5,
00038                              Real f_value6, Real f_value7, Real f_value8) : CSquareMatrix<3>() {
00039          SetFromValues(f_value0, f_value1, f_value2,
00040                        f_value3, f_value4, f_value5,
00041                        f_value6, f_value7, f_value8);               
00042       }
00043       
00044       void SetFromMatrix(const CMatrix<3,3>& c_matrix);
00045       
00046       void SetFromComponents(const CRotationMatrix2& c_rotation, const CVector2& c_translation);
00047       
00048       void SetFromValues(Real f_value0, Real f_value1, Real f_value2,
00049                          Real f_value3, Real f_value4, Real f_value5,
00050                          Real f_value6, Real f_value7, Real f_value8);
00051       
00052       void SetRotationMatrix(const CRotationMatrix2& c_rotation);
00053       
00054       const CRotationMatrix2 GetRotationMatrix() const;
00055       
00056       void SetTranslationVector(const CVector2& c_translation);
00057       
00058       const CVector2 GetTranslationVector() const;
00059    };
00060 }
00061 
00062 #endif
```

---

Generated on 10 Jul 2018 for ARGoS by 
 1.6.1 
