# Supplementary material for: Iterative improvement in the automatic modular design of robot swarms
Source: PeerJ Comput Sci. 2020 Dec 7;6:e322. doi: 10.7717/peerj-cs.322 (PMC7924708; doi:10.7717/peerj-cs.322)
Supplement: Supplemental Information 3 [file peerj-cs-06-322-s003.zip › argos3/doc/api/standalone/a00380_source.html]

ARGoS: core/utility/math/matrix/transformationmatrix3.cpp Source File


- Main Page
- Related Pages
- Namespaces
- Classes
- Files

- File List
- File Members

# core/utility/math/matrix/transformationmatrix3.cpp

Go to the documentation of this file.

```
00001 
00009 #include "transformationmatrix3.h"
00010 #include "rotationmatrix3.h"
00011 #include <argos3/core/utility/math/vector3.h>
00012 
00013 namespace argos {
00014 
00015    /****************************************/
00016    /****************************************/   
00017 
00018    void CTransformationMatrix3::SetFromComponents(const CRotationMatrix3& c_rotation, const CVector3& c_translation) {
00019       /* Set the rotation elements */
00020       SetRotationMatrix(c_rotation);
00021       
00022       /* Set the translation elements */
00023       SetTranslationVector(c_translation);
00024       
00025       /* Set the bottom row elements */
00026       m_pfValues[12] = 0.0f; m_pfValues[13] = 0.0f; m_pfValues[14] = 0.0f; m_pfValues[15] = 1.0f;
00027    }
00028 
00029    /****************************************/
00030    /****************************************/   
00031 
00032    void CTransformationMatrix3::SetFromMatrix(const CMatrix<4,4>& c_matrix) {
00033       m_pfValues[ 0] = c_matrix.m_pfValues[ 0];
00034       m_pfValues[ 1] = c_matrix.m_pfValues[ 1];
00035       m_pfValues[ 2] = c_matrix.m_pfValues[ 2];
00036       m_pfValues[ 3] = c_matrix.m_pfValues[ 3];
00037       m_pfValues[ 4] = c_matrix.m_pfValues[ 4];
00038       m_pfValues[ 5] = c_matrix.m_pfValues[ 5];
00039       m_pfValues[ 6] = c_matrix.m_pfValues[ 6];
00040       m_pfValues[ 7] = c_matrix.m_pfValues[ 7];
00041       m_pfValues[ 8] = c_matrix.m_pfValues[ 8];
00042       m_pfValues[ 9] = c_matrix.m_pfValues[ 9];
00043       m_pfValues[10] = c_matrix.m_pfValues[10];
00044       m_pfValues[11] = c_matrix.m_pfValues[11];
00045       m_pfValues[12] = c_matrix.m_pfValues[12];
00046       m_pfValues[13] = c_matrix.m_pfValues[13];
00047       m_pfValues[14] = c_matrix.m_pfValues[14];
00048       m_pfValues[15] = c_matrix.m_pfValues[15];
00049    }
00050    
00051    /****************************************/
00052    /****************************************/   
00053 
00054    void CTransformationMatrix3::SetFromValues(Real f_value0,  Real f_value1,  Real f_value2,  Real f_value3,
00055                                               Real f_value4,  Real f_value5,  Real f_value6,  Real f_value7, 
00056                                               Real f_value8,  Real f_value9,  Real f_value10, Real f_value11,
00057                                               Real f_value12, Real f_value13, Real f_value14, Real f_value15) {
00058       m_pfValues[ 0] = f_value0;
00059       m_pfValues[ 1] = f_value1;
00060       m_pfValues[ 2] = f_value2;
00061       m_pfValues[ 3] = f_value3;
00062       m_pfValues[ 4] = f_value4;
00063       m_pfValues[ 5] = f_value5;
00064       m_pfValues[ 6] = f_value6;
00065       m_pfValues[ 7] = f_value7;
00066       m_pfValues[ 8] = f_value8;
00067       m_pfValues[ 9] = f_value9;
00068       m_pfValues[10] = f_value10;
00069       m_pfValues[11] = f_value11;
00070       m_pfValues[12] = f_value12;
00071       m_pfValues[13] = f_value13;
00072       m_pfValues[14] = f_value14;
00073       m_pfValues[15] = f_value15;
00074    }
00075    
00076    /****************************************/
00077    /****************************************/   
00078 
00079    void CTransformationMatrix3::SetRotationMatrix(const CRotationMatrix3& c_rotation) {
00080       m_pfValues[0] = c_rotation.m_pfValues[0]; m_pfValues[1] = c_rotation.m_pfValues[1]; m_pfValues[ 2] = c_rotation.m_pfValues[2];
00081       m_pfValues[4] = c_rotation.m_pfValues[3]; m_pfValues[5] = c_rotation.m_pfValues[4]; m_pfValues[ 6] = c_rotation.m_pfValues[5];
00082       m_pfValues[8] = c_rotation.m_pfValues[6]; m_pfValues[9] = c_rotation.m_pfValues[7]; m_pfValues[10] = c_rotation.m_pfValues[8];
00083    }
00084    
00085    /****************************************/
00086    /****************************************/   
00087 
00088    const CRotationMatrix3 CTransformationMatrix3::GetRotationMatrix() const {
00089       return CRotationMatrix3(m_pfValues[0], m_pfValues[1], m_pfValues[2],
00090                               m_pfValues[4], m_pfValues[5], m_pfValues[6],
00091                               m_pfValues[8], m_pfValues[9], m_pfValues[10]);
00092    }
00093    
00094    /****************************************/
00095    /****************************************/   
00096 
00097    void CTransformationMatrix3::SetTranslationVector(const CVector3& c_translation) {
00098       m_pfValues[ 3] = c_translation.m_fX;
00099       m_pfValues[ 7] = c_translation.m_fY;
00100       m_pfValues[11] = c_translation.m_fZ; 
00101    }
00102    
00103    /****************************************/
00104    /****************************************/   
00105 
00106    const CVector3 CTransformationMatrix3::GetTranslationVector() const {
00107       return CVector3(m_pfValues[3], m_pfValues[7], m_pfValues[11]);
00108    }
00109 
00110    /****************************************/
00111    /****************************************/   
00112 
00113    CVector3 CTransformationMatrix3::operator*(const CVector3& c_vector) const {
00114       return CVector3(m_pfValues[0]*c_vector.m_fX + m_pfValues[1]*c_vector.m_fY + m_pfValues[2]*c_vector.m_fZ + m_pfValues[3],
00115                            m_pfValues[4]*c_vector.m_fX + m_pfValues[5]*c_vector.m_fY + m_pfValues[6]*c_vector.m_fZ + m_pfValues[7],
00116                            m_pfValues[8]*c_vector.m_fX + m_pfValues[9]*c_vector.m_fY + m_pfValues[10]*c_vector.m_fZ + m_pfValues[11]);
00117    }
00118 
00119    /****************************************/
00120    /****************************************/
00121 }
```

---

Generated on 10 Jul 2018 for ARGoS by 
 1.6.1 
