# Supplementary material for: Iterative improvement in the automatic modular design of robot swarms
Source: PeerJ Comput Sci. 2020 Dec 7;6:e322. doi: 10.7717/peerj-cs.322 (PMC7924708; doi:10.7717/peerj-cs.322)
Supplement: Supplemental Information 3 [file peerj-cs-06-322-s003.zip › argos3/doc/api/standalone/a00381_source.html]

ARGoS: core/utility/math/matrix/transformationmatrix3.h Source File


- Main Page
- Related Pages
- Namespaces
- Classes
- Files

- File List
- File Members

# core/utility/math/matrix/transformationmatrix3.h

Go to the documentation of this file.

```
00001 
00009 #ifndef TRANSFORMATION_MATRIX3_H
00010 #define TRANSFORMATION_MATRIX3_H
00011 
00012 namespace argos {
00013    class CVector3;
00014    class CRotationMatrix3;
00015 }
00016 
00017 #include "squarematrix.h"
00018 
00019 namespace argos {
00020 
00021    class CTransformationMatrix3 : public CSquareMatrix<4> {
00022    
00023    public:
00024      
00025       CTransformationMatrix3() : CSquareMatrix<4>() {
00026          SetIdentityMatrix();
00027       }
00028       
00029       CTransformationMatrix3(const CMatrix<4,4>& c_matrix) : CSquareMatrix<4>() {
00030          SetFromMatrix(c_matrix);
00031       }
00032       
00033       CTransformationMatrix3(const CRotationMatrix3& c_rotation, const CVector3& c_translation) : CSquareMatrix<4>() {
00034          SetFromComponents(c_rotation, c_translation);
00035       }
00036       
00037       CTransformationMatrix3(Real f_value0,  Real f_value1,  Real f_value2,  Real f_value3,
00038                              Real f_value4,  Real f_value5,  Real f_value6,  Real f_value7, 
00039                              Real f_value8,  Real f_value9,  Real f_value10, Real f_value11,
00040                              Real f_value12, Real f_value13, Real f_value14, Real f_value15) : CSquareMatrix<4>() {
00041          SetFromValues(f_value0,  f_value1,  f_value2,  f_value3,
00042                        f_value4,  f_value5,  f_value6,  f_value7, 
00043                        f_value8,  f_value9,  f_value10, f_value11,
00044                        f_value12, f_value13, f_value14, f_value15);              
00045       }
00046 
00047       void SetFromMatrix(const CMatrix<4,4>& c_matrix);
00048       
00049       void SetFromComponents(const CRotationMatrix3& c_rotation, const CVector3& c_translation);
00050       
00051       void SetFromValues(Real f_value0,  Real f_value1,  Real f_value2,  Real f_value3,
00052                          Real f_value4,  Real f_value5,  Real f_value6,  Real f_value7, 
00053                          Real f_value8,  Real f_value9,  Real f_value10, Real f_value11,
00054                          Real f_value12, Real f_value13, Real f_value14, Real f_value15);
00055       
00056       void SetRotationMatrix(const CRotationMatrix3& c_rotation);
00057       
00058       const CRotationMatrix3 GetRotationMatrix() const;
00059       
00060       void SetTranslationVector(const CVector3& c_translation);
00061       
00062       const CVector3 GetTranslationVector() const;
00063       
00064       CVector3 operator*(const CVector3& c_vector) const;
00065    };
00066 }
00067 
00068 #endif
```

---

Generated on 10 Jul 2018 for ARGoS by 
 1.6.1 
