# Supplementary material for: Iterative improvement in the automatic modular design of robot swarms
Source: PeerJ Comput Sci. 2020 Dec 7;6:e322. doi: 10.7717/peerj-cs.322 (PMC7924708; doi:10.7717/peerj-cs.322)
Supplement: Supplemental Information 3 [file peerj-cs-06-322-s003.zip › argos3/doc/api/standalone/a00382_source.html]

ARGoS: core/utility/math/plane.cpp Source File


- Main Page
- Related Pages
- Namespaces
- Classes
- Files

- File List
- File Members

# core/utility/math/plane.cpp

Go to the documentation of this file.

```
00001 #include "plane.h"
00002 #include "ray3.h"
00003 
00004 namespace argos {
00005 
00006    /****************************************/
00007    /****************************************/
00008 
00009    bool CPlane::Intersects(Real& f_t_on_ray,
00010                            const CRay3& c_ray) {
00011       /* Ray direction */
00012       CVector3 cRayDir;
00013       c_ray.GetDirection(cRayDir);
00014       /* Calculate f_t_on_ray */
00015       Real fNumerator = (m_cPosition-c_ray.GetStart()).DotProduct(m_cNormal);
00016       Real fDenominator = cRayDir.DotProduct(m_cNormal);
00017       /* Is ray parallel to plane? */
00018       if(Abs(fDenominator) > 1e-6) {
00019          /* No, it's not */
00020          f_t_on_ray = fNumerator / fDenominator / c_ray.GetLength();
00021          return (f_t_on_ray < 1.0f);
00022       }
00023       else {
00024          /* Yes, it is */
00025          /* Is ray coincident with the plane? */
00026          if(Abs(fNumerator) > 1e-6) {
00027             /* No, the ray is parallel to and far from the plane */
00028             /* No intersection possible */
00029             return false;
00030          }
00031          else {
00032             /* Yes, the ray coincides with the plane */
00033             f_t_on_ray = 0.0f;
00034             return true;
00035          }
00036       }
00037    }
00038 
00039    /****************************************/
00040    /****************************************/
00041 
00042 }
```

---

Generated on 10 Jul 2018 for ARGoS by 
 1.6.1 
