# Supplementary material for: Iterative improvement in the automatic modular design of robot swarms
Source: PeerJ Comput Sci. 2020 Dec 7;6:e322. doi: 10.7717/peerj-cs.322 (PMC7924708; doi:10.7717/peerj-cs.322)
Supplement: Supplemental Information 3 [file peerj-cs-06-322-s003.zip › argos3/doc/api/standalone/a00383_source.html]

ARGoS: core/utility/math/plane.h Source File


- Main Page
- Related Pages
- Namespaces
- Classes
- Files

- File List
- File Members

# core/utility/math/plane.h

Go to the documentation of this file.

```
00001 
00007 #ifndef PLANE_H
00008 #define PLANE_H
00009 
00010 namespace argos {
00011    class CPlane;
00012    class CRay3;
00013 }
00014 
00015 #include <argos3/core/utility/math/vector3.h>
00016 
00017 namespace argos {
00018 
00019    class CPlane {
00020 
00021    public:
00022 
00023       CPlane() :
00024          m_cNormal(CVector3::Z) {}
00025 
00026       CPlane(const CVector3& c_position,
00027              const CVector3& c_normal) :
00028          m_cPosition(c_position),
00029          m_cNormal(c_normal) {}
00030 
00031       ~CPlane() {}
00032 
00033       inline const CVector3& GetPosition() const {
00034          return m_cPosition;
00035       }
00036 
00037       inline void SetPosition(const CVector3& c_position) {
00038          m_cPosition = c_position;
00039       }
00040 
00041       inline const CVector3& GetNormal() const {
00042          return m_cNormal;
00043       }
00044 
00045       inline void SetNormal(const CVector3& c_normal) {
00046          m_cNormal = c_normal;
00047       }
00048 
00049       bool Intersects(Real& f_t_on_ray,
00050                       const CRay3& c_ray);
00051 
00052    private:
00053 
00054       CVector3 m_cPosition;
00055       CVector3 m_cNormal;
00056 
00057    };
00058 
00059 }
00060 
00061 #endif
```

---

Generated on 10 Jul 2018 for ARGoS by 
 1.6.1 
