# Supplementary material for: Iterative improvement in the automatic modular design of robot swarms
Source: PeerJ Comput Sci. 2020 Dec 7;6:e322. doi: 10.7717/peerj-cs.322 (PMC7924708; doi:10.7717/peerj-cs.322)
Supplement: Supplemental Information 3 [file peerj-cs-06-322-s003.zip › argos3/doc/api/standalone/a00384_source.html]

ARGoS: core/utility/math/quaternion.h Source File


- Main Page
- Related Pages
- Namespaces
- Classes
- Files

- File List
- File Members

# core/utility/math/quaternion.h

Go to the documentation of this file.

```
00001 
00007 #ifndef CQUATERNION_H
00008 #define CQUATERNION_H
00009 
00010 #include <argos3/core/utility/math/vector3.h>
00011 
00012 namespace argos {
00013 
00014    class CQuaternion {
00015 
00016    public:
00017       CQuaternion() {
00018          m_fValues[0] = 1.0;
00019          m_fValues[1] = 0.0;
00020          m_fValues[2] = 0.0;
00021          m_fValues[3] = 0.0;
00022       }
00023 
00024       CQuaternion(const CQuaternion& c_quaternion) {
00025          *this = c_quaternion;
00026       }
00027 
00028       CQuaternion(Real f_real,
00029                   Real f_img1,
00030                   Real f_img2,
00031                   Real f_img3) {
00032          m_fValues[0] = f_real;
00033          m_fValues[1] = f_img1;
00034          m_fValues[2] = f_img2;
00035          m_fValues[3] = f_img3;
00036       }
00037 
00038       CQuaternion(const CRadians& c_radians,
00039                   const CVector3& c_vector3) {
00040          FromAngleAxis(c_radians, c_vector3);
00041       }
00042 
00043       inline CQuaternion(const CVector3& c_vector1,
00044                          const CVector3& c_vector2) {
00045          BetweenTwoVectors(c_vector1, c_vector2);
00046       }
00047 
00048       inline Real GetW() const {
00049          return m_fValues[0];
00050       }
00051 
00052       inline Real GetX() const {
00053          return m_fValues[1];
00054       }
00055 
00056       inline Real GetY() const {
00057          return m_fValues[2];
00058       }
00059 
00060       inline Real GetZ() const {
00061          return m_fValues[3];
00062       }
00063 
00064       inline void SetW(Real f_w) {
00065          m_fValues[0] = f_w;
00066       }
00067 
00068       inline void SetX(Real f_x) {
00069          m_fValues[1] = f_x;
00070       }
00071 
00072       inline void SetY(Real f_y) {
00073          m_fValues[2] = f_y;
00074       }
00075 
00076       inline void SetZ(Real f_z) {
00077          m_fValues[3] = f_z;
00078       }
00079 
00080       inline void Set(Real f_w,
00081                       Real f_x,
00082                       Real f_y,
00083                       Real f_z) {
00084          m_fValues[0] = f_w;
00085          m_fValues[1] = f_x;
00086          m_fValues[2] = f_y;
00087          m_fValues[3] = f_z;
00088       }
00089 
00090       inline CQuaternion Conjugate() const {
00091          return CQuaternion(m_fValues[0],
00092                             -m_fValues[1],
00093                             -m_fValues[2],
00094                             -m_fValues[3]);
00095       }
00096 
00097       inline CQuaternion Inverse() const {
00098          return CQuaternion(m_fValues[0],
00099                             -m_fValues[1],
00100                             -m_fValues[2],
00101                             -m_fValues[3]);
00102       }
00103 
00104       inline Real Length() const {
00105          return ::sqrt(SquareLength());
00106       }
00107 
00108       inline Real SquareLength() const {
00109          return
00110             Square(m_fValues[0]) +
00111             Square(m_fValues[1]) +
00112             Square(m_fValues[2]) +
00113             Square(m_fValues[3]);
00114       }
00115 
00116       inline CQuaternion& Normalize() {
00117          Real fInvLength = 1.0 / Length();
00118          m_fValues[0] *= fInvLength;
00119          m_fValues[1] *= fInvLength;
00120          m_fValues[2] *= fInvLength;
00121          m_fValues[3] *= fInvLength;
00122          return *this;
00123       }
00124 
00125       inline CQuaternion& FromAngleAxis(const CRadians& c_angle,
00126                                         const CVector3& c_vector) {
00127          CRadians cHalfAngle = c_angle * 0.5;
00128          Real fSin, fCos;
00129 #ifdef ARGOS_SINCOS
00130          SinCos(cHalfAngle, fSin, fCos);
00131 #else
00132          fSin = Sin(cHalfAngle);
00133          fCos = Cos(cHalfAngle);
00134 #endif
00135          m_fValues[0] = fCos;
00136          m_fValues[1] = c_vector.GetX() * fSin;
00137          m_fValues[2] = c_vector.GetY() * fSin;
00138          m_fValues[3] = c_vector.GetZ() * fSin;
00139          return *this;
00140       }
00141 
00142       inline void ToAngleAxis(CRadians& c_angle,
00143                               CVector3& c_vector) const {
00144          Real fSquareLength =
00145             Square(m_fValues[1]) +
00146             Square(m_fValues[2]) +
00147             Square(m_fValues[3]);
00148          if(fSquareLength > 0.0f) {
00149             c_angle = 2.0f * ACos(m_fValues[0]);
00150             Real fInvLength = 1.0f / ::sqrt(fSquareLength);
00151             c_vector.Set(m_fValues[1] * fInvLength,
00152                          m_fValues[2] * fInvLength,
00153                          m_fValues[3] * fInvLength);
00154          }
00155          else {
00156             /* By default, to ease the support of robot orientation, no rotation refers to the Z axis */
00157             c_angle = CRadians::ZERO;
00158             c_vector = CVector3::Z;
00159          }
00160       }
00161 
00162       inline CQuaternion& FromEulerAngles(const CRadians& c_z_angle,
00163                                           const CRadians& c_y_angle,
00164                                           const CRadians& c_x_angle) {
00165          (*this) = CQuaternion(c_x_angle, CVector3::X) *
00166             CQuaternion(c_y_angle, CVector3::Y) *
00167             CQuaternion(c_z_angle, CVector3::Z);
00168          return (*this);
00169       }
00170 
00171       inline void ToEulerAngles(CRadians& c_z_angle,
00172                                 CRadians& c_y_angle,
00173                                 CRadians& c_x_angle) const {
00174          /* With the ZYX convention, gimbal lock happens when
00175             cos(y_angle) = 0, that is when y_angle = +- pi/2
00176             In this condition, the Z and X axis overlap and we
00177             lose one degree of freedom. It's a problem of the
00178             Euler representation of rotations that is not
00179             present when we deal with quaternions.
00180             For reasons of speed, we consider gimbal lock
00181             happened when fTest > 0.499 and when fTest < -0.499.
00182          */
00183          /* Computed to understand if we have gimbal lock or not */
00184          Real fTest =
00185             m_fValues[1] * m_fValues[3] +
00186             m_fValues[0] * m_fValues[2];
00187 
00188          if(fTest > 0.499f) {
00189             /* Gimbal lock */
00190             c_x_angle = CRadians::ZERO;
00191             c_y_angle = CRadians::PI_OVER_TWO;
00192             c_z_angle = ATan2(2.0f * (m_fValues[1] * m_fValues[2] + m_fValues[0] * m_fValues[3]),
00193                               1.0f - 2.0f * (m_fValues[1] * m_fValues[1] + m_fValues[3] * m_fValues[3]));
00194          }
00195          else if(fTest < -0.499f) {
00196             /* Gimbal lock */
00197             c_x_angle = CRadians::ZERO;
00198             c_y_angle = -CRadians::PI_OVER_TWO;
00199             c_z_angle = ATan2(2.0f * (m_fValues[1] * m_fValues[2] + m_fValues[0] * m_fValues[3]),
00200                               1.0f - 2.0f * (m_fValues[1] * m_fValues[1] + m_fValues[3] * m_fValues[3]));
00201          }
00202          else {
00203             /* Normal case */
00204             Real fSqValues[4] = {
00205                Square(m_fValues[0]),
00206                Square(m_fValues[1]),
00207                Square(m_fValues[2]),
00208                Square(m_fValues[3])
00209             };
00210             
00211             c_x_angle = ATan2(2.0 * (m_fValues[0] * m_fValues[1] - m_fValues[2] * m_fValues[3]),
00212                               fSqValues[0] - fSqValues[1] - fSqValues[2] + fSqValues[3]);
00213             c_y_angle = ASin(2.0 * (m_fValues[1] * m_fValues[3] + m_fValues[0] * m_fValues[2]));
00214             c_z_angle = ATan2(2.0 * (m_fValues[0] * m_fValues[3] - m_fValues[1] * m_fValues[2]),
00215                               fSqValues[0] + fSqValues[1] - fSqValues[2] - fSqValues[3]);
00216          }
00217       }
00218 
00219       inline CQuaternion& BetweenTwoVectors(const CVector3& c_vector1,
00220                                             const CVector3& c_vector2) {
00221          Real fProd =
00222             c_vector1.DotProduct(c_vector2) /
00223             Sqrt(c_vector1.SquareLength() * c_vector2.SquareLength());
00224          if(fProd > 0.999999f) {
00225             /* The two vectors are parallel, no rotation */
00226             m_fValues[0] = 1.0;
00227             m_fValues[1] = 0.0;
00228             m_fValues[2] = 0.0;
00229             m_fValues[3] = 0.0;
00230          }
00231          else if(fProd < -0.999999f) {
00232             /* The two vectors are anti-parallel */
00233             /* We need to set an arbitrary rotation axis */
00234             /* To find it, we calculate the cross product of c_vector1 with either X or Y,
00235                depending on which is not coplanar with c_vector1 */
00236             CVector3 cRotAxis = c_vector1;
00237             if(Abs(c_vector1.DotProduct(CVector3::X)) < 0.999999) {
00238                /* Use the X axis */
00239                cRotAxis.CrossProduct(CVector3::X);
00240             }
00241             else {
00242                /* Use the Y axis */
00243                cRotAxis.CrossProduct(CVector3::Y);
00244             }
00245             /* The wanted quaternion is a rotation around cRotAxis by 180 degrees */
00246             FromAngleAxis(CRadians::PI, cRotAxis);
00247          }
00248          else {
00249             /* The two vectors are not parallel nor anti-parallel */
00250             m_fValues[0] = Sqrt(c_vector1.SquareLength() * c_vector2.SquareLength()) + fProd;
00251             CVector3 cCrossProd(c_vector1);
00252             cCrossProd.CrossProduct(c_vector2);
00253             m_fValues[1] = cCrossProd.GetX();
00254             m_fValues[2] = cCrossProd.GetY();
00255             m_fValues[3] = cCrossProd.GetZ();
00256             Normalize();
00257          }
00258          return *this;
00259       }
00260 
00261       inline bool operator==(const CQuaternion& c_quaternion) {
00262          return (m_fValues[0] == c_quaternion.m_fValues[0] &&
00263                  m_fValues[1] == c_quaternion.m_fValues[1] &&
00264                  m_fValues[2] == c_quaternion.m_fValues[2] &&
00265                  m_fValues[3] == c_quaternion.m_fValues[3]);
00266       }      
00267 
00268       inline CQuaternion& operator=(const CQuaternion& c_quaternion) {
00269          if (&c_quaternion != this) {
00270             m_fValues[0] = c_quaternion.m_fValues[0];
00271             m_fValues[1] = c_quaternion.m_fValues[1];
00272             m_fValues[2] = c_quaternion.m_fValues[2];
00273             m_fValues[3] = c_quaternion.m_fValues[3];
00274          }
00275          return *this;
00276       }
00277 
00278       inline CQuaternion& operator+=(const CQuaternion& c_quaternion) {
00279          m_fValues[0] += c_quaternion.m_fValues[0];
00280          m_fValues[1] += c_quaternion.m_fValues[1];
00281          m_fValues[2] += c_quaternion.m_fValues[2];
00282          m_fValues[3] += c_quaternion.m_fValues[3];
00283          return *this;
00284       }
00285 
00286       inline CQuaternion& operator-=(const CQuaternion& c_quaternion) {
00287          m_fValues[0] -= c_quaternion.m_fValues[0];
00288          m_fValues[1] -= c_quaternion.m_fValues[1];
00289          m_fValues[2] -= c_quaternion.m_fValues[2];
00290          m_fValues[3] -= c_quaternion.m_fValues[3];
00291          return *this;
00292       }
00293 
00294       inline CQuaternion& operator*=(const CQuaternion& c_quaternion) {
00295          Real newv[4];
00296          newv[0] = m_fValues[0] * c_quaternion.m_fValues[0] -
00297             m_fValues[1] * c_quaternion.m_fValues[1] -
00298             m_fValues[2] * c_quaternion.m_fValues[2] -
00299             m_fValues[3] * c_quaternion.m_fValues[3];
00300          newv[1] = m_fValues[0] * c_quaternion.m_fValues[1] +
00301             m_fValues[1] * c_quaternion.m_fValues[0] +
00302             m_fValues[2] * c_quaternion.m_fValues[3] -
00303             m_fValues[3] * c_quaternion.m_fValues[2];
00304          newv[2] = m_fValues[0] * c_quaternion.m_fValues[2] -
00305             m_fValues[1] * c_quaternion.m_fValues[3] +
00306             m_fValues[2] * c_quaternion.m_fValues[0] +
00307             m_fValues[3] * c_quaternion.m_fValues[1];
00308          newv[3] = m_fValues[0] * c_quaternion.m_fValues[3] +
00309             m_fValues[1] * c_quaternion.m_fValues[2] -
00310             m_fValues[2] * c_quaternion.m_fValues[1] +
00311             m_fValues[3] * c_quaternion.m_fValues[0];
00312          m_fValues[0] = newv[0];
00313          m_fValues[1] = newv[1];
00314          m_fValues[2] = newv[2];
00315          m_fValues[3] = newv[3];
00316          return *this;
00317       }
00318 
00319       inline CQuaternion operator+(const CQuaternion& c_quaternion) const {
00320          CQuaternion result(*this);
00321          result += c_quaternion;
00322          return result;
00323       }
00324 
00325       inline CQuaternion operator-(const CQuaternion& c_quaternion) const {
00326          CQuaternion result(*this);
00327          result -= c_quaternion;
00328          return result;
00329       }
00330 
00331       inline CQuaternion operator*(const CQuaternion& c_quaternion) const {
00332          CQuaternion result(*this);
00333          result *= c_quaternion;
00334          return result;
00335       }
00336 
00344       inline friend std::ostream& operator<<(std::ostream& c_os, const CQuaternion& c_quaternion) {
00345          CRadians cZAngle, cYAngle, cXAngle;
00346          c_quaternion.ToEulerAngles(cZAngle, cYAngle, cXAngle);        
00347          c_os << ToDegrees(cZAngle).GetValue() << ","
00348               << ToDegrees(cYAngle).GetValue() << ","
00349               << ToDegrees(cXAngle).GetValue();
00350          return c_os;
00351       }
00352       
00360       inline friend std::istream& operator>>(std::istream& c_is, CQuaternion& c_quaternion) {
00361          Real fValues[3];
00362          ParseValues<Real>(c_is, 3, fValues, ',');
00363          c_quaternion.FromEulerAngles(ToRadians(CDegrees(fValues[0])),
00364                                       ToRadians(CDegrees(fValues[1])), 
00365                                       ToRadians(CDegrees(fValues[2])));
00366          return c_is;
00367       }
00368 
00369    private:
00370 
00371       Real m_fValues[4];
00372    };
00373 
00374 }
00375 
00376 #endif
```

---

Generated on 10 Jul 2018 for ARGoS by 
 1.6.1 
