# Supplementary material for: Iterative improvement in the automatic modular design of robot swarms
Source: PeerJ Comput Sci. 2020 Dec 7;6:e322. doi: 10.7717/peerj-cs.322 (PMC7924708; doi:10.7717/peerj-cs.322)
Supplement: Supplemental Information 3 [file peerj-cs-06-322-s003.zip › argos3/doc/api/standalone/a00385_source.html]

ARGoS: core/utility/math/range.h Source File


- Main Page
- Related Pages
- Namespaces
- Classes
- Files

- File List
- File Members

# core/utility/math/range.h

Go to the documentation of this file.

```
00001 
00007 #ifndef CRANGE_H
00008 #define CRANGE_H
00009 
00010 #include <argos3/core/utility/datatypes/datatypes.h>
00011 #include <argos3/core/utility/string_utilities.h>
00012 #include <argos3/core/utility/configuration/argos_exception.h>
00013 #include <iostream>
00014 
00015 namespace argos {
00016 
00017    template<typename T> class CRange {
00018 
00019    public:
00020 
00021       CRange(const T& t_min = T(),
00022              const T& t_max = T()) :
00023          m_tMin(t_min),
00024          m_tMax(t_max),
00025          m_tSpan(m_tMax - m_tMin) {
00026          ARGOS_ASSERT(t_min <= t_max,
00027                       "Error initializing CRange(" <<
00028                       t_min << ", " << t_max << "): " <<
00029                       t_min << " is not <= " << t_max);
00030       }
00031 
00032       inline T GetMin() const {
00033          return m_tMin;
00034       }
00035 
00036       inline void SetMin(const T& t_min) {
00037          ARGOS_ASSERT(t_min <= m_tMax,
00038                       "Error setting min CRange bound (" <<
00039                       t_min << "): " <<
00040                       t_min << " is not <= " << m_tMax);
00041          m_tMin = t_min;
00042          /* Same as, but faster than
00043             m_tSpan = m_tMax - m_tMin; */
00044          m_tSpan = m_tMax;
00045          m_tSpan -= m_tMin;
00046       }
00047 
00048       inline T GetMax() const {
00049          return m_tMax;
00050       }
00051 
00052       inline void SetMax(const T& t_max) {
00053          ARGOS_ASSERT(m_tMin <= t_max,
00054                       "Error setting max CRange bound (" <<
00055                       t_max << "): " <<
00056                       m_tMin << " is not <= " << t_max);
00057          m_tMax = t_max;
00058          /* Same as, but faster than
00059             m_tSpan = m_tMax - m_tMin; */
00060          m_tSpan = m_tMax;
00061          m_tSpan -= m_tMin;
00062       }
00063 
00064       inline T GetSpan() const {
00065          return m_tSpan;
00066       }
00067 
00068       inline void Set(const T& t_min, const T& t_max) {
00069          ARGOS_ASSERT(t_min <= t_max,
00070                       "Error setting CRange bounds (" <<
00071                       t_min << ", " << t_max << "): " <<
00072                       t_min << " is not <= " << t_max);
00073          m_tMin = t_min;
00074          m_tMax = t_max;
00075          /* Same as, but faster than
00076             m_tSpan = m_tMax - m_tMin; */
00077          m_tSpan = m_tMax;
00078          m_tSpan -= m_tMin;
00079       }
00080 
00081       inline bool WithinMinBoundIncludedMaxBoundIncluded(const T& t_value) const {
00082          return t_value >= m_tMin && t_value <= m_tMax;
00083       }
00084 
00085       inline bool WithinMinBoundIncludedMaxBoundExcluded(const T& t_value) const {
00086          return t_value >= m_tMin && t_value < m_tMax;
00087       }
00088 
00089       inline bool WithinMinBoundExcludedMaxBoundIncluded(const T& t_value) const {
00090          return t_value > m_tMin && t_value <= m_tMax;
00091       }
00092 
00093       inline bool WithinMinBoundExcludedMaxBoundExcluded(const T& t_value) const {
00094          return t_value > m_tMin && t_value < m_tMax;
00095       }
00096 
00097       inline void TruncValue(T& t_value) const {
00098          if (t_value > m_tMax) t_value = m_tMax;
00099          if (t_value < m_tMin) t_value = m_tMin;
00100       }
00101 
00102       inline Real NormalizeValue(const T& t_value) const {
00103          T tTmpValue(t_value);
00104          TruncValue(tTmpValue);
00105          return static_cast<Real>(tTmpValue - m_tMin) /
00106                 static_cast<Real>(m_tSpan);
00107       }
00108 
00109       template <typename U> void MapValueIntoRange(U& t_output_value,
00110                                                    const T& t_input_value,
00111                                                    const CRange<U>& c_range) const {
00112          t_output_value = (NormalizeValue(t_input_value) *
00113                            c_range.GetSpan()) + c_range.GetMin();
00114       }
00115 
00116       inline void WrapValue(T& t_value) const {
00117          while(t_value > m_tMax) t_value -= m_tSpan;
00118          while(t_value < m_tMin) t_value += m_tSpan;
00119       }
00120 
00121       inline friend std::ostream& operator<<(std::ostream& os,
00122                                              const CRange& c_range) {
00123          os << c_range.m_tMin << ":"
00124             << c_range.m_tMax;
00125          return os;
00126       }
00127 
00128       inline friend std::istream& operator>>(std::istream& is,
00129                                              CRange& c_range) {
00130          T tValues[2];
00131          ParseValues<T> (is, 2, tValues, ':');
00132          c_range.Set(tValues[0], tValues[1]);
00133          return is;
00134       }
00135 
00136    private:
00137 
00138       T m_tMin;
00139       T m_tMax;
00140       T m_tSpan;
00141 
00142    };
00143 
00144 }
00145 
00146 #endif
```

---

Generated on 10 Jul 2018 for ARGoS by 
 1.6.1 
