# Supplementary material for: Iterative improvement in the automatic modular design of robot swarms
Source: PeerJ Comput Sci. 2020 Dec 7;6:e322. doi: 10.7717/peerj-cs.322 (PMC7924708; doi:10.7717/peerj-cs.322)
Supplement: Supplemental Information 3 [file peerj-cs-06-322-s003.zip › argos3/doc/api/standalone/a00386_source.html]

ARGoS: core/utility/math/ray2.h Source File


- Main Page
- Related Pages
- Namespaces
- Classes
- Files

- File List
- File Members

# core/utility/math/ray2.h

Go to the documentation of this file.

```
00001 
00007 #ifndef RAY2_H
00008 #define RAY2_H
00009 
00010 namespace argos {
00011    class CRay2;
00012 }
00013 
00014 #include <argos3/core/utility/math/vector2.h>
00015 
00016 namespace argos {
00017 
00018    class CRay2 {
00019 
00020    public:
00021 
00022       CRay2() {
00023       }
00024 
00025       CRay2(const CVector2& c_start,
00026             const CVector2& c_end) :
00027          m_cStart(c_start), m_cEnd(c_end) {
00028       }
00029 
00030       CRay2(const CVector2& c_start,
00031             const CVector2& c_direction,
00032             Real f_length) {
00033          Set(c_start, c_direction, f_length);
00034       }
00035 
00036       inline const CVector2& GetStart() const {
00037          return m_cStart;
00038       }
00039 
00040       inline const CVector2& GetEnd() const {
00041          return m_cEnd;
00042       }
00043 
00044       inline void SetStart(const CVector2& c_start) {
00045          m_cStart = c_start;
00046       }
00047 
00048       inline void SetEnd(const CVector2& c_end) {
00049          m_cEnd = c_end;
00050       }
00051 
00052       inline void Set(const CVector2& c_start, const CVector2& c_end) {
00053          m_cStart = c_start;
00054          m_cEnd = c_end;
00055       }
00056 
00057       inline void Set(const CVector2& c_start, const CVector2& c_direction, Real f_length) {
00058          m_cStart = c_start;
00059          /* Same as, but faster than
00060             m_cEnd = m_cStart + f_length * c_direction; */
00061          m_cEnd = m_cStart;
00062          m_cEnd += f_length * c_direction;
00063       }
00064 
00065       inline void GetDirection(CVector2& c_buffer) const {
00066          /* Same as, but faster than
00067             c_buffer = (m_cEnd - m_cStart).Normalize(); */
00068          c_buffer = m_cEnd;
00069          c_buffer -= m_cStart;
00070          c_buffer.Normalize();
00071       }
00072 
00073       inline void GetInverseDirection(CVector2& c_buffer) const {
00074          /* Same as, but faster than
00075             c_buffer = (m_cEnd - m_cStart).Normalize(); */
00076          c_buffer = m_cStart;
00077          c_buffer -= m_cEnd;
00078          c_buffer.Normalize();
00079       }
00080 
00081       inline Real GetLength() const {
00082          return (m_cEnd - m_cStart).Length();
00083       }
00084 
00085       inline void ToVector(CVector2& c_buffer) const {
00086          /* Same as, but faster than
00087             c_buffer = m_cEnd - m_cStart; */
00088          c_buffer = m_cEnd;
00089          c_buffer -= m_cStart;
00090       }
00091 
00092       /* Returns the point on the line corresponding to f_t */
00093       inline void GetPoint(CVector2& c_point,
00094                            Real f_t) const {
00095          c_point.SetX(m_cStart.GetX() + f_t * (m_cEnd.GetX() - m_cStart.GetX()));
00096          c_point.SetY(m_cStart.GetY() + f_t * (m_cEnd.GetY() - m_cStart.GetY()));
00097       }
00098 
00099       /* Returns the distance from the ray2 start to the point on the line corresponding to f_t */
00100       inline Real GetDistance(Real f_t) const {
00101          return ::sqrt(Square(f_t * (m_cEnd.GetX() - m_cStart.GetX())) +
00102                        Square(f_t * (m_cEnd.GetY() - m_cStart.GetY())));
00103       }
00104 
00105       /* Returns <tt>true</tt> if the passed ray intersects the current one. */
00106       inline bool Intersects(const CRay2& c_ray) const {
00107          Real fDiscriminant =
00108             (c_ray.m_cEnd.GetY() - c_ray.m_cStart.GetY()) *
00109             (m_cEnd.GetX() - m_cStart.GetX()) -
00110             (c_ray.m_cEnd.GetX() - c_ray.m_cStart.GetX()) *
00111             (m_cEnd.GetY() - m_cStart.GetY());
00112          if(Abs(fDiscriminant) < 1e-4) {
00113             /* The rays are parallel */
00114             return false;
00115          }
00116          /* If we get here, we know the rays are not parallel */
00117          /* Calculate value of T on ray 1 for the intersection point */
00118          Real fT1 =
00119             (m_cStart.GetX() - c_ray.m_cStart.GetX()) *
00120             (c_ray.m_cEnd.GetY() - c_ray.m_cStart.GetY()) -
00121             (m_cStart.GetY() - c_ray.m_cStart.GetY()) *
00122             (c_ray.m_cEnd.GetX() - c_ray.m_cStart.GetX());
00123          /* If T on ray 1 is outside the ray, no intersection */
00124          if(fT1 < 0.0 || fT1 > 1.0) return false;
00125          /* Calculate value of T on ray 2 for the intersection point */
00126          Real fT2;
00127          if(Abs(c_ray.m_cEnd.GetY() - c_ray.m_cStart.GetY()) > 1e-4) {
00128             /* Ray 2 is not vertical */
00129             fT2 =
00130                (m_cStart.GetY() - c_ray.m_cStart.GetY()) +
00131                (m_cEnd.GetY() - m_cStart.GetY()) *
00132                fT1 /
00133                (c_ray.m_cEnd.GetY() - c_ray.m_cStart.GetY());
00134          }
00135          else {
00136             /* Ray 2 is vertical */
00137             fT2 =
00138                (m_cStart.GetX() - c_ray.m_cStart.GetX()) +
00139                (m_cEnd.GetX() - m_cStart.GetX()) *
00140                fT1 /
00141                (c_ray.m_cEnd.GetX() - c_ray.m_cStart.GetX());
00142          }
00143          /* If T on ray 2 is inside the ray, intersection */
00144          return(fT2 >= 0.0 && fT2 <= 1.0);
00145       }
00146 
00153       inline friend std::ostream& operator<<(std::ostream& c_os,
00154                                              const CRay2& c_ray) {
00155          c_os << c_ray.GetStart() << " -> " << c_ray.GetEnd();
00156          return c_os;
00157       }
00158 
00159    private:
00160 
00161       CVector2 m_cStart;
00162       CVector2 m_cEnd;
00163 
00164    };
00165 
00166 }
00167 
00168 #endif
```

---

Generated on 10 Jul 2018 for ARGoS by 
 1.6.1 
