# Supplementary material for: Iterative improvement in the automatic modular design of robot swarms
Source: PeerJ Comput Sci. 2020 Dec 7;6:e322. doi: 10.7717/peerj-cs.322 (PMC7924708; doi:10.7717/peerj-cs.322)
Supplement: Supplemental Information 3 [file peerj-cs-06-322-s003.zip › argos3/doc/api/standalone/a00387_source.html]

ARGoS: core/utility/math/ray3.cpp Source File


- Main Page
- Related Pages
- Namespaces
- Classes
- Files

- File List
- File Members

# core/utility/math/ray3.cpp

Go to the documentation of this file.

```
00001 
00007 #include "ray3.h"
00008 #include "plane.h"
00009 
00010 namespace argos {
00011 
00012    /****************************************/
00013    /****************************************/
00014 
00015    bool CRay3::Intersects(const CPlane& c_plane,
00016                           CVector3& c_point) const {
00017       CVector3 cRayVec;
00018       Real fDen = c_plane.GetNormal().DotProduct(ToVector(cRayVec));
00019       if(Abs(fDen) > 0.0f) {
00020          /* There could be intersection */
00021          Real fTOnRay = -c_plane.GetNormal().DotProduct(m_cStart - c_plane.GetPosition()) / fDen;
00022          if(fTOnRay < 0.0f || fTOnRay > 1.0f) {
00023             /* Intersection point is beyond ray extrema */
00024             return false;
00025          }
00026          else {
00027             /* There is an intersection */
00028             GetPoint(c_point, fTOnRay);
00029             return true;
00030          }
00031       }
00032       else {
00033          /* No intersection */
00034          return false;
00035       }
00036    }
00037    
00038    /****************************************/
00039    /****************************************/
00040 
00041 }
```

---

Generated on 10 Jul 2018 for ARGoS by 
 1.6.1 
