# Supplementary material for: Iterative improvement in the automatic modular design of robot swarms
Source: PeerJ Comput Sci. 2020 Dec 7;6:e322. doi: 10.7717/peerj-cs.322 (PMC7924708; doi:10.7717/peerj-cs.322)
Supplement: Supplemental Information 3 [file peerj-cs-06-322-s003.zip › argos3/doc/api/standalone/a00388_source.html]

ARGoS: core/utility/math/ray3.h Source File


- Main Page
- Related Pages
- Namespaces
- Classes
- Files

- File List
- File Members

# core/utility/math/ray3.h

Go to the documentation of this file.

```
00001 
00007 #ifndef RAY3_H
00008 #define RAY3_H
00009 
00010 namespace argos {
00011    class CRay3;
00012    class CPlane;
00013 }
00014 
00015 #include <argos3/core/utility/math/vector3.h>
00016 
00017 namespace argos {
00018 
00019    class CRay3 {
00020 
00021    public:
00022 
00023       CRay3() {
00024       }
00025 
00026       CRay3(const CVector3& c_start,
00027             const CVector3& c_end) :
00028          m_cStart(c_start), m_cEnd(c_end) {
00029       }
00030 
00031       CRay3(const CVector3& c_start,
00032             const CVector3& c_direction,
00033             Real f_length) {
00034          Set(c_start, c_direction, f_length);
00035       }
00036 
00037       inline CVector3& GetStart() {
00038          return m_cStart;
00039       }
00040 
00041       inline const CVector3& GetStart() const {
00042          return m_cStart;
00043       }
00044 
00045       inline CVector3& GetEnd() {
00046          return m_cEnd;
00047       }
00048 
00049       inline const CVector3& GetEnd() const {
00050          return m_cEnd;
00051       }
00052 
00053       inline void SetStart(const CVector3& c_start) {
00054          m_cStart = c_start;
00055       }
00056 
00057       inline void SetEnd(const CVector3& c_end) {
00058          m_cEnd = c_end;
00059       }
00060 
00061       inline void SetLength(Real f_length) {
00062           CVector3 c_direction;
00063           GetDirection(c_direction);
00064           Set(m_cStart, c_direction, f_length);
00065       }
00066 
00067       inline void Set(const CVector3& c_start, const CVector3& c_end) {
00068          m_cStart = c_start;
00069          m_cEnd = c_end;
00070       }
00071 
00072       inline void Set(const CVector3& c_start, const CVector3& c_direction, Real f_length) {
00073          m_cStart = c_start;
00074          /* Same as, but faster than
00075             m_cEnd = m_cStart + f_length * c_direction; */
00076          m_cEnd = m_cStart;
00077          m_cEnd += f_length * c_direction;
00078       }
00079 
00080       inline void GetDirection(CVector3& c_buffer) const {
00081          /* Same as, but faster than
00082             c_buffer = (m_cEnd - m_cStart).Normalize(); */
00083          c_buffer = m_cEnd;
00084          c_buffer -= m_cStart;
00085          c_buffer.Normalize();
00086       }
00087 
00088       inline void GetInverseDirection(CVector3& c_buffer) const {
00089          /* Same as, but faster than
00090             c_buffer = (m_cEnd - m_cStart).Normalize(); */
00091          c_buffer = m_cStart;
00092          c_buffer -= m_cEnd;
00093          c_buffer.Normalize();
00094       }
00095 
00096       inline Real GetLength() const {
00097          return (m_cEnd - m_cStart).Length();
00098       }
00099 
00100       inline CVector3& ToVector(CVector3& c_buffer) const {
00101          /* Same as, but faster than
00102             c_buffer = m_cEnd - m_cStart; */
00103          c_buffer = m_cEnd;
00104          c_buffer -= m_cStart;
00105          return c_buffer;
00106       }
00107 
00108       /* Returns the point on the line corresponding to f_t */
00109       inline void GetPoint(CVector3& c_point,
00110                            Real f_t) const {
00111          c_point.SetX(m_cStart.GetX() + f_t * (m_cEnd.GetX() - m_cStart.GetX()));
00112          c_point.SetY(m_cStart.GetY() + f_t * (m_cEnd.GetY() - m_cStart.GetY()));
00113          c_point.SetZ(m_cStart.GetZ() + f_t * (m_cEnd.GetZ() - m_cStart.GetZ()));
00114       }
00115 
00116       /* Returns the distance from the ray3 start to the point on the line corresponding to f_t */
00117       inline Real GetDistance(Real f_t) const {
00118          return ::sqrt(Square(f_t * (m_cEnd.GetX() - m_cStart.GetX())) +
00119                        Square(f_t * (m_cEnd.GetY() - m_cStart.GetY())) +
00120                        Square(f_t * (m_cEnd.GetZ() - m_cStart.GetZ())));
00121       }
00122 
00123       /*
00124        * Calculates the ray-plane intersection point.
00125        * @param c_plane The plane whose intersection with this ray must be calculated
00126        * @param c_point The resulting intersection point is returned here
00127        * @return true if intersection occurred, false if ray and plane are parallel or ray lies on plane
00128        */
00129       bool Intersects(const CPlane& c_plane,
00130                       CVector3& c_point) const;
00131 
00138       inline friend std::ostream& operator<<(std::ostream& c_os,
00139                                              const CRay3& c_ray) {
00140          c_os << c_ray.GetStart() << " -> " << c_ray.GetEnd();
00141          return c_os;
00142       }
00143 
00144    private:
00145 
00146       CVector3 m_cStart;
00147       CVector3 m_cEnd;
00148 
00149    };
00150 
00151 }
00152 
00153 #endif
```

---

Generated on 10 Jul 2018 for ARGoS by 
 1.6.1 
