# Supplementary material for: Iterative improvement in the automatic modular design of robot swarms
Source: PeerJ Comput Sci. 2020 Dec 7;6:e322. doi: 10.7717/peerj-cs.322 (PMC7924708; doi:10.7717/peerj-cs.322)
Supplement: Supplemental Information 3 [file peerj-cs-06-322-s003.zip › argos3/doc/api/standalone/a00389.html]

ARGoS: core/utility/math/rng.cpp File Reference


- Main Page
- Related Pages
- Namespaces
- Classes
- Files

- File List
- File Members

# core/utility/math/rng.cpp File Reference

`#include "rng.h"`  
`#include <argos3/core/utility/configuration/argos_exception.h>`  
`#include <argos3/core/utility/logging/argos_log.h>`  
`#include <cstring>`  
`#include <limits>`  
`#include <cmath>`  

Include dependency graph for rng.cpp:

Go to the source code of this file.

|  |  |
| --- | --- |
| Namespaces | |
| namespace | argos |

|  |  |
| --- | --- |
|  | The namespace containing all the ARGoS related code. |

| Defines | |
| #define | CHECK\_CATEGORY(category) |

---

## Define Documentation

|  |  |  |  |  |  |
| --- | --- | --- | --- | --- | --- |
| #define CHECK\_CATEGORY | ( | category |  | ) |  |

**Value:**

```
std::map<std::string, CCategory*>::iterator itCategory = m_mapCategories.find(category); \
   if(itCategory == m_mapCategories.end()) {                            \
      THROW_ARGOSEXCEPTION("CRandom:: can't find category \"" << category << "\"."); \
   }
```

Definition at line 32 of file rng.cpp.

---

Generated on 10 Jul 2018 for ARGoS by 
 1.6.1 
