# Supplementary material for: Iterative improvement in the automatic modular design of robot swarms
Source: PeerJ Comput Sci. 2020 Dec 7;6:e322. doi: 10.7717/peerj-cs.322 (PMC7924708; doi:10.7717/peerj-cs.322)
Supplement: Supplemental Information 3 [file peerj-cs-06-322-s003.zip › argos3/doc/api/standalone/a00389_source.html]

ARGoS: core/utility/math/rng.cpp Source File


- Main Page
- Related Pages
- Namespaces
- Classes
- Files

- File List
- File Members

# core/utility/math/rng.cpp

Go to the documentation of this file.

```
00001 
00009 #include "rng.h"
00010 #include <argos3/core/utility/configuration/argos_exception.h>
00011 #include <argos3/core/utility/logging/argos_log.h>
00012 #include <cstring>
00013 #include <limits>
00014 #include <cmath>
00015 
00016 namespace argos {
00017 
00018    /****************************************/
00019    /****************************************/
00020 
00021    /* Period parameters */
00022    static const SInt32 N = 624;
00023    static const SInt32 M = 397;
00024    static const UInt32 MATRIX_A = 0x9908b0dfUL;   /* constant vector a */
00025    static const UInt32 UPPER_MASK = 0x80000000UL; /* most significant w-r bits */
00026    static const UInt32 LOWER_MASK = 0x7fffffffUL; /* least significant r bits */
00027    static const CRange<UInt32> INT_RANGE = CRange<UInt32>(0, 0xFFFFFFFFUL);
00028 
00029    std::map<std::string, CRandom::CCategory*> CRandom::m_mapCategories;
00030 
00031    /* Checks that a category exists. It internally creates an iterator that points to the category, if found.  */
00032 #define CHECK_CATEGORY(category)                                        \
00033    std::map<std::string, CCategory*>::iterator itCategory = m_mapCategories.find(category); \
00034    if(itCategory == m_mapCategories.end()) {                            \
00035       THROW_ARGOSEXCEPTION("CRandom:: can't find category \"" << category << "\"."); \
00036    }
00037 
00038    /****************************************/
00039    /****************************************/
00040 
00041    CRandom::CRNG::CRNG(UInt32 un_seed) :
00042       m_unSeed(un_seed),
00043       m_punState(new UInt32[N]),
00044       m_nIndex(N+1) {
00045       Reset();
00046    }
00047    
00048    /****************************************/
00049    /****************************************/
00050    
00051    CRandom::CRNG::CRNG(const CRNG& c_rng) :
00052       m_unSeed(c_rng.m_unSeed),
00053       m_punState(new UInt32[N]),
00054       m_nIndex(c_rng.m_nIndex) {
00055       ::memcpy(m_punState, c_rng.m_punState, N * sizeof(UInt32));
00056    }
00057 
00058    /****************************************/
00059    /****************************************/
00060 
00061    CRandom::CRNG::~CRNG() {
00062       delete[] m_punState;
00063    }
00064 
00065    /****************************************/
00066    /****************************************/
00067 
00068    void CRandom::CRNG::Reset() {
00069       m_punState[0]= m_unSeed & 0xffffffffUL;
00070       for (m_nIndex = 1; m_nIndex < N; ++m_nIndex) {
00071          m_punState[m_nIndex] = 
00072             (1812433253UL * (m_punState[m_nIndex-1] ^ (m_punState[m_nIndex-1] >> 30)) + m_nIndex); 
00073          m_punState[m_nIndex] &= 0xffffffffUL;
00074       }
00075    }
00076    
00077    /****************************************/
00078    /****************************************/
00079 
00080    bool CRandom::CRNG::Bernoulli(Real f_true) {
00081       return Uniform32bit() < f_true * INT_RANGE.GetMax();
00082    }
00083 
00084    /****************************************/
00085    /****************************************/
00086 
00087    CRadians CRandom::CRNG::Uniform(const CRange<CRadians>& c_range) {
00088       CRadians cRetVal;
00089       INT_RANGE.MapValueIntoRange(cRetVal, Uniform32bit(), c_range);
00090       return cRetVal;
00091    }
00092    
00093    /****************************************/
00094    /****************************************/
00095 
00096    Real CRandom::CRNG::Uniform(const CRange<Real>& c_range) {
00097       Real fRetVal;
00098       INT_RANGE.MapValueIntoRange(fRetVal, Uniform32bit(), c_range);
00099       return fRetVal;
00100    }
00101    
00102    /****************************************/
00103    /****************************************/
00104 
00105    SInt32 CRandom::CRNG::Uniform(const CRange<SInt32>& c_range) {
00106       SInt32 nRetVal;
00107       INT_RANGE.MapValueIntoRange(nRetVal, Uniform32bit(), c_range);
00108       return nRetVal;
00109    }
00110    
00111    /****************************************/
00112    /****************************************/
00113 
00114    UInt32 CRandom::CRNG::Uniform(const CRange<UInt32>& c_range) {
00115       UInt32 unRetVal;
00116       INT_RANGE.MapValueIntoRange(unRetVal, Uniform32bit(), c_range);
00117       return unRetVal;
00118    }
00119    
00120    /****************************************/
00121    /****************************************/
00122 
00123    Real CRandom::CRNG::Exponential(Real f_mean) {
00124       static CRange<Real> fRange(0.0f, 1.0f);
00125       return -Log(Uniform(fRange)) * f_mean;
00126    }
00127    
00128    /****************************************/
00129    /****************************************/
00130 
00131    Real CRandom::CRNG::Gaussian(Real f_std_dev,
00132                                 Real f_mean) {
00133       /* This is the Box-Muller method in its cartesian variant
00134          see http://www.dspguru.com/dsp/howtos/how-to-generate-white-gaussian-noise
00135       */
00136       static CRange<Real> fRange(-1.0f, 1.0f);
00137       Real fNum1, fNum2;
00138       Real fSquare;
00139       do {
00140          fNum1 = Uniform(fRange);
00141          fNum2 = Uniform(fRange);
00142          fSquare = fNum1 * fNum1 + fNum2 * fNum2;
00143       } while(fSquare >= 1);
00144       return f_mean + f_std_dev * fNum1 * Sqrt(-2.0f * Log(fSquare) / fSquare);
00145    }
00146 
00147    /****************************************/
00148    /****************************************/
00149 
00150    Real CRandom::CRNG::Rayleigh(Real f_sigma) {
00151       /* Draw a number uniformly from (0,1) --- bounds excluded */
00152       static CRange<Real> cUnitRange(0.0f, 1.0f);
00153       Real fValue;
00154       do {
00155          fValue = Uniform(cUnitRange);
00156       }
00157       while(! cUnitRange.WithinMinBoundExcludedMaxBoundExcluded(fValue));
00158       /* Calculate the value to return from the definition of Rayleigh distribution
00159        * http://en.wikipedia.org/wiki/Rayleigh_distribution#Generating_Rayleigh-distributed_random_variates
00160        */
00161       return f_sigma * Sqrt(-2.0f * Log(fValue));
00162    }
00163 
00164 /****************************************/
00165    /****************************************/
00166 
00167    Real CRandom::CRNG::Lognormal(Real f_sigma, Real f_mu) {
00168       /* Draw a number uniformly from (0,1) */
00169       Real fValue;
00170       fValue = Gaussian(1,0);
00171       /* Calculate the value to return from the definition of Lognormal distribution
00172        * http://en.wikipedia.org/wiki/Log-normal_distribution#Generating_log-normally_distributed_random_variates
00173        */
00174       return std::exp(f_mu + f_sigma * fValue);
00175    }
00176    
00177    /****************************************/
00178    /****************************************/
00179 
00180    UInt32 CRandom::CRNG::Uniform32bit() {
00181       UInt32 y;
00182       static UInt32 mag01[2] = { 0x0UL, MATRIX_A };
00183       /* mag01[x] = x * MATRIX_A  for x=0,1 */
00184       
00185       if (m_nIndex >= N) { /* generate N words at one time */
00186          SInt32 kk;
00187          for (kk = 0; kk < N - M; ++kk) {
00188             y = (m_punState[kk] & UPPER_MASK) | (m_punState[kk+1] & LOWER_MASK);
00189             m_punState[kk] = m_punState[kk+M] ^ (y >> 1) ^ mag01[y & 0x1UL];
00190          }
00191          for (; kk < N - 1; ++kk) {
00192             y = (m_punState[kk] & UPPER_MASK) | (m_punState[kk+1] & LOWER_MASK);
00193             m_punState[kk] = m_punState[kk+(M-N)] ^ (y >> 1) ^ mag01[y & 0x1UL];
00194          }
00195          y = (m_punState[N-1] & UPPER_MASK) | (m_punState[0] & LOWER_MASK);
00196          m_punState[N-1] = m_punState[M-1] ^ (y >> 1) ^ mag01[y & 0x1UL];
00197          
00198          m_nIndex = 0;
00199       }
00200       
00201       y = m_punState[m_nIndex++];
00202       
00203       /* Tempering */
00204       y ^= (y >> 11);
00205       y ^= (y << 7) & 0x9d2c5680UL;
00206       y ^= (y << 15) & 0xefc60000UL;
00207       y ^= (y >> 18);
00208       
00209       return y;
00210    }
00211    
00212    /****************************************/
00213    /****************************************/
00214 
00215    CRandom::CCategory::CCategory(const std::string& str_id,
00216                                  UInt32 un_seed) :
00217       m_strId(str_id),
00218       m_unSeed(un_seed),
00219       m_cSeeder(un_seed),
00220       m_cSeedRange(1, std::numeric_limits<UInt32>::max()) {}
00221 
00222    /****************************************/
00223    /****************************************/
00224 
00225    CRandom::CCategory::~CCategory() {
00226       while(! m_vecRNGList.empty()) {
00227          delete m_vecRNGList.back();
00228          m_vecRNGList.pop_back();
00229       }
00230    }
00231 
00232    /****************************************/
00233    /****************************************/
00234 
00235    void CRandom::CCategory::SetSeed(UInt32 un_seed) {
00236       m_unSeed = un_seed;
00237       m_cSeeder.SetSeed(m_unSeed);
00238    }
00239 
00240    /****************************************/
00241    /****************************************/
00242 
00243    CRandom::CRNG* CRandom::CCategory::CreateRNG() {
00244       /* Get seed from internal RNG */
00245       UInt32 unSeed = m_cSeeder.Uniform(m_cSeedRange);
00246       /* Create new RNG */
00247       m_vecRNGList.push_back(new CRNG(unSeed));
00248       return m_vecRNGList.back();
00249    }
00250 
00251    /****************************************/
00252    /****************************************/
00253 
00254    void CRandom::CCategory::ResetRNGs() {
00255       /* Reset internal RNG */
00256       m_cSeeder.Reset();
00257       ReseedRNGs();
00258       /* Reset the RNGs */
00259       for(size_t i = 0; i < m_vecRNGList.size(); ++i) {
00260          m_vecRNGList[i]->Reset();
00261       }
00262    }
00263 
00264    /****************************************/
00265    /****************************************/
00266 
00267    void CRandom::CCategory::ReseedRNGs() {
00268       for(size_t i = 0; i < m_vecRNGList.size(); ++i) {
00269          /* Get seed from internal RNG */
00270          m_vecRNGList[i]->SetSeed(m_cSeeder.Uniform(m_cSeedRange));
00271       }
00272    }
00273 
00274    /****************************************/
00275    /****************************************/
00276 
00277    bool CRandom::CreateCategory(const std::string& str_category,
00278                                 UInt32 un_seed) {
00279       /* Is there a category already? */
00280       std::map<std::string, CCategory*>::iterator itCategory = m_mapCategories.find(str_category);
00281       if(itCategory == m_mapCategories.end()) {
00282          /* No, create it */
00283          m_mapCategories.insert(
00284             std::pair<std::string,
00285             CRandom::CCategory*>(str_category,
00286                                  new CRandom::CCategory(str_category,
00287                                                         un_seed)));
00288          return true;
00289       }
00290       return false;
00291    }
00292 
00293    /****************************************/
00294    /****************************************/
00295 
00296    CRandom::CCategory& CRandom::GetCategory(const std::string& str_category) {
00297       CHECK_CATEGORY(str_category);
00298       return *(itCategory->second);
00299    }
00300 
00301    /****************************************/
00302    /****************************************/
00303 
00304    bool CRandom::ExistsCategory(const std::string& str_category) {
00305       try {
00306          CHECK_CATEGORY(str_category);
00307          return true;
00308       }
00309       catch(CARGoSException& ex) {
00310          return false;
00311       }
00312    }
00313 
00314    /****************************************/
00315    /****************************************/
00316 
00317    void CRandom::RemoveCategory(const std::string& str_category) {
00318       CHECK_CATEGORY(str_category);
00319       delete itCategory->second;
00320       m_mapCategories.erase(itCategory);
00321    }
00322 
00323    /****************************************/
00324    /****************************************/
00325 
00326    CRandom::CRNG* CRandom::CreateRNG(const std::string& str_category) {
00327       CHECK_CATEGORY(str_category);
00328       return itCategory->second->CreateRNG();
00329    }
00330    
00331    /****************************************/
00332    /****************************************/
00333 
00334    UInt32 CRandom::GetSeedOf(const std::string& str_category) {
00335       CHECK_CATEGORY(str_category);
00336       return itCategory->second->GetSeed();
00337    }
00338 
00339    /****************************************/
00340    /****************************************/
00341 
00342    void CRandom::SetSeedOf(const std::string& str_category,
00343                            UInt32 un_seed) {
00344       CHECK_CATEGORY(str_category);
00345       itCategory->second->SetSeed(un_seed);
00346    }
00347 
00348    /****************************************/
00349    /****************************************/
00350 
00351    void CRandom::Reset() {
00352       for(std::map<std::string, CCategory*>::iterator itCategory = m_mapCategories.begin();
00353           itCategory != m_mapCategories.end();
00354           ++itCategory) {
00355          itCategory->second->ResetRNGs();
00356       }
00357    }
00358 
00359    /****************************************/
00360    /****************************************/
00361 
00362 }
```

---

Generated on 10 Jul 2018 for ARGoS by 
 1.6.1 
