# Supplementary material for: Iterative improvement in the automatic modular design of robot swarms
Source: PeerJ Comput Sci. 2020 Dec 7;6:e322. doi: 10.7717/peerj-cs.322 (PMC7924708; doi:10.7717/peerj-cs.322)
Supplement: Supplemental Information 3 [file peerj-cs-06-322-s003.zip › argos3/doc/api/standalone/a00390_source.html]

ARGoS: core/utility/math/rng.h Source File


- Main Page
- Related Pages
- Namespaces
- Classes
- Files

- File List
- File Members

# core/utility/math/rng.h

Go to the documentation of this file.

```
00001 
00009 #ifndef ARGOS_RANDOM
00010 #define ARGOS_RANDOM
00011 
00012 namespace argos {
00013    class CRandom;
00014 }
00015 
00016 #include <argos3/core/utility/math/angles.h>
00017 #include <argos3/core/utility/math/range.h>
00018 #include <map>
00019 
00020 namespace argos {
00021 
00081    class CRandom {
00082 
00083    public:
00084 
00090       class CRNG {
00091 
00092       public:
00093 
00100          CRNG(UInt32 un_seed);
00101 
00106          CRNG(const CRNG& c_rng);
00107 
00111          virtual ~CRNG();
00112 
00117          inline UInt32 GetSeed() const throw() {
00118             return m_unSeed;
00119          }
00120 
00127          inline void SetSeed(UInt32 un_seed) throw() {
00128             m_unSeed = un_seed;
00129          }
00130 
00135          void Reset();
00136 
00142          bool Bernoulli(Real f_true = 0.5);
00143          
00149          CRadians Uniform(const CRange<CRadians>& c_range);
00150          
00156          Real Uniform(const CRange<Real>& c_range);
00157          
00163          SInt32 Uniform(const CRange<SInt32>& c_range);
00164          
00170          UInt32 Uniform(const CRange<UInt32>& c_range);
00171          
00177          Real Exponential(Real f_mean);
00178          
00185          Real Gaussian(Real f_std_dev, Real f_mean = 0.0f);
00186          
00192          Real Rayleigh(Real f_sigma);
00193          
00200          Real Lognormal(Real f_sigma, Real f_mu);
00201 
00202       private:
00203 
00204          /*
00205           * Generates a random 32bit unsigned integer.
00206           * Used internally by all other functions.
00207           */
00208          UInt32 Uniform32bit();
00209 
00210       private:
00211 
00212          UInt32 m_unSeed;
00213          UInt32* m_punState;
00214          SInt32 m_nIndex;
00215 
00216       };
00217 
00222       class CCategory {
00223 
00224       public:
00225 
00231          CCategory(const std::string& str_id,
00232                    UInt32 un_seed);
00233 
00237          virtual ~CCategory();
00238 
00243          inline const std::string& GetId() const throw() {
00244             return m_strId;
00245          }
00250          void SetId(const std::string& str_id) {
00251             m_strId = str_id;
00252          }
00253 
00258          inline UInt32 GetSeed() const {
00259             return m_unSeed;
00260          }
00267          void SetSeed(UInt32 un_seed);
00268 
00273          CRNG* CreateRNG();
00274 
00278          void ResetRNGs();
00279 
00285          void ReseedRNGs();
00286 
00287       private:
00288 
00289          std::string m_strId;
00290          std::vector<CRNG*> m_vecRNGList;
00291          UInt32 m_unSeed;
00292          CRNG m_cSeeder;
00293          CRange<UInt32> m_cSeedRange;
00294       };
00295 
00296    public:
00297 
00304       static bool CreateCategory(const std::string& str_category,
00305                                  UInt32 un_seed);
00311       static CCategory& GetCategory(const std::string& str_category);
00312 
00318       static bool ExistsCategory(const std::string& str_category);
00319 
00324       static void RemoveCategory(const std::string& str_category);
00325 
00331       static CRNG* CreateRNG(const std::string& str_category);
00332 
00338       static UInt32 GetSeedOf(const std::string& str_category);
00339 
00347       static void SetSeedOf(const std::string& str_category,
00348                             UInt32 un_seed);
00349 
00353       static void Reset();
00354 
00355    private:
00356 
00357       static std::map<std::string, CCategory*> m_mapCategories;
00358    };
00359 
00360 }
00361 
00362 #endif
```

---

Generated on 10 Jul 2018 for ARGoS by 
 1.6.1 
