# Supplementary material for: Iterative improvement in the automatic modular design of robot swarms
Source: PeerJ Comput Sci. 2020 Dec 7;6:e322. doi: 10.7717/peerj-cs.322 (PMC7924708; doi:10.7717/peerj-cs.322)
Supplement: Supplemental Information 3 [file peerj-cs-06-322-s003.zip › argos3/doc/api/standalone/a00391_source.html]

ARGoS: core/utility/math/vector2.cpp Source File


- Main Page
- Related Pages
- Namespaces
- Classes
- Files

- File List
- File Members

# core/utility/math/vector2.cpp

Go to the documentation of this file.

```
00001 
00008 #include "vector2.h"
00009 
00010 namespace argos {
00011 
00012    const CVector2 CVector2::X(1.0, 0.0);
00013    const CVector2 CVector2::Y(0.0, 1.0);
00014 
00015 }
```

---

Generated on 10 Jul 2018 for ARGoS by 
 1.6.1 
