# Supplementary material for: Iterative improvement in the automatic modular design of robot swarms
Source: PeerJ Comput Sci. 2020 Dec 7;6:e322. doi: 10.7717/peerj-cs.322 (PMC7924708; doi:10.7717/peerj-cs.322)
Supplement: Supplemental Information 3 [file peerj-cs-06-322-s003.zip › argos3/doc/api/standalone/a00392_source.html]

ARGoS: core/utility/math/vector2.h Source File


- Main Page
- Related Pages
- Namespaces
- Classes
- Files

- File List
- File Members

# core/utility/math/vector2.h

Go to the documentation of this file.

```
00001 
00007 #ifndef VECTOR2_H
00008 #define VECTOR2_H
00009 
00010 namespace argos {
00011    class CRotationMatrix2;
00012 }
00013 
00014 #include <argos3/core/utility/math/general.h>
00015 #include <argos3/core/utility/math/angles.h>
00016 #include <argos3/core/utility/string_utilities.h>
00017 #include <iostream>
00018 #include <cmath>
00019 
00020 namespace argos {
00021 
00025    class CVector2 {
00026    
00027    friend class CRotationMatrix2;
00028    friend class CTransformationMatrix2;
00029 
00030    public:
00031 
00033       static const CVector2 X;
00034 
00036       static const CVector2 Y;
00037 
00043       CVector2() :
00044          m_fX(0.0),
00045          m_fY(0.0) {
00046       }
00047 
00055       CVector2(Real f_x,
00056                Real f_y) :
00057          m_fX(f_x),
00058          m_fY(f_y) {
00059       }
00060 
00068       CVector2(Real f_length,
00069                const CRadians& f_angle) :
00070          m_fX(Cos(f_angle) * f_length),
00071          m_fY(Sin(f_angle) * f_length) {
00072       }
00073 
00078       inline Real GetX() const {
00079          return m_fX;
00080       }
00081 
00086       inline void SetX(Real f_x) {
00087          m_fX = f_x;
00088       }
00089 
00094       inline Real GetY() const {
00095          return m_fY;
00096       }
00097 
00102       inline void SetY(Real f_y) {
00103          m_fY = f_y;
00104       }
00105 
00111       inline void Set(Real f_x, Real f_y) {
00112          m_fX = f_x;
00113          m_fY = f_y;
00114       }
00115 
00123       inline void FromPolarCoordinates(Real f_length,
00124                                        const CRadians& f_angle) {
00125          m_fX = Cos(f_angle) * f_length;
00126          m_fY = Sin(f_angle) * f_length;
00127       }
00128 
00133       inline Real SquareLength() const {
00134          return Square(m_fX) + Square(m_fY);
00135       }
00136 
00141       inline Real Length() const {
00142          return Sqrt(SquareLength());
00143       }
00144 
00151       inline CVector2& Normalize() {
00152          *this /= Length();
00153          return *this;
00154       }
00155 
00160       inline CRadians Angle() const {
00161          return ATan2(m_fY, m_fX);
00162       }
00163 
00169       inline CVector2& Rotate(const CRadians& c_angle) {
00170          Real fSin, fCos;
00171 #ifdef ARGOS_SINCOS
00172          SinCos(c_angle, fSin, fCos);
00173 #else
00174          fSin = Sin(c_angle);
00175          fCos = Cos(c_angle);
00176 #endif
00177          Real fX = m_fX * fCos - m_fY * fSin;
00178          Real fY = m_fX * fSin + m_fY * fCos;
00179          m_fX = fX;
00180          m_fY = fY;
00181          return *this;
00182       }
00183 
00189       inline Real DotProduct(const CVector2& c_vector2) const {
00190          return m_fX * c_vector2.m_fX + m_fY * c_vector2.m_fY;
00191       }
00192 
00198       inline Real CrossProduct(const CVector2& c_vector2) const {
00199          return m_fX * c_vector2.m_fY + m_fY * c_vector2.m_fX;
00200       }
00201 
00211       inline CVector2& Scale(Real f_scale_x,
00212                              Real f_scale_y) {
00213          m_fX *= f_scale_x;
00214          m_fY *= f_scale_y;
00215          return *this;
00216       }
00217 
00222       inline CVector2& Perpendicularize() {
00223          Real fNewX = -m_fY;
00224          m_fY = m_fX;
00225          m_fX = fNewX;
00226          return *this;
00227       }
00228 
00233       inline CVector2& Absolute() {
00234          m_fX = Abs(m_fX);
00235          m_fY = Abs(m_fY);
00236          return *this;
00237       }
00238 
00245       inline bool operator==(const CVector2& c_vector2) const {
00246          return (m_fX == c_vector2.m_fX && m_fY == c_vector2.m_fY);
00247       }
00248 
00255       inline bool operator!=(const CVector2& c_vector2) const {
00256          return (m_fX != c_vector2.m_fX || m_fY != c_vector2.m_fY);
00257       }
00258 
00264       inline CVector2& operator+=(const CVector2& c_vector2) {
00265          m_fX += c_vector2.m_fX;
00266          m_fY += c_vector2.m_fY;
00267          return *this;
00268       }
00269 
00275       inline CVector2& operator-=(const CVector2& c_vector2) {
00276          m_fX -= c_vector2.m_fX;
00277          m_fY -= c_vector2.m_fY;
00278          return *this;
00279       }
00280 
00286       inline CVector2& operator*=(Real f_value) {
00287          m_fX *= f_value;
00288          m_fY *= f_value;
00289          return *this;
00290       }
00291 
00297       inline CVector2& operator/=(Real f_value) {
00298          m_fX /= f_value;
00299          m_fY /= f_value;
00300          return *this;
00301       }
00302 
00308       inline CVector2 operator+(const CVector2& c_vector2) const {
00309          CVector2 cResult(*this);
00310          cResult += c_vector2;
00311          return cResult;
00312       }
00313 
00319       inline CVector2 operator-(const CVector2& c_vector2) const {
00320          CVector2 cResult(*this);
00321          cResult -= c_vector2;
00322          return cResult;
00323       }
00324 
00330       inline CVector2 operator*(Real f_value) const {
00331          CVector2 cResult(*this);
00332          cResult *= f_value;
00333          return cResult;
00334       }
00335 
00341       inline CVector2 operator/(Real f_value) const {
00342          CVector2 cResult(*this);
00343          cResult /= f_value;
00344          return cResult;
00345       }
00346 
00353       inline friend CVector2 operator*(Real f_value,
00354                                        const CVector2& c_vector2) {
00355          return c_vector2 * f_value;
00356       }
00357 
00358       inline CVector2 operator-() const {
00359          return CVector2(-m_fX, -m_fY);
00360       }
00361 
00368       inline friend std::ostream& operator<<(std::ostream& c_os,
00369                                              const CVector2& c_vector2) {
00370          c_os << c_vector2.m_fX << ","
00371               << c_vector2.m_fY;
00372          return c_os;
00373       }
00374 
00381       inline friend std::istream& operator>>(std::istream& c_is,
00382                                              CVector2& c_vector2) {
00383          Real fValues[2];
00384          ParseValues<Real> (c_is, 2, fValues, ',');
00385          c_vector2.Set(fValues[0], fValues[1]);
00386          return c_is;
00387       }
00388 
00389    private:
00390 
00392       Real m_fX;
00393 
00395       Real m_fY;
00396 
00397    };
00398 
00399    /****************************************/
00400    /****************************************/
00401 
00408    inline Real SquareDistance(const CVector2& c_v1, const CVector2& c_v2) {
00409       return (c_v1 - c_v2).SquareLength();
00410    }
00411 
00418    inline Real Distance(const CVector2& c_v1, const CVector2& c_v2) {
00419       return (c_v1 - c_v2).Length();
00420    }
00421 
00422    /****************************************/
00423    /****************************************/
00424 
00425 }
00426 
00427 #endif
```

---

Generated on 10 Jul 2018 for ARGoS by 
 1.6.1 
