# Supplementary material for: Iterative improvement in the automatic modular design of robot swarms
Source: PeerJ Comput Sci. 2020 Dec 7;6:e322. doi: 10.7717/peerj-cs.322 (PMC7924708; doi:10.7717/peerj-cs.322)
Supplement: Supplemental Information 3 [file peerj-cs-06-322-s003.zip › argos3/doc/api/standalone/a00393_source.html]

ARGoS: core/utility/math/vector3.cpp Source File


- Main Page
- Related Pages
- Namespaces
- Classes
- Files

- File List
- File Members

# core/utility/math/vector3.cpp

Go to the documentation of this file.

```
00001 
00009 #include "vector3.h"
00010 #include "quaternion.h"
00011 
00012 namespace argos {
00013 
00014    /****************************************/
00015    /****************************************/
00016 
00017    const CVector3 CVector3::X(1.0, 0.0, 0.0);
00018    const CVector3 CVector3::Y(0.0, 1.0, 0.0);
00019    const CVector3 CVector3::Z(0.0, 0.0, 1.0);
00020    const CVector3 CVector3::ZERO;
00021 
00022    /****************************************/
00023    /****************************************/
00024 
00025    CVector3& CVector3::Rotate(const CQuaternion& c_quaternion) {
00026       CQuaternion cResult;
00027       cResult = c_quaternion;
00028       cResult *= CQuaternion(0.0f, m_fX, m_fY, m_fZ);
00029       cResult *= c_quaternion.Inverse();
00030       m_fX = cResult.GetX();
00031       m_fY = cResult.GetY();
00032       m_fZ = cResult.GetZ();
00033       return *this;
00034    }
00035 
00036    /****************************************/
00037    /****************************************/
00038 
00039 }
```

---

Generated on 10 Jul 2018 for ARGoS by 
 1.6.1 
