# Supplementary material for: Iterative improvement in the automatic modular design of robot swarms
Source: PeerJ Comput Sci. 2020 Dec 7;6:e322. doi: 10.7717/peerj-cs.322 (PMC7924708; doi:10.7717/peerj-cs.322)
Supplement: Supplemental Information 3 [file peerj-cs-06-322-s003.zip › argos3/doc/api/standalone/a00394.html]

ARGoS: core/utility/math/vector3.h File Reference


- Main Page
- Related Pages
- Namespaces
- Classes
- Files

- File List
- File Members

# core/utility/math/vector3.h File Reference

`#include <argos3/core/utility/math/general.h>`  
`#include <argos3/core/utility/math/angles.h>`  
`#include <argos3/core/utility/math/vector2.h>`  
`#include <argos3/core/utility/string_utilities.h>`  
`#include <iostream>`  
`#include <cmath>`  

Include dependency graph for vector3.h:

This graph shows which files directly or indirectly include this file:

Go to the source code of this file.

|  |  |
| --- | --- |
| Classes | |
| class | argos::CVector3 |
|  | A 3D vector class. More... |
| Namespaces | |
| namespace | argos |

|  |  |
| --- | --- |
|  | The namespace containing all the ARGoS related code. |

| Functions | |
| Real | argos::SquareDistance (const CVector3 &c\_v1, const CVector3 &c\_v2) |
|  | Computes the square distance between the passed vectors. |
| Real | argos::Distance (const CVector3 &c\_v1, const CVector3 &c\_v2) |
|  | Computes the distance between the passed vectors. |

---

Generated on 10 Jul 2018 for ARGoS by 
 1.6.1 
