# Supplementary material for: Iterative improvement in the automatic modular design of robot swarms
Source: PeerJ Comput Sci. 2020 Dec 7;6:e322. doi: 10.7717/peerj-cs.322 (PMC7924708; doi:10.7717/peerj-cs.322)
Supplement: Supplemental Information 3 [file peerj-cs-06-322-s003.zip › argos3/doc/api/standalone/a00394_source.html]

ARGoS: core/utility/math/vector3.h Source File


- Main Page
- Related Pages
- Namespaces
- Classes
- Files

- File List
- File Members

# core/utility/math/vector3.h

Go to the documentation of this file.

```
00001 
00007 #ifndef VECTOR3_H
00008 #define VECTOR3_H
00009 
00010 namespace argos {
00011    class CVector3;
00012    class CVector2;
00013    class CQuaternion;
00014    class CRotationMatrix3;
00015 }
00016 
00017 #include <argos3/core/utility/math/general.h>
00018 #include <argos3/core/utility/math/angles.h>
00019 #include <argos3/core/utility/math/vector2.h>
00020 #include <argos3/core/utility/string_utilities.h>
00021 #include <iostream>
00022 #include <cmath>
00023 
00024 namespace argos {
00025 
00029    class CVector3 {
00030    
00031    public:
00032 
00034       static const CVector3 X;
00035 
00037       static const CVector3 Y;
00038 
00040       static const CVector3 Z;
00041 
00043       static const CVector3 ZERO;
00044 
00045    public:
00046 
00052       CVector3() :
00053          m_fX(0.0),
00054          m_fY(0.0),
00055          m_fZ(0.0) {
00056       }
00057 
00066       CVector3(Real f_x,
00067                Real f_y,
00068                Real f_z) :
00069          m_fX(f_x),
00070          m_fY(f_y),
00071          m_fZ(f_z) {
00072       }
00073 
00083       inline CVector3(Real f_length,
00084                       const CRadians& c_inclination,
00085                       const CRadians& c_azimuth) {
00086          FromSphericalCoords(f_length, c_inclination, c_azimuth);
00087       }
00088 
00093       inline Real GetX() const {
00094          return m_fX;
00095       }
00096 
00101       inline void SetX(const Real f_x) {
00102          m_fX = f_x;
00103       }
00104 
00109       inline Real GetY() const {
00110          return m_fY;
00111       }
00112 
00117       inline void SetY(const Real f_y) {
00118          m_fY = f_y;
00119       }
00120 
00125       inline Real GetZ() const {
00126          return m_fZ;
00127       }
00128 
00133       inline void SetZ(const Real f_z) {
00134          m_fZ = f_z;
00135       }
00136 
00143       inline void Set(const Real f_x,
00144                       const Real f_y,
00145                       const Real f_z) {
00146          m_fX = f_x;
00147          m_fY = f_y;
00148          m_fZ = f_z;
00149       }
00150 
00158       inline CVector3& FromSphericalCoords(Real f_length,
00159                                            const CRadians& c_inclination,
00160                                            const CRadians& c_azimuth) {
00161          Real fInclinationSin, fInclinationCos;
00162          Real fAzimuthSin, fAzimuthCos;
00163 #ifdef ARGOS_SINCOS
00164          SinCos(c_inclination, fInclinationSin, fInclinationCos);
00165          SinCos(c_azimuth, fAzimuthSin, fAzimuthCos);
00166 #else
00167          fInclinationSin = Sin(c_inclination);
00168          fInclinationCos = Cos(c_inclination);
00169          fAzimuthSin = Sin(c_azimuth);
00170          fAzimuthCos = Cos(c_azimuth);
00171 #endif
00172          m_fX = f_length * fInclinationSin * fAzimuthCos;
00173          m_fY = f_length * fInclinationSin * fAzimuthSin;
00174          m_fZ = f_length * fInclinationCos;
00175          return *this;
00176       }
00177 
00185       inline void ToSphericalCoords(Real& f_radius,
00186                                     CRadians& c_inclination,
00187                                     CRadians& c_azimuth) const {
00188          f_radius = Length();
00189          c_inclination = ACos(m_fZ / f_radius);
00190          c_azimuth = ATan2(m_fY, m_fX);
00191       }
00192 
00197       inline Real SquareLength() const {
00198          return Square(m_fX) + Square(m_fY) + Square(m_fZ);
00199       }
00200 
00205       inline Real Length() const {
00206          return Sqrt(SquareLength());
00207       }
00208 
00215       inline CVector3& Normalize() {
00216          *this /= Length();
00217          return *this;
00218       }
00219 
00225       inline CVector3& RotateX(const CRadians& c_angle) {
00226          Real fSin, fCos;
00227 #ifdef ARGOS_SINCOS
00228          SinCos(c_angle, fSin, fCos);
00229 #else
00230          fSin = Sin(c_angle);
00231          fCos = Cos(c_angle);
00232 #endif
00233          Real fNewY = m_fY * fCos - m_fZ * fSin;
00234          Real fNewZ = m_fY * fSin + m_fZ * fCos;
00235          m_fY = fNewY;
00236          m_fZ = fNewZ;
00237          return *this;
00238       }
00239 
00245       inline CVector3& RotateY(const CRadians& c_angle) {
00246          Real fSin, fCos;
00247 #ifdef ARGOS_SINCOS
00248          SinCos(c_angle, fSin, fCos);
00249 #else
00250          fSin = Sin(c_angle);
00251          fCos = Cos(c_angle);
00252 #endif
00253          Real fNewX = m_fX * fCos + m_fZ * fSin;
00254          Real fNewZ = m_fZ * fCos - m_fX * fSin;
00255          m_fX = fNewX;
00256          m_fZ = fNewZ;
00257          return *this;
00258       }
00259 
00265       inline CVector3& RotateZ(const CRadians& c_angle) {
00266          Real fSin, fCos;
00267 #ifdef ARGOS_SINCOS
00268          SinCos(c_angle, fSin, fCos);
00269 #else
00270          fSin = Sin(c_angle);
00271          fCos = Cos(c_angle);
00272 #endif
00273          Real fNewX = m_fX * fCos - m_fY * fSin;
00274          Real fNewY = m_fX * fSin + m_fY * fCos;
00275          m_fX = fNewX;
00276          m_fY = fNewY;
00277          return *this;
00278       }
00279 
00292       inline CVector3& RotateZ(const CVector2& c_vector) {
00293          Real fNewX = m_fX * c_vector.GetX() - m_fY * c_vector.GetY();
00294          Real fNewY = m_fX * c_vector.GetY() + m_fY * c_vector.GetX();
00295          m_fX = fNewX;
00296          m_fY = fNewY;
00297          return *this;
00298       }
00299 
00306       CVector3& Rotate(const CQuaternion& c_quaternion);
00307 
00313       inline CRadians GetAngleWith(const CVector3& c_other) {
00314          return CRadians(ACos(DotProduct(c_other) /
00315                               (Length() *
00316                                c_other.Length())));
00317       }
00318 
00323       inline CRadians GetXAngle() const {
00324          return ATan2(m_fZ, m_fY);
00325       }
00326 
00331       inline CRadians GetYAngle() const {
00332          return ATan2(m_fX, m_fZ);
00333       }
00334 
00339       inline CRadians GetZAngle() const {
00340          return ATan2(m_fY, m_fX);
00341       }
00342 
00348       inline Real DotProduct(const CVector3& c_vector3) const {
00349          return
00350             m_fX * c_vector3.m_fX +
00351             m_fY * c_vector3.m_fY +
00352             m_fZ * c_vector3.m_fZ;
00353       }
00354 
00361       inline CVector3& CrossProduct(const CVector3& c_vector3) {
00362          Real fNewX, fNewY, fNewZ;
00363          fNewX = m_fY * c_vector3.m_fZ - m_fZ * c_vector3.m_fY;
00364          fNewY = m_fZ * c_vector3.m_fX - m_fX * c_vector3.m_fZ;
00365          fNewZ = m_fX * c_vector3.m_fY - m_fY * c_vector3.m_fX;
00366          m_fX = fNewX;
00367          m_fY = fNewY;
00368          m_fZ = fNewZ;
00369          return *this;
00370       }
00371 
00377       inline CVector2& ProjectOntoXY(CVector2& c_proj) const {
00378          c_proj.Set(m_fX,m_fY);
00379          return c_proj;
00380       }
00381 
00387       inline CVector2& ProjectOntoYZ(CVector2& c_proj) const {
00388          c_proj.Set(m_fY,m_fZ);
00389          return c_proj;
00390       }
00391 
00397       inline CVector2& ProjectOntoXZ(CVector2& c_proj) const {
00398          c_proj.Set(m_fX,m_fZ);
00399          return c_proj;
00400       }
00401 
00407       inline CVector3& Negate() {
00408          m_fX = -m_fX;
00409          m_fY = -m_fY;
00410          m_fZ = -m_fZ;
00411          return *this;
00412       }
00413 
00421       inline Real operator[](UInt32 un_index) const {
00422          switch(un_index) {
00423             case 0: return m_fX;
00424             case 1: return m_fY;
00425             case 2: return m_fZ;
00426             default: THROW_ARGOSEXCEPTION("Real Vector3::operator[]: index " << un_index << " out of bounds");
00427          }
00428       }
00429 
00437       inline Real& operator[](UInt32 un_index) {
00438          switch(un_index) {
00439             case 0: return m_fX;
00440             case 1: return m_fY;
00441             case 2: return m_fZ;
00442             default: THROW_ARGOSEXCEPTION("Real Vector3::operator[]: index " << un_index << " out of bounds");
00443          }
00444       }
00445 
00452       inline bool operator==(const CVector3& c_vector3) const {
00453          return m_fX == c_vector3.m_fX && m_fY == c_vector3.m_fY && m_fZ == c_vector3.m_fZ;
00454       }
00455 
00462       inline bool operator!=(const CVector3& c_vector3) const {
00463          return !((*this) == c_vector3);
00464       }
00465 
00473       inline bool operator<(const CVector3& c_vector3) const {
00474          return m_fX < c_vector3.m_fX && m_fY < c_vector3.m_fY && m_fZ < c_vector3.m_fZ;
00475       }
00476 
00484       inline bool operator<=(const CVector3& c_vector3) const {
00485          return m_fX <= c_vector3.m_fX && m_fY <= c_vector3.m_fY && m_fZ <= c_vector3.m_fZ;
00486       }
00487 
00495       inline bool operator>(const CVector3& c_vector3) const {
00496          return m_fX > c_vector3.m_fX && m_fY > c_vector3.m_fY && m_fZ > c_vector3.m_fZ;
00497       }
00498 
00506       inline bool operator>=(const CVector3& c_vector3) const {
00507          return m_fX >= c_vector3.m_fX && m_fY >= c_vector3.m_fY && m_fZ >= c_vector3.m_fZ;
00508       }
00509 
00514       inline CVector3 operator-() const {
00515          return CVector3(-m_fX, -m_fY, -m_fZ);
00516       }
00517 
00523       inline CVector3& operator+=(const CVector3& c_vector3) {
00524          m_fX += c_vector3.m_fX;
00525          m_fY += c_vector3.m_fY;
00526          m_fZ += c_vector3.m_fZ;
00527          return *this;
00528       }
00529 
00535       inline CVector3& operator-=(const CVector3& c_vector3) {
00536          m_fX -= c_vector3.m_fX;
00537          m_fY -= c_vector3.m_fY;
00538          m_fZ -= c_vector3.m_fZ;
00539          return *this;
00540       }
00541 
00547       inline CVector3& operator*=(Real f_value) {
00548          m_fX *= f_value;
00549          m_fY *= f_value;
00550          m_fZ *= f_value;
00551          return *this;
00552       }
00553 
00559       inline CVector3& operator/=(Real f_value) {
00560          m_fX /= f_value;
00561          m_fY /= f_value;
00562          m_fZ /= f_value;
00563          return *this;
00564       }
00565 
00571       inline CVector3 operator+(const CVector3& c_vector3) const {
00572          CVector3 cResult(*this);
00573          cResult += c_vector3;
00574          return cResult;
00575       }
00576 
00582       inline CVector3 operator-(const CVector3& c_vector3) const {
00583          CVector3 cResult(*this);
00584          cResult -= c_vector3;
00585          return cResult;
00586       }
00587 
00593       inline CVector3 operator*(Real f_value) const {
00594          CVector3 cResult(*this);
00595          cResult *= f_value;
00596          return cResult;
00597       }
00598 
00604       inline CVector3 operator/(const Real f_value) const {
00605          CVector3 cResult(*this);
00606          cResult /= f_value;
00607          return cResult;
00608       }
00609 
00616       inline friend CVector3 operator*(Real f_value,
00617                                        const CVector3& c_vector3) {
00618          return c_vector3 * f_value;
00619       }
00620 
00627       inline friend std::ostream& operator<<(std::ostream& c_os,
00628                                              const CVector3& c_vector3) {
00629          c_os << c_vector3.m_fX << ","
00630               << c_vector3.m_fY << ","
00631               << c_vector3.m_fZ;
00632          return c_os;
00633       }
00634 
00641       inline friend std::istream& operator>>(std::istream& c_is,
00642                                              CVector3& c_vector3) {
00643          Real fValues[3];
00644          ParseValues<Real>(c_is, 3, fValues, ',');
00645          c_vector3.Set(fValues[0], fValues[1], fValues[2]);
00646          return c_is;
00647       }
00648       
00649    private:
00650       
00652       Real m_fX;
00653 
00655       Real m_fY;
00656 
00658       Real m_fZ;
00659 
00660       friend class CRotationMatrix3;
00661       friend class CTransformationMatrix3;
00662 
00663    };
00664 
00665    /****************************************/
00666    /****************************************/
00667 
00674    inline Real SquareDistance(const CVector3& c_v1, const CVector3& c_v2) {
00675       return (c_v1 - c_v2).SquareLength();
00676    }
00677 
00684    inline Real Distance(const CVector3& c_v1, const CVector3& c_v2) {
00685       return (c_v1 - c_v2).Length();
00686    }
00687 
00688 /****************************************/
00689 /****************************************/
00690 
00691 }
00692 
00693 #endif
```

---

Generated on 10 Jul 2018 for ARGoS by 
 1.6.1 
