# Supplementary material for: Iterative improvement in the automatic modular design of robot swarms
Source: PeerJ Comput Sci. 2020 Dec 7;6:e322. doi: 10.7717/peerj-cs.322 (PMC7924708; doi:10.7717/peerj-cs.322)
Supplement: Supplemental Information 3 [file peerj-cs-06-322-s003.zip › argos3/doc/api/standalone/a00395_source.html]

ARGoS: core/utility/networking/tcp\_socket.cpp Source File


- Main Page
- Related Pages
- Namespaces
- Classes
- Files

- File List
- File Members

# core/utility/networking/tcp\_socket.cpp

Go to the documentation of this file.

```
00001 #include "tcp_socket.h"
00002 
00003 #include <argos3/core/utility/string_utilities.h>
00004 
00005 #include <arpa/inet.h>
00006 #include <cstring>
00007 #include <errno.h>
00008 #include <netdb.h>
00009 #include <sys/types.h>
00010 #include <sys/socket.h>
00011 #include <unistd.h>
00012 
00013 namespace argos {
00014 
00015    /****************************************/
00016    /****************************************/
00017 
00018    CTCPSocket::CTCPSocket(int n_stream) :
00019       m_nStream(n_stream) {
00020    }
00021 
00022    /****************************************/
00023    /****************************************/
00024 
00025    CTCPSocket::~CTCPSocket() {
00026       Disconnect();
00027    }
00028 
00029    /****************************************/
00030    /****************************************/
00031 
00032    void CTCPSocket::Connect(const std::string& str_hostname,
00033                             SInt32 n_port) {
00034       /* Used to store the return value of the network function calls */
00035       int nRetVal;
00036       /* Get information on the available interfaces */
00037       ::addrinfo tHints, *ptInterfaceInfo;
00038       ::memset(&tHints, 0, sizeof(tHints));
00039       tHints.ai_family = AF_INET;       /* Only IPv4 is accepted */
00040       tHints.ai_socktype = SOCK_STREAM; /* TCP socket */
00041       nRetVal = ::getaddrinfo(str_hostname.c_str(),
00042                               ToString(n_port).c_str(),
00043                               &tHints,
00044                               &ptInterfaceInfo);
00045       if(nRetVal != 0) {
00046          THROW_ARGOSEXCEPTION("Error getting address information: " << ::gai_strerror(nRetVal));
00047       }
00048       /* Bind on the first interface available */
00049       m_nStream = -1;
00050       ::addrinfo* ptInterface = NULL;
00051       for(ptInterface = ptInterfaceInfo;
00052           (ptInterface != NULL) && (m_nStream == -1);
00053           ptInterface = ptInterface->ai_next) {
00054          m_nStream = ::socket(ptInterface->ai_family,
00055                               ptInterface->ai_socktype,
00056                               ptInterface->ai_protocol);
00057          if(m_nStream > 0) {
00058             if(::connect(m_nStream,
00059                          ptInterface->ai_addr,
00060                          ptInterface->ai_addrlen) == -1) {
00061                m_nStream = -1;
00062                THROW_ARGOSEXCEPTION("Can't connect to host: " << ::strerror(errno));
00063             }
00064          }
00065       }
00066       ::freeaddrinfo(ptInterfaceInfo);
00067    }
00068 
00069    /****************************************/
00070    /****************************************/
00071 
00072    void CTCPSocket::Listen(SInt32 n_port,
00073                            SInt32 n_queue_length) {
00074       /* Used to store the return value of the network function calls */
00075       int nRetVal;
00076       /* Get information on the available interfaces */
00077       ::addrinfo tHints, *ptInterfaceInfo;
00078       ::memset(&tHints, 0, sizeof(tHints));
00079       tHints.ai_family = AF_INET;       /* Only IPv4 is accepted */
00080       tHints.ai_socktype = SOCK_STREAM; /* TCP socket */
00081       tHints.ai_flags = AI_PASSIVE;     /* Necessary for bind() later on */
00082       nRetVal = ::getaddrinfo(NULL,
00083                               ToString(n_port).c_str(),
00084                               &tHints,
00085                               &ptInterfaceInfo);
00086       if(nRetVal != 0) {
00087          THROW_ARGOSEXCEPTION("Error getting local address information: " << ::gai_strerror(nRetVal));
00088       }
00089       /* Bind on the first interface available */
00090       m_nStream = -1;
00091       ::addrinfo* ptInterface = NULL;
00092       for(ptInterface = ptInterfaceInfo;
00093           (ptInterface != NULL) && (m_nStream == -1);
00094           ptInterface = ptInterface->ai_next) {
00095          m_nStream = ::socket(ptInterface->ai_family,
00096                               ptInterface->ai_socktype,
00097                               ptInterface->ai_protocol);
00098          if(m_nStream > 0) {
00099             int nTrue = 1;
00100             if((::setsockopt(m_nStream,
00101                              SOL_SOCKET,
00102                              SO_REUSEADDR,
00103                              &nTrue,
00104                              sizeof(nTrue)) != -1)
00105                &&
00106                (::bind(m_nStream,
00107                        ptInterface->ai_addr,
00108                        ptInterface->ai_addrlen) == -1)) {
00109                Disconnect();
00110             }
00111          }
00112       }
00113       ::freeaddrinfo(ptInterfaceInfo);
00114       if(m_nStream == -1) {
00115          THROW_ARGOSEXCEPTION("Can't bind socket to any interface");
00116       }
00117       /* Listen on the socket */
00118       if(::listen(m_nStream, n_queue_length) == -1) {
00119          Disconnect();
00120          THROW_ARGOSEXCEPTION("Can't listen on the socket" << ::strerror(errno));
00121       }
00122    }
00123 
00124    /****************************************/
00125    /****************************************/
00126 
00127    void CTCPSocket::Accept(CTCPSocket& c_socket) {
00128       /* Accept connections */
00129       ::sockaddr tAddress;
00130       ::socklen_t tAddressLen = sizeof(tAddress);
00131       int nNewStream = ::accept(m_nStream, &tAddress, &tAddressLen);
00132       if(nNewStream == -1) {
00133          Disconnect();
00134          THROW_ARGOSEXCEPTION("Error accepting connection: " << ::strerror(errno));
00135       }
00136       c_socket.m_nStream = nNewStream;
00137       c_socket.m_strAddress = ::inet_ntoa(reinterpret_cast< ::sockaddr_in* >(&tAddress)->sin_addr);
00138    }
00139 
00140    /****************************************/
00141    /****************************************/
00142 
00143    void CTCPSocket::Disconnect() {
00144       ::close(m_nStream);
00145       m_nStream = -1;
00146       m_strAddress = "";
00147    }
00148 
00149    /****************************************/
00150    /****************************************/
00151 
00152    void CTCPSocket::SendBuffer(const UInt8* pun_buffer,
00153                                size_t un_size) {
00154       ssize_t nSent;
00155       while(un_size > 0) {
00156          nSent = ::send(m_nStream, pun_buffer, un_size, 0);
00157          if(nSent < 0) {
00158             Disconnect();
00159             THROW_ARGOSEXCEPTION("Error sending data: " << ::strerror(errno));
00160          }
00161          un_size -= nSent;
00162          pun_buffer += nSent;
00163       }
00164    }
00165 
00166    /****************************************/
00167    /****************************************/
00168 
00169    bool CTCPSocket::ReceiveBuffer(UInt8* pun_buffer,
00170                                   size_t un_size) {
00171       ssize_t nReceived;
00172       while(un_size > 0) {
00173          nReceived = ::recv(m_nStream, pun_buffer, un_size, 0);
00174          if(nReceived < 0){
00175             Disconnect();
00176              THROW_ARGOSEXCEPTION("Error receiving data: " << ::strerror(errno));
00177          }
00178          if(nReceived == 0) return false;
00179          un_size -= nReceived;
00180          pun_buffer += nReceived;
00181       }
00182       return true;
00183    }
00184 
00185    /****************************************/
00186    /****************************************/
00187 
00188    void CTCPSocket::SendByteArray(const CByteArray& c_byte_array) {
00189       /* Send the length of the byte array */
00190       UInt32 unSizeNBO = htonl(c_byte_array.Size());
00191       SendBuffer(reinterpret_cast<UInt8*>(&unSizeNBO), sizeof(unSizeNBO));
00192       /* Send the actual data */
00193       SendBuffer(c_byte_array.ToCArray(), c_byte_array.Size());
00194    }
00195 
00196    /****************************************/
00197    /****************************************/
00198 
00199    bool CTCPSocket::ReceiveByteArray(CByteArray& c_byte_array) {
00200       /* Receive the length of the byte array */
00201       UInt32 unSizeNBO;
00202       if(ReceiveBuffer(reinterpret_cast<UInt8*>(&unSizeNBO), sizeof(unSizeNBO))) {
00203          /* Receive the actual data */
00204          c_byte_array.Resize(ntohl(unSizeNBO));
00205          if(ReceiveBuffer(c_byte_array.ToCArray(), c_byte_array.Size())) {
00206             return true;
00207          }
00208       }
00209       return false;
00210    }
00211 
00212    /****************************************/
00213    /****************************************/
00214 
00215 }
```

---

Generated on 10 Jul 2018 for ARGoS by 
 1.6.1 
