# Supplementary material for: Iterative improvement in the automatic modular design of robot swarms
Source: PeerJ Comput Sci. 2020 Dec 7;6:e322. doi: 10.7717/peerj-cs.322 (PMC7924708; doi:10.7717/peerj-cs.322)
Supplement: Supplemental Information 3 [file peerj-cs-06-322-s003.zip › argos3/doc/api/standalone/a00396_source.html]

ARGoS: core/utility/networking/tcp\_socket.h Source File


- Main Page
- Related Pages
- Namespaces
- Classes
- Files

- File List
- File Members

# core/utility/networking/tcp\_socket.h

Go to the documentation of this file.

```
00001 #ifndef TCPSOCKET_H
00002 #define TCPSOCKET_H
00003 
00004 namespace argos {
00005    class CTCPSocket;
00006 }
00007 
00008 #include <argos3/core/utility/datatypes/byte_array.h>
00009 #include <argos3/core/utility/datatypes/datatypes.h>
00010 
00011 namespace argos {
00012 
00013    class CTCPSocket {
00014 
00015    public:
00016 
00017       CTCPSocket(int n_stream = -1);
00018 
00019       ~CTCPSocket();
00020 
00025       inline bool IsConnected() const {
00026          return m_nStream != -1;
00027       }
00028 
00033       inline int GetStream() const {
00034          return m_nStream;
00035       }
00036 
00041       inline const std::string& GetAddress() const {
00042          return m_strAddress;
00043       }
00044 
00053       void Connect(const std::string& str_hostname,
00054                    SInt32 n_port);
00055 
00065       void Listen(SInt32 n_port,
00066                   SInt32 n_queue_length = 10);
00067 
00078       void Accept(CTCPSocket& c_socket);
00079 
00084       void Disconnect();
00085 
00092       void SendBuffer(const UInt8* pun_buffer,
00093                       size_t un_size);
00094 
00102       bool ReceiveBuffer(UInt8* pun_buffer,
00103                          size_t un_size);
00104 
00118       void SendByteArray(const CByteArray& c_byte_array);
00119 
00134       bool ReceiveByteArray(CByteArray& c_byte_array);
00135 
00136    private:
00137 
00139       int m_nStream;
00141       std::string m_strAddress;
00142 
00143    };
00144 
00145 }
00146 
00147 #endif
```

---

Generated on 10 Jul 2018 for ARGoS by 
 1.6.1 
