# Supplementary material for: Iterative improvement in the automatic modular design of robot swarms
Source: PeerJ Comput Sci. 2020 Dec 7;6:e322. doi: 10.7717/peerj-cs.322 (PMC7924708; doi:10.7717/peerj-cs.322)
Supplement: Supplemental Information 3 [file peerj-cs-06-322-s003.zip › argos3/doc/api/standalone/a00397_source.html]

ARGoS: core/utility/plugins/dynamic\_loading.cpp Source File


- Main Page
- Related Pages
- Namespaces
- Classes
- Files

- File List
- File Members

# core/utility/plugins/dynamic\_loading.cpp

Go to the documentation of this file.

```
00001 
00007 #include "dynamic_loading.h"
00008 
00009 #include <dirent.h>
00010 #include <cerrno>
00011 
00012 namespace argos {
00013 
00014    /****************************************/
00015    /****************************************/
00016 
00017    CDynamicLoading::TDLHandleMap CDynamicLoading::m_tOpenLibs;
00018    const std::string CDynamicLoading::DEFAULT_PLUGIN_PATH = ARGOS_INSTALL_PREFIX "/lib/argos3/";
00019 
00020    /****************************************/
00021    /****************************************/
00022 
00023    /*
00024     * Tries to load the given library
00025     * 1. Tries to load the library as passed
00026     * 2. If that fails, it appends the shared library extension and tries again;
00027     * 3. If also that fails, it appends the module library extension and tries a last time.
00028     * If all fails, t_handle is set to NULL and str_lib is left as-is;
00029     * In case of success, it sets t_handle to the handle of the load library, and fixes str_lib to
00030     * match the extension of the loaded library.
00031     */
00032    static CDynamicLoading::TDLHandle LoadLibraryTryingExtensions(std::string& str_lib,
00033                                                                  std::string& str_msg) {
00034       /* Try loading without changes to the given path */
00035       CDynamicLoading::TDLHandle tHandle = ::dlopen(str_lib.c_str(), RTLD_GLOBAL | RTLD_LAZY);
00036       str_msg = str_lib + ": ";
00037       if(tHandle == NULL) {
00038          str_msg += dlerror();
00039          /* Try adding the shared lib extension to the path */
00040          std::string strLibWExt = str_lib + "." + ARGOS_SHARED_LIBRARY_EXTENSION;
00041          tHandle = ::dlopen(strLibWExt.c_str(), RTLD_GLOBAL | RTLD_LAZY);
00042          str_msg += "\n" + strLibWExt + ": ";
00043          if(tHandle != NULL) {
00044             /* Success */
00045             str_lib = strLibWExt;
00046          }
00047          else {
00048             str_msg += dlerror();
00049             /* Try adding the module lib extension to the path */
00050             strLibWExt = str_lib + "." + ARGOS_MODULE_LIBRARY_EXTENSION;
00051             tHandle = ::dlopen(strLibWExt.c_str(), RTLD_GLOBAL | RTLD_LAZY);
00052             str_msg += "\n" + strLibWExt + ": ";
00053             if(tHandle != NULL) {
00054                /* Success */
00055                str_lib = strLibWExt;
00056             }
00057          }
00058       }
00059       str_msg += "OK";
00060       return tHandle;
00061    }
00062 
00063    /****************************************/
00064    /****************************************/
00065 
00066    CDynamicLoading::TDLHandle CDynamicLoading::LoadLibrary(const std::string& str_lib) {
00067       TDLHandle tHandle;
00068       /* Check if the provided path is absolute or relative */
00069       if(str_lib[0] == '/') {
00070          /*
00071           * Absolute path
00072           */
00073          /* First check if the library is already loaded */
00074          TDLHandleMap::iterator it = m_tOpenLibs.find(str_lib);
00075          if(it != m_tOpenLibs.end()) {
00076             /* Already loaded */
00077             return m_tOpenLibs[str_lib];
00078          }
00079          /* Not already loaded, load the library and bomb out in case of failure */
00080          std::string strLoadedLib = str_lib;
00081          std::string strMsg;
00082          tHandle = LoadLibraryTryingExtensions(strLoadedLib, strMsg);
00083          if(tHandle == NULL) {
00084             THROW_ARGOSEXCEPTION("Can't load library \""
00085                                  << str_lib
00086                                  << "\" even after trying to add extensions for shared library ("
00087                                  << ARGOS_SHARED_LIBRARY_EXTENSION
00088                                  << ") and module library ("
00089                                  << ARGOS_MODULE_LIBRARY_EXTENSION
00090                                  << "): "
00091                                  << std::endl
00092                                  << strMsg);
00093          }
00094          /* Store the handle to the loaded library */
00095          m_tOpenLibs[strLoadedLib] = tHandle;
00096          LOG << "[INFO] Loaded library \"" << strLoadedLib << "\"" << std::endl;
00097          LOG.Flush();
00098          return tHandle;
00099       }
00100       else {
00101          /*
00102           * Relative path, go through the plugin directories
00103           */
00104          /* String to store the full path to a library */
00105          std::string strLibPath;
00106          /* String to store the list of paths to search */
00107          std::string strPluginPath = ".:" + DEFAULT_PLUGIN_PATH;
00108          /* Get variable ARGOS_PLUGIN_PATH from the environment */
00109          if(::getenv("ARGOS_PLUGIN_PATH") != NULL) {
00110             /* Add value of the variable to list of paths to check */
00111             strPluginPath = std::string(::getenv("ARGOS_PLUGIN_PATH")) + ":" + strPluginPath;
00112          }
00113          /* Add : at the end to make parsing easier */
00114          if(strPluginPath[strPluginPath.length()-1] != ':') {
00115             strPluginPath.append(":");
00116          }
00117          /*
00118           * Go through paths and try to load the library
00119           */
00120          /* Parse the string */
00121          std::istringstream issPluginPath(strPluginPath);
00122          std::string strDir, strMsg;
00123          while(std::getline(issPluginPath, strDir, ':')) {
00124             /* Add '/' to dir if missing */
00125             if(strDir[strDir.length()-1] != '/') {
00126                strDir.append("/");
00127             }
00128             strLibPath = strDir + str_lib;
00129             /* First check if the library is already loaded */
00130             TDLHandleMap::iterator it = m_tOpenLibs.find(strLibPath);
00131             if(it != m_tOpenLibs.end()) {
00132                /* Already loaded */
00133                return m_tOpenLibs[strLibPath];
00134             }
00135             /* Not already loaded, try and load the library */
00136             tHandle = LoadLibraryTryingExtensions(strLibPath, strMsg);
00137             if(tHandle != NULL) {
00138                /* Store the handle to the loaded library */
00139                m_tOpenLibs[strLibPath] = tHandle;
00140                LOG << "[INFO] Loaded library \"" << strLibPath << "\"" << std::endl;
00141                LOG.Flush();
00142                return tHandle;
00143             }
00144          }
00145          /* If we get here, it's because no directory worked */
00146          THROW_ARGOSEXCEPTION("Can't load library \""
00147                               << str_lib
00148                               << "\" even after trying to add extensions for shared library ("
00149                               << ARGOS_SHARED_LIBRARY_EXTENSION
00150                               << ") and module library ("
00151                               << ARGOS_MODULE_LIBRARY_EXTENSION
00152                               << "): "
00153                               << std::endl
00154                               << strMsg);
00155       }
00156    }
00157 
00158    /****************************************/
00159    /****************************************/
00160 
00161    void CDynamicLoading::UnloadLibrary(const std::string& str_lib) {
00162       TDLHandleMap::iterator it = m_tOpenLibs.find(str_lib);
00163       if(it != m_tOpenLibs.end()) {
00164          if(::dlclose(it->second) != 0) {
00165             LOGERR << "[WARNING] Can't unload library \""
00166                    << str_lib
00167                    << "\": "
00168                    << dlerror()
00169                    << std::endl;
00170             LOGERR.Flush();
00171          }
00172       }
00173       else {
00174          THROW_ARGOSEXCEPTION("Can't unload library \""
00175                               << str_lib
00176                               << "\": library does not appear to have been loaded.");
00177       }
00178    }
00179 
00180    /****************************************/
00181    /****************************************/
00182 
00183    void CDynamicLoading::LoadAllLibraries() {      
00184       /* String to store the full path to a library */
00185       std::string strLibPath;
00186       /* String to store the list of paths to search */
00187       std::string strPluginPath = DEFAULT_PLUGIN_PATH;
00188       /* Get variable ARGOS_PLUGIN_PATH from the environment */
00189       if(::getenv("ARGOS_PLUGIN_PATH") != NULL) {
00190          /* Add value of the variable to list of paths to check */
00191          strPluginPath = std::string(::getenv("ARGOS_PLUGIN_PATH")) + ":" + strPluginPath;
00192       }
00193       /* Add : at the end to make parsing easier */
00194       if(strPluginPath[strPluginPath.length()-1] != ':') {
00195          strPluginPath.append(":");
00196       }
00197       /*
00198        * Go through paths and load all the libraries
00199        */
00200       /* Directory info */
00201       DIR* ptDir;
00202       struct dirent* ptDirData;
00203       /* Parse the string */
00204       std::istringstream issPluginPath(strPluginPath);
00205       std::string strDir;
00206       while(std::getline(issPluginPath, strDir, ':')) {
00207          /* Add '/' to dir if missing */
00208          if(strDir[strDir.length()-1] != '/') {
00209             strDir.append("/");
00210          }
00211          /* Try to open the directory */
00212          ptDir = ::opendir(strDir.c_str());
00213          if(ptDir != NULL) {
00214             /* Directory open, now go through the files in the directory */
00215             while((ptDirData = ::readdir(ptDir)) != NULL) {
00216                /* We have a file, check that it is a library file */
00217                if(strlen(ptDirData->d_name) > strlen(ARGOS_SHARED_LIBRARY_EXTENSION) &&
00218                   std::string(ptDirData->d_name).rfind("." ARGOS_SHARED_LIBRARY_EXTENSION) +
00219                   strlen(ARGOS_SHARED_LIBRARY_EXTENSION) + 1 == strlen(ptDirData->d_name)) {
00220                   /* It's a library file, load it */
00221                   LoadLibrary(strDir + ptDirData->d_name);
00222                }
00223                if(strcmp(ARGOS_SHARED_LIBRARY_EXTENSION, ARGOS_MODULE_LIBRARY_EXTENSION) != 0) {
00224                   if(strlen(ptDirData->d_name) > strlen(ARGOS_MODULE_LIBRARY_EXTENSION) &&
00225                      std::string(ptDirData->d_name).rfind("." ARGOS_MODULE_LIBRARY_EXTENSION) +
00226                      strlen(ARGOS_MODULE_LIBRARY_EXTENSION) + 1 == strlen(ptDirData->d_name)) {
00227                      /* It's a library file, load it */
00228                      LoadLibrary(strDir + ptDirData->d_name);
00229                   }
00230                }
00231             }
00232             /* Close directory */
00233             ::closedir(ptDir);
00234          }
00235          else {
00236             /* Error opening directory open, inform user without bombing out */
00237             LOGERR << "[WARNING] Error opening directory \""
00238                    << strDir
00239                    << "\": "
00240                    << ::strerror(errno)
00241                    << std::endl;
00242             LOGERR.Flush();
00243          }
00244       }
00245    }
00246 
00247    /****************************************/
00248    /****************************************/
00249 
00250    void CDynamicLoading::UnloadAllLibraries() {
00251       for(TDLHandleMap::iterator it = m_tOpenLibs.begin();
00252           it != m_tOpenLibs.end();
00253           ++it) {
00254          UnloadLibrary(it->first);
00255       }
00256       m_tOpenLibs.clear();
00257    }
00258 
00259    /****************************************/
00260    /****************************************/
00261 
00262 }
```

---

Generated on 10 Jul 2018 for ARGoS by 
 1.6.1 
