# Supplementary material for: Iterative improvement in the automatic modular design of robot swarms
Source: PeerJ Comput Sci. 2020 Dec 7;6:e322. doi: 10.7717/peerj-cs.322 (PMC7924708; doi:10.7717/peerj-cs.322)
Supplement: Supplemental Information 3 [file peerj-cs-06-322-s003.zip › argos3/doc/api/standalone/a00398_source.html]

ARGoS: core/utility/plugins/dynamic\_loading.h Source File


- Main Page
- Related Pages
- Namespaces
- Classes
- Files

- File List
- File Members

# core/utility/plugins/dynamic\_loading.h

Go to the documentation of this file.

```
00001 
00007 #ifndef DYNAMIC_LOADING_H
00008 #define DYNAMIC_LOADING_H
00009 
00010 #include <argos3/core/utility/configuration/argos_exception.h>
00011 #include <argos3/core/utility/logging/argos_log.h>
00012 
00013 #include <map>
00014 #include <string>
00015 
00016 #include <dlfcn.h>
00017 #include <cstdlib>
00018 
00019 namespace argos {
00020 
00024    class CDynamicLoading {
00025 
00026    public:
00027 
00031       typedef void* TDLHandle;
00032 
00033    public:
00034 
00059       static TDLHandle LoadLibrary(const std::string& str_lib);
00060 
00066       static void UnloadLibrary(const std::string& str_lib);
00067 
00073       static void LoadAllLibraries();
00074 
00079       static void UnloadAllLibraries();
00080 
00081    private:
00082 
00086       typedef std::map<std::string, TDLHandle> TDLHandleMap;
00087 
00091       static TDLHandleMap m_tOpenLibs;
00092 
00096       static const std::string DEFAULT_PLUGIN_PATH;
00097    };
00098 
00099 }
00100 
00101 #endif
```

---

Generated on 10 Jul 2018 for ARGoS by 
 1.6.1 
