# Supplementary material for: Iterative improvement in the automatic modular design of robot swarms
Source: PeerJ Comput Sci. 2020 Dec 7;6:e322. doi: 10.7717/peerj-cs.322 (PMC7924708; doi:10.7717/peerj-cs.322)
Supplement: Supplemental Information 4 [file peerj-cs-06-322-s004.zip › argos3-epuck/Contributors.pdf]

The *argos3-epuck* plugin has been developed at IRIDIA, *Université libre de Bruxelles*, also thanks to funding received from the European Research Council (ERC) under the European Union's Horizon 2020 research and innovation programme (ERC Consolidator project *Demiurge*: grant agreement No 681872)

Main contributors:

- Lorenzo Garattoni
- Gianpiero Francesca
- Carlo Pinciroli
- Mauro Birattari

Other contributors:

- Antoine Ligot
- Arne Brutschy
- Manuele Brambilla
- Brian Delhaisse
- Xavier Deschuyteneer
- Matteo Amaducci
- Davide Vichi
