# Supplementary material for: Iterative improvement in the automatic modular design of robot swarms
Source: PeerJ Comput Sci. 2020 Dec 7;6:e322. doi: 10.7717/peerj-cs.322 (PMC7924708; doi:10.7717/peerj-cs.322)
Supplement: Supplemental Information 4 [file peerj-cs-06-322-s004.zip › argos3-epuck/doc/Installation.pdf]

E-Puck Installation Guide

Lorenzo Garattoni
lgaratto@ulb.ac.be

July 9, 2015

1 Installation

1.1 Simulation

- Install dependencies:

```
sudo apt-get install libgsl0-dev libfreeimage-dev libqt4-dev
    freeglut3-dev libxi-dev libxmu-dev liblua5.1-dev doxygen graphviz
    asciidoc build-essential git cmake cmake-curses-gui
```

- Download argos3 and argos3-epuck:

```
git clone https://github.com/ilpincy/argos3.git ~/argos3

git clone https://iridia-dev.ulb.ac.be/projects/argos3-epuck.git ~/
    argos3-epuck
```

- Configure compilation for argos3:

```
cd ~/argos3

mkdir build_simulator

cd build_simulator

cmake -DCMAKE_BUILD_STYLE=Release ../src
```

- On Ubuntu, you might need to fix broken symlinks with openGL (commands for 32 bit systems):

```
sudo rm /usr/lib/i386-linux-gnu/libGL.so

sudo ln -s $(sudo find /usr/lib/*/mesa -name "libGL.so.*.*.*") /usr/
    lib/i386-linux-gnu/libGL.so
```

- Compile and install argos3:

```
make

make doc

sudo make install
```

- Configure compilation for argos3-epuck:

```
cd ~/argos3-epuck

mkdir build

cd build

cmake -DCMAKE_BUILD_STYLE=Release ../src
```

- Compile and install argos3-epuck:

```
make

sudo make install
```

- Check that everything went fine by running the following command (you should see e-puck among the available entities).

```
argos3 -q entities
```

- You are ready to run your simulations

1.2 Real robot

- Complete the steps listed in Section 1.1.
- Download and install the cross compilation tool (32 bits):

```
cd /tmp

wget mobots.epfl.ch/mx31moboard/sdk/angstrom-2010.4
    -test-20100622-i686-linux-armv6-linux-gnueabi-toolchain-mobots.tar
    .bz2

sudo tar xjvf angstrom-2010.4
    -test-20100622-i686-linux-armv6-linux-gnueabi-toolchain-mobots.tar
    .bz2 -C /
```

- If you are on 64 bits linux with multi arch support (debian testing, ubuntu 3.10, ...): add the support of 32 bits arch

```
sudo dpkg --add-architecture i386

sudo apt-get update
```

- The cross compilation tool is only for 32 bit arch. Add this missing libs:

```
sudo apt-get install libc6:i386 libmpfr4:i386 libgmp10:i386
```

- Fix problem with old libraries:

```
sudo ln -s $(find /usr/lib/i386-linux-gnu -name "libmpfr.so.*.*.*") /
    usr/lib/libmpfr.so.1

sudo ln -s $(find /usr/lib/i386-linux-gnu -name "libgmp.so.*.*.*") /
    usr/lib/libgmp.so.3
```

- Configure the cross-compilation of argos3:

```
cd ~/argos3

mkdir epuck_build

cd epuck_build

cmake -DCMAKE_BUILD_STYLE=Release -DCMAKE_TOOLCHAIN_FILE=~/
    argos3-epuck/src/cmake/TargetEPuck.cmake ../src
```

- Cross-compile and install argos3 (it will be installed in `/usr/local/arm-angstrom-linux-gnueabi/usr/local`)

```
make

sudo make install
```

- Configure the cross-compilation of argos3-epuck: Configure the plugin:

```
cd ~/argos3-epuck

mkdir epuck_build

cd epuck_build

cmake -DCMAKE_BUILD_STYLE=Release -DCMAKE_TOOLCHAIN_FILE=~/
    argos3-epuck/src/cmake/TargetEPuck.cmake ../src
```

- Cross-compile and install argos3-epuck (it will be installed in `/usr/local/arm-angstrom-linux-gnueabi/usr/local`)

```
make

sudo make install
```

1.3 On Real E-Puck

- Connect to the robot(s) via wifi.

  ```
  ssh root@10.0.1.XX
  ```

  Where XX is the number on the top of the e-puck and the password is "root".

- From your PC, upload the argos3 core libraries and the e-puck plugin library on the robot (generated with the steps in Section 1.1 and Section 1.2):

  ```
  ~/argos3-epuck/scripts/upload.sh libs XX
  ```

- Install your controller and its configuration file on the robot: Copy your controller to the e-puck:

  ```
  ~/argos3-epuck/scripts/upload.sh file /path/to/your_controller /
      destination/path/on/robot/cotroller XX
  ```

  ```
  ~/argos3-epuck/scripts/upload.sh file /path/to/your_configuration /
      destination/path/on/robot/configuration XX
  ```

  Connect to the e-puck and run your controller

  ```
  ssh root@10.0.1.XX
  ```

  ```
  ./controller -c configuration -i controller_ID
  ```
