# Supplementary figures and images for: Iterative improvement in the automatic modular design of robot swarms
Source: PeerJ Comput Sci. 2020 Dec 7;6:e322. doi: 10.7717/peerj-cs.322 (PMC7924708; doi:10.7717/peerj-cs.322)

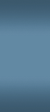

Supplement: Supplemental Information 12 [file peerj-cs-06-322-s012.zip › EvoStick/misc/config/NetworkGraph/doc.html/resources/background.gif]

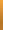

Supplement: Supplemental Information 12 [file peerj-cs-06-322-s012.zip › EvoStick/misc/config/NetworkGraph/doc.html/resources/tab.gif]

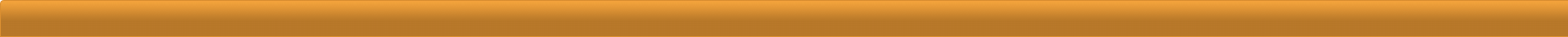

Supplement: Supplemental Information 12 [file peerj-cs-06-322-s012.zip › EvoStick/misc/config/NetworkGraph/doc.html/resources/titlebar.gif]

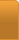

Supplement: Supplemental Information 12 [file peerj-cs-06-322-s012.zip › EvoStick/misc/config/NetworkGraph/doc.html/resources/titlebar_end.gif]

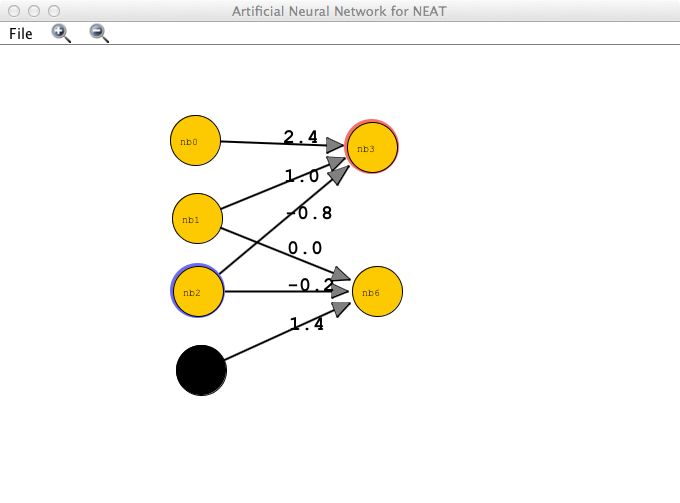

Supplement: Supplemental Information 12 [file peerj-cs-06-322-s012.zip › EvoStick/misc/config/NetworkGraph/examples/drawingTool.png]

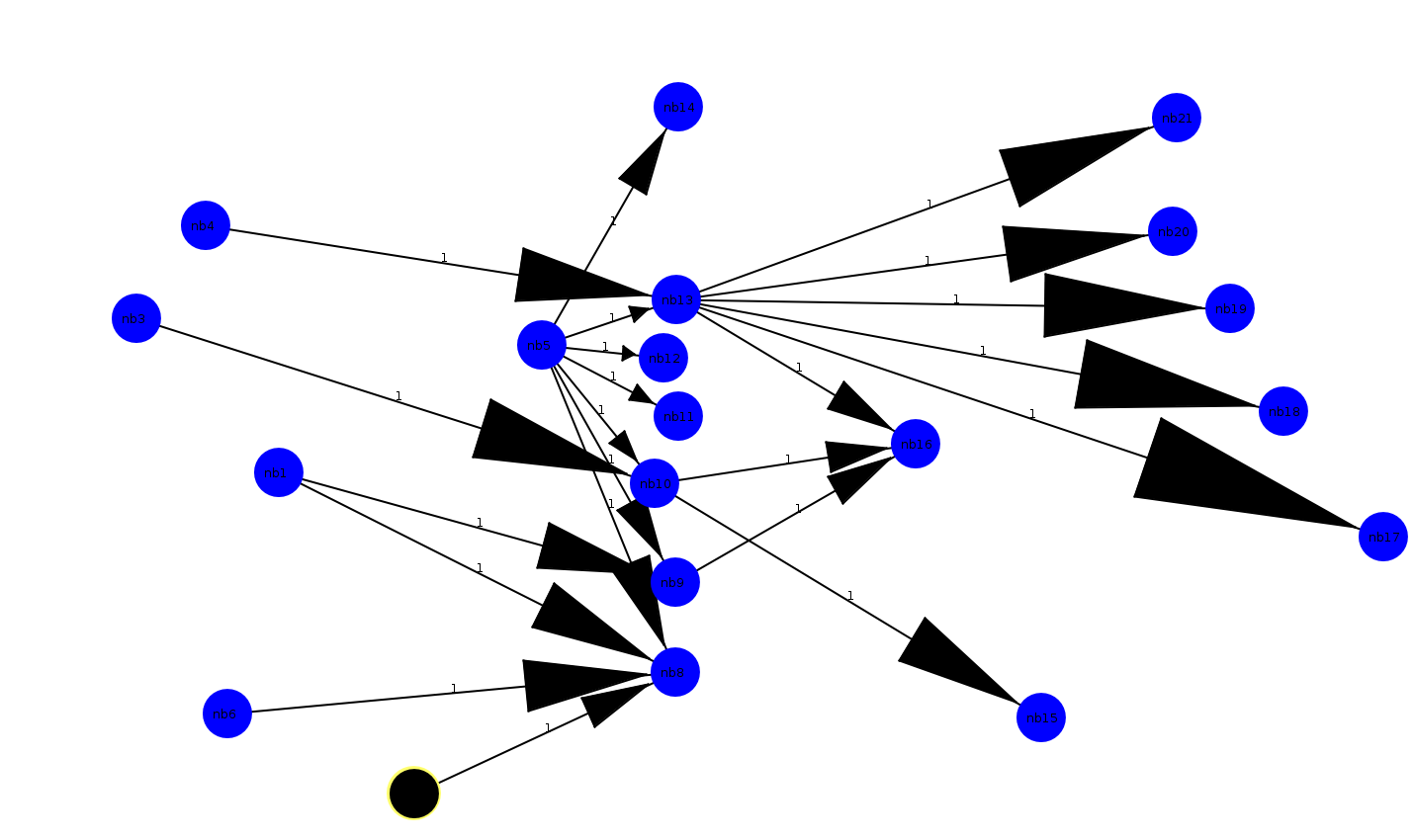

Supplement: Supplemental Information 12 [file peerj-cs-06-322-s012.zip › EvoStick/misc/config/NetworkGraph/examples/v1.png]

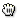

Supplement: Supplemental Information 12 [file peerj-cs-06-322-s012.zip › EvoStick/misc/config/NetworkGraph/images/closed.png]

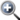

Supplement: Supplemental Information 12 [file peerj-cs-06-322-s012.zip › EvoStick/misc/config/NetworkGraph/images/zoomin.png]

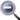

Supplement: Supplemental Information 12 [file peerj-cs-06-322-s012.zip › EvoStick/misc/config/NetworkGraph/images/zoomout.png]

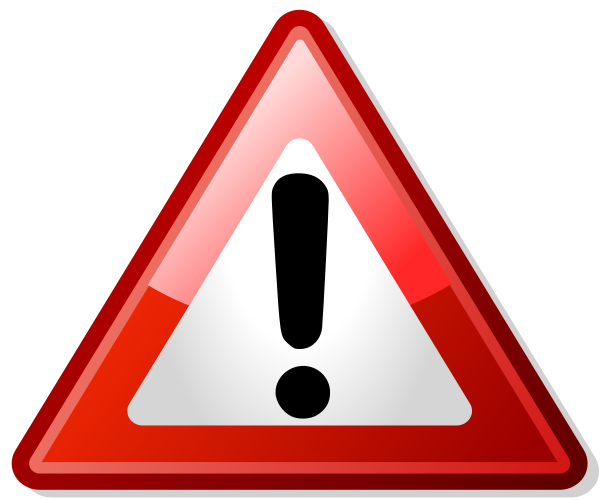

Supplement: Supplemental Information 13 — The R package containing irace [file peerj-cs-06-322-s013.gz › irace/vignettes/Warning-icon.png]

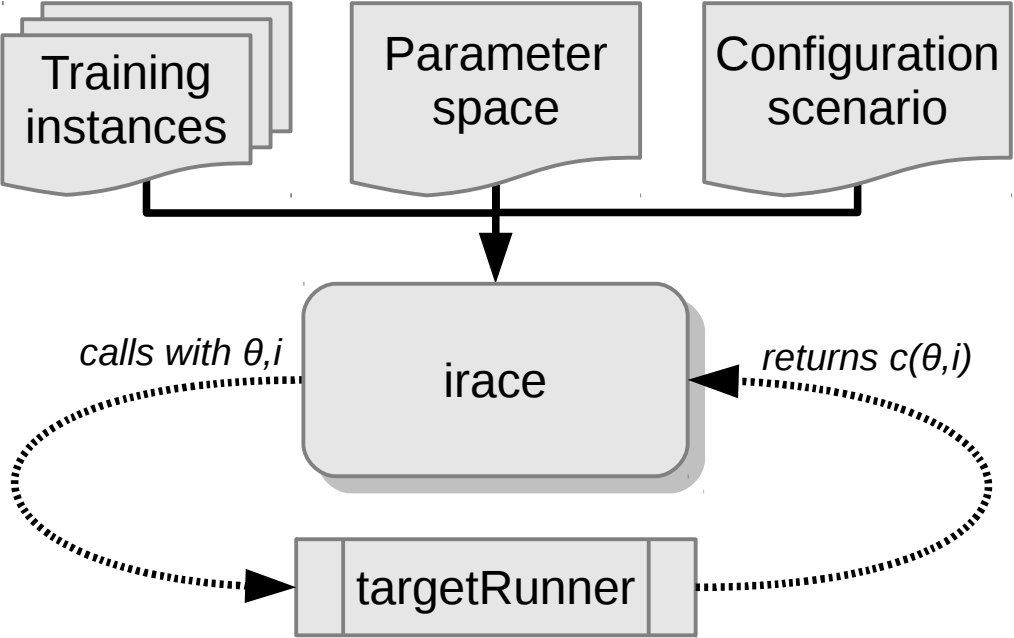

Supplement: Supplemental Information 13 — The R package containing irace [file peerj-cs-06-322-s013.gz › irace/vignettes/irace-scheme.pdf]

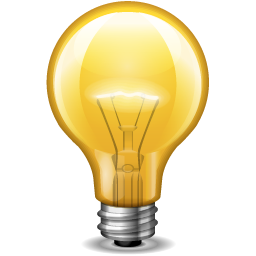

Supplement: Supplemental Information 13 — The R package containing irace [file peerj-cs-06-322-s013.gz › irace/vignettes/light-bulb-icon.png]
