# Supplementary material for: Iterative improvement in the automatic modular design of robot swarms
Source: PeerJ Comput Sci. 2020 Dec 7;6:e322. doi: 10.7717/peerj-cs.322 (PMC7924708; doi:10.7717/peerj-cs.322)
Supplement: Supplemental Information 13 — The R package containing irace [file peerj-cs-06-322-s013.gz › irace/inst/doc/irace-package.pdf]

The **irace** Package: User Guide

Manuel López-Ibáñez, Leslie Pérez Cáceres, Jérémie Dubois-Lacoste,
Thomas Stützle and Mauro Birattari
IRIDIA, CoDE, Université Libre de Bruxelles, Brussels, Belgium

Version 2.2, February 15, 2017

Contents

1 General information **4**
1.1 Background . . . 4
1.2 Version . . . 4
1.3 License . . . 4

2 Before starting **4**

3 Installation **5**
3.1 System requirements . . . 5
3.2 **irace** installation . . . 5
3.2.1 Install automatically within R . . . 6
3.2.2 Manual download and installation . . . 6
3.2.3 Local installation . . . 6
3.2.4 Testing the installation and invoking irace . . . 7

4 Running irace **8**
4.1 Step-by-step setup guide . . . 9
4.2 Setup example for ACOTSP . . . 12

5 Defining a configuration scenario **13**
5.1 Target algorithm parameters . . . 13
5.1.1 Parameter types . . . 13
5.1.2 Parameter domains . . . 14
5.1.3 Conditional parameters . . . 14
5.1.4 Parameter file format . . . 14
5.1.5 Parameters R format . . . 15
5.2 Target algorithm runner . . . 17
5.2.1 Target runner executable program . . . 17
5.2.2 Target runner R function . . . 18
5.3 Target evaluator . . . 19
5.3.1 Target evaluator executable program . . . 20
5.3.2 Target evaluator R function . . . 20
5.4 Training instances . . . 21

5.5 Initial configurations 22
5.6 Forbidden configurations 22

6 Parallelization **23**

7 Testing of configurations **25**

8 Recovering irace runs **25**

9 Output and results **26**
9.1 Text output 26
9.2 Data file output 29
9.3 Analysis of results 34

10 Advanced topics **37**
10.1 Tuning budget 37
10.2 Multi-objective tuning 38
10.3 Tuning for minimizing computation time 38
10.4 Heterogeneous scenarios 39
10.5 Choosing the statistical test 39
10.6 Complex parameters 40
10.7 Unreliable target algorithms 41

11 List of command-line and scenario options **41**
11.1 General options 41
11.2 Elitist **irace** 42
11.3 Internal **irace** options 43
11.4 Target algorithm parameters 44
11.5 Target algorithm execution 44
11.6 Initial configurations 45
11.7 Training instances 45
11.8 Tuning budget 45
11.9 Statistical test 46
11.10 Recovery 46
11.11 Testing 46

12 FAQ **47**
12.1 Is **irace** minimizing or maximizing the output of my algorithm? 47
12.2 Is it possible to configure a MATLAB algorithm with **irace**? 47
12.3 My program works perfectly on its own, but not when running under **irace**. Is irace broken? 47
12.4 My program may be buggy and run into an infinite loop. Is it possible to set a maximum timeout? 47
12.5 When using the mpi option, **irace** is aborted with an error message indicating that a function is not defined. How to fix this? 48
12.6 Error: 4 arguments passed to .Internal(nchar) which requires 3 48

13 Resources and contact information **48**

14 Acknowledgements **49**

Bibliography **49**

Appendix A Installing R **50**
A.1 GNU/Linux 50
A.2 OS X 50
A.3 Windows 50

Appendix B targetRunner troubleshooting checklist **50**

Appendix C Glossary **53**

Appendix D NEWS **53**

1 General information

1.1 Background

The **irace** package implements an *iterated racing* procedure, which is an extension of Iterated F-race (I/F-Race) [2]. The main use of **irace** is the automatic configuration of optimization and decision algorithms, that is, finding the most appropriate settings of an algorithm given a set of instances of a problem. However, it may also be useful for configuring other types of algorithms when performance depends on the used parameter settings. It builds upon the **race** package by Birattari and it is implemented in R. The **irace** package is available from CRAN. More information about **irace** is available at http://iridia.ulb.ac.be/irace.

1.2 Version

The current version of the **irace** package is 2.2. Previous versions of the package can be found in the CRAN website.

https://cran.r-project.org/web/packages/irace/

> Versions of **irace** before 2.0 are not compatible with the file formats detailed in this document.

The algorithm underlying the current version of **irace** and its motivation are described by López-Ibáñez et al. [5]. Details of the implementation before version 2.0 can be found in a previous technical report [4].

1.3 License

The **irace** package is Copyright © 2016 and distributed under the GNU General Public License version 3.0 (http://www.gnu.org/licenses/gpl-3.0.en.html). The **irace** package is free software (software libre): you can redistribute it and/or modify it under the terms of the GNU General Public License as published by the Free Software Foundation, either version 3 of the License, or (at your option) any later version.

The **irace** package is distributed in the hope that it will be useful, but WITHOUT ANY WARRANTY; without even the implied warranty of MERCHANTABILITY or FITNESS FOR A PARTICULAR PURPOSE.

Please be aware that the fact that this program is released as Free Software does not excuse you from scientific propriety, which obligates you to give appropriate credit! If you write a scientific paper describing research that made substantive use of this program, it is your obligation as a scientist to (a) mention the fashion in which this software was used in the Methods section; (b) mention the algorithm in the References section. The appropriate citation is:

> Manuel López-Ibáñez, Jérémie Dubois-Lacoste, Leslie Pérez Cáceres, Thomas Stützle, and Mauro Birattari. The **irace** package: Iterated Racing for Automatic Algorithm Configuration. *Operations Research Perspectives*, 3:43–58, 2016. doi: 10.1016/j.orp.2016.09.002

2 Before starting

The **irace** package provides an automatic configuration tool for tuning optimization algorithms, that is, automatically finding good configurations for the parameters values of a (target) algorithm saving the effort that normally requires manual tuning.

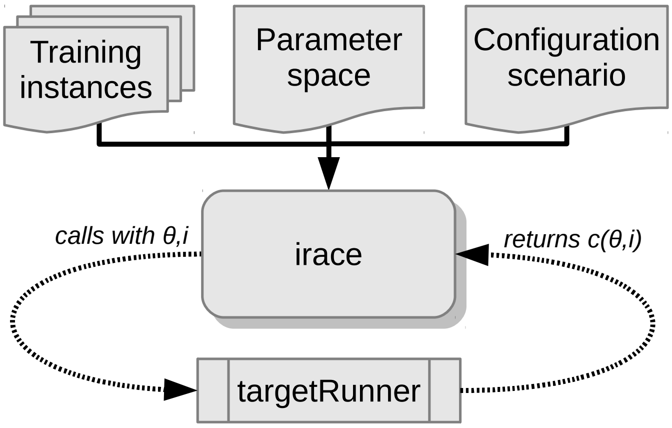

Figure 1: Scheme of **irace** flow of information.

Figure 1 gives a general scheme of how **irace** works. **Irace** receives as input a *parameter space definition* corresponding to the parameters of the target algorithm that will be tuned, a set of *instances* for which the parameters must be tuned for and a set of options for **irace** that define the *configuration scenario*. Then, **irace** searches in the parameter search space for good performing algorithm configurations by executing the target algorithm on different instances and with different parameter configurations. A `targetRunner` must be provided to execute the target algorithm with a specific parameter configuration (θ) and instance (i). The `targetRunner` function (or program) acts as an interface between the execution of the target algorithm and **irace**: It receives the instance and configuration as arguments and must return the evaluation of the execution of the target algorithm.

The following user guide contains guidelines for installing **irace**, defining configuration scenarios, and using **irace** to automatically configure your algorithms.

3 Installation

3.1 System requirements

- R (version $\geq$ 2.15) is required for running irace, but you don't need to know the R language to use it. R is freely available and you can download it from the R project website (`http://www.r-project.org`). See Appendix A for a quick installation guide of R.

- For GNU/Linux and OS X, the command-line executables `irace` and `parallel-irace` require GNU Bash. There is also a `irace.bat` for Windows. Individual examples may require additional software.

3.2 irace installation

The **irace** package can be installed automatically within R or by manual download and installation. We advise to use the automatic installation unless particular circumstances do not allow it. The instructions to install **irace** with the two mentioned methods are the following:

3.2.1 Install automatically within R

Execute the following line in the R console to install the package:

```
install.packages("irace")
```

Select a mirror close to your location, and test the installation in the R console with:

```
library("irace")
q() # To exit R
```

Alternatively, within the R graphical interface, you may use the `Packages and data->Package installer` menu on OS X or the `Packages` menu on Windows.

3.2.2 Manual download and installation

From the **irace** package CRAN website (https://cran.r-project.org/package=irace), download one of the three versions available depending on your operating system:

- `irace_2.2.tar.gz` (Unix/BSD/GNU/Linux)
- `irace_2.2.tgz` (OS X)
- `irace_2.2.zip` (Windows)

To install the package on GNU/Linux and OS X, you must execute the following command at the shell:

```
# Replace <package> with the path to the downloaded file.
R CMD INSTALL <package>
```

To install the package on Windows, open R and execute the following line on the R console:

```
# Replace <package> with the path to the downloaded file.
install.packages("<package>", repos = NULL)
```

If the previous installation instructions fail because of insufficient permissions and you do not have sufficient admin rights to install **irace** system-wide, then you need to force a local installation.

3.2.3 Local installation

Let's assume you wish to install **irace** on a path denoted by `<R_LIBS_USER>`, which is a filesystem path for which you have sufficient rights. This directory **must** exist before attempting the installation. Moreover, you must provide to R the path to this library when loading the package. However, the latter can be avoided by adding the path to the system variable `R_LIBS` or to the R internal variable `.libPaths`, as we will see below.[1]

On GNU/Linux or OS X, execute the following commands to install the package on a local directory:

[1] On Windows, see also https://cran.r-project.org/bin/windows/base/rw-FAQ.html#I-don_0027t-have-permission-to-write-to-the-R_002d3_002e3_002e1_005clibrary-directory.

```
export R_LIBS_USER="<R_LIBS_USER>"
# Create R_LIBS_USER if it doesn't exist
mkdir $R_LIBS_USER
# Replace <package> with the path to the downloaded file.
R CMD INSTALL --library=$R_LIBS_USER <package>
# Tell R where to find R_LIBS_USER
export R_LIBS=${R_LIBS_USER}:${R_LIBS}
```

On Windows, you can install the package on a local directory by executing the following lines in the R console:

```
# Replace <package> with the path to the downloaded file.
# Replace <R_LIBS_USER> with the path used for installation.
install.packages("<package>", repos = NULL, lib = "<R_LIBS_USER>")
# Tell R where to find R_LIBS_USER.
# This must be executed for every new session.
.libPaths(c("<R_LIBS_USER>", .libPaths()))
```

3.2.4 Testing the installation and invoking irace

Once **irace** has been installed, load the package and test that the installation was successful by opening an R console and executing:

```
# Load the package
library("irace")
# Obtain the installation path
system.file(package = "irace")
```

The last command must print out the filesystem path where **irace** is installed. In the remainder of this guide, the variable `$IRACE_HOME` is used to denote this path. When executing any provided command that includes the `$IRACE_HOME` variable do not forget to replace this variable with the installation path of **irace**.

On GNU/Linux or OS X, you can let the operating system know where to find **irace** by defining the `$IRACE_HOME` variable and adding it to the system `PATH`. Append the following commands to `~/.bash_profile`, `~/.bashrc` or `~/.profile`:

```
# Replace <IRACE_HOME> with the irace installation path
export IRACE_HOME=<IRACE_HOME>
export PATH=${IRACE_HOME}/bin/:$PATH
# Tell R where to find R_LIBS_USER
# Use the following line only if local installation was forced
export R_LIBS=${R_LIBS_USER}:${R_LIBS}
```

Then, open a new terminal and launch **irace** as follows:

```
irace --help
```

On Windows, you need to add both R and the installation path of **irace** to the environment variable `PATH`. To edit the `PATH`, search for "Environment variables" in the control panel, edit `PATH` and add a string similar to `C:\R_PATH\bin;C:\IRACE_HOME\bin` where `R_PATH` is the installation

path of R and IRACE_HOME is the installation path of **irace**. If **irace** was installed locally, you also need to edit the environment variable R_LIBS to add R_LIBS_USER. Then, open a new terminal (run program cmd.exe) and launch **irace** as:

```
irace.bat --help
```

Alternatively, you may directly invoke **irace** from within the R console by executing:

```
library("irace")
irace.cmdline("--help")
```

4 Running irace

Before performing the tuning of your algorithm, it is necessary to define a tuning scenario that will give **irace** all the necessary information to optimize the parameters of the algorithm. The tuning scenario is composed of the following elements:

1. Target algorithm parameter description (see Section 5.1).
2. Target algorithm runner (see Section 5.2).
3. Training instances list (see Section 5.4)
4. **irace** options (see Section 11).
5. *Optional:* Initial configurations (see Section 5.5).
6. *Optional:* Forbidden configurations (see Section 5.6).
7. *Optional:* Target algorithm evaluator (see Section 5.3).

These scenario elements can be provided as plain text files or as R objects. This user guide provides examples of both types, but we advise the use of plain text files, which we consider the simpler option.

For a step-by-step guide to create the scenario elements for your target algorithm continue to Section 4.1. For an example execution of **irace** using the **ACOTSP** scenario go to Section 4.2.

Once all the scenario elements are prepared you can execute **irace**, either using the command-line wrappers provided by the package or directly from the R console:

- Execute **irace** from the command-line as (on Windows, you should execute irace.bat):

  ```
  # $IRACE_HOME is the installation directory of irace.
  $IRACE_HOME/bin/irace --scenario scenario.txt
  ```

  For this example we assume that the needed scenario files have been set properly in the scenario.txt file using the options described in Section 11. Most **irace** options can be specified in the command line or directly in the scenario.txt file.

- Or execute **irace** from the R console as:

```r
library("irace")
parameters <- readParameters("parameters.txt")
scenario <- readScenario(filename = "scenario.txt",
                         scenario = defaultScenario())
irace(scenario = scenario, parameters = parameters)
```

The **irace** executable provides an option (`--check`) to check that the scenario is correctly defined. We recommend to perform a check every time you create a new scenario. When performing the check, **irace** will verify that the scenario and parameter definitions are correct and will test the execution of the target algorithm. To check your scenario execute the following commands:

- From the command-line (on Windows, execute `irace.bat`):

```
# $IRACE_HOME is the installation directory of irace.
$IRACE_HOME/bin/irace --scenario scenario.txt --check
```

- Or from the R console:

```r
library("irace")
parameters <- readParameters("parameters.txt")
scenario <- readScenario(filename = "scenario.txt",
                         scenario = defaultScenario())
checkIraceScenario(scenario = scenario, parameters = parameters)
```

4.1 Step-by-step setup guide

This section provides a guide to setup a basic execution of **irace**. The template files provided in the package (`$IRACE_HOME/templates`) will be used as basis for creating your new scenario. Please follow carefully the indications provided in each step and in the template files used; if you have doubts check the the sections that describe each option in detail.

1. Create a directory (e.g., `~/tuning/`) for the scenario setup. This directory will contain all the files that describe the scenario. On GNU/Linux or OS X, you can do this as follows:

```
mkdir ~/tuning
cd ~/tuning
```

2. Copy all the template files from the `$IRACE_HOME/templates/` directory to the scenario directory.

```
# $IRACE_HOME is the installation directory of irace.
cp $IRACE_HOME/templates/*.tmpl ~/tuning/
```

3. For each template in your tuning directory, remove the `.tmpl` suffix, and modify them following the next steps.

4. Define the target algorithm parameters to be tuned by following the instructions in `parameters.txt`. Available parameter types and other guidelines can be found in Section 5.1.

5. *Optional*: Define the initial parameter configuration(s) of your algorithm, which allows you to provide good starting configurations (if you know some) for the tuning. Follow the instructions in `configurations.txt` and set `configurationsFile="configurations.txt"` in `scenario.txt`. More information in Section 5.5. If you do not need to define initial configurations remove this file from the directory.

6. *Optional*: Define forbidden parameter value combinations, that is, configurations that **irace** must not consider in the tuning. Follow the instructions in `forbidden.txt` and update `scenario.txt` with `forbiddenFile = "forbidden.txt"`. More information about forbidden configurations in Section 5.6. If you do not need to define forbidden configurations remove this file from the directory.

7. Place the instances you would like to use for the tuning of your algorithm in the folder `~/tuning/Instances/`. In addition, you can create a file (e.g., `instances-list.txt`) that specifies which instances from that directory should be run and which instance-specific parameters to use. To use such an instance file, set the appropriate option in `scenario.txt`, e.g., `trainInstancesFile = "instances-list.txt"`. See Section 5.4 for guidelines.

8. Uncomment and assign in `scenario.txt` only the options for which you need a value different from the default. Some common parameters that you might want to adjust are:

   `execDir` (`--exec-dir`): the directory in which **irace** will execute the target algorithm; the default value is the current directory.

   `maxExperiments` (`--max-experiments`): the maximum number of executions of the target algorithm that **irace** will perform.

   `maxTime` (`--max-time`): the total maximum execution time of the target algorithm. Note that you must provide either `maxTime` or `maxExperiments`.

   For setting the tuning budget see Section 10.1. For more information on **irace** options and their default values, see Section 11.

9. Modify the `target-runner` script to run your algorithm. This script must execute your algorithm with the parameters and instance specified by **irace** and return the evaluation of the execution and *optionally* the execution time (`cost [time]`). When the `maxTime` option is used, returning `time` is mandatory. The `target-runner` template is written in GNU Bash scripting language, which can be executed easily in GNU/Linux and OS X systems. However, you may use any other programming language. As an example, we provide a Python example in the directory `$IRACE_HOME/examples/python`. Follow these instructions to adjust the given `target-runner` template to your algorithm:

   (a) Set the `EXE` variable with the path to the executable of the target algorithm.

   (b) Set the `FIXED_PARAMS` if you need extra arguments in the execution line of your algorithm. An example could be the time that your algorithm is required to run (`FIXED_PARAMS ="--time 60"`) or the number of evaluations required (`FIXED_PARAMS="--evaluations 10000"`).

   (c) The line provided in the template executes the executable described in the `EXE` variable.

   ```
   $EXE ${FIXED_PARAMS} -i ${INSTANCE} --seed ${SEED} ${CONFIG_PARAMS}
   ```

   You must change this line according to the way your algorithm is executed. In this example, the algorithm receives the instance to solve with the flag `-i` and the seed of

the random number generator with the flag `--seed`. The variable `CONFIG_PARAMS` adds to the command line the parameters that **irace** has given for the execution. You must set the command line execution as needed. For example, the instance might not need a flag and might need to be the first argument:

```
$EXE ${INSTANCE} ${FIXED_PARAMS} --seed ${SEED} ${CONFIG_PARAMS}
```

The output of your algorithm is saved to the file defined in the `$STDOUT` variable, and error output is saved in the file given by `$STDERR`. The line:

```
if [ -s "$STDOUT" ]; then
```

checks if the file containing the output of your algorithm is not empty. The example provided in the template assumes that your algorithm prints in the last output line the best result found (only a number). The line:

```
COST=$(cat ${STDOUT} | grep -e '^[[:space:]]*[+-]\?[0-9]' | cut -f1)
```

parses the output of your algorithm to obtain the result from the last line. The `target-runner` script must return **only** one number. In the template example, the result is returned with `echo "$COST"` (assuming `maxExperiments` is used) and the used files are deleted.

> The `target-runner` script must be executable.

You can test the target runner from the R console by checking the scenario as explained earlier in Section 4.

If you have problems related to the `target-runner` script when executing **irace**, see Appendix B for a check list to help diagnose common problems. For more information about the `targetRunner`, please see Section 5.2,

10. *Optional*: Modify the `target-evaluator` file. This is rarely needed and the `target-runner` template does not use it. Section 5.3 explains when a `targetEvaluator` is needed and how to define it.

Once the files have been prepared, you can execute **irace** using the command-line or directly from the R console:

- **On the console**, call the command:

```
cd ~/tuning/
$IRACE_HOME/bin/irace
```

- **On the R console**, open an R console and execute:

```r
library("irace")
# Go to the directory containing the scenario files
setwd("~/tuning")
# Create the R objects scenario and parameters
parameters <- readParameters("parameters.txt")
scenario <- readScenario(filename = "scenario.txt",
                         scenario = defaultScenario())
irace(scenario = scenario, parameters = parameters)
```

This will perform one run of **irace**. See the output of `irace --help` in the command-line or `irace.usage()` in R for quick information on additional **irace** parameters. For more information about **irace** options, see Section 11.

> Command-line options override the same options specified in the `scenario.txt` file.

4.2 Setup example for ACOTSP

The **ACOTSP** tuning example can be found in the package installation:

`$IRACE_HOME/examples/acotsp`

Additionally, a number of example scenarios can be found in the `examples` folder. More examples of tuning scenarios can be found in the Algorithm Configuration Library (AClib):

http://www.aclib.net/

In this section, we describe how to execute the **ACOTSP** scenario. If you wish to start setting up your own scenario, continue to the next section. For this example, we assume a GNU/Linux system but making the necessary changes in the commands and `targetRunner`, it can be executed in any system that has a C compiler. To execute this scenario follow the steps described in the following:

1. Create a directory for the tuning (e.g., `~/tuning/`) and copy the example scenario files located in the `examples` folder to the created directory:

```
mkdir ~/tuning
cd ~/tuning
# $IRACE_HOME is the installation directory of irace.
cp $IRACE_HOME/examples/acotsp/* ~/tuning/
```

2. Download the training instances from http://iridia.ulb.ac.be/irace/ to the `~/tuning/` directory.

3. Create the instance directory (e.g., `~/tuning/Instances`) and decompress the instance files on it.

```
mkdir ~/tuning/Instances/
cd ~/tuning/
tar -xvf tsp-instances-training.tar.bz2 Instances/
```

4. Download the **ACOTSP** software from http://www.aco-metaheuristic.org/aco-code/ to the `~/tuning/` directory and compile it.

```
cd ~/tuning/
tar -xvf ACOTSP-1.03.tgz
cd ~/tuning/ACOTSP-1.03
make
```

5. Create a directory for the executable and copy it:

```
mkdir ~/bin/
cp ~/tuning/ACOTSP-1.03/acotsp ~/bin/
```

6. Create a directory for executing the experiments and execute **irace**:

```
mkdir ~/tuning/acotsp-arena/
cd ~/tuning/
# $IRACE_HOME is the installation directory of irace.
$IRACE_HOME/bin/irace
```

7. Or you can also execute **irace** from the R console using:

```
library("irace")
setwd("~/tuning/")
parameters <- readParameters("parameters-acotsp.txt")
scenario <- readScenario(filename = "scenario.txt",
                         scenario = defaultScenario())
irace(scenario = scenario, parameters = parameters)
```

5 Defining a configuration scenario

5.1 Target algorithm parameters

The parameters of the target algorithm are defined by a parameter file as described in Section 5.1.4. Optionally, when executing **irace** from the R console, the parameters can be specified directly as an R object (see Section 5.1.5). For defining your parameters follow the guidelines provided in the following sections.

5.1.1 Parameter types

Each target parameter has an associated type that defines its domain and the way **irace** handles them internally. Understanding the nature of the domains of the target parameters is important to select appropriate types. The four basic types supported by **irace** are the following:

- *Real* parameters are numerical parameters that can take floating-point values within a given range. The range is specified as an interval '`(<lower bound>,<upper bound>)`'. This interval is closed, that is, the parameter value may eventually be one of the bounds. The possible values are rounded to a number of *decimal places* specified by option `digits`. For example, given the default number of digits of 4, the values 0.12345 and 0.12341 are both rounded to 0.1234.

- *Integer* parameters are numerical parameters that can take only integer values within the given range. The range is specified as for real parameters.

- *Categorical* parameters are defined by a set of possible values specified as '`(<value 1>, ..., <value n>)`'. The values are quoted or unquoted character strings. Empty strings and strings containing commas or spaces must be quoted.

- *Ordinal* parameters are defined by an *ordered* set of possible values in the same format as for categorical parameters. They are handled internally as integer parameters, where the integers correspond to the indexes of the values.

5.1.2 Parameter domains

For each target parameter, an interval or a set of values must be defined according to its type, as described above. There is no limit for the size of the set or the length of the interval, but keep in mind that larger domains could increase the difficulty of the tuning task. Choose always values that you consider relevant for the tuning. In case of doubt, we recommend to choose larger intervals, as occasionally best parameter settings may be not intuitive a priori. All intervals are considered as closed intervals.

It is possible to define parameters that will have always the same value. Such "*fixed*" parameters will not be tuned but their values are used when executing the target algorithm and they are affected by constraints defined on them. All fixed parameters must be defined as categorical parameters and have a domain of one element.

5.1.3 Conditional parameters

Conditional parameters are active only when others have certain values. These dependencies define a hierarchical relation between parameters. For example, the target algorithm may have a parameter `localsearch` that takes values `(sa,ts)` and another parameter `ts-length` that only needs to be set if the first parameter takes precisely the value `ts`. Thus, parameter `ts-length` is conditional on `localsearch == "ts"`.

5.1.4 Parameter file format

For simplicity, the description of the parameters space is given as a table. Each line of the table defines a configurable parameter

```
<name> <label> <type> <range> [ | <condition> ]
```

where each field is defined as follows:

<name> The name of the parameter as an unquoted alphanumeric string, e.g., '`ants`'.

<label> A *label* for this parameter. This is a string that will be passed together with the parameter to `targetRunner`. In the default `targetRunner` provided with the package (Section 5.2), this is the command-line switch used to pass the value of this parameter, for instance '`"--ants "`'.
The value of the parameter is concatenated *without separator* to the label when invoking `targetRunner`, thus *any whitespace in the label is significant*. Following the same example, when parameter `ants` takes value `5`, the default targetRunner will pass the parameter as `"--ants 5"`.

<type> The type of the parameter, either *integer*, *real*, *ordinal* or *categorical*, given as a single letter: '`i`', '`r`', '`o`' or '`c`'.

<range> The range or set of values of the parameter delimited by parentheses. e.g., `(0,1)` or `(a,b,c,d)`.

<condition> An optional *condition* that determines whether the parameter is enabled or disabled, thus making the parameter conditional. If the condition evaluates to false, then no value is assigned to this parameter, and neither the parameter value nor the corresponding label are passed to `targetRunner`. The condition must be a valid R logical expression[2]. The condition may contain the name of other parameters as long as the dependency graph does not contain any cycle. Otherwise, **irace** will detect the cycle and stop with an error.

As an example, Figure 2 shows the parameters file of the **ACOTSP** scenario.

```
# name       switch             type values               [conditions (using R syntax)]
algorithm    "--"               c    (as,mmas,eas,ras,acs)
localsearch  "--localsearch "   c    (0, 1, 2, 3)
alpha        "--alpha "         r    (0.00, 5.00)
beta         "--beta "          r    (0.00, 10.00)
rho          "--rho  "          r    (0.01, 1.00)
ants         "--ants "          i    (5, 100)
nnls         "--nnls "          i    (5, 50)              | localsearch %in% c(1, 2, 3)
q0           "--q0 "            r    (0.0, 1.0)           | algorithm == "acs"
dlb          "--dlb "           c    (0, 1)               | localsearch %in% c(1,2,3)
rasrank      "--rasranks "      i    (1, 100)             | algorithm == "ras"
elitistants  "--elitistants "   i    (1, 750)             | algorithm == "eas"
```

Figure 2: Parameter file (`parameters.txt`) for tuning **ACOTSP**.

5.1.5 Parameters R format

The target parameters are stored in an R `list` that you can obtain from the R console using the following command:

```
parameters <- readParameters(file = "parameters.txt")
```

See the help of the `readParameters` function (`?readParameters`) for more information. The structure of the parameter list that is created is as follows:

[2]For a quick list of R operators see: https://stat.ethz.ch/R-manual/R-devel/library/base/html/Syntax.html

`names`	Vector that contains the names of the parameters.
`types`	Vector that contains the type of each parameter 'i', 'c', 'r', 'o'.
`switches`	Vector that contains the labels of the parameters. e.g., switches to be used for the parameters on the command line.
`domain`	List of vectors, where each vector may contain two values (minimum, maximum) for real and integer parameters, or a set of values for categorical and ordinal parameters.
`conditions`	List of R logical expressions, with variables corresponding to parameter names.
`isFixed`	Logical vector that specifies which parameter is fixed and, thus, it does not need to be tuned.
`nbParameters`	An integer, the total number of parameters.
`nbFixed`	An integer, the number of parameters with a fixed value.
`nbVariable`	Number of variable (i.e., to be tuned) parameters.

The following example shows the structure of the `parameters` R object for the `algorithm`, `ants` and `q0` parameters of the **ACOTSP** scenario:

```
> print(parameters)

$names
[1] "algorithm" "ants"      "q0"

$types
algorithm      ants        q0
      "c"       "i"       "r"

$switches
algorithm      ants        q0
     "--" "--ants "   "--q0 "

$domain
$domain$algorithm
[1] "as"   "mmas" "eas"  "ras"  "acs"

$domain$ants
[1]   5 100

$domain$q0
[1] 0 1


$conditions
$conditions$algorithm
expression(TRUE)

$conditions$ants
expression(TRUE)
```

```
$conditions$q0
expression(algorithm %in% c("acs"))


$isFixed
algorithm      ants        q0
    FALSE     FALSE     FALSE

$nbParameters
[1] 3

$nbFixed
[1] 0

$nbVariable
[1] 3
```

5.2 Target algorithm runner

The execution of a candidate configuration on a single instance is done by means of a user-given auxiliary program or, alternatively, a user-given R function. The function (or program name) is specified by the option `targetRunner`. The `targetRunner` must return the evaluation of the execution unless a post-execution evaluation (e.g., multi-objective evaluation) is required, see Section 5.3 for details.

> The objective of **irace** is to minimize the obtained evaluations. If you wish to maximize, you can multiply the evaluations by `-1` before returning them to **irace**.

5.2.1 Target runner executable program

When `targetRunner` is an auxiliary executable program, it is invoked for each candidate configuration, passing as arguments:

```
<id.configuration> <id.instance> <seed> <instance> [<extra.params>] <configuration>
```

- `id.configuration` an alphanumeric string that uniquely identifies a configuration;
- `id.instance` an alphanumeric string that uniquely identifies a pair `(instance, seed)`;
- `seed` seed for the random number generator to be used for this evaluation, ignore the seed for deterministic algorithms;
- `instance` string giving the instance to be used for this evaluation;
- `extra.params` user-defined parameters associated to the instance;
- `configuration` the pairs parameter label-value that describe this candidate configuration. Typically given as command-line switches to be passed to the executable program.

The experiment list shown in Section 5.2.2, would result in the following execution line:

```
target-runner 1 113 734718556 /home/user/instances/tsp/2000-533.tsp \
 --eas --localsearch 0 --alpha 2.92 --beta 3.06 --rho 0.6 --ants 80
```

The command line switches that describe the candidate configuration are constructed by appending to each parameter label (switch), *without separator*, the value of the parameter, following the order given in the parameter table. The program `targetRunner` must print a real number, which corresponds to the cost measure of the candidate configuration for the given instance and optionally its execution time (mandatory when `maxTime` is used). The working directory of `targetRunner` is set to the execution directory specified by the option `execDir`. This allows the user to execute independent runs of **irace** in parallel using different values for `execDir`, without the runs interfering with each other.

5.2.2 Target runner R function

When `targetRunner` is an R function, it is invoked for each candidate configuration as:

```
targetRunner(experiment, scenario)
```

where `experiment` is a list that contains information about configuration and instance to execute one experiment, and `scenario` is the scenario list. The structure of the `experiment` list is as follows:

- `id.configuration` an alphanumeric string that uniquely identifies a configuration;
- `id.instance` an alphanumeric string that uniquely identifies a pair `(instance, seed)`;
- `seed` seed to be used for this evaluation;
- `instance` string giving the instance to be used for this evaluation;
- `extra.params` user-defined parameters associated to the instance;
- `configuration` 1-row data frame with a column per parameter name;
- `switches` vector of parameter switches (labels) in the order of parameters used in `configuration`.

The following is an example of an experiment list for the **ACOTSP** scenario:

```
> print(experiment)

$id.configuration
[1] 1

$id.instance
[1] 113

$seed
[1] 734718556

$configuration
  algorithm localsearch alpha beta rho ants nnls q0  dlb
1       eas           0  2.92 3.06 0.6   80   NA NA <NA>
  rasrank elitistants
```

```
1      NA         588

$instance
[1] "/home/user/instances/tsp/2000-533.tsp"

$extra.params
NULL

$switches
       algorithm      localsearch            alpha
            "--" "--localsearch "       "--alpha "
            beta              rho             ants
       "--beta "         "--rho  "         "--ants "
            nnls               q0              dlb
       "--nnls "           "--q0 "          "--dlb "
         rasrank      elitistants
   "--rasranks " "--elitistants "
```

If targetEvaluator is NULL, then the targetRunner function must return a list with at least one element "cost", the numerical value corresponding to the evaluation of the given configuration on the given instance.

If the scenario option maxTime is non-zero, then the list must contain at least another element "time" that reports the execution time for this call to targetRunner.

The return list may also contain the following optional elements that are used by **irace** for reporting errors in targetRunner:

- `error` is a string used to report an error;
- `outputRaw` is a string used to report the raw output of calls to an external program or function;
- `call` is a string used to report how targetRunner called an external program or function;

5.3 Target evaluator

Normally, targetRunner returns the cost of the execution of a candidate configuration (see Section 5.2). However, there are cases when the cost evaluation must be delayed until all candidate configurations in a race have been executed on a instance.

The targetEvaluator option defines an auxiliary program (or an R function) that allows postponing the evaluations of the candidate configurations. For each instance seen, the program targetEvaluator is only invoked after all the calls to targetRunner for all alive candidate configurations on the same instance have already finished.

> When using targetEvaluator, targetRunner must not return the evaluation of the configuration. If maxTime is used, targetRunner must return only execution time.

As an example, targetEvaluator may be used to dynamically find normalization bounds for the output returned by an algorithm for each individual instance. In this case, targetRunner will save the output of the algorithm, then the first call to targetEvaluator will examine the output produced by all calls to targetRunner for the same instance, update the normalization

bounds and return the normalized output. Subsequent calls to targetEvaluator for the same instance will simply return the normalized output.

A similar need arises when using quality measures for multi-objective optimization algorithms, such as the hypervolume, which typically require specifying reference points or sets. By using targetEvaluator, it is possible to dynamically compute the reference points or sets while **irace** is running. Examples are provided at `examples/hypervolume`. See also Section 10.2 for more information on how to tune multi-objective algorithms.

5.3.1 Target evaluator executable program

When targetEvaluator is an auxiliary executable program, it is invoked for each candidate with the following arguments:

```
<id.configuration> <id.instance> <seed> <instance> <num.configs> <all.conf.id>
```

`id.configuration`	an alphanumeric string that uniquely identifies a configuration;
`id.instance`	an alphanumeric string that uniquely identifies a pair (`instance, seed`);
`seed`	seed to be used for this evaluation;
`instance`	string giving the instance to be used for this evaluation;
`num.configs`	number of alive candidate configurations;
`all.conf.id`	list of IDs of the alive configurations separated by whitespace.

The targetEvaluator executable must print a numerical value corresponding to the cost measure of the candidate configuration on the given instance.

5.3.2 Target evaluator R function

When targetEvaluator is an R function, it is invoked for each candidate configuration as:

```
targetEvaluator(experiment, num.configurations, all.conf.id,
                scenario, target.runner.call)
```

where `experiment` is a list that contains information about one experiment (see Section 5.2.2), `num.configurations` is the number of configurations alive in the race, `all.conf.id` is the vector of IDs of the alive configurations, `scenario` is the scenario list and `target.tunner.call` is the string of the targetRunner execution line.

The function targetEvaluator must return a list with one element `"cost"`, the numerical value corresponding to the cost measure of the given configuration on the given instance.

The return list may also contain the following optional elements that are used by **irace** for reporting errors in `targetEvaluator`:

`error`	is a string used to report an error;
`outputRaw`	is a string used to report the raw output of calls to an external program or function;
`call`	is a string used to report how `targetEvaluator` called an external program or function;

5.4 Training instances

The **irace** options `trainInstancesDir` and `trainInstancesFile` specify where to find the training instances.

By default, the value of `trainInstancesFile` is empty. This means that **irace** will consider all files within the directory given by `trainInstancesDir` (by default `./Instances`) as training instances.

Otherwise, the value of `trainInstancesFile` may specify a text file. The format of this file is one instance per line, and the first alphanumeric string of each line corresponds to the instance filename. The remainder text within each line are considered as extra parameters to be supplied to `targetRunner` for this specific instance. The following example shows a training instance file for the **ACOTSP** scenario:

```
# Example training instances file
100/100-1_100-2.tsp --time 1
100/100-1_100-3.tsp --time 2
100/100-1_100-4.tsp --time 3
```

Figure 3: Training instances file for tuning **ACOTSP**.

The value of `trainInstancesDir` is always prefixed to the instance name, that is, the instances names are treated as relative to this directory. For example, given the above file as `trainInstancesFile` and the default value of `trainInstancesDir` (`./Instances`), then a possible invocation of `targetRunner` would be:

```
target-runner 1 113 734718 ./Instances/100/100-1_100-2.tsp --time 1 \
             --alpha 2.92 ...
```

Training instances do not need to be files, **irace** just passes their names to `targetRunner`, thus the names can denote benchmark functions or descriptive labels that the target algorithm understands. The extra instance parameters could actually be the definition of the instance. In that case, `trainInstancesDir` is usually set to the empty string (`--train-instances-dir=""`). For example,

```
# Example training instances file
rosenbrock_20 --function=12 --nvar 20
rosenbrock_30 --function=12 --nvar 30
rastrigin_20 --function=15 --nvar 20
rastrigin_30 --function=15 --nvar 30
```

Optionally, when executing **irace** from the R console, the list of instances and their specific parameters might be provided explicitly by means of the variables `scenario$instances` and `scenario$instances.extra.params`, respectively. Thus, the previous example would be equivalent to:

```
scenario$instances <- c("rosenbrock_20", "rosenbrock_40",
                        "rastrigin_20", "rastrigin_40")
scenario$instances.extra.params <-
  c("--function=12 --nvar 20", "--function=12 --nvar 30",
    "--function=15 --nvar 20", "--function=15 --nvar 30")
```

By default, **irace** assumes that the target algorithm is stochastic (the value of the option `deterministic` is 0), thus, the same configuration can be executed more than once on the same instance and obtain different results. In this case, **irace** generates pairs `(instance,seed)` by generating a random seed for each instance. In other words, configurations evaluated on the same instance use the same random seed. This is a well-known variance reduction technique called *common random numbers* [6]. If all available pairs are used within a run of **irace**, new pairs are generated with different seeds, that is, a configuration evaluated more than once per instance will use different random seeds.

If `deterministic` is set to 1, then each instance will be used at most once per race. This setting should only be used for target algorithms that do not have a stochastic behavior and, therefore, executing the target algorithm on the same instance several times with different seeds does not make sense.

> If `deterministic` is active and the number of training instances provided to **irace** is less than `firstTest` (default: 5), no statistical test will be performed on the race.

Finally, **irace** randomly re-orders the sequence of instances provided. This random sampling may be disabled by using the option `sampleInstances` (`--sample-instances 0`) if keeping the order provided in the instance file is important.

> We advise to always sample instances to prevent biasing the tuning due to the instance order. See also Section 10.4

5.5 Initial configurations

The scenario option `configurationsFile` allows specifying a text file that contains an initial set of configurations to start the execution of **irace**. If the number of initial configurations supplied in the file is less than the number of configurations required by **irace** in the first iteration, additional configurations will be sampled uniformly at random.

The format of the configurations file is one configuration per line, and one parameter value per column. The first line must give the parameter name corresponding to each column (names must match those given in the parameters file). Each configuration must satisfy the parameter conditions (`NA` should be used for those parameters that are not enabled for a given configuration) and not be forbidden by the constraints that define forbidden configurations (Section 5.6), if any.

Figure 4 gives an example file that corresponds to the **ACOTSP** scenario.

```
## Initial candidate configuration for irace
algorithm localsearch alpha beta rho  ants nnls dlb q0 rasrank elitistants
as        0           1.0   1.0  0.95 10   NA   NA  0  NA      NA
```

Figure 4: Initial configuration file (`default.txt`) for tuning **ACOTSP**.

We advise to use this feature when a default configuration of the target algorithm exists or when different sets of good parameter values are known. This will allow **irace** to start the search from those parameter values and attempt to improve their performance.

5.6 Forbidden configurations

The scenario option `forbiddenFile` specifies a text file containing logical expressions of parameter values that valid configurations should not satisfy, that is, no configuration that satisfies any of these logical expressions will be evaluated by **irace**. This is useful when some combination

of parameter values could cause the target algorithm to crash, consume excessive CPU time or memory, or when it is known that they do no produce satisfactory results.

The format of the forbidden configurations file is one constraint per line, where each constraint is a logical expression (in R syntax) containing parameter names as defined by the `parameterFile` (Section 5.1), values and logical operators. For a quick list of R logical operators see:

https://stat.ethz.ch/R-manual/R-devel/library/base/html/Syntax.html

If a parameter configuration is generated that makes any of the logical expressions evaluate to `TRUE`, then the configuration is considered forbidden and it is discarded. Figure 5 shows an example file that corresponds to the **ACOTSP** scenario.

```
## Examples of valid logical operators are:
## ==  !=  >=  <=  >  <  &  |  !  %in%
(alpha == 0.0) & (beta == 0.0)
```

Figure 5: Forbidden configurations file (`forbidden.txt`) for tuning **ACOTSP**.

If initial configuration are provided (Section 5.5), they must also comply with the constraints defined in `forbiddenFile`.

6 Parallelization

A single run of **irace** can be done much faster by executing the calls to `targetRunner` (the runs of the target algorithm) in parallel. There are four ways to parallelize a single run of **irace**:

- **Parallel processes**: The option `parallel` allows executing in parallel, within a single computer, the calls to `targetRunner`, by means of the **parallel** R package. For example, adding `--parallel N` to the command line of **irace** will launch in parallel up to N calls of the target algorithm.

- **MPI**: By enabling the option `mpi`, calls to `targetRunner` will be executed in parallel by using the message passing interface (MPI) protocol (requires the **Rmpi** R package). In this case, the option `parallel` controls the number of slave nodes used by **irace**. For example, adding `--mpi 1 --parallel N` to the command-line will create N slaves + 1 master, and execute up to N calls of `targetRunner` in parallel.

  The user is responsible for setting up the required MPI environment. MPI is commonly available in computing clusters and requires launching **irace** in some particular way. An example script for using MPI mode in a SGE cluster is given at `$IRACE_HOME/examples/mpi/`.

- **SGE cluster**: This mode uses the commands `qsub` and `qstat` often found in Sun Grid Engine (SGE) and compatible clusters. The command `qsub` must return a message that contains the string: `"Your job JOBID"`, where `JOBID` is a unique identifier for the job submitted. The command `qstat -j JOBID` must return nonzero if `JOBID` has finished its execution, and zero otherwise.

  Enabling the option `sgeCluster` (`--sge-cluster 1`), **irace** will launch in parallel as many calls of `targetRunner` as possible and use `qstat` to wait for cluster jobs.

In this mode, **irace** must run in the submission node of the cluster, and hence, `qsub` should not be used to invoke **irace** itself. The user must call `qsub` from within `targetRunner` with the appropriate settings for their cluster, otherwise `targetRunner` will not submit jobs to the cluster. The use of a separate `targetEvaluator` script is required to evaluate the results of `targetRunner` and return them to **irace**.

See the examples in `$IRACE_HOME/examples/sge-cluster/`.

- `targetRunnerParallel`: This option allows users to fully control the parallelization of the execution of `targetRunner`. Its value must be an R function that will be invoked by **irace** as follows:

```
  targetRunnerParallel(experiments, exec.target.runner, scenario)
```

where `experiments` is a list that contains elements with information about configurations and instances to be executed (see Section 5.2 for a description), `exec.target.runner` is the function within **irace** that takes care of executing `targetRunner`, check its output and, possibly, retry in case of error (`targetRunnerRetries`) and `scenario` is the scenario list. The `targetRunnerParallel` function may call the given `exec.target.runner` for each element in the `experiments` list. A trivial example would be:

```
targetRunnerParallel <- function(experiments, exec.target.runner, scenario)
{
  return (lapply(experiments, exec.target.runner, scenario = scenario))
}
```

However, the user is free to set up the calls in any way, perhaps implementing its own replacement for `exec.target.runner`.

The only requirement is that the `targetRunnerParallel` function must return a list of the same length as `experiments`, where each element is the output expected from the corresponding call to `targetRunner` (see Section 5.2). The following is an example of the output of a call to `targetRunnerParallel` with 2 experiments, in which the execution time is not reported:

```
   print(output)

## [[1]]
## [[1]]$cost
## [1] 38546312
##
## [[1]]$time
## [1] NA
##
##
## [[2]]
## [[2]]$cost
## [1] 39347203
##
## [[2]]$time
## [1] NA
```

7 Testing of configurations

Once the tuning process is finished, **irace** commonly returns a set of configurations corresponding to the elite configurations at the end of the run, ordered from best to worst. To further investigate the cost of these configurations, **irace** offers the possibility of evaluating these configurations on a test instance set, typically different from the training set used during the tuning phase. These evaluations will use the same settings for parallel execution, `targetRunner` and `targetEvaluator`.

The test instance set can be specified by the options `testInstancesDir` and `testInstancesFile`, or by setting directly the variable `scenario$testInstances`, which behave the same as their counterparts for the training instances (Section 5.4). In particular, each test instance is assigned a different seed in the same way as done for the training instances. In principle, **irace** evaluates each configuration on each testing instance just once, because evaluating one run on n instances is always better than evaluating n' runs on n/n' instances [1]. However, if the number of instances is limited, one can always duplicate instances as needed in the `testInstancesFile`, and **irace** will assign a different random seed to each instance.

The options `testNbElites` and `testIterationElites` control which configurations are evaluated during the testing phase. In particular, setting `testIterationElites = 1` will test not only the final set of elite configurations (those returned at the end of the training phase), but also the set of elites at the end of each race (iteration). The option `testNbElites` limits the maximum number of configurations considered within each set. Some examples:

- `testIterationElites = 0; testNbElites = 1` means that only the best configuration found during the run of **irace**, the final best, will be used in the testing phase.

- `testIterationElites = 1; testNbElites = 1` will test, in addition to the final best, the best configuration found at each iteration.

- `testIterationElites = 1; testNbElites = 2` will test the two best configurations found at each iteration, in addition to the final best and second-best configurations.

The testing can be also (re-)executed at a later time by using the following R command:

```
testing.main(logFile = "./irace.Rdata")
```

This line will load the **irace** results found in the generated `logFile` file to perform the testing. The testing results will be saved in the **irace** log file specified in `scenario$logFile` in the `iraceResults$testing` R object. The structure of the object is described in Section 9.2. For examples on how to analyse the data see Section 9.3.

Another alternative is to test a specific set of configurations using the command-line option `--only-test` as follows:

```
irace --only-test configurations.txt
```

where `configurations.txt` has the same format as the set of initial configurations (Section 5.5).

8 Recovering irace runs

Problems like power cuts, hardware malfunction or the need to use computational power for other tasks may occur during the execution of **irace**, terminating a run before completion. At the end of each iteration, **irace** saves an R data file (`logFile`, by default `"./irace.Rdata"`) that not

only contains information about the tuning progress (Section 9.2), but also internal information that allows recovering an incomplete execution.

To recover an incomplete **irace** run, set the option `recoveryFile` to the log file previously produced, and **irace** will continue the execution from the last saved iteration. The state of the random generator is saved and loaded, therefore, as long as the execution is continued in the same machine, the obtained results will be exactly the same as executing **irace** in one step (external factors, such as CPU load and disk caches, may affect the target algorithm and that may affect the results). You can specify the `recoveryFile` from the command-line or from the scenario file, and execute **irace** as described in Section 4. For example, from the command-line use:

```
irace --recovery-file "./irace-backup.Rdata"
```

> When recovering a previous run, **irace** will try to save data on the file specified by the `logFile` option. Thus, you must specify different files for `logFile` and `recoveryFile`. Before recovering, we strongly advise to rename the saved R data file as in the example above, which uses `"irace-backup.Rdata"`.

> Do not change anything in the log file or the scenario file before recovering, as it may have unexpected effects on the recovered run of **irace**. In case of doubt, please contact us first (Section 13).

9 Output and results

During its execution, **irace** prints information about the progress of the tuning in the standard output. Additionally, after each iteration, an R data file is saved (`logFile` option) containing the state of **irace**.

9.1 Text output

Figure 6 shows the output, up to the end of the first iteration, of a run of elitist **irace** applied to the **ACOTSP** scenario with 1000 evaluations as budget.

First, **irace** gives the user a warning informing that it has found a file with the default scenario filename and it will use it. Then, general information about the selected **irace** options is printed:

- `nbIterations` indicates the minimum number of iterations **irace** has calculated for the scenario. Depending on the development of the tuning the final iterations that are executed can be more.
- `minNbSurvival` indicates the minimum number of alive configurations that are required to continue a race. When less configurations are alive the race is stopped and a new iteration begins.
- `nbParameters` is the number of parameters of the scenario.
- `seed` is the number that was used to initialize the random number generator in **irace**.
- `confidence level` is the confidence level of the statistical test.
- `budget` is the total number of evaluations available for the tuning.
- `time budget` is the maximum execution time available for the tuning.

- `mu` is a value used for calculating the minimum number of iterations.
- `deterministic` indicates if the target algorithm is assumed to be deterministic.

```
*******************************************************************************
Warning: A default scenario file './scenario.txt' has been found and will be read
# 2016-11-04 19:05:11 CET: Elitist race
# Elitist new instances: 1
# Elitist limit: 2

# 2016-11-04 19:05:11 CET: Initialization
# nbIterations: 5
# minNbSurvival: 5
# nbParameters: 11
# seed: 123
# confidence level: 0.95
# budget: 1000
# mu: 5
# deterministic: FALSE

# 2016-11-04 19:05:11 CET: Iteration 1 of 5
# experimentsUsedSoFar: 0
# remainingBudget: 1000
# currentBudget: 200
# nbConfigurations: 33
  Markers:
     x No test is performed.
     - The test is performed and some configurations are discarded.
     = The test is performed but no configuration is discarded.
     ! The test is performed and configurations could be discarded but elite configurations are preserved.

+-+-----------+------+-----+------------+-----------+--------+-----+----+------+
| |   Instance| Alive| Best|   Mean best| Exp so far|  W time|  rho|KenW|  Qvar|
+-+-----------+------+-----+------------+-----------+--------+-----+----+------+
|x|          1|    33|   28| 23166743.00|         33|00:00:30|   NA|  NA|    NA|
|x|          2|    33|   28| 25845047.50|         66|00:00:31|+0.97|0.99|0.0053|
|x|          3|    33|   23| 24976880.00|         99|00:00:30|+0.97|0.98|0.0078|
|x|          4|    33|   28| 25882919.25|        132|00:00:31|+0.98|0.98|0.0067|
|-|          5|     2|   23| 26349755.40|        165|00:00:31|-0.20|0.04|0.6000|
+-+-----------+------+-----+------------+-----------+--------+-----+----+------+
Best configuration:          23    mean value:     26349755.40
Description of the best configuration:
   .ID. algorithm localsearch  alpha  beta    rho ants nnls     q0 dlb rasrank elitistants .PARENT.
23   23       acs           3 4.2705 3.679 0.8752   19   17 0.6667   1      NA          NA       NA


# 2016-11-04 19:07:47 CET: Elite configurations:
   algorithm localsearch  alpha   beta    rho ants nnls     q0 dlb rasrank elitistants
23       acs           3 4.2705 3.6790 0.8752   19   17 0.6667   1      NA          NA
28       acs           3 3.8770 3.7682 0.0517   39   17 0.8505   0      NA          NA
```

Figure 6: Sample text output of **irace**.

At each iteration, information about the progress of the execution is printed as follows:

- `experimentsUsedSoFar` is the number of experiments from the total budget that have been used up to the current iteration.

- `timeUsed` is the execution time used so far in the experiments. Only available when reported in the `targetRunner` (activate it with the `maxTime` option).
- `remainingBudget` is the number of experiments that have not been used yet.
- `timeEstimate` estimation of the mean execution time. This is used to calculate the remaining budget when `maxTime` is used.
- `currentBudget` is the number of evaluations **irace** has allocated to the current iteration.
- `nbConfigurations` is the number of configurations **irace** will use in the current iteration. In the first iteration, this number of configurations include the initial configurations provided; in later iterations, it includes the elite configurations from the previous iterations.

After the iteration information, a table shows the progress of the iteration execution. Each row of the table gives information about the execution of an instance in the race. The first column contains a symbol that describes the results of the statistical test:

`|x|` No statistical test was performed for this instance. The options `firstTest` and `eachTest` control on which instances the statistical test is performed.

`|-|` Statistical test performed and configurations have been discarded. The column `Alive` gives an indication of how many configurations have been discarded.

`|=|` Statistical test performed and no configurations have been discarded. This means **irace** needs more information to identify the best configurations.

`|!|` This indicator exists only for the elitist version of **irace**. It indicates that the statistical test was performed and some elite configurations show bad performance and could be discarded but they are kept because of the elitist rules. See option `elitist` in Section 11 for more information.

The `instance` column gives the number of `(instance,seed)` pair executed. This number corresponds to the index of the list found in `state$.irace$instancesList`. See Section 9.2 for more information.

The `Alive` column gives the number of configurations that have not been discarded after the statistical test was performed. The column `Best` gives the ID of the best configuration according to the instances seen so far in this race (i.e., not including previous iterations). The `Mean best` column gives the mean of the best configuration across the instances seen so far in this race. The `Exp so far` gives the number of experiments performed so far. The `W time` column gives the wall-clock time spent on that instance.

The columns `rho`, `KenW`, and `Qvar` give the values of Spearman's rank correlation coefficient rho, Kendall's concordance coefficient W, and a variance measure described in [7], respectively, of the configurations across the instances executed so far in this race. Use `rho`, `KenW` and `Qvar` to analyze how consistent is the performance of the configurations across the instances. Note that these values are only valid for the instances that were already executed in the iteration. Values close to 1 for `rho` and `KenW` and values close to 0 for `Qvar` indicate that the scenario is highly homogeneous. For heterogeneous scenarios, we provide advice in Section 10.4.

Finally, **irace** outputs the best configuration found and a list of the elite configurations. The elite configurations are configurations that did not show statistically significant difference during the race; they are ordered according to their mean performance on the executed instances.

9.2 Data file output

The R data file created by **irace** (logFile) contains an object called `iraceResults`. You can load this data in the R console by:

```
load("irace-output.Rdata")
```

The `iraceResults` object is a list, and the elements of a list can be accessed in R by using the `$` or `[[]]` operators:

```
> iraceResults$irace.version

[1] "2.2.1689:1690M"

> iraceResults[["irace.version"]]

[1] "2.2.1689:1690M"
```

The `iraceResults` list contains the following elements:

- `scenario`: The scenario R object containing the **irace** options used for the execution. See Section 11 and the help of the **irace** package; open an R console and type: `?defaultScenario`. See Section 11 for more information.

- `parameters`: The parameters R object containing the description of the target algorithm parameters. See Section 5.1.

- `allConfigurations`: The target algorithm configurations generated by **irace**. This object is a `data frame`, each row is a candidate configuration; the first column (`.ID.`) indicates the internal identifier of the configuration; the final column (`.PARENT.`) is the identifier of the configuration from which the current configuration was sampled; and the remaining columns correspond to the parameter values; each column is named as the parameter name specified in the parameter object.

```
> head(iraceResults$allConfigurations)

  .ID. algorithm localsearch  alpha   beta    rho ants nnls
1    1        as           3 2.8815 3.9545 0.4553   72    8
2    2       ras           1 4.1578 2.1517 0.5030   31   13
3    3       eas           0 2.7373 6.4424 0.6003   35   NA
4    4       ras           1 1.9410 1.6048 0.8639   96   30
5    5      mmas           2 0.5409 4.8703 0.1085   20   18
6    6       ras           0 4.3323 7.0857 0.7628   19   NA
  q0  dlb rasrank elitistants .PARENT.
1 NA    0      NA          NA       NA
2 NA    1      33          NA       NA
3 NA <NA>      NA         669       NA
4 NA    0     100          NA       NA
5 NA    1      NA          NA       NA
6 NA <NA>      36          NA       NA
```

- `allElites`: A list that contains one element per iteration. Each element contains the internal identifier of the elite candidate configurations of the corresponding iteration (identifiers correspond to `allConfigurations$.ID.`).

```
> print(iraceResults$allElites)

[[1]]
[1] 23 28

[[2]]
[1] 51 41 28 23

[[3]]
[1] 65 51 81 71

[[4]]
[1]  65 102 109

[[5]]
[1]  65 140 136 138 142

[[6]]
[1]  65 136 140 138 146

[[7]]
[1]  65 140 136 138 146
```

The configurations are ordered by mean performance, that is, the ID of the best configuration corresponds to the first ID. To obtain the values of the parameters of all elite configurations found by **irace** use:

```
> getFinalElites(irace.logFile = "irace-output.Rdata", n = 0)

  .ID. algorithm localsearch  alpha   beta    rho ants nnls
1   65       acs           3 1.6204 2.3432   0.65   40   10
2  140       acs           3 1.5916  4.581 0.6623   42   12
3  136       acs           3 4.7387 2.6375 0.4605   66   19
4  138       acs           3  1.657 3.7568 0.6338   39    7
5  146       acs           3 2.9621 3.0738 0.6904   56   16
      q0 dlb rasrank elitistants .PARENT.
1 0.0532   1      NA          NA       51
2 0.0454   1      NA          NA       65
3 0.8355   1      NA          NA      102
4 0.0325   1      NA          NA       65
5 0.0275   1      NA          NA       65
```

- `iterationElites`: A vector containing the best candidate configuration ID of each iteration. The best configuration found corresponds to the last one of this vector.

```
> print(iraceResults$iterationElites)

[1] 23 51 65 65 65 65 65
```

One can obtain the full configuration with:

```
> last <- length(iraceResults$iterationElites)
> id <- iraceResults$iterationElites[last]
> getConfigurationById(irace.logFile = "irace-output.Rdata", ids = id)

   .ID. algorithm localsearch  alpha   beta  rho ants nnls
65   65       acs           3 1.6204 2.3432 0.65   40   10
       q0 dlb rasrank elitistants .PARENT.
65 0.0532   1      NA          NA       51
```

- `experiments`: A matrix with configurations as columns and instances as rows. Column names correspond to the internal identifier of the configuration (`allConfigurations$.ID.`). The results of a particular configuration can be obtained using:

```
> # As an example, we use the best configuration found
> best.config <- getFinalElites(iraceResults = iraceResults, n = 1)
> id <- best.config$.ID.
> # Obtain the configurations using the identifier
> # of the best configuration
> all.exp <- iraceResults$experiments[,as.character(id)]
> all.exp[!is.na(all.exp)]

       1        2        3        4        5        6
23092770 28408585 23215699 28532010 28196078 23374947
       7        8        9       10       11       12
28440962 28393330 23131730 23273042 28425945 23264980
      13       14       15       16
28078660 28420396 23411677 28479495
```

When a configuration was not executed on an instance, its value is `NA`. A configuration may not be executed on an instance because: 1) it was not created yet when the instance was used, or 2) it was discarded by the statistical test and not executed on subsequent instances, or 3) the race terminated before this instance was considered.

Row names correspond to the identifier of the `(instance,seed)` pairs defined in `state$.irace$instancesList`. The instance and seed used for a particular experiment can be obtained with:

```
> # As an example, we get seed and instance of the experiments
> # of the best candidate.
> # Get index of the instances
> pair.id <- names(all.exp[!is.na(all.exp)])
> index <- iraceResults$state$.irace$instancesList[pair.id,"instance"]
```

```
> # Obtain the instance names
> iraceResults$scenario$instances[index]

 [1] "1000-3.tsp" "1500-3.tsp" "1000-4.tsp" "1500-2.tsp"
 [5] "1500-1.tsp" "1000-1.tsp" "1500-5.tsp" "1500-4.tsp"
 [9] "1000-2.tsp" "1000-5.tsp" "1500-5.tsp" "1000-5.tsp"
[13] "1500-1.tsp" "1500-4.tsp" "1000-1.tsp" "1500-2.tsp"

> # Get the seeds
> iraceResults$state$.irace$instancesList[index,"seed"]

 [1] 1291423063  618989142 1106017917  781881326 1890315053
 [6]  512660239  369736010  366457852 2066650079  864519670
[11]  369736010  864519670 1890315053  366457852  512660239
[16]  781881326
```

- `experimentLog`: A matrix with columns `iteration,instance,configuration`. This matrix contains the log of all the experiments that **irace** performs during its execution. The `instance` column refers to the index of the `state$.irace$instancesList` data frame.
- `softRestart`: A logical vector that indicates if a soft restart was performed on each iteration. If `FALSE`, then no soft restart was performed. See option `softRestart` in Section 11.
- `state`: A list that contains the state of **irace**, the recovery (Section 8) is done using the information contained in this object. The probabilistic model of the last elite configurations can be found here by doing:

```
> # As an example, we get the model probabilities for the
> # localsearch parameter.
> iraceResults$state$model["localsearch"]

$localsearch
$localsearch$`65`
[1] 0.0003406192 0.0003406192 0.0003406192 0.9989781425

$localsearch$`136`
[1] 0.0003406192 0.0003406192 0.0003406192 0.9989781425

$localsearch$`140`
[1] 0.0003406192 0.0003406192 0.0003406192 0.9989781425

$localsearch$`138`
[1] 0.0003406192 0.0003406192 0.0003406192 0.9989781425

$localsearch$`146`
[1] 0.0003406192 0.0003406192 0.0003406192 0.9989781425

> # The order of the probabilities corresponds to:
> iraceResults$parameters$domain$localsearch
```

```
[1] "0" "1" "2" "3"
```

The example shows a list that has one element per elite configuration (ID as element name). In this case, `localsearch` is a categorical parameter and it has a probability for each of its values.

- `testing`: A list that contains the testing results. The list contains the following elements:
  - `experiments`: Matrix of experiments in the same format as the `iraceResults$experiments` matrix. The column names indicate the candidate configuration identifier and the row names contain the name of the instances.

```
> # Get the experiments of the testing
> iraceResults$testing$experiments

          23       51       65      140      136      138
1t  23092745 23051175 22990020 23037470 23075260 22950192
2t  23194161 23161962 23088094 23059061 23091891 23129660
3t  22945832 22897901 22859548 22843498 22853508 22951710
4t  23267377 23232684 23230627 23222039 23252091 23214003
5t  23395153 23394636 23341288 23397462 23357325 23363846
6t  28501197 28516973 28509623 28385412 28352280 28403105
7t  28268948 28297752 28327431 28353541 28379836 28316938
8t  28130418 28143995 28110054 28133792 28200990 28169016
9t  28189479 28247877 28286336 28199681 28216944 28151761
10t 28620723 28687492 28627346 28619705 28674665 28671578
         146
1t  22992162
2t  23090099
3t  22853912
4t  23192286
5t  23338997
6t  28541594
7t  28361716
8t  28179508
9t  28139094
10t 28635274
```

  - `seeds`: The seeds used for the experiments, each seed corresponds to each instance in the rows of the test `experiments` matrix.

```
> # Get the experiments of the testing
> iraceResults$testing$seeds

        1t         2t         3t         4t         5t
 121390228  711920537 1468460454 1648782686 1402412889
        6t         7t         8t         9t        10t
2005328808    7550449  812584389 1382343924  212756813
```

  In the example, instance `1000-1.tsp` is executed with seed 121390228.

9.3 Analysis of results

The final configurations returned by **irace** are the elites of the final race. They are reported in decreasing order of performance, that is, the best configuration is reported first.

If testing is performed, you can further analyze the resulting best configurations by performing statistical tests in R or just plotting the results:

```
> results <- iraceResults$testing$experiments
> # Wilcoxon paired test
> conf <- gl(ncol(results), # number of configurations
+            nrow(results), # number of instances
+            labels = colnames(results))
> pairwise.wilcox.test (as.vector(results), conf, paired = TRUE, p.adj = "bonf")

Pairwise comparisons using Wilcoxon signed rank test

data:  as.vector(results) and conf

    23 51 65 140 136 138
51  1  -  -  -   -   -
65  1  1  -  -   -   -
140 1  1  1  -   -   -
136 1  1  1  1   -   -
138 1  1  1  1   1   -
146 1  1  1  1   1   1

P value adjustment method: bonferroni

> # Plot the results
> boxplot (iraceResults$testing$experiments,
+          ylab = "Solution cost",
+          xlab = "Configuration ID")
```

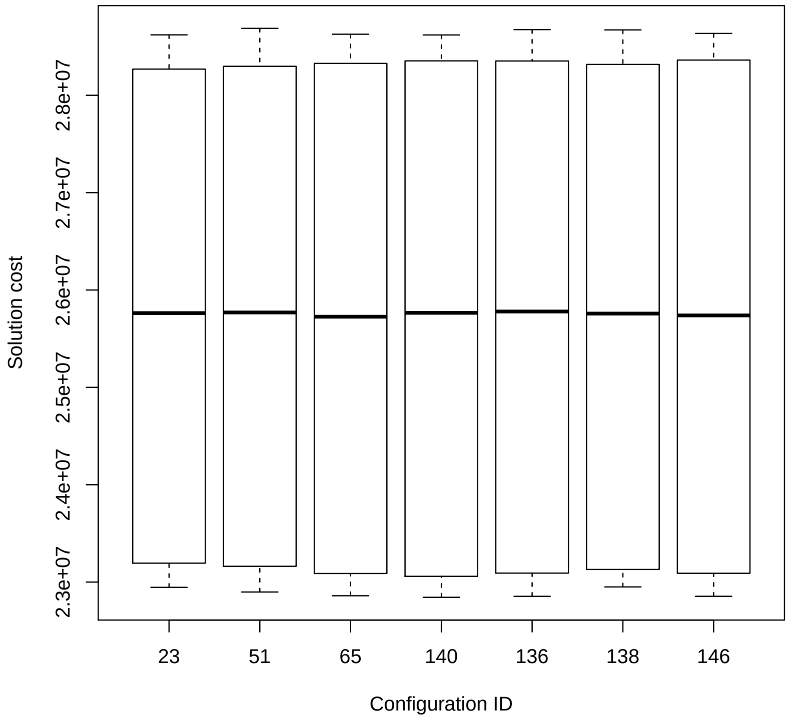

Figure 7: Boxplot of the testing results of the best configurations.

During the tuning, **irace** iteratively updates the sampling models of the parameters to focus on the best regions of the parameter search space. The frequency of the sampled configurations can provide insights on the parameter search space. We provide a function for plotting the frequency of the sampling of a set of configurations. For more information on this function, please see the R help, type in the R console: `?parameterFrequency`. The following example plots the frequency of the parameters sampled during one **irace** run:

```
> parameterFrequency(iraceResults$allConfigurations, iraceResults$parameters)

Plotting: algorithm
Plotting: localsearch
Plotting: alpha
Plotting: beta
Plotting: rho
Plotting: ants
Plotting: nnls
Plotting: q0
Plotting: dlb
Plotting: rasrank
Plotting: elitistants
```

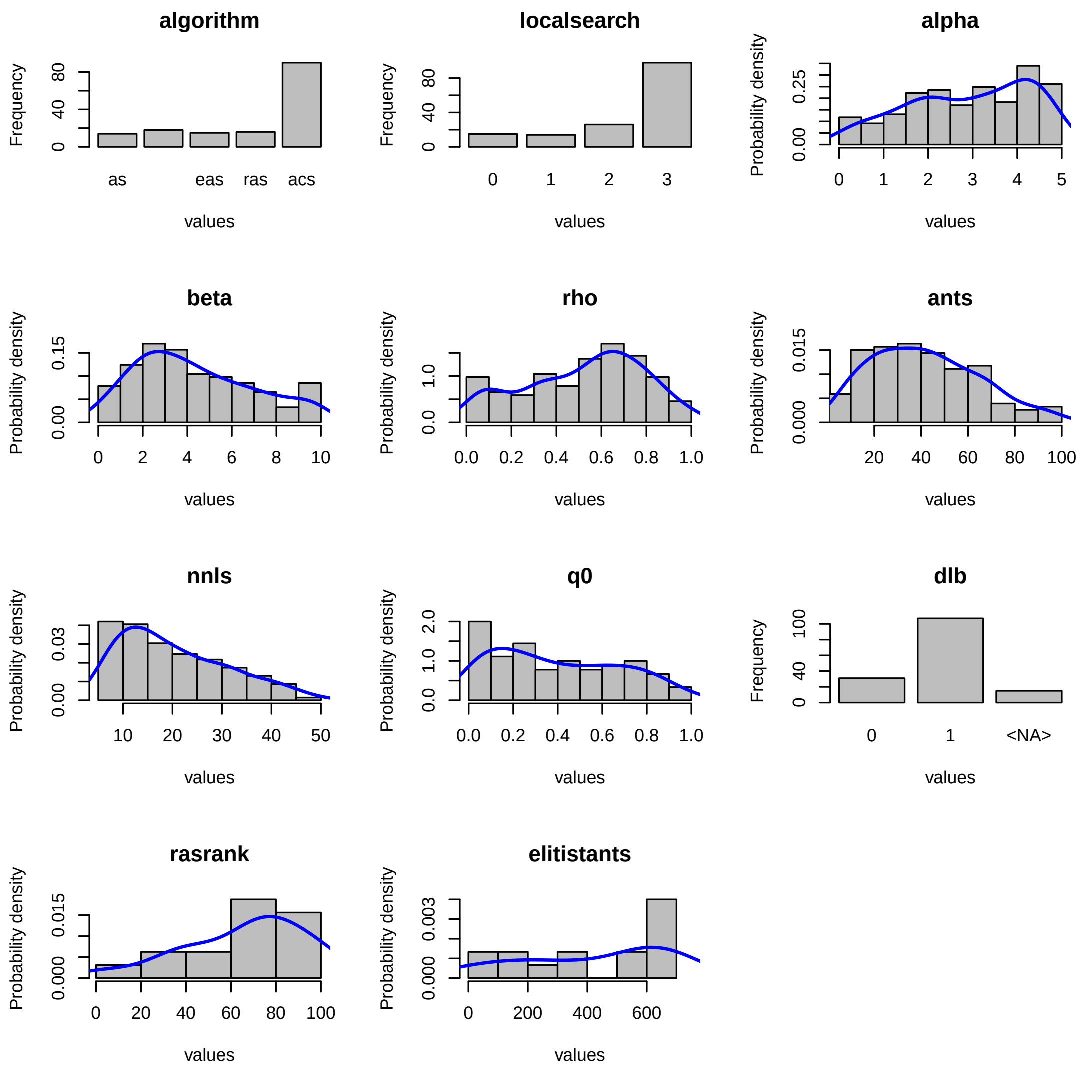

Figure 8: Parameters sampling frequency.

By using parallel coordinates plots, it is possible to analyze how the parameters interact with each other. For more information on this function, please see the R help, type in the R console: (`?parallelCoordinatesPlot`). The following example shows how to create a parallel coordinate plot of the configurations in the last two iterations of **irace**.

```
# Get last iteration number
last <- length(iraceResults$iterationElites)
# Get configurations in the last two iterations
conf <- getConfigurationByIteration(iraceResults = iraceResults,
                                    iterations = c(last - 1, last))
parallelCoordinatesPlot (conf, iraceResults$parameters,
```

```
param_names = c("algorithm", "alpha",
                "beta", "rho", "q0"),
hierarchy = FALSE)
```

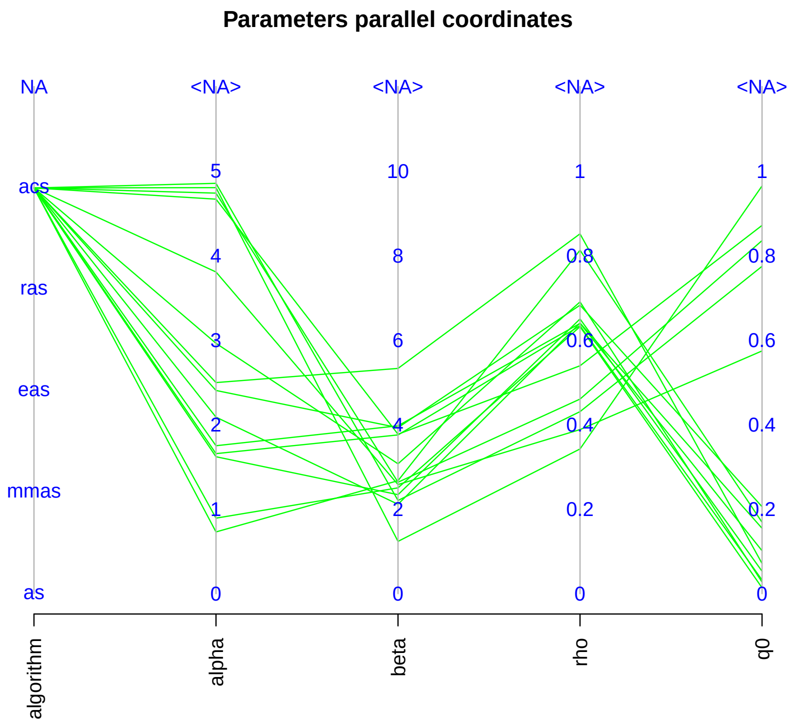

Figure 9: Parallel coordinate plots of the parameters of the configurations in the last two iterations of a run of **irace**.

10 Advanced topics

10.1 Tuning budget

Irace provides two options for setting the total tuning budget (`maxExperiments` and `maxTime`). Before setting the budget for the tuning, please consider the number of parameters that need to be tuned, available processing power and available time. The option `maxExperiments` limits the number of executions of `targetRunner` performed by **irace**. The option `maxTime` limits the total time of the `targetRunner` executions. When this latter option is used, `targetRunner` must return the evaluation cost together with the execution time (`"cost time"`).

When the goal is to minimize the computation time of an algorithm, and you wish to use maxTime as the tuning budget, targetRunner must return the time also as the evaluation cost, that is, return the time two times as "time time".

When using targetEvaluator and using maxTime as tuning budget, targetRunner just returns the time ("time") and targetEvaluator returns the cost.

When using maxTime, **irace** estimates the execution time of each targetRunner execution before the configuration. The amount of budget used for the estimation is set with the option budgetEstimation (default is 2%). The obtained estimation is adjusted after each iteration using the obtained results and it is used to estimate the number of experiments that can be executed. Internally, **irace** uses the number of remaining experiments to adjust the number of configurations tested in each race.

10.2 Multi-objective tuning

Currently, **irace** only optimizes one cost value at a time, which can be solution cost, computation time or any other objective that is returned to **irace** by the targetRunner. If the target algorithm is multi-objective, it will typically return not a single cost value, but a set of objective vectors (typically, a Pareto front). For tuning such a target algorithm with **irace**, there are two alternatives. If the algorithm returns a single vector of objective values, they can be aggregated into one single number by using, for example, a weighted sum. Otherwise, if the target algorithm returns a set of objective vectors, a unary quality metric (e.g., the hypervolume) may be used to evaluate the quality of the set.[3]

The use of aggregation or quality metrics often requires normalizing the different objectives. If normalization bounds are known a priori for each instance, normalized values can be computed by targetRunner. Otherwise, the bounds may be dynamically computed while running **irace**, by using targetEvaluator. In this case, targetRunner will save the output of the algorithm, then the first call to targetEvaluator will examine the output produced by all calls to targetRunner for the same instance, update the normalization bounds and return the normalized output. Subsequent calls to targetEvaluator for the same instance will simply return the normalized output. A similar approach can be used to dynamically compute the reference points or sets often required by unary quality metrics.

For more information about defining a targetEvaluator, see Section 5.3. Examples of tuning a multi-objective target algorithm using the hypervolume can be found in the examples at $IRACE_HOME/examples/hypervolume and $IRACE_HOME/examples/moaco.

10.3 Tuning for minimizing computation time

Irace was developed primarily for tuning algorithms that report solution cost. When using **irace** for tuning algorithms that report computation time to reach a target, the execution time of a configuration must be returned instead of the cost by the targetRunner. Even though **irace** can be used for minimizing computation time, **irace** may itself require more time to do so in its current version than other methods, such as ParamILS[4] or SMAC[5], since it does not make use of techniques, such as “adaptive capping”, that avoid long runs of the target algorithm.

We are currently extending **irace** with an adaptive capping mechanism.

[3] An implementation is publicly available at http://lopez-ibanez.eu/hypervolume [3]
[4] http://www.cs.ubc.ca/labs/beta/Projects/ParamILS/
[5] http://www.cs.ubc.ca/labs/beta/Projects/SMAC/

10.4 Heterogeneous scenarios

We classify a scenario as homogeneous when the target algorithm has a consistent performance regarding the instances; roughly speaking, good configurations tend to perform well and bad configurations tend to perform poorly on all instances of the problem. By contrast, in heterogeneous scenarios, the target algorithm has an inconsistent performance on different instances, that is, some configurations perform well for a subset of the instances, while they perform poorly for a different subset.

When facing a heterogeneous scenario, the first question should be whether the objective of tuning is to find configurations that perform reasonably well over all instances, even if they are not the best ones in any of them. If this is not the case, then it would be better to partition instances into more similar subsets and execute **irace** separately on each subset. This will lead to a portfolio of algorithm configurations, one for each subset, and algorithm selection techniques can be used to select the best configuration from the portfolio when facing a new instance.

If finding an overall good configuration for all the instances is the objective, then we recommend that instances are randomly sampled (option `sampleInstances`), unless one can provide the instances in a particular order that does not bias the tuning towards any subset. For example, let's assume a heterogeneous scenario with two classes of instances. If training instances are not sampled and the first ten instances belong to only one class, the tuning will be biased towards configurations that perform good for those instances. An optimal order would not ever present consecutively two instances of the same type.

In addition, it may be useful to increase the number of instances executed before doing a statistical test in order to see more instance classes before discarding configurations. The option `elitistNewInstances` in elitist **irace** (option `elitist`) can be used to increase the number of new instances executed in each iteration, e.g., `--elitist-new-instances 5` (default value is 1). For the non-elitist **irace**, the option `firstTest` may be used for the same purpose, e.g., `--first-test 10` (default value is 5).

While executing **irace**, the homogeneity of the scenario can be observed by examining the values of Spearman's rank correlation coefficient and Kendall's concordance coefficient in the text output of **irace**. See Section 9.1 for more information.

10.5 Choosing the statistical test

The statistical test used in **irace** identifies statistically bad performing configurations that can be discarded from the race in order to save budget. Different statistical tests use different criteria to compare the cost of the configurations, which has an effect on the tuning results.

Irace provides two types of statistical tests (option `testType`). Each test has different characteristics that are beneficial for different goals:

- Friedman test (`F-test`): This test uses the ranking of the configurations to analyze the differences between their performance. This makes the test suitable for scenarios where the numerical results and their scale are not significant to assess the cost of the configurations. For example, if the results for different instances have high numerical differences and evaluating the performance of the configurations using the mean could be deceiving. We recommend to use the `F-test` (default) when tuning for solution cost and whenever the best performing algorithm should be among the best in as many instances as possible.

- Student's t-test (`t-test`): This test uses the mean performance of the configurations to analyze the differences between the configurations. This makes the test suitable for scenarios where the differences between values obtained for different instances are relevant to assess

good configurations. We recommend using t-test, in particular, when the target algorithm is minimizing computation time and, in general, whenever the best configurations should obtain the best average solution cost.

The confidence level of the tests may be adjusted by using the option `confidence`. Increasing the value of `confidence` leads to a more strict statistical test. Keep in mind that a stricter test will require more budget to identify which configurations perform worse. A less strict test discards configurations faster by requiring less evidence against them and, therefore, it is more likely to discard good configurations.

10.6 Complex parameters

Some parameters may have complex dependencies. Ideally, parameters should be defined in the way that is more likely to help the search performed by **irace**. For example, when tuning a branch and bound algorithm, one may have the following parameters:

- branching (`b`) that takes values in `{0,1,2,3}`, where 0 indicates no branching will be used and the rest are different types of branching.
- stabilization (`s`) that takes values in `{0,1,2,3,4,5,6,7,8,9,10}`, of which for `b=0` only `{0,1,2,3,4,5}` are relevant.

In this case, it is not possible to describe the parameter space by defining only two parameters for **irace**. An extra parameter must be introduced as follows:

```
# name    label   type   range                    condition
b         "-b "   c      (0,1,2,3)
s1        "-s "   c      (0,1,2,3,4,5)            | b == "0"
s2        "-s "   c      (0,1,2,3,4,5,6,7,8,9,10) | b != "0"
```

Parameters whose values depend on the value of other parameters may also require using extra parameters or changing the parameters and processing them in `targetRunner`. For example, given the following parameters:

- Population size (`p`) takes the integer values $[1, 100]$.
- Selection size (`s`) takes the same values but no more than the population size, that is [1,p].

In this case, it is possible to describe the parameters `p` and `s` using surrogate parameters for **irace** that represent a ratio of the original interval as follows:

```
# name    label   type   range
p1        "-p "   r      (0.0,1.0)
s1        "-s "   r      (0.0,1.0)
```

and the values must be further processed in `targetRunner`. For example, if the surrogate parameter `p1` has value 0.5, mapping it to the original interval of $[1, 100]$, we obtain a value of $\texttt{p} = 51$. More than one value of the surrogate parameter (e.g., 0.501 and 0.502) result in the same final value. Parameter `s` has an interval that depends on the final value of parameter `p`, if the surrogate parameter `s1` has value 0.3, it must be mapped to the interval $[1, 51]$, giving a value of $\texttt{s} = 16$.

The processing within `targetRunner` can also split and join parameters. For example, assume the following parameters:

```
# name    label   type   range
m         "-m "   i      (1,250)
e         "-e "   r      (0.0,2.0)
```

These parameters could be used to define a value $\mathtt{m}\cdot 10^{\mathtt{e}}$ for another parameter (`--strength`) not known by **irace**. Then, `targetRunner` takes care of parsing `-m` and `-e`, computing the strength value and passing the parameter `--strength` together with its value to the target algorithm.

10.7 Unreliable target algorithms

There are some situations in which the target algorithm may fail to execute correctly. By default, **irace** stops as soon as a call to `targetRunner` or `targetEvaluator` fails, which helps to detect bugs in the target algorithm. Sometimes the failure cannot be fixed because it is due to system problems, network issues, memory limits, bugs for which no fix is available, or fixing them is impossible because there is no access to the source code.

In those cases, if the failure is caused by random errors or transient system problems, one may wish to ignore the error and try again the same call in the hope that it succeeds. The option `targetRunnerRetries` indicates the number of times a `targetRunner` execution is repeated if it fails. Use this option only if you know additional repetitions could be successful.

If the target algorithm consistently fails for a particular set of configurations, these configurations may be declared as forbidden (`forbiddenFile`) so that **irace** avoids them. On the other hand, if the configurations that cause the problem are unknown, the `targetRunner` script should detect the failure and return a penalty cost (a very large cost value) so that **irace** discards the failing configuration as soon as possible. The penalty must be set according to the range of the cost measure and the goals of the tuning. For example, a configuration that crashes on a particular instance, e.g., by running out of memory, might still be considered acceptable if it gives very good results on other instances.

11 List of command-line and scenario options

Most **irace** options can be specified in the command line using a flag or in the **irace** scenario file using the option name (or setting their value in the `scenario` list passed to the various R functions exported by the package). This section describes the various **irace** options that can be specified by the user in this way.

11.1 General options

`scenarioFile` *flag:* `-s` *or* `--scenario` *default:* `./scenario.txt`
: File that contains the scenario setup and other irace options. All options listed in this section can be included in this file. See `$IRACE_HOME/templates/` for an example.

`debugLevel` *flag:* `--debug-level` *default:* `0`
: Level of information to display in the text output of **irace**. A value of 0 silences all debug messages. Higher values provide more verbose debug messages. To see details about the text output of **irace**, see Section 9.1.

`seed` *flag:* `--seed` *default:* `NA`
: Seed to initiallize the random number generator. The seed must be a positive integer. If the seed is `NA`, a random seed will be generated.

`execDir` *flag:* `--exec-dir` *default:* `./`

Directory where the target algorithm executions will be performed. The default execution directory is the current directory.

> The execution directory must exist before executing **irace**, it will not be created automatically.

`logFile` *flag:* `-l` *or* `--log-file` *default:* `./irace.Rdata`

File to save tuning results as an R dataset. The provided path must be either an absolute path or relative to `execDir`. See Section 9.2 for details on the format of the R dataset.

11.2 Elitist irace

`elitist` *flag:* `--elitist` *default:* `1`

Enable/disable elitist **irace**.

In the **elitist** version of `irace` [5], elite configurations are not discarded from the race until non-elite configurations have been executed on the same instances as the elite configurations.

Each race begins by evaluating all configurations on a number of new instances. This number is defined by the option `elitistNewInstances`. After the new instances have been evaluated, configurations are evaluated on instances seen in the previous race. Elite configurations already have results for most of these previous instances and, therefore, do not need to be re-evaluated. Finally, after configurations have been evaluated on all these instances, the race continues by evaluating additional new instances.

The statistical tests can be performed at any moment during the race according to the setting of the options `firstTest` and `eachTest`. The elitist rule forbids discarding elite configurations, even if the show poor performance, until the last of the previous instances is seen in the race.

The **non-elitist** version of **irace** can discard elite configurations at any point of the race, instances are not re-used from one race to the next, and new instances are sampled for each race.

`elitistNewInstances` *flag:* `--elitist-new-instances` *default:* `1`

Number of new instances added to each race before evaluating instances from previous races (only for elitist **irace**).

> If `deterministic` is `TRUE` then the number of `elitistNewInstances` will be reduced or set to `0` once all instances have been evaluated.

`elitistLimit` *flag:* `--elitist-limit` *default:* `2`

Maximum number of statistical tests performed without successful elimination after all instances from the previous race have been evaluated. If the limit is reached, the current race is stopped. Only valid for elitist **irace**. Use 0 to disable the limit.

11.3 Internal irace options

`sampleInstances` *flag:* `--sample-instances` *default:* `1`

Enable/disable the sampling of the training instances. If the option `sampleInstances` is disabled, the instances are used in the order provided in the `trainInstancesFile` or in the order they are read from the `trainInstancesDir` when`trainInstancesFile` is not provided. For more information about training instances see Section 5.4.

`nbIterations` *flag:* `--iterations` *default:* `0`

Minimum number of iterations to be executed. Each iteration involves the generation of new configurations and the use of racing to select the best configurations. By default (with 0), **irace** calculates the minimum number of iterations as $N^{\text{iter}} = \lfloor 2 + \log_2 N^{\text{param}} \rfloor$, where N^{param} is the number of non-fixed parameters to be tuned. We recommend to use the default value.

`nbExperimentsPerIteration` *flag:* `--experiments-per-iteration` *default:* `0`

Number of experiments to execute per iteration. By default (when equal to 0), this value changes for each iteration and depends on the iteration index and the remaining budget. Further details are provided in the **irace** paper [5]. We recommend to use the default value.

`nbConfigurations` *flag:* `--num-configurations` *default:* `0`

The number of configurations that will be raced at each iteration. By default (when equal to 0), this value changes for each iteration and depends on `nbExperimentsPerIteration`, the iteration index and `mu`. The precise details are given in the **irace** paper [5]. We recommend to use the default value.

`mu` *flag:* `--mu` *default:* `5`

This value is used to determine the number of configurations to be sampled and evaluated at each iteration. The number of configurations will be calculated such that there is enough budget in each race to evaluate all configurations on at least $\mu + \min(5, j)$ training instances, where j is the index of the current iteration. The value of μ will be adjusted to never be lower than the value of `firstTest`. We recommend to use the default value and, if needed, adjust `firstTest` and `eachTest`, instead.

`minNbSurvival` *flag:* `--min-survival` *default:* `0`

The minimum number of configurations needed to continue the execution of a race. If the number of configurations alive in the race is not larger than this value, the current iteration will stop and a new iteration will start, even if there is budget left to continue the current race. By default (when equal to 0), the value is calculated automatically as $\lfloor 2 + \log_2 N^{\text{param}} \rfloor$, where N^{param} is the number of non-fixed parameters to be tuned.

`softRestart` *flag:* `--soft-restart` *default:* `1`

Enable/disable the soft-restart strategy that avoids premature convergence of the probabilistic model. When a sampled configuration is *similar* to its parent configuration, the probabilistic model of these configurations is soft restarted. The soft-restart mechanism is explained in the **irace** paper [5]. The similarity of categorical and ordinal parameters is given by the hamming distance, and the option `softRestartThreshold` defines the similarity of numerical parameters.

`softRestartThreshold` *flag:* `--soft-restart-threshold` *default:* `NA`

Soft restart threshold value for numerical parameters. If `NA`, it is computed as $10^{-digits}$, where `digits` corresponds to the **irace** option explained in this section.

11.4 Target algorithm parameters

`parameterFile` *flag:* `-p` *or* `--param-file` *default:* `./parameters.txt`
File that contains the description of the parameters of the target algorithm. See Section 5.1.

`digits` *flag:* `--digits` *default:* `4`
Maximum number of decimal places that are significant for numerical (real) parameters.

`forbiddenFile` *flag:* `--forbidden-file` *default:*
File containing a list of logical expressions that cannot be true for any evaluated configuration. If empty or `NULL`, no forbidden configurations are considered. See Section 5.6 for more information.

11.5 Target algorithm execution

`targetRunner` *flag:* `--target-runner` *default:* `./target-runner`
This option defines a script or an R function that evaluates a configuration of the target algorithm on a particular instance. See Section 5.2 for details.

`targetRunnerRetries` *flag:* `--target-runner-retries` *default:* `0`
Number of times to retry a call to `targetRunner` if the call failed.

`targetRunnerData` *default:* `NULL`
Optional data passed to `targetRunner`. This is ignored by the default `targetRunner` function, but it may be used by custom `targetRunner` functions to pass persistent data around.

`targetRunnerParallel` *default:* `NULL`
Optional R function to provide custom parallelization of `targetRunner`. See Section 6 for more information.

`targetEvaluator` *flag:* `--target-evaluator` *default:* `""`
Optional script or R function that returns a numerical value for an experiment after all configurations have been executed on a given instance using `targetRunner`. See Section 5.3 for details.

`deterministic` *flag:* `--deterministic` *default:* `0`
Enable/disable deterministic target algorithm mode. If the target algorithm is deterministic, configurations will be evaluated only once per instance. See Section 5.4 for more information.

> If the number of instances provided is less than the value specified for the option `firstTest`, no statistical test will be performed.

`parallel` *flag:* `--parallel` *default:* `0`
Number of calls of the `targetRunner` to execute in parallel. A value of 0 means no parallelization. For more information on parallelization, see Section 6.

`loadBalancing` *flag:* `--load-balancing` *default:* `1`
Enable/disable load-balancing when executing experiments in parallel. Load-balancing makes better use of computing resources, but increases communication overhead. If this overhead is large, disabling load-balancing may be faster. See Section 6.

`mpi` *flag:* `--mpi` *default:* `0`

Enable/disable use of **Rmpi** to execute the `targetRunner` in parallel using MPI protocol. When `mpi` is enabled, the option `parallel` is the number of slave nodes. See Section 6.

`sgeCluster` *flag:* `--sge-cluster` *default:* `0`

Enable/disable SGE cluster mode, which uses `qstat` to wait for cluster jobs to finish (`targetRunner` must invoke `qsub`). See Section 6.

11.6 Initial configurations

`configurationsFile` *flag:* `--configurations-file` *default:*

File containing a list of initial configurations. If empty or `NULL`, **irace** will not use initial configurations. See Section 5.5.

> The provided configurations must not violate the constraints described in `parameterFile` and `forbiddenFile`.

11.7 Training instances

`trainInstancesDir` *flag:* `--train-instances-dir` *default:* `./Instances`

Directory where tuning instances are located; either absolute path or relative to current directory. See Section 5.4.

`trainInstancesFile` *flag:* `--train-instances-file` *default:*

File containing a list of instances and optionally additional parameters for them. See Section 5.4.

> If `trainInstancesDir` is specified, the path contained in `trainInstancesFile` must be relative to the directory. When using an absolute path or for defining instances that are not files, set `trainInstancesDir=""`.

11.8 Tuning budget

`maxExperiments` *flag:* `--max-experiments` *default:* `0`

The maximum number of runs (invocations of `targetRunner`) that will be performed. It determines the maximum budget of experiments for the tuning. See Section 10.1.

`maxTime` *flag:* `--max-time` *default:* `0`

The maximum total time in seconds for the runs of `targetRunner` that will be performed. The mean execution time of each run is estimated in order to calculate the maximum number of experiments (see option `budgetEstimation`). When `maxTime` is positive, then `targetRunner` **must** return the execution time as its second output. See Section 10.1.

`budgetEstimation` *flag:* `--budget-estimation` *default:* `0.02`

The percentage of the budget used for estimating the mean execution time. Only used when `maxTime` > 0. See Section 10.1.

11.9 Statistical test

`testType` *flag:* `--test-type` *default:* `F-test`
Specifies the statistical test type:

`F-test` (Friedman test)

`t-test` (pairwise t-tests with no correction)

`t-test-bonferroni` (t-test with Bonferroni's correction for multiple comparisons)

`t-test-holm` (t-test with Holm's correction for multiple comparisons).

We recommend to not use corrections for multiple comparisons because the test typically becomes too strict and the search stagnates. See Section 10.5 for details about choosing the statistical test most appropriate for your scenario.

`firstTest` *flag:* `--first-test` *default:* `5`
Specifies how many instances are evaluated before the first elimination test.

> The value of `firstTest` must be a multiple of `eachTest`.

`eachTest` *flag:* `--each-test` *default:* `1`
Specifies how many instances are evaluated between elimination tests.

`confidence` *flag:* `--confidence` *default:* `0.95`
Confidence level for the elimination test.

11.10 Recovery

`recoveryFile` *flag:* `--recovery-file` *default:* `""`
Previously saved **irace** log file that should be used to recover the execution of **irace**; either absolute path or relative to the current directory. If empty or `NULL`, recovery is not performed. For more details about recovery, see Section 11.10.

11.11 Testing

`testNbElites` *flag:* `--test-num-elites` *default:* `1`
Number of elite configurations returned by irace that will be tested if test instances are provided. For more information about the testing, see Section 7.

`testIterationElites` *flag:* `--test-iteration-elites` *default:* `0`
Enable/disable testing the elite configurations found at each iteration.

`testInstancesDir` *flag:* `--test-instance-dir` *default:*
Directory where testing instances are located, either absolute or relative to the current directory.

`testInstancesFile` *flag:* `--test-instance-file` *default:*
File containing a list of test instances and, optionally, additional parameters for them.

`--only-test` *default:*
Run the configurations provided in the file argument on the test instances. See Section 7.

12 FAQ

12.1 Is irace minimizing or maximizing the output of my algorithm?

By default, **irace** considers that the value returned by `targetRunner` (or by `targetEvaluator`, if used) should be **minimized**. In case of a maximization problem, one can simply multiply the value by -1 before returning it to irace. This is done, for example, when maximizing the hypervolume (see the last lines in `$IRACE_HOME/examples/hypervolume/target-evaluator`).

12.2 Is it possible to configure a MATLAB algorithm with irace?

Definitely. There are two main ways to achieve this:

1. Edit the `targetRunner` script to call MATLAB in a non-interactive way. See the MATLAB documentation, or the following links.[6] You would need to pass the parameter received by `targetRunner` to your MATLAB script: `http://www.mathworks.nl/support/solutions/en/data/1-1BS5S/?solution=1-1BS5S`. There is a minimal example in:

   `$IRACE_HOME/examples/matlab/`.

2. Call MATLAB code directly from R using the **R.matlab** package (`https://cran.r-project.org/package=R.matlab`). This is a better option if you are experienced in R. Define `targetRunner` as an R function instead of a path to a script. The function should call your MATLAB code with appropriate parameters.

12.3 My program works perfectly on its own, but not when running under irace. Is irace broken?

Every time this was reported, it was a difficult-to-reproduce bug in the program, not in **irace**. We recommend that in `targetRunner`, you use `valgrind` to run your program. That is, if your program is called like:

```
$EXE ${FIXED_PARAMS} -i $INSTANCE ${CONFIG_PARAMS} \
 1> ${STDOUT} 2> ${STDERR}
```

then replace that line with:

```
valgrind --error-exitcode=1 $EXE ${FIXED_PARAMS} \
 -i $INSTANCE ${CONFIG_PARAMS} 1> ${STDOUT} 2> ${STDERR}
```

If there are bugs in your program, they will appear in `$STDERR`, thus do not delete those files.

12.4 My program may be buggy and run into an infinite loop. Is it possible to set a maximum timeout?

We are not aware of any way to achieve this using R. However, in GNU/Linux, it is easy to implement by using the `timeout` command in `targetRunner` when invoking your program.

[6] `http://stackoverflow.com/questions/1518072/suppress-start-message-of-matlab`
`http://stackoverflow.com/questions/4611195/how-to-call-matlab-from-command-line-and-print-to-stdout-before-exiting`

12.5 When using the mpi option, irace is aborted with an error message indicating that a function is not defined. How to fix this?

Rmpi does not work the same way when called from within a package and when called from a script or interactively. When **irace** creates the slave nodes, the slaves will load a copy of **irace** automatically. If the slave nodes are on different machines, they must have **irace** installed. If **irace** is not installed system-wide, R needs to be able to find **irace** on the slave nodes. This is usually done by setting `R_LIBS`, `.libPaths()` or by loading **irace** using `library()` or `require()` with the argument "`lib.loc`". The settings on the master are not applied to the slave nodes automatically, thus the slave nodes may need their own settings. After spawning the slaves, it is too late to modify those settings, thus modifying the shell variable `R_LIBS` seems the only valid way to tell the slaves where to find **irace**.

If the path is set correctly and the problem persists, please check these instructions:

1. Test that **irace** and **Rmpi** work. Run **irace** on a single machine (submit node), without calling `qsub`, `mpirun` or a similar wrapper around **irace** or R.

2. Test loading **irace** on the slave nodes. However, jobs submitted by `qsub`/`mpirun` may load R packages using a different mechanism from the way it happens if you log directly into the node (e.g., with `ssh`). Thus, you need to write a little R program such as:

```
library(Rmpi)
mpi.spawn.Rslaves(nslaves = 10)
x <- mpi.applyLB(1:10, function(x) {
  library(irace)
  return(path.package("irace")) })
      print(x)
```

   Submit this program to the cluster (using `qsub`/`mpirun`) like you would submit **irace**.

3. In the script `bin/parallel-irace-mpi`, the function `irace_main()` creates an MPI job for our cluster. You may need to speak with the admin of your cluster and ask them how to best submit a job for MPI. There may be some particular settings that you need. **Rmpi** normally creates log files; but **irace** suppresses those files unless `debugLevel` > 0.

Please contact us (Section 13) if you have further problems.

12.6 Error: 4 arguments passed to `.Internal(nchar)` which requires 3

This is a bug in R 3.2.0 on Windows. The solution is to update your version of R.

13 Resources and contact information

More information about the package can be found on the **irace** webpage:

http://iridia.ulb.ac.be/irace/

For questions and suggestions please contact the development team through the **irace** package Google group:

https://groups.google.com/d/forum/irace-package

or by sending an email to:

irace-package@googlegroups.com

14 Acknowledgements

We would like to thank all the people that directly or indirectly have collaborated in the development and improvement of **irace**: Prasanna Balaprakash, Zhi (Eric) Yuan, Franco Mascia, Alberto Franzin, Anthony Antoun, Esteban Diaz Leiva, Federico Caselli, and Pablo Valledor Pellicer.

Bibliography

[1] M. Birattari. On the estimation of the expected performance of a metaheuristic on a class of instances. how many instances, how many runs? Technical Report TR/IRIDIA/2004-001, IRIDIA, Université Libre de Bruxelles, Belgium, 2004.

[2] M. Birattari, Z. Yuan, P. Balaprakash, and T. Stützle. F-race and iterated F-race: An overview. In T. Bartz-Beielstein, M. Chiarandini, L. Paquete, and M. Preuss, editors, *Experimental Methods for the Analysis of Optimization Algorithms*, pages 311–336. Springer, Berlin, Germany, 2010.

[3] C. M. Fonseca, L. Paquete, and M. López-Ibáñez. An improved dimension-sweep algorithm for the hypervolume indicator. In *Proceedings of the 2006 Congress on Evolutionary Computation (CEC 2006)*, pages 1157–1163. IEEE Press, Piscataway, NJ, July 2006. doi: 10.1109/CEC.2006.1688440.

[4] M. López-Ibáñez, J. Dubois-Lacoste, T. Stützle, and M. Birattari. The irace package, iterated race for automatic algorithm configuration. Technical Report TR/IRIDIA/2011-004, IRIDIA, Université Libre de Bruxelles, Belgium, 2011. URL `http://iridia.ulb.ac.be/IridiaTrSeries/IridiaTr2011-004.pdf`.

[5] M. López-Ibáñez, J. Dubois-Lacoste, L. Pérez Cáceres, T. Stützle, and M. Birattari. The irace package: Iterated racing for automatic algorithm configuration. *Operations Research Perspectives*, 3:43–58, 2016. doi: 10.1016/j.orp.2016.09.002.

[6] C. C. McGeoch. Analyzing algorithms by simulation: Variance reduction techniques and simulation speedups. *ACM Computing Surveys*, 24(2):195–212, 1992. doi: 10.1145/130844.130853.

[7] M. Schneider and H. H. Hoos. Quantifying homogeneity of instance sets for algorithm configuration. In Y. Hamadi and M. Schoenauer, editors, *Learning and Intelligent Optimization, 6th International Conference, LION 6*, volume 7219 of *Lecture Notes in Computer Science*, pages 190–204. Springer, Heidelberg, Germany, 2012. doi: 10.1007/978-3-642-34413-8_14.

Appendix A Installing R

This section gives a quick R installation guide that will work in most cases. The official instructions are available at https://cran.r-project.org/doc/manuals/r-release/R-admin.html

A.1 GNU/Linux

You should install R from your package manager. On a Debian/Ubuntu system it will be something like:

```
sudo apt-get install r-base
```

Once R is installed, you can launch R from the Terminal and from the R prompt install the **irace** package (see Section 3.2).

A.2 OS X

You can install R directly from a CRAN mirror.[7] Alternatively, if you use homebrew, you can just brew the R formula from the science tap (unfortunately it does not come already bottled so you need to have Xcode[8] installed to compile it):

```
brew tap homebrew/science
brew install r
```

Once R is installed, you can launch R from the Terminal (or from your Applications), and from the R prompt install the **irace** package (see Section 3.2).

A.3 Windows

You can install R from a CRAN mirror.[9] We recommend that you install R on a filesystem path without spaces, special characters or long names, such as `C:\R`. Once R is installed, you can launch the R console and install the **irace** package from it (see Section 3.2).

Appendix B targetRunner troubleshooting checklist

If the `targetRunner` script fails to return the output expected by **irace**, it can be sometimes difficult to diagnose where the problem lies. The more descriptive errors provided by your script, the easier it will be to debug it. If you are using temporary files to redirect the output of your algorithm, check that these are created properly. We recommend to follow the structure of the example file (`target-runner`) provided in `$IRACE_HOME/templates`. The following examples are based on a file with those characteristics.

In case of failure of `targetRunner`, **irace** will print an error on its output describing which execution of `targetRunner` was not successful. Follow this checklist to detect where the problem is:

1. Make sure that your `targetRunner` script or program is at the specified location. If you see this error:

[7] https://cran.r-project.org/bin/macosx/

[8] Xcode download webpage: https://developer.apple.com/xcode/download/

[9] http://cran.r-project.org/bin/windows/

```
Error: == irace == target runner '~/tuning/target-runner' does not exist
```

it means that **irace** cannot find the `target-runner` file. Check that the file is at the path specified by the error.

2. Make sure that your `targetRunner` script is an executable file and the user running **irace** has permission to execute it. The following errors:

   ```
   Error: == irace == target runner '~/tuning/target-runner' is a directory,
   not a file
   ```

   or

   ```
   Error: == irace == target runner '~/tuning/target-runner' is not executable
   ```

   mean that your `targetRunner` is not an executable file. In the first case, the script is a folder and therefore there must be a problem with the name of the script. In the second case, you must make the file executable, which in GNU/Linux can be done by:

   ```
   chmod +x ~/tuning/target-runner
   ```

3. If your `targetRunner` script calls another program, make sure it is at the location described in the script (variable `EXE` in the examples and templates). A typical output for such an error is:

   ```
   Error: == irace == running command ''~/tuning/target-runner' 1 8 676651103
   ~/tuning/Instances/1000-16.tsp --ras --localsearch 2 --alpha 4.03 --beta 1.89
   --rho  0.02 --ants 37 --nnls 48 --dlb 0 --rasranks 15 2>\&1' had status 1
   == irace == The call to target.runner.default was:
   ~/tuning/target-runner 1 8 676651103 ~/tuning/Instances/1000-16.tsp --ras
   --localsearch 2 --alpha 4.03 --beta 1.89 --rho  0.02 --ants 37 --nnls 48
   --dlb 0 --rasranks 15
   == irace == The output was:
   Tue May  3 19:00:37 UTC 2016: error: ~/bin/acotsp: not found or not executable
   (pwd: ~/tuning/acotsp-arena)
   ```

   You may test your script by copying the command line shown in the error and executing `target-runner` directly on the execution directory (execDir). In this case, the command line is:

   ```
   ~/tuning/target-runner 1 8 676651103 ~/tuning/Instances/1000-16.tsp --ras \
    --localsearch 2 --alpha 4.03 --beta 1.89 --rho 0.02 --ants 37 --nnls 48 \
    --dlb 0 --rasranks 15
   ```

   This executes the `targetRunner` script as **irace** does. The output of this script must be only one number.

4. Check that your `targetRunner` script is actually returning one number as output. If you see an error as the following, this is your problem:

   ```
   Error: == irace == The output of '~/tuning/target-runner
   1 25 365157769 ~/tuning/Instances/1000-31.tsp --ras
   --localsearch 1 --alpha 0.26  --beta 6.95 --rho  0.69
   ```

```
--ants 56 --nnls 10 --dlb 0 --rasranks 7' is not numeric!
== irace == The output was:
Solution: 24479793
```

For testing your script, copy the command-line of `target-runner` and execute it directly on the execution directory (`execDir`):

```
~/tuning/target-runner 1 25 365157769 ~/tuning/Instances/1000-31.tsp --ras \
 --localsearch 1 --alpha 0.26 --beta 6.95 --rho 0.69 --ants 56 \
 --nnls 10 --dlb 0 --rasranks 7
```

This executes the `targetRunner` script as **irace** does. The output of this script must be only one number. In this example, the output of the script is "`Solution:  24479793`", which is not a number. The code that `targetRunner` uses to parse the output of the algorithm must be checked.

5. Check that your `targetRunner` script is creating the output files for your algorithm. If you see an error as:

```
== irace == The output was: Tue May  3 19:41:40 UTC 2016:
error: c1-9.stdout: No such file or directory
```

The output file of the execution of your algorithm has not been created (check permissions) or has been deleted before the result can be read.

6. Other errors can produce the following output:

```
== irace == The output was: Tue May  3 19:49:06 UTC 2016:
error: c1-23.stdout: Output is not a number
```

This might be because your `targetRunner` script is not executing your algorithm correctly. To further investigate this issue, comment out the line that eliminates the temporary files that saves the output of your algorithm. Similar to this one

```
rm -f "${STDOUT}" "${STDERR}"
```

Execute directly the `targetRunner` command-line that is provided in the error message, look in your execution directory for the files that are created. Check the `.stderr` file for errors and the `.stdout` file to see the output that your algorithm produces.

7. It is possible that transient bugs in the target algorithm are only visible when running within **irace**, and `targetRunner` appears to work fine when executed directly in the command-line outside **irace** (See FAQ in Section 12.3). We recommend that in `targetRunner`, you use `valgrind` to run your program. That is, if your program is called like:

```
    $EXE ${FIXED_PARAMS} -i $INSTANCE ${CONFIG_PARAMS} \
     1> ${STDOUT} 2> ${STDERR}
```

then replace that line with:

```
   valgrind --error-exitcode=1 $EXE ${FIXED_PARAMS} \
    -i $INSTANCE ${CONFIG_PARAMS} 1> ${STDOUT} 2> ${STDERR}
```

If there are bugs in your program, they will appear in `$STDERR`, thus do not delete those files.

8. If your `targetRunner` script works when running irace with `parallel=1` but it fails when using higher number of cores, this may be due to any number of reasons:

   - If you submit jobs through a queuing system, the running environment when using the queuing system may not be the same as when you launch **irace** yourself. The queuing system may also send the job to different machines depending on the number of CPUs requested.
   - The above is particularly problematic when using MPI, because some calls to `targetRunner` may run on different computers than the one running the master **irace** process. See FAQ in Section 12.5.
   - Does `targetRunner` read or create intermediate files? These files may cause a race condition when two calls to `targetRunner` happen at the same time. Maybe these files consume too much memory or fill the filesystem when there are simultaneous `targetRunner` calls?
   - Does the machine or the queuing system impose any limits on number of processes or CPU/memory/filesystem usage per job? Such limits may only trigger when more than one process is executed in parallel, killing the `targetRunner` process before it has a chance to print anything useful. In that case, **irace** may not detect the the program finished unexpectedly, only that the expected output was not printed.

Appendix C Glossary

Parameter tuning: Process of searching good settings for the parameters of an algorithm under a particular tuning scenario (instances, execution time, etc.).

Scenario: Settings that define an instance of the tuning problem. These settings include the algorithm to be tuned (target), budget for the execution of the target algorithm (execution time, evaluations, iterations, etc.), set of problem instances and all the information that is required to perform the tuning.

Target algorithm: Algorithm whose parameters will be tuned.

Target parameter: Parameter of the target algorithm that will be tuned.

irace option: Configurable option of **irace**.

Elite configurations: Best configurations found so far by **irace**. New configurations for the next iteration of **irace** are sampled from the probabilistic models associated to the elite configurations. All elite configurations are also included in the next iteration.

$IRACE_HOME: The filesystem path where **irace** is installed. You can find this information by opening an R console and executing:

```
system.file(package = "irace")
```

Appendix D NEWS

NEWS

```
* Fix a bug that re-executed elite results under some circumstances.
  (Leslie Pérez Cáceres)
```

```
 * Fix examples/sge-cluster for elitist irace. (Manuel López-Ibáñez)

 * Restrict the number of maximum configurations per race to 1024.
   (Leslie Pérez Cáceres)

 * Do not warn if the last line in the instance file does not terminate with a
   newline. (Manuel López-Ibáñez)

 * Fix bug when deterministic == 1.
   (Manuel López-Ibáñez, Leslie Pérez Cáceres)

 * Update manual and vignette with details about the expected arguments and
   return value of targetRunner and targetEvaluator. (Manuel López-Ibáñez)

 * Many updates to the User Guide vignette. (Manuel López-Ibáñez)

 * Fix \dontrun example in irace-package.Rd (Manuel López-Ibáñez)

 * Fix bug: If testInstances contains duplicates, results of testing are not
   correctly saved in iraceResults$testing$experiments nor reported correctly
   at the end of a run. Now unique IDs of the form 1t, 2t, ... are used for
   each testing instance. These IDs are used for the rownames of
   iraceResults$testing$experiments and the names of the scenario$testInstances
   and iraceResults$testing$seeds vectors.  (Manuel López-Ibáñez)

 * Fix bug where irace keeps retrying the target-runner call even if it
   succeeds. (Manuel López-Ibáñez)

 * New command-line parameter

       --only-test FILE

   which just evaluates the configurations given in FILE on the testing
   instances defined by the scenario. Useful if you decide on the testing
   instances only after running irace. (Manuel López-Ibáñez)


2.1

 * Fix CRAN errors in tests. (Manuel López-Ibáñez)

 * Avoid generating too many configurations at once if the initial time
   estimation is too small. (Manuel López-Ibáñez)

2.0

 * Minimum R version is 2.15.

 * Elitist irace by default, it can be disabled with parameter --elitist 0.
   (Leslie Pérez Cáceres, Manuel López-Ibáñez)

 * The parameter --test-type gains two additional values:

     t-test-bonferroni (t-test with Bonferroni's correction for multiple
                        comparisons),
     t-test-holm (t-test with Holm's correction for multiple comparisons)

   (Manuel López-Ibáñez)

 * MPI does not create log files with --debug-level 0.
   (Manuel López-Ibáñez)
```

```
* For simplicity, the parallel-irace-* scripts do not use an auxiliary
  `tune-main` script.  For customizing them, make a copy and edit them
  directly.
  (Manuel López-Ibáñez)

* New parameters:

  --target-runner-retries : Retry target-runner this many times in case
                            of error.
                                                  (Manuel López-Ibáñez)

* We print diversity measures after evaluating on each instance:
  (Leslie Pérez Cáceres)

  - Kendall's W (also known as Kendall's coefficient of concordance) If 1,
    all candidates have ranked in the same order in all instances.  If 0, the
    ranking of each candidate on each instance is essentially random.

            W = Friedman / (m * (k-1))

  - Spearman's rho: average (Spearman) correlation coefficient computed on the
    ranks of all pairs of raters. If there are no repeated data values, a
    perfect Spearman correlation of +1 or -1 occurs when each of the variables
    is a perfect monotone function of the other.

* Many internal and external interfaces have changed. For example, now we
  consistently use 'scenario' to denote the settings passed to irace and
  'configuration' instead of 'candidate' to denote the parameter settings
  passed to the target algorithm. Other changes are:

  parameters$boundary -> parameters$domain
  hookRun             -> targetRunner
  hookEvaluate        -> targetEvaluator
  tune-conf           -> scenario.txt
  instanceDir         -> trainInstancesDir
  instanceFile        -> trainInstancesFile
  testInstanceDir     -> testInstancesDir
  testInstanceFile    -> testInstancesFile

* Minimal example of configuring a MATLAB program
  (thanks to Esteban Diaz Leiva)

* Paths to files or directories given in the scenario file are relative to the
  scenario file (except for --log-file, which is an output file and it is
  relative to --exec-dir). Paths given in the command-line are relative to the
  current working directory. Given

     $ cat scenario/scenario.txt
     targetRunner <- "./target-runner"
     $ irace -s scenario/scenario.txt

  irace will search for "./scenario/target-runner", but given

     $ irace -s scenario/scenario.txt --target-runner ./target-runner

  irace will search for "./target-runner".      (Manuel López-Ibáñez)

* New command-line wrapper for Windows installed at
  'system.file("bin/irace.bat", package="irace")'
  (thanks to Anthony Antoun)
```

* Budget can be specified as maximum time (maxTime, --max-time) consumed by
  the target algorithm. See the documentation for the details about how this
  is handled.
  (Leslie Pérez Cáceres, Manuel López-Ibáñez)

1.07

* The best configurations found, either at the end or at each iteration of an
  irace run, can now be applied to a set of test instances different from the
  training instances. See options testInstanceDir, testInstanceFile,
  testNbElites, and testIterationElites. (Leslie Pérez Cáceres, Manuel López-Ibáñez)

* The R interfaces of hookRun, hookEvaluate and hookRunParallel have changed.
  See help(hook.run.default) and help(hook.evaluate.default) for examples of
  the new interfaces.

* Printing of race progress now reports the actual configuration and instance
  IDs, and numbers are printed in a more human-readable format.
  (Leslie Pérez Cáceres, Manuel López-Ibáñez)

* Reduce memory use for very large values of maxExperiments.
  (Manuel López-Ibáñez, thanks to Federico Caselli for identifying the issue)

* New option --load-balancing (loadBalancing) for disabling load-balancing
  when executing jobs in parallel. Load-balancing makes better use of
  computing resources, but increases communication overhead. If this overhead
  is large, disabling load-balancing may be faster.
  (Manuel López-Ibáñez, thanks to Federico Caselli for identifying the issue)

* The option --parallel in Windows now uses load-balancing by default.
  (Manuel López-Ibáñez)

* The wall-clock time after finishing each task is printed in the output.
  (Manuel López-Ibáñez, thanks to Federico Caselli for providing an initial
  patch)

1.06

* Fix bug that could introduce spurious whitespace when printing the
  final configurations. (Manuel López-Ibáñez)

* Fix bug if there are more initial candidates than needed for the
  first race. (Leslie Pérez Cáceres, Manuel López-Ibáñez)

* New configuration options, mainly for R users:

  - hookRunParallel: Optional R function to provide custom
    parallelization of hook.run.

  - hookRunData: Optional data passed to hookRun. This is ignored by
    the default hookRun function, but it may be used by custom hookRun R
    functions to pass persistent data around.
  (Manuel López-Ibáñez)

1.05

* New option --version. (Manuel López-Ibáñez)

* Terminate early if there is no sufficient budget to run irace with

```
   the given settings. (Manuel López-Ibáñez)

 * The option --parallel (without --mpi) now works under Windows.
   (Manuel López-Ibáñez, thanks to Pablo Valledor Pellicer for testing
   it)

 * Improved error handling when running under Rmpi. Now irace will
   terminate as soon as the master node detects at least one failed
   slave node. This avoids irace reporting two times the same error.
   Also, irace will print all the unique errors returned by all slaves
   and not just the first one.
   (Manuel López-Ibáñez)

 * Forbidden configurations may be specified in terms of constraints
   on their values. Forbidden configurations will never be evaluated by irace.
   See --forbidden-file and inst/templates/forbidden.tmpl.
   (Manuel López-Ibáñez)

 * New option --recovery-file (recoveryFile) allows resuming a
   previous irace run. (Leslie Pérez Cáceres)

 * The confidence level for the elimination test is now
   configurable with parameter --confidence. (Leslie Pérez Cáceres)

 * Much more robust handling of relative/absolute paths. Improved support
   for Windows. (Leslie Pérez Cáceres, Manuel López-Ibáñez)

 * Provide better error messages for incorrect parameter
   descriptions. (Manuel López-Ibáñez)
    Examples:

    x "" i (0, 0)       # lower and upper bounds are the same
    x "" r (1e-4, 5e-4) # given digits=2, ditto
    x "" i (-1, -2)     # lower bound must be smaller than upper bound
    x "" c ("a", "a")   # duplicated values

 * Print elapsed time for calls to hook-run if debugLevel >=1.
   (Manuel López-Ibáñez)

 * examples/hook-run-python/hook-run: A multi-purpose hook-run written
   in Python. (Franco Mascia)

 * Parallel mode in an SGE cluster (--sge-cluster) is more
   robust. (Manuel López-Ibáñez)

1.04

 * Replace obsolete package multicore by package parallel
   (requires R >= 2.14.0)

 * Use load-balancing (mc.preschedule = FALSE) in mclapply.

1.03

 * Use reg.finalizer to finish Rmpi properly without clobbering
   .Last().

 * Remove uses of deprecated as.real().

 * Nicer error handling in readParameters.

 * Add hypervolume (multi-objective) example.
```

```
 * Fix several bugs in the computation of similar candidates.

1.02

 * More concise output.

 * The parameters expName and expDescription are now useless and they
   were removed.

 * Faster computation of similar candidates (Jeremie Dubois-Lacoste
   and Leslie Pérez Cáceres).

 * Fix bug when saving instances in tunerResults$experiments.

 * irace.cmdline ("--help") does not try to quit R anymore.

1.01

 * Fix bug caused by file.exists (and possibly other functions)
   not handling directory names with a trailing backslash or slash on
   Windows.

 * Fix bug using per-instance parameters (Leslie Pérez Cáceres).

 * Fix bug when reading initial candidates from a file.
```
