# Supplementary material for: Iterative improvement in the automatic modular design of robot swarms
Source: PeerJ Comput Sci. 2020 Dec 7;6:e322. doi: 10.7717/peerj-cs.322 (PMC7924708; doi:10.7717/peerj-cs.322)
Supplement: Supplemental Information 4 [file peerj-cs-06-322-s004.zip › argos3-epuck/tech_report/IridiaTr2015-004r004.pdf]

Université Libre de Bruxelles

Institut de Recherches Interdisciplinaires
et de Développements en Intelligence Artificielle

Software Infrastructure for E-puck (and TAM)

L. Garattoni, G. Francesca, A. Brutschy,
C. Pinciroli, and M. Birattari

IRIDIA – Technical Report Series

Technical Report No.
TR/IRIDIA/2015-004

July 2015
Last revision: October 2016

IRIDIA – Technical Report Series
ISSN 1781-3794

Published by:

IRIDIA, *Institut de Recherches Interdisciplinaires et de Développements en Intelligence Artificielle*
Université Libre de Bruxelles
Av F. D. Roosevelt 50, CP 194/6
1050 Bruxelles, Belgium

Technical report number TR/IRIDIA/2015-004

Revision history:

TR/IRIDIA/2015-004.001	July 2015
TR/IRIDIA/2015-004.002	February 2016
TR/IRIDIA/2015-004.003	October 2016
TR/IRIDIA/2015-004.004	October 2016

The information provided is the sole responsibility of the authors and does not necessarily reflect the opinion of the members of IRIDIA. The authors take full responsibility for any copyright breaches that may result from publication of this paper in the IRIDIA – Technical Report Series. IRIDIA is not responsible for any use that might be made of data appearing in this publication.

Software Infrastructure for E-puck (and TAM)

Lorenzo Garattoni, Gianpiero Francesca, Arne Brutschy,
Carlo Pinciroli, and Mauro Birattari

1 Introduction

We present and describe an infrastructure that provides researchers an integrated environment for swarm robotics experiments. The infrastructure includes both a simulation framework in which researchers can create and develop their experiments and the physical devices on which those experiments are finally deployed.

When designing a swarm robotics system, the main problem that researchers must face is the definition of individual rules that will result in the expected collective behavior. As deriving the rules that should guide the single robots from the collective requirements is difficult and time-consuming, researchers generally use simulation tools to carry out this task. The design of the appropriate control software for robots is performed in a simulated environment through a trial-and-error process (or through automatic design techniques). One of the most widespread swarm robotics simulators is ARGoS [5]. ARGoS offers a modular architecture that is easy to extend and a really efficient simulation, even with large groups of robots.

Once the user is satisfied by the results obtained in the simulated environment, the goal is finally to deploy the solution on the physical robots with little effort. ARGoS provides the tools to upload and execute the same control software directly on the robots.

In this report, we describe an infrastructure composed by the e-puck robot [4] and a device for task abstraction called TAM [2]. The two platforms have been designed, customized, programmed, and integrated in the simulation of ARGoS such that researcher are provided a fully integrated environment for the development of swarm robotics experiments.

2 E-puck

The e-puck [4] is a small wheeled robot developed as an open tool for education and research purposes. Its main advantage over the competitors is the relatively low cost and thus a broad community of users. The base version of the e-puck features a limited set of sensors and actuators. Around the circular body of the robot 8 infra-red transceivers are positioned to perceive the presence of obstacles or the intensity of the environmental light. Other sensors are a color camera at the front of the robot, a microphone and a 3-axis accelerometer. The actuators of the base e-puck are the motors of the two wheels, which can be set to produce a speed between 0 and 18 cm/s, a ring of 8 red LEDs and a

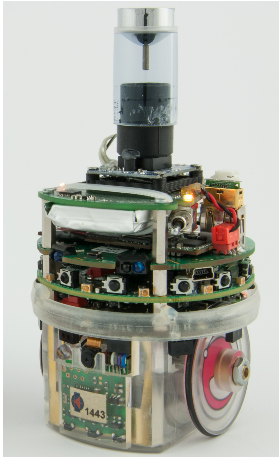

Figure 1: E-puck extended with range and bearing, Linux extension board and omni-directional camera

speaker. Additionally, the e-puck features a bright LED at the front. Given the very limited capabilities of the basic model, the e-pucks used at IRIDIA have been equipped with some extension boards. In particular, each e-puck features a ground sensor, which allows the robots to perceive the gray-scale color of the ground on which they navigate, a range and bearing device that enables local communications between robots (see Section 3), an omni-directional camera that provides a 360 degree view of the surroundings, and an embedded computer running Linux that enhances the computational power. The Linux extension board[1] features an ARM[2] processor and adds all the potentials of a computer running Linux, among which the possibility of using a USB dongle to connect to a Wifi network and the opportunity to add an additional board that integrates 3 RGB LEDs. Figure 1 shows the final configuration of the e-puck. The complete software model of the e-puck for ARGoS can be downloaded from `https://github.com/lgarattoni/argos3-epuck.git`.

2.1 E-puck firmware architecture

The addition of extension devices required a re-design of the software running on the e-pucks. Because of the hardware configuration, the sensors and actuators included in the basic model of the e-puck are controlled by a PIC microcontroller (dsPIC 30F6014A), which is programmed in the C language, while the additional sensors and actuators can be controlled directly from the Linux embedded computer. The ARM processor of the Linux board can access the ground and sensor and the range and bearing via I2C serial bus, while the omni-directional camera is accessed as a USB device. The PIC and the ARM processor are connected through a UART serial bus.

The goal of the re-design was to create a completely integrated infrastructure with ARGoS. Thanks to this infrastructure, researchers can develop control software for e-pucks in a simulated environment and then execute the same

[1] http://www.gctronic.com/doc/index.php/Overo_Extension
[2] http://www.arm.com/

code without modifications on the physical robots. For this reason the software running on the Linux embedded computer was written in C++, which is the language used to program the core of ARGoS as well as the control software for robots. Another requirement in the design of the e-puck software architecture was that, just like in ARGoS, the control of the robot must be performed in a loop composed of three phases executed in sequence: *sense*, *control*, and *act*. In the sense phase the sensors' readings are gathered and made available for the control phase, when a step of the control logic defined by the researcher is executed. The result of the control phase is a set of new values for the robot actuators, which are finally maneuvered in the act phase.

Given the above requirements, the resulting software architecture is shown in Figure 2. Its implementation can be found in the real-robot package of the e-puck model for ARGoS [1].

The software architecture is divided in two main components: the low-level loop written in C and executed on the PIC of the e-puck, and the main loop (real e-puck main) written in C++ that encapsulates the user-defined control software and is executed on the ARM processor of the Linux board. The two software components communicate with each other through a UART serial link. A complete cycle of control is composed of these steps:

1. The Linux board reads the sensors directly accessible (omni-directional camera, range and bearing sensor, ground sensor).

2. The PIC reads the value of every sensor directly connected (8 proximity sensors/light sensors, microphone, accelerometer).

3. The PIC sends the read sensor values to the Linux board through the serial link.

4. Once the values of every sensor is available, the control passes to the control step defined by the user. Depending on input values and the logic defined in the control step, new values for the actuators are set.

5. The Linux board communicates back to the PIC the new values for maneuvering the actuators of the basic e-puck (wheels, 8 red LEDs, speaker).

6. The PIC maneuvers the robot according to the new values.

7. The Linux board uses the new values to maneuver the actuators directly connected (range and bearing actuator, RGB LEDs).

These steps are repeated in the main loop every 100 ms, which is the default time step duration in ARGoS. The main loop also gives the start and the end commands to the PIC by sending specific messages through the serial bus. By keeping track of the state of the communication, the main loop can also handle and recover possible malfunctions.

2.2 E-puck in ARGoS

As mentioned previously, the goal of this work was to create an infrastructure that allows researcher to design and develop their robotic experiment in a simulated environment and then smoothly port the code developed on the

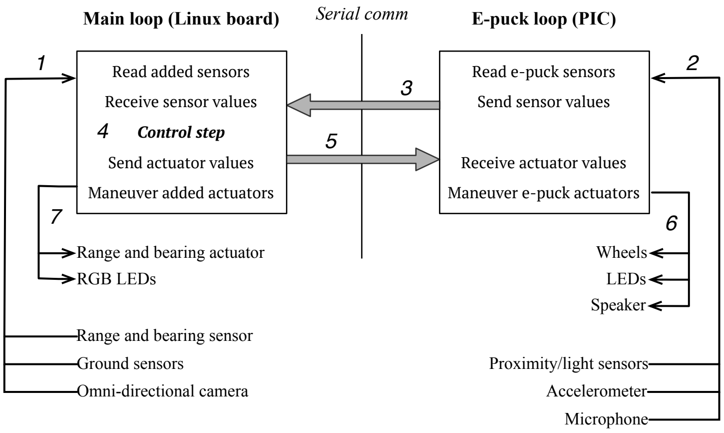

Figure 2: Steps of a cycle of control of the e-puck software architecture

real platforms without modification. To achieve this goal, the hardware and software details described in the previous section have been made completely transparent to the user, who must simply use an abstract control interface to access sensors and actuators. The same control interface is implemented by both the simulated and the real robot packages.

Real-robot package

In the real e-puck package the control interface is implemented to hide the low level details of the hardware and make them transparent to the user. Each sensor and actuator has its own implementation. In particular, sensors can be divided in two categories: the ones directly accessible from the Linux board and the ones accessible only through the PIC. In the former case, sensors are directly read and the data can be post-processed before being made available to the user control software (e.g. the images acquired from the omni-directional camera are processed to extract areas of neighboring pixels in the same range of color spectrum, these areas are aggregated in structures called blobs, and the blobs are provided to the user with information about relative position, size and color). For the latter type of sensors the data is acquired by the PIC and then insert in a data structure (real epuck base) that is sent back to the main loop through the serial bus. Once the communication is completed, the implementation of each sensor can read its own data from the received structure. Figure 3 shows the e-puck software architecture integrate in ARGoS.

The real e-puck package requires to be compiled for the specific hardware architecture before being transferred on the robot from a personal computer. Information and a step-by-step installation guide can be found in the documentation folder of the e-puck model for ARGoS [1].

Simulation package

In the simulation package the control interface is implemented to read and modify the simulated 3D space created by ARGoS. The simulated 3D space is composed by a set of data structures that contains the complete state of the simulation (position and orientation of robots and obstacles, for instance). The

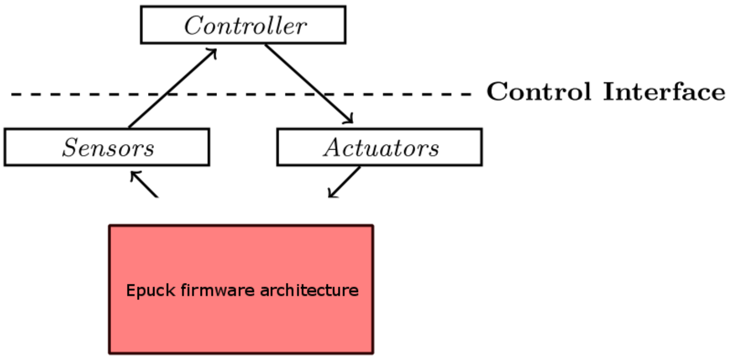

Figure 3: The architecture of the real e-puck package integrated in ARGoS

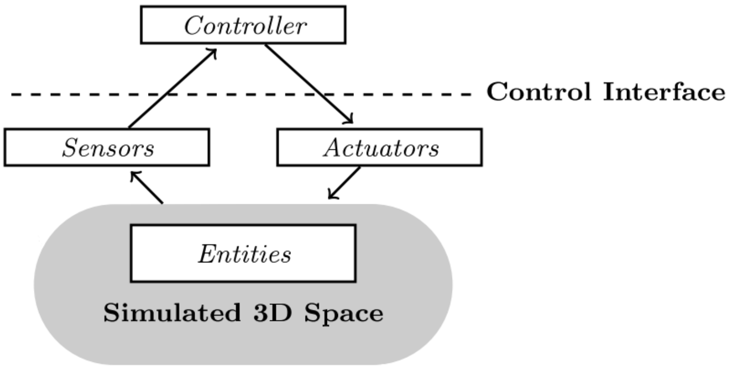

Figure 4: The architecture of the e-puck simulation package

state of the different functional components of the robots, i.e. the state of the range and bearing device (RAB), is also stored in this set of data structures. ARGoS keeps the simulated 3D space organized in items called entities. Different components' states are stored in different specialized entities (e.g. *RAB-equipped entity*).

Sensors and actuators are plug-ins that access the state of the simulated 3D space. Sensors have read-only access to the space, while actuators can modify it. Sensors and actuators are designed to only access the necessary specialized entities. A diagram of the e-puck simulation package in ARGoS is reported in Figure 4.

The simulation of sensors and actuators can be tuned by means of several parameters that the user can specify in the ARGoS configuration file of an experiment. Sensors and actuators can be used in an ideal version or in more realistic versions — by adding noise or assigning realistic values to other parameters that modify the behavior of the specific sensor/actuator. For a list of all the parameters available in ARGoS to modify the behavior of a specific device, the user can query the simulator by executing the following command:

```
$ argos3 -q sensor/actuator_name
```

Besides the implementation of real-robot and simulation packages, the e-puck

model for ARGoS contains a *testing* folder in which the user can find simple example controllers that can be compiled and execute both for simulation and physical robots.

Calibration
One of the most delicate aspect to consider when passing from simulation to the physical robots is the difference between sensors. Every detector is different from the others, sensor readings can be more or less noisy depending on environment conditions and other factors that are impossible to model in a simulated world. For these reasons, users can perform a preliminary step called *calibration* before executing their control software on the physical robots. The goals of the calibration phase are (i) reducing the effect of differences between sensors; (ii) normalizing the raw values given by the sensors in such a way that they are consistent to the ones used in simulation; and (iii) adjusting the ranges of normalization depending on the environmental conditions chosen for the particular experiment.

The e-puck model for ARGoS provides a calibration controller in the *testing* folder. When given the name of the sensor to calibrate and executed, the controller prints all the instructions that the user must follow in order to proceed with the calibration. The list of sensors that can be calibrated can be found in the `calibration_epuck.xml` configuration file for the calibration controller, in the *testing* folder. An example of execution for the calibration of the proximity sensors is as follows:

```
$ ./calibration -c calibration_epuck.xml -i proximity
```

The execution produces a xml calibration file that contains the range of each proximity sensors over which the raw values will be normalized. When using a sensor in their experiments, users must specify whether they want to use its calibrated version (by specifying the calibration file produced by the calibration controller) or the raw version. This is done in the xml configuration file of the experiment.

3 Range and bearing

Among the devices of the e-puck, one of the most powerful is certainly the range and bearing (RAB) [3]. The range and bearing enables local communication between the robots. The device is equipped with 12 infra-red emitters and 12 infra-red receivers through which it can send and receive messages. Upon reception of a message, the range and bearing is able to measure the relative distance (range) and direction (bearing) of the robot emitter.

3.1 Range and bearing firmware

To create the carrier of the emission module, the range and bearing firmware starts a pulse-width modulation (PWM) timer with a period of $1.09\,\mu$s. The timer generates an interrupt every $100\,\mu$s. The interrupt handler takes the buffered data and sends it to the hardware gates for its transmission. A Manchester code is implemented to allow any data sent at a certain distance to be received with the same intensity by the receiver. The transmission module can

be configured in order to send the same or different data from different emitters (single emitters can be disabled as well). Once a transmission request is sent by a master to the device, the communication module decomposes the data for the different emitters with a preamble (6 bits), the payload (16 bits in the original version) and a CRC (4 bits).

The reception module continuously listens if a message arrives. When it detects the preamble of a frame in one of the receivers, the module receives the payload and CRC. If the CRC check passes, the frame is stored in a buffer. If peak detectors of different sensors receive the same signal at the same time, the information is used to calculate the relative direction and distance to the emitter. These two values are also stored in a buffer available to the master.

The interface between the master and the device is given by registers that the master can write in order to send commands, parameters or data to the board, or read to receive responses or data back from the board.

The original firmware only supports messages with a fixed payload size of 16 bits. In many applications this may represent an important constraint, as 16 bits may be too many or too few. To meet the requirements of various experimental conditions, and to prevent the need of implementing costly communication protocols, the range and bearing firmware has been upgraded to support extended and parametric payload sizes. The available payload sizes are now 8, 16, 24 and 32 bits. The modification involved the extension of the data structures responsible of storing the data to send and receive, and a run-time allocation of this structures and management of the indexes over the structures, depending on the payload size sent by the master to the board. An additional register was added to the interface to let the master set the chosen payload size. Four other registers were added to read or write the two additional bytes of payload.

3.2 Range and bearing in ARGoS

The integration of the e-puck range and bearing in ARGoS follows the same principles described in Section 2.2. Because of its importance and its many different functions, it is anyway worth describing both the implementation for the physical robots and the simulation.

Real-robot package

The implementation of the range and bearing device for the real e-puck has the goal of creating an interface between the user-defined control logic and the firmware of the device described in the previous section. As the range and bearing is both an emitter and receiver of messages, both an actuator and a sensor have been implemented.

To realize a bidirectional communication, the user must add the range and bearing in both *actuators* and *sensors* subtrees of the xml configuration file of his/her experiment. Among the parameters that can be assigned to the range and bearing in the xml configuration file, the `data_size` sets the payload size that the device will use, expressed in bytes. The value of this parameter must match for the actuator and the sensor and is sent to the range and bearing firmware at initialization time by writing the designated register. Once the initialization is over and the Linux board and the range and bearing are aligned to the same payload size, the real function of sensor and actuator can begin.

To decrease the probability of message loss, the sensor starts a thread whose task is to regularly poll the range and bearing device asking for new messages received. Messages are added to a buffer that is read by the main loop once every control step (along with the distance and the direction of emission). Indeed, the main loop is busy handling the communication with the e-puck PIC for the most part of the 100 ms cycle of control. Limiting the reception of messages to the remaining part of the control cycle would compromise the performance by increasing the number of lost messages. The raw value of range given by the device represents a measure of signal strength between 0 and 4096. In the sensor, a conversion is performed to return a more meaningful value, i.e. a value of distance is centimeters. The conversion function, which was derived from experimental data, is reported below:

$$distance = gain * e^{\alpha + (\beta * strength)} \quad (1)$$

Where $\alpha = 9.06422$, $\beta = -0.00565$ and $gain = 0.08674$.

Similar considerations can be done regarding the actuator. Once the control logic defines a message to send, the message should be available to the other robots for the longer time-span possible, until a new message is set. Therefore, the actuator should keep invoking the range and bearing for the whole duration of the control cycle (one request to the range and bearing corresponds to a single emission of the message). This is achieved with a separated thread, which requests the emission of the message set by the control logic once every 20 ms (different periods were tested, with shorter periods causing an overload on the I2C bus). The actuator code is also responsible of keeping track of the state and the data assigned to the different emitters. The control logic might perform multiple data-emitter assignments during a control step, sometimes conflicting with each other. The actuator uses only the resulting final state of the emitters and, depending on this state, optimizes the number of operations on the I2C bus to control the range and bearing device. For instance, setting the same payload to each individual emitter would produce 12 write operations on the bus. By recognizing that the same data has been set for all emitters, the actuator produces instead a single operation on the bus that requests the emission of a payload from all emitters.

Simulation package

The simulated range and bearing allows e-pucks to perform situated communication in the simulated environment created by ARGoS. As explained in Section 2.2, the implementation of sensors and actuators provides an interface between the user-defined control logic and the simulated 3D space of ARGoS. Exactly like on the real robots, the simulated range and bearing comprises both a sensor and an actuator. The implementation of the range and bearing is associated to the range-and-bearing medium. In ARGoS, media are entities responsible of dispatching messages or other information from one equipped entity to one or multiple others. To be able to use the range and bearing, it is hence required to add a range-and-bearing medium to the `<media>` section of the xml configuration file of the experiment.

Regarding the range-and-bearing actuator, besides the aforementioned data size, it is possible to tune the range of transmission by assigning the desired value, in meters, to the `range` parameter in the xml file.

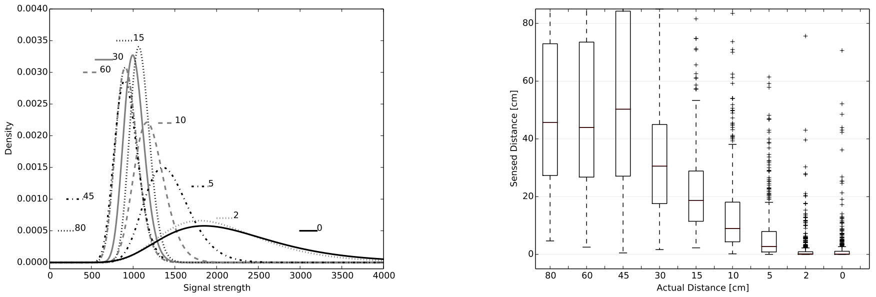

(a) (b)

Figure 5: In 5a the log-normal distributions for 9 fixed distances. In 5b, boxplot distributions of sensed distance against actual distance when real-range noise is active

The behavior of the sensor is instead more complex and configurable. If the user does not specify any additional parameter, the behavior of the sensor will be ideal: it will receive any message sent by robots in its range at a given time-step and it will be able to measure precisely the value of distance and bearing of each message. To simulate more accurately the behavior of the physical range and bearing device, several parameters are available. The number of messages that can be received in a single control cycle can be limited to the value of the `max_packets` parameter. Gaussian noise can be added to the values of range and bearing, with a standard deviation specified by the `noise_std_dev` parameter. The loss of packets can be simulated, by setting the loss probability to `loss_probability`. Finally, given the very noisy nature of distance measure when using physical robots, it is possible to specify whether to reproduce this noise in simulation by activating `real_range_noise`.

When `real_range_noise` is activated, the measures performed on physical robots are used to produce noise on the value of distance. The procedure adopted was as follows:

1. The distribution of signal strength values was calculated for several fixed distances on physical robots. The distribution of these values was approximated by a log-normal distribution for each distance. In the sensor, a look-up table contains the μ and σ parameters for the log-normal distribution corresponding to each distance. Figure 5a shows the log-normal distributions for the 9 distances.

2. The parameters μ and σ are calculated by interpolating the actual distance measured in ARGoS with the physical-robot data stored in the look-up table.

3. The simulated value of signal strength is calculated by drawing a number from the log-normal distribution obtained from the previous point.

4. The value of signal strength is converted in distance with Equation 1. The boxplots in Figure 5b show the distribution of final sensed distance in function of the actual distance between the robots in the simulation.

From he data gathered in several physical-robot experiments, a good configuration for the simulated range and bearing sensor to reproduce the behavior of the real sensor is the following:

```
                <epuck_range_and_bearing
implementation="medium" medium="rab"
loss_probability="0.3" check_occlusions="true"
real_range_noise="true" max_packets="5"
noise_std_dev=".01"/>
```

Calibration
The calibration of the range and bearing device can be done with the same calibration controller provided in the *testing* folder of the e-puck model for ARGoS. For the time being, only the value of distance read by the sensor can be calibrated. The calibration requires two robots placed at a distance of 20 cm between each other, running the same calibration controller. The robots make an average of the signal strength received over 100 messages. Thanks to this value, they calculate the correct *gain* parameter for the function reported in Equation 1 when $distance = 20$. The *gain* obtained will be used to convert (through Equation 1) the values of signal strength detected by the range and bearing sensor during robot experiments.

4 TAM

The infrastructure realized for simulated and physical-robot experiments comprises another device, the task abstraction module (TAM) [2]. The TAM represents single-robot, stationary tasks to be performed by an e-puck. The goal of the TAM is to abstract from details specific to task execution that are not the focus of an experiment. The TAM allows researchers to omit details on task execution and focus on the relevant properties of the tasks such as their logical interrelationships. Complex multi-robot tasks can be abstracted by groups of TAMs. First, a complex task is modeled as the set of its constituent single-robot subtasks and their interrelationships. Second, each single-robot subtask is abstracted by a single TAM and the behavior of the TAMs is coordinated such that it reflects the interrelationships identified by the model.

In the remainder of this section we describe the physical implementation of the TAM, the control framework and finally the integration with ARGoS.

4.1 TAM architecture

A TAM has the shape of a booth, into which an e-puck can enter. The length of every dimension of the TAM is 12 cm. The TAM is equipped with two light barriers, three RGB LEDs, and a IR transceiver for communication.

The TAM announces the task it abstracts by using its colored LEDs. An e-puck can perceive the LEDs of a TAM using its omni-directional camera. If the e-puck decides to perform the task represented by the TAM, it moves into the TAM by following the colored LEDs. The TAM can detect the presence of the robot by using its light barriers. Upon detection of the robot, the TAM reacts according to a user-defined logic; for example, by changing the color of

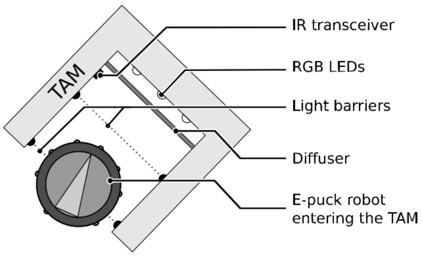

(a) Conceptual drawing of the TAM

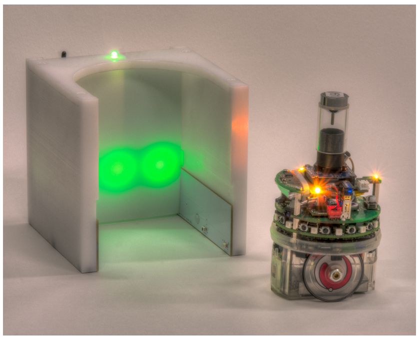

(b) A TAM and an e-puck robot

Figure 6: The conceptual drawing and physical realization of the TAM

its LEDs or by communicating with the robot. Communication between the TAM and the e-puck is realized using the IR transceiver and the e-puck library *IRcom*[3]. This communication enables experiments in which the behavior of the TAMs depends on the specific robots working on their tasks. Figure 6 shows a conceptual drawing of the TAM, and a real TAM with an e-puck robot.

The TAM features an XBee mesh networking module, which allows researchers to synchronize the behavior of multiple TAMs, enabling the representation of complex relationships between tasks and cooperative behaviors between robots. The XBee module can be configured to work on 4 different wireless channels, which allows up to four parallel experiments.

The TAM is open source under the *Creative Commons Attribution-Share-Alike 3.0 Unported License.*

The firmware of the TAM is based on Arduino, an open-source platform using an Atmel AVR micro-controller as central processor. The advantages of Arduino are the wide availability, large community, and relative ease of development compared to other embedded development platforms.

The goal in the design of the TAM's control framework was to create a centralized framework to control groups of TAMs. In this way, the TAM can be remotely controlled by a central computer, it can be used by the computer to gather experimental data, and it operates without being physically connected to the computer.

The control framework of the TAM is composed of two parts: the *firmware* based on Arduino running on each TAM, and the *coordinator*, running on the central computer. The latter is the software component that handles the wireless communication with all the TAMs connected, keeps the status of every TAM updated, and manages the relationships between the behavior of different TAMs. The *firmware* notifies all events and changes in sensory readings to the *coordinator*, and executes all commands that it receives in return. Commands and notifications are relayed using the wireless mesh network modules of the TAMs. The *coordinator* handles this exchange of commands/notifications and makes it transparent to the user, who can focus on the definition of the logic that controls the behavior of the TAMs.

[3]http://gna.org/projects/e-puck/

The software that composes the *coordinator* is programmed in Java[4] to ensure the maximum simplicity and portability. To set up an experiment, the user is required to define two Java classes: a *controller* and an *experiment*. The former is the software that the *coordinator* uses to control a single TAM. One instance of *controller* must be attached to each individual TAM. Similarly to a robot control cycle, the coordinator executes in a loop the *controller* step function, which at every execution takes the state of the TAM as input and produces commands for the same TAM depending on the user-defined logic. In the *experiment*, the user must attach a *controller* to each TAM and handle the logical interrelationships between TAMs.

All the material about the TAM, including schematics, firmware, coordinator, examples, and documentation can be be downloaded from `https://iridia-dev.ulb.ac.be/projects/iridia-tam/git`.

4.2 TAM in ARGoS

The software infrastructure would not be complete without the integration of the TAM in ARGoS. Because the *coordinator* and the control software are programmed in Java, there are some differences between the implementation of the TAM and the implementation of the robots in ARGoS. First, there is no real-robot implementation of the TAM directly in ARGoS, as the TAM's main loop of control is handled by *coordinator*, *experiments* and *controllers*, which are programmed in Java as described in the previous section. Second, to ensure the direct portability of the same control software from simulation to physical TAM, the simulation package provides a C++ wrapper for the Java code used on the physical device.

Simulation package

The goal of the simulated package is to wrap the Java software architecture of the real TAM in C++ entities that can be added to the ARGoS simulated 3D space. In this way, the user can create and test an experiment in simulation by implementing control software for e-pucks in C++ and control software for TAMs in Java. The burden of integrating everything in the same simulated world is handled by ARGoS. Once the user is satisfied with the results, the control software for both robots and TAMs can be executed on the real devices without modifications. The architecture of the TAM simulated package and its interactions with the physical TAM software architecture are shown in Figure 7.

Two Java classes *TAM* implement the same *TAM interface*, one used for the real TAM and the other one for the integration in ARGoS. When invoked by the *controller*, the *TAM* class used for the real TAM, on the left in Figure 7, relays commands to the *coordinator* or reads the state reported by the *coordinator* from the physical TAM. The implementation of *TAM* for ARGoS, on the other hand, simply keeps the internal state of the TAM and returns it or modifies it depending on the requests of the *controller*. The most important class for the integration with ARGoS, i.e. the one the realizes the wrapper for the Java code in C++, is *TAMs Controllable Entity*. This class is responsible of creating the *experiment*, instantiating a *TAM* object and a *controller* for each TAM added by the user to the experiment and binding each instance of *controller* to the right

[4]https://java.com/en/download/

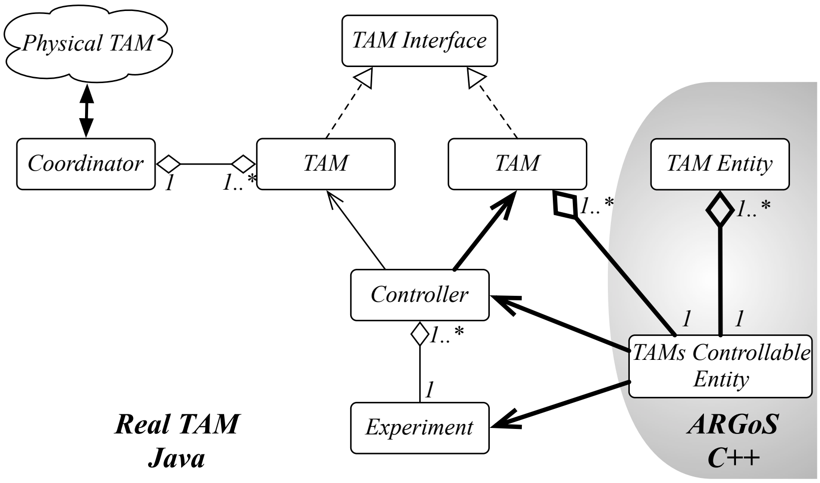

Figure 7: TAM software architecture. On the left side the Java classes for the real TAM. On the right side the C++ classes in ARGoS. The bold connections are the ones that realize the C++ wrapper in ARGoS

TAM. The interactions between the C++ code of *TAMs controllable entity* and the Java code of the TAM software infrastructure are realized through the JNI library[5]. The *TAMs Controllable Entity* is also responsible of managing the timing of the experiment by calling in a loop the step functions of *experiment* and *controllers*. Finally, after each iteration, the *TAMs Controllable Entity* takes the state of each *TAM* instance (which has just been modified by the step function of its *controller*) and updates the state of each corresponding *TAM entity* accordingly. The state of the *TAM entities* is used to materialize the TAMs in the 3D simulated space of ARGoS. The effects of events that change the state of a *TAM entity* in the simulation (a robot entering the TAM, for instance) are immediately reported to the corresponding *TAM* object's state, which will be used by the next step function call.

From the user's perspective, all these details are hidden. The user can focus on the implementation of the control logic for the TAMs by defining experiments and controllers. To add a TAM in the simulation, the user must insert it in the ARGoS xml configuration file, inside the `<arena>` section, as shown below:

```
<tams java_class_path="path/to/coordinator/bin"
      experiment_class="path/to/experiment"
      rab_medium="ircom_medium_name"
      led_medium="led_medium_name">
     <tam id="TAM01" movable="false">
          <body position="0,0,0"
                orientation="0,0,0" />
     </tam>
</tams>
```

[5]https://docs.oracle.com/javase/6/docs/technotes/guides/jni/

In the *coordinator* folder of the TAM material at `https://iridia-dev.ulb.ac.be/projects/iridia-tam/git`, the user can find an Eclipse project with all the Java code of the real TAM and examples of *experiments* and *controllers*. The *argos3* folder contains instead the code for the integration in ARGoS. The examples must be launched using the xml configuration files that can be found in the *testing* folder of the e-puck model for ARGoS [1].

5 Conclusion

In this report we have presented an infrastructure composed by e-puck robots and task abstraction modules. Besides the physical implementation of the devices, we integrated them in the ARGoS simulator and we designed the infrastructure such that the software developed in simulation can be directly ported on the real devices.

The infrastructure is still under development. Possible future work includes a better coexistence of the TAM in ARGoS with the possibility of programming it in C++ and the integration in the infrastructure of other useful tools for experiments, such as a tracking system [6]. A tracking system allows researchers to record and control the state of an experiment throughout its execution and to easily gather statistics. Additionally, a tracking system enables virtualization of aspects of the reality or parts of the robot itself. For example, researchers could virtualize sensors and actuators that are not mounted on the robots.

References

[1] E-puck model for ARGoS. `https://iridia-dev.ulb.ac.be/projects/argos3-epuck/git`.

[2] A. Brutschy, L. Garattoni, M. Brambilla, G. Francesca, G. Pini, M. Dorigo, and M. Birattari. The tam: abstracting complex tasks in swarm robotics research. *Swarm Intelligence*, 9(1):1–22, 2015.

[3] A. Gutierrez, A. Campo, M. Dorigo, J. Donate, F. Monasterio-Huelin, and L. Magdalena. Open e-puck range & bearing miniaturized board for local communication in swarm robotics. In *Robotics and Automation, 2009. ICRA '09. IEEE International Conference on*, pages 3111–3116, May 2009.

[4] F. Mondada, M. Bonani, X. Raemy, J. Pugh, C. Cianci, A. Klaptocz, S. Magnenat, J. Zufferey, D. Floreano, and A. Martinoli. The e-puck, a robot designed for education in engineering. In P. Gonçalves, P. Torres, and C. Alves, editors, *Proceedings of the 9th conference on autonomous robot systems and competitions*, volume 1, pages 59–65. IPCB, Castelo Branco, Portugal, 2009.

[5] C. Pinciroli, V. Trianni, R. O'Grady, G. Pini, A. Brutschy, M. Brambilla, N. Mathews, E. Ferrante, G. Di Caro, F. Ducatelle, M. Birattari, L. M. Gambardella, and M. Dorigo. ARGoS: a modular, parallel, multi-engine simulator for multi-robot systems. *Swarm Intelligence*, 6(4):271–295, 2012.

[6] A. Stranieri, A. Turgut, M. Salvaro, L. Garattoni, G. Francesca, A. Reina, M. Dorigo, and M. Birattari. Iridia's arena tracking system. Technical Report TR/IRIDIA/2013-013, IRIDIA, Université Libre de Bruxelles, Brussels, Belgium, 2013.
